# Publisher Correction: ITGB3-mediated uptake of small extracellular vesicles facilitates intercellular communication in breast cancer cells

**DOI:** 10.1038/s41467-020-18674-4

**Published:** 2020-09-15

**Authors:** Pedro Fuentes, Marta Sesé, Pedro J. Guijarro, Marta Emperador, Sara Sánchez-Redondo, Héctor Peinado, Stefan Hümmer, Santiago Ramón y Cajal

**Affiliations:** 1 Translational Molecular Pathology, Vall d’Hebron Research Institute (VHIR), Universitat Autònoma de Barcelona (UAB), Barcelona, Spain; 2 Spanish Biomedical Research Network Centre in Oncology (CIBERONC), Madrid, Spain; 3 Microenvironment & Metastasis Group, Molecular Oncology Program, Spanish National Cancer Research Centre (CNIO), Madrid, Spain; 4 Present Address: Tumor Biomarkers Group, Vall d’Hebron Institute of Oncology (VHIO), Barcelona, Spain

**Keywords:** Extracellular signalling molecules, Mechanisms of disease, Metastasis

Correction to: *Nature Communications* 10.1038/s41467-020-18081-9, published online 26 August 2020.

The original version of this Article contained an error in the spelling of the author Santiago Ramón y Cajal, which was incorrectly given as Santiago Ramón y. Cajal. This has now been corrected in both the PDF and HTML versions of the Article.

