# Supplementary material for: ITGB3-mediated uptake of small extracellular vesicles facilitates intercellular communication in breast cancer cells
Source: Nat Commun. 2020 Aug 26;11:4261. doi: 10.1038/s41467-020-18081-9 (PMC7450082; doi:10.1038/s41467-020-18081-9)
Supplement: Supplementary file 1 — Supplementary Information [file 41467_2020_18081_MOESM1_ESM.pdf]

Supplementary information

ITGB3-mediated uptake of small extracellular vesicles facilitates intercellular communication in breast cancer cells

Pedro Fuentes[1,2], Marta Sesé[1,2], Pedro J. Guijarro[1,2#], Marta Emperador[1,3#], Sara Sánchez-Redondo[4], Héctor Peinado[4], Stefan Hümmer[1,2*], Santiago Ramón y Cajal[1,2*]

Supplementary Figure 1

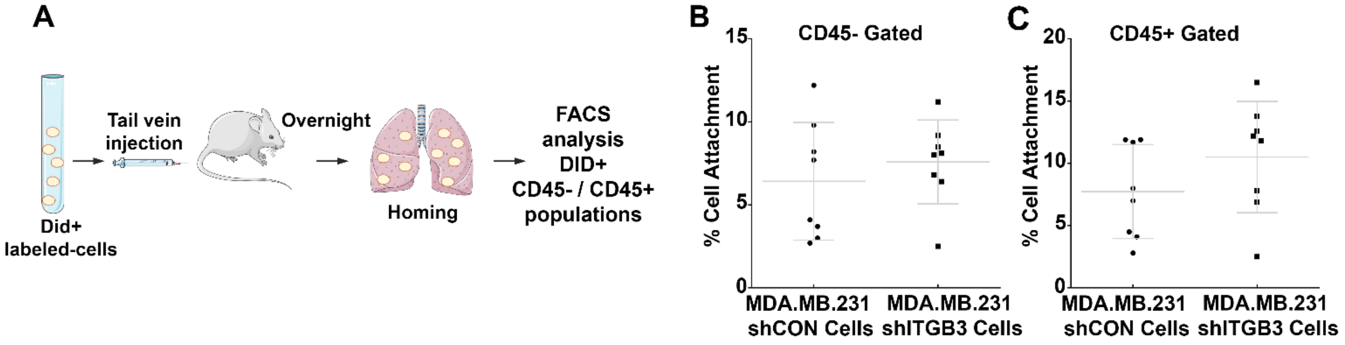

Supplementary Figure 1. shITGB3 MDA.MB.231 cells are capable of homing in the lung. A) Schematic workflow for lung homing tumour cells analysis. B-C) Flow cytometry analysis for DID+ labelled shITGB3 and shCON MDA.MB.231 cells gated for CD45- (left panel) and CD45+ (right panel). Source data are provided as a Source Data file.

(n= 8 animals). Data are represented as mean ± SD in b-c. Scheme created with BioRender.

Supplementary Figure 2

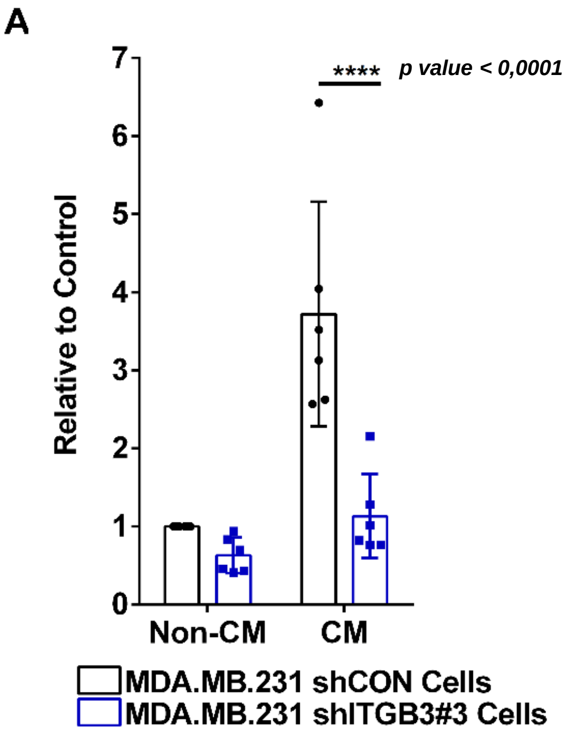

Supplementary Figure 2. Phenotype confirmation using a different ITGB3-targeting shRNA. A) Clonogenic cell growth analysis using shITGB3 #3. Source data are provided as a Source Data file.

(****p value<0.0001, n=6). Data are represented as mean ± SD. Statistical analysis including two-tailed unpaired Student's t-test data was carried out using GraphPad Prism 6.01.

Supplementary Figure 3

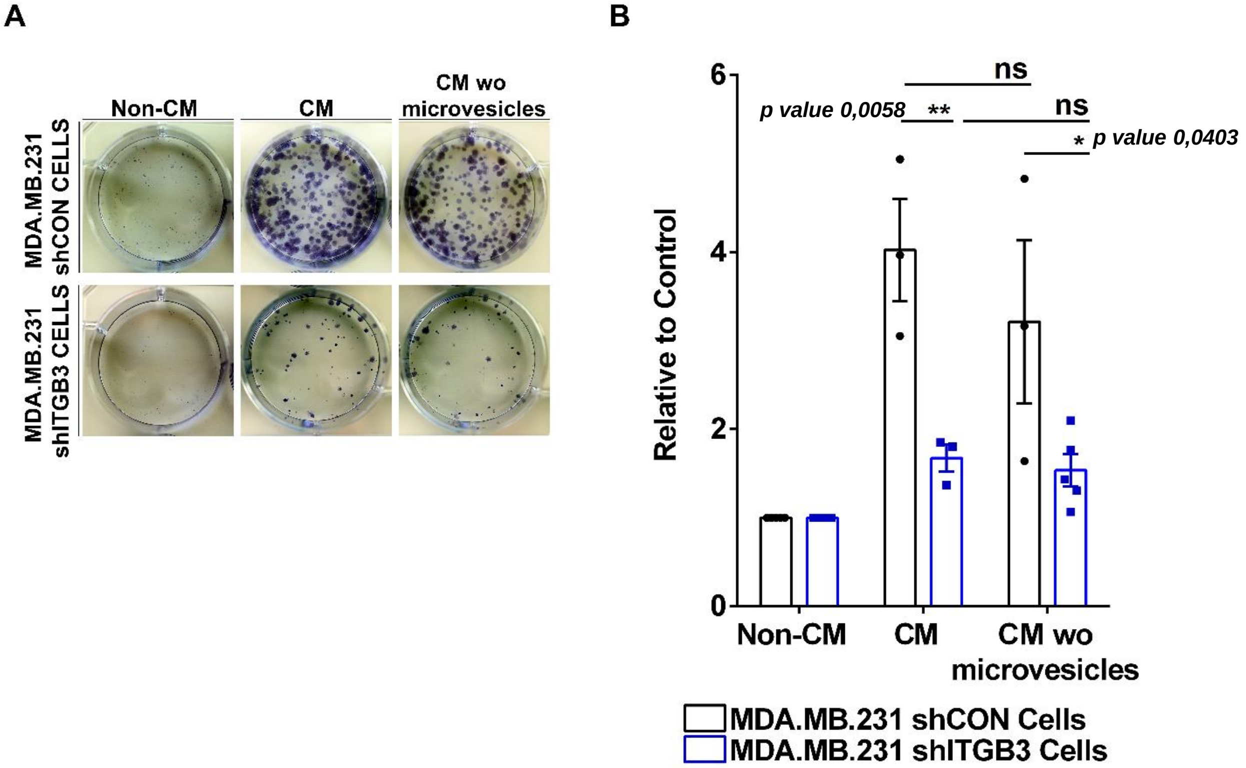

Supplementary Figure 3. Microvesicles are not able to sustain the clonogenic growth capacity. A) Representative pictures are shown for each cell population and condition. B) Conditioned medium (CM) was collected from exponentially growing MDA.MB.231 shCON cells. In the case of CM wo microvesicles, the same CM was split into two parts: one was used directly and the other was depleted of microvesicles by centrifugation at 10,000 g before use, to establish the response in clonal cell growth to the CM. Data are normalized to the non-conditioned control medium (DMEM). Source data are provided as a Source Data file.

(*p value<0.05, **p value<0.01, n=5). Data are represented as mean ± SD in b. Statistical analysis including two-way ANOVA multiple comparisons was carried out using GraphPad Prism 6.01.

Supplementary Figure 4

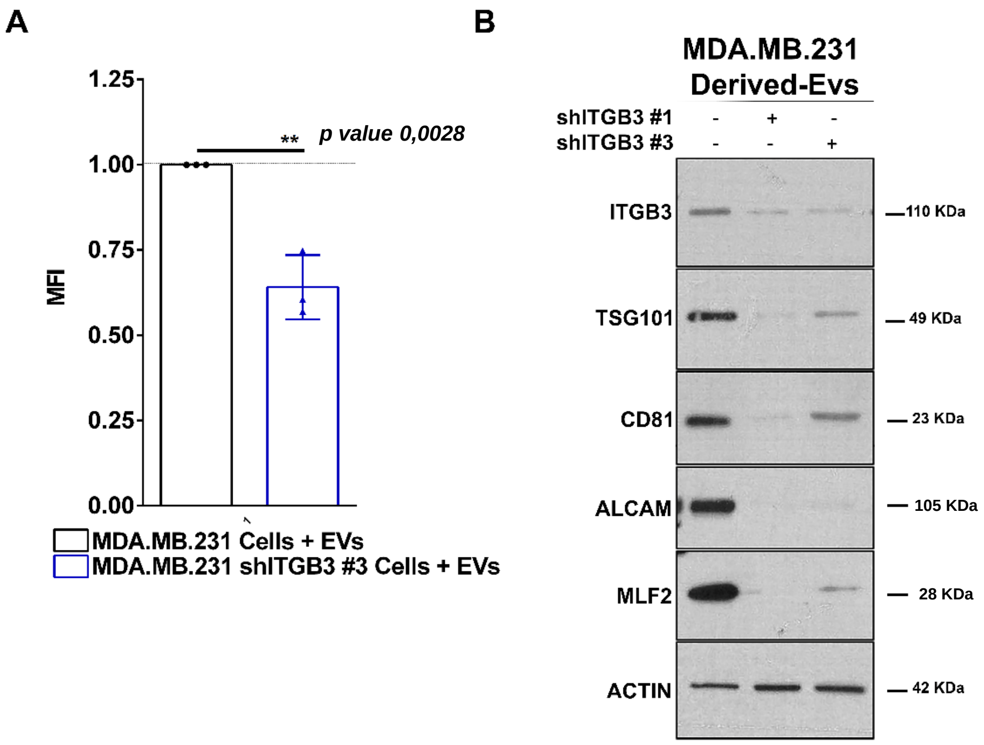

Supplementary Figure 4. Phenotype confirmation using a different ITGB3-targeting shRNA. A) Flow cytometry analysis for MDA.MB.231 shCON and MDA.MB.231 shITGB3 #3 cells after incubating with 2-5 µg/mL fluorescently-labelled EVs derived from MDA.MB.231 shCONTROL cells. B) Representative immunoblot analysis in two cell lines stably expressing different ITGB3-targeting shRNAs #1, #3. Source data are provided as a Source Data file.

(**p value<0.01, n=3). Data are represented as mean ± SD in a. Statistical analysis including two-tailed unpaired Student's t-test data was carried out using GraphPad Prism 6.01.

Supplementary Figure 5

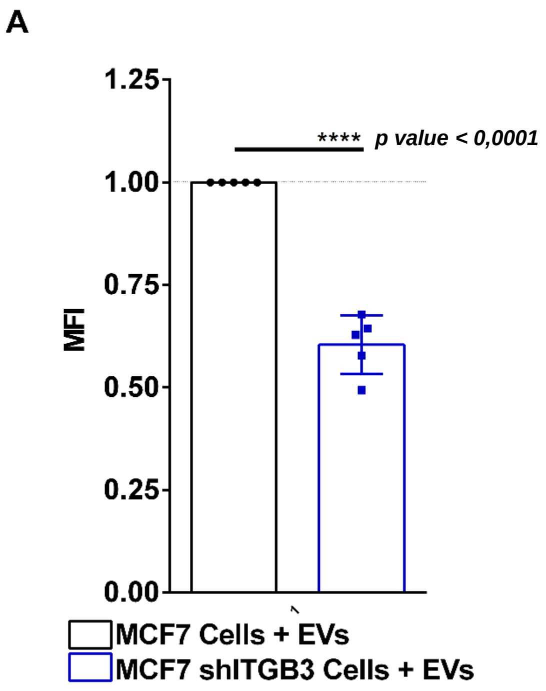

Supplementary Figure 5. Phenotype confirmation using a different breast cancer cell line. A) Flow cytometry analysis for MCF7 shCON and MCF7 shITGB3 cells after incubating with 2-5 µg/mL fluorescently-labelled EVs. Source data are provided as a Source Data file.

(****p value<0.0001, n=5). Data are represented as mean ± SD. Statistical analysis including two-tailed unpaired Student's t-test data was carried out using GraphPad Prism 6.01.

Supplementary Figure 6

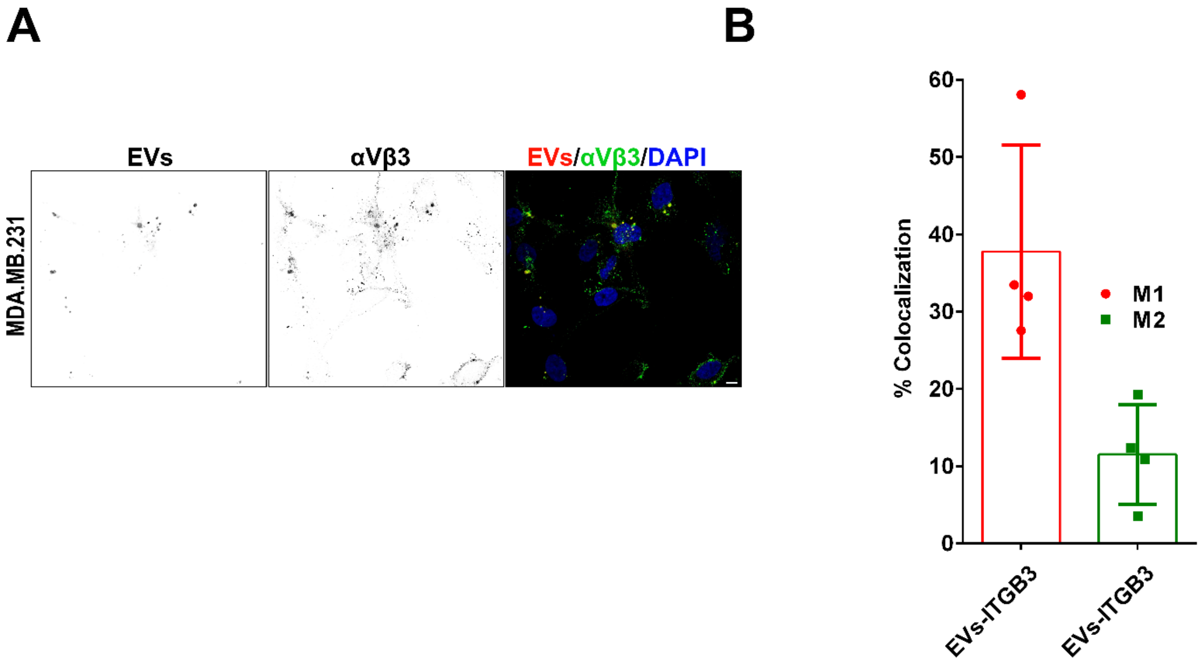

Supplementary Figure 6. Co-localization between EVs and ITGB3 at cellular membrane A) Representative confocal pictures of non-permeabilized MDA.MB.231 cells treated for 1h with PKH26-labelled shITGB3-derived EVs, fixed, and stained for αvβ3. B) Graphical representation of the Mander's coefficient values for determining co-localization between the different markers. (n=111 cells/condition). Bar represents 5µm. Source data are provided as a Source Data file.

Data are represented as mean ± SD in b.

Supplementary Figure 7

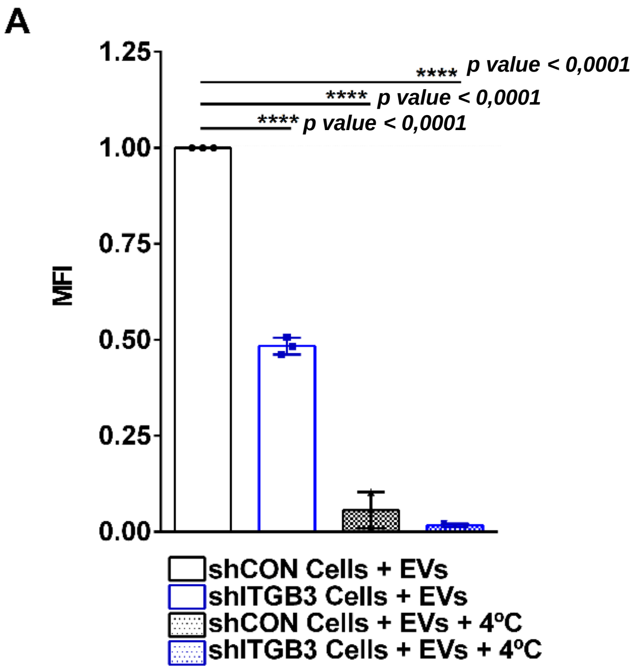

Supplementary Figure 7. EV uptake is an energy-dependent process. Flow cytometry analysis for MDA.MB.231 shCON and MDA.MB.231 shITGB3 cells after incubating with 2-5 µg/mL fluorescently-labelled EVs derived from MDA.MB.231 shCON cells at 37ºC and 4ºC. Source data are provided as a Source Data file.

(****p value<0.0001, n=3). Data are represented as mean ± SD. Statistical analysis including two-way ANOVA multiple comparisons was carried out using GraphPad Prism 6.01.

Supplementary Figure 8

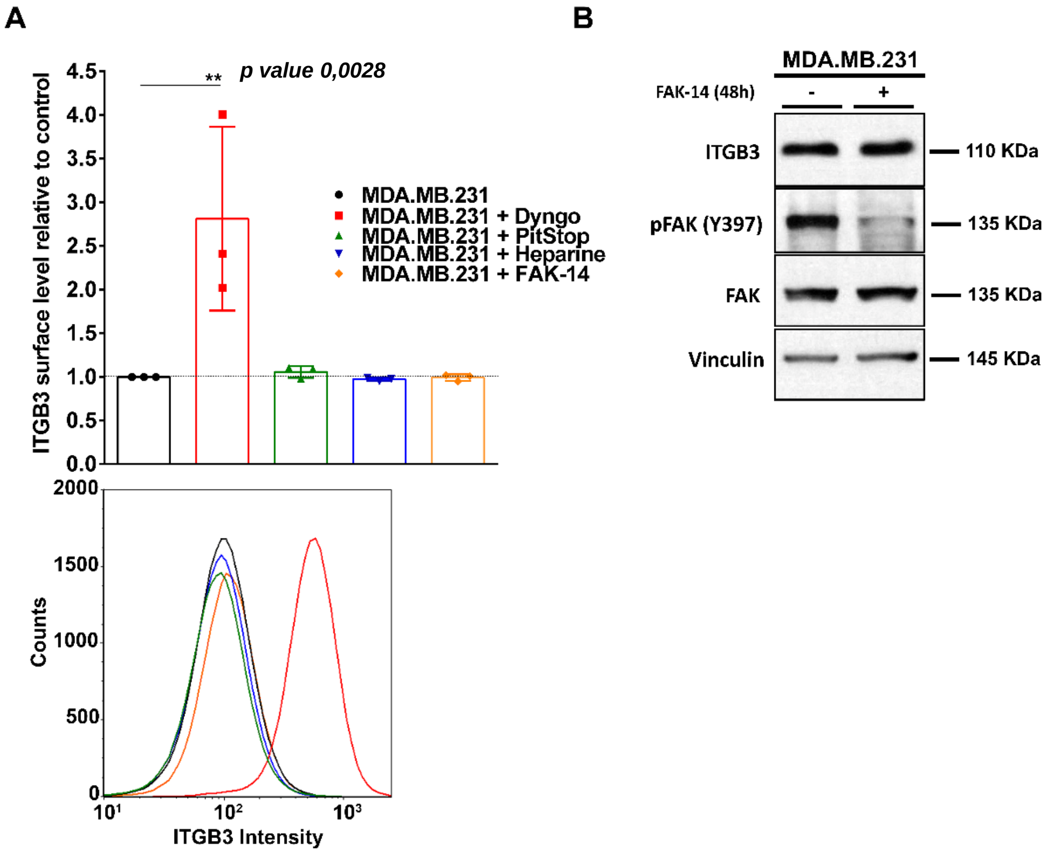

Supplementary Figure 8. Dynamin regulates ITGB3 localization. A) ITGB3 cell-surface level measured by FACS after 20 µM Dyngo4a, 10 µM Pitstop 2 or 10 µg/mL heparin for 30 minutes or with 15 µM FAK-14 for 14-16 hours. B) Representative immunoblot analysis of ITGB3 levels after 14-16 h of FAK-14 treatment. Source data are provided as a Source Data file.

(**p value<0.01, n=3). Data are represented as mean ± SD in a. Statistical analysis including two-tailed unpaired Student's t-test data was carried out using GraphPad Prism 6.01.

Supplementary Figure 9

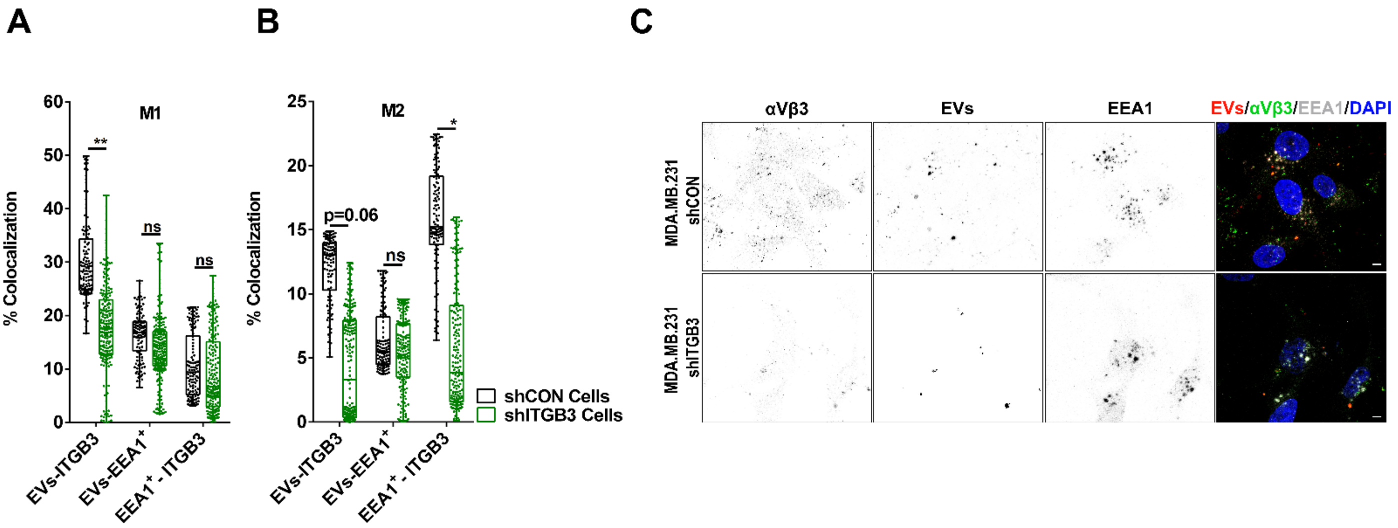

Supplementary Figure 9. Extracellular vesicles co-localizes with ITGB3 in endocytic-pathway structures. MDA.MB.231 shCON and MDA.MB.231 shITGB3 cells were treated for 1h with PKH26-labelled EVs, fixed, and stained for EEA1 and αvβ3 and EEA1 and LAMP1 respectively. A-B) Graphical representation of the Mander's coefficient values for determining co-localization between the different markers. (n= 75 cells/condition). C) Representative confocal pictures of MDA.MB.231 shCON and MDA.MB.231 shITGB3 cells. Bar represents 5µm.

(*p value<0.05, **p value<0.01). In a-b data are represented as boxplots using the MIN to MAX method. The middle line is the median, the lower and upper hinges correspond to the 25th to 75th percentiles. The upper whisker extends from the hinge to the largest value and the lower whisker extends from the hinge to the smallest value. Statistical analysis including two-way ANOVA multiple comparisons was carried out using GraphPad Prism 6.01.

Supplementary Figure 10

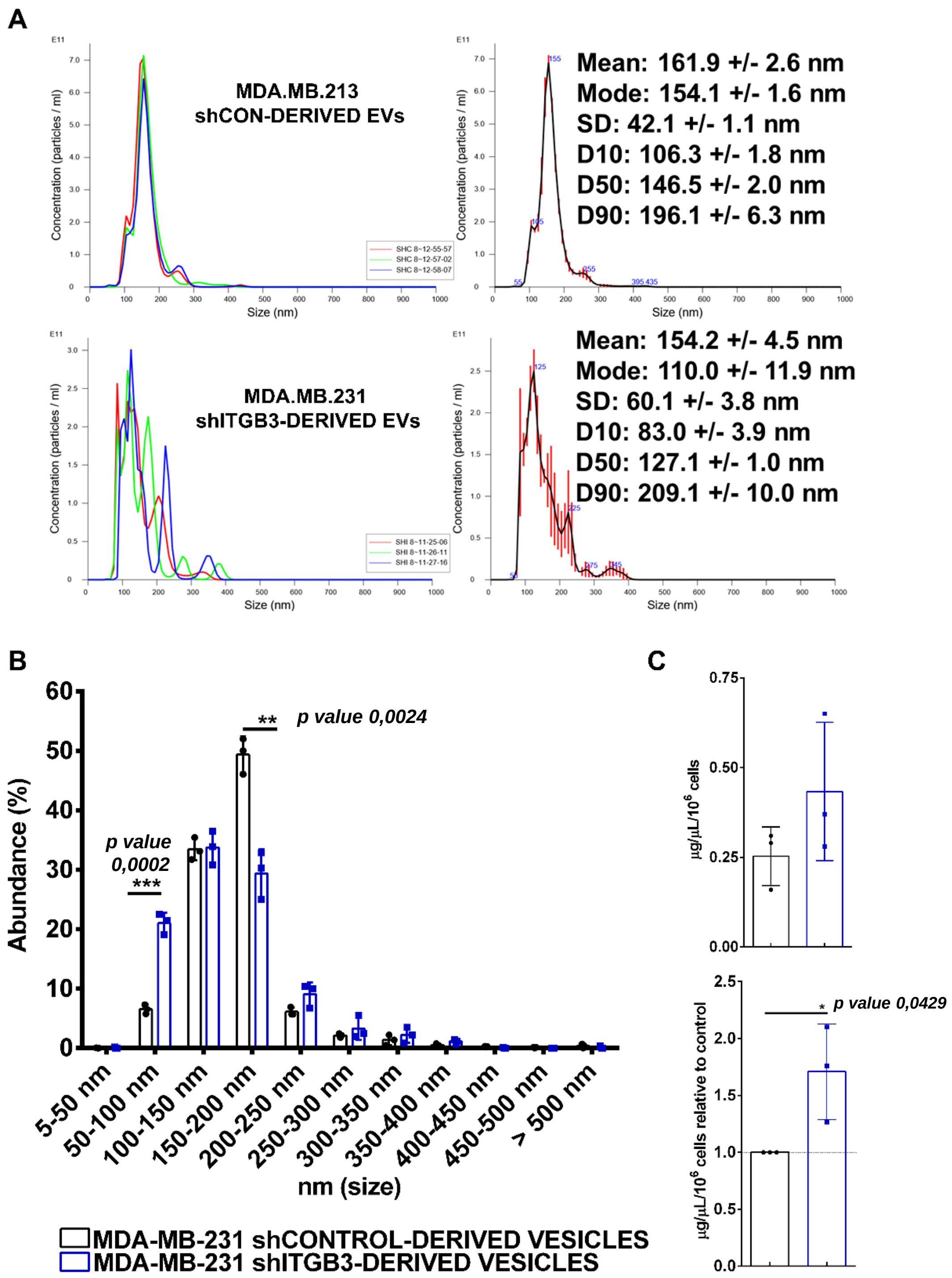

Supplementary Figure 10. Characterization of EVs isolated from MDA.MB.231 shCON and MDA.MB.231 shITGB3 cells. A) Nanosight Tracking Analysis (NTA) of MDA.MB.231 shCON- (upper) and MDA.MB.231 shITGB3-derived vesicles (lower). Average FTLA concentration/size (right). Error bars indicate +/-1 standard error of the mean. B) Vesicle size distribution based on NTA data. C) Protein concentration of isolated vesicle fractions normalized per million cells (upper) and normalized to shCON cells (lower). Source data are provided as a Source Data file.

(*p value<0.05, **p value<0.01, ***p value<0.001, n=3). Data are represented as mean ± SD in b-c. Statistical analysis including two-tailed unpaired Student's t-test was carried out using GraphPad Prism 6.01.

Supplementary Figure 11

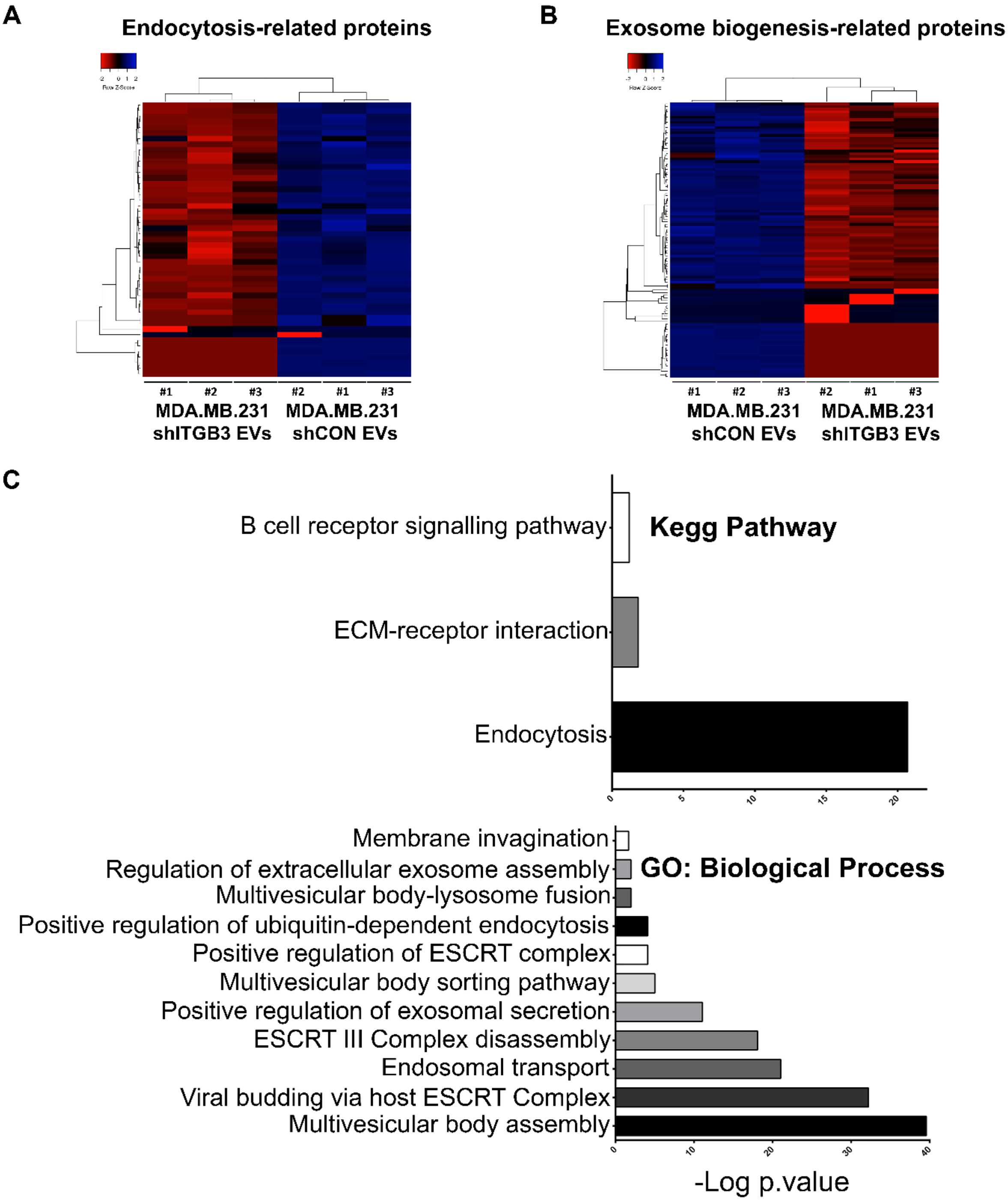

Supplementary Figure 11. Heat map representation showing the difference in the abundance of proteins with a described role in endocytosis (A) and exosome biogenesis (B). C) Gene ontology enrichment analysis of proteins with a log2 FC of -1 and a q.value < 0.05 in the comparison of proteins identified in the vesicle fraction of MDA.MB.231 shCON and MDA.MB.231 shITGB3 cells. In addition, proteins uniquely identified under either of the two conditions we included in the analysis. Pie charts are arranged by the mean –log10 p.values for the individual categories. Source data are provided as a Source Data file.

Supplementary Figure 12

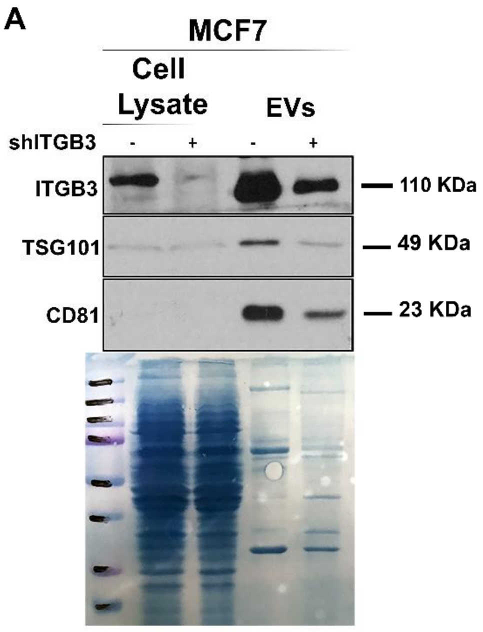

Supplementary Figure 12. Phenotype confirmation using a different breast cancer cell line. A) Representative immunoblot showing ITGB3, TSG101 and CD81 expression in MCF7 and MCF7 shITGB3 cells. Amido black staining (lower). Source data are provided as a Source Data file. Three independent experiments were performed with similar results.

Supplementary Figure 13

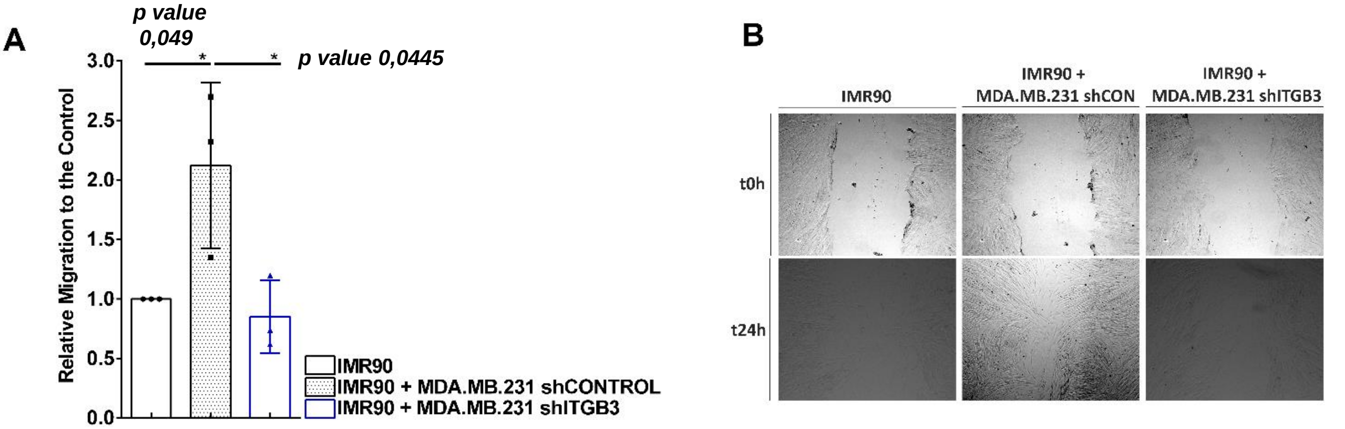

Supplementary Figure 13. IMR90 cells displayed an increased migratory capacity when co-cultured with MDA.MB.231 cells, but not when co-cultured with shITGB3 cells. A) Cell migration of IMR90 cell line using wound healing assay in a double chamber co-culture system. B) Representative pictures of wound healing assay at different time points and conditions. Source data are provided as a Source Data file.

(*p value<0.05, n=3). Data are represented as mean ± SD in a. Statistical analysis including two-tailed unpaired Student's t-test data was carried out using GraphPad Prism 6.01.

Supplementary Figure 14

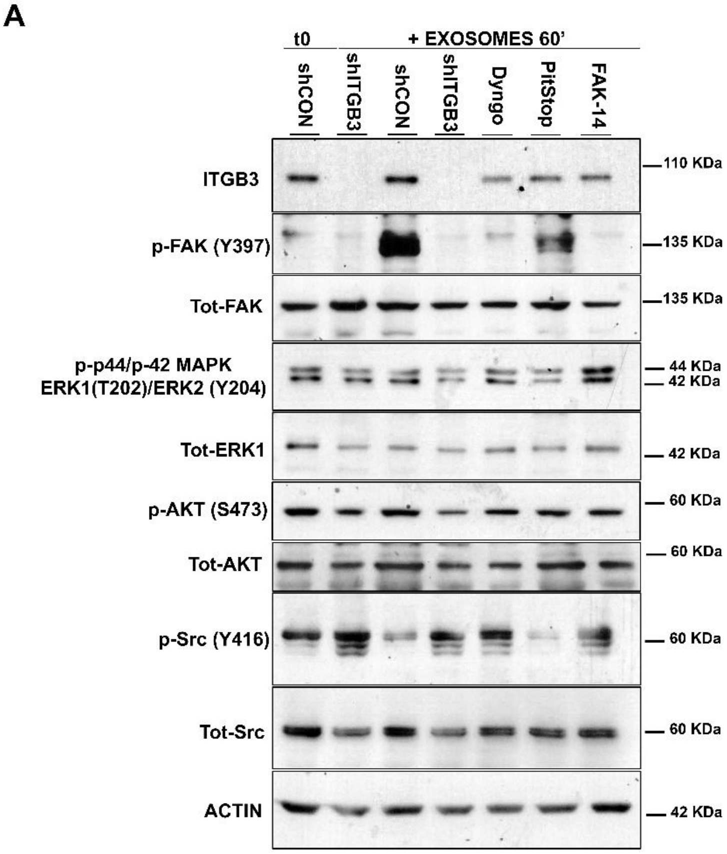

Supplementary Figure 14. Only FAK activity was stimulated by EV treatment among all tested signalling pathways reported to be downstream of ITGB3. A) Representative Western blot analysis. Source data are provided as a Source Data file. Three independent experiments were performed with similar results.

Supplementary Figure 15

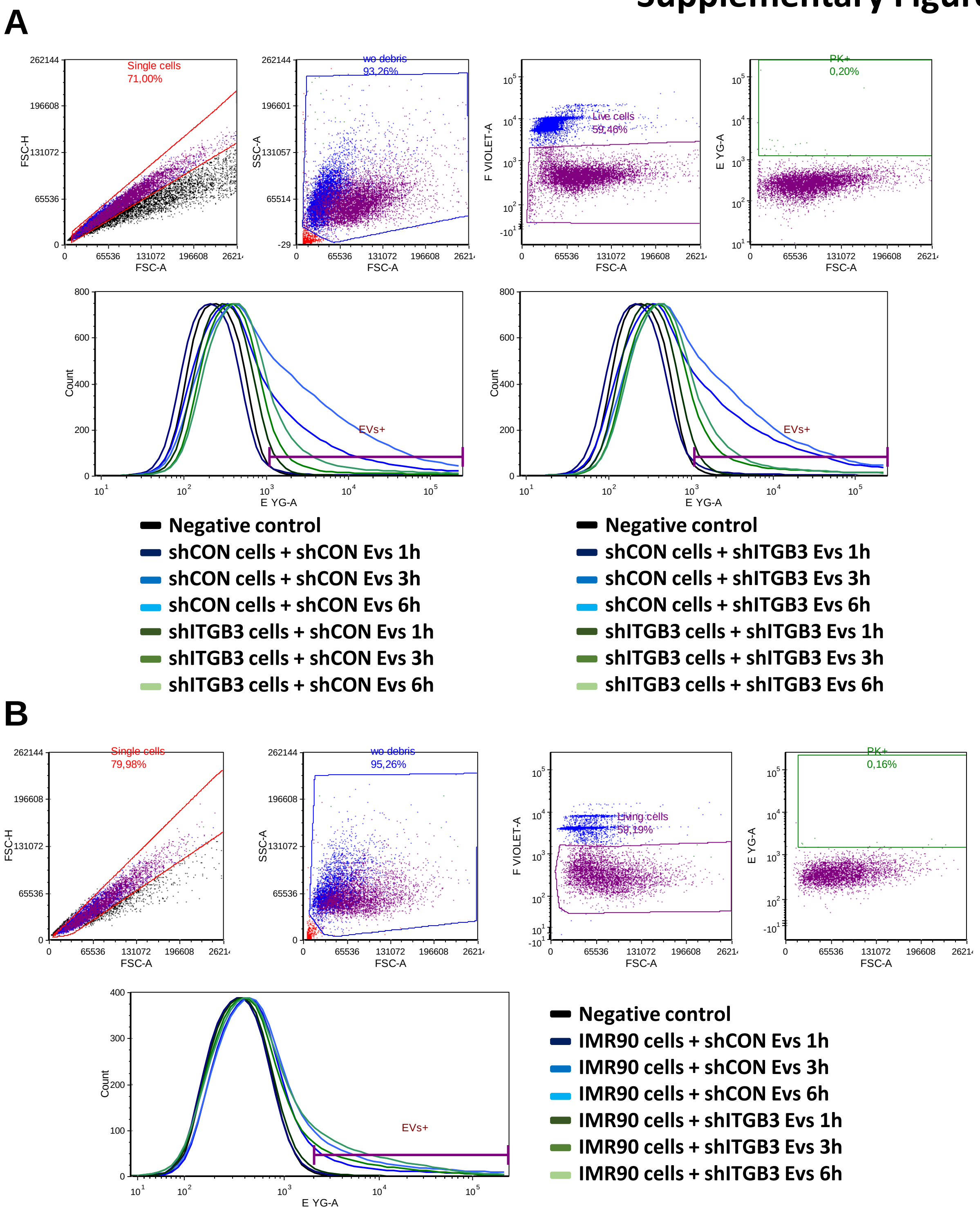

Supplementary Figure 15. FACS gating strategy and histogram intensity from which the bar charts for EVs internalization in A) Figure 1C, and B) Figure 1D were generated.

Supplementary figure 16

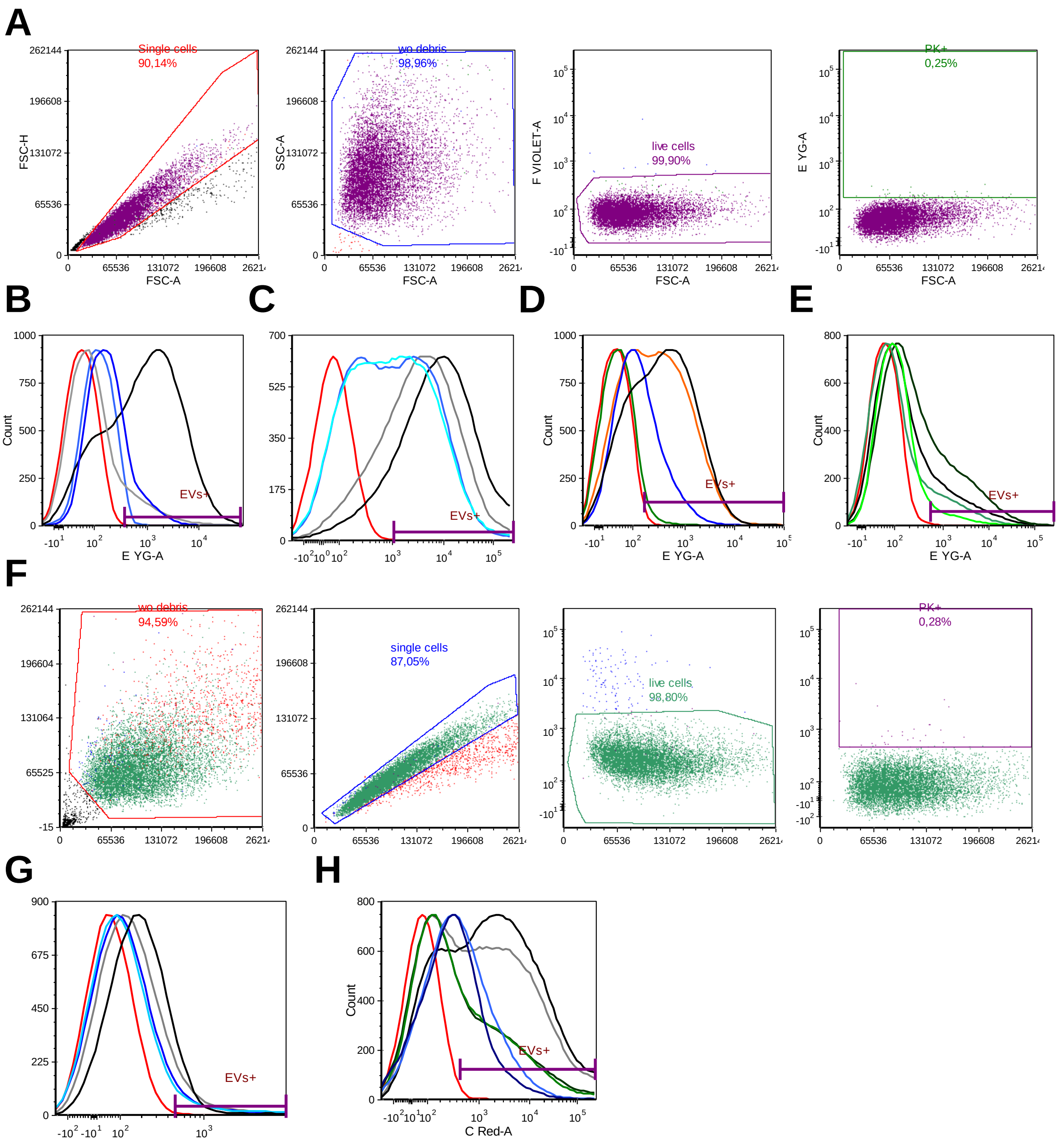

Supplementary Figure 16. FACS gating strategy and histogram intensity from which the bar charts for EVs internalization in A-E) Figure 2, and F-H) Figure 3A-B were generated.

Supplementary figure 17

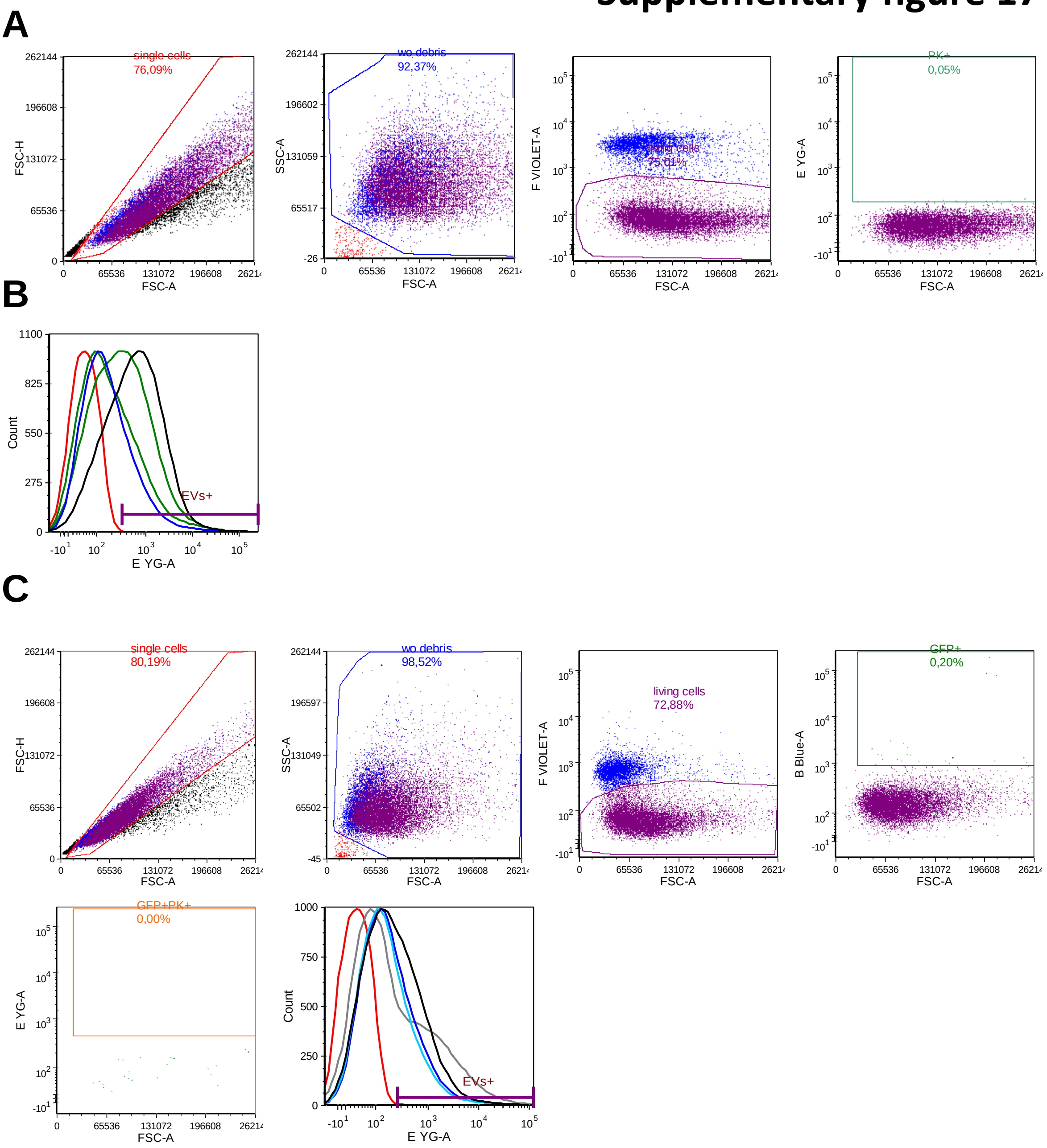

Supplementary Figure 17. FACS gating strategy and histogram intensity from which the bar charts for EVs internalization in A-B) Figure 6B, and C) Figure 6D were generated.

Supplementary figure 18

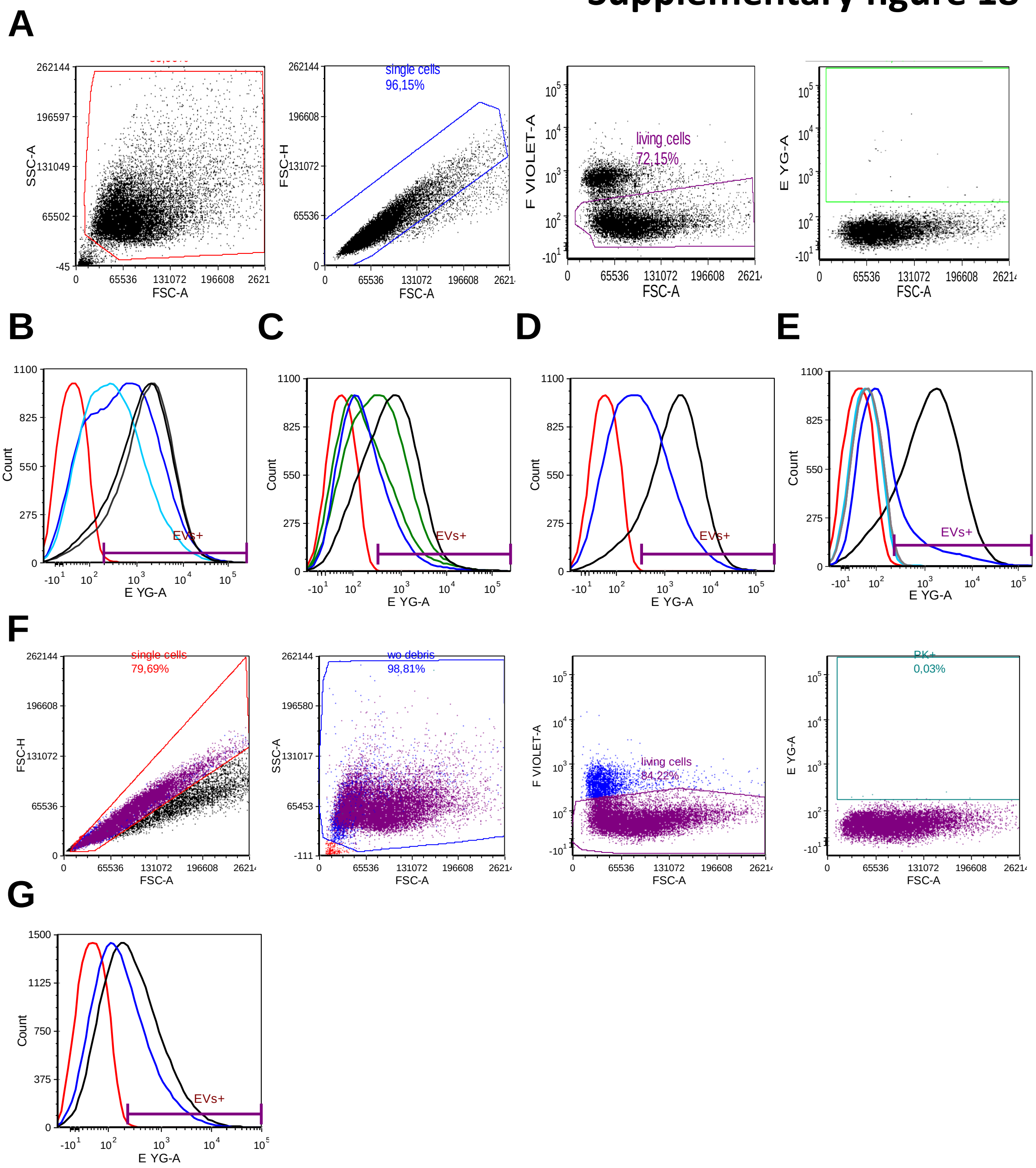

Supplementary Figure 18. FACS gating strategy and histogram intensity from which the bar charts for EVs internalization in A-D) Supplementary Figure 4, E) Supplementary Figure 5, and F-G) Supplementary Figure 7 were generated.
