# Supplementary material for: ITGB3-mediated uptake of small extracellular vesicles facilitates intercellular communication in breast cancer cells
Source: Nat Commun. 2020 Aug 26;11:4261. doi: 10.1038/s41467-020-18081-9 (PMC7450082; doi:10.1038/s41467-020-18081-9)
Supplement: Supplementary file 3 — Description of Additional Supplementary Files [file 41467_2020_18081_MOESM3_ESM.pdf]

Description of Additional Supplementary Files

File Name: Supplementary Data 1
Description: List of proteins identified by LC-MS/MS analysis on vesicle fractions obtained by ultracentrifugation of medium from shCON and shITGB3 cells.
