# Supplementary material for: ITGB3-mediated uptake of small extracellular vesicles facilitates intercellular communication in breast cancer cells
Source: Nat Commun. 2020 Aug 26;11:4261. doi: 10.1038/s41467-020-18081-9 (PMC7450082; doi:10.1038/s41467-020-18081-9)
Supplement: Supplementary file 4 — Supplementary Data 1 [file 41467_2020_18081_MOESM4_ESM.pdf]

							LOG TOP3 AREA sh-CTRL			LOG TOP3 AREA sh-ITGb3					NORMALIZED LOG TOP3 AREA sh-CTRL			NORMALIZED LOG TOP3 AREA sh-ITGb3							
**Accession**	**Description**	**ΣCoverage**	**Σ# Proteins**	**Σ# Unique Peptides**	**Σ# Peptides**	**Σ# PSMs**	**171026_SHSR_01**	**171026_SHSR_03**	**171026_SHSR_05**	**171026_SHSR_02**	**171026_SHSR_04**	**171026_SHSR_06**	**COUNT sh-CTRL**	**COUNT sh-ITGb3**	**171026_SHSR_01**	**171026_SHSR_03**	**171026_SHSR_05**	**171026_SHSR_02**	**171026_SHSR_04**	**171026_SHSR_06**	**AVG sh-CTRL**	**AVG sh-ITGb3**	**pvalue**	**qvalue**	**log2FC**
P62753	40S ribosomal protein S6 OS=Homo sapiens GN=RPS6 PE=1 SV=1 - [RS6_HUMAN]	31.33	1	9	9	54	25.79	25.50	25.45	27.83	27.60	27.53	3	3	25.95	25.57	25.51	27.58	27.82	27.27	25.68	27.56	0.001	0.003	1.878
P08865	40S ribosomal protein SA OS=Homo sapiens GN=RPSA PE=1 SV=4 - [RSSA_HUMAN]	57.29	1	13	13	78	26.14	26.35	26.49	28.11	28.26	28.46	3	3	26.31	26.42	26.55	27.86	28.48	28.20	26.42	28.18	0.001	0.003	1.756
P50990	T-complex protein 1 subunit theta OS=Homo sapiens GN=CCT8 PE=1 SV=4 - [TCPQ_HUMAN]	72.08	1	33	33	172	25.33	25.00	25.42	26.82	26.56	26.96	3	3	25.49	25.07	25.48	26.58	26.77	26.70	25.35	26.68	0.001	0.003	1.338
Q99729	Heterogeneous nuclear ribonucleoprotein A/B OS=Homo sapiens GN=HNRNPAB PE=1 SV=2 - [ROAA_HUMAN]	21.69	1	5	6	33	23.17	23.20	23.49	25.35	25.44	25.65	3	3	23.33	23.27	23.55	25.10	25.66	25.39	23.38	25.38	0.000	0.002	1.997
O43663	Protein regulator of cytokinesis 1 OS=Homo sapiens GN=PRC1 PE=1 SV=2 - [PRC1_HUMAN]	15.48	3	6	6	22	22.63	22.40	23.06	23.54	23.30	24.00	3	3	22.79	22.47	23.12	23.30	23.52	23.74	22.79	23.52	0.033	0.023	0.727
P17252	Protein kinase C alpha type OS=Homo sapiens GN=PRKCA PE=1 SV=4 - [KPCA_HUMAN]	21.58	4	10	11	27		22.32	22.37	24.15	24.09	24.14	2	3		22.39	22.43	23.91	24.31	23.88	22.41	24.03	0.003	0.005	1.622
P30040	Endoplasmic reticulum resident protein 29 OS=Homo sapiens GN=ERP29 PE=1 SV=4 - [ERP29_HUMAN]	56.32	1	12	12	74	23.97	25.08	25.02	26.39	27.39	27.42	3	3	24.13	25.15	25.08	26.14	27.61	27.16	24.78	26.97	0.016	0.014	2.187
Q53EZ4	Centrosomal protein of 55 kDa OS=Homo sapiens GN=CEP55 PE=1 SV=3 - [CEP55_HUMAN]	65.09	1	35	35	190	27.90	27.74	28.12	24.85	24.81	25.23	3	3	28.06	27.80	28.18	24.60	25.02	24.97	28.02	24.87	0.000	0.002	-3.150
P62318	Small nuclear ribonucleoprotein Sm D3 OS=Homo sapiens GN=SNRPD3 PE=1 SV=1 - [SMD3_HUMAN]	47.62	1	4	4	28	23.69	23.53	23.53	26.18	26.11	26.16	3	3	23.85	23.60	23.58	25.93	26.32	25.90	23.68	26.05	0.000	0.002	2.372
P14314	Glucosidase 2 subunit beta OS=Homo sapiens GN=PRKCSH PE=1 SV=2 - [GLU2B_HUMAN]	38.64	1	19	19	91	23.66	25.47	25.44	27.06	28.66	28.72	3	3	23.82	25.54	25.49	26.81	28.88	28.46	24.95	28.05	0.022	0.017	3.097
P05388	60S acidic ribosomal protein P0 OS=Homo sapiens GN=RPLP0 PE=1 SV=1 - [RLA0_HUMAN]	55.84	2	16	16	84	25.31	25.37	25.57	27.16	27.25	27.56	3	3	25.47	25.44	25.63	26.92	27.46	27.29	25.51	27.22	0.001	0.002	1.714
P62910	60S ribosomal protein L32 OS=Homo sapiens GN=RPL32 PE=1 SV=2 - [RL32_HUMAN]	52.59	1	9	9	42	24.49	24.39	24.41	27.05	27.14	27.00	3	3	24.65	24.45	24.47	26.80	27.35	26.73	24.52	26.96	0.000	0.002	2.441
Q71UM5	40S ribosomal protein S27-like OS=Homo sapiens GN=RPS27L PE=1 SV=3 - [RS27L_HUMAN]	41.67	1	1	5	21	23.61	24.06	24.28	25.30	25.78	26.11	3	3	23.77	24.13	24.34	25.06	26.00	25.85	24.08	25.63	0.010	0.010	1.556
Q9UNS2	COP9 signalosome complex subunit 3 OS=Homo sapiens GN=COPS3 PE=1 SV=3 - [CSN3_HUMAN]	13.48	1	4	4	15	20.84	20.08	20.90	23.10	22.15	23.10	3	3	21.00	20.14	20.96	22.86	22.37	22.84	20.70	22.69	0.003	0.006	1.985
Q9H4G4	Golgi-associated plant pathogenesis-related protein 1 OS=Homo sapiens GN=GLIPR2 PE=1 SV=3 - [GAPR1_HUMAN]	27.92	1	3	3	13	23.50	23.52	23.40	22.39		22.29	3	2	23.66	23.59	23.46	22.15		22.03	23.57	22.09	0.000	0.002	-1.480
P62266	40S ribosomal protein S23 OS=Homo sapiens GN=RPS23 PE=1 SV=3 - [RS23_HUMAN]	43.36	1	6	6	43	24.05	24.39	24.29	26.56	26.88	26.60	3	3	24.21	24.46	24.35	26.31	27.09	26.34	24.34	26.58	0.001	0.003	2.240
P42677	40S ribosomal protein S27 OS=Homo sapiens GN=RPS27 PE=1 SV=3 - [RS27_HUMAN]	41.67	1	1	5	30	23.40	23.84	24.02	25.66	25.94	26.33	3	3	23.56	23.91	24.07	25.41	26.16	26.07	23.85	25.88	0.002	0.004	2.033
P09651	Heterogeneous nuclear ribonucleoprotein A1 OS=Homo sapiens GN=HNRNPA1 PE=1 SV=5 - [ROA1_HUMAN]	41.13	2	12	13	95	25.75	25.82	25.87	27.42	27.64	27.71	3	3	25.91	25.89	25.93	27.18	27.86	27.44	25.91	27.49	0.001	0.004	1.584
P36578	60S ribosomal protein L4 OS=Homo sapiens GN=RPL4 PE=1 SV=5 - [RL4_HUMAN]	48.24	1	23	23	141	26.29	25.90	25.80	28.41	27.83	27.77	3	3	26.45	25.97	25.86	28.17	28.05	27.51	26.09	27.91	0.003	0.005	1.815
Q9UHD1	Cysteine and histidine-rich domain-containing protein 1 OS=Homo sapiens GN=CHORDC1 PE=1 SV=2 - [CHRD1_HUMAN]	55.42	1	13	13	44	22.65	22.62	23.01	24.74	24.50	25.05	3	3	22.81	22.69	23.07	24.50	24.72	24.78	22.86	24.67	0.000	0.002	1.810
P19338	Nucleolin OS=Homo sapiens GN=NCL PE=1 SV=3 - [NUCL_HUMAN]	29.58	1	17	17	86	23.55	24.23	23.47	27.12	27.42	26.85	3	3	23.72	24.30	23.53	26.88	27.63	26.59	23.85	27.04	0.001	0.003	3.187
P17844	Probable ATP-dependent RNA helicase DDX5 OS=Homo sapiens GN=DDX5 PE=1 SV=1 - [DDX5_HUMAN]	42.67	1	16	23	67	24.12	24.03	24.18	25.84	25.87	26.11	3	3	24.28	24.10	24.24	25.59	26.09	25.85	24.21	25.84	0.000	0.002	1.638
Q02790	Peptidyl-prolyl cis-trans isomerase FKBP4 OS=Homo sapiens GN=FKBP4 PE=1 SV=3 - [FKBP4_HUMAN]	55.12	1	21	21	92	22.89	22.64	22.85	25.26	25.07	25.48	3	3	23.06	22.71	22.91	25.01	25.29	25.22	22.89	25.18	0.000	0.002	2.282
Q9Y4L1	Hypoxia up-regulated protein 1 OS=Homo sapiens GN=HYOU1 PE=1 SV=1 - [HYOU1_HUMAN]	44.24	2	34	34	114	22.76	23.57	23.91	25.98	27.03	27.00	3	3	22.92	23.64	23.97	25.74	27.25	26.74	23.51	26.58	0.005	0.007	3.065
Q9Y3F4	Serine-threonine kinase receptor-associated protein OS=Homo sapiens GN=STRAP PE=1 SV=1 - [STRAP_HUMAN]	53.71	1	15	15	47	22.98	22.84	22.71	24.77	24.84	24.68	3	3	23.14	22.91	22.77	24.53	25.06	24.42	22.94	24.67	0.002	0.004	1.725
P62244	40S ribosomal protein S15a OS=Homo sapiens GN=RPS15A PE=1 SV=2 - [RS15A_HUMAN]	71.54	1	10	10	50	24.40	24.80	25.03	26.68	26.85	27.13	3	3	24.56	24.87	25.09	26.44	27.07	26.87	24.84	26.79	0.001	0.004	1.952
P84098	60S ribosomal protein L19 OS=Homo sapiens GN=RPL19 PE=1 SV=1 - [RL19_HUMAN]	26.53	1	6	6	23	24.25	23.74	24.47	26.55	26.09	26.57	3	3	24.41	23.81	24.53	26.30	26.30	26.31	24.25	26.30	0.001	0.003	2.054
Q9BX68	Histidine triad nucleotide-binding protein 2, mitochondrial OS=Homo sapiens GN=HINT2 PE=1 SV=1 - [HINT2_HUMAN]	27.61	1	3	3	9		22.80	22.65	23.87	23.54	23.39	2	3		22.87	22.71	23.63	23.75	23.13	22.79	23.50	0.067	0.037	0.713
O94808	Glutamine--fructose-6-phosphate aminotransferase [isomerizing] 2 OS=Homo sapiens GN=GFPT2 PE=1 SV=3 - [GFPT2_HUMAN]	25.95	1	8	13	31	22.88	22.71	23.67	24.32	24.12	25.25	3	3	23.04	22.78	23.73	24.07	24.34	24.99	23.18	24.47	0.031	0.022	1.284
P61978	Heterogeneous nuclear ribonucleoprotein K OS=Homo sapiens GN=HNRNPK PE=1 SV=1 - [HNRPK_HUMAN]	56.59	1	25	25	119	25.28	25.38	25.34	26.80	27.06	27.06	3	3	25.44	25.44	25.40	26.56	27.28	26.80	25.43	26.88	0.002	0.005	1.451
Q9UNE7	E3 ubiquitin-protein ligase CHIP OS=Homo sapiens GN=STUB1 PE=1 SV=2 - [CHIP_HUMAN]	54.79	1	12	13	50	23.81	23.72	23.43	24.82	24.67	24.52	3	3	23.97	23.79	23.49	24.57	24.89	24.26	23.75	24.57	0.023	0.018	0.823
O76021	Ribosomal L1 domain-containing protein 1 OS=Homo sapiens GN=RSL1D1 PE=1 SV=3 - [RL1D1_HUMAN]	22.86	1	10	10	34	22.39	22.01	22.42	24.53	24.32	24.46	3	3	22.55	22.08	22.48	24.28	24.54	24.20	22.37	24.34	0.000	0.002	1.970
P26373	60S ribosomal protein L13 OS=Homo sapiens GN=RPL13 PE=1 SV=4 - [RL13_HUMAN]	27.96	1	8	8	39	26.15	25.76	25.36	28.15	27.82	27.61	3	3	26.31	25.83	25.42	27.90	28.04	27.35	25.85	27.77	0.005	0.007	1.911
Q13148	TAR DNA-binding protein 43 OS=Homo sapiens GN=TARDBP PE=1 SV=1 - [TADBP_HUMAN]	13.04	1	3	3	16	22.31	22.89	22.84	23.50	23.97	24.06	3	3	22.47	22.96	22.90	23.26	24.19	23.80	22.78	23.75	0.035	0.024	0.971
Q8NBS9	Thioredoxin domain-containing protein 5 OS=Homo sapiens GN=TXNDC5 PE=1 SV=2 - [TXND5_HUMAN]	37.73	1	13	13	63	22.50	23.36	23.39	25.25	26.17	26.49	3	3	22.66	23.43	23.45	25.00	26.38	26.23	23.18	25.87	0.006	0.008	2.689
P62913	60S ribosomal protein L11 OS=Homo sapiens GN=RPL11 PE=1 SV=2 - [RL11_HUMAN]	50.00	1	10	10	48	24.81	25.20	25.42	27.08	27.27	27.40	3	3	24.97	25.27	25.48	26.83	27.49	27.14	25.24	27.15	0.001	0.004	1.912
P32969	60S ribosomal protein L9 OS=Homo sapiens GN=RPL9 PE=1 SV=1 - [RL9_HUMAN]	57.29	1	10	10	63	24.75	24.64	24.99	26.85	27.02	27.13	3	3	24.91	24.71	25.05	26.60	27.23	26.87	24.89	26.90	0.001	0.003	2.009
P25789	Proteasome subunit alpha type-4 OS=Homo sapiens GN=PSMA4 PE=1 SV=1 - [PSA4_HUMAN]	67.82	1	13	13	70	24.39	24.54	24.81	25.70	25.93	26.32	3	3	24.55	24.61	24.87	25.46	26.15	26.06	24.68	25.89	0.007	0.009	1.207
P62854	40S ribosomal protein S26 OS=Homo sapiens GN=RPS26 PE=1 SV=3 - [RS26_HUMAN]	31.30	2	3	3	24	25.20	25.09	25.07	27.39	27.14	26.97	3	3	25.36	25.16	25.13	27.15	27.35	26.71	25.22	27.07	0.001	0.003	1.854
P24534	Elongation factor 1-beta OS=Homo sapiens GN=EEF1B2 PE=1 SV=3 - [EF1B_HUMAN]	71.56	1	8	10	56	25.91	26.25	26.60	27.93	28.06	28.37	3	3	26.07	26.32	26.66	27.69	28.28	28.11	26.35	28.02	0.002	0.005	1.672
P46782	40S ribosomal protein S5 OS=Homo sapiens GN=RPS5 PE=1 SV=4 - [RS5_HUMAN]	50.00	1	9	9	59	24.75	24.49	24.96	26.79	26.74	26.91	3	3	24.91	24.55	25.02	26.55	26.96	26.65	24.83	26.72	0.001	0.002	1.889
P62701	40S ribosomal protein S4, X isoform OS=Homo sapiens GN=RPS4X PE=1 SV=2 - [RS4X_HUMAN]	65.78	3	21	21	156	25.43	25.67	25.79	27.52	27.61	27.59	3	3	25.59	25.74	25.85	27.27	27.83	27.33	25.73	27.47	0.001	0.003	1.748
Q9H3K6	BolA-like protein 2 OS=Homo sapiens GN=BOLA2 PE=1 SV=1 - [BOLA2_HUMAN]	59.30	1	4	4	14	22.98	22.53	22.72	24.79	24.50	24.83	3	3	23.14	22.60	22.78	24.54	24.71	24.57	22.84	24.61	0.000	0.002	1.771
P13010	X-ray repair cross-complementing protein 5 OS=Homo sapiens GN=XRCC5 PE=1 SV=3 - [XRCC5_HUMAN]	54.51	1	31	32	162	24.05	23.74	23.55	26.55	25.92	26.05	3	3	24.21	23.81	23.61	26.31	26.14	25.79	23.88	26.08	0.001	0.003	2.200
Q92599	Septin-8 OS=Homo sapiens GN=SEPT8 PE=1 SV=4 - [SEPT8_HUMAN]	12.22	2	1	6	21	22.67	22.62	23.00	24.07	23.84	24.24	3	3	22.83	22.69	23.06	23.82	24.06	23.98	22.86	23.95	0.001	0.003	1.092
P62241	40S ribosomal protein S8 OS=Homo sapiens GN=RPS8 PE=1 SV=2 - [RS8_HUMAN]	60.10	1	15	15	91	26.69	26.37	26.43	28.87	28.38	28.29	3	3	26.85	26.44	26.49	28.63	28.60	28.03	26.59	28.42	0.001	0.004	1.827
Q14103	Heterogeneous nuclear ribonucleoprotein D0 OS=Homo sapiens GN=HNRNPD PE=1 SV=1 - [HNRPD_HUMAN]	32.96	1	8	10	66	24.46	24.09	24.29	26.74	26.74	26.87	3	3	24.63	24.16	24.35	26.49	26.96	26.61	24.38	26.69	0.000	0.002	2.310
Q01130	Serine/arginine-rich splicing factor 2 OS=Homo sapiens GN=SRSF2 PE=1 SV=4 - [SRSF2_HUMAN]	22.62	2	5	5	27	23.70	23.75	24.09	26.06	25.97	26.10	3	3	23.87	23.82	24.15	25.81	26.19	25.84	23.95	25.95	0.000	0.002	2.001
P31943	Heterogeneous nuclear ribonucleoprotein H OS=Homo sapiens GN=HNRNPH1 PE=1 SV=4 - [HNRH1_HUMAN]	43.43	1	6	15	86	24.69	24.53	24.69	26.18	26.14	26.45	3	3	24.85	24.60	24.75	25.94	26.36	26.19	24.73	26.16	0.001	0.002	1.427
P22102	Trifunctional purine biosynthetic protein adenosine-3 OS=Homo sapiens GN=GART PE=1 SV=1 - [PUR2_HUMAN]	40.79	1	29	29	132	24.31	24.72	24.73	26.56	26.64	26.93	3	3	24.47	24.79	24.79	26.31	26.86	26.66	24.68	26.61	0.001	0.002	1.929
Q9ULC4	Malignant T-cell-amplified sequence 1 OS=Homo sapiens GN=MCTS1 PE=1 SV=1 - [MCTS1_HUMAN]	57.46	1	7	7	33	23.17	23.37	23.72	24.44	24.58	24.80	3	3	23.33	23.44	23.78	24.19	24.80	24.53	23.52	24.51	0.011	0.011	0.988
P49411	Elongation factor Tu, mitochondrial OS=Homo sapiens GN=TUFM PE=1 SV=2 - [EFTU_HUMAN]	63.05	1	23	23	87	23.64	23.74	24.30	25.80	25.92	26.16	3	3	23.80	23.81	24.36	25.56	26.13	25.90	23.99	25.86	0.002	0.004	1.872
Q86UP2	Kinectin OS=Homo sapiens GN=KTN1 PE=1 SV=1 - [KTN1_HUMAN]	10.54	1	12	12	24	21.09	21.35	21.37	23.34	23.30	23.30	3	3	21.25	21.42	21.43	23.09	23.52	23.04	21.37	23.22	0.000	0.002	1.848
Q08211	ATP-dependent RNA helicase A OS=Homo sapiens GN=DHX9 PE=1 SV=4 - [DHX9_HUMAN]	39.84	1	40	40	145	23.42	23.38	23.90	25.68	25.47	25.80	3	3	23.59	23.45	23.96	25.44	25.69	25.53	23.66	25.55	0.000	0.002	1.888
O75937	DnaJ homolog subfamily C member 8 OS=Homo sapiens GN=DNAJC8 PE=1 SV=2 - [DNJC8_HUMAN]	23.32	1	4	4	12	21.12	21.71	22.21	22.75	23.08	23.68	3	3	21.28	21.78	22.27	22.51	23.29	23.42	21.78	23.07	0.032	0.022	1.297
P27635	60S ribosomal protein L10 OS=Homo sapiens GN=RPL10 PE=1 SV=4 - [RL10_HUMAN]	52.34	2	13	13	77	26.42	25.93	26.23	27.96	27.78	27.96	3	3	26.58	26.00	26.29	27.72	27.99	27.70	26.29	27.80	0.001	0.004	1.513
P05386	60S acidic ribosomal protein P1 OS=Homo sapiens GN=RPLP1 PE=1 SV=1 - [RLA1_HUMAN]	89.47	1	3	5	59	26.45	27.11	27.24	29.01	29.49	29.38	3	3	26.61	27.18	27.30	28.77	29.70	29.12	27.03	29.20	0.003	0.006	2.166
P40429	60S ribosomal protein L13a OS=Homo sapiens GN=RPL13A PE=1 SV=2 - [RL13A_HUMAN]	49.26	2	13	13	51	24.77	24.60	24.81	27.31	26.79	26.99	3	3	24.93	24.67	24.87	27.07	27.01	26.73	24.82	26.93	0.000	0.002	2.111
P31689	DnaJ homolog subfamily A member 1 OS=Homo sapiens GN=DNAJA1 PE=1 SV=2 - [DNJA1_HUMAN]	68.51	4	20	21	172	28.92	28.88	29.26	27.81	27.95	28.24	3	3	29.08	28.95	29.32	27.56	28.17	27.97	29.12	27.90	0.004	0.007	-1.218
Q96EY1	DnaJ homolog subfamily A member 3, mitochondrial OS=Homo sapiens GN=DNAJA3 PE=1 SV=2 - [DNJA3_HUMAN]	4.79	1	1	1	4		20.38	20.27		21.32	21.20	2	2		20.45	20.33		21.53	20.94	20.39	21.24	0.108	0.053	0.849
O75506	Heat shock factor-binding protein 1 OS=Homo sapiens GN=HSBP1 PE=1 SV=1 - [HSBP1_HUMAN]	64.47	1	3	3	13	23.05	23.25	23.77	24.02	24.30	24.95	3	3	23.21	23.32	23.83	23.78	24.52	24.69	23.45	24.33	0.061	0.034	0.874
P46776	60S ribosomal protein L27a OS=Homo sapiens GN=RPL27A PE=1 SV=2 - [RL27A_HUMAN]	28.38	1	5	5	26	25.46	25.23	25.49	27.91	27.67	27.55	3	3	25.62	25.30	25.55	27.67	27.88	27.29	25.49	27.61	0.000	0.002	2.122
P14866	Heterogeneous nuclear ribonucleoprotein L OS=Homo sapiens GN=HNRNPL PE=1 SV=2 - [HNRPL_HUMAN]	50.93	1	14	15	83	23.84	23.54	23.61	26.10	26.25	25.96	3	3	24.00	23.61	23.67	25.86	26.46	25.70	23.76	26.01	0.001	0.003	2.243
P67809	Nuclease-sensitive element-binding protein 1 OS=Homo sapiens GN=YBX1 PE=1 SV=3 - [YBOX1_HUMAN]	47.53	2	10	13	98	24.67	25.15	25.10	27.74	27.67	27.91	3	3	24.83	25.22	25.16	27.49	27.89	27.65	25.07	27.68	0.000	0.002	2.607
P84103	Serine/arginine-rich splicing factor 3 OS=Homo sapiens GN=SRSF3 PE=1 SV=1 - [SRSF3_HUMAN]	23.17	1	3	3	22	24.98	25.11	25.35	26.69	26.75	26.76	3	3	25.14	25.18	25.41	26.44	26.96	26.50	25.24	26.63	0.002	0.004	1.391
P29692	Elongation factor 1-delta OS=Homo sapiens GN=EEF1D PE=1 SV=5 - [EF1D_HUMAN]	71.89	1	16	18	117	25.59	25.79	26.04	27.58	27.46	28.01	3	3	25.75	25.86	26.10	27.34	27.67	27.74	25.90	27.59	0.000	0.002	1.681
O76094	Signal recognition particle subunit SRP72 OS=Homo sapiens GN=SRP72 PE=1 SV=3 - [SRP72_HUMAN]	30.10	1	14	14	43	22.35	22.36	22.51	24.31	24.10	24.64	3	3	22.51	22.42	22.57	24.06	24.32	24.38	22.50	24.25	0.000	0.002	1.754
Q9Y617	Phosphoserine aminotransferase OS=Homo sapiens GN=PSAT1 PE=1 SV=2 - [SERC_HUMAN]	44.59	1	11	11	19	21.91		22.54	23.52	23.31	24.13	2	3	22.07		22.59	23.27	23.53	23.87	22.33	23.56	0.025	0.019	1.223
Q15019	Septin-2 OS=Homo sapiens GN=SEPT2 PE=1 SV=1 - [SEPT2_HUMAN]	32.96	1	10	10	44	24.46	24.44	24.64	25.46	25.54	25.86	3	3	24.62	24.51	24.70	25.21	25.76	25.60	24.61	25.52	0.006	0.008	0.914
O43681	ATPase ASNA1 OS=Homo sapiens GN=ASNA1 PE=1 SV=2 - [ASNA_HUMAN]	29.89	1	6	6	23	23.33	23.17	23.48	24.49	24.11	24.55	3	3	23.50	23.23	23.54	24.25	24.33	24.29	23.42	24.29	0.001	0.003	0.862
P20290	Transcription factor BTF3 OS=Homo sapiens GN=BTF3 PE=1 SV=1 - [BTF3_HUMAN]	37.86	1	7	7	30	23.90	24.20	24.21	25.64	25.62	25.85	3	3	24.06	24.27	24.27	25.39	25.83	25.59	24.20	25.61	0.001	0.003	1.407
P23919	Thymidylate kinase OS=Homo sapiens GN=DTYMK PE=1 SV=4 - [KTHY_HUMAN]	52.36	1	10	10	28	22.49	22.98	23.01	24.45	24.57	24.87	3	3	22.65	23.05	23.07	24.21	24.79	24.61	22.92	24.53	0.002	0.004	1.608
O14979	Heterogeneous nuclear ribonucleoprotein D-like OS=Homo sapiens GN=HNRNPDL PE=1 SV=3 - [HNRDL_HUMAN]	21.19	1	6	8	35	23.63	23.33	23.95	26.14	26.03	26.14	3	3	23.79	23.40	24.01	25.90	26.25	25.88	23.73	26.01	0.000	0.002	2.275
Q9UBC2	Epidermal growth factor receptor substrate 15-like 1 OS=Homo sapiens GN=EPS15L1 PE=1 SV=1 - [EP15R_HUMAN]	79.75	1	49	49	206	27.06	27.43	27.77	24.58	24.47	25.29	3	3	27.23	27.50	27.83	24.34	24.68	25.03	27.52	24.68	0.000	0.002	-2.836
P62269	40S ribosomal protein S18 OS=Homo sapiens GN=RPS18 PE=1 SV=3 - [RS18_HUMAN]	55.92	1	10	10	49	25.35	24.70	24.87	27.20	26.98	27.06	3	3	25.52	24.77	24.93	26.96	27.19	26.80	25.07	26.98	0.002	0.004	1.913
P30050	60S ribosomal protein L12 OS=Homo sapiens GN=RPL12 PE=1 SV=1 - [RL12_HUMAN]	58.18	2	8	8	47	24.24	24.66	24.77	27.17	27.08	27.23	3	3	24.41	24.73	24.83	26.92	27.29	26.97	24.65	27.06	0.000	0.002	2.410
P15880	40S ribosomal protein S2 OS=Homo sapiens GN=RPS2 PE=1 SV=2 - [RS2_HUMAN]	44.71	1	14	14	84	26.12	25.80	25.86	28.30	27.95	27.64	3	3	26.29	25.87	25.92	28.06	28.17	27.38	26.02	27.87	0.003	0.005	1.843
O60506	Heterogeneous nuclear ribonucleoprotein Q OS=Homo sapiens GN=SYNCRIP PE=1 SV=2 - [HNRPQ_HUMAN]	35.96	1	12	18	70	23.92	23.64	23.65	25.55	25.64	25.60	3	3	24.08	23.71	23.71	25.30	25.86	25.34	23.83	25.50	0.002	0.004	1.667
P07910	Heterogeneous nuclear ribonucleoproteins C1/C2 OS=Homo sapiens GN=HNRNPC PE=1 SV=4 - [HNRPC_HUMAN]	41.18	4	15	15	62	24.60	24.66	24.92	26.85	27.29	27.06	3	3	24.76	24.73	24.98	26.60	27.51	26.80	24.83	26.97	0.002	0.004	2.146
O00231	26S proteasome non-ATPase regulatory subunit 11 OS=Homo sapiens GN=PSMD11 PE=1 SV=3 - [PSD11_HUMAN]	65.64	1	22	22	72	23.82	23.66	23.76	25.37	25.11	25.55	3	3	23.98	23.73	23.82	25.13	25.32	25.29	23.85	25.25	0.000	0.002	1.402
P62277	40S ribosomal protein S13 OS=Homo sapiens GN=RPS13 PE=1 SV=2 - [RS13_HUMAN]	35.76	1	7	7	25	25.34	24.93	25.15	27.24	27.25	27.11	3	3	25.50	24.99	25.21	26.99	27.46	26.85	25.24	27.10	0.001	0.004	1.865
Q9HB71	Calcyclin-binding protein OS=Homo sapiens GN=CACYBP PE=1 SV=2 - [CYBP_HUMAN]	47.81	1	9	11	48	24.32	23.82	24.64	26.06	25.73	26.17	3	3	24.48	23.89	24.70	25.82	25.95	25.91	24.36	25.89	0.003	0.006	1.534
Q99996	A-kinase anchor protein 9 OS=Homo sapiens GN=AKAP9 PE=1 SV=3 - [AKAP9_HUMAN]	0.36	2	1	2	7	26.19	25.43	26.17	25.41	24.80	25.51	3	3	26.35	25.50	26.23	25.17	25.02	25.25	26.03	25.15	0.033	0.022	-0.880
Q9P2J5	Leucine--tRNA ligase, cytoplasmic OS=Homo sapiens GN=LARS PE=1 SV=2 - [SYLC_HUMAN]	41.67	1	36	36	122	23.25	22.85	23.12	25.02	25.00	25.32	3	3	23.41	22.92	23.18	24.77	25.21	25.06	23.17	25.01	0.001	0.003	1.845
P63220	40S ribosomal protein S21 OS=Homo sapiens GN=RPS21 PE=1 SV=1 - [RS21_HUMAN]	60.24	1	6	6	23	22.69	22.67	22.82	24.91	25.20	25.61	3	3	22.85	22.74	22.88	24.66	25.41	25.35	22.82	25.14	0.001	0.003	2.317
Q7L2H7	Eukaryotic translation initiation factor 3 subunit M OS=Homo sapiens GN=EIF3M PE=1 SV=1 - [EIF3M_HUMAN]	39.30	1	10	10	40	23.64	23.44	24.03	24.75	24.67	25.42	3	3	23.80	23.51	24.08	24.51	24.89	25.16	23.80	24.85	0.014	0.013	1.055
P62316	Small nuclear ribonucleoprotein Sm D2 OS=Homo sapiens GN=SNRPD2 PE=1 SV=1 - [SMD2_HUMAN]	74.58	1	10	10	38	22.96	22.10	22.89	25.64	25.47	25.94	3	3	23.12	22.17	22.95	25.39	25.69	25.68	22.75	25.59	0.001	0.003	2.839
P55795	Heterogeneous nuclear ribonucleoprotein H2 OS=Homo sapiens GN=HNRNPH2 PE=1 SV=1 - [HNRH2_HUMAN]	36.08	1	5	13	68	24.55	24.49	24.45	26.12	26.11	26.38	3	3	24.71	24.56	24.51	25.87	26.33	26.12	24.59	26.10	0.000	0.002	1.512
P12956	X-ray repair cross-complementing protein 6 OS=Homo sapiens GN=XRCC6 PE=1 SV=2 - [XRCC6_HUMAN]	54.84	1	29	29	144	24.58	24.32	24.25	27.36	26.76	26.47	3	3	24.74	24.39	24.31	27.12	26.97	26.21	24.48	26.77	0.002	0.004	2.283
O95433	Activator of 90 kDa heat shock protein ATPase homolog 1 OS=Homo sapiens GN=AHSA1 PE=1 SV=1 - [AHSA1_HUMAN]	44.08	1	14	14	41	24.25	23.27		26.07	25.06	25.71	2	3	24.42	23.34		25.83	25.28	25.45	23.88	25.52	0.036	0.024	1.642
P62304	Small nuclear ribonucleoprotein E OS=Homo sapiens GN=SNRPE PE=1 SV=1 - [RUXE_HUMAN]	52.17	1	3	3	18	23.17	23.17	23.98	24.86	25.12	25.53	3	3	23.33	23.24	24.04	24.62	25.34	25.27	23.54	25.08	0.011	0.011	1.540
P26640	Valine--tRNA ligase OS=Homo sapiens GN=VARS PE=1 SV=4 - [SYVC_HUMAN]	32.28	1	28	28	97	23.39	22.77	23.03	25.73	24.64	25.28	3	3	23.55	22.84	23.09	25.48	24.86	25.02	23.16	25.12	0.002	0.005	1.960
P25786	Proteasome subunit alpha type-1 OS=Homo sapiens GN=PSMA1 PE=1 SV=1 - [PSA1_HUMAN]	74.90	1	16	16	83	24.15	24.81	24.82	25.96	26.24	26.49	3	3	24.31	24.88	24.88	25.71	26.46	26.22	24.69	26.13	0.008	0.009	1.443
P62851	40S ribosomal protein S25 OS=Homo sapiens GN=RPS25 PE=1 SV=1 - [RS25_HUMAN]	36.80	1	5	5	22	24.72	24.48	24.93	27.30	27.07	27.02	3	3	24.88	24.55	24.99	27.05	27.28	26.76	24.80	27.03	0.000	0.002	2.228
P62081	40S ribosomal protein S7 OS=Homo sapiens GN=RPS7 PE=1 SV=1 - [RS7_HUMAN]	36.60	1	7	7	46	25.99	25.70	25.97	28.14	28.31	28.09	3	3	26.15	25.77	26.03	27.90	28.52	27.82	25.98	28.08	0.001	0.003	2.097
P61353	60S ribosomal protein L27 OS=Homo sapiens GN=RPL27 PE=1 SV=2 - [RL27_HUMAN]	44.85	1	6	6	35	24.43	24.34	24.63	27.52	27.18	27.05	3	3	24.59	24.41	24.69	27.27	27.39	26.79	24.56	27.15	0.000	0.002	2.591
P68402	Platelet-activating factor acetylhydrolase IB subunit beta OS=Homo sapiens GN=PAFAH1B2 PE=1 SV=1 - [PA1B2_HUMAN]	56.77	1	7	7	35	24.28	24.18	24.41	25.89	26.20	26.41	3	3	24.44	24.25	24.47	25.65	26.42	26.15	24.38	26.07	0.002	0.004	1.690
P49368	T-complex protein 1 subunit gamma OS=Homo sapiens GN=CCT3 PE=1 SV=4 - [TCPG_HUMAN]	68.07	1	31	31	205	25.85	25.53	25.72	27.04	26.89	27.23	3	3	26.01	25.60	25.78	26.79	27.11	26.97	25.80	26.96	0.002	0.004	1.162
Q9Y230	RuvB-like 2 OS=Homo sapiens GN=RUVBL2 PE=1 SV=3 - [RUVB2_HUMAN]	62.85	1	24	24	97	23.96	24.56	24.45	26.20	26.42	26.24	3	3	24.12	24.63	24.51	25.95	26.63	25.98	24.42	26.19	0.003	0.005	1.767
O75190	DnaJ homolog subfamily B member 6 OS=Homo sapiens GN=DNAJB6 PE=1 SV=2 - [DNJB6_HUMAN]	45.71	3	13	14	62	27.43	27.24	27.37	23.87	24.09	24.60	3	3	27.59	27.31	27.43	23.63	24.31	24.34	27.44	24.09	0.000	0.002	-3.354
P35268	60S ribosomal protein L22 OS=Homo sapiens GN=RPL22 PE=1 SV=2 - [RL22_HUMAN]	57.03	1	4	5	36	24.78	25.30	25.55	26.49	27.50	27.54	3	3	24.95	25.36	25.61	26.25	27.72	27.28	25.31	27.08	0.021	0.017	1.773
P34897	Serine hydroxymethyltransferase, mitochondrial OS=Homo sapiens GN=SHMT2 PE=1 SV=3 - [GLYM_HUMAN]	37.10	1	13	13	51	22.66	22.96	22.90	24.00	24.32	24.57	3	3	22.82	23.03	22.96	23.75	24.54	24.31	22.93	24.20	0.006	0.008	1.264
P06748	Nucleophosmin OS=Homo sapiens GN=NPM1 PE=1 SV=2 - [NPM_HUMAN]	56.12	2	13	13	131	26.47	26.53	26.82	28.19	28.71	28.64	3	3	26.63	26.60	26.88	27.94	28.93	28.38	26.70	28.42	0.005	0.007	1.713
P61254	60S ribosomal protein L26 OS=Homo sapiens GN=RPL26 PE=1 SV=1 - [RL26_HUMAN]	43.45	3	11	11	46	23.53	24.03	24.05	26.29	26.20	26.36	3	3	23.69	24.10	24.11	26.05	26.41	26.10	23.97	26.19	0.000	0.002	2.221
Q00839	Heterogeneous nuclear ribonucleoprotein U OS=Homo sapiens GN=HNRNPU PE=1 SV=6 - [HNRPU_HUMAN]	40.61	1	28	29	146	25.02	25.31	25.61	27.26	27.67	27.45	3	3	25.18	25.38	25.67	27.01	27.89	27.18	25.41	27.36	0.003	0.005	1.952
P62829	60S ribosomal protein L23 OS=Homo sapiens GN=RPL23 PE=1 SV=1 - [RL23_HUMAN]	41.43	1	6	6	31	25.34	25.22	25.53	26.99	27.29	27.22	3	3	25.50	25.29	25.59	26.74	27.51	26.96	25.46	27.07	0.003	0.005	1.612
P08708	40S ribosomal protein S17 OS=Homo sapiens GN=RPS17 PE=1 SV=2 - [RS17_HUMAN]	57.04	1	7	7	54	24.25	23.79	23.93	26.41	26.60	26.50	3	3	24.41	23.86	23.99	26.17	26.82	26.24	24.09	26.41	0.001	0.003	2.319
P46779	60S ribosomal protein L28 OS=Homo sapiens GN=RPL28 PE=1 SV=3 - [RL28_HUMAN]	44.53	1	8	8	35	24.37	24.99	24.70	26.96	26.99	27.08	3	3	24.53	25.06	24.76	26.72	27.20	26.82	24.78	26.91	0.001	0.002	2.131
P51665	26S proteasome non-ATPase regulatory subunit 7 OS=Homo sapiens GN=PSMD7 PE=1 SV=2 - [PSMD7_HUMAN]	50.00	1	12	12	57	23.91	23.72	24.19	26.05	26.04	25.97	3	3	24.07	23.79	24.25	25.81	26.26	25.71	24.04	25.92	0.001	0.003	1.887
Q15084	Protein disulfide-isomerase A6 OS=Homo sapiens GN=PDIA6 PE=1 SV=1 - [PDIA6_HUMAN]	52.50	1	15	15	78	24.82	25.06	25.26	26.35	27.02	27.15	3	3	24.99	25.13	25.32	26.10	27.23	26.89	25.14	26.74	0.010	0.011	1.598
P30520	Adenylosuccinate synthetase isozyme 2 OS=Homo sapiens GN=ADSS PE=1 SV=3 - [PURA2_HUMAN]	24.34	1	9	9	28	22.60	22.04	22.68	23.90	23.13	24.10	3	3	22.76	22.11	22.74	23.66	23.35	23.84	22.54	23.61	0.014	0.013	1.079
Q04637	Eukaryotic translation initiation factor 4 gamma 1 OS=Homo sapiens GN=EIF4G1 PE=1 SV=4 - [IF4G1_HUMAN]	15.70	2	22	22	65	22.72	22.25	22.53	24.11	23.90	24.35	3	3	22.88	22.32	22.59	23.87	24.12	24.09	22.60	24.03	0.001	0.004	1.427
Q99733	Nucleosome assembly protein 1-like 4 OS=Homo sapiens GN=NAP1L4 PE=1 SV=1 - [NP1L4_HUMAN]	53.87	1	11	13	69	24.87	24.98	25.01	26.72	26.44	26.87	3	3	25.03	25.05	25.07	26.47	26.66	26.61	25.05	26.58	0.000	0.001	1.532
Q13838	Spliceosome RNA helicase DDX39B OS=Homo sapiens GN=DDX39B PE=1 SV=1 - [DX39B_HUMAN]	44.63	1	5	16	69	24.06	24.78	24.95	25.83	26.14	26.54	3	3	24.22	24.85	25.01	25.59	26.36	26.28	24.69	26.07	0.016	0.014	1.380
Q9Y265	RuvB-like 1 OS=Homo sapiens GN=RUVBL1 PE=1 SV=1 - [RUVB1_HUMAN]	47.15	1	17	17	97	24.44	24.92	25.08	26.52	26.75	26.66	3	3	24.60	24.99	25.14	26.27	26.97	26.40	24.91	26.55	0.004	0.006	1.637
P62917	60S ribosomal protein L8 OS=Homo sapiens GN=RPL8 PE=1 SV=2 - [RL8_HUMAN]	46.30	1	13	13	71	24.44	24.30	24.56	27.30	26.49	27.18	3	3	24.60	24.37	24.62	27.06	26.71	26.92	24.53	26.90	0.000	0.002	2.364
P49207	60S ribosomal protein L34 OS=Homo sapiens GN=RPL34 PE=1 SV=3 - [RL34_HUMAN]	36.75	1	5	5	27	23.80	24.02	23.55	26.99	26.52	26.76	3	3	23.96	24.09	23.61	26.74	26.73	26.50	23.89	26.66	0.000	0.002	2.772
Q9UNM6	26S proteasome non-ATPase regulatory subunit 13 OS=Homo sapiens GN=PSMD13 PE=1 SV=2 - [PSD13_HUMAN]	59.57	1	17	17	77	23.83	23.41	23.84	25.06	24.67	25.41	3	3	23.99	23.48	23.90	24.81	24.89	25.15	23.79	24.95	0.003	0.006	1.161
Q02543	60S ribosomal protein L18a OS=Homo sapiens GN=RPL18A PE=1 SV=2 - [RL18A_HUMAN]	48.86	1	11	11	65	24.02	24.11	24.43	26.42	25.94	26.52	3	3	24.18	24.18	24.49	26.18	26.16	26.26	24.29	26.20	0.000	0.002	1.912
P18124	60S ribosomal protein L7 OS=Homo sapiens GN=RPL7 PE=1 SV=1 - [RL7_HUMAN]	49.60	1	14	14	95	26.69	25.79	26.36	28.63	27.97	28.01	3	3	26.85	25.86	26.42	28.38	28.19	27.75	26.38	28.11	0.007	0.009	1.731
Q9NZZ3	Charged multivesicular body protein 5 OS=Homo sapiens GN=CHMP5 PE=1 SV=1 - [CHMP5_HUMAN]	42.47	1	8	8	61	28.72	28.68	28.82	26.32	25.69	26.45	3	3	28.88	28.75	28.88	26.08	25.91	26.19	28.84	26.06	0.000	0.001	-2.778
P07237	Protein disulfide-isomerase OS=Homo sapiens GN=P4HB PE=1 SV=3 - [PDIA1_HUMAN]	55.31	1	25	25	129	24.30	25.26	25.03	25.98	27.05	27.21	3	3	24.47	25.32	25.09	25.74	27.26	26.95	24.96	26.65	0.034	0.023	1.688
Q16658	Fascin OS=Homo sapiens GN=FSCN1 PE=1 SV=3 - [FSCN1_HUMAN]	36.92	1	14	14	32	21.96	21.86		24.77	24.62	24.91	2	3	22.12	21.93		24.53	24.83	24.65	22.02	24.67	0.000	0.002	2.645
Q02878	60S ribosomal protein L6 OS=Homo sapiens GN=RPL6 PE=1 SV=3 - [RL6_HUMAN]	43.06	1	16	16	91	25.06	24.31	25.05	27.63	27.15	27.20	3	3	25.22	24.38	25.11	27.39	27.37	26.94	24.90	27.23	0.002	0.004	2.326

P62750	60S ribosomal protein L23a OS=Homo sapiens GN=RPL23A PE=1 SV=1 - [RL23A_HUMAN]	42.31	1	7	7	26	24.13	23.88	24.03	26.17	26.30	26.73	3	3	24.29	23.95	24.09	25.92	26.52	26.47	24.11	26.30	0.001	0.002	2.194
P21796	Voltage-dependent anion-selective channel protein 1 OS=Homo sapiens GN=VDAC1 PE=1 SV=2 - [VDAC1_HUMAN]	63.25	1	14	15	103	23.90	25.85	25.87	25.43	27.04	27.11	3	3	24.06	25.92	25.92	25.18	27.25	26.85	25.30	26.43	0.273	0.104	1.127
Q9NQR4	Omega-amidase NIT2 OS=Homo sapiens GN=NIT2 PE=1 SV=1 - [NIT2_HUMAN]	26.09	1	4	4	10	23.38	22.62	23.39	24.18	23.25	24.02	3	3	23.54	22.69	23.45	23.93	23.47	23.76	23.23	23.72	0.179	0.077	0.492
Q06210	Glutamine--fructose-6-phosphate aminotransferase [isomerizing] 1 OS=Homo sapiens GN=GFPT1 PE=1 SV=3 - [GFPT1_HUMAN]	43.35	1	19	24	72	23.68	23.16	23.95	24.75	24.49	25.36	3	3	23.84	23.23	24.01	24.50	24.71	25.10	23.69	24.77	0.022	0.017	1.079
P07814	Bifunctional glutamate/proline--tRNA ligase OS=Homo sapiens GN=EPRS PE=1 SV=5 - [SYEP_HUMAN]	33.73	1	37	38	131	23.58	23.80	24.03	25.12	25.25	25.91	3	3	23.74	23.87	24.08	24.87	25.47	25.65	23.90	25.33	0.005	0.007	1.431
P11586	C-1-tetrahydrofolate synthase, cytoplasmic OS=Homo sapiens GN=MTHFD1 PE=1 SV=3 - [C1TC_HUMAN]	61.71	1	42	42	206	24.83	24.55	24.40	26.26	26.12	26.30	3	3	24.99	24.62	24.46	26.02	26.34	26.04	24.69	26.13	0.002	0.004	1.441
P60866	40S ribosomal protein S20 OS=Homo sapiens GN=RPS20 PE=1 SV=1 - [RS20_HUMAN]	26.05	1	3	3	27	24.70	24.85	24.90	27.19	26.89	26.80	3	3	24.86	24.92	24.96	26.94	27.10	26.54	24.91	26.86	0.000	0.002	1.948
O14974	Protein phosphatase 1 regulatory subunit 12A OS=Homo sapiens GN=PPP1R12A PE=1 SV=1 - [MYPT1_HUMAN]	7.18	1	5	5	27	22.17	22.33	22.23	22.93	23.22	23.25	3	3	22.33	22.39	22.29	22.69	23.44	22.99	22.34	23.04	0.034	0.023	0.701
P39019	40S ribosomal protein S19 OS=Homo sapiens GN=RPS19 PE=1 SV=2 - [RS19_HUMAN]	46.90	1	8	8	31	24.08	24.80	24.86	26.60	26.96	26.75	3	3	24.24	24.87	24.92	26.35	27.17	26.49	24.68	26.67	0.004	0.006	1.995
Q9Y3U8	60S ribosomal protein L36 OS=Homo sapiens GN=RPL36 PE=1 SV=3 - [RL36_HUMAN]	40.95	1	9	10	42	24.52	23.96	24.57	27.17	26.64	26.61	3	3	24.69	24.02	24.63	26.92	26.86	26.35	24.45	26.71	0.001	0.004	2.265
Q15293	Reticulocalbin-1 OS=Homo sapiens GN=RCN1 PE=1 SV=1 - [RCN1_HUMAN]	43.81	1	11	11	39	23.55	24.38	24.14	25.10	25.82	26.04	3	3	23.71	24.45	24.20	24.85	26.03	25.78	24.12	25.55	0.027	0.020	1.433
Q8NAB2	Kelch repeat and BTB domain-containing protein 3 OS=Homo sapiens GN=KBTBD3 PE=2 SV=2 - [KBTB3_HUMAN]	1.15	1	1	1	11	24.73	24.80	24.85	26.68	26.33	26.88	3	3	24.89	24.87	24.91	26.43	26.54	26.62	24.89	26.53	0.000	0.001	1.641
P39023	60S ribosomal protein L3 OS=Homo sapiens GN=RPL3 PE=1 SV=2 - [RL3_HUMAN]	49.38	2	19	19	118	26.83	26.18	26.33	28.79	28.78	28.48	3	3	26.99	26.25	26.39	28.54	29.00	28.22	26.54	28.59	0.003	0.005	2.042
Q9H9H4	Vacuolar protein sorting-associated protein 37B OS=Homo sapiens GN=VPS37B PE=1 SV=1 - [VP37B_HUMAN]	89.12	1	19	19	163	28.50	28.59	28.80	26.16	25.66	26.56	3	3	28.66	28.66	28.86	25.91	25.87	26.30	28.73	26.03	0.000	0.002	-2.699
Q16222	UDP-N-acetylhexosamine pyrophosphorylase OS=Homo sapiens GN=UAP1 PE=1 SV=3 - [UAP1_HUMAN]	22.41	2	9	9	33	23.49	22.75	23.18	24.27	23.81	24.10	3	3	23.65	22.82	23.24	24.03	24.02	23.84	23.24	23.96	0.043	0.027	0.725
Q9UN37	Vacuolar protein sorting-associated protein 4A OS=Homo sapiens GN=VPS4A PE=1 SV=1 - [VPS4A_HUMAN]	52.86	2	16	20	97	27.54	27.35	27.35	25.16	24.32	25.00	3	3	27.70	27.42	27.41	24.91	24.53	24.73	27.51	24.73	0.000	0.002	-2.783
P54105	Methylosome subunit pICln OS=Homo sapiens GN=CLNS1A PE=1 SV=1 - [ICLN_HUMAN]	40.93	1	4	4	15	23.90	24.03	24.12	25.33	25.96	25.88	3	3	24.06	24.10	24.17	25.09	26.18	25.62	24.11	25.63	0.009	0.010	1.518
Q92841	Probable ATP-dependent RNA helicase DDX17 OS=Homo sapiens GN=DDX17 PE=1 SV=2 - [DDX17_HUMAN]	17.15	1	4	11	38	24.17	24.45	24.18	25.82	25.87	26.11	3	3	24.33	24.52	24.24	25.58	26.09	25.85	24.36	25.84	0.001	0.003	1.475
Q99613	Eukaryotic translation initiation factor 3 subunit C OS=Homo sapiens GN=EIF3C PE=1 SV=1 - [EIF3C_HUMAN]	27.49	3	19	19	57	22.80	21.70	22.64	24.60	23.99	24.39	3	3	22.96	21.77	22.70	24.36	24.20	24.13	22.48	24.23	0.009	0.010	1.752
Q9UK41	Vacuolar protein sorting-associated protein 28 homolog OS=Homo sapiens GN=VPS28 PE=1 SV=1 - [VPS28_HUMAN]	66.06	1	16	16	106	29.96	29.84	30.01	27.15	26.77	27.76	3	3	30.12	29.91	30.07	26.91	26.99	27.50	30.03	27.13	0.000	0.002	-2.901
Q9HAD4	WD repeat-containing protein 41 OS=Homo sapiens GN=WDR41 PE=2 SV=3 - [WDR41_HUMAN]	1.31	1	1	1	5	28.69	29.07	28.71	27.07		27.05	3	2	28.85	29.14	28.77	26.82		26.79	28.92	26.81	0.001	0.003	-2.115
P56192	Methionine--tRNA ligase, cytoplasmic OS=Homo sapiens GN=MARS PE=1 SV=2 - [SYMC_HUMAN]	40.67	1	23	23	58	21.19	21.64	22.28	24.46	24.05	25.04	3	3	21.35	21.71	22.34	24.22	24.27	24.78	21.80	24.42	0.001	0.004	2.621
P62847	40S ribosomal protein S24 OS=Homo sapiens GN=RPS24 PE=1 SV=1 - [RS24_HUMAN]	34.59	1	4	4	22	25.58	25.03	25.02	27.24	27.29	27.04	3	3	25.75	25.10	25.08	26.99	27.50	26.78	25.31	27.09	0.004	0.007	1.783
P62249	40S ribosomal protein S16 OS=Homo sapiens GN=RPS16 PE=1 SV=2 - [RS16_HUMAN]	45.21	1	8	8	34	24.55	25.11	24.74	27.27	27.10	27.21	3	3	24.71	25.18	24.80	27.02	27.32	26.95	24.89	27.10	0.000	0.002	2.204
P08621	U1 small nuclear ribonucleoprotein 70 kDa OS=Homo sapiens GN=SNRNP70 PE=1 SV=2 - [RU17_HUMAN]	34.78	1	16	16	50	23.17	22.35	22.12	25.19	24.97	24.84	3	3	23.34	22.42	22.18	24.94	25.19	24.58	22.65	24.90	0.005	0.007	2.260
A5D8V6	Vacuolar protein sorting-associated protein 37C OS=Homo sapiens GN=VPS37C PE=1 SV=2 - [VP37C_HUMAN]	28.73	1	7	7	44	26.14	25.87	26.15	23.66	22.64	23.64	3	3	26.30	25.94	26.21	23.42	22.86	23.38	26.15	23.22	0.000	0.002	-2.931
O43684	Mitotic checkpoint protein BUB3 OS=Homo sapiens GN=BUB3 PE=1 SV=1 - [BUB3_HUMAN]	46.04	1	11	11	39	23.67	23.38	23.54	24.58	24.05	24.39	3	3	23.83	23.45	23.60	24.33	24.27	24.13	23.63	24.24	0.008	0.009	0.619
Q07955	Serine/arginine-rich splicing factor 1 OS=Homo sapiens GN=SRSF1 PE=1 SV=2 - [SRSF1_HUMAN]	54.84	1	13	13	48	22.92	22.16	22.77	26.09	26.19	25.82	3	3	23.08	22.23	22.83	25.85	26.41	25.56	22.71	25.94	0.001	0.003	3.225
P17987	T-complex protein 1 subunit alpha OS=Homo sapiens GN=TCP1 PE=1 SV=1 - [TCPA_HUMAN]	67.81	1	30	30	157	24.80	24.41	24.47	26.12	25.93	26.28	3	3	24.96	24.48	24.53	25.87	26.15	26.01	24.66	26.01	0.001	0.004	1.354
Q99471	Prefoldin subunit 5 OS=Homo sapiens GN=PFDN5 PE=1 SV=2 - [PFD5_HUMAN]	53.25	1	6	6	26	22.89	23.17	23.11	24.66	24.47	24.80	3	3	23.05	23.24	23.17	24.42	24.69	24.54	23.15	24.55	0.000	0.002	1.396
P43490	Nicotinamide phosphoribosyltransferase OS=Homo sapiens GN=NAMPT PE=1 SV=1 - [NAMPT_HUMAN]	65.58	1	25	25	125	24.82	24.78	24.85	27.61	26.83	27.55	3	3	24.98	24.85	24.91	27.36	27.05	27.29	24.91	27.23	0.000	0.001	2.321
P62314	Small nuclear ribonucleoprotein Sm D1 OS=Homo sapiens GN=SNRPD1 PE=1 SV=1 - [SMD1_HUMAN]	54.62	1	4	4	39	24.60	24.06	23.62	26.43	26.02	26.08	3	3	24.76	24.13	23.68	26.18	26.24	25.82	24.19	26.08	0.005	0.007	1.888
P07305	Histone H1.0 OS=Homo sapiens GN=H1F0 PE=1 SV=3 - [H10_HUMAN]	27.84	1	5	5	30	24.30	24.41	24.20	27.90	27.10	27.02	3	3	24.46	24.48	24.26	27.65	27.32	26.76	24.40	27.24	0.000	0.002	2.841
Q16576	Histone-binding protein RBBP7 OS=Homo sapiens GN=RBBP7 PE=1 SV=1 - [RBBP7_HUMAN]	35.76	1	5	10	33	23.48	23.17	23.00	24.80	24.81	24.83	3	3	23.65	23.24	23.06	24.56	25.03	24.57	23.32	24.72	0.004	0.006	1.401
P55884	Eukaryotic translation initiation factor 3 subunit B OS=Homo sapiens GN=EIF3B PE=1 SV=3 - [EIF3B_HUMAN]	33.42	1	21	21	81	22.93	22.45	22.68	24.95	25.00	25.48	3	3	23.09	22.52	22.74	24.71	25.22	25.22	22.78	25.05	0.001	0.003	2.265
P11387	DNA topoisomerase 1 OS=Homo sapiens GN=TOP1 PE=1 SV=2 - [TOP1_HUMAN]	36.73	2	30	30	110	24.58	24.79	23.88	27.05	26.57	25.99	3	3	24.74	24.86	23.94	26.80	26.79	25.73	24.51	26.44	0.014	0.013	1.927
P49773	Histidine triad nucleotide-binding protein 1 OS=Homo sapiens GN=HINT1 PE=1 SV=2 - [HINT1_HUMAN]	78.57	1	8	8	59	24.26	24.81	24.62	26.49	26.42	26.68	3	3	24.42	24.88	24.68	26.24	26.64	26.42	24.66	26.43	0.001	0.002	1.777
P11940	Polyadenylate-binding protein 1 OS=Homo sapiens GN=PABPC1 PE=1 SV=2 - [PABP1_HUMAN]	34.59	4	13	20	132	25.83	26.17	26.33	27.02	27.04	27.30	3	3	25.99	26.24	26.39	26.78	27.26	27.04	26.21	27.03	0.011	0.011	0.820
Q13310	Polyadenylate-binding protein 4 OS=Homo sapiens GN=PABPC4 PE=1 SV=1 - [PABP4_HUMAN]	30.28	2	11	18	93	25.83	26.17	26.33	27.02	27.04	27.30	3	3	25.99	26.24	26.39	26.78	27.26	27.04	26.21	27.03	0.011	0.011	0.820
O43633	Charged multivesicular body protein 2a OS=Homo sapiens GN=CHMP2A PE=1 SV=1 - [CHM2A_HUMAN]	44.14	2	12	13	75	28.61	28.38	28.16	26.02	25.44	26.04	3	3	28.77	28.45	28.22	25.77	25.65	25.78	28.48	25.74	0.000	0.002	-2.742
O75531	Barrier-to-autointegration factor OS=Homo sapiens GN=BANF1 PE=1 SV=1 - [BAF_HUMAN]	70.79	2	7	8	59	27.44	27.00	26.10	28.92	28.94	28.13	3	3	27.61	27.07	26.16	28.68	29.16	27.87	26.95	28.57	0.045	0.028	1.625
P46777	60S ribosomal protein L5 OS=Homo sapiens GN=RPL5 PE=1 SV=3 - [RL5_HUMAN]	57.91	1	15	15	97	25.22	24.95	25.30	27.56	26.86	27.00	3	3	25.38	25.01	25.36	27.31	27.08	26.74	25.25	27.05	0.001	0.003	1.791
P22626	Heterogeneous nuclear ribonucleoproteins A2/B1 OS=Homo sapiens GN=HNRNPA2B1 PE=1 SV=2 - [ROA2_HUMAN]	57.79	1	17	18	83	25.16	25.16	25.57	27.70	27.27	27.40	3	3	25.32	25.23	25.63	27.45	27.49	27.14	25.39	27.36	0.000	0.002	1.967
P54577	Tyrosine--tRNA ligase, cytoplasmic OS=Homo sapiens GN=YARS PE=1 SV=4 - [SYYC_HUMAN]	46.59	1	23	23	67	22.67	21.94	22.43	24.70	24.53	25.27	3	3	22.83	22.01	22.49	24.45	24.75	25.01	22.44	24.74	0.001	0.004	2.294
O95340	Bifunctional 3'-phosphoadenosine 5'-phosphosulfate synthase 2 OS=Homo sapiens GN=PAPSS2 PE=1 SV=2 - [PAPS2_HUMAN]	32.25	1	8	9	39	22.82	22.46	22.88	23.82	23.85	24.04	3	3	22.98	22.53	22.94	23.57	24.07	23.78	22.82	23.81	0.008	0.009	0.989
P10809	60 kDa heat shock protein, mitochondrial OS=Homo sapiens GN=HSPD1 PE=1 SV=2 - [CH60_HUMAN]	69.11	1	35	35	174	24.14	25.94	25.90	26.20	27.55	27.42	3	3	24.30	26.01	25.96	25.96	27.76	27.16	25.42	26.96	0.118	0.056	1.537
P06576	ATP synthase subunit beta, mitochondrial OS=Homo sapiens GN=ATP5B PE=1 SV=3 - [ATPB_HUMAN]	69.94	1	25	25	146	23.73	25.11	25.63	25.43	27.07	27.02	3	3	23.89	25.18	25.69	25.19	27.29	26.76	24.92	26.41	0.145	0.066	1.492
Q13765	Nascent polypeptide-associated complex subunit alpha OS=Homo sapiens GN=NACA PE=1 SV=1 - [NACA_HUMAN]	25.58	3	4	4	27	23.11	23.85	24.17	26.46	26.73	26.55	3	3	23.27	23.92	24.23	26.21	26.95	26.28	23.81	26.48	0.002	0.004	2.673
Q09028	Histone-binding protein RBBP4 OS=Homo sapiens GN=RBBP4 PE=1 SV=3 - [RBBP4_HUMAN]	37.18	1	4	10	24	23.61	22.86	23.17	24.67	24.31	24.62	3	3	23.77	22.93	23.23	24.42	24.52	24.36	23.31	24.43	0.011	0.011	1.121
Q9UKS6	Protein kinase C and casein kinase substrate in neurons protein 3 OS=Homo sapiens GN=PACSIN3 PE=1 SV=2 - [PACN3_HUMAN]	33.49	1	13	13	61	25.47	24.63	24.81	23.67	23.28	23.45	3	3	25.63	24.70	24.86	23.42	23.49	23.18	25.07	23.37	0.005	0.007	-1.699
P83881	60S ribosomal protein L36a OS=Homo sapiens GN=RPL36A PE=1 SV=2 - [RL36A_HUMAN]	27.36	1	1	4	14	22.26	22.96	23.16	25.57	25.80	25.50	3	3	22.43	23.03	23.22	25.33	26.02	25.24	22.89	25.53	0.002	0.004	2.637
P15927	Replication protein A 32 kDa subunit OS=Homo sapiens GN=RPA2 PE=1 SV=1 - [RFA2_HUMAN]	46.30	1	8	8	25	23.03	22.67	22.92	25.13	24.79	24.46	3	3	23.20	22.74	22.98	24.89	25.00	24.19	22.97	24.69	0.004	0.006	1.723
P68104	Elongation factor 1-alpha 1 OS=Homo sapiens GN=EEF1A1 PE=1 SV=1 - [EF1A1_HUMAN]	65.58	3	22	22	377	30.07	29.96	29.93	31.45	31.68	31.89	3	3	30.23	30.03	29.99	31.20	31.90	31.62	30.08	31.57	0.002	0.005	1.490
Q00341	Vigilin OS=Homo sapiens GN=HDLBP PE=1 SV=2 - [VIGLN_HUMAN]	15.46	1	14	14	39	21.52	21.19		23.90	23.64	23.98	2	3	21.68	21.26		23.65	23.86	23.72	21.47	23.74	0.001	0.003	2.275
Q9Y3E7	Charged multivesicular body protein 3 OS=Homo sapiens GN=CHMP3 PE=1 SV=3 - [CHMP3_HUMAN]	48.20	1	9	9	45	27.74	27.49	27.40	25.27	24.70	25.44	3	3	27.90	27.56	27.46	25.03	24.92	25.18	27.64	25.04	0.000	0.002	-2.601
O00148	ATP-dependent RNA helicase DDX39A OS=Homo sapiens GN=DDX39A PE=1 SV=2 - [DX39A_HUMAN]	55.27	1	7	18	75	23.90	24.78	24.95	25.83	26.14	26.61	3	3	24.07	24.85	25.01	25.59	26.36	26.35	24.64	26.10	0.020	0.016	1.454
P55209	Nucleosome assembly protein 1-like 1 OS=Homo sapiens GN=NAP1L1 PE=1 SV=1 - [NP1L1_HUMAN]	51.66	1	13	15	148	27.57	27.38	27.38	28.89	29.14	29.25	3	3	27.73	27.45	27.44	28.65	29.35	28.99	27.54	29.00	0.003	0.005	1.458
P63173	60S ribosomal protein L38 OS=Homo sapiens GN=RPL38 PE=1 SV=2 - [RL38_HUMAN]	38.57	1	3	3	11	23.74	24.10	24.08	25.73	25.87	25.47	3	3	23.91	24.17	24.14	25.48	26.08	25.21	24.07	25.59	0.005	0.007	1.517
Q71U36	Tubulin alpha-1A chain OS=Homo sapiens GN=TUBA1A PE=1 SV=1 - [TBA1A_HUMAN]	82.04	3	2	32	334	29.63	29.41	29.48	30.56	30.33	30.72	3	3	29.79	29.48	29.54	30.31	30.55	30.46	29.61	30.44	0.002	0.004	0.834
P50454	Serpin H1 OS=Homo sapiens GN=SERPINH1 PE=1 SV=2 - [SERPH_HUMAN]	64.59	1	20	20	98	23.63	24.33	24.03	25.47	26.82	26.60	3	3	23.80	24.40	24.09	25.22	27.03	26.34	24.09	26.20	0.019	0.016	2.103
Q96KP4	Cytosolic non-specific dipeptidase OS=Homo sapiens GN=CNDP2 PE=1 SV=2 - [CNDP2_HUMAN]	64.00	1	21	21	65	23.15	23.34	23.46	25.33	24.89	25.59	3	3	23.31	23.40	23.52	25.09	25.11	25.33	23.41	25.18	0.000	0.002	1.763
P14678	Small nuclear ribonucleoprotein-associated proteins B and B' OS=Homo sapiens GN=SNRPB PE=1 SV=2 - [RSMB_HUMAN]	30.83	2	7	7	38	24.02	24.42	24.55	25.88	25.75	25.99	3	3	24.18	24.48	24.61	25.64	25.97	25.73	24.42	25.78	0.001	0.003	1.356
Q14258	E3 ubiquitin/ISG15 ligase TRIM25 OS=Homo sapiens GN=TRIM25 PE=1 SV=2 - [TRI25_HUMAN]	24.92	1	12	12	34	23.31	22.92	23.16	24.00	23.71	24.14	3	3	23.47	22.99	23.21	23.76	23.92	23.88	23.22	23.85	0.013	0.012	0.631
O75351	Vacuolar protein sorting-associated protein 4B OS=Homo sapiens GN=VPS4B PE=1 SV=2 - [VPS4B_HUMAN]	65.99	2	19	23	135	27.99	27.59	27.45	25.32	24.89	25.52	3	3	28.15	27.66	27.51	25.08	25.11	25.26	27.77	25.15	0.000	0.002	-2.623
P27797	Calreticulin OS=Homo sapiens GN=CALR PE=1 SV=1 - [CALR_HUMAN]	84.17	1	25	25	181	26.08	26.86	26.55	27.65	28.93	28.81	3	3	26.24	26.93	26.61	27.41	29.15	28.55	26.59	28.37	0.031	0.022	1.778
P42566	Epidermal growth factor receptor substrate 15 OS=Homo sapiens GN=EPS15 PE=1 SV=2 - [EPS15_HUMAN]	68.97	1	47	47	284	28.35	28.90	28.97	25.99	25.81	26.74	3	3	28.51	28.97	29.03	25.74	26.02	26.47	28.83	26.08	0.001	0.002	-2.754
P08237	ATP-dependent 6-phosphofructokinase, muscle type OS=Homo sapiens GN=PFKM PE=1 SV=2 - [PFKAM_HUMAN]	12.18	1	5	6	11	22.78	23.49	23.33	24.24	24.79	24.34	3	3	22.94	23.56	23.39	24.00	25.01	24.08	23.30	24.36	0.046	0.028	1.066
Q7LBR1	Charged multivesicular body protein 1b OS=Homo sapiens GN=CHMP1B PE=1 SV=1 - [CHM1B_HUMAN]	18.59	1	5	5	23	28.33	27.82	27.73	25.93	25.03	25.80	3	3	28.49	27.89	27.79	25.69	25.24	25.54	28.06	25.49	0.001	0.002	-2.568
P78318	Immunoglobulin-binding protein 1 OS=Homo sapiens GN=IGBP1 PE=1 SV=1 - [IGBP1_HUMAN]	18.29	1	4	4	13	19.20	18.44	19.35	21.83	21.66	21.58	3	3	19.36	18.51	19.41	21.59	21.87	21.32	19.09	21.59	0.002	0.004	2.499
O95782	AP-2 complex subunit alpha-1 OS=Homo sapiens GN=AP2A1 PE=1 SV=3 - [AP2A1_HUMAN]	53.33	1	30	40	148	26.10	26.05	27.23	24.74	24.19	25.28	3	3	26.26	26.12	27.29	24.49	24.40	25.02	26.56	24.64	0.010	0.010	-1.918
O94979	Protein transport protein Sec31A OS=Homo sapiens GN=SEC31A PE=1 SV=3 - [SC31A_HUMAN]	15.16	1	15	15	51	20.95	20.52	21.06	22.91	23.19	23.86	3	3	21.11	20.59	21.12	22.66	23.41	23.60	20.94	23.22	0.002	0.005	2.282
P62826	GTP-binding nuclear protein Ran OS=Homo sapiens GN=RAN PE=1 SV=3 - [RAN_HUMAN]	41.67	1	9	9	48	26.31	25.87	26.38	27.77	27.39	27.44	3	3	26.47	25.94	26.43	27.53	27.61	27.18	26.28	27.44	0.006	0.008	1.159
Q58FG1	Putative heat shock protein HSP 90-alpha A4 OS=Homo sapiens GN=HSP90AA4P PE=5 SV=1 - [HS904_HUMAN]	10.05	1	1	4	35	27.49	27.20	27.36	28.09	27.73	28.12	3	3	27.65	27.27	27.42	27.84	27.95	27.86	27.45	27.88	0.020	0.016	0.436
Q99816	Tumor susceptibility gene 101 protein OS=Homo sapiens GN=TSG101 PE=1 SV=2 - [TS101_HUMAN]	55.13	2	23	23	146	28.49	28.44	28.58	26.06	25.25	26.31	3	3	28.66	28.51	28.64	25.82	25.47	26.05	28.60	25.78	0.000	0.002	-2.821
P25705	ATP synthase subunit alpha, mitochondrial OS=Homo sapiens GN=ATP5A1 PE=1 SV=1 - [ATPA_HUMAN]	46.65	1	20	20	90	22.60	24.36	24.68	24.20	26.17	25.93	3	3	22.76	24.43	24.74	23.95	26.39	25.67	23.98	25.34	0.225	0.091	1.358
Q07065	Cytoskeleton-associated protein 4 OS=Homo sapiens GN=CKAP4 PE=1 SV=2 - [CKAP4_HUMAN]	48.50	2	24	24	87	22.40	23.64	23.77	24.70	25.27	26.07	3	3	22.57	23.70	23.83	24.46	25.48	25.80	23.37	25.25	0.030	0.021	1.883
O43776	Asparagine--tRNA ligase, cytoplasmic OS=Homo sapiens GN=NARS PE=1 SV=1 - [SYNC_HUMAN]	33.21	1	15	15	46	23.45	23.42	23.43	25.50	24.91	25.56	3	3	23.61	23.49	23.49	25.26	25.12	25.30	23.53	25.23	0.000	0.001	1.699
P63241	Eukaryotic translation initiation factor 5A-1 OS=Homo sapiens GN=EIF5A PE=1 SV=2 - [IF5A1_HUMAN]	69.48	3	11	11	104	27.14	27.15	27.30	27.95	28.02	28.45	3	3	27.30	27.22	27.36	27.71	28.24	28.18	27.29	28.04	0.012	0.012	0.751
P53999	Activated RNA polymerase II transcriptional coactivator p15 OS=Homo sapiens GN=SUB1 PE=1 SV=3 - [TCP4_HUMAN]	50.39	1	6	6	17	24.18	23.45	23.50	24.86	24.40	24.23	3	3	24.34	23.51	23.56	24.61	24.61	23.97	23.81	24.40	0.161	0.071	0.590
P50991	T-complex protein 1 subunit delta OS=Homo sapiens GN=CCT4 PE=1 SV=4 - [TCPD_HUMAN]	66.79	1	29	30	181	25.29	25.46	25.30	27.04	26.74	27.14	3	3	25.45	25.53	25.35	26.79	26.95	26.88	25.44	26.87	0.000	0.002	1.430
Q9BY43	Charged multivesicular body protein 4a OS=Homo sapiens GN=CHMP4A PE=1 SV=3 - [CHM4A_HUMAN]	47.30	1	10	10	43	26.56	26.35	26.31	24.26	23.81	24.58	3	3	26.72	26.41	26.37	24.02	24.03	24.32	26.50	24.12	0.000	0.002	-2.382
P61313	60S ribosomal protein L15 OS=Homo sapiens GN=RPL15 PE=1 SV=2 - [RL15_HUMAN]	42.16	1	9	9	48	25.07	25.00	25.29	27.89	27.19	27.26	3	3	25.23	25.07	25.35	27.64	27.40	27.00	25.22	27.35	0.000	0.002	2.132
P43686	26S protease regulatory subunit 6B OS=Homo sapiens GN=PSMC4 PE=1 SV=2 - [PRS6B_HUMAN]	73.21	1	22	23	92	24.56	24.08	24.22	25.71	25.74	25.55	3	3	24.72	24.15	24.28	25.46	25.96	25.29	24.38	25.57	0.011	0.011	1.187
P62857	40S ribosomal protein S28 OS=Homo sapiens GN=RPS28 PE=1 SV=1 - [RS28_HUMAN]	56.52	1	5	5	20	23.02	24.22	23.89	24.88	25.55	25.80	3	3	23.18	24.29	23.95	24.63	25.77	25.54	23.81	25.31	0.034	0.023	1.504
P43487	Ran-specific GTPase-activating protein OS=Homo sapiens GN=RANBP1 PE=1 SV=1 - [RANG_HUMAN]	54.23	1	6	6	39	24.01	23.53	24.21	25.13	25.16	25.73	3	3	24.17	23.60	24.27	24.88	25.38	25.47	24.02	25.25	0.012	0.012	1.229
P13639	Elongation factor 2 OS=Homo sapiens GN=EEF2 PE=1 SV=4 - [EF2_HUMAN]	63.64	1	48	49	350	26.39	26.37	26.55	27.60	27.22	27.77	3	3	26.55	26.44	26.61	27.35	27.44	27.51	26.53	27.43	0.000	0.002	0.902
P60900	Proteasome subunit alpha type-6 OS=Homo sapiens GN=PSMA6 PE=1 SV=1 - [PSA6_HUMAN]	52.03	1	11	11	61	23.97	24.57	24.43	25.94	25.95	26.39	3	3	24.13	24.64	24.49	25.70	26.17	26.13	24.42	26.00	0.002	0.004	1.574
P62906	60S ribosomal protein L10a OS=Homo sapiens GN=RPL10A PE=1 SV=2 - [RL10A_HUMAN]	47.47	1	11	11	52	24.09	24.57	24.88	27.21	26.74	27.31	3	3	24.25	24.64	24.93	26.97	26.95	27.05	24.61	26.99	0.000	0.002	2.382
P31948	Stress-induced-phosphoprotein 1 OS=Homo sapiens GN=STIP1 PE=1 SV=1 - [STIP1_HUMAN]	50.28	1	29	29	164	24.74	25.20	25.23	26.73	26.54	26.94	3	3	24.90	25.27	25.29	26.48	26.76	26.68	25.15	26.64	0.001	0.003	1.486
Q9BXN6	Sperm protein associated with the nucleus on the X chromosome D OS=Homo sapiens GN=SPANXD PE=1 SV=1 - [SPNXD_HUMAN]	46.39	2	1	3	16	24.82		25.66	26.20	26.16	26.99	2	3	24.98		25.72	25.96	26.37	26.73	25.35	26.35	0.086	0.045	1.001
Q15025	TNFAIP3-interacting protein 1 OS=Homo sapiens GN=TNIP1 PE=1 SV=2 - [TNIP1_HUMAN]	31.45	1	16	16	63	24.10	23.71	23.78	24.87	24.30	24.31	3	3	24.26	23.78	23.84	24.62	24.52	24.05	23.96	24.40	0.135	0.062	0.435
P41252	Isoleucine--tRNA ligase, cytoplasmic OS=Homo sapiens GN=IARS PE=1 SV=2 - [SYIC_HUMAN]	23.14	1	20	20	61	23.27	22.80	23.11	24.44	24.18	24.84	3	3	23.43	22.87	23.17	24.19	24.39	24.58	23.16	24.39	0.003	0.006	1.233
P31153	S-adenosylmethionine synthase isoform type-2 OS=Homo sapiens GN=MAT2A PE=1 SV=1 - [METK2_HUMAN]	30.13	2	8	8	31	23.63	24.12	24.27	25.55	25.44	25.75	3	3	23.79	24.19	24.33	25.30	25.66	25.49	24.10	25.48	0.002	0.004	1.383
O95084	Serine protease 23 OS=Homo sapiens GN=PRSS23 PE=1 SV=1 - [PRS23_HUMAN]	47.00	2	13	13	87	25.96	25.37	25.61	27.29	26.54	26.50	3	3	26.12	25.44	25.67	27.04	26.75	26.23	25.74	26.68	0.039	0.025	0.937
P30876	DNA-directed RNA polymerase II subunit RPB2 OS=Homo sapiens GN=POLR2B PE=1 SV=1 - [RPB2_HUMAN]	3.75	1	3	3	11	19.89	19.87	20.30	22.07	21.83	21.76	3	3	20.05	19.94	20.36	21.83	22.05	21.50	20.12	21.79	0.001	0.003	1.674
P61018	Ras-related protein Rab-4B OS=Homo sapiens GN=RAB4B PE=1 SV=1 - [RAB4B_HUMAN]	17.37	7	1	2	15	28.26	28.69	28.77	27.58	28.02	27.83	3	3	28.42	28.76	28.83	27.34	28.23	27.57	28.67	27.72	0.032	0.022	-0.952
P31939	Bifunctional purine biosynthesis protein PURH OS=Homo sapiens GN=ATIC PE=1 SV=3 - [PUR9_HUMAN]	83.11	1	38	38	178	24.36	24.69	24.17	26.53	26.34	26.64	3	3	24.53	24.76	24.23	26.28	26.55	26.38	24.50	26.40	0.000	0.002	1.900
Q5T4S7	E3 ubiquitin-protein ligase UBR4 OS=Homo sapiens GN=UBR4 PE=1 SV=1 - [UBR4_HUMAN]	5.69	1	15	15	30	21.41	20.36		23.39	22.42	23.56	2	3	21.57	20.43		23.15	22.64	23.30	21.00	23.03	0.027	0.020	2.032
Q13185	Chromobox protein homolog 3 OS=Homo sapiens GN=CBX3 PE=1 SV=4 - [CBX3_HUMAN]	30.05	2	4	4	17	22.25	22.85	23.08	24.12	24.10	24.79	3	3	22.42	22.92	23.14	23.87	24.31	24.53	22.83	24.24	0.008	0.009	1.412
Q9H444	Charged multivesicular body protein 4b OS=Homo sapiens GN=CHMP4B PE=1 SV=1 - [CHM4B_HUMAN]	58.48	3	15	15	93	29.94	29.73	29.65	27.14	26.89	27.70	3	3	30.10	29.80	29.71	26.89	27.10	27.44	29.87	27.15	0.000	0.002	-2.722
Q9P1Z9	Coiled-coil domain-containing protein 180 OS=Homo sapiens GN=CCDC180 PE=2 SV=2 - [CC180_HUMAN]	1.22	1	1	2	6	28.39	28.53	28.51	24.84	24.83		3	2	28.55	28.59	28.57	24.60	25.05		28.57	24.82	0.000	0.002	-3.747
P08670	Vimentin OS=Homo sapiens GN=VIM PE=1 SV=4 - [VIME_HUMAN]	72.75	10	45	49	350	27.59	28.29	28.41	29.64	29.65	30.12	3	3	27.75	28.36	28.47	29.40	29.87	29.86	28.19	29.71	0.005	0.007	1.516
P13674	Prolyl 4-hydroxylase subunit alpha-1 OS=Homo sapiens GN=P4HA1 PE=1 SV=2 - [P4HA1_HUMAN]	24.16	1	8	8	16	21.61	21.98	22.04	22.78	23.11	23.64	3	3	21.77	22.05	22.10	22.53	23.33	23.38	21.97	23.08	0.019	0.016	1.104
P28066	Proteasome subunit alpha type-5 OS=Homo sapiens GN=PSMA5 PE=1 SV=3 - [PSA5_HUMAN]	56.43	1	10	10	67	25.85	25.72	25.82	27.03	27.20	27.60	3	3	26.01	25.79	25.88	26.78	27.41	27.34	25.90	27.18	0.004	0.006	1.282
O95573	Long-chain-fatty-acid--CoA ligase 3 OS=Homo sapiens GN=ACSL3 PE=1 SV=3 - [ACSL3_HUMAN]	14.86	1	5	7	20	25.27	24.45	24.95	25.70	25.09	25.45	3	3	25.43	24.52	25.01	25.46	25.30	25.19	24.99	25.32	0.299	0.112	0.330
Q9BQE3	Tubulin alpha-1C chain OS=Homo sapiens GN=TUBA1C PE=1 SV=1 - [TBA1C_HUMAN]	81.96	2	3	32	331	29.63	29.41	29.42	30.56	30.33	30.72	3	3	29.79	29.48	29.48	30.31	30.55	30.46	29.58	30.44	0.002	0.005	0.854
P27708	CAD protein OS=Homo sapiens GN=CAD PE=1 SV=3 - [PYR1_HUMAN]	31.82	2	44	44	133	23.67	23.38	23.74	24.81	25.12	25.32	3	3	23.84	23.45	23.80	24.57	25.33	25.06	23.69	24.98	0.007	0.009	1.290
Q07021	Complement component 1 Q subcomponent-binding protein, mitochondrial OS=Homo sapiens GN=C1QBP PE=1 SV=1 - [C1QBP_HUMAN]	26.60	1	6	6	41	23.86	25.29	25.76	26.81	27.68	27.69	3	3	24.03	25.36	25.82	26.57	27.90	27.43	25.07	27.30	0.028	0.021	2.233
P05198	Eukaryotic translation initiation factor 2 subunit 1 OS=Homo sapiens GN=EIF2S1 PE=1 SV=3 - [IF2A_HUMAN]	57.46	1	15	15	66	23.72	23.77	23.77	25.18	25.04	25.67	3	3	23.88	23.84	23.83	24.93	25.25	25.41	23.85	25.20	0.001	0.003	1.347
Q8NI22	Multiple coagulation factor deficiency protein 2 OS=Homo sapiens GN=MCFD2 PE=1 SV=1 - [MCFD2_HUMAN]	52.05	1	3	3	12	22.96	24.12	23.98	24.17	24.91	24.99	3	3	23.12	24.19	24.04	23.92	25.13	24.73	23.79	24.59	0.174	0.075	0.806
P62714	Serine/threonine-protein phosphatase 2A catalytic subunit beta isoform OS=Homo sapiens GN=PPP2CB PE=1 SV=1 - [PP2AB_HUMAN]	55.99	2	12	13	59	25.08	24.72	24.92	25.82	25.69	26.07	3	3	25.24	24.79	24.98	25.58	25.91	25.81	25.00	25.77	0.010	0.010	0.762
P50579	Methionine aminopeptidase 2 OS=Homo sapiens GN=METAP2 PE=1 SV=1 - [MAP2_HUMAN]	20.08	1	7	7	25	21.73	21.80	22.22	22.66	22.74	22.85	3	3	21.89	21.87	22.28	22.42	22.95	22.59	22.01	22.65	0.037	0.024	0.641
Q8N1G4	Leucine-rich repeat-containing protein 47 OS=Homo sapiens GN=LRRC47 PE=1 SV=1 - [LRC47_HUMAN]	16.30	1	5	5	19	21.09	21.19	21.16	23.29	22.65	23.33	3	3	21.25	21.26	21.22	23.04	22.87	23.07	21.24	22.99	0.000	0.001	1.749
O43390	Heterogeneous nuclear ribonucleoprotein R OS=Homo sapiens GN=HNRNPR PE=1 SV=1 - [HNRPR_HUMAN]	31.44	1	11	17	59	22.99	22.21	23.28	25.35	25.42	25.45	3	3	23.15	22.28	23.34	25.10	25.64	25.19	22.92	25.31	0.003	0.005	2.390
O75131	Copine-3 OS=Homo sapiens GN=CPNE3 PE=1 SV=1 - [CPNE3_HUMAN]	42.46	4	16	17	86	23.71	24.28	24.11	25.99	25.75	26.08	3	3	23.87	24.35	24.17	25.74	25.96	25.82	24.13	25.84	0.000	0.002	1.709
O00232	26S proteasome non-ATPase regulatory subunit 12 OS=Homo sapiens GN=PSMD12 PE=1 SV=3 - [PSD12_HUMAN]	51.54	1	19	19	77	23.86	23.10	23.57	25.24	25.18	25.63	3	3	24.02	23.17	23.63	24.99	25.40	25.37	23.61	25.25	0.004	0.006	1.647
P47897	Glutamine--tRNA ligase OS=Homo sapiens GN=QARS PE=1 SV=1 - [SYQ_HUMAN]	28.00	1	15	16	47	22.54	22.02	22.03	24.25	23.96	24.61	3	3	22.70	22.09	22.09	24.00	24.18	24.35	22.29	24.18	0.001	0.003	1.883
P62280	40S ribosomal protein S11 OS=Homo sapiens GN=RPS11 PE=1 SV=3 - [RS11_HUMAN]	61.39	1	14	14	51	24.96	24.08	24.46	26.96	27.19	27.19	3	3	25.13	24.15	24.52	26.72	27.41	26.93	24.60	27.02	0.002	0.005	2.418
Q9NRX4	14 kDa phosphohistidine phosphatase OS=Homo sapiens GN=PHPT1 PE=1 SV=1 - [PHP14_HUMAN]	25.60	1	2	2	7	21.25	20.49	21.21	21.85		21.84	3	2	21.41	20.56	21.27	21.60		21.58	21.08	21.59	0.230	0.092	0.515
P62424	60S ribosomal protein L7a OS=Homo sapiens GN=RPL7A PE=1 SV=2 - [RL7A_HUMAN]	53.38	2	18	18	101	26.01	25.95	26.10	28.49	27.97	27.70	3	3	26.17	26.02	26.16	28.25	28.19	27.44	26.12	27.96	0.002	0.005	1.839
Q9UQ80	Proliferation-associated protein 2G4 OS=Homo sapiens GN=PA2G4 PE=1 SV=3 - [PA2G4_HUMAN]	55.08	1	20	20	72	23.35	23.19	22.79	25.54	25.17	25.76	3	3	23.51	23.26	22.85	25.29	25.39	25.50	23.20	25.39	0.000	0.002	2.190
Q15773	Myeloid leukemia factor 2 OS=Homo sapiens GN=MLF2 PE=1 SV=1 - [MLF2_HUMAN]	50.81	1	13	13	153	30.77	29.82	30.15	26.52	26.31	27.42	3	3	30.93	29.88	30.20	26.27	26.52	27.16	30.34	26.65	0.001	0.003	-3.686
P23396	40S ribosomal protein S3 OS=Homo sapiens GN=RPS3 PE=1 SV=2 - [RS3_HUMAN]	83.54	1	23	23	134	25.68	25.55	25.84	27.71	28.54	28.00	3	3	25.84	25.62	25.90	27.46	28.76	27.74	25.79	27.99	0.005	0.007	2.201
Q96C92	Serologically defined colon cancer antigen 3 OS=Homo sapiens GN=SDCCAG3 PE=1 SV=3 - [SDCG3_HUMAN]	32.87	1	11	11	44	25.97	26.21	26.01	22.71	22.39	23.57	3	3	26.13	26.28	26.06	22.46	22.61	23.31	26.16	22.79	0.000	0.002	-3.367
Q86YQ8	Copine-8 OS=Homo sapiens GN=CPNE8 PE=1 SV=2 - [CPNE8_HUMAN]	42.20	5	15	19	79	25.78	25.43	25.01	25.16	24.63	24.05	3	3	25.94	25.50	25.07	24.92	24.85	23.79	25.50	24.52	0.091	0.047	-0.983
P23246	Splicing factor, proline- and glutamine-rich OS=Homo sapiens GN=SFPQ PE=1 SV=2 - [SFPQ_HUMAN]	33.24	1	17	18	62	24.28	23.88	24.58	26.02	26.18	26.10	3	3	24.44	23.95	24.64	25.77	26.40	25.84	24.34	26.00	0.004	0.007	1.657

P43243	Matrin-3 OS=Homo sapiens GN=MATR3 PE=1 SV=2 - [MATR3_HUMAN]	26.92	1	15	15	40	23.50	23.85	24.25	24.90	25.62	25.39	3	3	23.66	23.92	24.31	24.65	25.83	25.13	23.96	25.20	0.034	0.023	1.238
P41091	Eukaryotic translation initiation factor 2 subunit 3 OS=Homo sapiens GN=EIF2S3 PE=1 SV=3 - [IF2G_HUMAN]	51.06	2	17	17	59	22.11	22.77	22.56	25.20	24.81	25.66	3	3	22.27	22.84	22.62	24.96	25.03	25.39	22.58	25.13	0.000	0.002	2.549
Q07866	Kinesin light chain 1 OS=Homo sapiens GN=KLC1 PE=1 SV=2 - [KLC1_HUMAN]	21.47	1	6	9	35	22.97	22.53	22.94	23.68	23.65	23.86	3	3	23.13	22.60	23.00	23.43	23.86	23.60	22.91	23.63	0.024	0.018	0.719
Q07020	60S ribosomal protein L18 OS=Homo sapiens GN=RPL18 PE=1 SV=2 - [RL18_HUMAN]	46.81	1	12	12	60	26.10	26.14	26.13	28.73	27.89	28.04	3	3	26.27	26.21	26.19	28.48	28.10	27.78	26.22	28.12	0.001	0.003	1.901
Q8IX04	Ubiquitin-conjugating enzyme E2 variant 3 OS=Homo sapiens GN=UEVLD PE=1 SV=2 - [UEVLD_HUMAN]	70.70	3	25	25	112	27.41	27.54	27.47	24.62	23.77	24.99	3	3	27.57	27.61	27.53	24.38	23.99	24.73	27.57	24.36	0.000	0.002	-3.205
P17858	ATP-dependent 6-phosphofructokinase, liver type OS=Homo sapiens GN=PFKL PE=1 SV=6 - [PFKAL_HUMAN]	25.51	1	8	10	29	22.90	23.36	23.67	24.49	24.39	25.21	3	3	23.06	23.43	23.73	24.24	24.60	24.95	23.40	24.60	0.013	0.013	1.193
P49458	Signal recognition particle 9 kDa protein OS=Homo sapiens GN=SRP9 PE=1 SV=2 - [SRP09_HUMAN]	47.67	1	4	4	22	26.02	26.92	27.15	25.00	25.29	25.71	3	3	26.18	26.98	27.21	24.75	25.51	25.45	26.79	25.24	0.017	0.015	-1.556
P53621	Coatomer subunit alpha OS=Homo sapiens GN=COPA PE=1 SV=2 - [COPA_HUMAN]	30.72	1	30	30	99	23.17	22.98	23.64	25.18	24.45	24.97	3	3	23.34	23.05	23.70	24.94	24.66	24.71	23.36	24.77	0.002	0.005	1.408
O94973	AP-2 complex subunit alpha-2 OS=Homo sapiens GN=AP2A2 PE=1 SV=2 - [AP2A2_HUMAN]	43.13	1	19	29	84	25.12	24.71	25.75	24.02	23.95	24.99	3	3	25.28	24.77	25.81	23.78	24.17	24.73	25.29	24.23	0.059	0.034	-1.063
O00560	Syntenin-1 OS=Homo sapiens GN=SDCBP PE=1 SV=1 - [SDCB1_HUMAN]	71.14	1	15	15	170	30.92	31.03	30.98	29.14	28.34	29.02	3	3	31.08	31.10	31.03	28.89	28.56	28.76	31.07	28.74	0.000	0.001	-2.333
Q13363	C-terminal-binding protein 1 OS=Homo sapiens GN=CTBP1 PE=1 SV=2 - [CTBP1_HUMAN]	7.50	1	1	1	12	22.61	22.41	22.87	23.55	23.88	24.01	3	3	22.77	22.48	22.93	23.30	24.09	23.75	22.73	23.72	0.020	0.016	0.988
Q99661	Kinesin-like protein KIF2C OS=Homo sapiens GN=KIF2C PE=1 SV=2 - [KIF2C_HUMAN]	10.48	1	5	5	10	19.94	19.68		21.77	21.42	21.95	2	3	20.10	19.75		21.53	21.64	21.69	19.92	21.62	0.001	0.004	1.694
O60841	Eukaryotic translation initiation factor 5B OS=Homo sapiens GN=EIF5B PE=1 SV=4 - [IF2P_HUMAN]	18.36	1	18	18	55	22.76	22.89	23.13	24.29	23.85	24.43	3	3	22.92	22.96	23.19	24.05	24.06	24.17	23.03	24.09	0.000	0.002	1.067
P61758	Prefoldin subunit 3 OS=Homo sapiens GN=VBP1 PE=1 SV=3 - [PFD3_HUMAN]	30.96	1	4	4	22	23.14	23.78	24.02	25.35	25.24	25.60	3	3	23.30	23.85	24.08	25.10	25.45	25.34	23.74	25.30	0.003	0.006	1.555
Q9BRA0	N-alpha-acetyltransferase 38, NatC auxiliary subunit OS=Homo sapiens GN=NAA38 PE=1 SV=1 - [LSMD1_HUMAN]	16.80	1	2	2	15	18.17	17.62	18.01	19.40	19.59	19.77	3	3	18.33	17.69	18.07	19.15	19.81	19.50	18.03	19.49	0.005	0.007	1.458
Q16543	Hsp90 co-chaperone Cdc37 OS=Homo sapiens GN=CDC37 PE=1 SV=1 - [CDC37_HUMAN]	48.15	1	14	14	75	25.41	25.46	25.70	26.53	26.75	27.44	3	3	25.57	25.52	25.76	26.28	26.97	27.18	25.62	26.81	0.013	0.013	1.194
P19105	Myosin regulatory light chain 12A OS=Homo sapiens GN=MYL12A PE=1 SV=2 - [ML12A_HUMAN]	56.73	3	12	12	93	25.68	26.05	26.11	26.59	27.13	27.55	3	3	25.84	26.12	26.17	26.34	27.34	27.29	26.04	26.99	0.050	0.030	0.947
P38646	Stress-70 protein, mitochondrial OS=Homo sapiens GN=HSPA9 PE=1 SV=2 - [GRP75_HUMAN]	47.28	1	28	28	148	24.44	25.11	25.96	26.56	27.11	27.29	3	3	24.60	25.18	26.02	26.32	27.32	27.03	25.27	26.89	0.033	0.023	1.625
O15372	Eukaryotic translation initiation factor 3 subunit H OS=Homo sapiens GN=EIF3H PE=1 SV=1 - [EIF3H_HUMAN]	40.34	1	11	11	48	23.35	23.33	23.24	24.77	24.58	25.18	3	3	23.51	23.40	23.30	24.52	24.80	24.92	23.40	24.75	0.001	0.002	1.344
Q71DI3	Histone H3.2 OS=Homo sapiens GN=HIST2H3A PE=1 SV=3 - [H32_HUMAN]	60.29	3	1	10	78	30.09	30.12	29.37	32.05	31.35	30.87	3	3	30.25	30.19	29.43	31.81	31.57	30.61	29.96	31.33	0.038	0.025	1.373
P84243	Histone H3.3 OS=Homo sapiens GN=H3F3A PE=1 SV=2 - [H33_HUMAN]	60.29	3	1	10	68	30.09	30.12	29.37	32.05	31.35	30.87	3	3	30.25	30.19	29.43	31.81	31.57	30.61	29.96	31.33	0.038	0.025	1.373
P05787	Keratin, type II cytoskeletal 8 OS=Homo sapiens GN=KRT8 PE=1 SV=7 - [K2C8_HUMAN]	66.67	6	23	32	93	25.98	26.32	26.92	27.52	27.91	27.92	3	3	26.15	26.39	26.98	27.27	28.12	27.66	26.51	27.68	0.028	0.021	1.178
Q99615	DnaJ homolog subfamily C member 7 OS=Homo sapiens GN=DNAJC7 PE=1 SV=2 - [DNJC7_HUMAN]	30.77	1	13	13	34	22.73	22.80	22.54	23.81	23.56	23.79	3	3	22.89	22.87	22.60	23.57	23.78	23.53	22.79	23.63	0.002	0.005	0.841
P35232	Prohibitin OS=Homo sapiens GN=PHB PE=1 SV=1 - [PHB_HUMAN]	73.53	1	15	15	79	22.42	24.47	24.70	24.55	26.05	26.04	3	3	22.58	24.54	24.76	24.30	26.27	25.78	23.96	25.45	0.176	0.076	1.494
P08238	Heat shock protein HSP 90-beta OS=Homo sapiens GN=HSP90AB1 PE=1 SV=4 - [HS90B_HUMAN]	68.51	3	35	54	415	29.37	29.08	29.30	30.09	29.72	30.29	3	3	29.53	29.15	29.36	29.84	29.94	30.03	29.35	29.94	0.009	0.010	0.587
Q99873	Protein arginine N-methyltransferase 1 OS=Homo sapiens GN=PRMT1 PE=1 SV=2 - [ANM1_HUMAN]	52.91	2	15	15	38	23.42	23.62	23.73	25.07	24.64	25.03	3	3	23.58	23.69	23.78	24.82	24.86	24.77	23.69	24.82	0.000	0.002	1.130
P52597	Heterogeneous nuclear ribonucleoprotein F OS=Homo sapiens GN=HNRNPF PE=1 SV=3 - [HNRPF_HUMAN]	42.41	1	9	11	66	24.20	23.59	23.79	25.80	26.16	26.25	3	3	24.36	23.66	23.85	25.55	26.38	25.99	23.96	25.97	0.003	0.005	2.017
Q8NBJ5	Procollagen galactosyltransferase 1 OS=Homo sapiens GN=COLGALT1 PE=1 SV=1 - [GT251_HUMAN]	24.76	2	11	11	39	21.51	21.42	21.91	23.20	24.15	24.51	3	3	21.67	21.49	21.97	22.96	24.36	24.25	21.71	23.86	0.010	0.011	2.148
P22234	Multifunctional protein ADE2 OS=Homo sapiens GN=PAICS PE=1 SV=3 - [PUR6_HUMAN]	58.35	1	24	24	132	25.70	25.81	25.99	26.85	26.58	27.23	3	3	25.86	25.88	26.04	26.60	26.79	26.97	25.93	26.79	0.002	0.004	0.857
P78527	DNA-dependent protein kinase catalytic subunit OS=Homo sapiens GN=PRKDC PE=1 SV=3 - [PRKDC_HUMAN]	17.76	1	55	55	124	21.63	22.04	22.38	23.91	25.71	25.86	3	3	21.79	22.11	22.44	23.66	25.93	25.60	22.11	25.06	0.016	0.014	2.952
O94826	Mitochondrial import receptor subunit TOM70 OS=Homo sapiens GN=TOMM70A PE=1 SV=1 - [TOM70_HUMAN]	14.80	1	6	6	16		22.06	22.40		23.20	23.61	2	2		22.13	22.46		23.41	23.35	22.29	23.38	0.024	0.018	1.085
Q99460	26S proteasome non-ATPase regulatory subunit 1 OS=Homo sapiens GN=PSMD1 PE=1 SV=2 - [PSMD1_HUMAN]	42.71	1	25	25	109	23.67	23.36	23.86	24.86	24.44	25.54	3	3	23.83	23.43	23.92	24.61	24.66	25.27	23.73	24.85	0.013	0.012	1.122
P21399	Cytoplasmic aconitate hydratase OS=Homo sapiens GN=ACO1 PE=1 SV=3 - [ACOC_HUMAN]	34.98	1	20	20	55	22.67	22.63	22.18	24.83	24.39	25.02	3	3	22.84	22.70	22.24	24.58	24.61	24.75	22.59	24.65	0.000	0.002	2.055
P68366	Tubulin alpha-4A chain OS=Homo sapiens GN=TUBA4A PE=1 SV=1 - [TBA4A_HUMAN]	61.61	2	6	27	282	29.17	29.24	29.20	30.04	30.08	30.46	3	3	29.33	29.31	29.26	29.79	30.29	30.20	29.30	30.10	0.007	0.009	0.792
P48643	T-complex protein 1 subunit epsilon OS=Homo sapiens GN=CCT5 PE=1 SV=1 - [TCPE_HUMAN]	71.72	1	35	36	207	26.00	25.61	25.56	27.06	27.05	27.30	3	3	26.16	25.68	25.62	26.81	27.26	27.04	25.82	27.04	0.005	0.007	1.220
P52566	Rho GDP-dissociation inhibitor 2 OS=Homo sapiens GN=ARHGDIB PE=1 SV=3 - [GDIR2_HUMAN]	63.68	1	11	11	60	24.81	25.33	25.28	27.26	26.88	27.75	3	3	24.98	25.39	25.34	27.01	27.10	27.49	25.24	27.20	0.001	0.002	1.963
Q15717	ELAV-like protein 1 OS=Homo sapiens GN=ELAVL1 PE=1 SV=2 - [ELAV1_HUMAN]	24.23	3	7	7	26	22.45	22.44	22.22	23.89	23.77	24.28	3	3	22.61	22.51	22.28	23.65	23.99	24.02	22.47	23.89	0.001	0.003	1.420
P47985	Cytochrome b-c1 complex subunit Rieske, mitochondrial OS=Homo sapiens GN=UQCRFS1 PE=1 SV=2 - [UCRI_HUMAN]	13.87	2	3	3	11		22.07	22.34		22.81	23.12	2	2		22.14	22.40		23.03	22.86	22.27	22.95	0.047	0.029	0.679
P62888	60S ribosomal protein L30 OS=Homo sapiens GN=RPL30 PE=1 SV=2 - [RL30_HUMAN]	63.48	1	7	8	44	23.22	24.62	24.43	27.03	27.07	27.11	3	3	23.38	24.69	24.49	26.78	27.29	26.85	24.19	26.97	0.003	0.005	2.789
Q96EY5	Multivesicular body subunit 12A OS=Homo sapiens GN=MVB12A PE=1 SV=1 - [MB12A_HUMAN]	43.96	1	8	8	61	27.46	28.23	28.03	24.96	24.55	25.60	3	3	27.63	28.30	28.09	24.72	24.77	25.34	28.00	24.94	0.000	0.002	-3.062
P02751	Fibronectin OS=Homo sapiens GN=FN1 PE=1 SV=4 - [FINC_HUMAN]	50.92	1	82	82	523	30.49	29.39	30.49	26.66	26.89	26.42	3	3	30.65	29.46	30.55	26.42	27.11	26.16	30.22	26.56	0.002	0.004	-3.659
Q99436	Proteasome subunit beta type-7 OS=Homo sapiens GN=PSMB7 PE=1 SV=1 - [PSB7_HUMAN]	70.40	1	12	12	48	24.24	23.87	23.86	25.78	25.95	26.40	3	3	24.40	23.94	23.92	25.53	26.17	26.14	24.09	25.95	0.002	0.004	1.859
Q06323	Proteasome activator complex subunit 1 OS=Homo sapiens GN=PSME1 PE=1 SV=1 - [PSME1_HUMAN]	57.03	1	14	14	51	23.04	22.93	22.44	25.65	24.99	25.81	3	3	23.20	23.00	22.50	25.40	25.21	25.55	22.90	25.39	0.000	0.002	2.486
O14965	Aurora kinase A OS=Homo sapiens GN=AURKA PE=1 SV=2 - [AURKA_HUMAN]	68.98	1	20	21	90	26.17	26.45	26.50	23.87	25.02	24.23	3	3	26.33	26.52	26.55	23.63	25.24	23.97	26.47	24.28	0.011	0.012	-2.192
P40227	T-complex protein 1 subunit zeta OS=Homo sapiens GN=CCT6A PE=1 SV=3 - [TCPZ_HUMAN]	68.36	2	28	28	155	25.18	24.93	24.52	26.45	26.37	26.52	3	3	25.34	25.00	24.58	26.20	26.58	26.26	24.97	26.35	0.005	0.007	1.373
Q9UNF0	Protein kinase C and casein kinase substrate in neurons protein 2 OS=Homo sapiens GN=PACSIN2 PE=1 SV=2 - [PACN2_HUMAN]	36.42	1	14	14	68	26.24	26.15	26.28	24.22	23.52	24.67	3	3	26.40	26.22	26.33	23.97	23.74	24.41	26.32	24.04	0.000	0.002	-2.280
P53990	IST1 homolog OS=Homo sapiens GN=IST1 PE=1 SV=1 - [IST1_HUMAN]	53.30	1	15	15	99	28.64	28.70	28.71	26.48	25.50	26.60	3	3	28.81	28.77	28.77	26.23	25.72	26.34	28.78	26.10	0.000	0.002	-2.687
Q9UL46	Proteasome activator complex subunit 2 OS=Homo sapiens GN=PSME2 PE=1 SV=4 - [PSME2_HUMAN]	52.72	1	10	10	56	23.27	23.02	22.94	25.23	24.57	25.48	3	3	23.43	23.08	22.99	24.98	24.78	25.22	23.17	24.99	0.001	0.003	1.823
P53618	Coatomer subunit beta OS=Homo sapiens GN=COPB1 PE=1 SV=3 - [COPB_HUMAN]	27.81	1	20	20	60	23.60	23.09	23.27	25.05	24.95	25.67	3	3	23.76	23.16	23.33	24.80	25.17	25.41	23.42	25.13	0.002	0.005	1.710
O96000	NADH dehydrogenase [ubiquinone] 1 beta subcomplex subunit 10 OS=Homo sapiens GN=NDUFB10 PE=1 SV=3 - [NDUBA_HUMAN]	19.77	1	3	3	7		21.42	21.29	20.51	22.77	22.56	2	3		21.49	21.35	20.27	22.99	22.30	21.42	21.85	0.710	0.221	0.431
Q96NE9	FERM domain-containing protein 6 OS=Homo sapiens GN=FRMD6 PE=1 SV=1 - [FRMD6_HUMAN]	16.40	1	6	6	23	22.66	21.91	22.75	20.75	20.17	21.60	3	3	22.82	21.98	22.81	20.50	20.39	21.34	22.54	20.74	0.012	0.012	-1.795
O43143	Pre-mRNA-splicing factor ATP-dependent RNA helicase DHX15 OS=Homo sapiens GN=DHX15 PE=1 SV=2 - [DHX15_HUMAN]	53.21	3	34	34	232	26.74	26.97	27.16	25.41	25.31	26.16	3	3	26.90	27.04	27.22	25.16	25.52	25.90	27.05	25.53	0.003	0.005	-1.527
P05362	Intercellular adhesion molecule 1 OS=Homo sapiens GN=ICAM1 PE=1 SV=2 - [ICAM1_HUMAN]	41.92	1	16	16	92	24.16	24.20	23.59	27.47	26.81	27.89	3	3	24.32	24.27	23.65	27.22	27.02	27.63	24.08	27.29	0.000	0.002	3.211
Q15437	Protein transport protein Sec23B OS=Homo sapiens GN=SEC23B PE=1 SV=2 - [SC23B_HUMAN]	14.08	1	6	6	16	22.71	22.55	22.68	23.99	23.41	24.12	3	3	22.87	22.62	22.74	23.75	23.62	23.86	22.74	23.74	0.001	0.002	1.000
P28074	Proteasome subunit beta type-5 OS=Homo sapiens GN=PSMB5 PE=1 SV=3 - [PSB5_HUMAN]	52.09	1	11	11	54	22.73	23.26	23.47	24.37	24.23	24.87	3	3	22.89	23.33	23.53	24.13	24.45	24.61	23.25	24.39	0.008	0.009	1.146
Q99426	Tubulin-folding cofactor B OS=Homo sapiens GN=TBCB PE=1 SV=2 - [TBCB_HUMAN]	42.21	1	8	8	24	21.75	22.47	22.45	23.77	23.68	24.30	3	3	21.91	22.54	22.51	23.52	23.90	24.04	22.32	23.82	0.004	0.007	1.500
P14927	Cytochrome b-c1 complex subunit 7 OS=Homo sapiens GN=UQCRB PE=1 SV=2 - [QCR7_HUMAN]	42.34	1	4	4	13		23.51	23.52		24.19	24.15	2	2		23.58	23.57		24.40	23.89	23.58	24.15	0.160	0.071	0.566
O43399	Tumor protein D54 OS=Homo sapiens GN=TPD52L2 PE=1 SV=2 - [TPD54_HUMAN]	61.65	1	11	11	42	24.42	23.93	25.17	26.51	25.97	26.44	3	3	24.58	24.00	25.22	26.26	26.19	26.18	24.60	26.21	0.011	0.011	1.608
O75367	Core histone macro-H2A.1 OS=Homo sapiens GN=H2AFY PE=1 SV=4 - [H2AY_HUMAN]	45.43	2	16	16	83	26.45	26.56	25.53	28.95	28.20	27.20	3	3	26.61	26.62	25.59	28.70	28.42	26.94	26.28	28.02	0.054	0.031	1.743
Q14498	RNA-binding protein 39 OS=Homo sapiens GN=RBM39 PE=1 SV=2 - [RBM39_HUMAN]	24.15	2	10	10	27	20.49	21.32	21.15	24.39	24.25	23.50	3	3	20.66	21.39	21.21	24.14	24.47	23.24	21.08	23.95	0.003	0.005	2.866
P46060	Ran GTPase-activating protein 1 OS=Homo sapiens GN=RANGAP1 PE=1 SV=1 - [RAGP1_HUMAN]	26.58	1	12	12	24	22.03	20.74	22.07	24.03	23.43	23.71	3	3	22.19	20.81	22.13	23.78	23.65	23.45	21.71	23.63	0.014	0.013	1.914
P30084	Enoyl-CoA hydratase, mitochondrial OS=Homo sapiens GN=ECHS1 PE=1 SV=4 - [ECHM_HUMAN]	24.14	1	5	5	19	22.41	22.78	22.81	23.26	23.33	23.71	3	3	22.58	22.85	22.87	23.02	23.54	23.45	22.77	23.34	0.039	0.025	0.572
Q9NS25	Sperm protein associated with the nucleus on the X chromosome B1 OS=Homo sapiens GN=SPANXB1 PE=2 SV=2 - [SPNXB_HUMAN]	77.67	1	4	6	41	25.33	24.25	24.77	26.65	26.50	26.67	3	3	25.49	24.32	24.83	26.41	26.72	26.41	24.88	26.51	0.010	0.011	1.626
Q9NTK5	Obg-like ATPase 1 OS=Homo sapiens GN=OLA1 PE=1 SV=2 - [OLA1_HUMAN]	49.75	1	15	15	50	23.81	23.70	23.66	25.25	24.60	25.13	3	3	23.97	23.77	23.72	25.00	24.81	24.87	23.82	24.90	0.000	0.002	1.076
P50897	Palmitoyl-protein thioesterase 1 OS=Homo sapiens GN=PPT1 PE=1 SV=1 - [PPT1_HUMAN]	31.37	1	5	5	22	23.29	22.44	21.96	24.53	24.16	24.07	3	3	23.45	22.51	22.02	24.28	24.38	23.81	22.66	24.16	0.031	0.022	1.496
P17980	26S protease regulatory subunit 6A OS=Homo sapiens GN=PSMC3 PE=1 SV=3 - [PRS6A_HUMAN]	54.67	1	18	18	115	25.10	24.61	24.55	26.27	26.47	26.51	3	3	25.26	24.68	24.61	26.03	26.69	26.25	24.85	26.32	0.007	0.008	1.470
O43852	Calumenin OS=Homo sapiens GN=CALU PE=1 SV=2 - [CALU_HUMAN]	67.94	1	16	16	73	23.40	24.02	23.97	24.73	26.09	26.21	3	3	23.57	24.09	24.03	24.49	26.31	25.95	23.89	25.58	0.044	0.027	1.688
P60228	Eukaryotic translation initiation factor 3 subunit E OS=Homo sapiens GN=EIF3E PE=1 SV=1 - [EIF3E_HUMAN]	31.91	1	11	11	44	23.79	23.48	23.63	24.95	24.52	25.30	3	3	23.95	23.55	23.69	24.71	24.74	25.04	23.73	24.83	0.002	0.005	1.100
P56537	Eukaryotic translation initiation factor 6 OS=Homo sapiens GN=EIF6 PE=1 SV=1 - [IF6_HUMAN]	72.65	1	8	8	57	25.16	25.03	25.04	26.93	26.06	26.64	3	3	25.33	25.10	25.10	26.68	26.28	26.38	25.18	26.45	0.001	0.003	1.273
P54619	5'-AMP-activated protein kinase subunit gamma-1 OS=Homo sapiens GN=PRKAG1 PE=1 SV=1 - [AAKG1_HUMAN]	17.82	1	3	3	15	21.01	20.47		22.15	21.69	22.61	2	3	21.17	20.54		21.90	21.91	22.35	20.86	22.05	0.029	0.021	1.194
Q99961	Endophilin-A2 OS=Homo sapiens GN=SH3GL1 PE=1 SV=1 - [SH3G1_HUMAN]	40.22	3	14	14	71	26.22	25.59	26.33	24.02	23.28	24.95	3	3	26.38	25.66	26.39	23.78	23.50	24.69	26.14	23.99	0.008	0.009	-2.154
Q9Y266	Nuclear migration protein nudC OS=Homo sapiens GN=NUDC PE=1 SV=1 - [NUDC_HUMAN]	51.36	1	16	16	67	24.42	24.72	24.48	26.28	25.87	26.40	3	3	24.58	24.79	24.54	26.03	26.08	26.14	24.63	26.08	0.000	0.002	1.450
Q9BRG1	Vacuolar protein-sorting-associated protein 25 OS=Homo sapiens GN=VPS25 PE=1 SV=1 - [VPS25_HUMAN]	72.16	1	11	11	33	25.82	25.76	25.97	24.40	23.34	23.80	3	3	25.98	25.83	26.03	24.15	23.55	23.54	25.95	23.75	0.000	0.002	-2.199
Q14764	Major vault protein OS=Homo sapiens GN=MVP PE=1 SV=4 - [MVP_HUMAN]	60.13	1	43	43	187	25.50	24.71	23.90	27.28	25.99	26.09	3	3	25.66	24.78	23.96	27.03	26.20	25.83	24.80	26.36	0.062	0.035	1.557
P55036	26S proteasome non-ATPase regulatory subunit 4 OS=Homo sapiens GN=PSMD4 PE=1 SV=1 - [PSMD4_HUMAN]	17.51	2	4	4	13	23.44	23.40	23.44	24.81	24.56	25.36	3	3	23.61	23.47	23.50	24.57	24.78	25.10	23.52	24.82	0.001	0.004	1.293
O95630	STAM-binding protein OS=Homo sapiens GN=STAMBP PE=1 SV=1 - [STABP_HUMAN]	43.87	1	15	15	78	26.50	27.05	26.92	24.43	24.09	25.11	3	3	26.66	27.12	26.98	24.18	24.31	24.85	26.92	24.45	0.001	0.002	-2.474
P05387	60S acidic ribosomal protein P2 OS=Homo sapiens GN=RPLP2 PE=1 SV=1 - [RLA2_HUMAN]	84.35	1	5	7	46	25.42	27.02	26.84	29.14	29.36	29.35	3	3	25.58	27.09	26.90	28.89	29.58	29.09	26.52	29.18	0.007	0.008	2.661
Q15370	Transcription elongation factor B polypeptide 2 OS=Homo sapiens GN=TCEB2 PE=1 SV=1 - [ELOB_HUMAN]	84.75	1	7	7	25	24.53	24.62	24.46	25.40	25.24	25.52	3	3	24.69	24.69	24.52	25.15	25.45	25.26	24.63	25.29	0.003	0.006	0.655
P39748	Flap endonuclease 1 OS=Homo sapiens GN=FEN1 PE=1 SV=1 - [FEN1_HUMAN]	35.53	1	8	8	34	22.22	23.41	21.71	24.49	25.45	25.01	3	3	22.38	23.48	21.77	24.24	25.67	24.75	22.54	24.89	0.023	0.018	2.343
Q12846	Syntaxin-4 OS=Homo sapiens GN=STX4 PE=1 SV=2 - [STX4_HUMAN]	45.12	1	9	9	35	23.33	23.08	22.85	24.96	24.08	24.54	3	3	23.49	23.15	22.91	24.72	24.30	24.28	23.18	24.43	0.005	0.007	1.246
P37235	Hippocalcin-like protein 1 OS=Homo sapiens GN=HPCAL1 PE=1 SV=3 - [HPCL1_HUMAN]	49.74	3	10	10	58	24.30	24.73	24.59	26.20	25.87	26.49	3	3	24.46	24.80	24.65	25.96	26.09	26.23	24.64	26.09	0.000	0.002	1.454
P09496	Clathrin light chain A OS=Homo sapiens GN=CLTA PE=1 SV=1 - [CLCA_HUMAN]	22.98	2	8	8	57	24.67	25.52	25.05	27.20	26.98	27.12	3	3	24.83	25.59	25.11	26.95	27.20	26.86	25.18	27.00	0.002	0.004	1.827
Q99832	T-complex protein 1 subunit eta OS=Homo sapiens GN=CCT7 PE=1 SV=2 - [TCPH_HUMAN]	63.54	1	28	28	150	25.27	25.24	25.05	26.45	26.38	26.84	3	3	25.43	25.31	25.11	26.20	26.59	26.58	25.28	26.46	0.002	0.004	1.177
P02545	Prelamin-A/C OS=Homo sapiens GN=LMNA PE=1 SV=1 - [LMNA_HUMAN]	51.96	1	31	32	110	22.88	25.69	23.49	24.88	26.93	25.60	3	3	23.04	25.76	23.55	24.64	27.15	25.34	24.12	25.71	0.228	0.092	1.593
Q15181	Inorganic pyrophosphatase OS=Homo sapiens GN=PPA1 PE=1 SV=2 - [IPYR_HUMAN]	68.51	1	13	14	97	25.28	24.67	24.68	26.19	25.88	26.25	3	3	25.44	24.74	24.74	25.95	26.10	25.99	24.97	26.01	0.012	0.012	1.037
P11388	DNA topoisomerase 2-alpha OS=Homo sapiens GN=TOP2A PE=1 SV=3 - [TOP2A_HUMAN]	11.04	2	12	12	36	20.89	21.38	21.56	23.30	22.95	23.11	3	3	21.05	21.45	21.62	23.06	23.17	22.85	21.37	23.03	0.001	0.003	1.651
P63151	Serine/threonine-protein phosphatase 2A 55 kDa regulatory subunit B alpha isoform OS=Homo sapiens GN=PPP2R2A PE=1 SV=1 - [2ABA_HUMAN]	34.90	4	10	10	27	23.15	22.41	22.71	24.29	24.37	24.53	3	3	23.32	22.48	22.77	24.05	24.59	24.27	22.86	24.30	0.008	0.009	1.446
P23284	Peptidyl-prolyl cis-trans isomerase B OS=Homo sapiens GN=PPIB PE=1 SV=2 - [PPIB_HUMAN]	54.63	1	14	14	70	23.98	25.62	25.40	26.29	26.96	27.22	3	3	24.14	25.69	25.46	26.05	27.18	26.96	25.10	26.73	0.052	0.030	1.629
Q9H0H5	Rac GTPase-activating protein 1 OS=Homo sapiens GN=RACGAP1 PE=1 SV=1 - [RGAP1_HUMAN]	41.14	2	21	21	109	26.33	26.02	26.63	25.65	25.45	25.67	3	3	26.49	26.09	26.69	25.41	25.66	25.40	26.43	25.49	0.009	0.010	-0.934
P28072	Proteasome subunit beta type-6 OS=Homo sapiens GN=PSMB6 PE=1 SV=4 - [PSB6_HUMAN]	38.49	1	6	6	30	23.21	23.89	23.52	25.77	25.46	26.23	3	3	23.37	23.96	23.58	25.52	25.68	25.96	23.64	25.72	0.001	0.003	2.087
Q7L1Q6	Basic leucine zipper and W2 domain-containing protein 1 OS=Homo sapiens GN=BZW1 PE=1 SV=1 - [BZW1_HUMAN]	48.45	1	12	15	51	24.12	23.61	24.18	24.91	24.99	25.28	3	3	24.28	23.68	24.24	24.66	25.21	25.02	24.07	24.96	0.023	0.018	0.895
P09543	2',3'-cyclic-nucleotide 3'-phosphodiesterase OS=Homo sapiens GN=CNP PE=1 SV=2 - [CN37_HUMAN]	75.53	2	38	40	377	30.44	30.65	30.61	27.88	26.97	28.38	3	3	30.60	30.72	30.67	27.63	27.19	28.12	30.66	27.65	0.000	0.002	-3.015
Q15369	Transcription elongation factor B polypeptide 1 OS=Homo sapiens GN=TCEB1 PE=1 SV=1 - [ELOC_HUMAN]	49.11	1	4	4	20	22.99	23.22	23.27	23.94	23.84	24.35	3	3	23.15	23.29	23.33	23.70	24.05	24.09	23.26	23.95	0.007	0.009	0.693
Q9NZN3	EH domain-containing protein 3 OS=Homo sapiens GN=EHD3 PE=1 SV=2 - [EHD3_HUMAN]	39.81	2	3	20	108	26.80	26.35	26.32	25.50	25.20	25.58	3	3	26.96	26.42	26.38	25.26	25.41	25.31	26.59	25.33	0.003	0.005	-1.258
Q08431	Lactadherin OS=Homo sapiens GN=MFGE8 PE=1 SV=2 - [MFGM_HUMAN]	76.49	1	25	25	174	29.03	28.82	29.09	28.12	27.34	28.09	3	3	29.19	28.89	29.15	27.88	27.56	27.83	29.08	27.76	0.001	0.003	-1.319
P13473	Lysosome-associated membrane glycoprotein 2 OS=Homo sapiens GN=LAMP2 PE=1 SV=2 - [LAMP2_HUMAN]	7.07	1	4	4	23	24.80	25.31	24.37	26.37	26.26	26.00	3	3	24.96	25.38	24.43	26.13	26.48	25.74	24.92	26.11	0.026	0.020	1.190
P40926	Malate dehydrogenase, mitochondrial OS=Homo sapiens GN=MDH2 PE=1 SV=3 - [MDHM_HUMAN]	55.62	1	14	14	71	22.80	24.52	24.54	26.34	26.61	27.04	3	3	22.96	24.59	24.60	26.09	26.83	26.78	24.05	26.57	0.013	0.013	2.518
Q13283	Ras GTPase-activating protein-binding protein 1 OS=Homo sapiens GN=G3BP1 PE=1 SV=1 - [G3BP1_HUMAN]	19.53	1	6	6	27	23.89	23.27	23.58	24.78	24.82	24.97	3	3	24.05	23.34	23.63	24.53	25.04	24.71	23.68	24.76	0.013	0.013	1.084
Q9UI30	Multifunctional methyltransferase subunit TRM112-like protein OS=Homo sapiens GN=TRMT112 PE=1 SV=1 - [TR112_HUMAN]	42.40	1	4	4	18	23.20	23.13	22.78	23.98	24.04	24.08	3	3	23.36	23.20	22.84	23.73	24.26	23.82	23.13	23.93	0.024	0.018	0.802
P54136	Arginine--tRNA ligase, cytoplasmic OS=Homo sapiens GN=RARS PE=1 SV=2 - [SYRC_HUMAN]	45.76	1	26	26	76	21.48	22.27	23.05	24.95	24.56	25.20	3	3	21.65	22.34	23.11	24.70	24.78	24.93	22.36	24.80	0.005	0.007	2.439
Q9UJZ1	Stomatin-like protein 2, mitochondrial OS=Homo sapiens GN=STOML2 PE=1 SV=1 - [STML2_HUMAN]	48.88	1	13	13	37	21.12	22.36	22.95	22.88	25.24	24.90	3	3	21.28	22.43	23.01	22.64	25.46	24.64	22.24	24.25	0.110	0.053	2.006
Q99497	Protein deglycase DJ-1 OS=Homo sapiens GN=PARK7 PE=1 SV=2 - [PARK7_HUMAN]	62.43	1	11	11	53	24.98	24.84	24.70	25.98	25.78	26.21	3	3	25.14	24.91	24.76	25.74	25.99	25.95	24.93	25.89	0.002	0.005	0.961
Q15018	BRISC complex subunit Abro1 OS=Homo sapiens GN=FAM175B PE=1 SV=2 - [F175B_HUMAN]	2.65	1	1	1	6	20.40	20.57	20.44	21.70	21.57	22.18	3	3	20.56	20.64	20.50	21.45	21.79	21.92	20.57	21.72	0.001	0.004	1.153
Q14697	Neutral alpha-glucosidase AB OS=Homo sapiens GN=GANAB PE=1 SV=3 - [GANAB_HUMAN]	54.56	1	35	35	155	23.87	24.71	24.36	25.40	25.80	26.29	3	3	24.03	24.78	24.42	25.15	26.01	26.03	24.41	25.73	0.021	0.017	1.322
Q13151	Heterogeneous nuclear ribonucleoprotein A0 OS=Homo sapiens GN=HNRNPA0 PE=1 SV=1 - [ROA0_HUMAN]	35.74	1	5	6	17	23.17	22.87	23.35	24.72	24.40	24.26	3	3	23.33	22.94	23.41	24.47	24.62	24.00	23.23	24.36	0.009	0.010	1.135
Q969Q0	60S ribosomal protein L36a-like OS=Homo sapiens GN=RPL36AL PE=1 SV=3 - [RL36L_HUMAN]	27.36	1	1	4	14	22.26	22.62	23.40	25.95	25.41	25.49	3	3	22.43	22.69	23.46	25.70	25.63	25.23	22.86	25.52	0.001	0.004	2.660
Q13347	Eukaryotic translation initiation factor 3 subunit I OS=Homo sapiens GN=EIF3I PE=1 SV=1 - [EIF3I_HUMAN]	82.46	1	20	20	65	24.29	22.44	23.28	26.32	26.07	26.53	3	3	24.45	22.51	23.34	26.08	26.29	26.27	23.43	26.21	0.008	0.009	2.780
P68431	Histone H3.1 OS=Homo sapiens GN=HIST1H3A PE=1 SV=2 - [H31_HUMAN]	60.29	3	1	10	96	30.09	30.24	29.37	32.05	31.35	30.87	3	3	30.25	30.31	29.43	31.81	31.57	30.61	30.00	31.33	0.045	0.028	1.331
Q15149	Plectin OS=Homo sapiens GN=PLEC PE=1 SV=3 - [PLEC_HUMAN]	41.42	1	158	163	471	24.46	23.98	23.93	25.69	26.08	26.05	3	3	24.62	24.05	23.99	25.44	26.30	25.79	24.22	25.84	0.007	0.009	1.620
P35606	Coatomer subunit beta' OS=Homo sapiens GN=COPB2 PE=1 SV=2 - [COPB2_HUMAN]	18.43	1	12	12	32	23.36	22.41	22.99	24.31	23.88	24.70	3	3	23.53	22.48	23.05	24.07	24.10	24.44	23.02	24.20	0.022	0.017	1.183
P51114	Fragile X mental retardation syndrome-related protein 1 OS=Homo sapiens GN=FXR1 PE=1 SV=3 - [FXR1_HUMAN]	14.49	2	6	8	20	22.22	21.80	22.51	23.63	23.99	23.86	3	3	22.38	21.87	22.57	23.39	24.20	23.60	22.28	23.73	0.011	0.011	1.454
Q14204	Cytoplasmic dynein 1 heavy chain 1 OS=Homo sapiens GN=DYNC1H1 PE=1 SV=5 - [DYHC1_HUMAN]	40.55	2	141	142	568	24.75	24.31	24.77	25.40	24.78	25.61	3	3	24.91	24.38	24.83	25.16	24.99	25.35	24.70	25.16	0.078	0.041	0.460
P49321	Nuclear autoantigenic sperm protein OS=Homo sapiens GN=NASP PE=1 SV=2 - [NASP_HUMAN]	24.87	1	12	12	33	20.73		21.08	23.38	24.29	23.95	2	3	20.90		21.14	23.14	24.50	23.69	21.02	23.78	0.013	0.012	2.760
Q9NP72	Ras-related protein Rab-18 OS=Homo sapiens GN=RAB18 PE=1 SV=1 - [RAB18_HUMAN]	64.08	1	10	10	38	24.17	23.83	23.93	24.93	24.44	24.98	3	3	24.33	23.90	23.99	24.68	24.65	24.72	24.08	24.69	0.010	0.011	0.612
Q14166	Tubulin--tyrosine ligase-like protein 12 OS=Homo sapiens GN=TTLL12 PE=1 SV=2 - [TTL12_HUMAN]	13.82	1	5	5	14	21.98	20.75	21.94	23.14	22.76	23.94	3	3	22.14	20.82	22.00	22.89	22.98	23.68	21.65	23.18	0.035	0.023	1.532
P62191	26S protease regulatory subunit 4 OS=Homo sapiens GN=PSMC1 PE=1 SV=1 - [PRS4_HUMAN]	68.64	1	20	22	120	25.69	25.17	25.17	26.52	26.61	26.59	3	3	25.85	25.24	25.23	26.27	26.83	26.33	25.44	26.48	0.019	0.016	1.039
P21333	Filamin-A OS=Homo sapiens GN=FLNA PE=1 SV=4 - [FLNA_HUMAN]	56.82	1	100	112	666	25.85	26.31	26.20	26.91	26.89	27.13	3	3	26.01	26.38	26.26	26.66	27.11	26.87	26.22	26.88	0.017	0.015	0.663
Q15233	Non-POU domain-containing octamer-binding protein OS=Homo sapiens GN=NONO PE=1 SV=4 - [NONO_HUMAN]	39.28	1	13	15	56	24.35	23.96	23.88	25.60	26.13	26.09	3	3	24.51	24.03	23.94	25.36	26.35	25.83	24.16	25.85	0.007	0.009	1.684
Q9NP79	Vacuolar protein sorting-associated protein VTA1 homolog OS=Homo sapiens GN=VTA1 PE=1 SV=1 - [VTA1_HUMAN]	39.41	1	11	11	53	27.19	27.33	27.28	25.68	24.90	25.75	3	3	27.35	27.40	27.34	25.44	25.12	25.48	27.37	25.35	0.000	0.002	-2.019
P02771	Alpha-fetoprotein OS=Homo sapiens GN=AFP PE=1 SV=1 - [FETA_HUMAN]	9.03	1	1	5	18	28.18	28.66	28.81	26.05	25.06	26.40	3	3	28.34	28.73	28.87	25.80	25.28	26.14	28.65	25.74	0.001	0.003	-2.909
Q9UKK9	ADP-sugar pyrophosphatase OS=Homo sapiens GN=NUDT5 PE=1 SV=1 - [NUDT5_HUMAN]	42.47	1	9	9	30	22.69	23.88	23.14	25.80	25.60	25.90	3	3	22.85	23.95	23.20	25.56	25.81	25.64	23.33	25.67	0.002	0.005	2.337
P46926	Glucosamine-6-phosphate isomerase 1 OS=Homo sapiens GN=GNPDA1 PE=1 SV=1 - [GNPI1_HUMAN]	41.52	2	6	6	20		23.52	23.57	23.64	23.93	24.02	2	3		23.59	23.63	23.39	24.14	23.76	23.61	23.76	0.624	0.202	0.152
Q9Y625	Glypican-6 OS=Homo sapiens GN=GPC6 PE=1 SV=1 - [GPC6_HUMAN]	29.01	2	11	11	37	25.47	25.33	25.55	23.39		23.64	3	2	25.63	25.40	25.61	23.14		23.38	25.55	23.26	0.000	0.002	-2.288
Q9Y5V3	Melanoma-associated antigen D1 OS=Homo sapiens GN=MAGED1 PE=1 SV=3 - [MAGD1_HUMAN]	3.47	1	1	2	7	23.13	21.51	21.46	24.99	24.64	24.84	3	3	23.29	21.58	21.52	24.75	24.86	24.58	22.13	24.73	0.011	0.012	2.597
P28838	Cytosol aminopeptidase OS=Homo sapiens GN=LAP3 PE=1 SV=3 - [AMPL_HUMAN]	40.08	1	16	16	57	21.70		21.47	25.32	24.97	25.42	2	3	21.86		21.53	25.08	25.19	25.16	21.70	25.14	0.000	0.002	3.443
P05067	Amyloid beta A4 protein OS=Homo sapiens GN=APP PE=1 SV=3 - [A4_HUMAN]	16.88	1	8	8	28	22.84	22.63	21.99	23.73	23.33	22.47	3	3	23.00	22.70	22.04	23.48	23.54	22.21	22.58	23.08	0.389	0.137	0.500

O00567	Nucleolar protein 56 OS=Homo sapiens GN=NOP56 PE=1 SV=4 - [NOP56_HUMAN]	40.24	1	19	19	53	21.64	22.93	23.39	24.26	24.84	24.86	3	3	21.80	23.00	23.45	24.01	25.06	24.60	22.75	24.56	0.036	0.024	1.805
Q9UQ16	Dynamin-3 OS=Homo sapiens GN=DNM3 PE=1 SV=4 - [DYN3_HUMAN]	10.59	1	1	8	22	24.34	24.11	23.96	25.10	25.09	25.32	3	3	24.51	24.18	24.02	24.86	25.31	25.06	24.24	25.07	0.012	0.012	0.839
P52272	Heterogeneous nuclear ribonucleoprotein M OS=Homo sapiens GN=HNRNPM PE=1 SV=3 - [HNRPM_HUMAN]	60.41	1	34	34	110	23.82	23.77	24.00	25.36	26.59	26.21	3	3	23.98	23.84	24.06	25.12	26.81	25.95	23.96	25.96	0.015	0.014	1.995
P24539	ATP synthase F(0) complex subunit B1, mitochondrial OS=Homo sapiens GN=ATP5F1 PE=1 SV=2 - [AT5F1_HUMAN]	7.81	1	2	2	9	20.88	23.02	23.02	21.95	23.67	24.20	3	3	21.04	23.09	23.08	21.70	23.89	23.94	22.41	23.18	0.486	0.166	0.771
O75832	26S proteasome non-ATPase regulatory subunit 10 OS=Homo sapiens GN=PSMD10 PE=1 SV=1 - [PSD10_HUMAN]	19.91	1	3	3	10	21.30	21.69	21.23	23.57	23.18	23.97	3	3	21.46	21.76	21.29	23.32	23.40	23.71	21.50	23.48	0.000	0.002	1.975
P46778	60S ribosomal protein L21 OS=Homo sapiens GN=RPL21 PE=1 SV=2 - [RL21_HUMAN]	25.62	1	5	5	29	25.06	25.36	25.51	27.68	27.34	26.95	3	3	25.22	25.43	25.57	27.43	27.55	26.69	25.41	27.22	0.003	0.006	1.817
P53004	Biliverdin reductase A OS=Homo sapiens GN=BLVRA PE=1 SV=2 - [BIEA_HUMAN]	9.46	1	2	2	6	21.90	20.95		23.33	22.26	23.47	2	3	22.06	21.02		23.08	22.47	23.20	21.54	22.92	0.066	0.036	1.382
P12268	Inosine-5'-monophosphate dehydrogenase 2 OS=Homo sapiens GN=IMPDH2 PE=1 SV=2 - [IMDH2_HUMAN]	60.51	2	26	26	161	26.21	26.26	25.57	27.33	28.09	27.65	3	3	26.37	26.33	25.63	27.09	28.30	27.39	26.11	27.59	0.027	0.020	1.486
Q01813	ATP-dependent 6-phosphofructokinase, platelet type OS=Homo sapiens GN=PFKP PE=1 SV=2 - [PFKAP_HUMAN]	51.79	1	29	31	197	24.91	24.99	24.94	26.45	25.99	26.80	3	3	25.08	25.06	24.99	26.21	26.20	26.54	25.04	26.32	0.000	0.002	1.275
Q9GZZ1	N-alpha-acetyltransferase 50 OS=Homo sapiens GN=NAA50 PE=1 SV=1 - [NAA50_HUMAN]	43.20	1	6	6	14	22.28	22.27	22.13	23.32	23.10	23.62	3	3	22.44	22.34	22.19	23.08	23.32	23.36	22.32	23.25	0.001	0.004	0.926
P36957	Dihydrolipoyllysine-residue succinyltransferase component of 2-oxoglutarate dehydrogenase complex, mitochondrial OS=Homo sapiens GN=DLST PE=1 SV=4 - [ODO2_HUMAN]	28.26	1	9	9	28	19.25	21.39	22.33	21.55	24.50	24.04	3	3	19.41	21.46	22.39	21.31	24.72	23.78	21.09	23.27	0.180	0.077	2.182
Q8WUM4	Programmed cell death 6-interacting protein OS=Homo sapiens GN=PDCD6IP PE=1 SV=1 - [PDC6I_HUMAN]	63.36	1	55	55	413	29.59	29.65	29.67	27.44	26.35	27.74	3	3	29.75	29.72	29.73	27.19	26.56	27.48	29.73	27.08	0.001	0.003	-2.656
Q96AE4	Far upstream element-binding protein 1 OS=Homo sapiens GN=FUBP1 PE=1 SV=3 - [FUBP1_HUMAN]	8.07	1	4	4	11	20.35	20.73	21.29	22.13	21.83	23.34	3	3	20.52	20.80	21.35	21.89	22.05	23.08	20.89	22.34	0.031	0.022	1.451
Q01518	Adenylyl cyclase-associated protein 1 OS=Homo sapiens GN=CAP1 PE=1 SV=5 - [CAP1_HUMAN]	51.79	1	23	24	186	26.22	26.12	25.95	27.42	26.99	27.55	3	3	26.38	26.19	26.01	27.17	27.20	27.29	26.19	27.22	0.001	0.003	1.030
O95757	Heat shock 70 kDa protein 4L OS=Homo sapiens GN=HSPA4L PE=1 SV=3 - [HS74L_HUMAN]	23.72	1	10	14	37	24.80	24.68	24.38	26.09	25.93	26.44	3	3	24.97	24.75	24.44	25.84	26.15	26.18	24.72	26.06	0.002	0.004	1.340
Q13045	Protein flightless-1 homolog OS=Homo sapiens GN=FLII PE=1 SV=2 - [FLII_HUMAN]	11.98	1	10	10	21		20.49	20.59	22.96	23.07	23.42	2	3		20.56	20.65	22.71	23.28	23.16	20.60	23.05	0.002	0.004	2.447
Q9Y3A5	Ribosome maturation protein SBDS OS=Homo sapiens GN=SBDS PE=1 SV=4 - [SBDS_HUMAN]	45.60	1	9	9	19	22.03	22.71	22.54	22.43	23.30	23.30	3	3	22.19	22.78	22.60	22.19	23.51	23.04	22.52	22.91	0.412	0.143	0.389
Q92633	Lysophosphatidic acid receptor 1 OS=Homo sapiens GN=LPAR1 PE=1 SV=3 - [LPAR1_HUMAN]	3.57	1	1	1	5	24.59		24.88	23.43	21.77	23.60	2	3	24.75		24.94	23.18	21.99	23.34	24.84	22.84	0.036	0.024	-2.007
P62195	26S protease regulatory subunit 8 OS=Homo sapiens GN=PSMC5 PE=1 SV=1 - [PRS8_HUMAN]	65.52	1	19	21	93	24.99	24.43	24.47	25.97	26.22	26.18	3	3	25.15	24.50	24.53	25.72	26.44	25.92	24.73	26.03	0.013	0.012	1.300
O14818	Proteasome subunit alpha type-7 OS=Homo sapiens GN=PSMA7 PE=1 SV=1 - [PSA7_HUMAN]	56.45	2	12	12	60	24.69	25.22	24.62	26.43	26.33	26.71	3	3	24.86	25.29	24.68	26.18	26.54	26.45	24.94	26.39	0.002	0.005	1.450
Q9BRA2	Thioredoxin domain-containing protein 17 OS=Homo sapiens GN=TXNDC17 PE=1 SV=1 - [TXD17_HUMAN]	38.21	1	4	4	22	23.90	24.02	24.10	25.53	24.97	25.86	3	3	24.06	24.09	24.16	25.29	25.18	25.60	24.10	25.36	0.001	0.003	1.251
P08729	Keratin, type II cytoskeletal 7 OS=Homo sapiens GN=KRT7 PE=1 SV=5 - [K2C7_HUMAN]	15.99	7	4	9	28	25.81	26.79	26.92	27.49	27.89	27.88	3	3	25.97	26.86	26.98	27.24	28.11	27.62	26.60	27.66	0.060	0.034	1.055
Q9UNF1	Melanoma-associated antigen D2 OS=Homo sapiens GN=MAGED2 PE=1 SV=2 - [MAGD2_HUMAN]	21.95	2	11	12	45	23.05	21.33	21.95	24.50	24.08	24.31	3	3	23.21	21.40	22.01	24.26	24.30	24.05	22.21	24.20	0.021	0.017	1.993
Q9UQN3	Charged multivesicular body protein 2b OS=Homo sapiens GN=CHMP2B PE=1 SV=1 - [CHM2B_HUMAN]	23.47	2	6	6	22	26.24	25.92	26.08	23.62	23.02	24.55	3	3	26.40	25.99	26.14	23.37	23.23	24.29	26.18	23.63	0.002	0.004	-2.547
Q07617	Sperm-associated antigen 1 OS=Homo sapiens GN=SPAG1 PE=1 SV=3 - [SPAG1_HUMAN]	6.80	1	3	4	12	21.04	21.16	21.17	22.79	22.10	22.45	3	3	21.20	21.23	21.23	22.54	22.31	22.19	21.22	22.35	0.000	0.002	1.131
P20618	Proteasome subunit beta type-1 OS=Homo sapiens GN=PSMB1 PE=1 SV=2 - [PSB1_HUMAN]	61.83	1	11	11	62	23.06	24.12	24.29	25.39	25.58	25.67	3	3	23.22	24.19	24.35	25.14	25.80	25.41	23.92	25.45	0.019	0.016	1.529
Q14152	Eukaryotic translation initiation factor 3 subunit A OS=Homo sapiens GN=EIF3A PE=1 SV=1 - [EIF3A_HUMAN]	30.54	1	32	32	87	23.48	22.63	22.57	24.90	24.36	25.12	3	3	23.64	22.70	22.63	24.65	24.58	24.86	22.99	24.70	0.007	0.009	1.706
P78371	T-complex protein 1 subunit beta OS=Homo sapiens GN=CCT2 PE=1 SV=4 - [TCPB_HUMAN]	80.93	1	33	33	242	26.28	25.16	25.37	27.51	27.46	27.57	3	3	26.44	25.23	25.43	27.26	27.67	27.31	25.70	27.42	0.012	0.012	1.713
Q15427	Splicing factor 3B subunit 4 OS=Homo sapiens GN=SF3B4 PE=1 SV=1 - [SF3B4_HUMAN]	19.58	1	4	4	16		22.50	22.99	24.12	24.14	24.48	2	3		22.57	23.05	23.87	24.35	24.22	22.81	24.15	0.014	0.013	1.338
Q92925	SWI/SNF-related matrix-associated actin-dependent regulator of chromatin subfamily D member 2 OS=Homo sapiens GN=SMARCD2 PE=1 SV=3 - [SMRD2_HUMAN]	3.77	1	1	1	6	20.12	20.43	19.84	21.75	21.28	21.24	3	3	20.28	20.50	19.90	21.50	21.50	20.97	20.23	21.32	0.012	0.012	1.098
P04406	Glyceraldehyde-3-phosphate dehydrogenase OS=Homo sapiens GN=GAPDH PE=1 SV=3 - [G3P_HUMAN]	74.63	1	17	18	297	30.09	29.84	29.49	30.94	30.73	30.93	3	3	30.25	29.91	29.55	30.69	30.94	30.67	29.90	30.77	0.017	0.015	0.863
Q92598	Heat shock protein 105 kDa OS=Homo sapiens GN=HSPH1 PE=1 SV=1 - [HS105_HUMAN]	48.83	3	30	32	154	25.14	25.09	24.99	26.49	26.95	27.53	3	3	25.30	25.16	25.05	26.24	27.17	27.27	25.17	26.89	0.007	0.008	1.722
P09132	Signal recognition particle 19 kDa protein OS=Homo sapiens GN=SRP19 PE=1 SV=3 - [SRP19_HUMAN]	28.47	1	3	3	15	20.30	21.28	21.48	22.82	22.71	23.10	3	3	20.47	21.35	21.54	22.58	22.93	22.84	21.12	22.78	0.009	0.010	1.662
P26196	Probable ATP-dependent RNA helicase DDX6 OS=Homo sapiens GN=DDX6 PE=1 SV=2 - [DDX6_HUMAN]	22.98	1	7	7	19	22.80	22.59	23.17	23.16	23.30	23.77	3	3	22.96	22.65	23.23	22.92	23.51	23.51	22.95	23.31	0.232	0.093	0.364
P20700	Lamin-B1 OS=Homo sapiens GN=LMNB1 PE=1 SV=2 - [LMNB1_HUMAN]	37.54	1	16	19	39	20.46	21.14	22.53	22.37	24.55	24.77	3	3	20.62	21.21	22.59	22.12	24.77	24.51	21.47	23.80	0.086	0.045	2.328
Q92783	Signal transducing adapter molecule 1 OS=Homo sapiens GN=STAM PE=1 SV=3 - [STAM1_HUMAN]	30.37	1	10	12	53	24.25	24.19	23.22	25.38	24.82	24.41	3	3	24.41	24.26	23.28	25.13	25.04	24.14	23.98	24.77	0.172	0.075	0.788
P62263	40S ribosomal protein S14 OS=Homo sapiens GN=RPS14 PE=1 SV=3 - [RS14_HUMAN]	66.89	1	8	8	52	25.26	24.35	25.25	27.37	28.03	27.59	3	3	25.43	24.42	25.30	27.12	28.25	27.33	25.05	27.57	0.006	0.008	2.516
P23258	Tubulin gamma-1 chain OS=Homo sapiens GN=TUBG1 PE=1 SV=2 - [TBG1_HUMAN]	20.40	2	4	4	14	22.78	22.52	22.60	23.70	23.53		3	2	22.94	22.59	22.66	23.45	23.74		22.73	23.60	0.016	0.015	0.866
P78504	Protein jagged-1 OS=Homo sapiens GN=JAG1 PE=1 SV=3 - [JAG1_HUMAN]	2.38	1	3	3	20	22.83	23.47	22.95	21.42	21.37	21.81	3	3	22.99	23.54	23.01	21.17	21.59	21.55	23.18	21.44	0.001	0.004	-1.746
P11413	Glucose-6-phosphate 1-dehydrogenase OS=Homo sapiens GN=G6PD PE=1 SV=4 - [G6PD_HUMAN]	46.41	1	20	20	52	22.64	22.15	21.66	25.21	24.05	25.26	3	3	22.80	22.22	21.72	24.97	24.27	25.00	22.25	24.74	0.003	0.005	2.495
Q9HAC8	Ubiquitin domain-containing protein 1 OS=Homo sapiens GN=UBTD1 PE=1 SV=1 - [UBTD1_HUMAN]	19.82	1	3	4	12	24.25	24.07	24.00	23.43		23.26	3	2	24.41	24.14	24.06	23.18		23.00	24.20	23.09	0.005	0.007	-1.112
P51858	Hepatoma-derived growth factor OS=Homo sapiens GN=HDGF PE=1 SV=1 - [HDGF_HUMAN]	33.75	1	6	6	15	22.08	21.21	20.76	23.91	23.53	24.15	3	3	22.24	21.28	20.82	23.66	23.74	23.89	21.45	23.76	0.005	0.007	2.314
P18859	ATP synthase-coupling factor 6, mitochondrial OS=Homo sapiens GN=ATP5J PE=1 SV=1 - [ATP5J_HUMAN]	38.89	1	3	3	8		23.00	23.53	22.60	24.02	24.67	2	3		23.07	23.59	22.36	24.24	24.40	23.33	23.67	0.724	0.225	0.337
P61513	60S ribosomal protein L37a OS=Homo sapiens GN=RPL37A PE=1 SV=2 - [RL37A_HUMAN]	51.09	1	4	4	14	22.54	23.86	23.86	24.77	25.03	25.51	3	3	22.70	23.93	23.92	24.53	25.24	25.25	23.52	25.01	0.035	0.023	1.489
P49840	Glycogen synthase kinase-3 alpha OS=Homo sapiens GN=GSK3A PE=1 SV=2 - [GSK3A_HUMAN]	51.14	1	11	19	71	26.31	26.52	26.50	23.25	22.47	24.42	3	3	26.47	26.59	26.56	23.01	22.68	24.16	26.54	23.28	0.002	0.004	-3.255
P50570	Dynamin-2 OS=Homo sapiens GN=DNM2 PE=1 SV=2 - [DYN2_HUMAN]	42.41	2	20	27	87	24.73	24.07	24.13	25.64	25.78	25.86	3	3	24.89	24.14	24.18	25.39	26.00	25.59	24.40	25.66	0.014	0.013	1.257
Q15691	Microtubule-associated protein RP/EB family member 1 OS=Homo sapiens GN=MAPRE1 PE=1 SV=3 - [MARE1_HUMAN]	69.40	1	13	14	59	23.87	23.94	23.92	24.91	24.51	25.03	3	3	24.04	24.01	23.98	24.66	24.73	24.77	24.01	24.72	0.000	0.002	0.710
P67870	Casein kinase II subunit beta OS=Homo sapiens GN=CSNK2B PE=1 SV=1 - [CSK2B_HUMAN]	49.30	1	7	7	45	24.48	24.48	23.79	26.05	25.45	25.72	3	3	24.64	24.55	23.85	25.80	25.66	25.46	24.35	25.64	0.008	0.009	1.295
O00571	ATP-dependent RNA helicase DDX3X OS=Homo sapiens GN=DDX3X PE=1 SV=3 - [DDX3X_HUMAN]	41.39	3	23	24	108	24.79	24.55	24.60	25.47	25.49	25.91	3	3	24.95	24.62	24.65	25.22	25.70	25.65	24.74	25.53	0.013	0.013	0.785
P55072	Transitional endoplasmic reticulum ATPase OS=Homo sapiens GN=VCP PE=1 SV=4 - [TERA_HUMAN]	72.70	2	58	58	371	25.87	26.32	26.39	27.30	27.04	27.61	3	3	26.03	26.39	26.45	27.06	27.26	27.35	26.29	27.22	0.004	0.006	0.931
Q9NR12	PDZ and LIM domain protein 7 OS=Homo sapiens GN=PDLIM7 PE=1 SV=1 - [PDLI7_HUMAN]	33.92	1	11	11	25	23.32	24.40	24.38	22.40	23.21	22.64	3	3	23.48	24.47	24.44	22.15	23.43	22.38	24.13	22.65	0.044	0.028	-1.475
P19623	Spermidine synthase OS=Homo sapiens GN=SRM PE=1 SV=1 - [SPEE_HUMAN]	39.07	1	8	8	32	23.48	23.26	23.21	24.55	24.06	24.76	3	3	23.64	23.33	23.27	24.31	24.28	24.50	23.41	24.36	0.002	0.004	0.947
P63167	Dynein light chain 1, cytoplasmic OS=Homo sapiens GN=DYNLL1 PE=1 SV=1 - [DYL1_HUMAN]	58.43	1	2	5	58	28.43	28.89	28.94	26.33	25.96	27.44	3	3	28.59	28.96	29.00	26.09	26.18	27.18	28.85	26.48	0.003	0.005	-2.368
P51659	Peroxisomal multifunctional enzyme type 2 OS=Homo sapiens GN=HSD17B4 PE=1 SV=3 - [DHB4_HUMAN]	22.69	1	11	11	21		21.79	22.42	21.80	23.21	23.70	2	3		21.86	22.48	21.56	23.42	23.44	22.17	22.81	0.504	0.171	0.635
Q9H2G2	STE20-like serine/threonine-protein kinase OS=Homo sapiens GN=SLK PE=1 SV=1 - [SLK_HUMAN]	8.58	1	6	7	31	21.63	21.13	22.13	22.73	22.90	23.10	3	3	21.79	21.20	22.19	22.49	23.11	22.84	21.72	22.81	0.033	0.022	1.089
P30101	Protein disulfide-isomerase A3 OS=Homo sapiens GN=PDIA3 PE=1 SV=4 - [PDIA3_HUMAN]	56.24	1	21	22	134	25.55	25.88	25.63	26.61	27.75	27.75	3	3	25.71	25.95	25.69	26.36	27.97	27.49	25.78	27.27	0.037	0.024	1.491
P35998	26S protease regulatory subunit 7 OS=Homo sapiens GN=PSMC2 PE=1 SV=3 - [PRS7_HUMAN]	54.04	1	20	20	87	24.68	23.97	24.31	25.71	26.05	26.01	3	3	24.84	24.03	24.37	25.46	26.27	25.75	24.41	25.83	0.013	0.013	1.413
P22061	Protein-L-isoaspartate(D-aspartate) O-methyltransferase OS=Homo sapiens GN=PCMT1 PE=1 SV=4 - [PIMT_HUMAN]	30.84	1	5	5	15	23.85	23.46	23.59	24.55	23.94	24.55	3	3	24.01	23.53	23.65	24.31	24.15	24.29	23.73	24.25	0.027	0.020	0.519
Q96FW1	Ubiquitin thioesterase OTUB1 OS=Homo sapiens GN=OTUB1 PE=1 SV=2 - [OTUB1_HUMAN]	35.79	1	8	8	28	22.52	22.11	22.25	23.64	23.52	24.40	3	3	22.68	22.18	22.31	23.40	23.74	24.14	22.39	23.76	0.006	0.008	1.368
P16989	Y-box-binding protein 3 OS=Homo sapiens GN=YBX3 PE=1 SV=4 - [YBOX3_HUMAN]	11.83	2	1	4	16	24.20	24.97	24.29	25.99	25.90	26.11	3	3	24.36	25.04	24.34	25.74	26.12	25.85	24.58	25.90	0.007	0.008	1.322
P00505	Aspartate aminotransferase, mitochondrial OS=Homo sapiens GN=GOT2 PE=1 SV=3 - [AATM_HUMAN]	40.00	1	13	13	38		22.81	23.49	25.12	25.05	25.49	2	3		22.88	23.55	24.88	25.27	25.23	23.22	25.13	0.008	0.009	1.907
P68371	Tubulin beta-4B chain OS=Homo sapiens GN=TUBB4B PE=1 SV=1 - [TBB4B_HUMAN]	82.02	3	1	28	409	29.82	29.36	29.83	30.48	30.51	31.19	3	3	29.98	29.42	29.89	30.24	30.72	30.93	29.76	30.63	0.032	0.022	0.868
P04350	Tubulin beta-4A chain OS=Homo sapiens GN=TUBB4A PE=1 SV=2 - [TBB4A_HUMAN]	76.80	1	1	25	370	29.82	29.36	29.83	30.48	30.51	31.19	3	3	29.98	29.42	29.89	30.24	30.72	30.93	29.76	30.63	0.032	0.022	0.868
P49720	Proteasome subunit beta type-3 OS=Homo sapiens GN=PSMB3 PE=1 SV=2 - [PSB3_HUMAN]	33.17	1	5	5	37	23.87	24.25	23.70	25.08	25.33	25.67	3	3	24.03	24.32	23.76	24.83	25.55	25.41	24.04	25.26	0.011	0.011	1.228
Q9BUL8	Programmed cell death protein 10 OS=Homo sapiens GN=PDCD10 PE=1 SV=1 - [PDC10_HUMAN]	51.89	1	8	8	31	24.23	24.00	23.83	24.62	24.50	24.58	3	3	24.40	24.07	23.89	24.37	24.72	24.32	24.12	24.47	0.144	0.065	0.352
O00429	Dynamin-1-like protein OS=Homo sapiens GN=DNM1L PE=1 SV=2 - [DNM1L_HUMAN]	31.11	1	15	15	44	22.63	22.29	21.79	24.53	23.45	24.18	3	3	22.79	22.36	21.85	24.28	23.66	23.92	22.33	23.95	0.008	0.009	1.622
P13489	Ribonuclease inhibitor OS=Homo sapiens GN=RNH1 PE=1 SV=2 - [RINI_HUMAN]	70.28	1	19	19	137	25.16	25.13	25.15	26.16	26.16	26.92	3	3	25.32	25.20	25.21	25.91	26.38	26.66	25.24	26.32	0.008	0.009	1.073
O75947	ATP synthase subunit d, mitochondrial OS=Homo sapiens GN=ATP5H PE=1 SV=3 - [ATP5H_HUMAN]	40.99	1	6	6	14		22.63	22.64		24.04	24.23	2	2		22.70	22.70		24.26	23.97	22.70	24.11	0.010	0.011	1.411
P99999	Cytochrome c OS=Homo sapiens GN=CYCS PE=1 SV=2 - [CYC_HUMAN]	55.24	2	6	6	21	22.04	22.11		23.26	23.48	24.74	2	3	22.20	22.18		23.01	23.69	24.48	22.19	23.73	0.067	0.037	1.540
P17096	High mobility group protein HMG-I/HMG-Y OS=Homo sapiens GN=HMGA1 PE=1 SV=3 - [HMGA1_HUMAN]	41.12	1	8	8	52	24.71	25.47	25.45	28.43	27.95	27.35	3	3	24.87	25.54	25.51	28.18	28.16	27.09	25.31	27.81	0.004	0.006	2.505
O60884	DnaJ homolog subfamily A member 2 OS=Homo sapiens GN=DNAJA2 PE=1 SV=1 - [DNJA2_HUMAN]	59.22	1	21	21	136	28.54	27.96	27.96	26.82	26.77	27.10	3	3	28.70	28.03	28.02	26.57	26.98	26.84	28.25	26.80	0.005	0.007	-1.453
Q16629	Serine/arginine-rich splicing factor 7 OS=Homo sapiens GN=SRSF7 PE=1 SV=1 - [SRSF7_HUMAN]	34.45	1	8	8	28	22.26	24.05	23.30	25.97	25.88	25.93	3	3	22.42	24.12	23.36	25.72	26.10	25.67	23.30	25.83	0.008	0.009	2.530
Q9HD42	Charged multivesicular body protein 1a OS=Homo sapiens GN=CHMP1A PE=1 SV=1 - [CHM1A_HUMAN]	27.04	1	9	9	52	27.88	27.31	27.01	24.81	24.94	25.53	3	3	28.04	27.38	27.07	24.56	25.16	25.27	27.50	25.00	0.002	0.005	-2.500
O60888	Protein CutA OS=Homo sapiens GN=CUTA PE=1 SV=2 - [CUTA_HUMAN]	41.34	1	4	4	24	25.09	24.08	25.73	26.85	26.28	26.79	3	3	25.25	24.15	25.79	26.61	26.50	26.53	25.07	26.54	0.038	0.025	1.477
Q9BUF5	Tubulin beta-6 chain OS=Homo sapiens GN=TUBB6 PE=1 SV=1 - [TBB6_HUMAN]	79.82	1	11	24	219	29.07	28.57	28.72	29.66	29.35	29.88	3	3	29.24	28.64	28.78	29.42	29.57	29.62	28.88	29.54	0.027	0.020	0.650
Q14596	Next to BRCA1 gene 1 protein OS=Homo sapiens GN=NBR1 PE=1 SV=3 - [NBR1_HUMAN]	15.53	1	8	8	31	22.82	22.35	23.02	23.86	23.26	23.52	3	3	22.98	22.42	23.08	23.61	23.48	23.26	22.83	23.45	0.053	0.031	0.620
Q9H5V8	CUB domain-containing protein 1 OS=Homo sapiens GN=CDCP1 PE=1 SV=3 - [CDCP1_HUMAN]	29.07	1	24	24	121	24.91	25.08	24.63	27.31	26.24	26.89	3	3	25.07	25.15	24.69	27.07	26.46	26.63	24.97	26.72	0.002	0.004	1.747
Q13868	Exosome complex component RRP4 OS=Homo sapiens GN=EXOSC2 PE=1 SV=2 - [EXOS2_HUMAN]	14.33	1	2	2	8	21.11	21.71	21.73	22.76	22.52	22.87	3	3	21.28	21.78	21.79	22.51	22.73	22.61	21.61	22.62	0.005	0.007	1.003
Q9UM00	Transmembrane and coiled-coil domain-containing protein 1 OS=Homo sapiens GN=TMCO1 PE=1 SV=1 - [TMCO1_HUMAN]	17.02	1	3	3	12	21.77	22.46	23.18	23.66	23.41	24.41	3	3	21.93	22.52	23.24	23.41	23.63	24.15	22.57	23.73	0.056	0.032	1.164
Q12931	Heat shock protein 75 kDa, mitochondrial OS=Homo sapiens GN=TRAP1 PE=1 SV=3 - [TRAP1_HUMAN]	7.24	1	3	4	15	29.82	29.46	29.59	29.31	28.45	28.90	3	3	29.98	29.53	29.65	29.07	28.66	28.64	29.72	28.79	0.009	0.010	-0.926
Q13243	Serine/arginine-rich splicing factor 5 OS=Homo sapiens GN=SRSF5 PE=1 SV=1 - [SRSF5_HUMAN]	19.85	1	3	4	10		23.82	24.05	24.93	24.30	24.60	2	3		23.89	24.11	24.69	24.51	24.34	24.00	24.51	0.046	0.028	0.512
P35579	Myosin-9 OS=Homo sapiens GN=MYH9 PE=1 SV=4 - [MYH9_HUMAN]	56.58	3	96	111	741	25.88	26.14	26.24	26.93	26.85	27.70	3	3	26.04	26.21	26.29	26.68	27.06	27.44	26.18	27.06	0.019	0.016	0.881
P52292	Importin subunit alpha-1 OS=Homo sapiens GN=KPNA2 PE=1 SV=1 - [IMA1_HUMAN]	57.47	2	17	17	78	24.75	24.48	24.41	25.54	25.03	25.54	3	3	24.91	24.55	24.47	25.30	25.24	25.28	24.65	25.27	0.010	0.010	0.628
P49841	Glycogen synthase kinase-3 beta OS=Homo sapiens GN=GSK3B PE=1 SV=2 - [GSK3B_HUMAN]	61.19	1	14	22	100	27.76	27.87	27.92	24.92	23.52	25.74	3	3	27.92	27.94	27.98	24.67	23.74	25.48	27.95	24.63	0.003	0.005	-3.319
Q9UMS4	Pre-mRNA-processing factor 19 OS=Homo sapiens GN=PRPF19 PE=1 SV=1 - [PRP19_HUMAN]	37.90	13	11	12	41	22.72	22.83		25.24	25.69	25.72	2	3	22.88	22.90		24.99	25.91	25.46	22.89	25.45	0.005	0.007	2.559
Q7KZF4	Staphylococcal nuclease domain-containing protein 1 OS=Homo sapiens GN=SND1 PE=1 SV=1 - [SND1_HUMAN]	40.22	1	27	27	66	23.19	22.43	21.80	24.79	24.47	24.97	3	3	23.35	22.50	21.86	24.55	24.68	24.71	22.57	24.64	0.009	0.010	2.076
P62333	26S protease regulatory subunit 10B OS=Homo sapiens GN=PSMC6 PE=1 SV=1 - [PRS10_HUMAN]	58.10	1	21	21	86	25.16	24.06	24.63	26.15	26.17	26.32	3	3	25.33	24.13	24.69	25.90	26.39	26.06	24.71	26.12	0.020	0.016	1.402
P48556	26S proteasome non-ATPase regulatory subunit 8 OS=Homo sapiens GN=PSMD8 PE=1 SV=2 - [PSMD8_HUMAN]	32.57	1	7	7	31	23.44	23.58	23.22	24.91	24.50	25.18	3	3	23.60	23.65	23.28	24.67	24.72	24.92	23.51	24.77	0.001	0.003	1.259
Q9Y678	Coatomer subunit gamma-1 OS=Homo sapiens GN=COPG1 PE=1 SV=1 - [COPG1_HUMAN]	42.45	1	20	23	59	22.67	22.76	22.64	24.19	23.88	24.92	3	3	22.83	22.83	22.69	23.95	24.10	24.66	22.78	24.23	0.003	0.005	1.449
Q8WXE1	ATR-interacting protein OS=Homo sapiens GN=ATRIP PE=1 SV=1 - [ATRIP_HUMAN]	1.39	1	1	1	5	22.91	22.48		24.62	24.41	24.62	2	3	23.07	22.55		24.37	24.63	24.36	22.81	24.45	0.005	0.007	1.645
Q14376	UDP-glucose 4-epimerase OS=Homo sapiens GN=GALE PE=1 SV=2 - [GALE_HUMAN]	27.87	1	6	6	23	22.66	22.37	22.60	23.85	23.12	24.21	3	3	22.82	22.44	22.66	23.61	23.34	23.95	22.64	23.63	0.009	0.010	0.988
Q5VW32	BRO1 domain-containing protein BROX OS=Homo sapiens GN=BROX PE=1 SV=1 - [BROX_HUMAN]	54.01	1	15	15	84	27.89	28.19	28.05	25.67	24.25	25.88	3	3	28.05	28.26	28.11	25.42	24.47	25.62	28.14	25.17	0.001	0.003	-2.970
P60510	Serine/threonine-protein phosphatase 4 catalytic subunit OS=Homo sapiens GN=PPP4C PE=1 SV=1 - [PP4C_HUMAN]	9.12	1	1	2	9	23.82	24.30	24.37	25.12	24.90	25.41	3	3	23.98	24.37	24.43	24.87	25.11	25.15	24.26	25.05	0.009	0.010	0.788
P00387	NADH-cytochrome b5 reductase 3 OS=Homo sapiens GN=CYB5R3 PE=1 SV=3 - [NB5R3_HUMAN]	29.24	1	6	6	19	22.75	23.44	23.78	23.21	23.75	24.43	3	3	22.92	23.51	23.84	22.96	23.97	24.17	23.42	23.70	0.578	0.190	0.279
Q8IUE6	Histone H2A type 2-B OS=Homo sapiens GN=HIST2H2AB PE=1 SV=3 - [H2A2B_HUMAN]	33.85	1	2	5	34	29.98	30.52	29.94	32.47	31.68	31.78	3	3	30.14	30.59	30.00	32.22	31.89	31.52	30.24	31.88	0.004	0.006	1.634
P30153	Serine/threonine-protein phosphatase 2A 65 kDa regulatory subunit A alpha isoform OS=Homo sapiens GN=PPP2R1A PE=1 SV=4 - [2AAA_HUMAN]	52.46	1	18	23	114	24.52	24.27	24.29	25.37	25.36	25.99	3	3	24.69	24.34	24.34	25.12	25.57	25.73	24.46	25.48	0.009	0.010	1.019
Q16777	Histone H2A type 2-C OS=Homo sapiens GN=HIST2H2AC PE=1 SV=4 - [H2A2C_HUMAN]	36.43	10	2	6	88	31.48	31.83	31.30	33.80	33.16	32.55	3	3	31.64	31.90	31.36	33.56	33.38	32.29	31.63	33.08	0.027	0.020	1.444
Q8NC51	Plasminogen activator inhibitor 1 RNA-binding protein OS=Homo sapiens GN=SERBP1 PE=1 SV=2 - [PAIRB_HUMAN]	30.39	1	13	13	37	20.86	22.60	21.41	24.84	24.45	25.09	3	3	21.02	22.67	21.47	24.60	24.67	24.83	21.72	24.70	0.004	0.006	2.980
P83731	60S ribosomal protein L24 OS=Homo sapiens GN=RPL24 PE=1 SV=1 - [RL24_HUMAN]	31.85	1	6	6	20	24.22	24.98	24.95	27.30	26.50	26.92	3	3	24.38	25.05	25.00	27.05	26.72	26.66	24.81	26.81	0.001	0.004	1.999
P13861	cAMP-dependent protein kinase type II-alpha regulatory subunit OS=Homo sapiens GN=PRKAR2A PE=1 SV=2 - [KAP2_HUMAN]	31.93	1	8	8	27	22.49	22.76	23.17	23.00	23.87	24.22	3	3	22.65	22.83	23.23	22.76	24.09	23.96	22.90	23.60	0.200	0.083	0.701
P61604	10 kDa heat shock protein, mitochondrial OS=Homo sapiens GN=HSPE1 PE=1 SV=2 - [CH10_HUMAN]	60.78	1	7	7	27	23.33	24.60	25.13	26.18	26.10	26.80	3	3	23.49	24.67	25.19	25.94	26.32	26.54	24.45	26.26	0.027	0.020	1.816
P63244	Guanine nucleotide-binding protein subunit beta-2-like 1 OS=Homo sapiens GN=GNB2L1 PE=1 SV=3 - [GBLP_HUMAN]	72.87	1	20	20	116	25.59	25.17	25.49	26.35	26.71	27.12	3	3	25.75	25.24	25.55	26.10	26.93	26.86	25.52	26.63	0.021	0.017	1.115
Q9NYJ8	TGF-beta-activated kinase 1 and MAP3K7-binding protein 2 OS=Homo sapiens GN=TAB2 PE=1 SV=1 - [TAB2_HUMAN]	1.01	1	1	1	6	24.19	23.62	23.71	24.37	23.98	24.08	3	3	24.36	23.69	23.77	24.12	24.20	23.81	23.94	24.05	0.679	0.215	0.108
Q9NRN7	L-aminoadipate-semialdehyde dehydrogenase-phosphopantetheinyl transferase OS=Homo sapiens GN=AASDHPPT PE=1 SV=2 - [ADPPT_HUMAN]	12.94	1	3	3	10	22.65	22.68	22.68	23.75	23.18	23.49	3	3	22.81	22.75	22.73	23.51	23.40	23.23	22.76	23.38	0.002	0.004	0.613
P22460	Potassium voltage-gated channel subfamily A member 5 OS=Homo sapiens GN=KCNA5 PE=1 SV=4 - [KCNA5_HUMAN]	1.79	1	1	1	11	26.60	27.53	27.64	24.72	23.79	25.36	3	3	26.76	27.60	27.70	24.47	24.01	25.10	27.35	24.53	0.003	0.005	-2.828
Q8WV92	MIT domain-containing protein 1 OS=Homo sapiens GN=MITD1 PE=1 SV=1 - [MITD1_HUMAN]	62.25	1	13	13	83	26.95	27.62	27.47	25.59	24.69	25.42	3	3	27.11	27.69	27.53	25.34	24.90	25.16	27.44	25.14	0.000	0.002	-2.305
P50914	60S ribosomal protein L14 OS=Homo sapiens GN=RPL14 PE=1 SV=4 - [RL14_HUMAN]	31.16	1	10	10	54	25.89	25.91	26.90	28.40	28.04	28.02	3	3	26.06	25.98	26.95	28.16	28.26	27.76	26.33	28.06	0.008	0.009	1.727
Q9NVA2	Septin-11 OS=Homo sapiens GN=SEPT11 PE=1 SV=3 - [SEP11_HUMAN]	59.21	3	14	19	71	23.90	24.29	24.27	25.45	24.99	25.59	3	3	24.06	24.36	24.33	25.21	25.21	25.33	24.25	25.25	0.001	0.003	0.996
C9J7I0	UBAP1-MVB12-associated (UMA)-domain containing protein 1 OS=Homo sapiens GN=UMAD1 PE=4 SV=1 - [UMAD1_HUMAN]	29.41	1	2	2	7	23.17	22.99	23.48		21.61	22.27	3	2	23.33	23.06	23.54		21.82	22.01	23.31	21.92	0.005	0.007	-1.392
Q99714	3-hydroxyacyl-CoA dehydrogenase type-2 OS=Homo sapiens GN=HSD17B10 PE=1 SV=3 - [HCD2_HUMAN]	83.52	1	15	15	68	22.99	22.99	23.38	24.96	26.86	25.66	3	3	23.15	23.06	23.44	24.72	27.07	25.40	23.22	25.73	0.024	0.018	2.513
P31942	Heterogeneous nuclear ribonucleoprotein H3 OS=Homo sapiens GN=HNRNPH3 PE=1 SV=2 - [HNRH3_HUMAN]	25.72	3	7	8	22	23.14	22.72	23.11	24.28	25.26	25.06	3	3	23.31	22.79	23.17	24.04	25.47	24.80	23.09	24.77	0.019	0.016	1.684
P60842	Eukaryotic initiation factor 4A-I OS=Homo sapiens GN=EIF4A1 PE=1 SV=1 - [IF4A1_HUMAN]	81.03	1	14	28	202	26.87	26.46	26.57	27.58	27.92	28.17	3	3	27.03	26.53	26.63	27.34	28.13	27.91	26.73	27.79	0.019	0.016	1.062
Q9Y5S9	RNA-binding protein 8A OS=Homo sapiens GN=RBM8A PE=1 SV=1 - [RBM8A_HUMAN]	55.75	1	6	6	21	22.83	23.81	23.43	23.86	24.30	24.52	3	3	22.99	23.87	23.49	23.61	24.51	24.25	23.45	24.13	0.143	0.065	0.675
Q01081	Splicing factor U2AF 35 kDa subunit OS=Homo sapiens GN=U2AF1 PE=1 SV=3 - [U2AF1_HUMAN]	49.17	2	8	8	30	22.75	23.84	23.99	25.69	25.84	25.35	3	3	22.92	23.91	24.05	25.45	26.06	25.09	23.62	25.53	0.014	0.013	1.908
P61158	Actin-related protein 3 OS=Homo sapiens GN=ACTR3 PE=1 SV=3 - [ARP3_HUMAN]	62.20	3	19	19	87	25.11	24.95	24.93	26.05	25.74	26.51	3	3	25.27	25.02	24.99	25.80	25.96	26.25	25.09	26.00	0.005	0.007	0.910
P25788	Proteasome subunit alpha type-3 OS=Homo sapiens GN=PSMA3 PE=1 SV=2 - [PSA3_HUMAN]	49.41	1	12	12	58	22.95	23.81	23.53	25.40	24.91	25.40	3	3	23.11	23.88	23.59	25.15	25.12	25.14	23.53	25.14	0.002	0.004	1.610
Q9Y3I0	tRNA-splicing ligase RtcB homolog OS=Homo sapiens GN=RTCB PE=1 SV=1 - [RTCB_HUMAN]	36.04	1	14	14	56	23.58	23.43	23.03	24.47	24.01	24.31	3	3	23.74	23.49	23.09	24.22	24.22	24.05	23.44	24.17	0.021	0.017	0.723
P30566	Adenylosuccinate lyase OS=Homo sapiens GN=ADSL PE=1 SV=2 - [PUR8_HUMAN]	34.30	1	10	10	30	22.05	22.07	21.90	24.18	23.12	24.24	3	3	22.21	22.14	21.96	23.93	23.33	23.98	22.11	23.75	0.002	0.004	1.641
P62899	60S ribosomal protein L31 OS=Homo sapiens GN=RPL31 PE=1 SV=1 - [RL31_HUMAN]	40.80	1	5	5	18	24.17	25.31	24.54	26.51	26.34	26.45	3	3	24.33	25.38	24.60	26.26	26.56	26.18	24.77	26.33	0.010	0.010	1.564
P07108	Acyl-CoA-binding protein OS=Homo sapiens GN=DBI PE=1 SV=2 - [ACBP_HUMAN]	59.77	5	5	5	33	25.41	25.37	25.63	25.97	25.76	26.48	3	3	25.57	25.44	25.69	25.72	25.98	26.22	25.56	25.97	0.063	0.035	0.410
P28070	Proteasome subunit beta type-4 OS=Homo sapiens GN=PSMB4 PE=1 SV=4 - [PSB4_HUMAN]	54.92	1	9	9	40	24.57	24.31	24.55	25.00	25.22	25.50	3	3	24.74	24.38	24.61	24.76	25.43	25.24	24.57	25.14	0.067	0.037	0.567
P53396	ATP-citrate synthase OS=Homo sapiens GN=ACLY PE=1 SV=3 - [ACLY_HUMAN]	50.50	1	47	47	230	25.17	25.10	25.14	26.58	25.84	26.82	3	3	25.34	25.17	25.20	26.33	26.05	26.56	25.24	26.32	0.002	0.005	1.079
P33316	Deoxyuridine 5'-triphosphate nucleotidohydrolase, mitochondrial OS=Homo sapiens GN=DUT PE=1 SV=4 - [DUT_HUMAN]	19.44	1	4	4	13	21.63	22.28	22.54	23.71	23.31	23.77	3	3	21.79	22.34	22.60	23.46	23.52	23.51	22.25	23.50	0.006	0.008	1.254
Q16555	Dihydropyrimidinase-related protein 2 OS=Homo sapiens GN=DPYSL2 PE=1 SV=1 - [DPYL2_HUMAN]	58.74	2	18	21	94	24.29	24.09	24.08	25.13	24.94	25.66	3	3	24.45	24.16	24.14	24.89	25.16	25.40	24.25	25.15	0.007	0.009	0.897
P61923	Coatomer subunit zeta-1 OS=Homo sapiens GN=COPZ1 PE=1 SV=1 - [COPZ1_HUMAN]	7.91	1	1	1	5		22.64	23.00	23.99	23.90	24.46	2	3		22.71	23.06	23.74	24.12	24.20	22.89	24.02	0.015	0.013	1.132
P35244	Replication protein A 14 kDa subunit OS=Homo sapiens GN=RPA3 PE=1 SV=1 - [RFA3_HUMAN]	71.90	1	5	5	28	23.13	24.03	23.95	25.65	25.72	25.10	3	3	23.29	24.10	24.01	25.40	25.94	24.84	23.80	25.39	0.017	0.015	1.595

O75083	WD repeat-containing protein 1 OS=Homo sapiens GN=WDR1 PE=1 SV=4 - [WDR1_HUMAN]	65.51	1	25	25	141	25.71	25.67	25.32	27.04	26.63	27.38	3	3	25.87	25.74	25.38	26.80	26.84	27.12	25.66	26.92	0.002	0.004	1.257
Q6IBS0	Twinfilin-2 OS=Homo sapiens GN=TWF2 PE=1 SV=2 - [TWF2_HUMAN]	44.70	1	9	11	34	23.22	22.37		24.34	23.66	24.33	2	3	23.38	22.44		24.10	23.88	24.07	22.91	24.02	0.056	0.032	1.107
P40222	Alpha-taxilin OS=Homo sapiens GN=TXLNA PE=1 SV=3 - [TXLNA_HUMAN]	54.40	2	25	26	126	24.86	26.18	26.39	23.25	22.63	23.96	3	3	25.02	26.25	26.45	23.01	22.84	23.70	25.91	23.18	0.006	0.008	-2.727
O43765	Small glutamine-rich tetratricopeptide repeat-containing protein alpha OS=Homo sapiens GN=SGTA PE=1 SV=1 - [SGTA_HUMAN]	33.23	1	9	9	29	18.72	20.84	21.00	24.06	23.52	24.09	3	3	18.88	20.91	21.06	23.81	23.74	23.83	20.28	23.79	0.008	0.009	3.508
P78417	Glutathione S-transferase omega-1 OS=Homo sapiens GN=GSTO1 PE=1 SV=2 - [GSTO1_HUMAN]	49.38	1	14	14	45	23.87	23.99	24.08	25.36	24.72	25.77	3	3	24.03	24.06	24.14	25.12	24.94	25.51	24.08	25.19	0.003	0.005	1.111
Q9Y383	Putative RNA-binding protein Luc7-like 2 OS=Homo sapiens GN=LUC7L2 PE=1 SV=2 - [LC7L2_HUMAN]	29.08	1	9	11	31	21.70		22.03	24.08	23.72	24.08	2	3	21.86		22.08	23.83	23.94	23.82	21.97	23.86	0.000	0.002	1.891
Q9UL15	BAG family molecular chaperone regulator 5 OS=Homo sapiens GN=BAG5 PE=1 SV=1 - [BAG5_HUMAN]	48.10	1	15	15	48	24.34	24.07	24.15	23.30	22.00	22.98	3	3	24.50	24.14	24.21	23.05	22.22	22.72	24.28	22.66	0.004	0.006	-1.619
Q92616	Translational activator GCN1 OS=Homo sapiens GN=GCN1L1 PE=1 SV=6 - [GCN1L_HUMAN]	35.27	1	64	64	172	22.82	20.76	22.49	24.75	24.81	24.98	3	3	22.99	20.83	22.55	24.50	25.02	24.71	22.12	24.75	0.018	0.015	2.624
Q96AG4	Leucine-rich repeat-containing protein 59 OS=Homo sapiens GN=LRRC59 PE=1 SV=1 - [LRC59_HUMAN]	37.13	1	9	9	36	23.24	23.88	23.79	24.44	24.67	25.56	3	3	23.40	23.95	23.85	24.19	24.89	25.30	23.73	24.79	0.043	0.027	1.059
P61221	ATP-binding cassette sub-family E member 1 OS=Homo sapiens GN=ABCE1 PE=1 SV=1 - [ABCE1_HUMAN]	41.07	1	17	17	67	23.46	23.74	23.91	24.60	24.27	24.64	3	3	23.63	23.81	23.97	24.36	24.48	24.38	23.80	24.41	0.005	0.007	0.605
P34932	Heat shock 70 kDa protein 4 OS=Homo sapiens GN=HSPA4 PE=1 SV=4 - [HSP74_HUMAN]	54.05	1	37	39	185	24.84	25.09	24.72	26.17	25.99	26.71	3	3	25.00	25.16	24.78	25.93	26.21	26.45	24.98	26.20	0.003	0.005	1.214
P15170	Eukaryotic peptide chain release factor GTP-binding subunit ERF3A OS=Homo sapiens GN=GSPT1 PE=1 SV=1 - [ERF3A_HUMAN]	45.69	2	18	18	60	23.77	23.40	22.98	24.90	25.11	25.50	3	3	23.93	23.47	23.04	24.66	25.33	25.24	23.48	25.08	0.009	0.010	1.598
P30048	Thioredoxin-dependent peroxide reductase, mitochondrial OS=Homo sapiens GN=PRDX3 PE=1 SV=3 - [PRDX3_HUMAN]	33.98	1	5	5	21	21.90	23.73	23.11	23.33	24.35	24.42	3	3	22.06	23.80	23.17	23.09	24.56	24.16	23.01	23.94	0.241	0.095	0.927
Q16181	Septin-7 OS=Homo sapiens GN=SEPT7 PE=1 SV=2 - [SEPT7_HUMAN]	31.81	2	10	11	59	24.21	24.49	24.59	25.61	25.09	25.78	3	3	24.38	24.56	24.65	25.36	25.30	25.52	24.53	25.40	0.001	0.003	0.864
Q9NVZ3	Adaptin ear-binding coat-associated protein 2 OS=Homo sapiens GN=NECAP2 PE=1 SV=1 - [NECP2_HUMAN]	23.19	1	3	3	11	25.17	25.09	24.99	23.75	23.49	24.31	3	3	25.33	25.16	25.05	23.50	23.71	24.05	25.18	23.75	0.001	0.004	-1.424
Q9Y281	Cofilin-2 OS=Homo sapiens GN=CFL2 PE=1 SV=1 - [COF2_HUMAN]	65.66	1	5	11	52	27.64	27.89	27.69	28.83	28.56	29.27	3	3	27.80	27.96	27.75	28.59	28.78	29.01	27.84	28.79	0.002	0.005	0.955
Q13509	Tubulin beta-3 chain OS=Homo sapiens GN=TUBB3 PE=1 SV=2 - [TBB3_HUMAN]	66.67	1	7	23	273	29.59	29.11	29.18	30.12	29.94	30.38	3	3	29.76	29.18	29.24	29.87	30.15	30.12	29.39	30.05	0.032	0.022	0.654
P21589	5'-nucleotidase OS=Homo sapiens GN=NT5E PE=1 SV=1 - [5NTD_HUMAN]	57.14	2	27	27	235	29.57	29.35	29.19	28.43	27.28	28.15	3	3	29.73	29.42	29.25	28.18	27.50	27.89	29.47	27.86	0.003	0.005	-1.609
P30044	Peroxiredoxin-5, mitochondrial OS=Homo sapiens GN=PRDX5 PE=1 SV=4 - [PRDX5_HUMAN]	56.07	1	9	9	46	24.01	24.22	23.48	25.43	25.49	26.02	3	3	24.17	24.29	23.54	25.18	25.70	25.76	24.00	25.55	0.006	0.008	1.548
P51991	Heterogeneous nuclear ribonucleoprotein A3 OS=Homo sapiens GN=HNRNPA3 PE=1 SV=2 - [ROA3_HUMAN]	38.89	1	11	12	55	23.66	24.25	25.00	25.75	26.24	25.90	3	3	23.82	24.32	25.06	25.51	26.46	25.64	24.40	25.87	0.035	0.023	1.466
O00303	Eukaryotic translation initiation factor 3 subunit F OS=Homo sapiens GN=EIF3F PE=1 SV=1 - [EIF3F_HUMAN]	27.45	1	9	9	31	23.76	23.40	23.49	24.58	25.32	25.32	3	3	23.92	23.46	23.55	24.33	25.54	25.06	23.65	24.98	0.024	0.018	1.331
P61421	V-type proton ATPase subunit d 1 OS=Homo sapiens GN=ATP6V0D1 PE=1 SV=1 - [VA0D1_HUMAN]	12.82	1	3	3	9	22.06	21.26	21.98	23.31		23.06	3	2	22.22	21.33	22.04	23.07		22.80	21.86	22.93	0.062	0.035	1.070
Q9BQA1	Methylosome protein 50 OS=Homo sapiens GN=WDR77 PE=1 SV=1 - [MEP50_HUMAN]	28.95	1	8	8	27	23.12	22.58	22.81	25.88	23.91	24.40	3	3	23.28	22.65	22.87	25.64	24.13	24.14	22.93	24.63	0.033	0.023	1.703
P09497	Clathrin light chain B OS=Homo sapiens GN=CLTB PE=1 SV=1 - [CLCB_HUMAN]	24.02	1	6	6	32	23.32	24.69	24.60	25.87	25.86	26.16	3	3	23.48	24.76	24.66	25.62	26.07	25.90	24.30	25.87	0.022	0.017	1.563
P25398	40S ribosomal protein S12 OS=Homo sapiens GN=RPS12 PE=1 SV=3 - [RS12_HUMAN]	71.21	1	9	9	72	25.14	25.52	25.32	27.06	26.50	27.71	3	3	25.30	25.59	25.38	26.81	26.72	27.45	25.42	27.00	0.003	0.005	1.572
P62805	Histone H4 OS=Homo sapiens GN=HIST1H4A PE=1 SV=2 - [H4_HUMAN]	62.14	1	16	16	161	30.55	30.98	30.38	32.63	32.19	31.36	3	3	30.71	31.05	30.44	32.38	32.40	31.10	30.73	31.96	0.057	0.033	1.230
P09211	Glutathione S-transferase P OS=Homo sapiens GN=GSTP1 PE=1 SV=2 - [GSTP1_HUMAN]	42.38	1	7	7	31	23.29	23.79	22.70	25.64	25.13	25.90	3	3	23.45	23.86	22.76	25.39	25.34	25.64	23.36	25.46	0.003	0.006	2.101
Q14240	Eukaryotic initiation factor 4A-II OS=Homo sapiens GN=EIF4A2 PE=1 SV=2 - [IF4A2_HUMAN]	54.30	1	6	20	95	26.76	26.22	26.33	27.48	27.75	28.10	3	3	26.92	26.29	26.39	27.23	27.96	27.84	26.53	27.68	0.018	0.016	1.143
P55084	Trifunctional enzyme subunit beta, mitochondrial OS=Homo sapiens GN=HADHB PE=1 SV=3 - [ECHB_HUMAN]	23.63	1	9	9	14		21.96	21.49		24.26	24.18	2	2		22.03	21.55		24.47	23.92	21.79	24.20	0.022	0.017	2.410
Q16851	UTP--glucose-1-phosphate uridylyltransferase OS=Homo sapiens GN=UGP2 PE=1 SV=5 - [UGPA_HUMAN]	41.34	1	13	13	41	24.11	23.07	22.99	25.10	24.59	25.28	3	3	24.27	23.14	23.05	24.85	24.80	25.01	23.49	24.89	0.025	0.018	1.403
O60701	UDP-glucose 6-dehydrogenase OS=Homo sapiens GN=UGDH PE=1 SV=1 - [UGDH_HUMAN]	36.64	1	12	12	32	22.71	22.38	21.83	23.50	23.51	23.62	3	3	22.87	22.45	21.89	23.26	23.73	23.36	22.41	23.45	0.031	0.022	1.042
P23381	Tryptophan--tRNA ligase, cytoplasmic OS=Homo sapiens GN=WARS PE=1 SV=2 - [SYWC_HUMAN]	54.99	1	16	16	63	24.21	24.08	24.11	25.14	24.64	25.44	3	3	24.37	24.14	24.17	24.89	24.85	25.18	24.23	24.97	0.004	0.006	0.745
P50995	Annexin A11 OS=Homo sapiens GN=ANXA11 PE=1 SV=1 - [ANX11_HUMAN]	35.84	2	14	14	61	23.79	24.74	24.18	25.98	25.63	25.92	3	3	23.96	24.81	24.24	25.73	25.84	25.66	24.34	25.75	0.005	0.007	1.410
P13073	Cytochrome c oxidase subunit 4 isoform 1, mitochondrial OS=Homo sapiens GN=COX4I1 PE=1 SV=1 - [COX41_HUMAN]	25.44	1	5	5	12		22.40	22.78		24.13	24.81	2	2		22.47	22.84		24.34	24.55	22.66	24.45	0.014	0.013	1.792
P47914	60S ribosomal protein L29 OS=Homo sapiens GN=RPL29 PE=1 SV=2 - [RL29_HUMAN]	14.47	1	2	2	9	23.95		23.69	26.00	25.78	26.11	2	3	24.11		23.74	25.76	26.00	25.85	23.93	25.87	0.001	0.004	1.944
Q04837	Single-stranded DNA-binding protein, mitochondrial OS=Homo sapiens GN=SSBP1 PE=1 SV=1 - [SSBP_HUMAN]	54.05	1	7	7	44	23.01	23.87	23.83	25.97	26.01	25.05	3	3	23.17	23.94	23.89	25.72	26.23	24.79	23.67	25.58	0.018	0.015	1.911
A6NHL2	Tubulin alpha chain-like 3 OS=Homo sapiens GN=TUBAL3 PE=1 SV=2 - [TBAL3_HUMAN]	11.66	1	1	6	27	26.39	26.69	27.00	26.78	27.64	27.91	3	3	26.55	26.76	27.06	26.53	27.86	27.65	26.79	27.35	0.271	0.103	0.558
P14735	Insulin-degrading enzyme OS=Homo sapiens GN=IDE PE=1 SV=4 - [IDE_HUMAN]	4.22	1	3	3	11	20.58	20.82	20.77	22.78	21.72	22.84	3	3	20.74	20.89	20.83	22.53	21.94	22.58	20.82	22.35	0.002	0.004	1.528
Q9NQC3	Reticulon-4 OS=Homo sapiens GN=RTN4 PE=1 SV=2 - [RTN4_HUMAN]	7.89	1	4	4	12	24.02	23.67	23.67	25.66	24.34	25.22	3	3	24.18	23.74	23.73	25.41	24.56	24.95	23.88	24.98	0.019	0.016	1.094
O60443	Non-syndromic hearing impairment protein 5 OS=Homo sapiens GN=DFNA5 PE=1 SV=2 - [DFNA5_HUMAN]	5.04	1	2	2	8	21.41	21.62	21.46	22.41	22.18	22.83	3	3	21.57	21.69	21.52	22.17	22.40	22.57	21.59	22.38	0.003	0.006	0.785
P07437	Tubulin beta chain OS=Homo sapiens GN=TUBB PE=1 SV=2 - [TBB5_HUMAN]	82.21	2	4	27	451	29.95	29.48	29.84	30.48	30.55	31.19	3	3	30.11	29.55	29.90	30.24	30.76	30.93	29.85	30.65	0.041	0.026	0.792
P41250	Glycine--tRNA ligase OS=Homo sapiens GN=GARS PE=1 SV=3 - [SYG_HUMAN]	49.39	1	26	26	124	24.74	24.47	24.54	25.13	25.20	25.51	3	3	24.90	24.54	24.60	24.88	25.42	25.25	24.68	25.18	0.061	0.034	0.498
P10619	Lysosomal protective protein OS=Homo sapiens GN=CTSA PE=1 SV=2 - [PPGB_HUMAN]	7.50	1	2	2	9	22.34		22.48	23.12	22.92	23.14	2	3	22.50		22.54	22.87	23.14	22.88	22.52	22.96	0.031	0.022	0.440
P78344	Eukaryotic translation initiation factor 4 gamma 2 OS=Homo sapiens GN=EIF4G2 PE=1 SV=1 - [IF4G2_HUMAN]	15.55	1	9	9	26	21.89	22.01	22.41	22.51	23.56	23.63	3	3	22.05	22.07	22.47	22.26	23.77	23.37	22.20	23.13	0.118	0.056	0.935
Q9Y6M7	Sodium bicarbonate cotransporter 3 OS=Homo sapiens GN=SLC4A7 PE=1 SV=2 - [S4A7_HUMAN]	4.61	1	3	3	18	23.03	22.48	22.83	23.49	22.93	23.74	3	3	23.19	22.55	22.89	23.25	23.15	23.48	22.88	23.29	0.119	0.056	0.416
Q9Y5K6	CD2-associated protein OS=Homo sapiens GN=CD2AP PE=1 SV=1 - [CD2AP_HUMAN]	41.31	1	22	22	74	25.22	25.78	26.00	22.99	21.94	24.25	3	3	25.38	25.85	26.06	22.74	22.15	23.99	25.76	22.96	0.008	0.009	-2.801
Q96BW5	Phosphotriesterase-related protein OS=Homo sapiens GN=PTER PE=1 SV=1 - [PTER_HUMAN]	20.34	1	5	5	12	23.16	22.91		23.96	23.58	23.45	2	3	23.32	22.98		23.71	23.80	23.19	23.15	23.57	0.228	0.092	0.415
Q16186	Proteasomal ubiquitin receptor ADRM1 OS=Homo sapiens GN=ADRM1 PE=1 SV=2 - [ADRM1_HUMAN]	17.44	1	5	5	14	22.42		22.10	24.47	24.46	24.54	2	3	22.58		22.16	24.23	24.68	24.28	22.37	24.40	0.004	0.006	2.024
Q9NZN4	EH domain-containing protein 2 OS=Homo sapiens GN=EHD2 PE=1 SV=2 - [EHD2_HUMAN]	62.98	1	21	24	85	25.84	25.73	25.77	24.98	24.01	24.89	3	3	26.00	25.80	25.83	24.73	24.22	24.63	25.88	24.53	0.001	0.004	-1.351
P27824	Calnexin OS=Homo sapiens GN=CANX PE=1 SV=2 - [CALX_HUMAN]	50.34	1	25	25	145	24.96	26.18	26.17	26.85	26.93	27.91	3	3	25.12	26.25	26.23	26.60	27.14	27.65	25.87	27.13	0.058	0.033	1.262
P29401	Transketolase OS=Homo sapiens GN=TKT PE=1 SV=3 - [TKT_HUMAN]	61.80	2	28	28	145	23.99	25.28	24.95	26.21	26.16	26.81	3	3	24.15	25.35	25.01	25.96	26.37	26.55	24.84	26.29	0.022	0.017	1.457
Q15102	Platelet-activating factor acetylhydrolase IB subunit gamma OS=Homo sapiens GN=PAFAH1B3 PE=1 SV=1 - [PA1B3_HUMAN]	44.59	1	6	6	30	23.20	22.96	23.29	23.82	23.33	24.18	3	3	23.37	23.03	23.35	23.58	23.54	23.92	23.25	23.68	0.056	0.032	0.430
Q96FJ2	Dynein light chain 2, cytoplasmic OS=Homo sapiens GN=DYNLL2 PE=1 SV=1 - [DYL2_HUMAN]	58.43	1	2	5	50	27.07	28.26	28.49	25.65	25.09	26.65	3	3	27.23	28.33	28.55	25.40	25.31	26.39	28.04	25.70	0.012	0.012	-2.337
P56134	ATP synthase subunit f, mitochondrial OS=Homo sapiens GN=ATP5J2 PE=1 SV=3 - [ATPK_HUMAN]	25.53	1	2	2	6		22.50	23.20		23.39	23.96	2	2		22.57	23.26		23.61	23.70	22.92	23.65	0.170	0.074	0.738
Q9NUQ3	Gamma-taxilin OS=Homo sapiens GN=TXLNG PE=1 SV=2 - [TXLNG_HUMAN]	34.85	2	15	16	42	23.19	24.13	24.52	21.90	21.22	22.80	3	3	23.35	24.20	24.58	21.65	21.44	22.54	24.04	21.87	0.012	0.012	-2.168
O15371	Eukaryotic translation initiation factor 3 subunit D OS=Homo sapiens GN=EIF3D PE=1 SV=1 - [EIF3D_HUMAN]	36.86	1	14	14	42	22.61	22.84		24.52	24.44	24.66	2	3	22.77	22.91		24.27	24.66	24.40	22.84	24.44	0.002	0.004	1.602
O75116	Rho-associated protein kinase 2 OS=Homo sapiens GN=ROCK2 PE=1 SV=4 - [ROCK2_HUMAN]	7.28	1	6	8	11	20.87		20.44	24.63	20.88	23.60	2	3	21.03		20.50	24.39	21.09	23.34	20.76	22.94	0.185	0.079	2.177
P62495	Eukaryotic peptide chain release factor subunit 1 OS=Homo sapiens GN=ETF1 PE=1 SV=3 - [ERF1_HUMAN]	31.81	1	12	12	39	22.79	23.04	23.10	24.37	23.66	24.54	3	3	22.95	23.11	23.16	24.12	23.87	24.28	23.07	24.09	0.002	0.004	1.020
P61088	Ubiquitin-conjugating enzyme E2 N OS=Homo sapiens GN=UBE2N PE=1 SV=1 - [UBE2N_HUMAN]	65.13	2	7	7	56	26.71	26.30	26.36	27.40	26.58	27.01	3	3	26.87	26.37	26.42	27.16	26.79	26.75	26.56	26.90	0.169	0.074	0.343
P04156	Major prion protein OS=Homo sapiens GN=PRNP PE=1 SV=1 - [PRIO_HUMAN]	26.88	1	4	4	12	25.85	25.93	26.05	24.65	23.74	25.03	3	3	26.01	26.00	26.11	24.40	23.96	24.77	26.04	24.38	0.002	0.005	-1.662
P49588	Alanine--tRNA ligase, cytoplasmic OS=Homo sapiens GN=AARS PE=1 SV=2 - [SYAC_HUMAN]	36.67	1	25	25	80	23.96	23.13	23.25	25.11	24.35	25.61	3	3	24.12	23.20	23.31	24.87	24.57	25.35	23.54	24.93	0.020	0.016	1.383
Q13740	CD166 antigen OS=Homo sapiens GN=ALCAM PE=1 SV=2 - [CD166_HUMAN]	43.40	1	17	17	95	27.02	27.43	27.14	25.86	24.86	25.68	3	3	27.18	27.50	27.19	25.61	25.08	25.42	27.29	25.37	0.001	0.002	-1.922
Q16643	Drebrin OS=Homo sapiens GN=DBN1 PE=1 SV=4 - [DREB_HUMAN]	18.95	1	11	11	27	23.54	23.58	24.10	24.23	24.11	24.36	3	3	23.70	23.65	24.16	23.98	24.33	24.10	23.84	24.14	0.191	0.080	0.300
P68400	Casein kinase II subunit alpha OS=Homo sapiens GN=CSNK2A1 PE=1 SV=1 - [CSK21_HUMAN]	51.15	4	14	16	63	23.95	24.66	24.69	26.11	25.58	26.02	3	3	24.11	24.73	24.75	25.86	25.79	25.76	24.53	25.81	0.004	0.006	1.275
Q9H4B7	Tubulin beta-1 chain OS=Homo sapiens GN=TUBB1 PE=1 SV=1 - [TBB1_HUMAN]	20.18	1	5	9	70	28.38	28.05	28.18	29.04	28.66	29.46	3	3	28.54	28.12	28.24	28.79	28.88	29.20	28.30	28.96	0.020	0.016	0.658
O00487	26S proteasome non-ATPase regulatory subunit 14 OS=Homo sapiens GN=PSMD14 PE=1 SV=1 - [PSDE_HUMAN]	24.84	1	6	6	43	24.55	23.52	24.31	25.37	25.59	25.74	3	3	24.71	23.59	24.37	25.13	25.80	25.48	24.22	25.47	0.032	0.022	1.244
Q9Y224	UPF0568 protein C14orf166 OS=Homo sapiens GN=C14orf166 PE=1 SV=1 - [CN166_HUMAN]	46.72	2	10	10	41	24.17	24.15	24.13	23.70	23.72	23.95	3	3	24.33	24.22	24.19	23.45	23.94	23.69	24.25	23.69	0.020	0.016	-0.553
Q15843	NEDD8 OS=Homo sapiens GN=NEDD8 PE=1 SV=1 - [NEDD8_HUMAN]	41.98	1	3	3	23	23.35	23.98	24.30	25.77	25.16	25.52	3	3	23.52	24.04	24.36	25.52	25.38	25.26	23.97	25.39	0.005	0.007	1.413
P12270	Nucleoprotein TPR OS=Homo sapiens GN=TPR PE=1 SV=3 - [TPR_HUMAN]	4.66	1	10	10	19	24.94	23.97	24.58	23.00	23.17	22.48	3	3	25.10	24.04	24.63	22.76	23.38	22.22	24.59	22.79	0.017	0.015	-1.807
O60762	Dolichol-phosphate mannosyltransferase subunit 1 OS=Homo sapiens GN=DPM1 PE=1 SV=1 - [DPM1_HUMAN]	12.69	1	2	2	9	22.49	22.24	22.82	22.97	23.51	23.79	3	3	22.65	22.31	22.88	22.73	23.73	23.53	22.61	23.33	0.109	0.053	0.714
P30479	HLA class I histocompatibility antigen, B-41 alpha chain OS=Homo sapiens GN=HLA-B PE=1 SV=1 - [1B41_HUMAN]	55.25	14	3	16	87	25.17	25.49	25.45	27.51	26.39	27.67	3	3	25.33	25.56	25.51	27.27	26.61	27.41	25.47	27.10	0.003	0.005	1.630
Q13885	Tubulin beta-2A chain OS=Homo sapiens GN=TUBB2A PE=1 SV=1 - [TBB2A_HUMAN]	65.17	1	3	24	337	29.95	29.48	29.59	30.46	30.40	30.91	3	3	30.11	29.55	29.65	30.22	30.62	30.65	29.77	30.49	0.031	0.022	0.725
P49770	Translation initiation factor eIF-2B subunit beta OS=Homo sapiens GN=EIF2B2 PE=1 SV=3 - [EI2BB_HUMAN]	17.66	1	5	5	22	22.62	20.08	21.32	23.89	23.48	24.04	3	3	22.78	20.15	21.37	23.65	23.70	23.78	21.44	23.71	0.040	0.026	2.273
P07741	Adenine phosphoribosyltransferase OS=Homo sapiens GN=APRT PE=1 SV=2 - [APT_HUMAN]	67.22	1	11	11	51	24.92	24.63	24.25	25.51	25.72	25.79	3	3	25.08	24.70	24.31	25.27	25.93	25.53	24.70	25.58	0.041	0.026	0.880
Q96T51	RUN and FYVE domain-containing protein 1 OS=Homo sapiens GN=RUFY1 PE=1 SV=2 - [RUFY1_HUMAN]	8.19	1	4	4	9	20.01	18.81	19.01	22.15	23.00	21.03	3	3	20.17	18.88	19.07	21.90	23.22	20.77	19.37	21.96	0.034	0.023	2.589
P0C0S5	Histone H2A.Z OS=Homo sapiens GN=H2AFZ PE=1 SV=2 - [H2AZ_HUMAN]	31.25	2	3	5	58	30.40	30.84	30.25	32.90	32.01	31.55	3	3	30.56	30.91	30.31	32.66	32.23	31.29	30.60	32.06	0.029	0.021	1.464
O60716	Catenin delta-1 OS=Homo sapiens GN=CTNND1 PE=1 SV=1 - [CTND1_HUMAN]	22.83	1	18	18	57	23.89	23.46	23.51	23.53	22.71	23.14	3	3	24.06	23.53	23.57	23.28	22.93	22.88	23.72	23.03	0.031	0.022	-0.691
O43665	Regulator of G-protein signaling 10 OS=Homo sapiens GN=RGS10 PE=1 SV=2 - [RGS10_HUMAN]	6.94	1	1	1	6	21.16	21.43	20.94	22.43	22.09	22.69	3	3	21.32	21.50	21.00	22.19	22.31	22.43	21.27	22.31	0.003	0.005	1.037
Q9Y4F1	FERM, RhoGEF and pleckstrin domain-containing protein 1 OS=Homo sapiens GN=FARP1 PE=1 SV=1 - [FARP1_HUMAN]	7.85	1	4	4	13	23.53	23.36	23.55	21.88		22.19	3	2	23.69	23.43	23.61	21.63		21.93	23.58	21.78	0.001	0.004	-1.798
P18463	HLA class I histocompatibility antigen, B-37 alpha chain OS=Homo sapiens GN=HLA-B PE=1 SV=1 - [1B37_HUMAN]	35.91	5	0	10	51	25.09	25.49	25.45	27.51	26.39	27.67	3	3	25.25	25.56	25.51	27.27	26.61	27.41	25.44	27.10	0.003	0.006	1.657
P18621	60S ribosomal protein L17 OS=Homo sapiens GN=RPL17 PE=1 SV=3 - [RL17_HUMAN]	46.20	1	9	9	60	25.99	24.98	24.97	26.97	27.42	27.57	3	3	26.16	25.05	25.03	26.72	27.64	27.31	25.41	27.22	0.017	0.015	1.813
P07900	Heat shock protein HSP 90-alpha OS=Homo sapiens GN=HSP90AA1 PE=1 SV=5 - [HS90A_HUMAN]	66.94	3	27	47	389	29.41	29.21	29.43	29.97	29.46	30.11	3	3	29.57	29.28	29.49	29.73	29.68	29.85	29.45	29.75	0.038	0.025	0.306
Q13137	Calcium-binding and coiled-coil domain-containing protein 2 OS=Homo sapiens GN=CALCOCO2 PE=1 SV=1 - [CACO2_HUMAN]	43.72	1	15	15	50	23.73	23.36	22.98	24.69	23.77	24.10	3	3	23.89	23.43	23.04	24.45	23.98	23.84	23.46	24.09	0.107	0.052	0.635
Q08170	Serine/arginine-rich splicing factor 4 OS=Homo sapiens GN=SRSF4 PE=1 SV=2 - [SRSF4_HUMAN]	11.74	1	2	5	23	22.60	23.46	23.73	24.79	24.61	24.71	3	3	22.76	23.53	23.79	24.55	24.83	24.45	23.36	24.61	0.019	0.016	1.253
P23526	Adenosylhomocysteinase OS=Homo sapiens GN=AHCY PE=1 SV=4 - [SAHH_HUMAN]	55.09	1	19	20	96	25.03	24.89	24.49	26.92	25.78	26.94	3	3	25.20	24.96	24.55	26.67	26.00	26.68	24.90	26.45	0.006	0.008	1.551
P62140	Serine/threonine-protein phosphatase PP1-beta catalytic subunit OS=Homo sapiens GN=PPP1CB PE=1 SV=3 - [PP1B_HUMAN]	61.77	1	5	16	123	25.87	25.95	26.19	26.30	26.27	26.94	3	3	26.03	26.02	26.25	26.05	26.48	26.68	26.10	26.40	0.202	0.084	0.307
Q13751	Laminin subunit beta-3 OS=Homo sapiens GN=LAMB3 PE=1 SV=1 - [LAMB3_HUMAN]	1.79	1	1	2	7	28.39	28.53	28.51	25.57	23.92	21.30	3	3	28.55	28.59	28.57	25.33	24.14	21.04	28.57	23.50	0.017	0.015	-5.069
O15511	Actin-related protein 2/3 complex subunit 5 OS=Homo sapiens GN=ARPC5 PE=1 SV=3 - [ARPC5_HUMAN]	61.59	1	7	7	35	24.33	24.05	23.80	24.60	24.65	24.10	3	3	24.49	24.12	23.86	24.36	24.86	23.84	24.16	24.35	0.606	0.197	0.195
Q96CV9	Optineurin OS=Homo sapiens GN=OPTN PE=1 SV=2 - [OPTN_HUMAN]	16.81	1	8	8	22	21.28	21.23	20.69	22.26	21.59	21.58	3	3	21.44	21.30	20.75	22.01	21.81	21.32	21.16	21.71	0.137	0.063	0.549
Q07960	Rho GTPase-activating protein 1 OS=Homo sapiens GN=ARHGAP1 PE=1 SV=1 - [RHG01_HUMAN]	41.69	1	12	12	32	21.34	22.87	22.72	24.61	24.37	24.39	3	3	21.50	22.93	22.78	24.37	24.58	24.13	22.41	24.36	0.014	0.013	1.952
P38919	Eukaryotic initiation factor 4A-III OS=Homo sapiens GN=EIF4A3 PE=1 SV=4 - [IF4A3_HUMAN]	57.42	1	17	20	61	22.77	22.60	23.78	24.26	25.69	25.27	3	3	22.93	22.67	23.84	24.01	25.91	25.01	23.15	24.97	0.048	0.029	1.828
Q13561	Dynactin subunit 2 OS=Homo sapiens GN=DCTN2 PE=1 SV=4 - [DCTN2_HUMAN]	33.67	1	10	10	23	22.21	22.39	21.43	23.17	23.18	23.21	3	3	22.37	22.46	21.48	22.92	23.40	22.95	22.11	23.09	0.048	0.029	0.984
P25787	Proteasome subunit alpha type-2 OS=Homo sapiens GN=PSMA2 PE=1 SV=2 - [PSA2_HUMAN]	48.72	1	6	6	58	22.93	24.50	24.34	25.81	25.66	26.04	3	3	23.10	24.56	24.40	25.56	25.87	25.78	24.02	25.74	0.022	0.017	1.718
P41567	Eukaryotic translation initiation factor 1 OS=Homo sapiens GN=EIF1 PE=1 SV=1 - [EIF1_HUMAN]	53.10	2	5	5	27	24.53	24.41	24.78	24.31	23.83	24.21	3	3	24.69	24.48	24.84	24.07	24.05	23.95	24.67	24.02	0.004	0.006	-0.650
P45880	Voltage-dependent anion-selective channel protein 2 OS=Homo sapiens GN=VDAC2 PE=1 SV=2 - [VDAC2_HUMAN]	54.76	1	11	11	66	23.48	25.54	25.63	24.93	26.08	26.60	3	3	23.64	25.61	25.69	24.69	26.30	26.34	24.98	25.77	0.410	0.143	0.794
Q9ULC3	Ras-related protein Rab-23 OS=Homo sapiens GN=RAB23 PE=1 SV=1 - [RAB23_HUMAN]	40.93	1	8	8	34	24.83	24.59	24.53	23.74	22.83	23.85	3	3	24.99	24.66	24.59	23.49	23.05	23.59	24.75	23.38	0.003	0.005	-1.369
O75533	Splicing factor 3B subunit 1 OS=Homo sapiens GN=SF3B1 PE=1 SV=3 - [SF3B1_HUMAN]	14.26	1	12	12	27	22.74		21.68	24.61	23.52	23.98	2	3	22.90		21.74	24.37	23.74	23.72	22.32	23.94	0.051	0.030	1.621
Q9NPQ8	Synembryn-A OS=Homo sapiens GN=RIC8A PE=1 SV=3 - [RIC8A_HUMAN]	11.49	1	3	4	12	23.11	22.54		24.34	24.05	23.99	2	3	23.27	22.61		24.09	24.27	23.73	22.94	24.03	0.042	0.026	1.092
Q9Y262	Eukaryotic translation initiation factor 3 subunit L OS=Homo sapiens GN=EIF3L PE=1 SV=1 - [EIF3L_HUMAN]	33.16	1	14	14	41	22.80	22.58	22.46	24.04	23.34	24.43	3	3	22.96	22.65	22.52	23.79	23.55	24.17	22.71	23.84	0.007	0.009	1.127
P63208	S-phase kinase-associated protein 1 OS=Homo sapiens GN=SKP1 PE=1 SV=2 - [SKP1_HUMAN]	44.79	1	7	7	35	23.22	23.52	22.99	24.62	24.05	24.47	3	3	23.39	23.59	23.05	24.37	24.26	24.21	23.34	24.28	0.005	0.007	0.938
P07737	Profilin-1 OS=Homo sapiens GN=PFN1 PE=1 SV=2 - [PROF1_HUMAN]	80.71	1	5	9	93	29.52	29.45	29.33	30.41	29.82	30.42	3	3	29.68	29.51	29.39	30.17	30.04	30.15	29.53	30.12	0.003	0.006	0.591
P46783	40S ribosomal protein S10 OS=Homo sapiens GN=RPS10 PE=1 SV=1 - [RS10_HUMAN]	53.33	2	9	9	26	24.05	24.73	25.20	24.64	26.20	26.04	3	3	24.22	24.80	25.26	24.40	26.41	25.77	24.76	25.53	0.313	0.116	0.769
O60610	Protein diaphanous homolog 1 OS=Homo sapiens GN=DIAPH1 PE=1 SV=2 - [DIAP1_HUMAN]	14.78	1	12	12	29	22.10	22.74	22.07	23.45	23.44	24.02	3	3	22.26	22.81	22.13	23.21	23.65	23.76	22.40	23.54	0.013	0.012	1.139
Q13200	26S proteasome non-ATPase regulatory subunit 2 OS=Homo sapiens GN=PSMD2 PE=1 SV=3 - [PSMD2_HUMAN]	59.47	1	38	38	179	25.14	25.30	25.54	26.42	25.79	26.34	3	3	25.30	25.37	25.60	26.18	26.01	26.08	25.43	26.09	0.003	0.005	0.663
Q8NBI5	Solute carrier family 43 member 3 OS=Homo sapiens GN=SLC43A3 PE=1 SV=2 - [S43A3_HUMAN]	15.68	1	6	6	30	24.89	24.60	24.99	24.56	23.65	24.27	3	3	25.06	24.67	25.05	24.32	23.86	24.01	24.92	24.06	0.010	0.010	-0.860
Q92974	Rho guanine nucleotide exchange factor 2 OS=Homo sapiens GN=ARHGEF2 PE=1 SV=4 - [ARHG2_HUMAN]	3.55	1	2	2	5	21.53		21.67	22.18	20.81	22.20	2	3	21.69		21.73	21.93	21.02	21.94	21.71	21.63	0.855	0.252	-0.078
Q58FF6	Putative heat shock protein HSP 90-beta 4 OS=Homo sapiens GN=HSP90AB4P PE=5 SV=1 - [H90B4_HUMAN]	11.29	1	1	7	42	28.92	28.55	28.80	29.62	28.92	29.82	3	3	29.09	28.62	28.86	29.38	29.13	29.56	28.85	29.36	0.052	0.030	0.503
P14625	Endoplasmin OS=Homo sapiens GN=HSP90B1 PE=1 SV=1 - [ENPL_HUMAN]	57.41	2	45	46	280	25.44	26.96	27.24	28.49	28.88	28.38	3	3	25.60	27.03	27.30	28.24	29.10	28.12	26.64	28.49	0.039	0.025	1.843
P04080	Cystatin-B OS=Homo sapiens GN=CSTB PE=1 SV=2 - [CYTB_HUMAN]	76.53	1	5	5	26		24.68	25.13	25.76	25.42	26.06	2	3		24.74	25.19	25.51	25.64	25.80	24.97	25.65	0.041	0.026	0.682
P38159	RNA-binding motif protein, X chromosome OS=Homo sapiens GN=RBMX PE=1 SV=3 - [RBMX_HUMAN]	18.41	4	7	7	20		22.78	22.45	24.60	24.46	24.52	2	3		22.85	22.51	24.36	24.67	24.26	22.68	24.43	0.003	0.006	1.753
Q99653	Calcineurin B homologous protein 1 OS=Homo sapiens GN=CHP1 PE=1 SV=3 - [CHP1_HUMAN]	37.44	1	5	5	14	22.88	22.20	22.90	23.42	23.62	23.75	3	3	23.04	22.27	22.96	23.18	23.84	23.49	22.76	23.50	0.075	0.040	0.743
Q01105	Protein SET OS=Homo sapiens GN=SET PE=1 SV=3 - [SET_HUMAN]	36.55	2	6	6	36	22.95	24.98	25.19	25.55	26.08	26.54	3	3	23.11	25.05	25.25	25.30	26.29	26.28	24.47	25.96	0.121	0.057	1.491
Q06830	Peroxiredoxin-1 OS=Homo sapiens GN=PRDX1 PE=1 SV=1 - [PRDX1_HUMAN]	73.87	1	12	15	121	27.60	27.82	27.80	28.95	28.32	29.28	3	3	27.77	27.89	27.86	28.70	28.54	29.02	27.84	28.75	0.003	0.006	0.917
Q9NR45	Sialic acid synthase OS=Homo sapiens GN=NANS PE=1 SV=2 - [SIAS_HUMAN]	41.23	1	11	11	26	23.49	23.37		24.28	24.01	24.23	2	3	23.65	23.44		24.04	24.22	23.97	23.54	24.08	0.025	0.018	0.533
O95817	BAG family molecular chaperone regulator 3 OS=Homo sapiens GN=BAG3 PE=1 SV=3 - [BAG3_HUMAN]	8.17	1	4	4	9		21.55	21.52	23.18	23.08	23.43	2	3		21.62	21.58	22.94	23.30	23.17	21.60	23.13	0.002	0.004	1.537
P04083	Annexin A1 OS=Homo sapiens GN=ANXA1 PE=1 SV=2 - [ANXA1_HUMAN]	73.99	1	24	24	188	27.64	28.46	28.30	29.46	29.20	29.29	3	3	27.80	28.53	28.36	29.22	29.42	29.03	28.23	29.22	0.016	0.014	0.992
P69905	Hemoglobin subunit alpha OS=Homo sapiens GN=HBA1 PE=1 SV=2 - [HBA_HUMAN]	62.68	2	3	7	58	29.92	28.97	29.47	30.29	29.91	30.59	3	3	30.08	29.04	29.53	30.05	30.12	30.33	29.55	30.17	0.119	0.056	0.617
P46781	40S ribosomal protein S9 OS=Homo sapiens GN=RPS9 PE=1 SV=3 - [RS9_HUMAN]	45.88	1	15	15	59	24.46	24.16	24.27	30.31	28.85	26.24	3	3	24.62	24.23	24.33	30.06	29.06	25.97	24.39	28.37	0.032	0.022	3.975
P23528	Cofilin-1 OS=Homo sapiens GN=CFL1 PE=1 SV=3 - [COF1_HUMAN]	72.89	1	9	15	112	28.92	28.89	28.43	29.97	29.52	30.14	3	3	29.08	28.96	28.49	29.72	29.74	29.88	28.84	29.78	0.007	0.009	0.935
Q00610	Clathrin heavy chain 1 OS=Homo sapiens GN=CLTC PE=1 SV=5 - [CLH1_HUMAN]	73.37	1	80	100	749	27.67	27.50	27.23	29.24	28.10	29.05	3	3	27.83	27.57	27.29	29.00	28.32	28.79	27.56	28.70	0.011	0.011	1.139
O00622	Protein CYR61 OS=Homo sapiens GN=CYR61 PE=1 SV=1 - [CYR61_HUMAN]	62.73	1	20	20	122	26.89	26.10	26.70	29.35	28.33	27.51	3	3	27.05	26.16	26.76	29.10	28.55	27.25	26.66	28.30	0.054	0.032	1.641
P25685	DnaJ homolog subfamily B member 1 OS=Homo sapiens GN=DNAJB1 PE=1 SV=4 - [DNJB1_HUMAN]	38.53	1	12	13	54	24.10	24.53	24.39	24.59	24.69	24.75	3	3	24.26	24.60	24.44	24.35	24.91	24.48	24.43	24.58	0.494	0.168	0.147
P61326	Protein mago nashi homolog OS=Homo sapiens GN=MAGOH PE=1 SV=1 - [MGN_HUMAN]	31.51	2	4	4	13	22.52	22.70	22.68	23.64	23.07	23.61	3	3	22.68	22.77	22.74	23.40	23.28	23.35	22.73	23.34	0.000	0.002	0.614
P26641	Elongation factor 1-gamma OS=Homo sapiens GN=EEF1G PE=1 SV=3 - [EF1G_HUMAN]	69.11	1	25	25	124	26.03	25.72	25.97	27.01	26.33	27.62	3	3	26.20	25.79	26.03	26.77	26.55	27.36	26.01	26.89	0.030	0.022	0.885
Q15185	Prostaglandin E synthase 3 OS=Homo sapiens GN=PTGES3 PE=1 SV=1 - [TEBP_HUMAN]	42.50	1	6	6	38	25.92	25.60	25.43	26.43	26.36	26.77	3	3	26.09	25.67	25.49	26.18	26.58	26.51	25.75	26.42	0.034	0.023	0.676
Q9Y508	E3 ubiquitin-protein ligase RNF114 OS=Homo sapiens GN=RNF114 PE=1 SV=1 - [RN114_HUMAN]	15.35	1	3	3	7	21.56	21.87		20.14	20.09		2	2	21.72	21.94		19.89	20.30		21.83	20.10	0.018	0.015	-1.729

P68871	Hemoglobin subunit beta OS=Homo sapiens GN=HBB PE=1 SV=2 - [HBB_HUMAN]	78.91	2	8	9	26	29.49	28.38	29.30	29.81	29.37	30.14	3	3	29.65	28.45	29.36	29.56	29.58	29.88	29.15	29.68	0.236	0.094	0.523
Q14137	Ribosome biogenesis protein BOP1 OS=Homo sapiens GN=BOP1 PE=1 SV=2 - [BOP1_HUMAN]	13.94	1	6	6	17	22.68	22.60	22.99	24.86	23.84	23.82	3	3	22.84	22.67	23.05	24.61	24.05	23.56	22.85	24.07	0.020	0.016	1.220
Q96PU5	E3 ubiquitin-protein ligase NEDD4-like OS=Homo sapiens GN=NEDD4L PE=1 SV=2 - [NED4L_HUMAN]	29.33	1	16	20	63	24.91	24.34	24.17	24.39	22.86	23.25	3	3	25.07	24.41	24.23	24.15	23.08	22.99	24.57	23.40	0.061	0.034	-1.170
O43242	26S proteasome non-ATPase regulatory subunit 3 OS=Homo sapiens GN=PSMD3 PE=1 SV=2 - [PSMD3_HUMAN]	45.13	1	20	20	79	24.43	24.22	23.89	25.33	25.24	26.00	3	3	24.60	24.29	23.95	25.09	25.46	25.74	24.28	25.43	0.012	0.012	1.151
P60033	CD81 antigen OS=Homo sapiens GN=CD81 PE=1 SV=1 - [CD81_HUMAN]	25.00	1	3	3	42	29.91	30.06	30.31	28.46	26.87	28.89	3	3	30.07	30.12	30.36	28.21	27.09	28.62	30.19	27.98	0.009	0.010	-2.212
P35367	Histamine H1 receptor OS=Homo sapiens GN=HRH1 PE=1 SV=1 - [HRH1_HUMAN]	11.50	1	3	3	12	22.29	20.71	21.86	22.79	20.87	22.35	3	3	22.46	20.78	21.92	22.55	21.09	22.09	21.72	21.91	0.786	0.238	0.191
P61163	Alpha-centractin OS=Homo sapiens GN=ACTR1A PE=1 SV=1 - [ACTZ_HUMAN]	56.91	1	7	15	58	24.82	23.06	24.36	25.71	25.41	25.65	3	3	24.98	23.13	24.42	25.47	25.62	25.38	24.18	25.49	0.076	0.040	1.316
CON_007071011	SWISS-PROT:P12763 Bos taurus Alpha-2-HS-glycoprotein precursor	44.85	2	13	13	213	29.74	28.94	29.33	30.14	29.64	30.47	3	3	29.90	29.01	29.39	29.89	29.86	30.21	29.43	29.99	0.121	0.057	0.554
Q96PD2	Discoidin, CUB and LCCL domain-containing protein 2 OS=Homo sapiens GN=DCBLD2 PE=1 SV=1 - [DCBD2_HUMAN]	3.23	1	2	2	11	21.76	22.23	21.36	22.69		22.53	3	2	21.92	22.30	21.42	22.44		22.27	21.88	22.35	0.250	0.098	0.477
A8MWD9	Putative small nuclear ribonucleoprotein G-like protein 15 OS=Homo sapiens GN=SNRPGP15 PE=5 SV=2 - [RUXGL_HUMAN]	17.11	2	2	2	7	24.10	23.36	23.72	24.77	25.50	25.51	3	3	24.26	23.43	23.78	24.52	25.72	25.25	23.82	25.16	0.034	0.023	1.341
CON_008237951	SWISS-PROT:Q3MHN5 Bos taurus Vitamin D-binding protein precursor	39.24	2	13	13	70	25.92	24.62	24.73	26.42	25.40	26.10	3	3	26.08	24.69	24.78	26.18	25.62	25.84	25.18	25.88	0.220	0.090	0.694
Q9Y624	Junctional adhesion molecule A OS=Homo sapiens GN=F11R PE=1 SV=1 - [JAM1_HUMAN]	39.46	1	11	11	42	24.74	25.02	25.01	23.45	22.88	24.26	3	3	24.90	25.09	25.07	23.20	23.09	24.00	25.02	23.43	0.005	0.007	-1.590
Q96J02	E3 ubiquitin-protein ligase Itchy homolog OS=Homo sapiens GN=ITCH PE=1 SV=2 - [ITCH_HUMAN]	19.05	1	13	14	42	24.02	24.33	24.32	23.29	22.23	23.06	3	3	24.18	24.40	24.38	23.04	22.45	22.80	24.32	22.76	0.001	0.003	-1.559
P14868	Aspartate--tRNA ligase, cytoplasmic OS=Homo sapiens GN=DARS PE=1 SV=2 - [SYDC_HUMAN]	52.89	1	24	24	81	23.19	24.14	23.31	24.96	24.83	25.50	3	3	23.35	24.21	23.37	24.72	25.04	25.24	23.65	25.00	0.014	0.013	1.354
P49327	Fatty acid synthase OS=Homo sapiens GN=FASN PE=1 SV=3 - [FAS_HUMAN]	51.65	1	86	86	416	26.27	26.22	26.33	27.44	26.70	27.86	3	3	26.43	26.29	26.39	27.19	26.91	27.60	26.37	27.23	0.013	0.013	0.865
P45974	Ubiquitin carboxyl-terminal hydrolase 5 OS=Homo sapiens GN=USP5 PE=1 SV=2 - [UBP5_HUMAN]	19.70	1	10	10	38	23.53	21.91	23.07	24.31	24.36	24.80	3	3	23.69	21.98	23.13	24.07	24.58	24.54	22.93	24.40	0.051	0.030	1.463
P36871	Phosphoglucomutase-1 OS=Homo sapiens GN=PGM1 PE=1 SV=3 - [PGM1_HUMAN]	53.38	1	20	20	70	21.88	22.32	21.91	24.49	23.44	25.46	3	3	22.04	22.39	21.97	24.24	23.66	25.20	22.13	24.37	0.009	0.010	2.232
Q96CW1	AP-2 complex subunit mu OS=Homo sapiens GN=AP2M1 PE=1 SV=2 - [AP2M1_HUMAN]	50.34	1	18	18	69	26.09	25.96	26.24	25.14	24.30	25.64	3	3	26.25	26.03	26.30	24.89	24.51	25.38	26.20	24.93	0.009	0.010	-1.265
Q8TF42	Ubiquitin-associated and SH3 domain-containing protein B OS=Homo sapiens GN=UBASH3B PE=1 SV=2 - [UBS3B_HUMAN]	49.46	2	21	21	67	25.92	25.01	25.97	20.63	22.29	23.90	3	3	26.08	25.08	26.02	20.39	22.51	23.64	25.73	22.18	0.024	0.018	-3.552
P15529	Membrane cofactor protein OS=Homo sapiens GN=CD46 PE=1 SV=3 - [MCP_HUMAN]	12.76	1	6	6	29	24.53	25.62	24.76	23.22	22.19	22.93	3	3	24.69	25.69	24.82	22.98	22.40	22.67	25.07	22.68	0.003	0.005	-2.384
Q12765	Secernin-1 OS=Homo sapiens GN=SCRN1 PE=1 SV=2 - [SCRN1_HUMAN]	34.06	1	12	12	30	22.24	22.70	21.23	24.13	24.27	25.09	3	3	22.40	22.77	21.28	23.88	24.49	24.83	22.15	24.40	0.013	0.012	2.248
P49257	Protein ERGIC-53 OS=Homo sapiens GN=LMAN1 PE=1 SV=2 - [LMAN1_HUMAN]	22.94	1	7	7	25	22.38	23.04	22.70	24.04	23.66	24.72	3	3	22.54	23.11	22.76	23.79	23.87	24.46	22.81	24.04	0.010	0.010	1.236
Q9NR30	Nucleolar RNA helicase 2 OS=Homo sapiens GN=DDX21 PE=1 SV=5 - [DDX21_HUMAN]	37.16	1	22	24	60		22.53	23.13	25.47	25.46	25.43	2	3		22.60	23.19	25.23	25.68	25.17	22.90	25.36	0.004	0.006	2.461
Q99747	Gamma-soluble NSF attachment protein OS=Homo sapiens GN=NAPG PE=1 SV=1 - [SNAG_HUMAN]	28.21	1	8	8	27	22.36	22.64	22.31	23.98	23.15	23.88	3	3	22.53	22.71	22.37	23.74	23.37	23.62	22.53	23.58	0.002	0.005	1.042
P63010	AP-2 complex subunit beta OS=Homo sapiens GN=AP2B1 PE=1 SV=1 - [AP2B1_HUMAN]	55.39	1	25	38	186	26.08	25.90	26.11	24.79	23.47	25.20	3	3	26.24	25.97	26.17	24.54	23.69	24.94	26.13	24.39	0.010	0.010	-1.739
Q9BQL6	Fermitin family homolog 1 OS=Homo sapiens GN=FERMT1 PE=1 SV=1 - [FERM1_HUMAN]	4.28	1	1	2	9	24.07	23.96	25.13	23.54		24.72	3	2	24.23	24.03	25.19	23.30		24.46	24.48	23.88	0.412	0.143	-0.604
O15143	Actin-related protein 2/3 complex subunit 1B OS=Homo sapiens GN=ARPC1B PE=1 SV=3 - [ARC1B_HUMAN]	33.06	1	9	10	45	24.57	24.06	23.98	25.31	25.25	26.07	3	3	24.73	24.13	24.04	25.06	25.46	25.81	24.30	25.44	0.020	0.016	1.144
P43007	Neutral amino acid transporter A OS=Homo sapiens GN=SLC1A4 PE=1 SV=1 - [SATT_HUMAN]	10.53	1	4	4	15	23.75	24.26	24.39	22.91		23.73	3	2	23.92	24.33	24.45	22.66		23.47	24.23	23.07	0.050	0.030	-1.166
O94804	Serine/threonine-protein kinase 10 OS=Homo sapiens GN=STK10 PE=1 SV=1 - [STK10_HUMAN]	14.88	1	9	10	26	21.19	21.72	20.84	23.19	22.61	23.70	3	3	21.35	21.79	20.90	22.94	22.83	23.44	21.35	23.07	0.006	0.007	1.727
P21926	CD9 antigen OS=Homo sapiens GN=CD9 PE=1 SV=4 - [CD9_HUMAN]	23.25	1	6	6	44	28.61	29.06	29.19	27.13	25.52	27.54	3	3	28.77	29.13	29.25	26.88	25.73	27.28	29.05	26.63	0.008	0.009	-2.415
Q9UJ98	Cohesin subunit SA-3 OS=Homo sapiens GN=STAG3 PE=1 SV=2 - [STAG3_HUMAN]	0.57	1	1	1	4	28.40		28.63	25.24		26.16	2	2	28.56		28.68	24.99		25.90	28.62	25.44	0.020	0.016	-3.177
Q6P1N0	Coiled-coil and C2 domain-containing protein 1A OS=Homo sapiens GN=CC2D1A PE=1 SV=1 - [C2D1A_HUMAN]	29.97	1	21	21	67	24.33	25.07	25.03	22.95	21.46	23.19	3	3	24.49	25.14	25.09	22.71	21.68	22.93	24.91	22.44	0.005	0.007	-2.471
Q7Z2Z2	Elongation factor Tu GTP-binding domain-containing protein 1 OS=Homo sapiens GN=EFTUD1 PE=1 SV=2 - [ETUD1_HUMAN]	1.52	1	2	2	10	27.94	28.38	27.43	24.31	22.13	25.28	3	3	28.10	28.45	27.49	24.06	22.35	25.02	28.01	23.81	0.007	0.009	-4.203
Q14566	DNA replication licensing factor MCM6 OS=Homo sapiens GN=MCM6 PE=1 SV=1 - [MCM6_HUMAN]	11.81	1	8	8	20		19.32	20.03	21.86	22.87	22.80	2	3		19.39	20.09	21.62	23.09	22.53	19.74	22.41	0.022	0.017	2.673
P22695	Cytochrome b-c1 complex subunit 2, mitochondrial OS=Homo sapiens GN=UQCRC2 PE=1 SV=3 - [QCR2_HUMAN]	15.89	1	5	5	13	21.02	23.30	22.84	22.58	23.76	24.20	3	3	21.18	23.37	22.90	22.34	23.98	23.94	22.48	23.42	0.336	0.123	0.937
Q16698	2,4-dienoyl-CoA reductase, mitochondrial OS=Homo sapiens GN=DECR1 PE=1 SV=1 - [DECR_HUMAN]	10.45	1	2	2	8	21.74	21.76	21.42	22.58	22.45	23.16	3	3	21.90	21.83	21.48	22.34	22.67	22.90	21.74	22.64	0.013	0.012	0.898
P52209	6-phosphogluconate dehydrogenase, decarboxylating OS=Homo sapiens GN=PGD PE=1 SV=3 - [6PGD_HUMAN]	40.37	1	14	14	65	24.28	24.53	24.61	25.76	25.01	26.25	3	3	24.44	24.60	24.66	25.52	25.22	25.99	24.57	25.58	0.012	0.012	1.007
Q86VP1	Tax1-binding protein 1 OS=Homo sapiens GN=TAX1BP1 PE=1 SV=2 - [TAXB1_HUMAN]	37.39	1	24	24	83	23.22	23.61	22.50	26.15	24.47	25.13	3	3	23.38	23.68	22.56	25.91	24.68	24.87	23.20	25.15	0.018	0.016	1.950
Q08257	Quinone oxidoreductase OS=Homo sapiens GN=CRYZ PE=1 SV=1 - [QOR_HUMAN]	11.25	1	3	3	9	22.20	23.52		20.99	21.97	22.39	2	3	22.36	23.59		20.75	22.19	22.13	22.98	21.69	0.190	0.080	-1.288
P05026	Sodium/potassium-transporting ATPase subunit beta-1 OS=Homo sapiens GN=ATP1B1 PE=1 SV=1 - [AT1B1_HUMAN]	43.56	1	12	12	86	26.74	26.93	26.77	28.60	27.49	28.63	3	3	26.90	27.00	26.83	28.36	27.71	28.37	26.91	28.14	0.005	0.007	1.234
Q12974	Protein tyrosine phosphatase type IVA 2 OS=Homo sapiens GN=PTP4A2 PE=1 SV=1 - [TP4A2_HUMAN]	30.54	1	2	5	8	23.20	21.57	22.62	24.73		24.59	3	2	23.36	21.63	22.68	24.48		24.33	22.56	24.41	0.065	0.036	1.849
P20645	Cation-dependent mannose-6-phosphate receptor OS=Homo sapiens GN=M6PR PE=1 SV=1 - [MPRD_HUMAN]	35.38	1	7	7	43	23.18	24.02	23.50	26.34	24.96	25.71	3	3	23.34	24.09	23.55	26.10	25.18	25.45	23.66	25.58	0.006	0.007	1.916
O00541	Pescadillo homolog OS=Homo sapiens GN=PES1 PE=1 SV=1 - [PESC_HUMAN]	17.35	1	7	7	16	19.01	19.09		22.68	23.84	22.89	2	3	19.18	19.16		22.44	24.06	22.63	19.17	23.04	0.010	0.010	3.873
P14550	Alcohol dehydrogenase [NADP(+)] OS=Homo sapiens GN=AKR1A1 PE=1 SV=3 - [AK1A1_HUMAN]	27.38	1	5	6	11	22.86	22.27	22.67	23.49		23.48	3	2	23.02	22.34	22.73	23.24		23.22	22.70	23.23	0.127	0.059	0.536
O14980	Exportin-1 OS=Homo sapiens GN=XPO1 PE=1 SV=1 - [XPO1_HUMAN]	25.49	1	19	19	64	23.49	23.31	23.97	24.16	23.71	25.16	3	3	23.65	23.38	24.03	23.92	23.93	24.90	23.69	24.25	0.209	0.086	0.564
O95372	Acyl-protein thioesterase 2 OS=Homo sapiens GN=LYPLA2 PE=1 SV=1 - [LYPA2_HUMAN]	52.81	1	6	6	22	23.93	22.64	23.26	24.37	24.15	24.66	3	3	24.09	22.70	23.32	24.12	24.36	24.39	23.37	24.29	0.088	0.046	0.922
O14828	Secretory carrier-associated membrane protein 3 OS=Homo sapiens GN=SCAMP3 PE=1 SV=3 - [SCAM3_HUMAN]	31.12	1	7	7	34	23.47	22.51		26.42	24.78	25.50	2	3	23.63	22.58		26.17	25.00	25.24	23.10	25.47	0.030	0.021	2.367
Q13177	Serine/threonine-protein kinase PAK 2 OS=Homo sapiens GN=PAK2 PE=1 SV=3 - [PAK2_HUMAN]	29.58	1	8	10	31	23.83	24.12	24.01	24.64	24.35	24.60	3	3	23.99	24.19	24.07	24.39	24.57	24.34	24.08	24.44	0.016	0.015	0.351
P53992	Protein transport protein Sec24C OS=Homo sapiens GN=SEC24C PE=1 SV=3 - [SC24C_HUMAN]	11.24	1	8	8	21	21.87	21.87	22.22	23.78	22.42	23.92	3	3	22.03	21.94	22.28	23.54	22.63	23.66	22.08	23.28	0.024	0.018	1.194
Q92945	Far upstream element-binding protein 2 OS=Homo sapiens GN=KHSRP PE=1 SV=4 - [FUBP2_HUMAN]	28.41	1	14	14	34	19.35		20.52	24.40	24.42	24.40	2	3	19.51		20.58	24.16	24.64	24.14	20.04	24.31	0.002	0.005	4.268
P09104	Gamma-enolase OS=Homo sapiens GN=ENO2 PE=1 SV=3 - [ENOG_HUMAN]	30.41	1	5	9	83	26.26	26.84	26.74	28.08	27.36	28.31	3	3	26.42	26.91	26.80	27.83	27.57	28.05	26.71	27.82	0.005	0.007	1.110
P00441	Superoxide dismutase [Cu-Zn] OS=Homo sapiens GN=SOD1 PE=1 SV=2 - [SODC_HUMAN]	62.99	1	6	6	42	24.43	26.17	25.75	27.35	27.05	27.67	3	3	24.59	26.24	25.80	27.10	27.26	27.41	25.54	27.26	0.027	0.020	1.716
P55010	Eukaryotic translation initiation factor 5 OS=Homo sapiens GN=EIF5 PE=1 SV=2 - [IF5_HUMAN]	20.65	1	8	8	31	22.22	21.23	22.77	23.65	23.64	23.55	3	3	22.38	21.30	22.83	23.41	23.85	23.29	22.17	23.52	0.051	0.030	1.342
O00308	NEDD4-like E3 ubiquitin-protein ligase WWP2 OS=Homo sapiens GN=WWP2 PE=1 SV=2 - [WWP2_HUMAN]	10.46	2	7	8	25	23.42	23.66	23.67	21.85	20.99	22.81	3	3	23.58	23.73	23.73	21.60	21.21	22.54	23.68	21.79	0.009	0.010	-1.892
P62136	Serine/threonine-protein phosphatase PP1-alpha catalytic subunit OS=Homo sapiens GN=PPP1CA PE=1 SV=1 - [PP1A_HUMAN]	81.21	1	5	19	141	26.18	25.95	26.41	26.39	26.36	27.09	3	3	26.34	26.02	26.47	26.15	26.57	26.83	26.27	26.52	0.369	0.132	0.242
P36873	Serine/threonine-protein phosphatase PP1-gamma catalytic subunit OS=Homo sapiens GN=PPP1CC PE=1 SV=1 - [PP1G_HUMAN]	78.02	1	5	18	131	26.18	25.95	26.41	26.39	26.36	27.09	3	3	26.34	26.02	26.47	26.15	26.57	26.83	26.27	26.52	0.369	0.132	0.242
O95810	Serum deprivation-response protein OS=Homo sapiens GN=SDPR PE=1 SV=3 - [SDPR_HUMAN]	11.29	1	4	4	14	22.84	22.51	22.09	23.43	23.23	23.63	3	3	23.00	22.57	22.15	23.19	23.44	23.37	22.58	23.33	0.041	0.026	0.759
O15144	Actin-related protein 2/3 complex subunit 2 OS=Homo sapiens GN=ARPC2 PE=1 SV=1 - [ARPC2_HUMAN]	62.00	1	15	15	70	25.14	24.04	24.90	26.02	24.46	26.28	3	3	25.30	24.11	24.95	25.77	24.68	26.02	24.79	25.49	0.267	0.103	0.701
Q13867	Bleomycin hydrolase OS=Homo sapiens GN=BLMH PE=1 SV=1 - [BLMH_HUMAN]	28.57	1	8	8	30	22.71	23.54	23.22	24.64	24.10	25.14	3	3	22.87	23.61	23.28	24.40	24.31	24.88	23.26	24.53	0.010	0.011	1.274
Q92734	Protein TFG OS=Homo sapiens GN=TFG PE=1 SV=2 - [TFG_HUMAN]	24.25	4	7	7	40	25.10	25.10	25.01	25.61	25.30	25.72	3	3	25.27	25.17	25.06	25.36	25.52	25.46	25.17	25.45	0.019	0.016	0.280
P32119	Peroxiredoxin-2 OS=Homo sapiens GN=PRDX2 PE=1 SV=5 - [PRDX2_HUMAN]	65.15	1	10	11	70	26.24	26.67	26.36	27.68	27.15	28.09	3	3	26.41	26.74	26.42	27.44	27.36	27.83	26.52	27.54	0.005	0.007	1.019
P16152	Carbonyl reductase [NADPH] 1 OS=Homo sapiens GN=CBR1 PE=1 SV=3 - [CBR1_HUMAN]	47.65	2	9	9	38	24.03	24.15	23.75	25.18	24.51	25.11	3	3	24.19	24.22	23.81	24.93	24.73	24.85	24.07	24.84	0.006	0.008	0.761
O00244	Copper transport protein ATOX1 OS=Homo sapiens GN=ATOX1 PE=1 SV=1 - [ATOX1_HUMAN]	36.76	1	2	2	10	23.55	23.03	24.57	25.06	24.24	24.98	3	3	23.71	23.10	24.63	24.81	24.46	24.71	23.81	24.66	0.136	0.062	0.848
Q8WXA9	Splicing regulatory glutamine/lysine-rich protein 1 OS=Homo sapiens GN=SREK1 PE=1 SV=1 - [SREK1_HUMAN]	1.97	1	1	1	5	19.03	19.41		21.69	21.43	21.73	2	3	19.19	19.48		21.44	21.65	21.47	19.33	21.52	0.001	0.002	2.188
Q9NR31	GTP-binding protein SAR1a OS=Homo sapiens GN=SAR1A PE=1 SV=1 - [SAR1A_HUMAN]	65.15	1	6	10	65	25.01	24.78	24.49	25.77	25.22	25.84	3	3	25.17	24.85	24.55	25.52	25.43	25.58	24.86	25.51	0.024	0.018	0.652
Q8IYI6	Exocyst complex component 8 OS=Homo sapiens GN=EXOC8 PE=1 SV=2 - [EXOC8_HUMAN]	4.69	1	3	3	6	22.94	23.97	24.28		20.25	21.46	3	2	23.10	24.04	24.33		20.47	21.20	23.83	20.83	0.012	0.012	-2.990
P20042	Eukaryotic translation initiation factor 2 subunit 2 OS=Homo sapiens GN=EIF2S2 PE=1 SV=2 - [IF2B_HUMAN]	45.65	1	15	15	53		22.68	22.19	24.93	24.79	24.99	2	3		22.75	22.25	24.68	25.01	24.73	22.50	24.81	0.002	0.004	2.311
CON_Trypsin	SWISS-PROT:P00761 TRYP_PIG Trypsin - Sus scrofa Pig	41.99	1	9	9	108	33.03	32.52	32.82	33.10	32.79	33.05	3	3	33.19	32.59	32.88	32.86	33.00	32.79	32.89	32.88	0.994	0.281	-0.001
Q9Y6M5	Zinc transporter 1 OS=Homo sapiens GN=SLC30A1 PE=1 SV=3 - [ZNT1_HUMAN]	17.55	1	6	6	7	22.82		21.73	23.88		23.14	2	2	22.98		21.79	23.63		22.88	22.38	23.26	0.341	0.124	0.873
Q92504	Zinc transporter SLC39A7 OS=Homo sapiens GN=SLC39A7 PE=1 SV=2 - [S39A7_HUMAN]	8.32	1	2	2	5		22.69	21.39		23.17	22.03	2	2		22.76	21.45		23.39	21.77	22.11	22.58	0.693	0.217	0.475
P12004	Proliferating cell nuclear antigen OS=Homo sapiens GN=PCNA PE=1 SV=1 - [PCNA_HUMAN]	57.09	1	12	12	63	24.64	24.35	23.57	25.45	25.48	25.87	3	3	24.81	24.42	23.63	25.20	25.70	25.61	24.29	25.50	0.032	0.022	1.215
CON_007078841	SWISS-PROT:P17690 Bos taurus Beta-2-glycoprotein 1 precursor	44.35	2	10	10	58	24.71	25.45	25.61	25.62	25.69	26.37	3	3	24.88	25.52	25.67	25.38	25.90	26.11	25.36	25.80	0.248	0.097	0.442
O60814	Histone H2B type 1-K OS=Homo sapiens GN=HIST1H2BK PE=1 SV=3 - [H2B1K_HUMAN]	61.11	10	3	12	117	30.47	31.36	30.36	33.18	32.69	31.31	3	3	30.63	31.42	30.42	32.94	32.90	31.05	30.83	32.30	0.102	0.051	1.468
P46459	Vesicle-fusing ATPase OS=Homo sapiens GN=NSF PE=1 SV=3 - [NSF_HUMAN]	17.74	1	13	13	25	21.85	22.17	20.95	22.94	23.02	23.29	3	3	22.01	22.24	21.01	22.69	23.24	23.03	21.76	22.99	0.039	0.025	1.234
Q08209	Serine/threonine-protein phosphatase 2B catalytic subunit alpha isoform OS=Homo sapiens GN=PPP3CA PE=1 SV=1 - [PP2BA_HUMAN]	24.95	3	8	9	22	27.14	28.15	26.81	25.09	23.46	25.00	3	3	27.31	28.22	26.87	24.84	23.67	24.74	27.46	24.42	0.005	0.007	-3.046
P00492	Hypoxanthine-guanine phosphoribosyltransferase OS=Homo sapiens GN=HPRT1 PE=1 SV=2 - [HPRT_HUMAN]	59.17	2	10	10	50	24.24	24.59	24.03	25.63	25.07	25.84	3	3	24.41	24.66	24.08	25.38	25.28	25.57	24.38	25.41	0.005	0.007	1.028
Q92499	ATP-dependent RNA helicase DDX1 OS=Homo sapiens GN=DDX1 PE=1 SV=2 - [DDX1_HUMAN]	24.05	1	13	13	40	23.30	23.66	23.05	24.46	23.98	24.30	3	3	23.46	23.72	23.11	24.22	24.20	24.04	23.43	24.15	0.019	0.016	0.719
P63098	Calcineurin subunit B type 1 OS=Homo sapiens GN=PPP3R1 PE=1 SV=2 - [CANB1_HUMAN]	40.00	1	4	4	13		23.06	23.26	23.60	23.65	24.05	2	3		23.13	23.31	23.36	23.87	23.79	23.22	23.67	0.128	0.059	0.452
Q13409	Cytoplasmic dynein 1 intermediate chain 2 OS=Homo sapiens GN=DYNC1I2 PE=1 SV=3 - [DC1I2_HUMAN]	24.92	1	9	9	19	23.28	20.15	23.26	24.01	22.48	24.52	3	3	23.44	20.22	23.32	23.77	22.70	24.26	22.32	23.58	0.338	0.124	1.252
Q9UBF2	Coatomer subunit gamma-2 OS=Homo sapiens GN=COPG2 PE=1 SV=1 - [COPG2_HUMAN]	11.02	1	3	6	18	22.56	22.89	22.45	23.84	23.38	24.28	3	3	22.73	22.96	22.51	23.59	23.60	24.02	22.73	23.74	0.006	0.008	1.004
P63172	Dynein light chain Tctex-type 1 OS=Homo sapiens GN=DYNLT1 PE=1 SV=1 - [DYLT1_HUMAN]	38.94	1	3	3	13	22.81	21.75	21.84	23.24	22.98	23.49	3	3	22.97	21.81	21.90	22.99	23.20	23.23	22.23	23.14	0.074	0.040	0.912
P30466	HLA class I histocompatibility antigen, B-18 alpha chain OS=Homo sapiens GN=HLA-B PE=1 SV=1 - [1B18_HUMAN]	37.29	8	0	10	48	25.09	25.49	25.45	27.27	26.05	27.58	3	3	25.25	25.56	25.51	27.02	26.27	27.32	25.44	26.87	0.012	0.012	1.433
CON_006922051	SWISS-PROT:P06868 Bos taurus Plasminogen precursor	63.05	2	39	40	193	25.71	24.04	26.12	26.36	26.39	27.65	3	3	25.87	24.11	26.18	26.12	26.61	27.38	25.39	26.70	0.152	0.068	1.315
Q99623	Prohibitin-2 OS=Homo sapiens GN=PHB2 PE=1 SV=2 - [PHB2_HUMAN]	56.86	1	15	15	44		24.71	25.22	23.93	26.20	26.33	2	3		24.78	25.28	23.68	26.42	26.07	25.03	25.39	0.768	0.234	0.363
P68032	Actin, alpha cardiac muscle 1 OS=Homo sapiens GN=ACTC1 PE=1 SV=1 - [ACTC_HUMAN]	39.52	4	2	15	227	31.68	31.27	31.39	32.04	31.56	32.18	3	3	31.84	31.34	31.45	31.79	31.78	31.92	31.55	31.83	0.148	0.067	0.284
Q9NS69	Mitochondrial import receptor subunit TOM22 homolog OS=Homo sapiens GN=TOMM22 PE=1 SV=3 - [TOM22_HUMAN]	33.80	1	3	3	9		21.72	22.40	22.54	24.31	24.33	2	3		21.79	22.46	22.30	24.53	24.07	22.12	23.63	0.197	0.082	1.509
P06899	Histone H2B type 1-J OS=Homo sapiens GN=HIST1H2BJ PE=1 SV=3 - [H2B1J_HUMAN]	59.52	8	2	11	107	30.38	31.24	30.30	33.06	32.61	31.17	3	3	30.54	31.30	30.35	32.81	32.83	30.91	30.73	32.18	0.107	0.052	1.450
O15145	Actin-related protein 2/3 complex subunit 3 OS=Homo sapiens GN=ARPC3 PE=1 SV=3 - [ARPC3_HUMAN]	38.20	1	7	7	23	24.05	24.05	24.03	25.25	24.35	25.05	3	3	24.21	24.12	24.09	25.01	24.56	24.79	24.14	24.79	0.008	0.009	0.646
P00558	Phosphoglycerate kinase 1 OS=Homo sapiens GN=PGK1 PE=1 SV=3 - [PGK1_HUMAN]	74.82	2	26	26	203	27.25	27.39	27.06	28.42	27.75	28.52	3	3	27.41	27.45	27.12	28.18	27.97	28.26	27.33	28.14	0.004	0.006	0.806
P30046	D-dopachrome decarboxylase OS=Homo sapiens GN=DDT PE=1 SV=3 - [DOPD_HUMAN]	50.00	2	4	4	20	22.49	24.87	24.93	26.36	26.63	26.32	3	3	22.66	24.94	24.99	26.11	26.85	26.06	24.19	26.34	0.057	0.033	2.145
P24821	Tenascin OS=Homo sapiens GN=TNC PE=1 SV=3 - [TENA_HUMAN]	34.12	1	53	53	174	26.65	25.26	27.13	24.09	23.53	21.95	3	3	26.81	25.33	27.19	23.84	23.75	21.69	26.44	23.09	0.021	0.017	-3.351
Q9Y490	Talin-1 OS=Homo sapiens GN=TLN1 PE=1 SV=3 - [TLN1_HUMAN]	57.73	1	87	96	527	25.60	25.38	25.78	26.40	25.58	26.51	3	3	25.76	25.45	25.84	26.15	25.79	26.25	25.68	26.06	0.106	0.052	0.382
P30085	UMP-CMP kinase OS=Homo sapiens GN=CMPK1 PE=1 SV=3 - [KCY_HUMAN]	54.59	1	9	9	40	23.43	23.77	23.41	24.46	24.17	24.94	3	3	23.59	23.84	23.47	24.22	24.38	24.68	23.63	24.43	0.010	0.011	0.794
Q13443	Disintegrin and metalloproteinase domain-containing protein 9 OS=Homo sapiens GN=ADAM9 PE=1 SV=1 - [ADAM9_HUMAN]	25.89	1	15	15	43	23.77	24.65	24.43	22.80	21.73	22.99	3	3	23.93	24.72	24.49	22.55	21.94	22.73	24.38	22.41	0.004	0.006	-1.970
P67936	Tropomyosin alpha-4 chain OS=Homo sapiens GN=TPM4 PE=1 SV=3 - [TPM4_HUMAN]	58.06	2	12	20	128	25.30	26.63	26.66	27.39	27.32	27.71	3	3	25.46	26.70	26.72	27.15	27.53	27.45	26.29	27.38	0.067	0.037	1.082
Q9Y6G9	Cytoplasmic dynein 1 light intermediate chain 1 OS=Homo sapiens GN=DYNC1LI1 PE=1 SV=3 - [DC1L1_HUMAN]	26.58	1	10	11	32	23.44	22.65	22.35	24.05	24.07	24.61	3	3	23.60	22.72	22.41	23.81	24.29	24.35	22.91	24.15	0.035	0.024	1.236
P09525	Annexin A4 OS=Homo sapiens GN=ANXA4 PE=1 SV=4 - [ANXA4_HUMAN]	66.14	1	19	20	92	24.82	25.58	24.98	26.33	25.94	26.16	3	3	24.98	25.65	25.04	26.08	26.16	25.90	25.23	26.05	0.022	0.017	0.823
Q9Y4K0	Lysyl oxidase homolog 2 OS=Homo sapiens GN=LOXL2 PE=1 SV=1 - [LOXL2_HUMAN]	7.75	1	4	4	11	22.55	21.94	23.07	23.33	22.84	23.28	3	3	22.71	22.01	23.13	23.08	23.06	23.02	22.62	23.05	0.250	0.098	0.438
O43175	D-3-phosphoglycerate dehydrogenase OS=Homo sapiens GN=PHGDH PE=1 SV=4 - [SERA_HUMAN]	31.89	1	9	9	26	22.10	22.42	22.25	23.78	22.83	23.87	3	3	22.27	22.49	22.31	23.53	23.04	23.61	22.36	23.39	0.005	0.007	1.038
P61247	40S ribosomal protein S3a OS=Homo sapiens GN=RPS3A PE=1 SV=2 - [RS3A_HUMAN]	63.26	1	22	22	107	25.37	25.38	28.45	29.74	27.57	29.83	3	3	25.53	25.45	28.50	29.49	27.78	29.57	26.49	28.95	0.102	0.051	2.457
Q13772	Nuclear receptor coactivator 4 OS=Homo sapiens GN=NCOA4 PE=1 SV=1 - [NCOA4_HUMAN]	30.13	1	12	12	39	23.88	23.58	23.40	23.38	21.42	21.53	3	3	24.05	23.65	23.46	23.13	21.64	21.27	23.72	22.01	0.045	0.028	-1.708
P06753	Tropomyosin alpha-3 chain OS=Homo sapiens GN=TPM3 PE=1 SV=2 - [TPM3_HUMAN]	28.77	2	4	12	85	25.13	26.58	26.58	27.35	27.33	27.62	3	3	25.29	26.65	26.64	27.11	27.55	27.36	26.20	27.34	0.071	0.039	1.144
Q04323	UBX domain-containing protein 1 OS=Homo sapiens GN=UBXN1 PE=1 SV=2 - [UBXN1_HUMAN]	25.25	1	5	5	15		28.47	26.65	24.37	26.97	24.59	2	3		28.54	26.71	24.12	27.18	24.33	27.62	25.21	0.194	0.081	-2.411
P31930	Cytochrome b-c1 complex subunit 1, mitochondrial OS=Homo sapiens GN=UQCRC1 PE=1 SV=3 - [QCR1_HUMAN]	16.67	1	5	6	17		22.71	22.95		23.94	24.63	2	2		22.78	23.01		24.16	24.37	22.89	24.26	0.013	0.012	1.373
P49006	MARCKS-related protein OS=Homo sapiens GN=MARCKSL1 PE=1 SV=2 - [MRP_HUMAN]	56.41	1	6	6	38	26.39	26.93	26.94	27.89	27.28	28.36	3	3	26.55	26.99	27.00	27.64	27.49	28.10	26.85	27.75	0.019	0.016	0.898
P13693	Translationally-controlled tumor protein OS=Homo sapiens GN=TPT1 PE=1 SV=1 - [TCTP_HUMAN]	79.65	2	9	9	59	26.97	26.46	26.43	27.25	27.12	27.52	3	3	27.13	26.53	26.49	27.01	27.34	27.25	26.72	27.20	0.102	0.051	0.483
Q96CX2	BTB/POZ domain-containing protein KCTD12 OS=Homo sapiens GN=KCTD12 PE=1 SV=1 - [KCD12_HUMAN]	6.77	1	2	2	7	22.35	21.60	21.86	22.67	22.77	23.21	3	3	22.52	21.67	21.91	22.42	22.99	22.95	22.03	22.79	0.073	0.039	0.753
Q6XZF7	Dynamin-binding protein OS=Homo sapiens GN=DNMBP PE=1 SV=1 - [DNMBP_HUMAN]	5.20	1	6	6	27	22.56	22.37	24.95	21.99	20.20	22.46	3	3	22.72	22.44	25.01	21.74	20.42	22.20	23.39	21.45	0.117	0.056	-1.941
P61769	Beta-2-microglobulin OS=Homo sapiens GN=B2M PE=1 SV=1 - [B2MG_HUMAN]	40.34	1	4	4	45	25.66	27.48	27.45	28.76	28.40	29.09	3	3	25.82	27.55	27.51	28.51	28.61	28.83	26.96	28.65	0.043	0.027	1.690
O15027	Protein transport protein Sec16A OS=Homo sapiens GN=SEC16A PE=1 SV=3 - [SC16A_HUMAN]	2.20	1	3	3	12	21.41	20.44	20.64	21.93	21.56	22.57	3	3	21.57	20.51	20.70	21.69	21.78	22.31	20.93	21.93	0.058	0.033	1.000
P31431	Syndecan-4 OS=Homo sapiens GN=SDC4 PE=1 SV=2 - [SDC4_HUMAN]	22.73	1	4	4	22	24.93	25.04	24.90	24.32	22.88	23.71	3	3	25.09	25.11	24.96	24.07	23.10	23.45	25.05	23.54	0.006	0.008	-1.514
P09488	Glutathione S-transferase Mu 1 OS=Homo sapiens GN=GSTM1 PE=1 SV=3 - [GSTM1_HUMAN]	5.05	3	1	1	5	19.15	19.18		21.03	21.76	22.51	2	3	19.31	19.25		20.79	21.98	22.25	19.28	21.67	0.026	0.019	2.393
Q86SZ2	Trafficking protein particle complex subunit 6B OS=Homo sapiens GN=TRAPPC6B PE=1 SV=1 - [TPC6B_HUMAN]	12.03	1	1	1	14	21.63	21.45	21.76	22.04	21.69	22.56	3	3	21.79	21.52	21.82	21.79	21.90	22.30	21.71	22.00	0.192	0.081	0.284
P07339	Cathepsin D OS=Homo sapiens GN=CTSD PE=1 SV=1 - [CATD_HUMAN]	33.25	1	11	11	48	23.76	24.49	23.52	24.63	24.71	24.51	3	3	23.93	24.56	23.58	24.39	24.93	24.25	24.02	24.52	0.231	0.092	0.498
Q92973	Transportin-1 OS=Homo sapiens GN=TNPO1 PE=1 SV=2 - [TNPO1_HUMAN]	25.17	1	10	13	53	24.19	24.20	24.88	24.73	25.99	25.77	3	3	24.35	24.27	24.94	24.49	26.21	25.51	24.52	25.40	0.179	0.077	0.883
Q01469	Fatty acid-binding protein, epidermal OS=Homo sapiens GN=FABP5 PE=1 SV=3 - [FABP5_HUMAN]	67.41	2	7	7	38	24.22	24.91	24.74	25.51	25.19	25.75	3	3	24.38	24.98	24.80	25.26	25.41	25.49	24.72	25.39	0.024	0.018	0.665
P59998	Actin-related protein 2/3 complex subunit 4 OS=Homo sapiens GN=ARPC4 PE=1 SV=3 - [ARPC4_HUMAN]	49.40	1	7	7	32	24.75	24.62	24.15	25.56	25.15	25.85	3	3	24.91	24.69	24.21	25.31	25.37	25.59	24.60	25.42	0.021	0.017	0.819
O00762	Ubiquitin-conjugating enzyme E2 C OS=Homo sapiens GN=UBE2C PE=1 SV=1 - [UBE2C_HUMAN]	55.87	1	5	5	31	23.74	22.55	22.91	24.10	24.16	24.06	3	3	23.90	22.62	22.96	23.86	24.37	23.80	23.16	24.01	0.116	0.056	0.847
P51571	Translocon-associated protein subunit delta OS=Homo sapiens GN=SSR4 PE=1 SV=1 - [SSRD_HUMAN]	32.37	1	5	5	24		23.49	23.52	23.71	24.59	25.05	2	3		23.56	23.58	23.46	24.80	24.79	23.57	24.35	0.268	0.103	0.780
P35625	Metalloproteinase inhibitor 3 OS=Homo sapiens GN=TIMP3 PE=1 SV=2 - [TIMP3_HUMAN]	50.24	1	9	9	37	24.28	24.68	24.31	25.28	24.90	25.07	3	3	24.44	24.75	24.37	25.03	25.12	24.81	24.52	24.99	0.034	0.023	0.466
Q96FN4	Copine-2 OS=Homo sapiens GN=CPNE2 PE=1 SV=3 - [CPNE2_HUMAN]	15.51	4	5	6	16	23.47	22.73	22.68	23.69	23.53	23.68	3	3	23.63	22.80	22.74	23.44	23.75	23.42	23.06	23.54	0.195	0.082	0.477
Q13501	Sequestosome-1 OS=Homo sapiens GN=SQSTM1 PE=1 SV=1 - [SQSTM_HUMAN]	74.77	1	17	17	164	27.88	27.87	26.58	29.24	28.30	28.49	3	3	28.04	27.94	26.64	28.99	28.52	28.22	27.54	28.58	0.109	0.053	1.036
P08962	CD63 antigen OS=Homo sapiens GN=CD63 PE=1 SV=2 - [CD63_HUMAN]	21.85	2	5	5	29	27.12	27.39	27.67	26.69	25.47	25.68	3	3	27.28	27.46	27.72	26.44	25.69	25.42	27.49	25.85	0.008	0.009	-1.641
P16403	Histone H1.2 OS=Homo sapiens GN=HIST1H1C PE=1 SV=2 - [H12_HUMAN]	24.41	4	1	8	52	28.12	28.93	28.83	30.94	29.88	30.06	3	3	28.28	29.00	28.89	30.70	30.09	29.80	28.73	30.20	0.013	0.013	1.470
Q15008	26S proteasome non-ATPase regulatory subunit 6 OS=Homo sapiens GN=PSMD6 PE=1 SV=1 - [PSMD6_HUMAN]	42.16	1	14	14	54	22.94	23.09	23.84	25.26	23.71	25.12	3	3	23.10	23.16	23.90	25.02	23.93	24.86	23.39	24.60	0.046	0.029	1.213
Q9BY32	Inosine triphosphate pyrophosphatase OS=Homo sapiens GN=ITPA PE=1 SV=2 - [ITPA_HUMAN]	36.60	1	5	5	15	22.01	21.38		23.67	23.69	23.57	2	3	22.18	21.44		23.42	23.90	23.31	21.81	23.54	0.017	0.015	1.732

P21980	Protein-glutamine gamma-glutamyltransferase 2 OS=Homo sapiens GN=TGM2 PE=1 SV=2 - [TGM2_HUMAN]	54.44	1	27	28	168	25.68	25.51	25.56	26.32	25.80	26.69	3	3	25.84	25.58	25.62	26.07	26.01	26.43	25.68	26.17	0.032	0.022	0.496
Q14847	LIM and SH3 domain protein 1 OS=Homo sapiens GN=LASP1 PE=1 SV=2 - [LASP1_HUMAN]	53.64	1	12	12	51	23.74	24.37	24.16	25.64	24.84	25.31	3	3	23.90	24.44	24.22	25.40	25.05	25.05	24.19	25.17	0.007	0.009	0.979
Q8N357	Solute carrier family 35 member F6 OS=Homo sapiens GN=SLC35F6 PE=1 SV=1 - [S35F6_HUMAN]	3.23	1	1	1	6	19.96	20.27	19.83	22.19	20.75	21.42	3	3	20.13	20.34	19.89	21.94	20.97	21.16	20.12	21.36	0.019	0.016	1.238
P61916	Epididymal secretory protein E1 OS=Homo sapiens GN=NPC2 PE=1 SV=1 - [NPC2_HUMAN]	43.05	1	4	4	12		23.08	22.34	23.08	23.85	23.42	2	3		23.15	22.40	22.83	24.06	23.15	22.77	23.35	0.375	0.133	0.575
O00154	Cytosolic acyl coenzyme A thioester hydrolase OS=Homo sapiens GN=ACOT7 PE=1 SV=3 - [BACH_HUMAN]	25.26	1	7	7	19	23.00	22.76	22.05	23.60	24.48	24.68	3	3	23.16	22.83	22.11	23.35	24.69	24.42	22.70	24.15	0.047	0.029	1.455
P16435	NADPH--cytochrome P450 reductase OS=Homo sapiens GN=POR PE=1 SV=2 - [NCPR_HUMAN]	5.17	1	2	2	11	21.16	21.21	21.09	21.71	21.68	22.44	3	3	21.33	21.28	21.15	21.46	21.90	22.18	21.25	21.85	0.051	0.030	0.595
Q9NWU2	Glucose-induced degradation protein 8 homolog OS=Homo sapiens GN=GID8 PE=1 SV=1 - [GID8_HUMAN]	35.96	1	4	4	15	22.27	21.85		23.73	23.91	24.00	2	3	22.43	21.92		23.49	24.13	23.74	22.18	23.78	0.014	0.013	1.605
P00568	Adenylate kinase isoenzyme 1 OS=Homo sapiens GN=AK1 PE=1 SV=3 - [KAD1_HUMAN]	45.36	1	6	6	20	23.18	21.94	21.51	23.55	23.31	23.33	3	3	23.34	22.01	21.57	23.31	23.53	23.07	22.30	23.30	0.143	0.065	0.996
P15374	Ubiquitin carboxyl-terminal hydrolase isozyme L3 OS=Homo sapiens GN=UCHL3 PE=1 SV=1 - [UCHL3_HUMAN]	45.22	1	8	8	33	23.85	23.57	23.01	24.57	24.09	24.65	3	3	24.02	23.64	23.07	24.33	24.30	24.39	23.57	24.34	0.050	0.030	0.765
Q06033	Inter-alpha-trypsin inhibitor heavy chain H3 OS=Homo sapiens GN=ITIH3 PE=1 SV=2 - [ITIH3_HUMAN]	3.93	1	3	3	14	24.02	23.14	23.47	24.41	24.20	25.17	3	3	24.18	23.21	23.53	24.17	24.42	24.91	23.64	24.50	0.076	0.040	0.855
P61081	NEDD8-conjugating enzyme Ubc12 OS=Homo sapiens GN=UBE2M PE=1 SV=1 - [UBC12_HUMAN]	45.36	1	8	8	35	23.70	23.60	23.33	24.80	23.85	24.62	3	3	23.86	23.67	23.39	24.55	24.07	24.36	23.64	24.33	0.025	0.019	0.690
O14964	Hepatocyte growth factor-regulated tyrosine kinase substrate OS=Homo sapiens GN=HGS PE=1 SV=1 - [HGS_HUMAN]	25.61	1	18	18	74	23.95	23.80	23.24	24.84	24.04	24.42	3	3	24.11	23.87	23.30	24.60	24.26	24.16	23.76	24.34	0.104	0.051	0.579
Q9H8Y8	Golgi reassembly-stacking protein 2 OS=Homo sapiens GN=GORASP2 PE=1 SV=3 - [GORS2_HUMAN]	10.84	1	3	3	15	22.53	22.55	21.47	23.08	23.05	22.88	3	3	22.69	22.62	21.53	22.83	23.26	22.62	22.28	22.91	0.209	0.086	0.628
P36542	ATP synthase subunit gamma, mitochondrial OS=Homo sapiens GN=ATP5C1 PE=1 SV=1 - [ATPG_HUMAN]	26.51	1	6	6	19		21.75	22.29	22.67	23.97	23.85	2	3		21.82	22.35	22.43	24.19	23.59	22.08	23.40	0.154	0.069	1.321
Q14542	Equilibrative nucleoside transporter 2 OS=Homo sapiens GN=SLC29A2 PE=1 SV=3 - [S29A2_HUMAN]	2.63	1	1	1	5	23.57	23.66	23.82	22.25		21.94	3	2	23.74	23.73	23.88	22.00		21.68	23.78	21.84	0.001	0.003	-1.944
P10412	Histone H1.4 OS=Homo sapiens GN=HIST1H1E PE=1 SV=2 - [H14_HUMAN]	23.74	4	1	8	46	28.09	28.93	28.81	30.94	29.82	30.06	3	3	28.25	29.00	28.86	30.70	30.03	29.80	28.71	30.18	0.014	0.013	1.469
Q15785	Mitochondrial import receptor subunit TOM34 OS=Homo sapiens GN=TOMM34 PE=1 SV=2 - [TOM34_HUMAN]	42.72	1	11	11	50	24.48	24.48	24.38	24.87	24.57	24.60	3	3	24.64	24.55	24.44	24.62	24.79	24.34	24.54	24.58	0.789	0.238	0.041
Q96IX5	Up-regulated during skeletal muscle growth protein 5 OS=Homo sapiens GN=USMG5 PE=1 SV=1 - [USMG5_HUMAN]	25.86	1	1	1	5		22.61	23.51	21.92	23.36	24.58	2	3		22.68	23.57	21.67	23.57	24.32	23.12	23.19	0.957	0.273	0.063
Q9BQG0	Myb-binding protein 1A OS=Homo sapiens GN=MYBBP1A PE=1 SV=2 - [MBB1A_HUMAN]	11.14	1	10	10	29		21.10	21.45	23.17	23.74	23.29	2	3		21.17	21.51	22.92	23.95	23.03	21.34	23.30	0.021	0.017	1.963
Q9NZL9	Methionine adenosyltransferase 2 subunit beta OS=Homo sapiens GN=MAT2B PE=1 SV=1 - [MAT2B_HUMAN]	20.06	1	5	5	16		22.45	22.44	24.02	23.74	24.28	2	3		22.51	22.50	23.78	23.95	24.02	22.51	23.92	0.001	0.003	1.411
Q9NUU7	ATP-dependent RNA helicase DDX19A OS=Homo sapiens GN=DDX19A PE=1 SV=1 - [DD19A_HUMAN]	12.76	2	4	4	16	21.87	21.47	20.97	22.71	22.53	23.55	3	3	22.03	21.54	21.03	22.47	22.74	23.29	21.53	22.83	0.026	0.019	1.300
Q92522	Histone H1x OS=Homo sapiens GN=H1FX PE=1 SV=1 - [H1X_HUMAN]	17.84	1	4	4	12		20.28	20.40	23.37	23.30	22.50	2	3		20.35	20.46	23.12	23.51	22.24	20.41	22.96	0.014	0.013	2.550
P40261	Nicotinamide N-methyltransferase OS=Homo sapiens GN=NNMT PE=1 SV=1 - [NNMT_HUMAN]	26.89	1	4	4	18	22.49	22.56	22.93	23.26	22.88	24.28	3	3	22.65	22.63	22.99	23.02	23.09	24.02	22.76	23.38	0.147	0.066	0.618
P53350	Serine/threonine-protein kinase PLK1 OS=Homo sapiens GN=PLK1 PE=1 SV=1 - [PLK1_HUMAN]	23.88	1	9	9	31	24.65	24.29	24.83	23.46		23.12	3	2	24.81	24.36	24.89	23.22		22.86	24.69	23.04	0.007	0.009	-1.648
P46940	Ras GTPase-activating-like protein IQGAP1 OS=Homo sapiens GN=IQGAP1 PE=1 SV=1 - [IQGA1_HUMAN]	66.39	2	78	82	428	27.69	27.47	27.16	26.68	26.00	26.87	3	3	27.85	27.54	27.22	26.44	26.21	26.60	27.54	26.42	0.006	0.008	-1.121
P12235	ADP/ATP translocase 1 OS=Homo sapiens GN=SLC25A4 PE=1 SV=4 - [ADT1_HUMAN]	36.91	2	1	9	41		25.31	25.57	25.88	26.72	27.61	2	3		25.38	25.63	25.63	26.94	27.35	25.50	26.64	0.190	0.080	1.139
P33176	Kinesin-1 heavy chain OS=Homo sapiens GN=KIF5B PE=1 SV=1 - [KINH_HUMAN]	49.12	3	40	40	154	23.61	23.43	23.45	24.75	24.34	26.28	3	3	23.77	23.50	23.51	24.50	24.56	26.02	23.59	25.03	0.047	0.029	1.435
P37173	TGF-beta receptor type-2 OS=Homo sapiens GN=TGFBR2 PE=1 SV=2 - [TGFR2_HUMAN]	15.52	1	7	7	13	22.03	21.96	21.70	23.41		22.65	3	2	22.19	22.03	21.76	23.16		22.39	21.99	22.78	0.098	0.049	0.784
Q9Y3C6	Peptidyl-prolyl cis-trans isomerase-like 1 OS=Homo sapiens GN=PPIL1 PE=1 SV=1 - [PPIL1_HUMAN]	28.92	1	4	4	15	22.93	23.21	23.13	23.94	23.40	24.13	3	3	23.09	23.28	23.19	23.69	23.61	23.87	23.19	23.73	0.005	0.007	0.540
P13667	Protein disulfide-isomerase A4 OS=Homo sapiens GN=PDIA4 PE=1 SV=2 - [PDIA4_HUMAN]	48.84	1	28	29	88		22.91	24.01	25.10	26.05	26.18	2	3		22.98	24.07	24.86	26.27	25.92	23.53	25.68	0.051	0.030	2.156
P11766	Alcohol dehydrogenase class-3 OS=Homo sapiens GN=ADH5 PE=1 SV=4 - [ADHX_HUMAN]	46.79	1	10	10	41	23.74	23.71	23.66	24.50	24.12	25.23	3	3	23.90	23.77	23.72	24.25	24.34	24.97	23.80	24.52	0.036	0.024	0.722
Q92747	Actin-related protein 2/3 complex subunit 1A OS=Homo sapiens GN=ARPC1A PE=1 SV=2 - [ARC1A_HUMAN]	40.54	1	7	8	35	24.26	23.61	23.60	24.69	24.70	25.52	3	3	24.42	23.68	23.66	24.45	24.92	25.26	23.92	24.87	0.051	0.030	0.951
Q86VN1	Vacuolar protein-sorting-associated protein 36 OS=Homo sapiens GN=VPS36 PE=1 SV=1 - [VPS36_HUMAN]	56.99	1	18	18	47	25.11	24.64	24.92		20.54	22.12	3	2	25.27	24.70	24.98		20.76	21.86	24.99	21.31	0.004	0.006	-3.680
Q9H223	EH domain-containing protein 4 OS=Homo sapiens GN=EHD4 PE=1 SV=1 - [EHD4_HUMAN]	51.20	2	17	24	110	26.18	26.03	25.59	25.52	24.72	25.27	3	3	26.34	26.10	25.65	25.28	24.93	25.00	26.03	25.07	0.013	0.013	-0.960
Q96RT1	Protein LAP2 OS=Homo sapiens GN=ERBB2IP PE=1 SV=2 - [LAP2_HUMAN]	24.15	1	25	25	80	24.53	24.26	24.24	23.88	22.49	23.64	3	3	24.69	24.33	24.30	23.63	22.70	23.38	24.44	23.24	0.017	0.015	-1.203
Q16881	Thioredoxin reductase 1, cytoplasmic OS=Homo sapiens GN=TXNRD1 PE=1 SV=3 - [TRXR1_HUMAN]	28.20	2	12	12	53	23.35	24.11	23.58	24.60	24.34	24.95	3	3	23.51	24.18	23.64	24.36	24.55	24.69	23.78	24.54	0.028	0.021	0.759
P35637	RNA-binding protein FUS OS=Homo sapiens GN=FUS PE=1 SV=1 - [FUS_HUMAN]	13.31	1	3	4	19	23.15	23.45	23.73	23.86	23.60	24.61	3	3	23.31	23.52	23.79	23.61	23.82	24.35	23.54	23.93	0.211	0.087	0.388
Q96FZ7	Charged multivesicular body protein 6 OS=Homo sapiens GN=CHMP6 PE=1 SV=3 - [CHMP6_HUMAN]	43.28	1	7	7	30	24.61	24.54	24.70	24.11	22.70	23.87	3	3	24.77	24.61	24.76	23.87	22.92	23.60	24.71	23.46	0.012	0.012	-1.250
Q04760	Lactoylglutathione lyase OS=Homo sapiens GN=GLO1 PE=1 SV=4 - [LGUL_HUMAN]	57.07	1	8	8	38	23.55	24.53	24.39	25.41	24.85	25.74	3	3	23.72	24.60	24.45	25.17	25.07	25.48	24.26	25.24	0.031	0.022	0.981
P60709	Actin, cytoplasmic 1 OS=Homo sapiens GN=ACTB PE=1 SV=1 - [ACTB_HUMAN]	80.00	6	1	28	689	31.77	31.58	31.42	32.29	31.79	32.39	3	3	31.93	31.64	31.47	32.04	32.01	32.13	31.68	32.06	0.050	0.030	0.378
P63261	Actin, cytoplasmic 2 OS=Homo sapiens GN=ACTG1 PE=1 SV=1 - [ACTG_HUMAN]	80.00	6	1	28	689	31.77	31.58	31.42	32.29	31.79	32.39	3	3	31.93	31.64	31.47	32.04	32.01	32.13	31.68	32.06	0.050	0.030	0.378
Q9UHD8	Septin-9 OS=Homo sapiens GN=SEPT9 PE=1 SV=2 - [SEPT9_HUMAN]	21.84	1	10	10	28		22.33	22.34	23.95	23.94	24.72	2	3		22.40	22.40	23.71	24.16	24.46	22.40	24.11	0.009	0.010	1.710
Q969T9	WW domain-binding protein 2 OS=Homo sapiens GN=WBP2 PE=1 SV=1 - [WBP2_HUMAN]	15.33	1	4	4	10	22.63	22.53	21.95	23.95	22.97	24.15	3	3	22.79	22.60	22.00	23.71	23.18	23.89	22.46	23.59	0.024	0.018	1.129
O43493	Trans-Golgi network integral membrane protein 2 OS=Homo sapiens GN=TGOLN2 PE=1 SV=2 - [TGON2_HUMAN]	12.71	1	4	4	25	21.37	21.92	20.85	22.39	22.29	22.64	3	3	21.53	21.99	20.91	22.14	22.51	22.38	21.48	22.34	0.058	0.033	0.866
P06733	Alpha-enolase OS=Homo sapiens GN=ENO1 PE=1 SV=2 - [ENOA_HUMAN]	76.27	2	25	29	289	28.01	28.90	28.83	29.86	29.38	31.53	3	3	28.17	28.97	28.89	29.61	29.59	31.27	28.67	30.16	0.072	0.039	1.487
P19525	Interferon-induced, double-stranded RNA-activated protein kinase OS=Homo sapiens GN=EIF2AK2 PE=1 SV=2 - [E2AK2_HUMAN]	9.44	1	4	4	13	22.37	21.90		23.61	23.74	23.89	2	3	22.54	21.97		23.37	23.95	23.63	22.25	23.65	0.019	0.016	1.400
P49755	Transmembrane emp24 domain-containing protein 10 OS=Homo sapiens GN=TMED10 PE=1 SV=2 - [TMEDA_HUMAN]	47.95	1	9	9	54	24.92	24.84	24.41	25.39	25.32	25.78	3	3	25.08	24.91	24.47	25.14	25.53	25.52	24.82	25.40	0.060	0.034	0.576
Q96AC1	Fermitin family homolog 2 OS=Homo sapiens GN=FERMT2 PE=1 SV=1 - [FERM2_HUMAN]	49.85	1	24	25	133	26.30	26.05	26.09	25.45	24.52	25.77	3	3	26.46	26.12	26.15	25.20	24.74	25.51	26.24	25.15	0.012	0.012	-1.095
P14406	Cytochrome c oxidase subunit 7A2, mitochondrial OS=Homo sapiens GN=COX7A2 PE=1 SV=1 - [CX7A2_HUMAN]	27.71	1	2	2	7		23.69	24.23	23.78	24.57	24.83	2	3		23.76	24.29	23.53	24.79	24.56	24.03	24.30	0.654	0.209	0.267
P12429	Annexin A3 OS=Homo sapiens GN=ANXA3 PE=1 SV=3 - [ANXA3_HUMAN]	59.75	1	19	19	64	23.20	24.22	23.98	25.01	24.56	25.13	3	3	23.36	24.29	24.04	24.77	24.78	24.87	23.90	24.80	0.032	0.022	0.904
Q5T0F9	Coiled-coil and C2 domain-containing protein 1B OS=Homo sapiens GN=CC2D1B PE=1 SV=1 - [C2D1B_HUMAN]	22.49	1	12	12	27	23.54	24.08	24.25	22.06		22.04	3	2	23.71	24.15	24.31	21.82		21.78	24.06	21.80	0.002	0.005	-2.257
O94907	Dickkopf-related protein 1 OS=Homo sapiens GN=DKK1 PE=1 SV=1 - [DKK1_HUMAN]	12.03	1	3	3	8	21.79	22.34	21.66	22.77	22.52	22.28	3	3	21.95	22.41	21.72	22.52	22.74	22.02	22.03	22.43	0.244	0.096	0.400
O00264	Membrane-associated progesterone receptor component 1 OS=Homo sapiens GN=PGRMC1 PE=1 SV=3 - [PGRC1_HUMAN]	59.49	1	6	7	31	24.08	25.07	24.74	24.43	25.52	25.90	3	3	24.24	25.14	24.80	24.18	25.74	25.64	24.73	25.19	0.461	0.159	0.463
P52565	Rho GDP-dissociation inhibitor 1 OS=Homo sapiens GN=ARHGDIA PE=1 SV=3 - [GDIR1_HUMAN]	61.76	1	8	8	47	25.83	26.47	26.38	27.32	26.72	27.49	3	3	25.99	26.54	26.44	27.07	26.93	27.23	26.32	27.08	0.017	0.015	0.755
P43034	Platelet-activating factor acetylhydrolase IB subunit alpha OS=Homo sapiens GN=PAFAH1B1 PE=1 SV=2 - [LIS1_HUMAN]	28.29	1	8	9	44	23.25	23.66	23.61	24.49	23.87	24.97	3	3	23.41	23.73	23.67	24.24	24.09	24.71	23.61	24.35	0.024	0.018	0.741
Q6IAA8	Ragulator complex protein LAMTOR1 OS=Homo sapiens GN=LAMTOR1 PE=1 SV=2 - [LTOR1_HUMAN]	49.07	1	4	4	22	24.67	23.83		25.46	25.01	24.72	2	3	24.83	23.90		25.21	25.22	24.46	24.36	24.97	0.296	0.111	0.602
P13987	CD59 glycoprotein OS=Homo sapiens GN=CD59 PE=1 SV=1 - [CD59_HUMAN]	25.78	1	5	5	40	29.00	29.03	29.11	28.06	26.80	28.49	3	3	29.16	29.10	29.17	27.82	27.02	28.23	29.14	27.69	0.015	0.014	-1.455
P35754	Glutaredoxin-1 OS=Homo sapiens GN=GLRX PE=1 SV=2 - [GLRX1_HUMAN]	70.75	1	4	4	19	22.81	23.37	22.69	24.24	23.61	24.24	3	3	22.97	23.44	22.75	24.00	23.83	23.98	23.05	23.93	0.014	0.013	0.881
P10644	cAMP-dependent protein kinase type I-alpha regulatory subunit OS=Homo sapiens GN=PRKAR1A PE=1 SV=1 - [KAP0_HUMAN]	37.01	2	12	12	57	24.81	24.39	23.01	25.14	25.09	24.44	3	3	24.97	24.46	23.07	24.90	25.31	24.18	24.17	24.79	0.394	0.139	0.627
P42025	Beta-centractin OS=Homo sapiens GN=ACTR1B PE=1 SV=1 - [ACTY_HUMAN]	34.31	1	1	9	24	23.33	23.38	22.70	24.35	23.84	24.71	3	3	23.49	23.45	22.76	24.11	24.05	24.44	23.23	24.20	0.023	0.018	0.966
O96008	Mitochondrial import receptor subunit TOM40 homolog OS=Homo sapiens GN=TOMM40 PE=1 SV=1 - [TOM40_HUMAN]	52.91	1	11	11	30	20.78	22.77	24.05		24.71	25.34	3	2	20.94	22.84	24.11		24.93	25.08	22.63	25.00	0.140	0.064	2.374
CON_006954891	SWISS-PROT:P34955 Bos taurus Alpha-1-antiproteinase precursor	50.72	1	21	22	211	29.44	27.94	28.16	29.59	28.95	29.15	3	3	29.60	28.00	28.22	29.35	29.17	28.89	28.61	29.14	0.365	0.131	0.529
P09622	Dihydrolipoyl dehydrogenase, mitochondrial OS=Homo sapiens GN=DLD PE=1 SV=2 - [DLDH_HUMAN]	31.43	1	12	12	21		22.68	21.85	23.28	24.27	24.24	2	3		22.75	21.90	23.04	24.49	23.98	22.33	23.83	0.097	0.049	1.506
Q96CS7	Pleckstrin homology domain-containing family B member 2 OS=Homo sapiens GN=PLEKHB2 PE=1 SV=1 - [PKHB2_HUMAN]	33.78	1	6	6	31	24.96	24.75	24.16	24.30	23.65	23.95	3	3	25.13	24.82	24.21	24.06	23.87	23.69	24.72	23.87	0.042	0.027	-0.847
Q14195	Dihydropyrimidinase-related protein 3 OS=Homo sapiens GN=DPYSL3 PE=1 SV=1 - [DPYL3_HUMAN]	8.60	1	1	4	16	23.79	24.12	24.60	23.40	23.47	24.47	3	3	23.95	24.18	24.65	23.16	23.69	24.21	24.26	23.69	0.192	0.081	-0.577
Q9HBH0	Rho-related GTP-binding protein RhoF OS=Homo sapiens GN=RHOF PE=1 SV=1 - [RHOF_HUMAN]	37.44	1	5	5	16	23.04	23.55	23.63	22.36		22.61	3	2	23.20	23.61	23.69	22.11		22.35	23.50	22.23	0.010	0.010	-1.269
P40616	ADP-ribosylation factor-like protein 1 OS=Homo sapiens GN=ARL1 PE=1 SV=1 - [ARL1_HUMAN]	41.99	1	5	5	22	22.60	22.83	23.29	24.16	23.14	24.18	3	3	22.76	22.90	23.35	23.92	23.35	23.92	23.00	23.73	0.049	0.029	0.725
P50552	Vasodilator-stimulated phosphoprotein OS=Homo sapiens GN=VASP PE=1 SV=3 - [VASP_HUMAN]	48.68	1	15	15	67	24.06	23.82	24.05	24.35	23.91	24.54	3	3	24.23	23.89	24.11	24.10	24.12	24.28	24.07	24.17	0.453	0.156	0.095
P22392	Nucleoside diphosphate kinase B OS=Homo sapiens GN=NME2 PE=1 SV=1 - [NDKB_HUMAN]	76.32	1	1	10	101	26.99	27.93	27.77	28.45	28.16	28.81	3	3	27.16	28.00	27.83	28.21	28.38	28.55	27.66	28.38	0.061	0.034	0.716
P07954	Fumarate hydratase, mitochondrial OS=Homo sapiens GN=FH PE=1 SV=3 - [FUMH_HUMAN]	39.02	1	14	14	48		23.64	23.35	24.35	24.87	25.21	2	3		23.71	23.41	24.10	25.08	24.95	23.56	24.71	0.067	0.037	1.153
Q53H12	Acylglycerol kinase, mitochondrial OS=Homo sapiens GN=AGK PE=1 SV=2 - [AGK_HUMAN]	5.92	1	1	1	4		22.54	22.80		23.55	24.33	2	2		22.61	22.86		23.77	24.07	22.73	23.92	0.026	0.019	1.183
P16949	Stathmin OS=Homo sapiens GN=STMN1 PE=1 SV=3 - [STMN1_HUMAN]	38.26	2	5	5	25	22.23	24.37	23.91	25.51	25.03	25.69	3	3	22.40	24.44	23.97	25.26	25.25	25.42	23.60	25.31	0.052	0.030	1.707
P04844	Dolichyl-diphosphooligosaccharide--protein glycosyltransferase subunit 2 OS=Homo sapiens GN=RPN2 PE=1 SV=3 - [RPN2_HUMAN]	29.48	1	11	11	39	23.12	23.71	24.09	23.42	24.07	25.09	3	3	23.28	23.78	24.15	23.18	24.29	24.83	23.74	24.10	0.546	0.182	0.361
Q8IVM0	Coiled-coil domain-containing protein 50 OS=Homo sapiens GN=CCDC50 PE=1 SV=1 - [CCD50_HUMAN]	22.88	1	5	5	12		23.53	23.61	22.63	21.92	22.55	2	3		23.60	23.67	22.38	22.13	22.29	23.64	22.27	0.001	0.003	-1.365
P26368	Splicing factor U2AF 65 kDa subunit OS=Homo sapiens GN=U2AF2 PE=1 SV=4 - [U2AF2_HUMAN]	21.68	1	7	7	19	23.07	23.08		24.23	23.84	24.46	2	3	23.23	23.15		23.98	24.06	24.20	23.19	24.08	0.002	0.004	0.890
P17812	CTP synthase 1 OS=Homo sapiens GN=CTPS1 PE=1 SV=2 - [PYRG1_HUMAN]	27.41	2	11	11	32	23.56	23.05	23.49	23.79	23.94	23.86	3	3	23.72	23.12	23.55	23.55	24.16	23.60	23.46	23.77	0.315	0.117	0.303
Q8WVJ2	NudC domain-containing protein 2 OS=Homo sapiens GN=NUDCD2 PE=1 SV=1 - [NUDC2_HUMAN]	41.40	1	4	4	16	23.11	22.74	22.49	23.33	23.89	24.06	3	3	23.27	22.81	22.55	23.09	24.10	23.80	22.88	23.66	0.098	0.050	0.786
P61160	Actin-related protein 2 OS=Homo sapiens GN=ACTR2 PE=1 SV=1 - [ARP2_HUMAN]	39.09	1	14	14	49	24.69	24.66	24.65	25.67	24.84	25.94	3	3	24.85	24.73	24.71	25.42	25.05	25.68	24.76	25.39	0.029	0.021	0.623
Q9H3U1	Protein unc-45 homolog A OS=Homo sapiens GN=UNC45A PE=1 SV=1 - [UN45A_HUMAN]	7.20	1	4	4	7	20.91	20.02	21.53		21.20	22.30	3	2	21.07	20.09	21.59		21.41	22.04	20.92	21.72	0.281	0.107	0.804
Q9BTU6	Phosphatidylinositol 4-kinase type 2-alpha OS=Homo sapiens GN=PI4K2A PE=1 SV=1 - [P4K2A_HUMAN]	13.57	1	6	6	12	22.18	22.05	21.03	22.92		21.52	3	2	22.34	22.12	21.09	22.67		21.25	21.85	21.96	0.887	0.257	0.112
P49189	4-trimethylaminobutyraldehyde dehydrogenase OS=Homo sapiens GN=ALDH9A1 PE=1 SV=3 - [AL9A1_HUMAN]	37.85	1	15	15	60	23.23	23.69	23.81	24.58	23.86	24.99	3	3	23.39	23.76	23.87	24.33	24.08	24.72	23.67	24.38	0.041	0.026	0.704
CON_006919981	SWISS-PROT:Q58D62 Bos taurus Fetuin-B precursor	27.65	1	8	8	53	26.12	24.61	25.21	26.30	25.58	26.52	3	3	26.28	24.68	25.27	26.06	25.79	26.26	25.41	26.04	0.268	0.103	0.625
Q9Y2J2	Band 4.1-like protein 3 OS=Homo sapiens GN=EPB41L3 PE=1 SV=2 - [E41L3_HUMAN]	15.55	1	8	11	34	23.39	23.10	22.82	24.25	23.40	24.43	3	3	23.55	23.17	22.88	24.00	23.62	24.17	23.20	23.93	0.045	0.028	0.730
Q12907	Vesicular integral-membrane protein VIP36 OS=Homo sapiens GN=LMAN2 PE=1 SV=1 - [LMAN2_HUMAN]	40.17	1	10	10	30	23.19	24.44	23.75	24.76	24.78	26.07	3	3	23.35	24.51	23.81	24.51	25.00	25.81	23.89	25.11	0.074	0.040	1.220
Q00325	Phosphate carrier protein, mitochondrial OS=Homo sapiens GN=SLC25A3 PE=1 SV=2 - [MPCP_HUMAN]	24.59	1	9	9	38	24.25	25.30	25.43	25.37	25.53	27.06	3	3	24.41	25.36	25.49	25.12	25.74	26.80	25.09	25.89	0.252	0.098	0.797
P12236	ADP/ATP translocase 3 OS=Homo sapiens GN=SLC25A6 PE=1 SV=4 - [ADT3_HUMAN]	48.99	2	3	13	53		25.46	25.65	25.88	26.72	27.61	2	3		25.52	25.71	25.63	26.94	27.35	25.62	26.64	0.226	0.091	1.023
Q15046	Lysine--tRNA ligase OS=Homo sapiens GN=KARS PE=1 SV=3 - [SYK_HUMAN]	30.82	1	15	15	35	23.66	23.92	23.44	24.02	26.06	24.67	3	3	23.82	23.99	23.50	23.77	26.27	24.41	23.77	24.82	0.242	0.095	1.048
P58546	Myotrophin OS=Homo sapiens GN=MTPN PE=1 SV=2 - [MTPN_HUMAN]	44.07	1	4	4	17	23.49	24.37	23.80	24.86	24.53	25.11	3	3	23.66	24.44	23.86	24.62	24.75	24.85	23.98	24.74	0.036	0.024	0.753
O95816	BAG family molecular chaperone regulator 2 OS=Homo sapiens GN=BAG2 PE=1 SV=1 - [BAG2_HUMAN]	61.14	1	12	12	51	24.99	24.55	24.49	29.08	25.18	27.02	3	3	25.15	24.62	24.55	28.83	25.39	26.76	24.77	26.99	0.095	0.048	2.220
P43307	Translocon-associated protein subunit alpha OS=Homo sapiens GN=SSR1 PE=1 SV=3 - [SSRA_HUMAN]	21.68	1	4	4	12	22.64	24.10	24.29		25.21	25.00	3	2	22.80	24.17	24.35		25.42	24.74	23.77	25.08	0.150	0.067	1.310
P60468	Protein transport protein Sec61 subunit beta OS=Homo sapiens GN=SEC61B PE=1 SV=2 - [SC61B_HUMAN]	27.08	1	2	2	8	21.94	23.34	23.69	24.00	23.75	24.69	3	3	22.10	23.41	23.75	23.75	23.97	24.43	23.09	24.05	0.149	0.067	0.963
Q14203	Dynactin subunit 1 OS=Homo sapiens GN=DCTN1 PE=1 SV=3 - [DCTN1_HUMAN]	12.21	2	13	14	33	25.25	25.27	24.76	24.31	23.62	24.49	3	3	25.41	25.34	24.82	24.07	23.84	24.23	25.19	24.04	0.006	0.008	-1.148
Q9Y277	Voltage-dependent anion-selective channel protein 3 OS=Homo sapiens GN=VDAC3 PE=1 SV=1 - [VDAC3_HUMAN]	16.96	1	3	4	12	23.49	25.29	25.46	25.16	25.99	25.86	3	3	23.65	25.36	25.52	24.92	26.21	25.60	24.84	25.57	0.359	0.129	0.731
Q3SY84	Keratin, type II cytoskeletal 71 OS=Homo sapiens GN=KRT71 PE=1 SV=3 - [K2C71_HUMAN]	4.97	2	1	3	13	24.24	24.16	24.07	24.65	24.26	24.75	3	3	24.40	24.22	24.13	24.41	24.48	24.49	24.25	24.46	0.071	0.038	0.208
Q15059	Bromodomain-containing protein 3 OS=Homo sapiens GN=BRD3 PE=1 SV=1 - [BRD3_HUMAN]	1.24	2	1	1	10	32.76	33.72	33.53	32.09	31.15	32.55	3	3	32.93	33.79	33.59	31.85	31.37	32.29	33.43	31.83	0.013	0.012	-1.600
Q15366	Poly(rC)-binding protein 2 OS=Homo sapiens GN=PCBP2 PE=1 SV=1 - [PCBP2_HUMAN]	49.04	2	7	13	67	26.49	26.37	26.51	26.81	26.60	27.37	3	3	26.65	26.44	26.57	26.56	26.82	27.11	26.55	26.83	0.177	0.076	0.275
Q6IQ22	Ras-related protein Rab-12 OS=Homo sapiens GN=RAB12 PE=1 SV=3 - [RAB12_HUMAN]	13.52	7	2	3	16	26.71	27.71	27.78	28.57	28.02	28.82	3	3	26.87	27.78	27.84	28.33	28.23	28.56	27.50	28.38	0.056	0.032	0.879
Q99829	Copine-1 OS=Homo sapiens GN=CPNE1 PE=1 SV=1 - [CPNE1_HUMAN]	60.52	1	21	21	100	25.29	25.30	25.33	26.23	25.41	26.31	3	3	25.46	25.37	25.39	25.98	25.62	26.05	25.40	25.89	0.023	0.018	0.482
P05141	ADP/ATP translocase 2 OS=Homo sapiens GN=SLC25A5 PE=1 SV=7 - [ADT2_HUMAN]	45.30	2	6	14	53		25.48	25.65	25.89	26.72	27.61	2	3		25.55	25.71	25.65	26.94	27.35	25.63	26.64	0.225	0.091	1.016
Q13155	Aminoacyl tRNA synthase complex-interacting multifunctional protein 2 OS=Homo sapiens GN=AIMP2 PE=1 SV=2 - [AIMP2_HUMAN]	20.00	1	4	4	16	22.72		22.21	24.44	24.43	24.92	2	3	22.89		22.27	24.19	24.65	24.66	22.58	24.50	0.008	0.009	1.921
Q9H4A4	Aminopeptidase B OS=Homo sapiens GN=RNPEP PE=1 SV=2 - [AMPB_HUMAN]	10.92	1	4	4	9	22.57	22.30	22.26	23.67	22.54	24.00	3	3	22.73	22.37	22.32	23.42	22.76	23.73	22.47	23.30	0.058	0.033	0.834
Q58FG0	Putative heat shock protein HSP 90-alpha A5 OS=Homo sapiens GN=HSP90AA5P PE=2 SV=1 - [HS905_HUMAN]	15.57	1	1	6	38	28.26	28.06	28.33	28.75	28.13	28.94	3	3	28.42	28.13	28.39	28.50	28.35	28.68	28.31	28.51	0.210	0.087	0.197
Q9H2U2	Inorganic pyrophosphatase 2, mitochondrial OS=Homo sapiens GN=PPA2 PE=1 SV=2 - [IPYR2_HUMAN]	8.38	1	1	2	4	24.08		21.52	25.03		23.02	2	2	24.24		21.58	24.78		22.76	22.91	23.77	0.659	0.210	0.858
O75844	CAAX prenyl protease 1 homolog OS=Homo sapiens GN=ZMPSTE24 PE=1 SV=2 - [FACE1_HUMAN]	12.63	1	5	5	14	20.87	21.75	22.22	22.11	21.88	23.54	3	3	21.03	21.82	22.28	21.87	22.10	23.28	21.71	22.42	0.282	0.107	0.706
Q9UG01	Intraflagellar transport protein 172 homolog OS=Homo sapiens GN=IFT172 PE=1 SV=2 - [IF172_HUMAN]	0.74	1	1	1	16	32.35	33.39	33.35	31.35		31.76	3	2	32.51	33.46	33.41	31.10		31.50	33.13	31.30	0.023	0.018	-1.828
CON_007155481	SWISS-PROT:P15497 Bos taurus Apolipoprotein A-I precursor	66.04	2	19	20	120	28.61	27.31	27.71	28.90	28.37	28.08	3	3	28.77	27.38	27.77	28.65	28.58	27.82	27.97	28.35	0.482	0.165	0.381
Q9NQW7	Xaa-Pro aminopeptidase 1 OS=Homo sapiens GN=XPNPEP1 PE=1 SV=3 - [XPP1_HUMAN]	8.67	1	4	4	9	21.08	21.23	21.28	23.49		22.79	3	2	21.24	21.30	21.34	23.24		22.53	21.29	22.89	0.009	0.010	1.593
O60361	Putative nucleoside diphosphate kinase OS=Homo sapiens GN=NME2P1 PE=5 SV=1 - [NDK8_HUMAN]	67.15	1	1	8	78	26.65	27.62	27.58	28.45	27.91	28.56	3	3	26.81	27.68	27.64	28.21	28.12	28.30	27.38	28.21	0.044	0.028	0.832
P39687	Acidic leucine-rich nuclear phosphoprotein 32 family member A OS=Homo sapiens GN=ANP32A PE=1 SV=1 - [AN32A_HUMAN]	18.47	2	3	5	23	22.84	23.34	23.72	23.75	23.53	24.12	3	3	23.00	23.41	23.78	23.51	23.75	23.86	23.40	23.70	0.284	0.107	0.306
Q7RTS7	Keratin, type II cytoskeletal 74 OS=Homo sapiens GN=KRT74 PE=1 SV=2 - [K2C74_HUMAN]	7.37	3	1	4	14	24.29	24.16	24.55	24.77	24.26	24.75	3	3	24.45	24.22	24.61	24.52	24.48	24.49	24.43	24.50	0.579	0.190	0.068
P05114	Non-histone chromosomal protein HMG-14 OS=Homo sapiens GN=HMGN1 PE=1 SV=3 - [HMGN1_HUMAN]	26.00	1	2	2	10	19.98	20.53		23.02	22.44	22.24	2	3	20.14	20.60		22.77	22.65	21.98	20.37	22.47	0.010	0.011	2.102
Q7Z6Z7	E3 ubiquitin-protein ligase HUWE1 OS=Homo sapiens GN=HUWE1 PE=1 SV=3 - [HUWE1_HUMAN]	13.37	1	35	35	71	21.40		20.59	23.91	24.38	24.60	2	3	21.56		20.65	23.66	24.60	24.34	21.10	24.20	0.008	0.009	3.096
P13796	Plastin-2 OS=Homo sapiens GN=LCP1 PE=1 SV=6 - [PLSL_HUMAN]	63.80	1	23	29	115	24.26	24.38	24.08	26.17	24.59	26.28	3	3	24.42	24.45	24.14	25.93	24.81	26.02	24.33	25.58	0.035	0.024	1.250
P55786	Puromycin-sensitive aminopeptidase OS=Homo sapiens GN=NPEPPS PE=1 SV=2 - [PSA_HUMAN]	20.46	2	12	12	27	22.37	21.62		24.56	22.99	24.61	2	3	22.53	21.69		24.32	23.21	24.35	22.11	23.96	0.049	0.030	1.851
P37837	Transaldolase OS=Homo sapiens GN=TALDO1 PE=1 SV=2 - [TALDO_HUMAN]	30.27	1	11	11	47	23.64	23.87	23.67	24.59	23.98	24.79	3	3	23.80	23.94	23.73	24.35	24.20	24.52	23.82	24.36	0.009	0.010	0.535
P15531	Nucleoside diphosphate kinase A OS=Homo sapiens GN=NME1 PE=1 SV=1 - [NDKA_HUMAN]	85.53	1	6	10	97	26.65	27.68	27.53	28.39	27.93	28.55	3	3	26.81	27.75	27.59	28.15	28.15	28.29	27.39	28.20	0.051	0.030	0.809
Q9Y6M4	Casein kinase I isoform gamma-3 OS=Homo sapiens GN=CSNK1G3 PE=1 SV=2 - [KC1G3_HUMAN]	35.12	1	6	14	48	24.37	24.84	25.09	23.97	22.20	23.65	3	3	24.54	24.91	25.15	23.72	22.42	23.39	24.87	23.18	0.017	0.015	-1.691
P09429	High mobility group protein B1 OS=Homo sapiens GN=HMGB1 PE=1 SV=3 - [HMGB1_HUMAN]	40.93	3	5	6	17		23.12	23.11	24.70	24.94	24.24	2	3		23.18	23.17	24.46	25.15	23.98	23.18	24.53	0.054	0.032	1.352
Q9H0E2	Toll-interacting protein OS=Homo sapiens GN=TOLLIP PE=1 SV=1 - [TOLIP_HUMAN]	28.10	1	7	7	25	24.14	24.10	23.51	24.25	24.30	23.98	3	3	24.30	24.17	23.57	24.00	24.51	23.72	24.02	24.08	0.855	0.252	0.063
Q9UBQ7	Glyoxylate reductase/hydroxypyruvate reductase OS=Homo sapiens GN=GRHPR PE=1 SV=1 - [GRHPR_HUMAN]	26.83	1	5	5	10		22.70	22.57	23.21	23.22	22.89	2	3		22.77	22.63	22.97	23.43	22.63	22.70	23.01	0.382	0.135	0.310
P0CG48	Polyubiquitin-C OS=Homo sapiens GN=UBC PE=1 SV=3 - [UBC_HUMAN]	83.50	3	1	8	152	32.14	33.35	33.51	31.95	31.47	32.09	3	3	32.30	33.42	33.57	31.71	31.69	31.83	33.10	31.74	0.028	0.021	-1.356
P62979	Ubiquitin-40S ribosomal protein S27a OS=Homo sapiens GN=RPS27A PE=1 SV=2 - [RS27A_HUMAN]	59.62	2	3	10	174	32.14	33.35	33.51	31.95	31.47	32.09	3	3	32.30	33.42	33.57	31.71	31.69	31.83	33.10	31.74	0.028	0.021	-1.356
P58107	Epiplakin OS=Homo sapiens GN=EPPK1 PE=1 SV=2 - [EPIPL_HUMAN]	5.42	1	2	5	16	23.96	23.49	23.39	24.84	23.82	25.25	3	3	24.12	23.56	23.45	24.60	24.04	24.99	23.71	24.54	0.073	0.039	0.832
P49915	GMP synthase [glutamine-hydrolyzing] OS=Homo sapiens GN=GMPS PE=1 SV=1 - [GUAA_HUMAN]	13.71	1	7	7	21	22.26	22.68		23.85	23.65	23.94	2	3	22.42	22.75		23.60	23.87	23.68	22.59	23.72	0.006	0.008	1.130
P53794	Sodium/myo-inositol cotransporter OS=Homo sapiens GN=SLC5A3 PE=3 SV=2 - [SC5A3_HUMAN]	11.98	1	5	5	16	21.73	22.31	23.09	23.26		24.02	3	2	21.89	22.38	23.15	23.01		23.76	22.47	23.38	0.195	0.082	0.911
CON_006905341	SWISS-PROT:Q29443| Bos taurus Gene_Symbol=TF Serotransferrin	68.89	3	39	39	256	26.48	26.67	26.35	27.73	26.88	26.97	3	3	26.64	26.74	26.41	27.49	27.09	26.71	26.60	27.10	0.112	0.054	0.500

P63027	Vesicle-associated membrane protein 2 OS=Homo sapiens GN=VAMP2 PE=1 SV=3 - [VAMP2_HUMAN]	34.48	2	1	4	18	25.13	26.72	26.67	25.02	25.32	25.24	3	3	25.29	26.79	26.73	24.78	25.54	24.98	26.27	25.10	0.095	0.048	-1.173
P07602	Prosaposin OS=Homo sapiens GN=PSAP PE=1 SV=2 - [SAP_HUMAN]	25.00	1	11	11	36	23.00	24.40	24.90	25.28	25.46	25.29	3	3	23.17	24.47	24.96	25.03	25.68	25.03	24.20	25.25	0.144	0.065	1.047
Q99447	Ethanolamine-phosphate cytidylyltransferase OS=Homo sapiens GN=PCYT2 PE=1 SV=1 - [PCY2_HUMAN]	6.94	1	2	2	7	20.66	20.29	20.68	20.72	21.08	21.36	3	3	20.82	20.36	20.74	20.47	21.29	21.10	20.64	20.96	0.333	0.123	0.314
Q9HCP0	Casein kinase I isoform gamma-1 OS=Homo sapiens GN=CSNK1G1 PE=1 SV=1 - [KC1G1_HUMAN]	21.09	1	3	9	33	23.93	24.38	24.56	23.53	21.49	23.06	3	3	24.09	24.45	24.62	23.28	21.71	22.80	24.39	22.60	0.022	0.017	-1.791
Q13162	Peroxiredoxin-4 OS=Homo sapiens GN=PRDX4 PE=1 SV=1 - [PRDX4_HUMAN]	48.34	1	7	9	30	26.58	25.87	24.89	27.13	26.75	27.33	3	3	26.74	25.94	24.95	26.88	26.97	27.07	25.88	26.97	0.102	0.051	1.096
CON_007025901	SWISS-PROT:Q28065 Bos taurus C4b-binding protein alpha chain precursor	11.97	1	5	5	17	23.17	23.71	23.58	24.00	23.81	24.71	3	3	23.33	23.78	23.64	23.76	24.02	24.45	23.58	24.08	0.113	0.054	0.492
Q99959	Plakophilin-2 OS=Homo sapiens GN=PKP2 PE=1 SV=2 - [PKP2_HUMAN]	15.89	1	11	11	23	23.50	23.63	23.44	23.15	21.94	22.78	3	3	23.66	23.70	23.50	22.90	22.16	22.52	23.62	22.53	0.008	0.009	-1.092
Q15041	ADP-ribosylation factor-like protein 6-interacting protein 1 OS=Homo sapiens GN=ARL6IP1 PE=1 SV=2 - [AR6P1_HUMAN]	11.82	1	3	3	9	21.69	22.27	22.01	22.81		23.89	3	2	21.85	22.34	22.06	22.57		23.63	22.08	23.10	0.103	0.051	1.015
P50502	Hsc70-interacting protein OS=Homo sapiens GN=ST13 PE=1 SV=2 - [F10A1_HUMAN]	25.47	3	8	8	39	24.11	24.63	24.06	24.64	24.90	25.42	3	3	24.27	24.70	24.12	24.40	25.12	25.16	24.36	24.89	0.154	0.069	0.529
P10768	S-formylglutathione hydrolase OS=Homo sapiens GN=ESD PE=1 SV=2 - [ESTD_HUMAN]	43.97	1	7	7	21	23.52	22.95	23.97	24.13	24.19	24.14	3	3	23.68	23.02	24.03	23.88	24.40	23.88	23.57	24.06	0.232	0.093	0.483
P18669	Phosphoglycerate mutase 1 OS=Homo sapiens GN=PGAM1 PE=1 SV=2 - [PGAM1_HUMAN]	62.20	3	11	11	94	26.56	27.37	27.41	28.22	27.59	28.25	3	3	26.72	27.44	27.47	27.97	27.81	27.99	27.21	27.92	0.047	0.029	0.715
Q14126	Desmoglein-2 OS=Homo sapiens GN=DSG2 PE=1 SV=2 - [DSG2_HUMAN]	23.26	1	15	15	66	25.23	24.52	24.66	24.51	22.81	24.36	3	3	25.39	24.59	24.72	24.27	23.02	24.10	24.90	23.80	0.076	0.040	-1.100
Q9H4M9	EH domain-containing protein 1 OS=Homo sapiens GN=EHD1 PE=1 SV=2 - [EHD1_HUMAN]	77.15	2	21	35	214	27.71	27.28	26.70	26.64	26.15	26.64	3	3	27.88	27.35	26.76	26.39	26.36	26.38	27.33	26.38	0.042	0.027	-0.952
P26599	Polypyrimidine tract-binding protein 1 OS=Homo sapiens GN=PTBP1 PE=1 SV=1 - [PTBP1_HUMAN]	44.26	3	15	15	68	24.23	25.15	25.17	25.70	25.33	25.92	3	3	24.39	25.21	25.23	25.46	25.55	25.66	24.94	25.56	0.097	0.049	0.612
P12081	Histidine--tRNA ligase, cytoplasmic OS=Homo sapiens GN=HARS PE=1 SV=2 - [SYHC_HUMAN]	44.79	2	19	19	41	23.22	24.01	23.43	24.81	24.11	25.25	3	3	23.38	24.08	23.49	24.56	24.32	24.99	23.65	24.63	0.029	0.021	0.976
O00186	Syntaxin-binding protein 3 OS=Homo sapiens GN=STXBP3 PE=1 SV=2 - [STXB3_HUMAN]	23.82	1	11	11	45	23.02	22.54	22.34	24.23	22.85	24.73	3	3	23.18	22.61	22.40	23.98	23.06	24.47	22.73	23.84	0.080	0.042	1.109
P04004	Vitronectin OS=Homo sapiens GN=VTN PE=1 SV=1 - [VTNC_HUMAN]	10.46	1	4	4	19	27.09	24.39	24.74	27.29	27.40	26.97	3	3	27.26	24.46	24.80	27.04	27.62	26.71	25.51	27.12	0.154	0.069	1.618
Q9UQE7	Structural maintenance of chromosomes protein 3 OS=Homo sapiens GN=SMC3 PE=1 SV=2 - [SMC3_HUMAN]	7.40	1	7	8	20	21.33	21.04	21.15	23.38	21.14	23.29	3	3	21.49	21.11	21.21	23.13	21.36	23.03	21.27	22.51	0.103	0.051	1.234
Q13247	Serine/arginine-rich splicing factor 6 OS=Homo sapiens GN=SRSF6 PE=1 SV=2 - [SRSF6_HUMAN]	15.12	1	3	6	26	22.01	23.46	24.28	24.79	24.61	24.75	3	3	22.17	23.53	24.34	24.55	24.83	24.49	23.34	24.62	0.117	0.056	1.278
P22692	Insulin-like growth factor-binding protein 4 OS=Homo sapiens GN=IGFBP4 PE=1 SV=2 - [IBP4_HUMAN]	32.95	1	5	5	16	21.77	22.33		23.46	23.30	23.44	2	3	21.93	22.40		23.22	23.52	23.18	22.16	23.31	0.014	0.013	1.142
Q9NV96	Cell cycle control protein 50A OS=Homo sapiens GN=TMEM30A PE=1 SV=1 - [CC50A_HUMAN]	10.25	1	3	3	9	22.86		22.95	24.52	23.14	23.90	2	3	23.02		23.01	24.28	23.35	23.64	23.01	23.76	0.126	0.059	0.743
O75369	Filamin-B OS=Homo sapiens GN=FLNB PE=1 SV=2 - [FLNB_HUMAN]	55.61	2	86	97	394	25.64	25.54	25.59	26.10	25.63	26.43	3	3	25.80	25.61	25.65	25.85	25.84	26.16	25.69	25.95	0.092	0.047	0.266
O15260	Surfeit locus protein 4 OS=Homo sapiens GN=SURF4 PE=1 SV=3 - [SURF4_HUMAN]	6.69	1	1	1	12	21.36	22.73	22.83	22.70	22.79	23.89	3	3	21.52	22.80	22.89	22.45	23.01	23.63	22.40	23.03	0.325	0.120	0.626
P53675	Clathrin heavy chain 2 OS=Homo sapiens GN=CLTCL1 PE=1 SV=2 - [CLH2_HUMAN]	12.74	1	1	21	145	26.07	26.37	26.29	27.81	26.43	27.79	3	3	26.23	26.44	26.35	27.56	26.65	27.53	26.34	27.25	0.041	0.026	0.909
Q9BVK6	Transmembrane emp24 domain-containing protein 9 OS=Homo sapiens GN=TMED9 PE=1 SV=2 - [TMED9_HUMAN]	26.38	1	6	7	26	23.99	24.28	23.81	24.65	24.40	25.03	3	3	24.15	24.35	23.87	24.41	24.62	24.77	24.12	24.60	0.053	0.031	0.475
P26006	Integrin alpha-3 OS=Homo sapiens GN=ITGA3 PE=1 SV=5 - [ITA3_HUMAN]	38.06	1	34	34	278	29.20	29.32	29.30	28.80	27.99	28.98	3	3	29.37	29.39	29.36	28.55	28.20	28.72	29.37	28.49	0.005	0.007	-0.877
P22413	Ectonucleotide pyrophosphatase/phosphodiesterase family member 1 OS=Homo sapiens GN=ENPP1 PE=1 SV=2 - [ENPP1_HUMAN]	55.46	1	37	37	244	27.21	27.71	27.38	26.76	25.53	26.65	3	3	27.37	27.78	27.44	26.51	25.74	26.38	27.53	26.21	0.008	0.009	-1.316
Q04721	Neurogenic locus notch homolog protein 2 OS=Homo sapiens GN=NOTCH2 PE=1 SV=3 - [NOTC2_HUMAN]	9.63	2	14	14	35	22.96	23.40	23.17	24.07	23.62	23.56	3	3	23.13	23.47	23.23	23.82	23.83	23.30	23.28	23.65	0.142	0.065	0.375
P62158	Calmodulin OS=Homo sapiens GN=CALM1 PE=1 SV=2 - [CALM_HUMAN]	60.40	3	11	11	80	26.35	28.07	28.09	28.90	28.47	29.11	3	3	26.51	28.14	28.15	28.66	28.69	28.85	27.60	28.73	0.108	0.053	1.130
P35221	Catenin alpha-1 OS=Homo sapiens GN=CTNNA1 PE=1 SV=1 - [CTNA1_HUMAN]	32.23	2	17	17	61	24.47	24.27	24.55	25.18	24.29	25.16	3	3	24.63	24.34	24.61	24.94	24.50	24.90	24.53	24.78	0.207	0.086	0.253
P17301	Integrin alpha-2 OS=Homo sapiens GN=ITGA2 PE=1 SV=1 - [ITA2_HUMAN]	45.64	1	42	42	295	26.85	27.18	27.03	28.52	27.21	28.66	3	3	27.02	27.25	27.09	28.27	27.43	28.40	27.12	28.03	0.042	0.027	0.916
O95865	N(G),N(G)-dimethylarginine dimethylaminohydrolase 2 OS=Homo sapiens GN=DDAH2 PE=1 SV=1 - [DDAH2_HUMAN]	62.11	1	7	8	27	21.94	23.23	22.77	24.24	23.36	24.28	3	3	22.10	23.30	22.83	24.00	23.57	24.02	22.74	23.86	0.041	0.026	1.120
Q9NX63	MICOS complex subunit MIC19 OS=Homo sapiens GN=CHCHD3 PE=1 SV=1 - [MIC19_HUMAN]	30.40	1	7	7	15		20.83	22.19		24.05	24.00	2	2		20.90	22.25		24.27	23.74	21.58	24.00	0.079	0.042	2.428
Q9HD45	Transmembrane 9 superfamily member 3 OS=Homo sapiens GN=TM9SF3 PE=1 SV=2 - [TM9S3_HUMAN]	12.56	1	5	5	19	24.26	24.36	23.96	24.77	24.39	24.69	3	3	24.42	24.43	24.02	24.52	24.61	24.43	24.29	24.52	0.183	0.078	0.230
P08758	Annexin A5 OS=Homo sapiens GN=ANXA5 PE=1 SV=2 - [ANXA5_HUMAN]	77.81	1	25	26	222	27.75	28.75	28.31	29.42	28.93	29.14	3	3	27.91	28.82	28.37	29.17	29.14	28.88	28.37	29.06	0.067	0.037	0.697
P52569	Cationic amino acid transporter 2 OS=Homo sapiens GN=SLC7A2 PE=1 SV=2 - [CTR2_HUMAN]	3.50	1	1	2	6	24.27	24.49	24.56	24.58		24.73	3	2	24.43	24.56	24.62	24.34		24.47	24.54	24.41	0.230	0.092	-0.132
Q9UGV2	Protein NDRG3 OS=Homo sapiens GN=NDRG3 PE=1 SV=2 - [NDRG3_HUMAN]	43.20	1	9	9	23	23.08	23.44	22.55	24.29	23.67	24.93	3	3	23.24	23.51	22.61	24.04	23.89	24.67	23.12	24.20	0.040	0.026	1.077
P50750	Cyclin-dependent kinase 9 OS=Homo sapiens GN=CDK9 PE=1 SV=3 - [CDK9_HUMAN]	8.06	18	2	3	14	23.69	24.09	25.08	23.66	22.49	23.66	3	3	23.85	24.16	25.13	23.41	22.71	23.40	24.38	23.17	0.055	0.032	-1.209
P13726	Tissue factor OS=Homo sapiens GN=F3 PE=1 SV=1 - [TF_HUMAN]	34.58	1	9	9	62	27.96	28.48	28.38	27.75	26.77	27.61	3	3	28.12	28.55	28.44	27.50	26.98	27.35	28.37	27.28	0.006	0.007	-1.089
Q15043	Zinc transporter ZIP14 OS=Homo sapiens GN=SLC39A14 PE=1 SV=3 - [S39AE_HUMAN]	8.33	1	3	3	25	25.43	24.71	25.38	25.20	23.96	25.22	3	3	25.59	24.78	25.44	24.95	24.18	24.96	25.27	24.70	0.187	0.079	-0.573
P60174	Triosephosphate isomerase OS=Homo sapiens GN=TPI1 PE=1 SV=3 - [TPIS_HUMAN]	75.17	1	18	18	130	26.44	27.72	27.55	28.25	27.83	28.66	3	3	26.60	27.79	27.61	28.01	28.04	28.40	27.33	28.15	0.104	0.051	0.816
O95373	Importin-7 OS=Homo sapiens GN=IPO7 PE=1 SV=1 - [IPO7_HUMAN]	28.32	2	20	20	59	22.92	23.23	23.39	24.59	23.49	26.60	3	3	23.08	23.29	23.45	24.34	23.71	26.34	23.28	24.80	0.130	0.060	1.519
O43488	Aflatoxin B1 aldehyde reductase member 2 OS=Homo sapiens GN=AKR7A2 PE=1 SV=3 - [ARK72_HUMAN]	23.12	2	5	5	14	23.22	22.96	23.23	23.75	23.56	23.24	3	3	23.38	23.03	23.29	23.50	23.78	22.98	23.23	23.42	0.511	0.173	0.187
Q86VP6	Cullin-associated NEDD8-dissociated protein 1 OS=Homo sapiens GN=CAND1 PE=1 SV=2 - [CAND1_HUMAN]	11.22	1	9	9	22	22.10	22.95	23.22	23.75		24.12	3	2	22.26	23.02	23.28	23.50		23.86	22.86	23.68	0.142	0.065	0.827
O60343	TBC1 domain family member 4 OS=Homo sapiens GN=TBC1D4 PE=1 SV=2 - [TBCD4_HUMAN]	2.31	1	2	2	12	19.88	19.12	19.29	20.01	21.45	20.72	3	3	20.05	19.19	19.35	19.76	21.67	20.45	19.53	20.63	0.149	0.067	1.098
O95747	Serine/threonine-protein kinase OSR1 OS=Homo sapiens GN=OXSR1 PE=1 SV=1 - [OXSR1_HUMAN]	40.23	3	15	16	63	23.53	23.71	23.69	24.22	23.72	24.56	3	3	23.69	23.77	23.75	23.98	23.93	24.30	23.74	24.07	0.048	0.029	0.333
Q13011	Delta(3,5)-Delta(2,4)-dienoyl-CoA isomerase, mitochondrial OS=Homo sapiens GN=ECH1 PE=1 SV=2 - [ECH1_HUMAN]	32.62	1	8	8	22		22.40	21.21	22.96	24.15	24.43	2	3		22.47	21.27	22.72	24.37	24.17	21.87	23.75	0.102	0.051	1.885
P30041	Peroxiredoxin-6 OS=Homo sapiens GN=PRDX6 PE=1 SV=3 - [PRDX6_HUMAN]	75.89	1	16	16	98	26.23	26.12	25.84	26.63	26.28	26.90	3	3	26.39	26.19	25.90	26.39	26.49	26.64	26.16	26.51	0.094	0.048	0.349
Q9NX58	Cell growth-regulating nucleolar protein OS=Homo sapiens GN=LYAR PE=1 SV=2 - [LYAR_HUMAN]	37.99	1	11	11	29	23.20	23.09	18.23	24.97	24.50	24.54	3	3	23.36	23.16	18.29	24.72	24.72	24.28	21.60	24.57	0.149	0.067	2.972
Q08380	Galectin-3-binding protein OS=Homo sapiens GN=LGALS3BP PE=1 SV=1 - [LG3BP_HUMAN]	52.99	1	23	23	170	29.26	27.71	27.42	29.27	29.51	28.94	3	3	29.42	27.78	27.48	29.02	29.73	28.68	28.23	29.14	0.248	0.097	0.915
P26639	Threonine--tRNA ligase, cytoplasmic OS=Homo sapiens GN=TARS PE=1 SV=3 - [SYTC_HUMAN]	54.36	2	31	32	122	23.80	24.26	24.38	25.28	24.27	25.57	3	3	23.97	24.33	24.44	25.03	24.49	25.31	24.24	24.94	0.067	0.037	0.699
O00592	Podocalyxin OS=Homo sapiens GN=PODXL PE=1 SV=2 - [PODXL_HUMAN]	15.95	1	7	7	30	25.43	25.71	24.94	23.91	23.45	24.87	3	3	25.59	25.78	25.00	23.66	23.66	24.61	25.45	23.98	0.020	0.016	-1.475
O43809	Cleavage and polyadenylation specificity factor subunit 5 OS=Homo sapiens GN=NUDT21 PE=1 SV=1 - [CPSF5_HUMAN]	37.44	1	6	6	31	23.65	23.69	23.58	23.71	24.06	24.31	3	3	23.81	23.76	23.64	23.46	24.28	24.05	23.74	23.93	0.481	0.164	0.192
O95297	Myelin protein zero-like protein 1 OS=Homo sapiens GN=MPZL1 PE=1 SV=1 - [MPZL1_HUMAN]	30.48	1	7	7	36	24.68	25.06	24.98	24.52	22.42	23.52	3	3	24.84	25.13	25.04	24.27	22.64	23.26	25.00	23.39	0.029	0.021	-1.615
Q13813	Spectrin alpha chain, non-erythrocytic 1 OS=Homo sapiens GN=SPTAN1 PE=1 SV=3 - [SPTN1_HUMAN]	43.69	1	83	83	300	24.12	24.48	23.98	24.66	24.74	25.53	3	3	24.28	24.55	24.04	24.41	24.95	25.27	24.29	24.88	0.114	0.055	0.586
O75954	Tetraspanin-9 OS=Homo sapiens GN=TSPAN9 PE=1 SV=1 - [TSN9_HUMAN]	24.69	1	4	4	17	24.35	24.77	25.00	22.94	19.39	23.75	3	3	24.51	24.84	25.06	22.70	19.60	23.49	24.80	21.93	0.074	0.040	-2.873
Q8NGA1	Olfactory receptor 1M1 OS=Homo sapiens GN=OR1M1 PE=2 SV=1 - [OR1M1_HUMAN]	2.24	1	1	1	6	26.41		27.11	28.05	27.77	27.99	2	3	26.57		27.17	27.80	27.99	27.73	26.87	27.84	0.028	0.021	0.971
Q52LW3	Rho GTPase-activating protein 29 OS=Homo sapiens GN=ARHGAP29 PE=1 SV=2 - [RHG29_HUMAN]	2.78	1	3	3	9	19.67	21.55	21.36		18.36	19.65	3	2	19.83	21.62	21.42		18.58	19.39	20.96	18.98	0.088	0.045	-1.975
Q9C0B5	Palmitoyltransferase ZDHHC5 OS=Homo sapiens GN=ZDHHC5 PE=1 SV=2 - [ZDHC5_HUMAN]	20.00	1	9	9	32	24.35	24.08	24.23	23.98	21.80	23.45	3	3	24.51	24.15	24.28	23.73	22.01	23.19	24.31	22.98	0.062	0.035	-1.338
Q03405	Urokinase plasminogen activator surface receptor OS=Homo sapiens GN=PLAUR PE=1 SV=1 - [UPAR_HUMAN]	43.28	1	9	9	73	27.33	27.46	27.74	26.94	26.28	27.53	3	3	27.49	27.53	27.80	26.69	26.49	27.27	27.61	26.82	0.035	0.024	-0.787
P19823	Inter-alpha-trypsin inhibitor heavy chain H2 OS=Homo sapiens GN=ITIH2 PE=1 SV=2 - [ITIH2_HUMAN]	6.13	1	5	5	30	26.36	24.89	25.26	26.40	25.75	26.61	3	3	26.52	24.96	25.32	26.15	25.97	26.34	25.60	26.16	0.315	0.117	0.556
P02794	Ferritin heavy chain OS=Homo sapiens GN=FTH1 PE=1 SV=2 - [FRIH_HUMAN]	66.67	1	11	11	50	24.68	24.32	24.21	26.34	25.87	24.19	3	3	24.84	24.39	24.27	26.10	26.09	23.93	24.50	25.37	0.304	0.113	0.874
O14787	Transportin-2 OS=Homo sapiens GN=TNPO2 PE=1 SV=3 - [TNPO2_HUMAN]	7.69	1	1	4	14	22.96	23.21	24.00	23.93	23.22	25.34	3	3	23.12	23.28	24.06	23.68	23.44	25.08	23.48	24.07	0.379	0.134	0.581
Q14315	Filamin-C OS=Homo sapiens GN=FLNC PE=1 SV=3 - [FLNC_HUMAN]	28.04	1	43	55	191	25.49	25.61	25.66	26.12	25.60	26.31	3	3	25.66	25.68	25.72	25.88	25.81	26.05	25.68	25.92	0.036	0.024	0.230
O14786	Neuropilin-1 OS=Homo sapiens GN=NRP1 PE=1 SV=3 - [NRP1_HUMAN]	42.25	3	26	26	127	26.27	26.51	26.69	25.98	24.34	25.87	3	3	26.43	26.58	26.75	25.73	24.56	25.61	26.59	25.30	0.028	0.021	-1.288
O75347	Tubulin-specific chaperone A OS=Homo sapiens GN=TBCA PE=1 SV=3 - [TBCA_HUMAN]	52.78	1	7	7	26	21.67	23.34	23.08	23.87	23.42	24.38	3	3	21.84	23.41	23.14	23.62	23.64	24.12	22.79	23.79	0.123	0.058	0.998
Q96S97	Myeloid-associated differentiation marker OS=Homo sapiens GN=MYADM PE=1 SV=2 - [MYADM_HUMAN]	10.25	1	2	2	15	24.88	25.97	25.36	24.80	24.14	22.46	3	3	25.04	26.04	25.42	24.55	24.36	22.20	25.50	23.70	0.091	0.047	-1.798
Q13263	Transcription intermediary factor 1-beta OS=Homo sapiens GN=TRIM28 PE=1 SV=5 - [TIF1B_HUMAN]	28.14	2	14	14	32	22.73	22.52		24.02	24.97	24.55	2	3	22.90	22.59		23.77	25.18	24.29	22.74	24.41	0.055	0.032	1.670
O75955	Flotillin-1 OS=Homo sapiens GN=FLOT1 PE=1 SV=3 - [FLOT1_HUMAN]	46.37	1	15	15	60	24.05	23.43	23.06	24.35	23.73	24.32	3	3	24.21	23.50	23.12	24.10	23.95	24.05	23.61	24.03	0.261	0.101	0.426
O14744	Protein arginine N-methyltransferase 5 OS=Homo sapiens GN=PRMT5 PE=1 SV=4 - [ANM5_HUMAN]	31.24	1	14	14	41	23.52	23.64	23.76	24.13	23.77	25.17	3	3	23.68	23.71	23.82	23.89	23.99	24.91	23.74	24.26	0.185	0.079	0.526
P40121	Macrophage-capping protein OS=Homo sapiens GN=CAPG PE=1 SV=2 - [CAPG_HUMAN]	62.07	1	14	14	80	25.11	25.53	25.60	25.89	25.52	26.26	3	3	25.27	25.60	25.66	25.64	25.73	26.00	25.51	25.79	0.159	0.070	0.283
Q9Y285	Phenylalanine--tRNA ligase alpha subunit OS=Homo sapiens GN=FARSA PE=1 SV=3 - [SYFA_HUMAN]	30.51	1	9	9	29	23.20	21.80	22.52	23.16	23.93	24.32	3	3	23.36	21.87	22.58	22.91	24.15	24.06	22.60	23.71	0.133	0.061	1.103
Q96C90	Protein phosphatase 1 regulatory subunit 14B OS=Homo sapiens GN=PPP1R14B PE=1 SV=3 - [PP14B_HUMAN]	30.61	1	2	2	13	23.37	24.60	24.47	25.16	24.69	25.41	3	3	23.53	24.67	24.53	24.91	24.91	25.15	24.24	24.99	0.112	0.054	0.748
Q14257	Reticulocalbin-2 OS=Homo sapiens GN=RCN2 PE=1 SV=1 - [RCN2_HUMAN]	28.39	1	5	5	17	22.87	23.47	21.25	23.30	24.71	24.62	3	3	23.03	23.53	21.30	23.06	24.93	24.36	22.62	24.12	0.163	0.072	1.491
P00491	Purine nucleoside phosphorylase OS=Homo sapiens GN=PNP PE=1 SV=2 - [PNPH_HUMAN]	54.33	1	11	11	62	24.38	24.60	24.31	25.53	24.58	25.82	3	3	24.55	24.67	24.37	25.29	24.80	25.56	24.53	25.22	0.045	0.028	0.686
P30154	Serine/threonine-protein phosphatase 2A 65 kDa regulatory subunit A beta isoform OS=Homo sapiens GN=PPP2R1B PE=1 SV=3 - [2AAB_HUMAN]	14.64	1	1	6	32	23.91	23.70	23.09	24.68	23.93	25.10	3	3	24.07	23.77	23.15	24.43	24.15	24.84	23.66	24.47	0.074	0.040	0.811
P48729	Casein kinase I isoform alpha OS=Homo sapiens GN=CSNK1A1 PE=1 SV=2 - [KC1A_HUMAN]	37.98	2	11	11	43	24.66	25.62	25.93	22.18		21.13	3	2	24.83	25.69	25.99	21.93		20.86	25.50	21.40	0.006	0.008	-4.102
O95466	Formin-like protein 1 OS=Homo sapiens GN=FMNL1 PE=1 SV=3 - [FMNL1_HUMAN]	29.45	1	25	27	93	24.76	24.35	24.69	24.44	23.26	24.49	3	3	24.92	24.42	24.75	24.20	23.48	24.23	24.70	23.97	0.064	0.035	-0.729
P08727	Keratin, type I cytoskeletal 19 OS=Homo sapiens GN=KRT19 PE=1 SV=4 - [K1C19_HUMAN]	24.25	10	4	9	32	25.90	24.17	24.43	25.92	25.13	25.03	3	3	26.06	24.24	24.49	25.67	25.34	24.77	24.93	25.26	0.626	0.202	0.331
O43865	Adenosylhomocysteinase 2 OS=Homo sapiens GN=AHCYL1 PE=1 SV=2 - [SAHH2_HUMAN]	31.89	2	10	11	34	22.02	22.64	22.97	23.56	22.66	23.94	3	3	22.18	22.71	23.03	23.31	22.88	23.68	22.64	23.29	0.126	0.059	0.649
Q9UBP0	Spastin OS=Homo sapiens GN=SPAST PE=1 SV=1 - [SPAST_HUMAN]	24.03	1	11	11	24	23.92	24.07	23.89	23.04	23.84	23.70	3	3	24.09	24.14	23.95	22.79	24.06	23.44	24.06	23.43	0.165	0.072	-0.628
P57721	Poly(rC)-binding protein 3 OS=Homo sapiens GN=PCBP3 PE=2 SV=2 - [PCBP3_HUMAN]	18.33	2	1	6	33	26.37	26.21	26.33	26.71	26.32	27.23	3	3	26.53	26.28	26.39	26.46	26.53	26.96	26.40	26.65	0.221	0.090	0.251
Q9UL42	Paraneoplastic antigen Ma2 OS=Homo sapiens GN=PNMA2 PE=1 SV=2 - [PNMA2_HUMAN]	9.62	1	2	2	8	20.98	21.21	21.09	20.30	19.90		3	2	21.14	21.28	21.15	20.06	20.12		21.19	20.09	0.000	0.002	-1.098
P42766	60S ribosomal protein L35 OS=Homo sapiens GN=RPL35 PE=1 SV=2 - [RL35_HUMAN]	32.52	1	4	5	18	25.02		25.72	26.62	26.24	26.54	2	3	25.19		25.78	26.37	26.46	26.28	25.48	26.37	0.031	0.022	0.889
CON_007161211	SWISS-PROT:Q95121 Bos taurus Pigment epithelium-derived factor precursor	53.61	2	15	15	103	26.68	24.82	25.86	26.67	26.43	27.00	3	3	26.84	24.89	25.92	26.42	26.64	26.74	25.88	26.60	0.277	0.105	0.718
Q01844	RNA-binding protein EWS OS=Homo sapiens GN=EWSR1 PE=1 SV=1 - [EWS_HUMAN]	3.51	1	2	2	6		21.53	20.77	24.62	23.45	24.50	2	3		21.60	20.83	24.37	23.66	24.24	21.22	24.09	0.006	0.007	2.876
Q92626	Peroxidasin homolog OS=Homo sapiens GN=PXDN PE=1 SV=2 - [PXDN_HUMAN]	18.32	1	18	18	42	23.88	23.33	23.25	24.60	23.38	23.58	3	3	24.04	23.40	23.31	24.36	23.59	23.32	23.58	23.76	0.674	0.214	0.175
Q9HAB3	Solute carrier family 52, riboflavin transporter, member 2 OS=Homo sapiens GN=SLC52A2 PE=1 SV=1 - [S52A2_HUMAN]	1.80	1	1	1	6	22.65	23.05	22.58	24.61	22.98	24.70	3	3	22.81	23.12	22.64	24.37	23.19	24.44	22.86	24.00	0.056	0.032	1.144
Q8IXU6	Solute carrier family 35 member F2 OS=Homo sapiens GN=SLC35F2 PE=1 SV=1 - [S35F2_HUMAN]	3.48	1	1	1	5	22.38	23.04	22.56	24.13		23.45	3	2	22.54	23.11	22.62	23.89		23.19	22.75	23.54	0.110	0.053	0.783
P02792	Ferritin light chain OS=Homo sapiens GN=FTL PE=1 SV=2 - [FRIL_HUMAN]	29.71	1	5	5	26	23.88	23.56	22.29	25.57	25.01	22.24	3	3	24.04	23.63	22.35	25.33	25.22	21.98	23.34	24.18	0.529	0.178	0.834
P78368	Casein kinase I isoform gamma-2 OS=Homo sapiens GN=CSNK1G2 PE=1 SV=1 - [KC1G2_HUMAN]	26.27	1	2	10	29	24.21	24.84	25.06	23.95	21.49	23.59	3	3	24.37	24.91	25.12	23.71	21.71	23.33	24.80	22.91	0.045	0.028	-1.885
P01023	Alpha-2-macroglobulin OS=Homo sapiens GN=A2M PE=1 SV=3 - [A2MG_HUMAN]	4.68	1	5	7	61	27.72	26.15	25.83	27.93	27.15	28.21	3	3	27.89	26.22	25.89	27.68	27.36	27.95	26.66	27.67	0.193	0.081	1.002
P04075	Fructose-bisphosphate aldolase A OS=Homo sapiens GN=ALDOA PE=1 SV=2 - [ALDOA_HUMAN]	77.47	2	20	22	169	27.35	27.86	27.77	28.61	27.82	28.84	3	3	27.51	27.93	27.83	28.36	28.03	28.58	27.76	28.32	0.049	0.029	0.566
P60891	Ribose-phosphate pyrophosphokinase 1 OS=Homo sapiens GN=PRPS1 PE=1 SV=2 - [PRPS1_HUMAN]	39.94	1	4	11	48	24.16	24.31	24.05	24.69	24.61	25.88	3	3	24.32	24.38	24.11	24.45	24.83	25.62	24.27	24.96	0.121	0.057	0.694
P49591	Serine--tRNA ligase, cytoplasmic OS=Homo sapiens GN=SARS PE=1 SV=3 - [SYSC_HUMAN]	29.38	1	11	11	36	23.33	23.47	22.43	23.95	23.56	23.76	3	3	23.49	23.54	22.49	23.71	23.77	23.50	23.17	23.66	0.241	0.095	0.487
Q96HE7	ERO1-like protein alpha OS=Homo sapiens GN=ERO1L PE=1 SV=2 - [ERO1A_HUMAN]	41.45	2	14	14	34	22.30	22.14	22.11	22.23	24.67	25.47	3	3	22.46	22.21	22.17	21.98	24.89	25.21	22.28	24.03	0.165	0.073	1.747
Q99567	Nuclear pore complex protein Nup88 OS=Homo sapiens GN=NUP88 PE=1 SV=2 - [NUP88_HUMAN]	1.75	1	1	1	5	20.79		20.70	21.42	21.09	21.95	2	3	20.95		20.76	21.17	21.30	21.69	20.86	21.39	0.086	0.045	0.532
Q5ZPR3	CD276 antigen OS=Homo sapiens GN=CD276 PE=1 SV=1 - [CD276_HUMAN]	31.09	1	8	8	43	25.04	25.20	25.26	24.48	22.88	24.77	3	3	25.20	25.27	25.32	24.23	23.09	24.51	25.27	23.95	0.039	0.025	-1.320
P46934	E3 ubiquitin-protein ligase NEDD4 OS=Homo sapiens GN=NEDD4 PE=1 SV=4 - [NEDD4_HUMAN]	5.46	1	1	5	17	22.44	22.96	22.78	22.34	22.18	22.52	3	3	22.60	23.03	22.84	22.09	22.39	22.26	22.82	22.25	0.019	0.016	-0.577
Q13557	Calcium/calmodulin-dependent protein kinase type II subunit delta OS=Homo sapiens GN=CAMK2D PE=1 SV=3 - [KCC2D_HUMAN]	7.41	1	3	3	10	21.24		21.08	21.98	22.03	22.55	2	3	21.40		21.14	21.74	22.25	22.29	21.27	22.09	0.046	0.028	0.820
Q96FQ6	Protein S100-A16 OS=Homo sapiens GN=S100A16 PE=1 SV=1 - [S10AG_HUMAN]	72.82	1	6	6	37	24.86	25.55	25.43	24.61	23.59	24.75	3	3	25.02	25.62	25.49	24.37	23.81	24.49	25.38	24.22	0.014	0.013	-1.157
O14579	Coatomer subunit epsilon OS=Homo sapiens GN=COPE PE=1 SV=3 - [COPE_HUMAN]	47.40	1	8	8	26	24.05	23.85	23.47	24.65	23.84	24.33	3	3	24.21	23.92	23.53	24.40	24.05	24.07	23.88	24.17	0.275	0.105	0.288
Q99808	Equilibrative nucleoside transporter 1 OS=Homo sapiens GN=SLC29A1 PE=1 SV=3 - [S29A1_HUMAN]	10.96	1	5	5	36	25.88	25.85	26.19	25.37	22.77	25.30	3	3	26.04	25.92	26.25	25.13	22.98	25.04	26.07	24.38	0.076	0.040	-1.685
CON_007135052	SWISS-PROT:Q2UVX4| Fragment	67.55	2	90	90	615	28.29	26.64	27.79	28.25	27.58	28.60	3	3	28.45	26.71	27.85	28.01	27.80	28.34	27.67	28.05	0.517	0.174	0.379
Q8TF05	Serine/threonine-protein phosphatase 4 regulatory subunit 1 OS=Homo sapiens GN=PPP4R1 PE=1 SV=1 - [PP4R1_HUMAN]	3.47	1	2	2	8	21.30		21.03	22.21	22.05	22.85	2	3	21.46		21.09	21.96	22.27	22.59	21.28	22.27	0.035	0.023	0.996
P29323	Ephrin type-B receptor 2 OS=Homo sapiens GN=EPHB2 PE=1 SV=5 - [EPHB2_HUMAN]	18.10	8	12	14	47	25.09	25.16	25.04	24.21	23.89	25.06	3	3	25.25	25.22	25.10	23.97	24.11	24.80	25.19	24.29	0.027	0.020	-0.896
P04843	Dolichyl-diphosphooligosaccharide--protein glycosyltransferase subunit 1 OS=Homo sapiens GN=RPN1 PE=1 SV=1 - [RPN1_HUMAN]	35.58	1	16	16	58	22.95	24.24	24.64	23.55	24.31	24.86	3	3	23.11	24.31	24.70	23.31	24.52	24.60	24.04	24.14	0.879	0.256	0.103
P61956	Small ubiquitin-related modifier 2 OS=Homo sapiens GN=SUMO2 PE=1 SV=3 - [SUMO2_HUMAN]	23.16	3	2	2	15	24.00	25.09	25.18	25.75	25.17	26.00	3	3	24.17	25.16	25.24	25.50	25.39	25.73	24.85	25.54	0.128	0.059	0.688
Q658P3	Metalloreductase STEAP3 OS=Homo sapiens GN=STEAP3 PE=1 SV=2 - [STEA3_HUMAN]	10.66	1	4	4	17	22.41	22.24	21.77	24.88	22.10	24.39	3	3	22.57	22.31	21.83	24.63	22.31	24.13	22.23	23.69	0.119	0.056	1.458
P40925	Malate dehydrogenase, cytoplasmic OS=Homo sapiens GN=MDH1 PE=1 SV=4 - [MDHC_HUMAN]	40.72	1	13	13	60	24.96	25.25	25.04	26.43	25.16	26.62	3	3	25.12	25.32	25.10	26.19	25.38	26.36	25.18	25.98	0.063	0.035	0.792
Q92544	Transmembrane 9 superfamily member 4 OS=Homo sapiens GN=TM9SF4 PE=1 SV=2 - [TM9S4_HUMAN]	11.53	1	5	5	20	22.11	23.55	22.59	24.36	23.49	24.14	3	3	22.28	23.62	22.65	24.11	23.71	23.88	22.85	23.90	0.066	0.036	1.050
Q13418	Integrin-linked protein kinase OS=Homo sapiens GN=ILK PE=1 SV=2 - [ILK_HUMAN]	38.27	1	14	14	55	24.83	24.26	24.43	23.50	22.73	24.51	3	3	24.99	24.33	24.49	23.26	22.94	24.25	24.60	23.48	0.064	0.036	-1.121
P10599	Thioredoxin OS=Homo sapiens GN=TXN PE=1 SV=3 - [THIO_HUMAN]	54.29	1	6	6	34	26.32	27.53	27.45	27.93	27.64	28.12	3	3	26.49	27.60	27.51	27.69	27.86	27.86	27.20	27.80	0.172	0.075	0.602
P27701	CD82 antigen OS=Homo sapiens GN=CD82 PE=1 SV=1 - [CD82_HUMAN]	11.61	1	3	3	14	25.19	26.33	27.16	24.80	24.03	26.56	3	3	25.35	26.40	27.22	24.55	24.24	26.30	26.32	25.03	0.197	0.082	-1.294
O43617	Trafficking protein particle complex subunit 3 OS=Homo sapiens GN=TRAPPC3 PE=1 SV=1 - [TPPC3_HUMAN]	10.00	1	2	2	7		21.23	21.05	22.11	21.73	22.08	2	3		21.30	21.11	21.87	21.95	21.82	21.20	21.88	0.004	0.006	0.677
P06454	Prothymosin alpha OS=Homo sapiens GN=PTMA PE=1 SV=2 - [PTMA_HUMAN]	26.13	1	3	3	30	22.30	22.43	24.59	24.08	24.54	24.47	3	3	22.46	22.50	24.65	23.83	24.75	24.20	23.20	24.26	0.240	0.095	1.062
Q9NPF0	CD320 antigen OS=Homo sapiens GN=CD320 PE=1 SV=1 - [CD320_HUMAN]	6.38	1	2	2	7	22.69	21.87		25.53	23.25	22.33	2	3	22.85	21.94		25.28	23.46	22.07	22.39	23.60	0.403	0.141	1.212
P42892	Endothelin-converting enzyme 1 OS=Homo sapiens GN=ECE1 PE=1 SV=2 - [ECE1_HUMAN]	31.04	1	18	18	85	23.92	24.04	23.78	25.23	23.95	25.41	3	3	24.08	24.11	23.84	24.98	24.17	25.15	24.01	24.77	0.074	0.040	0.757
P35580	Myosin-10 OS=Homo sapiens GN=MYH10 PE=1 SV=3 - [MYH10_HUMAN]	26.11	1	24	39	157	25.29	25.65	25.74	26.20	25.62	27.15	3	3	25.46	25.72	25.79	25.95	25.84	26.89	25.66	26.23	0.179	0.077	0.570
O96013	Serine/threonine-protein kinase PAK 4 OS=Homo sapiens GN=PAK4 PE=1 SV=1 - [PAK4_HUMAN]	20.64	3	7	7	15	22.49	23.87	23.91		22.07	23.05	3	2	22.65	23.94	23.97		22.29	22.79	23.52	22.54	0.195	0.082	-0.984
P23229	Integrin alpha-6 OS=Homo sapiens GN=ITGA6 PE=1 SV=5 - [ITA6_HUMAN]	45.66	1	41	41	243	26.31	26.11	25.86	26.90	26.07	26.52	3	3	26.47	26.18	25.91	26.66	26.28	26.26	26.19	26.40	0.362	0.130	0.212
P50395	Rab GDP dissociation inhibitor beta OS=Homo sapiens GN=GDI2 PE=1 SV=2 - [GDIB_HUMAN]	66.07	1	18	25	205	26.40	26.20	26.12	28.73	26.04	29.16	3	3	26.57	26.27	26.18	28.48	26.26	28.90	26.34	27.88	0.136	0.062	1.541
Q01082	Spectrin beta chain, non-erythrocytic 1 OS=Homo sapiens GN=SPTBN1 PE=1 SV=2 - [SPTB2_HUMAN]	39.21	4	67	68	262	24.34	24.43	24.33	25.17	24.38	25.47	3	3	24.50	24.50	24.38	24.92	24.59	25.21	24.46	24.91	0.070	0.038	0.446
Q15393	Splicing factor 3B subunit 3 OS=Homo sapiens GN=SF3B3 PE=1 SV=4 - [SF3B3_HUMAN]	17.58	1	15	15	36		21.35	23.15	24.15	23.65	24.25	2	3		21.42	23.21	23.90	23.87	23.99	22.31	23.92	0.095	0.048	1.606
Q06481	Amyloid-like protein 2 OS=Homo sapiens GN=APLP2 PE=1 SV=2 - [APLP2_HUMAN]	17.43	1	8	8	29	22.51	23.38		25.10	24.62	24.13	2	3	22.67	23.45		24.86	24.83	23.87	23.06	24.52	0.065	0.036	1.458
Q9NV70	Exocyst complex component 1 OS=Homo sapiens GN=EXOC1 PE=1 SV=4 - [EXOC1_HUMAN]	7.61	1	4	4	11	21.79	22.19		21.89	22.41	22.45	2	3	21.95	22.26		21.65	22.63	22.19	22.10	22.15	0.902	0.261	0.051

P62330	ADP-ribosylation factor 6 OS=Homo sapiens GN=ARF6 PE=1 SV=2 - [ARF6_HUMAN]	84.00	4	10	12	94	26.55	26.69	26.76	26.29	25.29	26.44	3	3	26.71	26.76	26.82	26.05	25.50	26.18	26.76	25.91	0.015	0.014	-0.853
P55735	Protein SEC13 homolog OS=Homo sapiens GN=SEC13 PE=1 SV=3 - [SEC13_HUMAN]	31.68	1	6	6	27	24.65	24.33	23.77	24.87	24.89	25.61	3	3	24.81	24.40	23.83	24.63	25.11	25.35	24.35	25.03	0.127	0.059	0.682
P20742	Pregnancy zone protein OS=Homo sapiens GN=PZP PE=1 SV=4 - [PZP_HUMAN]	2.29	1	1	3	37	27.81	26.16	25.99	27.83	27.25	28.16	3	3	27.97	26.23	26.05	27.58	27.47	27.90	26.75	27.65	0.222	0.090	0.904
O75874	Isocitrate dehydrogenase [NADP] cytoplasmic OS=Homo sapiens GN=IDH1 PE=1 SV=2 - [IDHC_HUMAN]	36.23	2	11	11	52	24.21	24.30	24.19	24.86	24.29	24.56	3	3	24.37	24.37	24.25	24.62	24.51	24.30	24.33	24.48	0.220	0.090	0.147
P63104	14-3-3 protein zeta/delta OS=Homo sapiens GN=YWHAZ PE=1 SV=1 - [1433Z_HUMAN]	71.43	1	17	19	173	27.97	28.60	28.58	29.54	28.53	29.68	3	3	28.14	28.67	28.64	29.30	28.75	29.41	28.48	29.15	0.069	0.037	0.668
Q14974	Importin subunit beta-1 OS=Homo sapiens GN=KPNB1 PE=1 SV=2 - [IMB1_HUMAN]	56.96	1	34	34	200	25.42	25.19	25.47	26.34	25.11	26.46	3	3	25.58	25.26	25.52	26.09	25.33	26.20	25.46	25.87	0.228	0.092	0.417
O43291	Kunitz-type protease inhibitor 2 OS=Homo sapiens GN=SPINT2 PE=1 SV=2 - [SPIT2_HUMAN]	13.49	1	4	4	8	21.96	21.95	21.61	23.27		22.23	3	2	22.12	22.02	21.67	23.02		21.97	21.94	22.50	0.285	0.108	0.559
Q9Y371	Endophilin-B1 OS=Homo sapiens GN=SH3GLB1 PE=1 SV=1 - [SHLB1_HUMAN]	13.70	1	5	5	9	22.08		21.47	22.87	22.30	23.14	2	3	22.24		21.53	22.62	22.52	22.88	21.89	22.67	0.079	0.042	0.785
P54920	Alpha-soluble NSF attachment protein OS=Homo sapiens GN=NAPA PE=1 SV=3 - [SNAA_HUMAN]	66.78	2	15	15	44	23.63	23.82	22.78	24.74	23.80	24.84	3	3	23.79	23.89	22.84	24.50	24.02	24.58	23.51	24.37	0.085	0.044	0.859
Q92542	Nicastrin OS=Homo sapiens GN=NCSTN PE=1 SV=2 - [NICA_HUMAN]	17.63	1	10	10	34	23.42	24.45	24.17	26.00	25.52	24.28	3	3	23.58	24.52	24.22	25.76	25.74	24.02	24.11	25.17	0.173	0.075	1.060
Q15121	Astrocytic phosphoprotein PEA-15 OS=Homo sapiens GN=PEA15 PE=1 SV=2 - [PEA15_HUMAN]	38.46	1	4	4	22	23.80	23.08	23.08	23.73	23.83	23.92	3	3	23.96	23.15	23.14	23.48	24.04	23.66	23.42	23.73	0.383	0.136	0.313
P54764	Ephrin type-A receptor 4 OS=Homo sapiens GN=EPHA4 PE=1 SV=1 - [EPHA4_HUMAN]	2.43	1	1	2	7	25.59	25.93	25.65	24.59	23.89	25.64	3	3	25.75	26.00	25.71	24.35	24.11	25.38	25.82	24.61	0.039	0.025	-1.208
O14578	Citron Rho-interacting kinase OS=Homo sapiens GN=CIT PE=1 SV=2 - [CTRO_HUMAN]	11.30	1	14	14	31	21.97	21.01	22.57	21.53	20.96	21.45	3	3	22.14	21.08	22.63	21.28	21.17	21.19	21.95	21.21	0.184	0.079	-0.736
P19784	Casein kinase II subunit alpha' OS=Homo sapiens GN=CSNK2A2 PE=1 SV=1 - [CSK22_HUMAN]	21.43	3	4	6	19	23.29	23.19	22.96	24.37	23.20	25.18	3	3	23.46	23.26	23.02	24.13	23.42	24.92	23.25	24.15	0.115	0.055	0.907
P40939	Trifunctional enzyme subunit alpha, mitochondrial OS=Homo sapiens GN=HADHA PE=1 SV=2 - [ECHA_HUMAN]	28.44	1	14	14	32		23.92	23.57	22.02	24.45	24.72	2	3		23.99	23.63	21.78	24.67	24.46	23.81	23.63	0.894	0.259	-0.175
P56199	Integrin alpha-1 OS=Homo sapiens GN=ITGA1 PE=1 SV=2 - [ITA1_HUMAN]	25.19	1	20	21	81	23.43	23.69	23.71	24.88	23.54	25.35	3	3	23.60	23.76	23.77	24.63	23.75	25.09	23.71	24.49	0.118	0.056	0.783
Q9NY35	Claudin domain-containing protein 1 OS=Homo sapiens GN=CLDND1 PE=1 SV=1 - [CLDN1_HUMAN]	17.00	1	4	4	16	25.02	25.04	25.12	24.85	23.41	24.65	3	3	25.18	25.11	25.18	24.60	23.63	24.39	25.16	24.21	0.032	0.022	-0.955
Q8IVI9	Nostrin OS=Homo sapiens GN=NOSTRIN PE=1 SV=2 - [NOSTN_HUMAN]	1.58	1	1	1	6	24.37	25.54	25.31	26.49	25.53	26.36	3	3	24.53	25.61	25.37	26.25	25.75	26.10	25.17	26.03	0.075	0.040	0.859
P00367	Glutamate dehydrogenase 1, mitochondrial OS=Homo sapiens GN=GLUD1 PE=1 SV=2 - [DHE3_HUMAN]	42.11	3	18	18	46		23.16	24.35	23.25	25.32	25.32	2	3		23.23	24.40	23.00	25.54	25.06	23.82	24.53	0.559	0.185	0.718
P11908	Ribose-phosphate pyrophosphokinase 2 OS=Homo sapiens GN=PRPS2 PE=1 SV=2 - [PRPS2_HUMAN]	35.53	1	2	9	38	24.16	24.31	24.05	24.49	24.67	25.88	3	3	24.32	24.38	24.11	24.25	24.88	25.62	24.27	24.92	0.185	0.079	0.645
Q14444	Caprin-1 OS=Homo sapiens GN=CAPRIN1 PE=1 SV=2 - [CAPR1_HUMAN]	14.95	1	10	10	26		22.59	20.73	24.40	24.32	24.57	2	3		22.66	20.79	24.16	24.53	24.31	21.72	24.33	0.035	0.023	2.611
Q13641	Trophoblast glycoprotein OS=Homo sapiens GN=TPBG PE=1 SV=1 - [TPBG_HUMAN]	21.43	1	6	6	33	24.11	24.47	24.65	25.60	24.39	25.58	3	3	24.27	24.54	24.71	25.36	24.60	25.31	24.51	25.09	0.101	0.051	0.585
Q9NZ01	Very-long-chain enoyl-CoA reductase OS=Homo sapiens GN=TECR PE=1 SV=1 - [TECR_HUMAN]	12.01	1	5	5	12	22.63	22.96	22.91		23.52	24.19	3	2	22.79	23.03	22.97		23.74	23.92	22.93	23.83	0.004	0.006	0.902
P31150	Rab GDP dissociation inhibitor alpha OS=Homo sapiens GN=GDI1 PE=1 SV=2 - [GDIA_HUMAN]	57.27	1	12	19	91	25.91	25.70	25.58	28.64	25.38	29.06	3	3	26.07	25.77	25.64	28.39	25.60	28.80	25.83	27.60	0.156	0.069	1.771
P20908	Collagen alpha-1(V) chain OS=Homo sapiens GN=COL5A1 PE=1 SV=3 - [CO5A1_HUMAN]	13.11	1	16	16	69	26.02	25.71	26.26	25.85	25.13	24.36	3	3	26.18	25.78	26.32	25.60	25.35	24.10	26.09	25.01	0.094	0.048	-1.078
Q9Y6W5	Wiskott-Aldrich syndrome protein family member 2 OS=Homo sapiens GN=WASF2 PE=1 SV=3 - [WASF2_HUMAN]	10.24	2	3	3	13	22.47	22.60	22.37	22.63	22.63	22.84	3	3	22.63	22.67	22.43	22.38	22.85	22.58	22.58	22.60	0.868	0.254	0.027
Q9P2B2	Prostaglandin F2 receptor negative regulator OS=Homo sapiens GN=PTGFRN PE=1 SV=2 - [FPRP_HUMAN]	23.66	1	16	16	41	21.10		23.17	24.78	23.34	24.80	2	3	21.26		23.23	24.54	23.56	24.54	22.24	24.21	0.102	0.051	1.967
Q9NVJ2	ADP-ribosylation factor-like protein 8B OS=Homo sapiens GN=ARL8B PE=1 SV=1 - [ARL8B_HUMAN]	47.31	2	6	6	30	23.71	24.02	23.74	24.61	23.90	24.76	3	3	23.87	24.09	23.80	24.36	24.12	24.50	23.92	24.33	0.044	0.027	0.408
P09960	Leukotriene A-4 hydrolase OS=Homo sapiens GN=LTA4H PE=1 SV=2 - [LKHA4_HUMAN]	33.22	1	14	14	40	23.27	22.97	22.96	24.17	22.88	24.37	3	3	23.43	23.04	23.02	23.93	23.09	24.11	23.16	23.71	0.184	0.079	0.545
P61086	Ubiquitin-conjugating enzyme E2 K OS=Homo sapiens GN=UBE2K PE=1 SV=3 - [UBE2K_HUMAN]	59.50	2	7	7	22	24.12	24.86	24.93	24.21	23.11	23.94	3	3	24.28	24.93	24.99	23.96	23.33	23.68	24.73	23.66	0.021	0.017	-1.077
P07947	Tyrosine-protein kinase Yes OS=Homo sapiens GN=YES1 PE=1 SV=3 - [YES_HUMAN]	42.17	21	11	17	75	25.45	25.29	25.35	25.01	23.67	25.19	3	3	25.62	25.36	25.41	24.77	23.89	24.93	25.46	24.53	0.048	0.029	-0.934
Q16401	26S proteasome non-ATPase regulatory subunit 5 OS=Homo sapiens GN=PSMD5 PE=1 SV=3 - [PSMD5_HUMAN]	10.71	1	3	3	16	21.62	21.59	21.39	22.58	21.47	22.74	3	3	21.78	21.66	21.45	22.33	21.68	22.48	21.63	22.17	0.112	0.054	0.536
O00410	Importin-5 OS=Homo sapiens GN=IPO5 PE=1 SV=4 - [IPO5_HUMAN]	37.56	2	23	23	72	23.42	23.44	23.63	24.42	23.31	24.91	3	3	23.58	23.51	23.69	24.17	23.52	24.64	23.60	24.11	0.190	0.080	0.518
Q8TEX9	Importin-4 OS=Homo sapiens GN=IPO4 PE=1 SV=2 - [IPO4_HUMAN]	10.36	1	6	6	17	21.82		23.67	23.88	23.45	24.55	2	3	21.99		23.73	23.63	23.67	24.29	22.86	23.86	0.250	0.098	1.006
Q70UQ0	Inhibitor of nuclear factor kappa-B kinase-interacting protein OS=Homo sapiens GN=IKBIP PE=1 SV=1 - [IKIP_HUMAN]	20.57	1	7	7	15		21.70	21.74	23.54	23.70	22.60	2	3		21.77	21.80	23.29	23.92	22.34	21.79	23.18	0.100	0.050	1.396
Q8TCZ2	CD99 antigen-like protein 2 OS=Homo sapiens GN=CD99L2 PE=1 SV=1 - [C99L2_HUMAN]	29.77	1	6	6	24	24.32	24.72	23.19	25.71	24.53	25.28	3	3	24.48	24.79	23.25	25.46	24.75	25.02	24.17	25.08	0.152	0.068	0.906
Q9H3S7	Tyrosine-protein phosphatase non-receptor type 23 OS=Homo sapiens GN=PTPN23 PE=1 SV=1 - [PTN23_HUMAN]	9.29	1	12	12	31	22.59	22.38	22.40	22.20	21.46	22.41	3	3	22.75	22.45	22.46	21.96	21.68	22.14	22.56	21.93	0.020	0.016	-0.631
O15067	Phosphoribosylformylglycinamidine synthase OS=Homo sapiens GN=PFAS PE=1 SV=4 - [PUR4_HUMAN]	10.84	1	10	10	24	22.30		21.35	23.27	22.27	23.68	2	3	22.46		21.41	23.03	22.49	23.41	21.94	22.98	0.141	0.064	1.039
Q13325	Interferon-induced protein with tetratricopeptide repeats 5 OS=Homo sapiens GN=IFIT5 PE=1 SV=1 - [IFIT5_HUMAN]	4.77	1	2	2	5		20.84	19.86		19.91	19.47	2	2		20.91	19.91		20.13	19.21	20.41	19.67	0.387	0.137	-0.743
CON_006941072	SWISS-PROT:P02584 Bos taurus Profilin-1	60.00	1	2	6	51	28.39	28.43	28.38	29.23	28.30	29.38	3	3	28.55	28.50	28.44	28.98	28.51	29.12	28.50	28.87	0.114	0.054	0.376
Q9Y4G6	Talin-2 OS=Homo sapiens GN=TLN2 PE=1 SV=4 - [TLN2_HUMAN]	6.06	1	2	11	39	24.25	24.64	23.76	25.04	24.59	25.43	3	3	24.41	24.71	23.82	24.79	24.80	25.16	24.31	24.92	0.104	0.051	0.607
Q9BV40	Vesicle-associated membrane protein 8 OS=Homo sapiens GN=VAMP8 PE=1 SV=1 - [VAMP8_HUMAN]	53.00	1	5	5	21	21.51	22.66	22.33	24.39	22.63	23.55	3	3	21.67	22.73	22.39	24.15	22.85	23.29	22.26	23.43	0.077	0.041	1.166
P62937	Peptidyl-prolyl cis-trans isomerase A OS=Homo sapiens GN=PPIA PE=1 SV=2 - [PPIA_HUMAN]	70.30	6	12	12	98	28.97	29.55	29.41	29.64	29.46	29.95	3	3	29.13	29.62	29.47	29.40	29.68	29.69	29.40	29.59	0.348	0.126	0.185
O75340	Programmed cell death protein 6 OS=Homo sapiens GN=PDCD6 PE=1 SV=1 - [PDCD6_HUMAN]	71.20	1	11	11	53	24.66	25.82	25.27	26.56	25.79	26.09	3	3	24.82	25.89	25.33	26.32	26.01	25.83	25.35	26.05	0.106	0.052	0.706
Q9P0V9	Septin-10 OS=Homo sapiens GN=SEPT10 PE=1 SV=2 - [SEP10_HUMAN]	13.00	1	2	3	7	19.98	21.69		22.89	22.90	23.22	2	3	20.15	21.76		22.65	23.11	22.96	20.95	22.91	0.052	0.031	1.957
CON_006883162	SWISS-PROT:P41361 Bos taurus Antithrombin-III precursor	21.29	2	8	8	18	23.61	22.75	22.25	23.69	23.35	24.10	3	3	23.77	22.82	22.31	23.45	23.57	23.84	22.97	23.62	0.218	0.089	0.651
Q9NR50	Translation initiation factor eIF-2B subunit gamma OS=Homo sapiens GN=EIF2B3 PE=1 SV=1 - [EI2BG_HUMAN]	14.82	1	5	5	14	21.09		20.93	22.18	23.20	23.56	2	3	21.25		20.99	21.94	23.41	23.29	21.12	22.88	0.066	0.036	1.756
P08648	Integrin alpha-5 OS=Homo sapiens GN=ITGA5 PE=1 SV=2 - [ITA5_HUMAN]	27.55	1	17	17	97	26.47	26.50	26.43	25.98	25.26	26.43	3	3	26.63	26.57	26.49	25.73	25.48	26.17	26.57	25.79	0.020	0.016	-0.772
CON_006965071	SWISS-PROT:P00978 Bos taurus AMBP protein precursor	31.25	2	9	9	39	26.08	23.72	25.02	25.83	25.27	26.59	3	3	26.24	23.79	25.08	25.59	25.48	26.32	25.04	25.80	0.370	0.132	0.762
Q68D91	Metallo-beta-lactamase domain-containing protein 2 OS=Homo sapiens GN=MBLAC2 PE=1 SV=3 - [MBLC2_HUMAN]	34.05	1	7	7	18	23.39	23.48	23.03	22.61	21.59		3	2	23.55	23.55	23.09	22.37	21.81		23.40	22.09	0.020	0.016	-1.308
P60903	Protein S100-A10 OS=Homo sapiens GN=S100A10 PE=1 SV=2 - [S10AA_HUMAN]	36.08	1	4	4	66	27.08	28.17	28.13	28.01	28.15	28.53	3	3	27.24	28.24	28.19	27.77	28.36	28.27	27.89	28.13	0.551	0.183	0.243
P11166	Solute carrier family 2, facilitated glucose transporter member 1 OS=Homo sapiens GN=SLC2A1 PE=1 SV=2 - [GTR1_HUMAN]	22.97	1	12	13	103	27.93	27.96	27.87	27.65	25.91	27.47	3	3	28.09	28.03	27.93	27.41	26.13	27.21	28.02	26.92	0.052	0.030	-1.101
O75695	Protein XRP2 OS=Homo sapiens GN=RP2 PE=1 SV=4 - [XRP2_HUMAN]	26.29	1	9	9	27	24.59	24.89	24.16	23.93	22.33	23.95	3	3	24.75	24.96	24.22	23.68	22.55	23.69	24.64	23.31	0.038	0.025	-1.335
P30501	HLA class I histocompatibility antigen, Cw-2 alpha chain OS=Homo sapiens GN=HLA-C PE=1 SV=1 - [1C02_HUMAN]	24.59	2	1	7	47	27.35	27.12	27.29	28.29	26.96	28.41	3	3	27.51	27.19	27.35	28.04	27.18	28.15	27.35	27.79	0.245	0.096	0.437
P16615	Sarcoplasmic/endoplasmic reticulum calcium ATPase 2 OS=Homo sapiens GN=ATP2A2 PE=1 SV=1 - [AT2A2_HUMAN]	29.17	3	21	21	66	23.53	24.65	24.43	24.46	24.48	25.57	3	3	23.69	24.72	24.49	24.22	24.70	25.31	24.30	24.74	0.373	0.133	0.444
O75146	Huntingtin-interacting protein 1-related protein OS=Homo sapiens GN=HIP1R PE=1 SV=2 - [HIP1R_HUMAN]	2.06	1	2	2	6	18.89	18.93		20.39	19.54	20.57	2	3	19.05	19.00		20.15	19.75	20.30	19.03	20.07	0.016	0.014	1.042
P31949	Protein S100-A11 OS=Homo sapiens GN=S100A11 PE=1 SV=2 - [S10AB_HUMAN]	80.00	1	6	6	58	27.42	28.31	27.83	28.38	28.19	28.53	3	3	27.58	28.38	27.89	28.13	28.40	28.27	27.95	28.27	0.265	0.102	0.317
P37108	Signal recognition particle 14 kDa protein OS=Homo sapiens GN=SRP14 PE=1 SV=2 - [SRP14_HUMAN]	66.91	1	8	8	22	24.96	27.18	26.77	25.27	25.03	25.06	3	3	25.13	27.25	26.83	25.02	25.25	24.80	26.40	25.02	0.106	0.052	-1.378
P00533	Epidermal growth factor receptor OS=Homo sapiens GN=EGFR PE=1 SV=2 - [EGFR_HUMAN]	36.53	3	34	34	228	25.99	26.28	26.29	27.07	26.11	27.24	3	3	26.15	26.35	26.35	26.83	26.33	26.98	26.28	26.71	0.107	0.052	0.428
P22314	Ubiquitin-like modifier-activating enzyme 1 OS=Homo sapiens GN=UBA1 PE=1 SV=3 - [UBA1_HUMAN]	35.63	1	22	22	125	24.72	24.65	24.69	25.24	24.56	25.29	3	3	24.88	24.72	24.75	24.99	24.77	25.03	24.78	24.93	0.187	0.079	0.150
P34910	Protein EVI2B OS=Homo sapiens GN=EVI2B PE=1 SV=2 - [EVI2B_HUMAN]	11.16	1	3	3	12	24.61	24.06	23.56	25.27	24.03	25.15	3	3	24.77	24.13	23.61	25.02	24.25	24.89	24.17	24.72	0.256	0.099	0.546
P21964	Catechol O-methyltransferase OS=Homo sapiens GN=COMT PE=1 SV=2 - [COMT_HUMAN]	52.40	1	8	8	32	23.92	23.88	27.25	22.90	23.86	24.40	3	3	24.08	23.95	27.31	22.66	24.08	24.14	25.11	23.62	0.282	0.107	-1.489
Q9Y3E5	Peptidyl-tRNA hydrolase 2, mitochondrial OS=Homo sapiens GN=PTRH2 PE=1 SV=1 - [PTH2_HUMAN]	50.84	1	6	6	29		22.31	20.84	24.36	23.65	24.21	2	3		22.38	20.90	24.12	23.86	23.95	21.64	23.98	0.025	0.019	2.338
Q9BZG1	Ras-related protein Rab-34 OS=Homo sapiens GN=RAB34 PE=1 SV=1 - [RAB34_HUMAN]	37.84	1	8	8	26	24.25	24.24	24.22	24.09	23.02	24.00	3	3	24.41	24.31	24.28	23.85	23.23	23.74	24.33	23.61	0.020	0.016	-0.727
P57678	Gem-associated protein 4 OS=Homo sapiens GN=GEMIN4 PE=1 SV=2 - [GEMI4_HUMAN]	4.06	2	2	3	10	22.76	22.51		25.19	23.47	23.56	2	3	22.92	22.58		24.95	23.68	23.30	22.75	23.98	0.157	0.070	1.229
Q09160	HLA class I histocompatibility antigen, A-80 alpha chain OS=Homo sapiens GN=HLA-A PE=1 SV=1 - [1A80_HUMAN]	19.45	1	0	6	39	27.30	26.99	27.22	28.10	26.84	28.18	3	3	27.46	27.06	27.28	27.85	27.05	27.92	27.27	27.61	0.321	0.119	0.342
Q14165	Malectin OS=Homo sapiens GN=MLEC PE=1 SV=1 - [MLEC_HUMAN]	22.26	1	4	4	17	21.28	22.63		23.20	23.38	24.45	2	3	21.44	22.70		22.96	23.60	24.19	22.07	23.58	0.104	0.051	1.513
P14324	Farnesyl pyrophosphate synthase OS=Homo sapiens GN=FDPS PE=1 SV=4 - [FPPS_HUMAN]	15.75	1	6	6	31	25.42	25.33	25.41	25.12	24.13	25.33	3	3	25.58	25.40	25.47	24.87	24.34	25.07	25.49	24.76	0.032	0.022	-0.723
O43169	Cytochrome b5 type B OS=Homo sapiens GN=CYB5B PE=1 SV=2 - [CYB5B_HUMAN]	37.67	1	3	3	19	25.79	26.79	25.17	24.85	26.16	25.29	3	3	25.96	26.86	25.23	24.60	26.37	25.03	26.01	25.33	0.394	0.139	-0.679
P11279	Lysosome-associated membrane glycoprotein 1 OS=Homo sapiens GN=LAMP1 PE=1 SV=3 - [LAMP1_HUMAN]	14.63	1	7	7	39	26.09	27.22	26.68	27.09	27.17	27.14	3	3	26.25	27.29	26.74	26.85	27.38	26.88	26.76	27.04	0.472	0.162	0.275
Q96HC4	PDZ and LIM domain protein 5 OS=Homo sapiens GN=PDLIM5 PE=1 SV=5 - [PDLI5_HUMAN]	17.28	1	8	8	29		22.94	22.24	24.29	23.85	24.60	2	3		23.01	22.30	24.04	24.06	24.34	22.65	24.15	0.014	0.013	1.497
Q9Y5M8	Signal recognition particle receptor subunit beta OS=Homo sapiens GN=SRPRB PE=1 SV=3 - [SRPRB_HUMAN]	23.62	1	5	5	13		21.99	21.98	22.73	22.55	23.43	2	3		22.06	22.04	22.48	22.76	23.17	22.05	22.81	0.061	0.034	0.756
Q00796	Sorbitol dehydrogenase OS=Homo sapiens GN=SORD PE=1 SV=4 - [DHSO_HUMAN]	23.81	1	6	6	10	21.21	21.82		23.83	22.83	24.17	2	3	21.37	21.89		23.58	23.04	23.91	21.63	23.51	0.016	0.014	1.884
P52926	High mobility group protein HMGI-C OS=Homo sapiens GN=HMGA2 PE=1 SV=1 - [HMGA2_HUMAN]	37.61	1	2	2	15		21.10	21.27	22.94	22.64	21.86	2	3		21.16	21.33	22.70	22.85	21.60	21.25	22.38	0.113	0.054	1.135
Q9Y2W1	Thyroid hormone receptor-associated protein 3 OS=Homo sapiens GN=THRAP3 PE=1 SV=2 - [TR150_HUMAN]	9.01	1	5	5	12	20.46	20.08	20.10	20.05	22.08	22.94	3	3	20.62	20.15	20.16	19.81	22.30	22.68	20.31	21.60	0.233	0.093	1.286
O00151	PDZ and LIM domain protein 1 OS=Homo sapiens GN=PDLIM1 PE=1 SV=4 - [PDLI1_HUMAN]	19.45	1	4	4	19	22.26	22.82	23.18	22.69	22.73	23.65	3	3	22.42	22.89	23.24	22.44	22.95	23.39	22.85	22.93	0.845	0.250	0.076
Q6NZI2	Polymerase I and transcript release factor OS=Homo sapiens GN=PTRF PE=1 SV=1 - [PTRF_HUMAN]	34.62	1	9	9	50	24.95	24.69	24.26	25.02	24.96	25.35	3	3	25.11	24.76	24.32	24.77	25.17	25.09	24.73	25.01	0.340	0.124	0.281
Q10567	AP-1 complex subunit beta-1 OS=Homo sapiens GN=AP1B1 PE=1 SV=2 - [AP1B1_HUMAN]	27.08	1	6	19	85	25.12	25.13	25.18	24.53	23.47	25.20	3	3	25.28	25.20	25.24	24.29	23.69	24.94	25.24	24.30	0.061	0.034	-0.934
P80723	Brain acid soluble protein 1 OS=Homo sapiens GN=BASP1 PE=1 SV=2 - [BASP1_HUMAN]	88.99	1	18	18	197	27.18	28.35	28.25	28.85	28.34	28.82	3	3	27.34	28.42	28.31	28.61	28.56	28.56	28.02	28.57	0.184	0.079	0.551
P25445	Tumor necrosis factor receptor superfamily member 6 OS=Homo sapiens GN=FAS PE=1 SV=1 - [TNR6_HUMAN]	3.28	1	1	1	6	20.44	20.48	20.85	20.38	19.54	20.64	3	3	20.60	20.55	20.91	20.13	19.75	20.38	20.69	20.09	0.048	0.029	-0.600
P11234	Ras-related protein Ral-B OS=Homo sapiens GN=RALB PE=1 SV=1 - [RALB_HUMAN]	49.03	1	4	11	78	25.30	25.68	25.51	26.18	25.53	26.20	3	3	25.47	25.75	25.57	25.93	25.75	25.94	25.59	25.87	0.055	0.032	0.280
P30043	Flavin reductase (NADPH) OS=Homo sapiens GN=BLVRB PE=1 SV=3 - [BLVRB_HUMAN]	23.30	1	4	4	13	23.57	22.92	22.68	23.87	22.90	23.52	3	3	23.73	22.99	22.74	23.62	23.12	23.26	23.15	23.33	0.616	0.200	0.181
P11216	Glycogen phosphorylase, brain form OS=Homo sapiens GN=PYGB PE=1 SV=5 - [PYGB_HUMAN]	33.81	1	16	21	67	23.95	23.29	23.52	24.51	23.24	24.98	3	3	24.11	23.36	23.58	24.26	23.46	24.72	23.68	24.15	0.341	0.124	0.466
Q9NX76	CKLF-like MARVEL transmembrane domain-containing protein 6 OS=Homo sapiens GN=CMTM6 PE=1 SV=1 - [CKLF6_HUMAN]	5.46	1	1	1	5	23.11		23.45	24.24	22.58	23.87	2	3	23.27		23.51	24.00	22.80	23.61	23.39	23.47	0.877	0.256	0.079
P11177	Pyruvate dehydrogenase E1 component subunit beta, mitochondrial OS=Homo sapiens GN=PDHB PE=1 SV=3 - [ODPB_HUMAN]	13.93	1	4	4	9		22.38	21.70	22.35	23.02	23.40	2	3		22.45	21.76	22.11	23.23	23.14	22.11	22.83	0.270	0.103	0.719
CON_007107832	SWISS-PROT:P01966 Bos taurus Hemoglobin subunit alpha	78.17	2	4	8	125	31.33	30.02	30.88	31.22	30.85	31.41	3	3	31.50	30.09	30.94	30.98	31.07	31.15	30.84	31.07	0.614	0.199	0.225
P30626	Sorcin OS=Homo sapiens GN=SRI PE=1 SV=1 - [SORCN_HUMAN]	61.11	1	11	11	50	23.40	24.78	24.50	25.07	24.80	25.01	3	3	23.56	24.85	24.56	24.83	25.01	24.74	24.32	24.86	0.248	0.097	0.538
Q8N4C8	Misshapen-like kinase 1 OS=Homo sapiens GN=MINK1 PE=1 SV=2 - [MINK1_HUMAN]	5.78	1	3	8	15	22.32	21.55	21.57	21.54	19.03	21.58	3	3	22.48	21.62	21.62	21.29	19.24	21.31	21.91	20.62	0.157	0.070	-1.293
P61225	Ras-related protein Rap-2b OS=Homo sapiens GN=RAP2B PE=1 SV=1 - [RAP2B_HUMAN]	62.84	1	5	9	62	26.04	25.86	25.72	25.66	24.38	25.67	3	3	26.20	25.93	25.78	25.41	24.60	25.41	25.97	25.14	0.049	0.029	-0.831
P35080	Profilin-2 OS=Homo sapiens GN=PFN2 PE=1 SV=3 - [PROF2_HUMAN]	27.86	1	2	2	11	23.69	23.89	23.74	24.02		24.68	3	2	23.85	23.96	23.79	23.78		24.41	23.87	24.10	0.416	0.145	0.231
P60660	Myosin light polypeptide 6 OS=Homo sapiens GN=MYL6 PE=1 SV=2 - [MYL6_HUMAN]	56.95	2	8	8	53	27.74	27.48	27.19	27.66	28.01	28.54	3	3	27.90	27.55	27.25	27.42	28.23	28.28	27.57	27.98	0.289	0.109	0.410
CON_P02676	Fibrinogen beta chain OS=Bos taurus GN=FGB PE=1 SV=2 - [FIBB_BOVIN]	32.48	2	13	13	54	24.41	23.89	24.96	24.87	23.79	25.63	3	3	24.57	23.96	25.02	24.63	24.00	25.37	24.52	24.67	0.787	0.238	0.145
P02533	Keratin, type I cytoskeletal 14 OS=Homo sapiens GN=KRT14 PE=1 SV=4 - [K1C14_HUMAN]	31.78	16	5	15	44	26.45	24.50	24.53	26.40	25.24	24.85	3	3	26.62	24.57	24.59	26.16	25.46	24.59	25.26	25.40	0.871	0.255	0.141
Q9UL25	Ras-related protein Rab-21 OS=Homo sapiens GN=RAB21 PE=1 SV=3 - [RAB21_HUMAN]	52.00	1	10	10	59	24.01	24.89	24.55	23.90	22.22	23.92	3	3	24.17	24.96	24.61	23.65	22.44	23.66	24.58	23.25	0.046	0.028	-1.327
P63165	Small ubiquitin-related modifier 1 OS=Homo sapiens GN=SUMO1 PE=1 SV=1 - [SUMO1_HUMAN]	34.65	1	3	3	12		22.57	21.81	23.80	23.42	24.21	2	3		22.64	21.87	23.55	23.64	23.95	22.26	23.71	0.021	0.017	1.455
Q92692	Nectin-2 OS=Homo sapiens GN=PVRL2 PE=1 SV=1 - [PVRL2_HUMAN]	28.62	1	11	11	47	24.69	25.14	24.66	24.50	23.56	24.44	3	3	24.85	25.21	24.72	24.25	23.77	24.18	24.93	24.07	0.015	0.013	-0.858
P62306	Small nuclear ribonucleoprotein F OS=Homo sapiens GN=SNRPF PE=1 SV=1 - [RUXF_HUMAN]	31.40	1	3	3	12	25.07	24.64	24.81	25.89	24.67	25.00	3	3	25.23	24.71	24.87	25.64	24.88	24.74	24.94	25.09	0.658	0.210	0.153
P46108	Adapter molecule crk OS=Homo sapiens GN=CRK PE=1 SV=2 - [CRK_HUMAN]	16.78	1	4	4	10	21.77		22.91	23.23	23.08	23.42	2	3	21.93		22.97	22.98	23.30	23.16	22.45	23.15	0.183	0.078	0.696
Q5TAT6	Collagen alpha-1(XIII) chain OS=Homo sapiens GN=COL13A1 PE=1 SV=1 - [CODA1_HUMAN]	3.49	1	2	2	7	23.66	23.61		26.47	24.60		2	2	23.82	23.68		26.22	24.82		23.75	25.52	0.130	0.060	1.770
Q9BT67	NEDD4 family-interacting protein 1 OS=Homo sapiens GN=NDFIP1 PE=1 SV=1 - [NFIP1_HUMAN]	7.69	1	1	1	5	22.88	22.95		24.25	23.44	23.79	2	3	23.04	23.02		24.01	23.65	23.53	23.03	23.73	0.033	0.023	0.699
P06132	Uroporphyrinogen decarboxylase OS=Homo sapiens GN=UROD PE=1 SV=2 - [DCUP_HUMAN]	34.88	1	7	7	22	22.51	23.05	22.78	23.58	22.81	23.80	3	3	22.67	23.12	22.84	23.33	23.03	23.53	22.87	23.30	0.097	0.049	0.424
Q15363	Transmembrane emp24 domain-containing protein 2 OS=Homo sapiens GN=TMED2 PE=1 SV=1 - [TMED2_HUMAN]	33.83	1	6	6	37	25.32	24.42	24.12	25.36	24.86	25.83	3	3	25.48	24.49	24.18	25.11	25.08	25.57	24.72	25.25	0.274	0.104	0.537
Q96PY5	Formin-like protein 2 OS=Homo sapiens GN=FMNL2 PE=1 SV=3 - [FMNL2_HUMAN]	9.58	2	7	9	18	22.66	22.21	22.17	23.15		23.57	3	2	22.82	22.28	22.23	22.90		23.31	22.44	23.11	0.105	0.052	0.664
Q9H4A6	Golgi phosphoprotein 3 OS=Homo sapiens GN=GOLPH3 PE=1 SV=1 - [GOLP3_HUMAN]	12.42	2	2	2	8	22.28	21.42	21.57	22.35	21.53	22.30	3	3	22.45	21.49	21.63	22.11	21.75	22.03	21.85	21.96	0.749	0.230	0.109
O60749	Sorting nexin-2 OS=Homo sapiens GN=SNX2 PE=1 SV=2 - [SNX2_HUMAN]	8.29	1	3	4	8	21.90		21.95	22.27	21.85	23.00	2	3	22.07		22.01	22.02	22.07	22.74	22.04	22.28	0.481	0.165	0.241
P05783	Keratin, type I cytoskeletal 18 OS=Homo sapiens GN=KRT18 PE=1 SV=2 - [K1C18_HUMAN]	51.16	9	11	20	59	25.53	24.28	24.09	25.23	25.47	25.42	3	3	25.69	24.35	24.15	24.99	25.69	25.16	24.73	25.28	0.357	0.129	0.550
Q9Y6E2	Basic leucine zipper and W2 domain-containing protein 2 OS=Homo sapiens GN=BZW2 PE=1 SV=1 - [BZW2_HUMAN]	10.74	1	1	4	12	22.88	21.93	22.81	22.69	23.38	23.58	3	3	23.04	22.00	22.87	22.44	23.59	23.32	22.64	23.12	0.366	0.131	0.482
P53680	AP-2 complex subunit sigma OS=Homo sapiens GN=AP2S1 PE=1 SV=2 - [AP2S1_HUMAN]	18.31	1	3	3	9	24.50	25.33	25.16	24.22	22.90	24.85	3	3	24.66	25.40	25.22	23.98	23.11	24.59	25.10	23.89	0.067	0.037	-1.204
P04632	Calpain small subunit 1 OS=Homo sapiens GN=CAPNS1 PE=1 SV=1 - [CPNS1_HUMAN]	58.21	2	12	12	77	24.75	25.28	25.05	25.11	25.21	25.94	3	3	24.91	25.35	25.11	24.86	25.42	25.68	25.12	25.32	0.503	0.171	0.201
P18827	Syndecan-1 OS=Homo sapiens GN=SDC1 PE=1 SV=3 - [SDC1_HUMAN]	16.13	1	4	4	18	26.19	25.47	25.60		22.95	24.76	3	2	26.35	25.54	25.66		23.16	24.50	25.85	23.83	0.043	0.027	-2.016
CON_IPI003114935	SWISS-PROT:P05784 Tax_Id=10090 Gene_Symbol=Krt18 Keratin, type I cytoskeletal 18	30.73	9	2	12	48	26.41	24.09	24.43	26.01	25.84	25.89	3	3	26.57	24.16	24.49	25.77	26.06	25.62	25.07	25.82	0.385	0.136	0.746
Q92597	Protein NDRG1 OS=Homo sapiens GN=NDRG1 PE=1 SV=1 - [NDRG1_HUMAN]	70.81	1	13	13	93	27.09	27.24	26.36	26.15	25.80	26.62	3	3	27.25	27.31	26.42	25.90	26.01	26.36	26.99	26.09	0.048	0.029	-0.902
O14763	Tumor necrosis factor receptor superfamily member 10B OS=Homo sapiens GN=TNFRSF10B PE=1 SV=2 - [TR10B_HUMAN]	26.14	1	9	9	36	23.89	23.76	23.31	24.77	23.52	24.35	3	3	24.05	23.82	23.37	24.53	23.74	24.09	23.75	24.12	0.293	0.110	0.369
P54725	UV excision repair protein RAD23 homolog A OS=Homo sapiens GN=RAD23A PE=1 SV=1 - [RD23A_HUMAN]	23.69	1	6	7	30	24.25	25.26	25.37	24.43	23.98	24.71	3	3	24.41	25.33	25.43	24.18	24.19	24.45	25.06	24.28	0.081	0.043	-0.783
P35659	Protein DEK OS=Homo sapiens GN=DEK PE=1 SV=1 - [DEK_HUMAN]	34.13	1	11	11	36		21.10	21.33	24.58	23.85	22.23	2	3		21.17	21.39	24.34	24.06	21.97	21.28	23.45	0.111	0.054	2.174
CON_007161581	SWISS-PROT:Q05443 Bos taurus Lumican precursor	44.44	2	11	11	65	25.72	22.54	24.18	25.31	25.03	25.61	3	3	25.88	22.61	24.24	25.06	25.24	25.35	24.24	25.22	0.360	0.130	0.978
Q9C0H2	Protein tweety homolog 3 OS=Homo sapiens GN=TTYH3 PE=1 SV=3 - [TTYH3_HUMAN]	7.27	1	3	3	17	23.21	23.21	23.29	23.36	22.10	22.72	3	3	23.37	23.28	23.35	23.12	22.31	22.45	23.33	22.63	0.048	0.029	-0.701
O60502	Protein O-GlcNAcase OS=Homo sapiens GN=MGEA5 PE=1 SV=2 - [OGA_HUMAN]	3.60	1	2	2	7	22.64		22.23	22.98	23.22	23.28	2	3	22.80		22.29	22.73	23.43	23.02	22.55	23.06	0.208	0.086	0.517
Q8WUD1	Ras-related protein Rab-2B OS=Homo sapiens GN=RAB2B PE=1 SV=1 - [RAB2B_HUMAN]	25.46	1	1	5	16	23.48	23.87	23.55	24.14	23.73	24.06	3	3	23.64	23.94	23.61	23.89	23.94	23.80	23.73	23.88	0.268	0.103	0.146
CON_007164551	SWISS-PROT:P02070 Bos taurus Hemoglobin subunit beta	75.17	4	9	10	86	30.14	28.53	29.38	29.91	29.44	30.25	3	3	30.30	28.60	29.44	29.66	29.66	29.99	29.45	29.77	0.557	0.184	0.324
P61586	Transforming protein RhoA OS=Homo sapiens GN=RHOA PE=1 SV=1 - [RHOA_HUMAN]	81.87	1	8	16	158	27.85	28.26	28.21	27.72	27.30	28.12	3	3	28.01	28.33	28.27	27.48	27.52	27.86	28.20	27.62	0.020	0.016	-0.581
P52789	Hexokinase-2 OS=Homo sapiens GN=HK2 PE=1 SV=2 - [HXK2_HUMAN]	26.61	2	14	17	31		22.44	22.42	22.00	23.02	24.24	2	3		22.51	22.48	21.75	23.23	23.98	22.50	22.99	0.602	0.196	0.492
O43747	AP-1 complex subunit gamma-1 OS=Homo sapiens GN=AP1G1 PE=1 SV=5 - [AP1G1_HUMAN]	7.06	5	3	4	7	23.15	24.37	22.10		23.45	21.81	3	2	23.31	24.44	22.16		23.67	21.55	23.30	22.61	0.591	0.193	-0.695
P21281	V-type proton ATPase subunit B, brain isoform OS=Homo sapiens GN=ATP6V1B2 PE=1 SV=3 - [VATB2_HUMAN]	13.70	2	5	5	22	23.64	24.27	23.97	24.24	24.15	24.37	3	3	23.81	24.34	24.03	23.99	24.36	24.10	24.06	24.15	0.643	0.206	0.095
P07948	Tyrosine-protein kinase Lyn OS=Homo sapiens GN=LYN PE=1 SV=3 - [LYN_HUMAN]	32.23	19	10	12	35	24.51	24.55	24.56	24.52	23.19	24.23	3	3	24.67	24.62	24.62	24.27	23.41	23.97	24.64	23.89	0.041	0.026	-0.752
P09471	Guanine nucleotide-binding protein G(o) subunit alpha OS=Homo sapiens GN=GNAO1 PE=1 SV=4 - [GNAO_HUMAN]	20.06	1	4	6	30	26.73	27.10	27.05	28.18	26.81	28.00	3	3	26.89	27.17	27.11	27.93	27.02	27.74	27.06	27.57	0.154	0.069	0.507

P55085	Proteinase-activated receptor 2 OS=Homo sapiens GN=F2RL1 PE=1 SV=1 - [PAR2_HUMAN]	6.80	1	3	3	10	23.17	22.44	24.50	20.00	23.10	22.40	3	3	23.33	22.51	24.56	19.75	23.32	22.14	23.47	21.74	0.225	0.091	-1.728
P30530	Tyrosine-protein kinase receptor UFO OS=Homo sapiens GN=AXL PE=1 SV=3 - [UFO_HUMAN]	20.58	2	14	14	53	24.24	24.17	24.00	24.13	22.22	23.72	3	3	24.41	24.24	24.06	23.88	22.44	23.46	24.24	23.26	0.090	0.046	-0.979
Q9UMX0	Ubiquilin-1 OS=Homo sapiens GN=UBQLN1 PE=1 SV=2 - [UBQL1_HUMAN]	24.62	1	4	9	35	23.41	24.50	24.24	24.83	24.57	24.47	3	3	23.57	24.57	24.30	24.58	24.79	24.21	24.15	24.53	0.334	0.123	0.377
Q15365	Poly(rC)-binding protein 1 OS=Homo sapiens GN=PCBP1 PE=1 SV=2 - [PCBP1_HUMAN]	62.36	2	11	16	116	26.84	26.87	26.94	27.11	26.79	27.59	3	3	27.01	26.94	27.00	26.86	27.01	27.33	26.98	27.07	0.576	0.190	0.085
P62993	Growth factor receptor-bound protein 2 OS=Homo sapiens GN=GRB2 PE=1 SV=1 - [GRB2_HUMAN]	27.19	1	6	6	19	22.24	23.14	23.10	23.81	23.22	23.33	3	3	22.40	23.21	23.16	23.57	23.44	23.07	22.92	23.36	0.221	0.090	0.435
P48637	Glutathione synthetase OS=Homo sapiens GN=GSS PE=1 SV=1 - [GSHB_HUMAN]	23.00	1	8	8	19	22.00	23.26	23.10	23.48	23.24	23.44	3	3	22.16	23.33	23.16	23.23	23.46	23.17	22.88	23.29	0.342	0.125	0.404
Q13907	Isopentenyl-diphosphate Delta-isomerase 1 OS=Homo sapiens GN=IDI1 PE=1 SV=2 - [IDI1_HUMAN]	51.10	3	9	9	45	23.39	23.71	23.84	23.93	23.53	24.73	3	3	23.56	23.77	23.90	23.68	23.74	24.47	23.74	23.97	0.463	0.159	0.221
Q92538	Golgi-specific brefeldin A-resistance guanine nucleotide exchange factor 1 OS=Homo sapiens GN=GBF1 PE=1 SV=2 - [GBF1_HUMAN]	2.04	1	2	3	15	27.07	25.53	25.80	26.91	26.04	26.40	3	3	27.24	25.60	25.86	26.67	26.25	26.14	26.23	26.35	0.830	0.247	0.122
Q9UHB9	Signal recognition particle subunit SRP68 OS=Homo sapiens GN=SRP68 PE=1 SV=2 - [SRP68_HUMAN]	26.16	1	11	11	19		21.23	18.76	23.54	22.63	23.54	2	3		21.30	18.82	23.30	22.84	23.28	20.06	23.14	0.047	0.029	3.078
P10321	HLA class I histocompatibility antigen, Cw-7 alpha chain OS=Homo sapiens GN=HLA-C PE=1 SV=3 - [1C07_HUMAN]	28.96	2	0	7	46	27.71	27.66	27.77	28.62	27.37	28.77	3	3	27.87	27.72	27.83	28.37	27.59	28.50	27.81	28.16	0.297	0.111	0.346
O14907	Tax1-binding protein 3 OS=Homo sapiens GN=TAX1BP3 PE=1 SV=2 - [TX1B3_HUMAN]	13.71	1	1	1	5	24.76		24.72	24.65	24.15	24.33	2	3	24.92		24.78	24.40	24.37	24.07	24.85	24.28	0.030	0.022	-0.570
P17813	Endoglin OS=Homo sapiens GN=ENG PE=1 SV=2 - [EGLN_HUMAN]	27.96	1	12	12	49	24.24	24.82	24.49	24.20	22.41	24.06	3	3	24.40	24.89	24.55	23.96	22.62	23.80	24.61	23.46	0.061	0.034	-1.153
P08779	Keratin, type I cytoskeletal 16 OS=Homo sapiens GN=KRT16 PE=1 SV=4 - [K1C16_HUMAN]	34.88	14	4	15	46	26.45	24.50	24.53	26.40	25.24	24.75	3	3	26.62	24.57	24.59	26.16	25.46	24.49	25.26	25.37	0.902	0.261	0.109
P54578	Ubiquitin carboxyl-terminal hydrolase 14 OS=Homo sapiens GN=USP14 PE=1 SV=3 - [UBP14_HUMAN]	36.23	1	14	14	49	24.18	24.12	24.21	24.45	24.03	24.89	3	3	24.34	24.19	24.27	24.20	24.25	24.63	24.26	24.36	0.539	0.180	0.096
P48059	LIM and senescent cell antigen-like-containing domain protein 1 OS=Homo sapiens GN=LIMS1 PE=1 SV=4 - [LIMS1_HUMAN]	19.69	2	5	5	14	23.32	23.63	23.17	24.57	23.49	23.61	3	3	23.48	23.70	23.23	24.32	23.71	23.35	23.47	23.79	0.363	0.131	0.322
P10301	Ras-related protein R-Ras OS=Homo sapiens GN=RRAS PE=1 SV=1 - [RRAS_HUMAN]	55.05	1	7	10	45	24.93	25.22	24.96	23.62	23.78	25.39	3	3	25.09	25.29	25.02	23.38	23.99	25.13	25.14	24.17	0.136	0.062	-0.968
P14923	Junction plakoglobin OS=Homo sapiens GN=JUP PE=1 SV=3 - [PLAK_HUMAN]	17.18	1	7	9	22	23.16	23.30	22.99	22.78		22.88	3	2	23.32	23.37	23.05	22.53		22.62	23.25	22.58	0.016	0.014	-0.671
P06737	Glycogen phosphorylase, liver form OS=Homo sapiens GN=PYGL PE=1 SV=4 - [PYGL_HUMAN]	17.71	1	8	13	39	23.56	23.16	22.79	24.38	22.87	24.51	3	3	23.73	23.23	22.85	24.13	23.08	24.25	23.27	23.82	0.287	0.108	0.552
Q9Y376	Calcium-binding protein 39 OS=Homo sapiens GN=CAB39 PE=1 SV=1 - [CAB39_HUMAN]	26.69	2	9	9	23	23.16	22.51	22.46	23.25		22.78	3	2	23.32	22.58	22.52	23.00		22.51	22.81	22.76	0.907	0.262	-0.048
Q9P273	Teneurin-3 OS=Homo sapiens GN=TENM3 PE=2 SV=3 - [TEN3_HUMAN]	8.71	1	13	13	25	22.72	23.22	23.09	22.76		23.25	3	2	22.88	23.29	23.15	22.52		22.99	23.11	22.76	0.230	0.092	-0.353
P29144	Tripeptidyl-peptidase 2 OS=Homo sapiens GN=TPP2 PE=1 SV=4 - [TPP2_HUMAN]	19.78	1	17	17	42	23.35	23.40	22.20	24.31	23.04	24.01	3	3	23.51	23.47	22.26	24.07	23.25	23.75	23.08	23.69	0.267	0.103	0.612
P61019	Ras-related protein Rab-2A OS=Homo sapiens GN=RAB2A PE=1 SV=1 - [RAB2A_HUMAN]	28.30	1	1	5	19	24.07	24.51	23.55	24.89	24.21	24.94	3	3	24.23	24.58	23.61	24.65	24.42	24.68	24.14	24.58	0.210	0.086	0.441
Q8WV24	Pleckstrin homology-like domain family A member 1 OS=Homo sapiens GN=PHLDA1 PE=1 SV=4 - [PHLA1_HUMAN]	9.23	1	4	4	21	24.54	24.67	24.39	24.16	23.49	24.50	3	3	24.70	24.74	24.45	23.92	23.70	24.24	24.63	23.95	0.020	0.017	-0.676
P82970	High mobility group nucleosome-binding domain-containing protein 5 OS=Homo sapiens GN=HMGN5 PE=1 SV=1 - [HMGN5_HUMAN]	12.41	1	2	2	12		19.75	19.37	21.80	20.26	21.26	2	3		19.81	19.43	21.56	20.47	21.00	19.62	21.01	0.048	0.029	1.389
Q99442	Translocation protein SEC62 OS=Homo sapiens GN=SEC62 PE=1 SV=1 - [SEC62_HUMAN]	7.52	1	4	4	13	25.24	26.65	21.80	22.18	22.31	22.93	3	3	25.40	26.72	21.86	21.94	22.52	22.67	24.66	22.38	0.195	0.082	-2.281
Q6P9G4	Transmembrane protein 154 OS=Homo sapiens GN=TMEM154 PE=2 SV=2 - [TM154_HUMAN]	17.49	1	2	2	6	22.24	22.32	23.03	23.30		23.31	3	2	22.40	22.39	23.09	23.06		23.05	22.63	23.05	0.248	0.097	0.426
Q9UPN3	Microtubule-actin cross-linking factor 1, isoforms 1/2/3/5 OS=Homo sapiens GN=MACF1 PE=1 SV=4 - [MACF1_HUMAN]	1.64	1	8	9	16	23.46	25.08		22.84	22.72	23.02	2	3	23.62	25.15		22.59	22.94	22.76	24.39	22.76	0.069	0.038	-1.626
P25686	DnaJ homolog subfamily B member 2 OS=Homo sapiens GN=DNAJB2 PE=1 SV=3 - [DNJB2_HUMAN]	17.90	1	4	5	12	24.38	23.51	23.43	22.43		22.92	3	2	24.54	23.58	23.49	22.19		22.66	23.87	22.42	0.054	0.031	-1.448
O43752	Syntaxin-6 OS=Homo sapiens GN=STX6 PE=1 SV=1 - [STX6_HUMAN]	65.88	1	10	10	22	24.11	24.83	25.48	24.42	22.43	24.61	3	3	24.27	24.90	25.53	24.18	22.64	24.35	24.90	23.72	0.145	0.066	-1.180
P20073	Annexin A7 OS=Homo sapiens GN=ANXA7 PE=1 SV=3 - [ANXA7_HUMAN]	36.27	1	14	15	70	25.05	25.90	25.39	25.81	25.63	26.26	3	3	25.21	25.97	25.45	25.56	25.85	26.00	25.54	25.80	0.370	0.132	0.261
P61020	Ras-related protein Rab-5B OS=Homo sapiens GN=RAB5B PE=1 SV=1 - [RAB5B_HUMAN]	51.63	1	5	8	43	25.26	26.24	24.38	25.79	26.05	25.94	3	3	25.42	26.31	24.44	25.55	26.26	25.68	25.39	25.83	0.492	0.167	0.440
Q96G03	Phosphoglucomutase-2 OS=Homo sapiens GN=PGM2 PE=1 SV=4 - [PGM2_HUMAN]	8.66	1	4	4	10		22.80	22.41	23.89	23.11	23.62	2	3		22.87	22.47	23.65	23.33	23.36	22.67	23.45	0.030	0.021	0.772
Q9NRV9	Heme-binding protein 1 OS=Homo sapiens GN=HEBP1 PE=1 SV=1 - [HEBP1_HUMAN]	53.44	1	7	7	43	23.25	23.65	23.18	23.93	23.37	24.27	3	3	23.41	23.72	23.24	23.68	23.59	24.01	23.46	23.76	0.180	0.078	0.307
Q9GZM5	Protein YIPF3 OS=Homo sapiens GN=YIPF3 PE=1 SV=1 - [YIPF3_HUMAN]	14.29	1	2	2	6	22.33	23.20		25.06	23.89		2	2	22.50	23.27		24.82	24.10		22.88	24.46	0.095	0.048	1.580
P23588	Eukaryotic translation initiation factor 4B OS=Homo sapiens GN=EIF4B PE=1 SV=2 - [IF4B_HUMAN]	14.08	1	7	7	20	21.00	21.84	21.63	22.87	21.35	22.87	3	3	21.16	21.91	21.69	22.63	21.57	22.61	21.59	22.27	0.177	0.076	0.679
Q15404	Ras suppressor protein 1 OS=Homo sapiens GN=RSU1 PE=1 SV=3 - [RSU1_HUMAN]	38.63	1	8	8	29	25.18	23.58	23.72	24.91	24.24	24.77	3	3	25.34	23.65	23.78	24.67	24.45	24.51	24.26	24.54	0.628	0.203	0.287
CON_006943041	SWISS-PROT:P17697 Bos taurus Clusterin precursor	38.27	1	12	12	51	26.54	23.45	24.11	25.86	25.85	25.81	3	3	26.70	23.52	24.17	25.61	26.06	25.55	24.80	25.74	0.390	0.137	0.946
Q15029	116 kDa U5 small nuclear ribonucleoprotein component OS=Homo sapiens GN=EFTUD2 PE=1 SV=1 - [U5S1_HUMAN]	28.09	1	17	18	47	24.71		22.63	25.41	24.69	25.48	2	3	24.87		22.69	25.16	24.91	25.22	23.78	25.10	0.208	0.086	1.317
O14908	PDZ domain-containing protein GIPC1 OS=Homo sapiens GN=GIPC1 PE=1 SV=2 - [GIPC1_HUMAN]	34.53	2	10	10	42	24.69	24.17	23.71	24.95	24.06	24.46	3	3	24.85	24.24	23.77	24.71	24.28	24.20	24.29	24.40	0.770	0.234	0.109
Q9H3N1	Thioredoxin-related transmembrane protein 1 OS=Homo sapiens GN=TMX1 PE=1 SV=1 - [TMX1_HUMAN]	23.21	1	5	5	15	22.66	23.13	23.38	23.27	22.88	24.29	3	3	22.82	23.20	23.44	23.03	23.10	24.03	23.16	23.38	0.570	0.188	0.228
Q9NYV4	Cyclin-dependent kinase 12 OS=Homo sapiens GN=CDK12 PE=1 SV=2 - [CDK12_HUMAN]	1.34	18	1	2	8	24.66	25.07	25.08	24.74	22.36	24.60	3	3	24.82	25.14	25.13	24.49	22.57	24.34	25.03	23.80	0.121	0.057	-1.228
P19256	Lymphocyte function-associated antigen 3 OS=Homo sapiens GN=CD58 PE=1 SV=1 - [LFA3_HUMAN]	13.20	1	3	3	14	25.82	25.94	25.79	25.50	24.32	25.86	3	3	25.98	26.01	25.85	25.26	24.53	25.60	25.95	25.13	0.063	0.035	-0.815
P29317	Ephrin type-A receptor 2 OS=Homo sapiens GN=EPHA2 PE=1 SV=2 - [EPHA2_HUMAN]	57.79	6	41	43	231	26.54	26.59	26.87	26.78	24.95	26.30	3	3	26.70	26.66	26.93	26.53	25.16	26.03	26.76	25.91	0.106	0.052	-0.853
O94812	BAI1-associated protein 3 OS=Homo sapiens GN=BAIAP3 PE=1 SV=2 - [BAIP3_HUMAN]	1.01	3	1	2	9	28.52	27.66	31.66	24.53	29.27	26.45	3	3	28.69	27.73	31.72	24.29	29.49	26.19	29.38	26.66	0.233	0.093	-2.721
Q9BXB5	Oxysterol-binding protein-related protein 10 OS=Homo sapiens GN=OSBPL10 PE=1 SV=2 - [OSB10_HUMAN]	6.15	1	3	3	5	20.87		21.05	20.14		20.87	2	2	21.03		21.10	19.89		20.61	21.07	20.25	0.154	0.069	-0.814
Q9BRX2	Protein pelota homolog OS=Homo sapiens GN=PELO PE=1 SV=2 - [PELO_HUMAN]	21.82	1	4	4	9	22.07	21.65	21.98	21.90	22.29	22.40	3	3	22.24	21.72	22.04	21.65	22.51	22.13	22.00	22.10	0.748	0.230	0.100
P04792	Heat shock protein beta-1 OS=Homo sapiens GN=HSPB1 PE=1 SV=2 - [HSPB1_HUMAN]	81.95	1	11	11	63	26.11	25.47	25.52	25.88	26.04	26.22	3	3	26.27	25.54	25.58	25.63	26.26	25.96	25.80	25.95	0.632	0.204	0.154
P39656	Dolichyl-diphosphooligosaccharide--protein glycosyltransferase 48 kDa subunit OS=Homo sapiens GN=DDOST PE=1 SV=4 - [OST48_HUMAN]	29.82	1	8	8	34	24.16	23.16	24.72	23.81	23.98	25.86	3	3	24.32	23.23	24.78	23.56	24.20	25.60	24.11	24.45	0.672	0.213	0.346
P20674	Cytochrome c oxidase subunit 5A, mitochondrial OS=Homo sapiens GN=COX5A PE=1 SV=2 - [COX5A_HUMAN]	56.67	1	5	6	22		25.46	24.64	23.49	25.79	26.10	2	3		25.53	24.70	23.24	26.01	25.84	25.12	25.03	0.947	0.270	-0.086
Q06124	Tyrosine-protein phosphatase non-receptor type 11 OS=Homo sapiens GN=PTPN11 PE=1 SV=2 - [PTN11_HUMAN]	22.11	1	9	9	22	23.08	22.75	22.58	23.63	22.51	24.40	3	3	23.24	22.82	22.64	23.38	22.73	24.14	22.90	23.42	0.312	0.116	0.515
P48426	Phosphatidylinositol 5-phosphate 4-kinase type-2 alpha OS=Homo sapiens GN=PIP4K2A PE=1 SV=2 - [PI42A_HUMAN]	15.52	2	5	5	14	20.88	22.41		24.03	23.12	23.72	2	3	21.04	22.48		23.78	23.34	23.45	21.76	23.52	0.052	0.030	1.763
P30464	HLA class I histocompatibility antigen, B-15 alpha chain OS=Homo sapiens GN=HLA-B PE=1 SV=2 - [1B15_HUMAN]	36.46	8	0	10	56	26.46	26.64	26.56	27.87	26.18	27.67	3	3	26.62	26.71	26.62	27.63	26.39	27.41	26.65	27.14	0.266	0.102	0.494
P30490	HLA class I histocompatibility antigen, B-52 alpha chain OS=Homo sapiens GN=HLA-B PE=1 SV=1 - [1B52_HUMAN]	33.43	6	0	9	54	26.46	26.64	26.56	27.87	26.18	27.67	3	3	26.62	26.71	26.62	27.63	26.39	27.41	26.65	27.14	0.266	0.102	0.494
Q9Y2Z0	Protein SGT1 homolog OS=Homo sapiens GN=SUGT1 PE=1 SV=3 - [SGT1_HUMAN]	20.55	1	5	5	19	23.25		21.44	23.80	23.38	23.87	2	3	23.41		21.50	23.55	23.60	23.61	22.46	23.59	0.210	0.087	1.131
P30086	Phosphatidylethanolamine-binding protein 1 OS=Homo sapiens GN=PEBP1 PE=1 SV=3 - [PEBP1_HUMAN]	73.80	1	10	10	60	25.22	26.81	26.78	27.03	26.64	27.20	3	3	25.38	26.87	26.84	26.79	26.85	26.94	26.36	26.86	0.371	0.132	0.498
Q96FZ5	CKLF-like MARVEL transmembrane domain-containing protein 7 OS=Homo sapiens GN=CMTM7 PE=2 SV=1 - [CKLF7_HUMAN]	4.57	1	1	1	6	23.35	23.45	23.53	23.55	21.79	23.08	3	3	23.51	23.52	23.59	23.30	22.01	22.82	23.54	22.71	0.094	0.048	-0.832
P18084	Integrin beta-5 OS=Homo sapiens GN=ITGB5 PE=1 SV=1 - [ITB5_HUMAN]	35.54	3	19	19	70	25.42	25.37	24.78	24.92	22.54	24.94	3	3	25.59	25.44	24.84	24.67	22.75	24.68	25.29	24.03	0.139	0.064	-1.253
P08243	Asparagine synthetase [glutamine-hydrolyzing] OS=Homo sapiens GN=ASNS PE=1 SV=4 - [ASNS_HUMAN]	24.96	1	10	10	22	22.92	23.15	22.87	22.73	23.54	23.76	3	3	23.08	23.22	22.92	22.49	23.76	23.50	23.08	23.25	0.687	0.216	0.172
Q8IYJ1	Copine-9 OS=Homo sapiens GN=CPNE9 PE=1 SV=3 - [CPNE9_HUMAN]	10.13	4	1	5	15	23.98	24.09	23.40	23.40	22.78	23.68	3	3	24.14	24.16	23.46	23.16	23.00	23.42	23.92	23.19	0.049	0.029	-0.729
Q9BS26	Endoplasmic reticulum resident protein 44 OS=Homo sapiens GN=ERP44 PE=1 SV=1 - [ERP44_HUMAN]	27.09	1	8	9	30	20.39		24.15	23.34	24.25	24.79	2	3	20.56		24.21	23.10	24.46	24.53	22.38	24.03	0.349	0.126	1.649
P36405	ADP-ribosylation factor-like protein 3 OS=Homo sapiens GN=ARL3 PE=1 SV=2 - [ARL3_HUMAN]	36.81	1	4	4	12	22.55	23.43		24.32	23.81	24.21	2	3	22.71	23.50		24.07	24.03	23.95	23.10	24.02	0.055	0.032	0.913
Q4KMQ2	Anoctamin-6 OS=Homo sapiens GN=ANO6 PE=1 SV=2 - [ANO6_HUMAN]	4.51	1	3	3	8	22.37	22.91	21.47	20.46		21.05	3	2	22.53	22.98	21.53	20.21		20.79	22.35	20.50	0.054	0.031	-1.844
P08174	Complement decay-accelerating factor OS=Homo sapiens GN=CD55 PE=1 SV=4 - [DAF_HUMAN]	43.31	1	12	12	70	26.93	31.74	26.79	26.58	25.30	26.50	3	3	27.09	31.81	26.85	26.33	25.52	26.24	28.59	26.03	0.193	0.081	-2.554
O15118	Niemann-Pick C1 protein OS=Homo sapiens GN=NPC1 PE=1 SV=2 - [NPC1_HUMAN]	8.61	1	8	8	16	23.96	20.54		24.57	23.40	23.90	2	3	24.12	20.61		24.32	23.61	23.64	22.36	23.86	0.346	0.126	1.496
Q92882	Osteoclast-stimulating factor 1 OS=Homo sapiens GN=OSTF1 PE=1 SV=2 - [OSTF1_HUMAN]	34.58	1	4	4	11		22.11	22.10	23.46	22.33	23.12	2	3		22.18	22.16	23.22	22.54	22.86	22.17	22.87	0.067	0.037	0.707
Q93096	Protein tyrosine phosphatase type IVA 1 OS=Homo sapiens GN=PTP4A1 PE=1 SV=2 - [TP4A1_HUMAN]	41.62	1	2	5	16	23.21	22.44	22.62	23.82	22.13	23.88	3	3	23.37	22.51	22.68	23.58	22.34	23.62	22.85	23.18	0.542	0.181	0.330
P11021	78 kDa glucose-regulated protein OS=Homo sapiens GN=HSPA5 PE=1 SV=2 - [GRP78_HUMAN]	55.50	1	33	35	209	29.47	30.20	30.14	30.37	30.11	30.33	3	3	29.63	30.27	30.20	30.12	30.33	30.07	30.03	30.18	0.552	0.183	0.141
CON_006888152	SWISS-PROT:P81644 Bos taurus Apolipoprotein A-II precursor	28.00	1	5	5	25	22.08	26.11	25.65	26.75	26.39	25.77	3	3	22.25	26.18	25.71	26.50	26.60	25.51	24.71	26.21	0.310	0.116	1.495
Q6UXN9	WD repeat-containing protein 82 OS=Homo sapiens GN=WDR82 PE=1 SV=1 - [WDR82_HUMAN]	12.78	13	2	3	7	24.03		23.55	21.02	21.70	22.96	2	3	24.20		23.61	20.78	21.92	22.70	23.90	21.80	0.068	0.037	-2.105
P20338	Ras-related protein Rab-4A OS=Homo sapiens GN=RAB4A PE=1 SV=3 - [RAB4A_HUMAN]	11.01	7	1	2	13	28.26	28.69	28.77	28.57	28.02	27.83	3	3	28.42	28.76	28.83	28.33	28.23	27.57	28.67	28.05	0.082	0.043	-0.622
Q96QD8	Sodium-coupled neutral amino acid transporter 2 OS=Homo sapiens GN=SLC38A2 PE=1 SV=2 - [S38A2_HUMAN]	15.61	1	5	5	35	25.79	25.33	25.00	26.64	25.03	25.63	3	3	25.95	25.40	25.06	26.40	25.25	25.37	25.47	25.67	0.680	0.215	0.199
O75044	SLIT-ROBO Rho GTPase-activating protein 2 OS=Homo sapiens GN=SRGAP2 PE=1 SV=2 - [SRGP2_HUMAN]	5.70	4	4	4	13	22.32	21.69	21.83	23.22	21.27	23.02	3	3	22.49	21.76	21.88	22.97	21.48	22.76	22.04	22.40	0.523	0.175	0.362
Q13636	Ras-related protein Rab-31 OS=Homo sapiens GN=RAB31 PE=1 SV=1 - [RAB31_HUMAN]	22.16	1	1	3	10	23.43	24.25	24.14	23.90		24.23	3	2	23.59	24.32	24.20	23.66		23.97	24.03	23.81	0.530	0.178	-0.221
Q969P0	Immunoglobulin superfamily member 8 OS=Homo sapiens GN=IGSF8 PE=1 SV=1 - [IGSF8_HUMAN]	31.65	1	11	11	47	25.00	25.13	25.04	25.07	23.45	24.84	3	3	25.16	25.20	25.10	24.82	23.67	24.58	25.15	24.36	0.086	0.045	-0.797
P13797	Plastin-3 OS=Homo sapiens GN=PLS3 PE=1 SV=4 - [PLST_HUMAN]	58.25	1	20	27	138	25.25	25.20	24.78	26.00	24.80	26.28	3	3	25.41	25.27	24.84	25.75	25.01	26.02	25.18	25.60	0.292	0.110	0.420
O00161	Synaptosomal-associated protein 23 OS=Homo sapiens GN=SNAP23 PE=1 SV=1 - [SNP23_HUMAN]	57.35	1	11	11	62	24.34	24.50	24.33	25.31	24.22	24.85	3	3	24.50	24.57	24.39	25.06	24.44	24.59	24.49	24.69	0.347	0.126	0.208
Q9UHD2	Serine/threonine-protein kinase TBK1 OS=Homo sapiens GN=TBK1 PE=1 SV=1 - [TBK1_HUMAN]	3.43	1	1	1	5	23.21		22.62	22.73	21.76	22.53	2	3	23.37		22.68	22.49	21.98	22.27	23.02	22.25	0.093	0.048	-0.780
Q9BXS4	Transmembrane protein 59 OS=Homo sapiens GN=TMEM59 PE=1 SV=1 - [TMM59_HUMAN]	15.48	1	5	5	19	22.95	23.18		25.55	23.65	24.46	2	3	23.11	23.25		25.31	23.86	24.20	23.18	24.46	0.109	0.053	1.280
O14936	Peripheral plasma membrane protein CASK OS=Homo sapiens GN=CASK PE=1 SV=3 - [CSKP_HUMAN]	9.61	1	6	6	19	23.64	23.33	23.62	23.40		22.28	3	2	23.80	23.40	23.68	23.16		22.02	23.63	22.59	0.105	0.052	-1.038
Q99584	Protein S100-A13 OS=Homo sapiens GN=S100A13 PE=1 SV=1 - [S10AD_HUMAN]	46.94	1	5	5	23	23.97	25.26	25.16	24.33	23.30	24.55	3	3	24.13	25.33	25.22	24.09	23.51	24.29	24.89	23.96	0.107	0.052	-0.930
P51572	B-cell receptor-associated protein 31 OS=Homo sapiens GN=BCAP31 PE=1 SV=3 - [BAP31_HUMAN]	20.73	1	7	7	29	23.28	23.62	23.71	23.77	23.32	25.12	3	3	23.44	23.69	23.77	23.53	23.54	24.86	23.63	23.97	0.490	0.167	0.343
P11233	Ras-related protein Ral-A OS=Homo sapiens GN=RALA PE=1 SV=1 - [RALA_HUMAN]	48.54	1	4	11	77	25.80	26.28	25.94	26.72	26.07	26.31	3	3	25.96	26.35	26.00	26.47	26.28	26.05	26.11	26.27	0.407	0.142	0.161
Q96IZ0	PRKC apoptosis WT1 regulator protein OS=Homo sapiens GN=PAWR PE=1 SV=1 - [PAWR_HUMAN]	3.82	1	1	1	6		21.68	21.78	22.00	21.26	21.70	2	3		21.75	21.84	21.76	21.48	21.44	21.79	21.56	0.181	0.078	-0.232
Q9NVD7	Alpha-parvin OS=Homo sapiens GN=PARVA PE=1 SV=1 - [PARVA_HUMAN]	20.97	3	7	7	17	23.17	22.46	22.69	22.58	22.17	22.86	3	3	23.33	22.53	22.75	22.33	22.38	22.60	22.87	22.44	0.160	0.071	-0.433
P10155	60 kDa SS-A/Ro ribonucleoprotein OS=Homo sapiens GN=TROVE2 PE=1 SV=2 - [RO60_HUMAN]	9.85	1	4	4	10	22.95	22.86	22.32	23.03	22.83	23.27	3	3	23.11	22.93	22.38	22.79	23.05	23.01	22.81	22.95	0.580	0.190	0.141
P18433	Receptor-type tyrosine-protein phosphatase alpha OS=Homo sapiens GN=PTPRA PE=1 SV=2 - [PTPRA_HUMAN]	14.59	1	7	8	13	22.96	23.01	22.70	21.59		22.47	3	2	23.12	23.08	22.76	21.35		22.20	22.99	21.77	0.042	0.026	-1.212
P33527	Multidrug resistance-associated protein 1 OS=Homo sapiens GN=ABCC1 PE=1 SV=3 - [MRP1_HUMAN]	8.88	1	7	7	15		21.47	21.16	22.40	21.88	23.59	2	3		21.54	21.22	22.15	22.09	23.33	21.38	22.53	0.121	0.057	1.146
Q9Y696	Chloride intracellular channel protein 4 OS=Homo sapiens GN=CLIC4 PE=1 SV=4 - [CLIC4_HUMAN]	82.21	2	17	19	120	26.26	26.32	25.92	26.03	24.63	26.05	3	3	26.42	26.39	25.98	25.78	24.84	25.79	26.27	25.47	0.083	0.043	-0.794
O60664	Perilipin-3 OS=Homo sapiens GN=PLIN3 PE=1 SV=3 - [PLIN3_HUMAN]	29.95	1	9	9	33	24.45	23.84	23.91	23.88	23.21	24.18	3	3	24.62	23.91	23.97	23.63	23.42	23.92	24.17	23.66	0.130	0.060	-0.508
P62258	14-3-3 protein epsilon OS=Homo sapiens GN=YWHAE PE=1 SV=1 - [1433E_HUMAN]	76.47	1	19	20	155	27.58	28.50	28.45	29.07	28.18	28.97	3	3	27.74	28.57	28.51	28.83	28.40	28.71	28.28	28.65	0.281	0.107	0.369
Q9Y287	Integral membrane protein 2B OS=Homo sapiens GN=ITM2B PE=1 SV=1 - [ITM2B_HUMAN]	19.17	1	3	3	13	23.84	23.53	22.00	24.57	23.07	24.53	3	3	24.00	23.60	22.05	24.32	23.28	24.27	23.22	23.96	0.339	0.124	0.739
P06756	Integrin alpha-V OS=Homo sapiens GN=ITGAV PE=1 SV=2 - [ITAV_HUMAN]	36.45	1	34	34	129	25.23	25.23	24.96	25.06	24.08	25.06	3	3	25.39	25.30	25.02	24.81	24.29	24.80	25.24	24.64	0.043	0.027	-0.602
P01112	GTPase HRas OS=Homo sapiens GN=HRAS PE=1 SV=1 - [RASH_HUMAN]	73.02	1	3	12	79	26.10	26.92	26.94	26.67	24.88	25.98	3	3	26.26	26.99	27.00	26.43	25.09	25.72	26.75	25.75	0.093	0.048	-1.001
P40938	Replication factor C subunit 3 OS=Homo sapiens GN=RFC3 PE=1 SV=2 - [RFC3_HUMAN]	3.93	1	1	1	5	20.77	21.09	20.41		21.32	21.91	3	2	20.93	21.16	20.47		21.54	21.65	20.85	21.59	0.067	0.037	0.741
Q15436	Protein transport protein Sec23A OS=Homo sapiens GN=SEC23A PE=1 SV=2 - [SC23A_HUMAN]	17.12	1	7	7	33	22.77	22.75	22.17	23.31	22.40	23.79	3	3	22.94	22.82	22.23	23.06	22.61	23.53	22.66	23.07	0.299	0.112	0.409
Q15836	Vesicle-associated membrane protein 3 OS=Homo sapiens GN=VAMP3 PE=1 SV=3 - [VAMP3_HUMAN]	53.00	2	4	7	28	25.30	25.47	25.64	25.39	25.32	25.24	3	3	25.46	25.53	25.70	25.15	25.54	24.98	25.57	25.22	0.127	0.059	-0.345
Q9BR76	Coronin-1B OS=Homo sapiens GN=CORO1B PE=1 SV=1 - [COR1B_HUMAN]	31.70	1	10	11	31		23.45	22.14	24.70	23.89	25.01	2	3		23.52	22.20	24.45	24.11	24.75	22.86	24.44	0.064	0.036	1.574
P49721	Proteasome subunit beta type-2 OS=Homo sapiens GN=PSMB2 PE=1 SV=1 - [PSB2_HUMAN]	40.80	1	6	6	21		23.42	23.26	24.04	23.65	24.77	2	3		23.49	23.32	23.79	23.87	24.51	23.41	24.06	0.119	0.056	0.649
Q15058	Kinesin-like protein KIF14 OS=Homo sapiens GN=KIF14 PE=1 SV=1 - [KIF14_HUMAN]	6.98	1	9	9	15	21.46	21.26	23.46	24.40	23.44	22.29	3	3	21.62	21.32	23.52	24.15	23.66	22.03	22.15	23.28	0.297	0.111	1.126
Q9UKE5	TRAF2 and NCK-interacting protein kinase OS=Homo sapiens GN=TNIK PE=1 SV=1 - [TNIK_HUMAN]	2.72	1	1	5	11	22.50	22.21	22.05		19.03	21.58	3	2	22.66	22.28	22.11		19.24	21.31	22.35	20.28	0.081	0.043	-2.073
P16520	Guanine nucleotide-binding protein G(I)/G(S)/G(T) subunit beta-3 OS=Homo sapiens GN=GNB3 PE=1 SV=1 - [GBB3_HUMAN]	7.06	1	1	3	12	27.33	27.61	28.21	27.59	26.34	27.83	3	3	27.49	27.68	28.27	27.35	26.55	27.57	27.82	27.16	0.164	0.072	-0.659
P20339	Ras-related protein Rab-5A OS=Homo sapiens GN=RAB5A PE=1 SV=2 - [RAB5A_HUMAN]	66.98	1	6	9	79	25.81	26.42	25.43	26.26	26.15	26.34	3	3	25.97	26.49	25.49	26.01	26.36	26.08	25.98	26.15	0.616	0.199	0.168
Q16563	Synaptophysin-like protein 1 OS=Homo sapiens GN=SYPL1 PE=1 SV=1 - [SYPL1_HUMAN]	6.95	1	2	2	11	25.17	24.73	25.23	24.97	23.91	25.37	3	3	25.34	24.80	25.28	24.72	24.12	25.11	25.14	24.65	0.217	0.089	-0.489
P63000	Ras-related C3 botulinum toxin substrate 1 OS=Homo sapiens GN=RAC1 PE=1 SV=1 - [RAC1_HUMAN]	58.85	4	5	10	64	27.10	27.53	27.47	27.20	25.73	27.30	3	3	27.26	27.60	27.53	26.96	25.95	27.04	27.46	26.65	0.091	0.047	-0.810
Q9Y3L5	Ras-related protein Rap-2c OS=Homo sapiens GN=RAP2C PE=1 SV=1 - [RAP2C_HUMAN]	60.11	2	6	10	66	25.65	25.54	25.25	25.41	23.67	25.42	3	3	25.81	25.61	25.31	25.17	23.88	25.16	25.57	24.74	0.136	0.062	-0.839
Q13404	Ubiquitin-conjugating enzyme E2 variant 1 OS=Homo sapiens GN=UBE2V1 PE=1 SV=2 - [UB2V1_HUMAN]	63.95	2	10	10	26		24.37	23.95	25.54	24.55	25.26	2	3		24.44	24.01	25.30	24.77	25.00	24.22	25.02	0.052	0.030	0.797
O43583	Density-regulated protein OS=Homo sapiens GN=DENR PE=1 SV=2 - [DENR_HUMAN]	35.35	1	5	5	19	21.52		23.49	23.90	23.80	23.82	2	3	21.68		23.55	23.65	24.01	23.56	22.61	23.74	0.214	0.088	1.132
O14879	Interferon-induced protein with tetratricopeptide repeats 3 OS=Homo sapiens GN=IFIT3 PE=1 SV=1 - [IFIT3_HUMAN]	8.57	1	3	3	10	20.89	20.83	20.49	20.72	21.06	22.68	3	3	21.05	20.90	20.55	20.47	21.28	22.42	20.83	21.39	0.393	0.138	0.559
Q9Y639	Neuroplastin OS=Homo sapiens GN=NPTN PE=1 SV=2 - [NPTN_HUMAN]	16.83	1	6	6	40	24.44	25.46	25.31	25.60	25.07	25.94	3	3	24.60	25.53	25.37	25.35	25.29	25.68	25.17	25.44	0.430	0.149	0.273
Q86VI3	Ras GTPase-activating-like protein IQGAP3 OS=Homo sapiens GN=IQGAP3 PE=1 SV=2 - [IQGA3_HUMAN]	5.40	1	3	7	16	22.57	25.01	24.39	23.60	21.83	22.79	3	3	22.73	25.08	24.45	23.35	22.05	22.53	24.09	22.64	0.144	0.065	-1.447
O60684	Importin subunit alpha-7 OS=Homo sapiens GN=KPNA6 PE=1 SV=1 - [IMA7_HUMAN]	20.15	2	3	7	30	23.19	23.70	23.88	23.69	23.46	24.37	3	3	23.35	23.77	23.93	23.44	23.67	24.11	23.68	23.74	0.835	0.248	0.058
P04040	Catalase OS=Homo sapiens GN=CAT PE=1 SV=3 - [CATA_HUMAN]	29.60	1	10	10	22		23.33	22.82	22.36	23.47	23.86	2	3		23.40	22.88	22.11	23.69	23.60	23.14	23.13	0.993	0.281	-0.007
P37802	Transgelin-2 OS=Homo sapiens GN=TAGLN2 PE=1 SV=3 - [TAGL2_HUMAN]	89.95	2	17	17	132	26.51	26.46	26.25	26.78	26.26	27.13	3	3	26.67	26.53	26.31	26.53	26.47	26.87	26.50	26.63	0.490	0.167	0.123
Q9BWD1	Acetyl-CoA acetyltransferase, cytosolic OS=Homo sapiens GN=ACAT2 PE=1 SV=2 - [THIC_HUMAN]	41.56	1	9	9	47	24.36	25.29	24.99	25.14	24.87	25.92	3	3	24.53	25.36	25.05	24.89	25.09	25.66	24.98	25.21	0.522	0.175	0.235
P01116	GTPase KRas OS=Homo sapiens GN=KRAS PE=1 SV=1 - [RASK_HUMAN]	61.90	1	2	9	56	26.10	26.84	26.82	26.74	24.88	25.84	3	3	26.26	26.91	26.88	26.50	25.09	25.58	26.68	25.73	0.108	0.052	-0.955
CON_007011661	SWISS-PROT:P01044-1 Bos taurus Isoform HMW of Kininogen-1 precursor	13.53	2	6	6	14	23.48		22.38	23.69	23.32	24.06	2	3	23.65		22.44	23.45	23.54	23.80	23.04	23.59	0.325	0.120	0.553
P15311	Ezrin OS=Homo sapiens GN=EZR PE=1 SV=4 - [EZRI_HUMAN]	58.36	1	24	37	236	27.41	28.28	28.12	28.62	27.96	28.56	3	3	27.57	28.35	28.17	28.37	28.17	28.29	28.03	28.28	0.364	0.131	0.249
P24666	Low molecular weight phosphotyrosine protein phosphatase OS=Homo sapiens GN=ACP1 PE=1 SV=3 - [PPAC_HUMAN]	39.24	1	5	5	11	23.54	23.45	22.97	23.61	23.38	23.82	3	3	23.70	23.52	23.02	23.37	23.60	23.56	23.42	23.51	0.687	0.216	0.093
P30493	HLA class I histocompatibility antigen, B-55 alpha chain OS=Homo sapiens GN=HLA-B PE=1 SV=1 - [1B55_HUMAN]	35.08	7	0	9	46	26.46	26.64	26.56	27.71	26.05	27.66	3	3	26.62	26.71	26.62	27.46	26.27	27.39	26.65	27.04	0.368	0.132	0.393
Q9NY33	Dipeptidyl peptidase 3 OS=Homo sapiens GN=DPP3 PE=1 SV=2 - [DPP3_HUMAN]	8.82	1	5	5	13		22.49	22.25	23.44	22.59	23.06	2	3		22.56	22.31	23.19	22.80	22.80	22.43	22.93	0.082	0.043	0.499
P54852	Epithelial membrane protein 3 OS=Homo sapiens GN=EMP3 PE=1 SV=1 - [EMP3_HUMAN]	9.82	1	1	1	5	23.59	23.93	24.16	22.53		24.04	3	2	23.75	24.00	24.22	22.29		23.78	23.99	23.03	0.197	0.082	-0.961
Q14019	Coactosin-like protein OS=Homo sapiens GN=COTL1 PE=1 SV=3 - [COTL1_HUMAN]	70.42	1	9	9	46	25.98	25.97	25.70	25.74	25.22	25.89	3	3	26.14	26.04	25.76	25.50	25.44	25.63	25.98	25.52	0.021	0.017	-0.463
P01111	GTPase NRas OS=Homo sapiens GN=NRAS PE=1 SV=1 - [RASN_HUMAN]	64.55	1	2	11	71	26.10	26.92	26.94	26.76	24.88	25.98	3	3	26.26	26.99	27.00	26.52	25.09	25.72	26.75	25.78	0.113	0.054	-0.971
P13646	Keratin, type I cytoskeletal 13 OS=Homo sapiens GN=KRT13 PE=1 SV=4 - [K1C13_HUMAN]	12.23	8	2	5	12	25.77	25.24	23.52	25.54	25.50	25.32	3	3	25.93	25.31	23.58	25.29	25.72	25.06	24.94	25.36	0.602	0.196	0.413
Q8TAD7	Overexpressed in colon carcinoma 1 protein OS=Homo sapiens GN=OCC1 PE=1 SV=2 - [OCC1_HUMAN]	53.97	1	2	2	6	23.86	26.14	25.99	24.78	23.45	24.64	3	3	24.02	26.21	26.05	24.53	23.66	24.38	25.43	24.19	0.177	0.077	-1.233
O15400	Syntaxin-7 OS=Homo sapiens GN=STX7 PE=1 SV=4 - [STX7_HUMAN]	41.76	1	5	5	15	22.84	23.23	23.06	23.50	22.90	23.71	3	3	23.00	23.30	23.12	23.26	23.11	23.45	23.14	23.27	0.369	0.132	0.134

Q01581	Hydroxymethylglutaryl-CoA synthase, cytoplasmic OS=Homo sapiens GN=HMGCS1 PE=1 SV=2 - [HMCS1_HUMAN]	23.08	2	9	9	24	23.48	22.83	23.20	23.13	21.76	23.47	3	3	23.65	22.90	23.26	22.89	21.98	23.21	23.27	22.69	0.252	0.098	-0.574
O75674	TOM1-like protein 1 OS=Homo sapiens GN=TOM1L1 PE=1 SV=2 - [TM1L1_HUMAN]	11.13	1	2	2	13	24.10	24.37	23.87	23.95	23.62	24.01	3	3	24.26	24.44	23.93	23.71	23.84	23.75	24.21	23.76	0.046	0.028	-0.447
P29279	Connective tissue growth factor OS=Homo sapiens GN=CTGF PE=1 SV=2 - [CTGF_HUMAN]	61.03	1	19	19	86	25.34	25.39	25.36	25.46	25.28	24.46	3	3	25.50	25.46	25.42	25.21	25.50	24.20	25.46	24.97	0.283	0.107	-0.489
P08134	Rho-related GTP-binding protein RhoC OS=Homo sapiens GN=RHOC PE=1 SV=1 - [RHOC_HUMAN]	67.88	1	3	11	107	27.44	27.82	27.82	27.33	27.12	27.94	3	3	27.61	27.89	27.88	27.09	27.33	27.68	27.79	27.37	0.093	0.048	-0.427
P50281	Matrix metalloproteinase-14 OS=Homo sapiens GN=MMP14 PE=1 SV=3 - [MMP14_HUMAN]	32.99	3	20	20	76	25.14	24.87	24.85	25.25	22.96	24.80	3	3	25.30	24.94	24.91	25.00	23.17	24.54	25.05	24.24	0.222	0.090	-0.815
P09382	Galectin-1 OS=Homo sapiens GN=LGALS1 PE=1 SV=2 - [LEG1_HUMAN]	86.67	1	9	9	84	26.61	26.55	26.45	26.81	26.37	27.21	3	3	26.77	26.62	26.51	26.57	26.59	26.95	26.63	26.70	0.671	0.213	0.067
O14737	Programmed cell death protein 5 OS=Homo sapiens GN=PDCD5 PE=1 SV=3 - [PDCD5_HUMAN]	57.60	1	7	7	25		22.81	23.83	23.75	23.30	23.87	2	3		22.88	23.89	23.51	23.51	23.61	23.38	23.54	0.702	0.219	0.159
Q14651	Plastin-1 OS=Homo sapiens GN=PLS1 PE=1 SV=2 - [PLSI_HUMAN]	13.83	1	1	6	27	23.91	24.06	23.41	24.81	23.43	25.04	3	3	24.07	24.13	23.47	24.56	23.65	24.78	23.89	24.33	0.339	0.124	0.440
P35241	Radixin OS=Homo sapiens GN=RDX PE=1 SV=1 - [RADI_HUMAN]	48.71	1	12	29	183	27.41	28.31	28.12	28.62	27.96	28.56	3	3	27.57	28.38	28.18	28.37	28.17	28.29	28.04	28.28	0.401	0.140	0.235
CON_006876511	SWISS-PROT:P28800 Bos taurus Alpha-2-antiplasmin precursor	16.87	2	6	6	20	24.50	23.07	23.83	24.34	23.35	24.14	3	3	24.67	23.14	23.89	24.10	23.57	23.88	23.90	23.85	0.923	0.265	-0.048
O00469	Procollagen-lysine,2-oxoglutarate 5-dioxygenase 2 OS=Homo sapiens GN=PLOD2 PE=1 SV=2 - [PLOD2_HUMAN]	5.02	1	2	2	8	22.91	22.60	23.16		22.23	23.13	3	2	23.08	22.67	23.22		22.44	22.87	22.99	22.66	0.300	0.112	-0.332
O15230	Laminin subunit alpha-5 OS=Homo sapiens GN=LAMA5 PE=1 SV=8 - [LAMA5_HUMAN]	2.49	1	5	5	10		19.70	20.72	22.06	21.13	20.85	2	3		19.77	20.78	21.82	21.34	20.59	20.27	21.25	0.201	0.084	0.975
P26038	Moesin OS=Homo sapiens GN=MSN PE=1 SV=3 - [MOES_HUMAN]	69.67	1	35	52	432	27.41	28.31	28.14	28.62	27.96	28.56	3	3	27.57	28.38	28.20	28.37	28.17	28.29	28.05	28.28	0.411	0.143	0.231
P12814	Alpha-actinin-1 OS=Homo sapiens GN=ACTN1 PE=1 SV=2 - [ACTN1_HUMAN]	65.02	1	26	45	324	26.84	27.25	27.08	27.69	26.77	27.98	3	3	27.00	27.32	27.14	27.44	26.99	27.72	27.15	27.38	0.380	0.135	0.229
P15121	Aldose reductase OS=Homo sapiens GN=AKR1B1 PE=1 SV=3 - [ALDR_HUMAN]	58.54	1	13	14	78	24.75	25.51	24.83	24.95	25.32	25.60	3	3	24.91	25.58	24.89	24.71	25.54	25.34	25.13	25.20	0.851	0.251	0.068
Q9BZL1	Ubiquitin-like protein 5 OS=Homo sapiens GN=UBL5 PE=1 SV=1 - [UBL5_HUMAN]	24.66	1	2	2	6	23.59	23.21	23.54	23.39		23.53	3	2	23.76	23.28	23.60	23.14		23.26	23.55	23.20	0.163	0.072	-0.343
P51149	Ras-related protein Rab-7a OS=Homo sapiens GN=RAB7A PE=1 SV=1 - [RAB7A_HUMAN]	80.19	3	17	17	138	26.84	27.37	27.21	27.19	26.16	26.81	3	3	27.01	27.44	27.27	26.95	26.37	26.55	27.24	26.62	0.044	0.027	-0.615
P01892	HLA class I histocompatibility antigen, A-2 alpha chain OS=Homo sapiens GN=HLA-A PE=1 SV=1 - [1A02_HUMAN]	59.73	1	1	20	163	28.24	28.13	28.25	28.97	27.73	28.98	3	3	28.41	28.20	28.31	28.72	27.94	28.71	28.30	28.46	0.588	0.192	0.157
Q13144	Translation initiation factor eIF-2B subunit epsilon OS=Homo sapiens GN=EIF2B5 PE=1 SV=3 - [EI2BE_HUMAN]	21.08	1	9	9	23	24.43	22.58	23.56		23.41	23.62	3	2	24.59	22.65	23.62		23.63	23.36	23.62	23.49	0.874	0.255	-0.126
Q15382	GTP-binding protein Rheb OS=Homo sapiens GN=RHEB PE=1 SV=1 - [RHEB_HUMAN]	51.09	1	10	10	36	25.12	24.44	24.12	24.39	24.05	24.41	3	3	25.28	24.51	24.18	24.15	24.27	24.14	24.66	24.19	0.225	0.091	-0.472
Q9NP97	Dynein light chain roadblock-type 1 OS=Homo sapiens GN=DYNLRB1 PE=1 SV=3 - [DLRB1_HUMAN]	38.54	1	2	2	6	24.08	23.24	23.82	24.18	24.60		3	2	24.24	23.31	23.88	23.93	24.82		23.81	24.38	0.323	0.119	0.568
O15173	Membrane-associated progesterone receptor component 2 OS=Homo sapiens GN=PGRMC2 PE=1 SV=1 - [PGRC2_HUMAN]	16.59	1	2	3	9		24.29	24.03	23.80	24.33	24.58	2	3		24.36	24.09	23.56	24.55	24.32	24.22	24.14	0.848	0.251	-0.084
P60981	Destrin OS=Homo sapiens GN=DSTN PE=1 SV=3 - [DEST_HUMAN]	66.06	1	9	10	55	25.95	25.95	25.66	26.00	25.86	26.54	3	3	26.11	26.02	25.72	25.75	26.07	26.28	25.95	26.04	0.683	0.215	0.086
A6NCE7	Microtubule-associated proteins 1A/1B light chain 3 beta 2 OS=Homo sapiens GN=MAP1LC3B2 PE=2 SV=1 - [MP3B2_HUMAN]	30.40	3	4	4	17	25.38	25.93	26.05	25.94	24.74	25.22	3	3	25.54	26.00	26.11	25.70	24.96	24.96	25.88	25.20	0.086	0.045	-0.679
P05106	Integrin beta-3 OS=Homo sapiens GN=ITGB3 PE=1 SV=2 - [ITB3_HUMAN]	11.17	1	8	8	19	22.80	22.89	23.56	23.05	21.54	23.36	3	3	22.96	22.96	23.62	22.81	21.75	23.10	23.18	22.55	0.246	0.097	-0.628
Q9Y3B3	Transmembrane emp24 domain-containing protein 7 OS=Homo sapiens GN=TMED7 PE=1 SV=2 - [TMED7_HUMAN]	29.02	1	5	5	25	23.84	23.91	22.87	23.90	23.73	24.89	3	3	24.00	23.98	22.92	23.65	23.94	24.63	23.63	24.08	0.390	0.137	0.441
P15151	Poliovirus receptor OS=Homo sapiens GN=PVR PE=1 SV=2 - [PVR_HUMAN]	18.71	1	6	6	44	25.01	25.70	25.74	25.38	23.76	25.44	3	3	25.17	25.77	25.80	25.14	23.98	25.18	25.58	24.77	0.140	0.064	-0.815
Q02241	Kinesin-like protein KIF23 OS=Homo sapiens GN=KIF23 PE=1 SV=3 - [KIF23_HUMAN]	50.63	1	38	39	187	26.30	25.46	26.02	26.22	25.18	26.10	3	3	26.46	25.53	26.08	25.98	25.39	25.84	26.03	25.74	0.421	0.146	-0.289
Q12913	Receptor-type tyrosine-protein phosphatase eta OS=Homo sapiens GN=PTPRJ PE=1 SV=3 - [PTPRJ_HUMAN]	15.41	1	12	12	43	23.42	23.08	23.16	24.57	22.35	24.52	3	3	23.58	23.15	23.21	24.33	22.57	24.25	23.31	23.72	0.531	0.178	0.403
Q13362	Serine/threonine-protein phosphatase 2A 56 kDa regulatory subunit gamma isoform OS=Homo sapiens GN=PPP2R5C PE=1 SV=3 - [2A5G_HUMAN]	8.02	1	1	3	11		23.14	22.04	23.82	23.24	23.82	2	3		23.21	22.10	23.58	23.46	23.56	22.65	23.53	0.126	0.059	0.879
Q9Y3B4	Splicing factor 3B subunit 6 OS=Homo sapiens GN=SF3B6 PE=1 SV=1 - [SF3B6_HUMAN]	20.80	1	2	2	7	23.16	23.31	23.50		23.52	23.51	3	2	23.32	23.38	23.56		23.74	23.25	23.42	23.49	0.743	0.229	0.073
P29992	Guanine nucleotide-binding protein subunit alpha-11 OS=Homo sapiens GN=GNA11 PE=1 SV=2 - [GNA11_HUMAN]	54.87	3	9	16	97	25.84	25.57	25.58	26.03	24.50	25.45	3	3	26.00	25.64	25.64	25.78	24.71	25.19	25.76	25.23	0.187	0.079	-0.529
Q9Y6B6	GTP-binding protein SAR1b OS=Homo sapiens GN=SAR1B PE=1 SV=1 - [SAR1B_HUMAN]	36.36	1	2	6	19	24.96	23.62	22.96	24.46	24.08	24.88	3	3	25.12	23.69	23.02	24.22	24.29	24.61	23.94	24.37	0.534	0.179	0.430
Q8NC42	E3 ubiquitin-protein ligase RNF149 OS=Homo sapiens GN=RNF149 PE=2 SV=2 - [RN149_HUMAN]	12.50	1	3	3	8	24.10	24.12	23.40	23.85	24.32	24.59	3	3	24.26	24.19	23.46	23.60	24.54	24.33	23.97	24.16	0.653	0.209	0.186
Q9H6X2	Anthrax toxin receptor 1 OS=Homo sapiens GN=ANTXR1 PE=1 SV=2 - [ANTR1_HUMAN]	6.21	1	1	2	7	22.41	22.79	22.17	22.43		22.64	3	2	22.57	22.86	22.23	22.19		22.38	22.56	22.28	0.351	0.127	-0.272
P52294	Importin subunit alpha-5 OS=Homo sapiens GN=KPNA1 PE=1 SV=3 - [IMA5_HUMAN]	16.54	1	2	6	30	23.04	23.60	23.61	23.79	23.19	24.22	3	3	23.21	23.67	23.67	23.55	23.41	23.96	23.51	23.64	0.611	0.198	0.125
CON_007104904	SWISS-PROT:P01030| Fragment	20.87	1	9	11	28	23.64		22.54	23.67	23.23	23.45	2	3	23.80		22.60	23.43	23.45	23.19	23.20	23.35	0.756	0.231	0.157
P17174	Aspartate aminotransferase, cytoplasmic OS=Homo sapiens GN=GOT1 PE=1 SV=3 - [AATC_HUMAN]	48.18	1	13	13	32	21.92	22.91		24.56	23.01	24.56	2	3	22.08	22.98		24.32	23.22	24.30	22.53	23.95	0.091	0.047	1.413
Q8NG11	Tetraspanin-14 OS=Homo sapiens GN=TSPAN14 PE=1 SV=1 - [TSN14_HUMAN]	15.93	1	4	4	21	25.04	25.13	25.47	25.37	23.73	25.22	3	3	25.20	25.20	25.53	25.12	23.94	24.96	25.31	24.68	0.176	0.076	-0.634
Q9UBM7	7-dehydrocholesterol reductase OS=Homo sapiens GN=DHCR7 PE=1 SV=1 - [DHCR7_HUMAN]	2.74	1	1	1	5		22.18	22.80	21.05	20.48	22.74	2	3		22.25	22.86	20.80	20.70	22.48	22.55	21.33	0.214	0.088	-1.226
P51153	Ras-related protein Rab-13 OS=Homo sapiens GN=RAB13 PE=1 SV=1 - [RAB13_HUMAN]	59.61	2	8	11	54	26.80	27.23	27.21	26.94	26.35	27.15	3	3	26.96	27.30	27.27	26.69	26.57	26.89	27.18	26.72	0.032	0.022	-0.461
Q53GA4	Pleckstrin homology-like domain family A member 2 OS=Homo sapiens GN=PHLDA2 PE=1 SV=2 - [PHLA2_HUMAN]	18.42	1	3	3	8	23.38	23.26	22.93	24.02	22.90	23.48	3	3	23.54	23.33	22.99	23.78	23.11	23.22	23.29	23.37	0.767	0.234	0.083
P52434	DNA-directed RNA polymerases I, II, and III subunit RPABC3 OS=Homo sapiens GN=POLR2H PE=1 SV=4 - [RPAB3_HUMAN]	14.67	1	3	3	13	22.06		21.42	22.12	22.85	23.10	2	3	22.22		21.48	21.87	23.06	22.84	21.85	22.59	0.270	0.103	0.740
Q15642	Cdc42-interacting protein 4 OS=Homo sapiens GN=TRIP10 PE=1 SV=3 - [CIP4_HUMAN]	35.77	1	17	17	79	24.30	24.23	24.02	24.19	23.70	24.16	3	3	24.46	24.30	24.08	23.94	23.92	23.90	24.28	23.92	0.033	0.023	-0.361
P12931	Proto-oncogene tyrosine-protein kinase Src OS=Homo sapiens GN=SRC PE=1 SV=3 - [SRC_HUMAN]	12.87	4	1	7	19	24.24	23.85	22.99	23.62	22.96	23.45	3	3	24.40	23.92	23.05	23.38	23.18	23.19	23.79	23.25	0.249	0.097	-0.543
O95166	Gamma-aminobutyric acid receptor-associated protein OS=Homo sapiens GN=GABARAP PE=1 SV=1 - [GBRAP_HUMAN]	34.19	2	3	4	18		23.51	23.72	23.01	23.08	23.72	2	3		23.58	23.78	22.77	23.30	23.46	23.68	23.17	0.167	0.073	-0.506
O60784	Target of Myb protein 1 OS=Homo sapiens GN=TOM1 PE=1 SV=2 - [TOM1_HUMAN]	5.89	1	2	2	6	21.94	21.90		22.87	21.92	22.21	2	3	22.10	21.97		22.62	22.13	21.95	22.04	22.23	0.512	0.173	0.195
P09972	Fructose-bisphosphate aldolase C OS=Homo sapiens GN=ALDOC PE=1 SV=2 - [ALDOC_HUMAN]	15.66	2	1	3	23	25.53	26.62	25.22	25.69	26.31	26.67	3	3	25.69	26.69	25.28	25.44	26.53	26.41	25.89	26.13	0.676	0.214	0.244
O14672	Disintegrin and metalloproteinase domain-containing protein 10 OS=Homo sapiens GN=ADAM10 PE=1 SV=1 - [ADA10_HUMAN]	30.61	1	22	22	117	24.90	25.65	25.84	25.45	23.94	25.37	3	3	25.06	25.72	25.90	25.21	24.16	25.11	25.56	24.82	0.155	0.069	-0.735
CON_007006224	SWISS-PROT:Q9TTE1|TREMBL:Q27983;Q28921 Bos taurus Gene_Symbol=SERPINA3 Serpin A3-1	25.30	2	9	9	26	24.80	23.16		24.32	23.15	22.78	2	3	24.96	23.23		24.08	23.37	22.52	24.09	23.32	0.440	0.152	-0.771
P06396	Gelsolin OS=Homo sapiens GN=GSN PE=1 SV=1 - [GELS_HUMAN]	12.92	1	9	9	31	23.76	23.53	24.06	23.65	23.45	25.58	3	3	23.92	23.60	24.12	23.41	23.67	25.31	23.88	24.13	0.706	0.220	0.249
Q6P9B6	TLD domain-containing protein 1 OS=Homo sapiens GN=TLDC1 PE=1 SV=2 - [TLDC1_HUMAN]	31.36	1	9	9	36	23.88	24.12	23.85	23.95	22.57	23.97	3	3	24.04	24.19	23.91	23.71	22.79	23.71	24.04	23.40	0.112	0.054	-0.643
Q96I99	Succinyl-CoA ligase [GDP-forming] subunit beta, mitochondrial OS=Homo sapiens GN=SUCLG2 PE=1 SV=2 - [SUCB2_HUMAN]	11.34	1	5	5	12	22.34	22.15	22.05		22.14	22.45	3	2	22.50	22.21	22.11		22.36	22.19	22.27	22.27	0.995	0.281	-0.001
P05023	Sodium/potassium-transporting ATPase subunit alpha-1 OS=Homo sapiens GN=ATP1A1 PE=1 SV=1 - [AT1A1_HUMAN]	49.95	6	49	49	395	28.17	28.21	27.99	29.25	27.54	28.95	3	3	28.33	28.28	28.05	29.00	27.76	28.69	28.22	28.48	0.531	0.178	0.263
P01130	Low-density lipoprotein receptor OS=Homo sapiens GN=LDLR PE=1 SV=1 - [LDLR_HUMAN]	24.88	1	14	14	89	24.20	24.53	24.14	25.23	24.09	24.58	3	3	24.36	24.60	24.20	24.98	24.31	24.32	24.39	24.54	0.579	0.190	0.151
Q9P289	Serine/threonine-protein kinase 26 OS=Homo sapiens GN=STK26 PE=1 SV=2 - [STK26_HUMAN]	20.43	1	4	7	17	23.09	23.09	21.53	24.06	22.30	22.95	3	3	23.25	23.16	21.59	23.81	22.52	22.69	22.67	23.01	0.640	0.206	0.340
P51148	Ras-related protein Rab-5C OS=Homo sapiens GN=RAB5C PE=1 SV=2 - [RAB5C_HUMAN]	72.22	1	7	10	88	26.69	26.99	26.61	27.32	26.63	27.07	3	3	26.85	27.06	26.67	27.07	26.84	26.81	26.86	26.91	0.751	0.230	0.047
P49366	Deoxyhypusine synthase OS=Homo sapiens GN=DHPS PE=1 SV=1 - [DHYS_HUMAN]	33.88	1	6	6	13	22.06		21.78	22.05	22.07	22.70	2	3	22.23		21.84	21.80	22.28	22.44	22.03	22.18	0.651	0.208	0.145
P62328	Thymosin beta-4 OS=Homo sapiens GN=TMSB4X PE=1 SV=2 - [TYB4_HUMAN]	25.00	1	1	1	6		28.63	28.70	27.97	27.64	28.72	2	3		28.70	28.76	27.73	27.86	28.46	28.73	28.01	0.092	0.047	-0.713
Q8IWA5	Choline transporter-like protein 2 OS=Homo sapiens GN=SLC44A2 PE=1 SV=3 - [CTL2_HUMAN]	3.68	1	3	3	8	23.35	25.02	24.87	23.99		24.85	3	2	23.51	25.09	24.93	23.74		24.59	24.51	24.17	0.664	0.212	-0.345
Q6DD88	Atlastin-3 OS=Homo sapiens GN=ATL3 PE=1 SV=1 - [ATLA3_HUMAN]	18.67	1	7	7	18	23.26	23.61	21.56		23.53	24.04	3	2	23.42	23.68	21.62		23.75	23.77	22.91	23.76	0.382	0.135	0.854
P13804	Electron transfer flavoprotein subunit alpha, mitochondrial OS=Homo sapiens GN=ETFA PE=1 SV=1 - [ETFA_HUMAN]	29.13	1	7	7	23	21.98	21.95	21.55	21.20	22.97	22.77	3	3	22.14	22.01	21.61	20.96	23.18	22.51	21.92	22.22	0.684	0.216	0.298
CON_007126931	SWISS-PROT:Q03247 Bos taurus Apolipoprotein E precursor	50.63	2	17	17	66	25.82	24.00	23.69	25.35	24.63	24.40	3	3	25.98	24.07	23.75	25.11	24.85	24.14	24.60	24.70	0.901	0.261	0.100
P15153	Ras-related C3 botulinum toxin substrate 2 OS=Homo sapiens GN=RAC2 PE=1 SV=1 - [RAC2_HUMAN]	55.73	3	5	10	48	26.76	27.22	27.33	27.02	25.73	27.32	3	3	26.92	27.29	27.39	26.77	25.95	27.06	27.20	26.59	0.168	0.074	-0.607
P16070	CD44 antigen OS=Homo sapiens GN=CD44 PE=1 SV=3 - [CD44_HUMAN]	11.59	1	8	8	67	28.01	28.73	28.66	28.58	27.21	28.24	3	3	28.17	28.80	28.72	28.33	27.43	27.98	28.56	27.91	0.120	0.057	-0.651
O15427	Monocarboxylate transporter 4 OS=Homo sapiens GN=SLC16A3 PE=1 SV=1 - [MOT4_HUMAN]	12.26	1	6	6	36	26.07	26.07	26.17	26.15	24.29	26.40	3	3	26.23	26.14	26.23	25.91	24.51	26.14	26.20	25.52	0.253	0.099	-0.682
Q14160	Protein scribble homolog OS=Homo sapiens GN=SCRIB PE=1 SV=4 - [SCRIB_HUMAN]	17.85	1	15	17	53	24.13	23.30	23.34	24.52	22.88	24.23	3	3	24.29	23.37	23.40	24.27	23.10	23.97	23.69	23.78	0.852	0.251	0.093
Q53GQ0	Very-long-chain 3-oxoacyl-CoA reductase OS=Homo sapiens GN=HSD17B12 PE=1 SV=2 - [DHB12_HUMAN]	28.53	1	6	6	14	22.11	24.02	24.19		23.11	24.24	3	2	22.27	24.09	24.25		23.32	23.98	23.54	23.65	0.903	0.261	0.114
O95819	Mitogen-activated protein kinase kinase kinase kinase 4 OS=Homo sapiens GN=MAP4K4 PE=1 SV=2 - [M4K4_HUMAN]	12.03	1	6	11	29	22.90	22.59	22.03	22.98	19.92	22.43	3	3	23.06	22.66	22.09	22.73	20.14	22.17	22.60	21.68	0.332	0.122	-0.923
P60953	Cell division control protein 42 homolog OS=Homo sapiens GN=CDC42 PE=1 SV=2 - [CDC42_HUMAN]	62.83	3	9	10	66	27.57	28.06	27.72	27.75	26.73	27.78	3	3	27.73	28.13	27.78	27.50	26.95	27.52	27.88	27.32	0.070	0.038	-0.556
O00233	26S proteasome non-ATPase regulatory subunit 9 OS=Homo sapiens GN=PSMD9 PE=1 SV=3 - [PSMD9_HUMAN]	31.84	1	6	6	15	22.69	26.19	25.93	24.12	23.63	24.38	3	3	22.85	26.25	25.99	23.88	23.85	24.12	25.03	23.95	0.379	0.135	-1.081
Q01085	Nucleolysin TIAR OS=Homo sapiens GN=TIAL1 PE=1 SV=1 - [TIAR_HUMAN]	11.20	1	1	3	8	23.81	22.98	23.44	23.42	23.10	23.37	3	3	23.97	23.05	23.50	23.18	23.32	23.11	23.51	23.20	0.330	0.122	-0.305
O94760	N(G),N(G)-dimethylarginine dimethylaminohydrolase 1 OS=Homo sapiens GN=DDAH1 PE=1 SV=3 - [DDAH1_HUMAN]	68.42	1	13	14	58	23.05	24.44	24.13	24.73	23.84	24.51	3	3	23.21	24.51	24.19	24.48	24.06	24.24	23.97	24.26	0.514	0.173	0.292
P41743	Protein kinase C iota type OS=Homo sapiens GN=PRKCI PE=1 SV=2 - [KPCI_HUMAN]	20.13	1	6	6	15	22.15	24.92	22.40	25.40	22.82	24.95	3	3	22.31	24.99	22.46	25.15	23.04	24.69	23.25	24.29	0.391	0.138	1.039
P54709	Sodium/potassium-transporting ATPase subunit beta-3 OS=Homo sapiens GN=ATP1B3 PE=1 SV=1 - [AT1B3_HUMAN]	36.20	1	8	8	39	24.98	24.84	25.43	26.29	24.19	26.03	3	3	25.14	24.91	25.49	26.05	24.40	25.77	25.18	25.41	0.696	0.218	0.224
Q8TBC3	SH3KBP1-binding protein 1 OS=Homo sapiens GN=SHKBP1 PE=1 SV=2 - [SHKB1_HUMAN]	5.80	1	2	2	7	22.66	22.30		22.45	22.31	22.97	2	3	22.82	22.37		22.21	22.53	22.71	22.59	22.48	0.687	0.216	-0.113
P07195	L-lactate dehydrogenase B chain OS=Homo sapiens GN=LDHB PE=1 SV=2 - [LDHB_HUMAN]	59.28	2	19	21	167	27.97	27.59	27.51	28.30	27.10	28.72	3	3	28.13	27.66	27.57	28.06	27.32	28.46	27.79	27.95	0.695	0.218	0.158
P84085	ADP-ribosylation factor 5 OS=Homo sapiens GN=ARF5 PE=1 SV=2 - [ARF5_HUMAN]	75.00	4	4	11	69	27.17	27.38	27.46	27.24	26.55	27.56	3	3	27.33	27.45	27.52	26.99	26.76	27.30	27.43	27.02	0.066	0.036	-0.414
P14174	Macrophage migration inhibitory factor OS=Homo sapiens GN=MIF PE=1 SV=4 - [MIF_HUMAN]	35.65	1	3	3	18	26.07	27.93	27.74	27.98	27.54	27.75	3	3	26.23	28.00	27.80	27.74	27.76	27.49	27.34	27.66	0.605	0.197	0.318
CON_004203124	SWISS-PROT:P07744 Tax_Id=10090 Gene_Symbol=Krt4 Keratin, type II cytoskeletal 4	10.10	6	2	8	24	24.54	24.16	25.91	24.72	26.76	25.23	3	3	24.70	24.22	25.97	24.48	26.97	24.97	24.96	25.47	0.610	0.198	0.510
P23458	Tyrosine-protein kinase JAK1 OS=Homo sapiens GN=JAK1 PE=1 SV=2 - [JAK1_HUMAN]	12.91	1	10	10	23	22.27	22.63	22.48	23.56	22.09	22.86	3	3	22.43	22.70	22.54	23.31	22.31	22.60	22.56	22.74	0.582	0.190	0.185
P06493	Cyclin-dependent kinase 1 OS=Homo sapiens GN=CDK1 PE=1 SV=3 - [CDK1_HUMAN]	47.14	20	11	12	46	25.43	24.78	25.08	25.29	24.46	25.23	3	3	25.59	24.85	25.14	25.05	24.68	24.97	25.19	24.90	0.292	0.110	-0.293
Q15286	Ras-related protein Rab-35 OS=Homo sapiens GN=RAB35 PE=1 SV=1 - [RAB35_HUMAN]	72.64	8	11	13	85	27.67	28.18	28.20	27.87	27.34	28.16	3	3	27.83	28.25	28.26	27.63	27.56	27.90	28.11	27.70	0.077	0.041	-0.418
P16188	HLA class I histocompatibility antigen, A-30 alpha chain OS=Homo sapiens GN=HLA-A PE=1 SV=2 - [1A30_HUMAN]	33.70	6	0	9	44	27.90	27.74	27.79	28.57	27.20	28.56	3	3	28.06	27.81	27.85	28.32	27.42	28.30	27.91	28.01	0.750	0.230	0.105
P47755	F-actin-capping protein subunit alpha-2 OS=Homo sapiens GN=CAPZA2 PE=1 SV=3 - [CAZA2_HUMAN]	33.92	1	4	6	26	25.07	24.93	24.37	24.85	23.89	24.76	3	3	25.23	25.00	24.43	24.61	24.11	24.50	24.89	24.40	0.160	0.071	-0.484
Q86YZ3	Hornerin OS=Homo sapiens GN=HRNR PE=1 SV=2 - [HORN_HUMAN]	5.96	1	3	3	16	19.86	18.60	18.75	19.74	20.00		3	2	20.02	18.67	18.81	19.49	20.21		19.17	19.85	0.347	0.126	0.685
Q13637	Ras-related protein Rab-32 OS=Homo sapiens GN=RAB32 PE=1 SV=3 - [RAB32_HUMAN]	10.67	1	2	2	7	22.72	23.28	21.65	22.59		22.99	3	2	22.88	23.35	21.71	22.35		22.73	22.64	22.54	0.884	0.257	-0.103
P02538	Keratin, type II cytoskeletal 6A OS=Homo sapiens GN=KRT6A PE=1 SV=3 - [K2C6A_HUMAN]	25.35	7	6	18	53	25.47	24.05	26.03	25.71	26.82	25.23	3	3	25.63	24.12	26.09	25.46	27.04	24.97	25.28	25.82	0.562	0.186	0.543
P08133	Annexin A6 OS=Homo sapiens GN=ANXA6 PE=1 SV=3 - [ANXA6_HUMAN]	70.73	2	54	55	443	27.72	28.38	28.17	28.64	28.00	28.41	3	3	27.88	28.45	28.22	28.40	28.21	28.15	28.19	28.25	0.727	0.225	0.067
O43854	EGF-like repeat and discoidin I-like domain-containing protein 3 OS=Homo sapiens GN=EDIL3 PE=1 SV=1 - [EDIL3_HUMAN]	62.71	1	29	29	257	28.93	29.63	29.63	29.17	28.66	29.60	3	3	29.09	29.70	29.69	28.93	28.88	29.34	29.50	29.05	0.147	0.066	-0.446
O15127	Secretory carrier-associated membrane protein 2 OS=Homo sapiens GN=SCAMP2 PE=1 SV=2 - [SCAM2_HUMAN]	14.29	1	3	3	15	23.44	23.59	23.25	23.48		22.81	3	2	23.60	23.66	23.31	23.24		22.55	23.53	22.90	0.119	0.056	-0.629
P21291	Cysteine and glycine-rich protein 1 OS=Homo sapiens GN=CSRP1 PE=1 SV=3 - [CSRP1_HUMAN]	55.96	1	8	8	37	23.27	24.27	24.12	23.29	24.03	25.13	3	3	23.43	24.34	24.18	23.05	24.25	24.87	23.99	24.06	0.913	0.263	0.070
O43813	LanC-like protein 1 OS=Homo sapiens GN=LANCL1 PE=1 SV=1 - [LANC1_HUMAN]	14.04	1	4	4	11	22.12	22.83	22.60	22.39		22.57	3	2	22.28	22.90	22.66	22.15		22.31	22.61	22.23	0.211	0.087	-0.385
Q9NZV1	Cysteine-rich motor neuron 1 protein OS=Homo sapiens GN=CRIM1 PE=1 SV=1 - [CRIM1_HUMAN]	8.98	1	6	6	14	22.85	22.77	21.73	23.58	22.13	22.66	3	3	23.01	22.84	21.79	23.33	22.35	22.40	22.55	22.69	0.785	0.238	0.146
O96019	Actin-like protein 6A OS=Homo sapiens GN=ACTL6A PE=1 SV=1 - [ACL6A_HUMAN]	19.35	2	5	5	22	23.50	23.71	23.79	23.92	23.74	23.67	3	3	23.66	23.78	23.85	23.68	23.96	23.41	23.76	23.68	0.653	0.209	-0.082
Q9UHY1	Nuclear receptor-binding protein OS=Homo sapiens GN=NRBP1 PE=1 SV=1 - [NRBP_HUMAN]	5.98	1	1	1	7		22.35	21.93	22.33	22.23	23.19	2	3		22.42	21.99	22.08	22.44	22.93	22.21	22.49	0.489	0.167	0.280
P05161	Ubiquitin-like protein ISG15 OS=Homo sapiens GN=ISG15 PE=1 SV=5 - [ISG15_HUMAN]	35.15	1	5	5	14	24.15	24.61	21.51		24.30	24.54	3	2	24.31	24.68	21.57		24.52	24.28	23.52	24.40	0.538	0.180	0.880
P31641	Sodium- and chloride-dependent taurine transporter OS=Homo sapiens GN=SLC6A6 PE=1 SV=2 - [SC6A6_HUMAN]	2.74	1	1	1	9	23.34	23.63		23.50	22.05	23.47	2	3	23.50	23.70		23.26	22.26	23.21	23.60	22.91	0.201	0.084	-0.692
P17931	Galectin-3 OS=Homo sapiens GN=LGALS3 PE=1 SV=5 - [LEG3_HUMAN]	23.20	1	5	5	23	23.83	24.05	24.03	24.11	22.92	24.02	3	3	24.00	24.12	24.09	23.86	23.13	23.76	24.07	23.58	0.103	0.051	-0.485
P09913	Interferon-induced protein with tetratricopeptide repeats 2 OS=Homo sapiens GN=IFIT2 PE=1 SV=1 - [IFIT2_HUMAN]	2.75	1	1	1	11	20.32	19.91	20.50	19.27	19.61	20.96	3	3	20.48	19.98	20.56	19.02	19.83	20.70	20.34	19.85	0.395	0.139	-0.492
Q92900	Regulator of nonsense transcripts 1 OS=Homo sapiens GN=UPF1 PE=1 SV=2 - [RENT1_HUMAN]	13.82	1	11	11	22	21.77	23.31	23.54	22.85		23.42	3	2	21.93	23.38	23.59	22.60		23.16	22.97	22.88	0.911	0.263	-0.085
P20340	Ras-related protein Rab-6A OS=Homo sapiens GN=RAB6A PE=1 SV=3 - [RAB6A_HUMAN]	41.83	8	7	8	41	26.83	27.30	27.66	27.16	26.64	27.41	3	3	26.99	27.37	27.72	26.91	26.86	27.15	27.36	26.97	0.164	0.072	-0.386
CON_007107991	SWISS-PROT:P00735 Bos taurus Prothrombin precursor Fragment	46.88	3	23	23	165	28.21	26.76	27.56	27.65	27.39	28.37	3	3	28.37	26.83	27.62	27.40	27.60	28.11	27.61	27.70	0.853	0.251	0.097
P13647	Keratin, type II cytoskeletal 5 OS=Homo sapiens GN=KRT5 PE=1 SV=3 - [K2C5_HUMAN]	17.29	6	4	14	45	25.41	24.05	26.03	25.58	26.82	25.23	3	3	25.57	24.12	26.09	25.33	27.04	24.97	25.26	25.78	0.580	0.190	0.522
Q08722	Leukocyte surface antigen CD47 OS=Homo sapiens GN=CD47 PE=1 SV=1 - [CD47_HUMAN]	8.67	1	3	3	12	25.47	25.65	25.68	26.04	24.14	25.40	3	3	25.63	25.71	25.74	25.80	24.35	25.14	25.69	25.10	0.226	0.091	-0.598
P61224	Ras-related protein Rap-1b OS=Homo sapiens GN=RAP1B PE=1 SV=1 - [RAP1B_HUMAN]	80.43	2	5	13	86	27.51	27.74	27.60	27.69	26.54	27.71	3	3	27.67	27.81	27.66	27.44	26.75	27.45	27.71	27.22	0.104	0.051	-0.496
Q8N6T3	ADP-ribosylation factor GTPase-activating protein 1 OS=Homo sapiens GN=ARFGAP1 PE=1 SV=2 - [ARFG1_HUMAN]	3.45	1	1	1	5	20.70	21.31		22.38	21.11	22.10	2	3	20.86	21.38		22.13	21.33	21.84	21.12	21.77	0.171	0.075	0.646
Q5SSJ5	Heterochromatin protein 1-binding protein 3 OS=Homo sapiens GN=HP1BP3 PE=1 SV=1 - [HP1B3_HUMAN]	16.46	1	8	8	16	21.50	23.29		23.60	23.03	22.70	2	3	21.66	23.36		23.35	23.25	22.44	22.51	23.01	0.542	0.181	0.503
P63279	SUMO-conjugating enzyme UBC9 OS=Homo sapiens GN=UBE2I PE=1 SV=1 - [UBC9_HUMAN]	53.16	1	6	6	17	21.23	23.19	23.09	22.98	21.95	24.44	3	3	21.39	23.26	23.15	22.73	22.16	24.18	22.60	23.02	0.644	0.207	0.424
P21266	Glutathione S-transferase Mu 3 OS=Homo sapiens GN=GSTM3 PE=1 SV=3 - [GSTM3_HUMAN]	22.67	1	4	4	23	22.51	22.98	22.77	22.93	22.52	23.54	3	3	22.67	23.05	22.83	22.68	22.73	23.28	22.85	22.90	0.836	0.248	0.049
Q13492	Phosphatidylinositol-binding clathrin assembly protein OS=Homo sapiens GN=PICALM PE=1 SV=2 - [PICAL_HUMAN]	16.87	2	9	9	25	23.99	23.93	23.45	22.69		23.62	3	2	24.15	24.00	23.51	22.45		23.36	23.89	22.90	0.101	0.051	-0.988
P14618	Pyruvate kinase PKM OS=Homo sapiens GN=PKM PE=1 SV=4 - [KPYM_HUMAN]	76.46	2	38	38	405	30.12	30.02	29.85	30.00	29.24	30.14	3	3	30.28	30.09	29.91	29.76	29.45	29.88	30.09	29.70	0.075	0.040	-0.396
P02795	Metallothionein-2 OS=Homo sapiens GN=MT2A PE=1 SV=1 - [MT2_HUMAN]	52.46	6	2	2	8	25.22	27.47	27.00	26.92	19.90	27.27	3	3	25.38	27.54	27.06	26.68	20.12	27.01	26.66	24.60	0.428	0.149	-2.060
Q9NRX5	Serine incorporator 1 OS=Homo sapiens GN=SERINC1 PE=1 SV=1 - [SERC1_HUMAN]	8.61	1	3	3	20	25.07	25.30	25.18	25.57	23.61	25.12	3	3	25.23	25.37	25.24	25.32	23.83	24.86	25.28	24.67	0.241	0.095	-0.610
Q12959	Disks large homolog 1 OS=Homo sapiens GN=DLG1 PE=1 SV=2 - [DLG1_HUMAN]	33.30	4	18	18	72	24.25	24.88	24.70	24.65	23.72	24.59	3	3	24.41	24.95	24.76	24.41	23.94	24.33	24.71	24.23	0.088	0.045	-0.482
Q96TA1	Niban-like protein 1 OS=Homo sapiens GN=FAM129B PE=1 SV=3 - [NIBL1_HUMAN]	40.21	1	22	22	95	25.34	24.76	24.94	25.06	23.67	25.44	3	3	25.51	24.83	25.00	24.82	23.89	25.18	25.11	24.63	0.332	0.122	-0.480
P06744	Glucose-6-phosphate isomerase OS=Homo sapiens GN=GPI PE=1 SV=4 - [G6PI_HUMAN]	39.61	2	14	19	99	25.40	25.22	25.36	25.75	24.76	26.15	3	3	25.56	25.29	25.42	25.50	24.98	25.88	25.42	25.46	0.914	0.263	0.032
Q13610	Periodic tryptophan protein 1 homolog OS=Homo sapiens GN=PWP1 PE=1 SV=1 - [PWP1_HUMAN]	17.37	1	5	5	10	21.31	21.25	23.35		23.04	23.07	3	2	21.48	21.32	23.41		23.26	22.81	22.07	23.03	0.355	0.128	0.966
P48509	CD151 antigen OS=Homo sapiens GN=CD151 PE=1 SV=3 - [CD151_HUMAN]	13.04	1	4	4	9	25.67	25.54	26.56	28.40	24.70	26.65	3	3	25.83	25.61	26.62	28.16	24.92	26.39	26.02	26.49	0.659	0.210	0.469
Q14738	Serine/threonine-protein phosphatase 2A 56 kDa regulatory subunit delta isoform OS=Homo sapiens GN=PPP2R5D PE=1 SV=1 - [2A5D_HUMAN]	11.13	1	2	4	13		23.14	22.04	23.63	22.80	24.23	2	3		23.21	22.10	23.38	23.01	23.97	22.65	23.45	0.242	0.095	0.799
P10114	Ras-related protein Rap-2a OS=Homo sapiens GN=RAP2A PE=1 SV=1 - [RAP2A_HUMAN]	49.73	1	3	7	42	25.62	25.54	25.25	25.41	24.30	25.76	3	3	25.79	25.61	25.31	25.17	24.52	25.50	25.57	25.06	0.191	0.080	-0.505
O60669	Monocarboxylate transporter 2 OS=Homo sapiens GN=SLC16A7 PE=1 SV=2 - [MOT2_HUMAN]	3.14	1	2	2	9	22.01	21.77	21.70	22.17		20.74	3	2	22.17	21.84	21.76	21.92		20.48	21.92	21.20	0.289	0.109	-0.723
Q9BTT6	Leucine-rich repeat-containing protein 1 OS=Homo sapiens GN=LRRC1 PE=1 SV=1 - [LRRC1_HUMAN]	9.35	1	1	3	10	22.99	22.72	22.45	22.87		23.18	3	2	23.15	22.79	22.51	22.62		22.92	22.82	22.77	0.865	0.254	-0.049
Q13526	Peptidyl-prolyl cis-trans isomerase NIMA-interacting 1 OS=Homo sapiens GN=PIN1 PE=1 SV=1 - [PIN1_HUMAN]	23.93	1	2	2	7		23.77	23.48	24.32	23.59	24.56	2	3		23.84	23.54	24.07	23.81	24.30	23.69	24.06	0.186	0.079	0.370
P10316	HLA class I histocompatibility antigen, A-69 alpha chain OS=Homo sapiens GN=HLA-A PE=1 SV=2 - [1A69_HUMAN]	55.62	2	0	18	145	28.04	28.00	28.07	28.80	27.37	28.77	3	3	28.20	28.07	28.13	28.55	27.59	28.50	28.14	28.22	0.812	0.243	0.080
P30419	Glycylpeptide N-tetradecanoyltransferase 1 OS=Homo sapiens GN=NMT1 PE=1 SV=2 - [NMT1_HUMAN]	27.82	2	8	8	24	24.11	23.62	22.71	24.39	22.99	24.14	3	3	24.27	23.68	22.77	24.14	23.21	23.87	23.58	23.74	0.767	0.234	0.164

Q99536	Synaptic vesicle membrane protein VAT-1 homolog OS=Homo sapiens GN=VAT1 PE=1 SV=2 - [VAT1_HUMAN]	56.23	2	14	14	61	26.09	26.03	24.57	26.26	25.23	26.82	3	3	26.25	26.10	24.62	26.02	25.45	26.56	25.66	26.01	0.597	0.195	0.350
P00749	Urokinase-type plasminogen activator OS=Homo sapiens GN=PLAU PE=1 SV=2 - [UROK_HUMAN]	53.60	1	19	19	124	24.62	25.23	25.47	24.86	24.41	25.48	3	3	24.78	25.30	25.53	24.61	24.63	25.22	25.20	24.82	0.266	0.103	-0.384
Q01628	Interferon-induced transmembrane protein 3 OS=Homo sapiens GN=IFITM3 PE=1 SV=2 - [IFM3_HUMAN]	48.87	4	3	3	30	24.94	26.57	26.20	27.45	25.45	26.69	3	3	25.10	26.64	26.26	27.20	25.67	26.43	26.00	26.43	0.537	0.179	0.431
P61006	Ras-related protein Rab-8A OS=Homo sapiens GN=RAB8A PE=1 SV=1 - [RAB8A_HUMAN]	57.97	11	5	13	100	27.56	28.08	28.09	27.79	27.32	28.09	3	3	27.72	28.15	28.15	27.54	27.54	27.83	28.01	27.64	0.097	0.049	-0.370
Q14254	Flotillin-2 OS=Homo sapiens GN=FLOT2 PE=1 SV=2 - [FLOT2_HUMAN]	37.15	1	13	13	46	23.78	23.66	22.70	24.31	22.84	24.18	3	3	23.94	23.73	22.75	24.07	23.05	23.92	23.48	23.68	0.694	0.218	0.204
P62834	Ras-related protein Rap-1A OS=Homo sapiens GN=RAP1A PE=1 SV=1 - [RAP1A_HUMAN]	76.63	1	3	11	66	27.33	27.56	27.46	27.63	26.44	27.51	3	3	27.50	27.63	27.52	27.38	26.66	27.25	27.55	27.10	0.117	0.056	-0.452
Q9C0J9	Class E basic helix-loop-helix protein 41 OS=Homo sapiens GN=BHLHE41 PE=1 SV=1 - [BHE41_HUMAN]	1.24	1	1	1	6	26.94	27.67	27.30	27.45	26.18	27.23	3	3	27.10	27.74	27.36	27.20	26.39	26.97	27.40	26.86	0.146	0.066	-0.545
Q9NUQ9	Protein FAM49B OS=Homo sapiens GN=FAM49B PE=1 SV=1 - [FA49B_HUMAN]	47.22	2	10	10	43	25.06	24.99	24.92	25.18	23.68	25.22	3	3	25.22	25.06	24.98	24.93	23.89	24.96	25.09	24.59	0.239	0.095	-0.493
Q8NFJ5	Retinoic acid-induced protein 3 OS=Homo sapiens GN=GPRC5A PE=1 SV=2 - [RAI3_HUMAN]	19.89	1	4	4	29	25.95	25.88	25.82	26.82	24.27	25.30	3	3	26.11	25.95	25.88	26.58	24.49	25.04	25.98	25.37	0.384	0.136	-0.613
Q7Z5G4	Golgin subfamily A member 7 OS=Homo sapiens GN=GOLGA7 PE=1 SV=2 - [GOGA7_HUMAN]	34.31	1	4	4	34	24.53	24.47	24.41	24.96	23.24	24.35	3	3	24.69	24.54	24.46	24.71	23.46	24.09	24.57	24.09	0.261	0.101	-0.481
Q02750	Dual specificity mitogen-activated protein kinase kinase 1 OS=Homo sapiens GN=MAP2K1 PE=1 SV=2 - [MP2K1_HUMAN]	37.91	2	10	10	52	24.59	24.02	23.95	24.26	24.17	24.66	3	3	24.75	24.09	24.01	24.01	24.39	24.40	24.28	24.27	0.949	0.271	-0.018
P78386	Keratin, type II cuticular Hb5 OS=Homo sapiens GN=KRT85 PE=1 SV=1 - [KRT85_HUMAN]	3.94	5	1	3	11	23.93		27.28	24.28	26.66	25.42	2	3	24.09		27.34	24.04	26.88	25.16	25.72	25.36	0.839	0.249	-0.356
P55263	Adenosine kinase OS=Homo sapiens GN=ADK PE=1 SV=2 - [ADK_HUMAN]	28.18	1	6	6	15	23.95	22.08	22.41	23.21	22.93	23.22	3	3	24.11	22.15	22.47	22.96	23.14	22.95	22.91	23.02	0.866	0.254	0.110
P08195	4F2 cell-surface antigen heavy chain OS=Homo sapiens GN=SLC3A2 PE=1 SV=3 - [4F2_HUMAN]	50.32	1	26	26	197	28.67	28.67	28.61	28.94	27.50	28.72	3	3	28.83	28.74	28.67	28.69	27.72	28.46	28.75	28.29	0.200	0.083	-0.458
Q13620	Cullin-4B OS=Homo sapiens GN=CUL4B PE=1 SV=4 - [CUL4B_HUMAN]	13.03	1	6	9	20	20.93	21.89		24.58	21.18	24.75	2	3	21.10	21.95		24.34	21.40	24.49	21.53	23.41	0.255	0.099	1.882
P48739	Phosphatidylinositol transfer protein beta isoform OS=Homo sapiens GN=PITPNB PE=1 SV=2 - [PIPNB_HUMAN]	42.44	2	8	8	20		23.47	23.61	23.80	21.51	23.99	2	3		23.54	23.67	23.55	21.73	23.73	23.61	23.00	0.518	0.174	-0.604
P52788	Spermine synthase OS=Homo sapiens GN=SMS PE=1 SV=2 - [SPSY_HUMAN]	56.28	1	11	11	34	23.68	23.76	24.01	24.43	23.43	24.14	3	3	23.85	23.83	24.07	24.18	23.64	23.88	23.91	23.90	0.938	0.268	-0.014
P40123	Adenylyl cyclase-associated protein 2 OS=Homo sapiens GN=CAP2 PE=1 SV=1 - [CAP2_HUMAN]	17.19	1	5	6	17	25.15	25.50	25.50	25.81	25.09	25.79	3	3	25.31	25.57	25.56	25.56	25.31	25.52	25.48	25.47	0.900	0.261	-0.016
Q9NS86	LanC-like protein 2 OS=Homo sapiens GN=LANCL2 PE=1 SV=1 - [LANC2_HUMAN]	2.89	1	2	2	8	20.23	21.47		21.30	21.26	21.46	2	3	20.39	21.54		21.06	21.47	21.20	20.97	21.24	0.587	0.192	0.278
P09758	Tumor-associated calcium signal transducer 2 OS=Homo sapiens GN=TACSTD2 PE=1 SV=3 - [TACD2_HUMAN]	36.22	1	9	9	64	25.42	25.78	25.72	26.32	25.10	26.34	3	3	25.58	25.85	25.78	26.07	25.31	26.08	25.74	25.82	0.767	0.234	0.085
Q15758	Neutral amino acid transporter B(0) OS=Homo sapiens GN=SLC1A5 PE=1 SV=2 - [AAAT_HUMAN]	21.26	1	10	10	67	27.60	27.41	27.43	28.76	26.76	27.80	3	3	27.76	27.48	27.49	28.52	26.98	27.54	27.57	27.68	0.831	0.247	0.105
Q8NCA5	Protein FAM98A OS=Homo sapiens GN=FAM98A PE=1 SV=1 - [FA98A_HUMAN]	12.14	1	4	4	12	22.52	23.24	23.62	22.66	22.54		3	2	22.68	23.31	23.68	22.41	22.75		23.22	22.58	0.208	0.086	-0.640
P18085	ADP-ribosylation factor 4 OS=Homo sapiens GN=ARF4 PE=1 SV=3 - [ARF4_HUMAN]	86.67	3	8	13	83	27.19	27.38	27.46	27.37	26.55	27.57	3	3	27.36	27.45	27.52	27.12	26.77	27.31	27.44	27.06	0.085	0.045	-0.377
P18465	HLA class I histocompatibility antigen, B-57 alpha chain OS=Homo sapiens GN=HLA-B PE=1 SV=1 - [1B57_HUMAN]	38.67	2	0	9	48	26.46	26.64	26.56	27.68	25.61	27.58	3	3	26.62	26.71	26.62	27.44	25.83	27.32	26.65	26.86	0.704	0.220	0.213
P10124	Serglycin OS=Homo sapiens GN=SRGN PE=1 SV=3 - [SRGN_HUMAN]	32.91	1	4	4	15	23.70	22.90	23.10	24.21	23.52	22.58	3	3	23.86	22.97	23.16	23.97	23.74	22.32	23.33	23.34	0.987	0.279	0.010
Q14699	Raftlin OS=Homo sapiens GN=RFTN1 PE=1 SV=4 - [RFTN1_HUMAN]	48.27	1	19	19	99	25.73	25.83	25.66	26.08	24.34	25.83	3	3	25.89	25.90	25.72	25.83	24.56	25.57	25.84	25.32	0.259	0.101	-0.516
CON_007041271	SWISS-PROT:Q95M17 Bos taurus Acidic mammalian chitinase precursor	22.46	2	7	7	13	22.23		22.05	21.74		24.25	2	2	22.39		22.11	21.50		23.98	22.25	22.74	0.734	0.227	0.489
Q9UBI6	Guanine nucleotide-binding protein G(I)/G(S)/G(O) subunit gamma-12 OS=Homo sapiens GN=GNG12 PE=1 SV=3 - [GBG12_HUMAN]	66.67	1	4	4	36	27.09	27.87	27.79	28.13	27.22	28.21	3	3	27.25	27.94	27.85	27.89	27.43	27.94	27.68	27.75	0.790	0.238	0.077
Q9UL26	Ras-related protein Rab-22A OS=Homo sapiens GN=RAB22A PE=1 SV=2 - [RB22A_HUMAN]	41.75	1	4	6	19	23.73	24.03	23.94	23.66		24.23	3	2	23.89	24.10	24.00	23.42		23.97	24.00	23.69	0.262	0.101	-0.304
O60488	Long-chain-fatty-acid--CoA ligase 4 OS=Homo sapiens GN=ACSL4 PE=1 SV=2 - [ACSL4_HUMAN]	55.84	1	28	30	114	25.77	25.68	25.58	25.89	25.27	26.47	3	3	25.93	25.75	25.64	25.65	25.49	26.21	25.77	25.78	0.966	0.275	0.011
P05534	HLA class I histocompatibility antigen, A-24 alpha chain OS=Homo sapiens GN=HLA-A PE=1 SV=2 - [1A24_HUMAN]	35.62	3	0	10	66	27.90	27.74	27.79	28.57	27.08	28.52	3	3	28.06	27.81	27.85	28.32	27.30	28.26	27.91	27.96	0.884	0.257	0.053
P04439	HLA class I histocompatibility antigen, A-3 alpha chain OS=Homo sapiens GN=HLA-A PE=1 SV=2 - [1A03_HUMAN]	33.42	7	0	9	43	27.90	27.74	27.79	28.57	27.08	28.52	3	3	28.06	27.81	27.85	28.32	27.30	28.26	27.91	27.96	0.884	0.257	0.053
P30825	High affinity cationic amino acid transporter 1 OS=Homo sapiens GN=SLC7A1 PE=1 SV=1 - [CTR1_HUMAN]	9.70	1	4	5	28	24.84	25.16	25.10	25.02	23.95	25.36	3	3	25.01	25.23	25.16	24.77	24.16	25.10	25.13	24.68	0.187	0.079	-0.450
P31946	14-3-3 protein beta/alpha OS=Homo sapiens GN=YWHAB PE=1 SV=3 - [1433B_HUMAN]	64.63	1	8	13	117	27.15	28.08	28.01	28.87	27.38	28.13	3	3	27.31	28.15	28.07	28.62	27.60	27.87	27.84	28.03	0.672	0.213	0.185
P61026	Ras-related protein Rab-10 OS=Homo sapiens GN=RAB10 PE=1 SV=1 - [RAB10_HUMAN]	64.00	8	9	13	84	27.63	28.15	28.15	27.89	27.37	28.17	3	3	27.79	28.22	28.21	27.64	27.59	27.91	28.07	27.71	0.104	0.051	-0.360
Q15599	Na(+)/H(+) exchange regulatory cofactor NHE-RF2 OS=Homo sapiens GN=SLC9A3R2 PE=1 SV=2 - [NHRF2_HUMAN]	16.62	1	5	5	16	22.67	23.77	23.06	24.03	22.82	23.73	3	3	22.83	23.84	23.12	23.78	23.04	23.47	23.26	23.43	0.673	0.213	0.168
Q9UQB8	Brain-specific angiogenesis inhibitor 1-associated protein 2 OS=Homo sapiens GN=BAIAP2 PE=1 SV=1 - [BAIP2_HUMAN]	4.71	1	2	2	6	22.08	22.66	22.51	22.17		22.10	3	2	22.24	22.73	22.57	21.93		21.84	22.51	21.89	0.046	0.028	-0.627
Q9P1F3	Costars family protein ABRACL OS=Homo sapiens GN=ABRACL PE=1 SV=1 - [ABRAL_HUMAN]	43.21	1	3	3	13	24.91	24.93	24.92	24.96	24.87	25.01	3	3	25.07	25.00	24.98	24.71	25.09	24.75	25.02	24.85	0.239	0.095	-0.171
P18206	Vinculin OS=Homo sapiens GN=VCL PE=1 SV=4 - [VINC_HUMAN]	51.32	1	44	44	199	25.27	26.01	25.81	25.93	25.39	26.43	3	3	25.43	26.08	25.87	25.69	25.60	26.17	25.79	25.82	0.926	0.266	0.025
P63313	Thymosin beta-10 OS=Homo sapiens GN=TMSB10 PE=1 SV=2 - [TYB10_HUMAN]	31.82	1	1	1	9	25.92	28.53	28.73	27.14	27.10	27.64	3	3	26.08	28.60	28.79	26.89	27.32	27.38	27.82	27.20	0.519	0.174	-0.626
P81605	Dermcidin OS=Homo sapiens GN=DCD PE=1 SV=2 - [DCD_HUMAN]	20.00	1	2	2	9	24.73	24.70	23.76	26.24	24.09	23.83	3	3	24.90	24.77	23.82	26.00	24.30	23.57	24.49	24.62	0.880	0.256	0.128
Q9UBQ0	Vacuolar protein sorting-associated protein 29 OS=Homo sapiens GN=VPS29 PE=1 SV=1 - [VPS29_HUMAN]	48.90	1	7	7	44	24.46	24.01	23.17	24.31	23.57	24.69	3	3	24.62	24.08	23.23	24.06	23.79	24.43	23.98	24.10	0.805	0.242	0.118
Q9Y6E0	Serine/threonine-protein kinase 24 OS=Homo sapiens GN=STK24 PE=1 SV=1 - [STK24_HUMAN]	24.83	1	5	8	25	23.33	23.09		24.14	22.87	23.52	2	3	23.49	23.16		23.89	23.09	23.26	23.32	23.41	0.805	0.242	0.091
P62070	Ras-related protein R-Ras2 OS=Homo sapiens GN=RRAS2 PE=1 SV=1 - [RRAS2_HUMAN]	49.02	1	7	10	50	25.38	25.59	25.51	25.44	24.40	25.91	3	3	25.54	25.66	25.57	25.20	24.62	25.65	25.59	25.16	0.221	0.090	-0.435
Q6NUK1	Calcium-binding mitochondrial carrier protein SCaMC-1 OS=Homo sapiens GN=SLC25A24 PE=1 SV=2 - [SCMC1_HUMAN]	15.30	1	6	6	10		22.36	21.40	21.76	21.70	23.83	2	3		22.43	21.46	21.51	21.92	23.57	21.94	22.33	0.691	0.217	0.389
P26022	Pentraxin-related protein PTX3 OS=Homo sapiens GN=PTX3 PE=1 SV=3 - [PTX3_HUMAN]	39.11	1	11	11	62	25.77	24.03	23.83	24.74	24.64	25.35	3	3	25.93	24.10	23.88	24.50	24.85	25.09	24.64	24.81	0.808	0.242	0.174
Q6PIU2	Neutral cholesterol ester hydrolase 1 OS=Homo sapiens GN=NCEH1 PE=1 SV=3 - [NCEH1_HUMAN]	23.04	1	6	6	19		23.16	22.71	23.26	22.83	23.88	2	3		23.23	22.77	23.02	23.05	23.62	23.00	23.23	0.509	0.172	0.228
P50148	Guanine nucleotide-binding protein G(q) subunit alpha OS=Homo sapiens GN=GNAQ PE=1 SV=4 - [GNAQ_HUMAN]	44.29	2	5	12	68	24.73	24.83	24.90	24.93	23.76	25.15	3	3	24.89	24.90	24.96	24.69	23.97	24.89	24.92	24.52	0.222	0.090	-0.402
P46939	Utrophin OS=Homo sapiens GN=UTRN PE=1 SV=2 - [UTRO_HUMAN]	2.62	1	6	6	16	24.27	24.64	25.07	24.32		24.89	3	2	24.43	24.71	25.13	24.07		24.63	24.76	24.35	0.310	0.115	-0.407
P35613	Basigin OS=Homo sapiens GN=BSG PE=1 SV=2 - [BASI_HUMAN]	30.91	1	9	9	75	27.34	27.74	27.78	27.71	27.29	28.28	3	3	27.50	27.81	27.83	27.46	27.51	28.02	27.72	27.66	0.814	0.243	-0.052
Q96BJ3	Axin interactor, dorsalization-associated protein OS=Homo sapiens GN=AIDA PE=1 SV=1 - [AIDA_HUMAN]	24.51	1	5	5	12	23.28	23.50	23.31	23.20		23.58	3	2	23.45	23.57	23.36	22.96		23.32	23.46	23.14	0.130	0.060	-0.320
O95239	Chromosome-associated kinesin KIF4A OS=Homo sapiens GN=KIF4A PE=1 SV=3 - [KIF4A_HUMAN]	12.01	4	11	12	33	22.49	21.76	23.53	23.08	22.71	22.73	3	3	22.65	21.83	23.59	22.84	22.92	22.47	22.69	22.74	0.923	0.265	0.054
P14621	Acylphosphatase-2 OS=Homo sapiens GN=ACYP2 PE=1 SV=2 - [ACYP2_HUMAN]	30.30	1	3	3	9	21.49	22.59	21.78	22.12	21.93	22.39	3	3	21.65	22.66	21.84	21.88	22.14	22.13	22.05	22.05	0.999	0.281	0.000
P07996	Thrombospondin-1 OS=Homo sapiens GN=THBS1 PE=1 SV=2 - [TSP1_HUMAN]	46.32	3	44	44	183	27.16	24.71	25.38	26.96	24.55	25.58	3	3	27.32	24.78	25.44	26.71	24.76	25.32	25.85	25.60	0.806	0.242	-0.251
O00159	Unconventional myosin-Ic OS=Homo sapiens GN=MYO1C PE=1 SV=4 - [MYO1C_HUMAN]	53.43	3	46	47	246	26.22	26.16	26.33	26.29	25.09	26.74	3	3	26.38	26.23	26.39	26.04	25.30	26.48	26.33	25.94	0.318	0.118	-0.394
Q96QK1	Vacuolar protein sorting-associated protein 35 OS=Homo sapiens GN=VPS35 PE=1 SV=2 - [VPS35_HUMAN]	29.65	1	16	16	52	23.72	24.16	24.02	24.57	23.37	24.65	3	3	23.88	24.23	24.08	24.32	23.59	24.39	24.06	24.10	0.903	0.261	0.036
Q9UK45	U6 snRNA-associated Sm-like protein LSm7 OS=Homo sapiens GN=LSM7 PE=1 SV=1 - [LSM7_HUMAN]	24.27	1	1	1	6	22.99	23.62	23.68	23.71	21.40		3	2	23.16	23.69	23.74	23.47	21.62		23.53	22.54	0.270	0.103	-0.987
Q92930	Ras-related protein Rab-8B OS=Homo sapiens GN=RAB8B PE=1 SV=2 - [RAB8B_HUMAN]	57.97	11	5	12	82	27.47	28.06	28.07	27.79	27.31	28.08	3	3	27.63	28.13	28.13	27.54	27.52	27.82	27.96	27.63	0.153	0.069	-0.334
Q95604	HLA class I histocompatibility antigen, Cw-17 alpha chain OS=Homo sapiens GN=HLA-C PE=1 SV=1 - [1C17_HUMAN]	28.49	2	0	7	46	28.04	28.00	28.07	28.80	27.22	28.77	3	3	28.20	28.07	28.13	28.55	27.43	28.50	28.14	28.16	0.944	0.270	0.027
P84095	Rho-related GTP-binding protein RhoG OS=Homo sapiens GN=RHOG PE=1 SV=1 - [RHOG_HUMAN]	68.06	3	9	10	63	26.33	26.13	26.18	26.60	25.27	26.31	3	3	26.49	26.20	26.24	26.35	25.49	26.05	26.31	25.96	0.266	0.103	-0.347
O15162	Phospholipid scramblase 1 OS=Homo sapiens GN=PLSCR1 PE=1 SV=1 - [PLS1_HUMAN]	15.72	1	4	4	11	23.25	23.51	23.86	23.46		23.22	3	2	23.41	23.58	23.92	23.22		22.96	23.64	23.09	0.085	0.045	-0.549
P46821	Microtubule-associated protein 1B OS=Homo sapiens GN=MAP1B PE=1 SV=2 - [MAP1B_HUMAN]	3.57	3	6	6	12	22.85	21.93	22.67	22.68	22.02	22.64	3	3	23.01	21.99	22.73	22.43	22.24	22.38	22.58	22.35	0.504	0.171	-0.226
Q04941	Proteolipid protein 2 OS=Homo sapiens GN=PLP2 PE=1 SV=1 - [PLP2_HUMAN]	18.42	1	2	2	11	25.39	28.24	27.31	26.96	25.33	27.04	3	3	25.55	28.31	27.37	26.71	25.55	26.78	27.08	26.35	0.463	0.159	-0.731
Q9Y263	Phospholipase A-2-activating protein OS=Homo sapiens GN=PLAA PE=1 SV=2 - [PLAP_HUMAN]	19.62	1	11	11	19	22.50	22.31	22.28	23.44	21.04	23.60	3	3	22.66	22.38	22.34	23.20	21.26	23.34	22.46	22.60	0.849	0.251	0.138
P84090	Enhancer of rudimentary homolog OS=Homo sapiens GN=ERH PE=1 SV=1 - [ERH_HUMAN]	44.23	1	4	4	11	23.02	23.58		23.39	23.44	23.39	2	3	23.18	23.65		23.14	23.66	23.13	23.42	23.31	0.739	0.228	-0.103
Q12792	Twinfilin-1 OS=Homo sapiens GN=TWF1 PE=1 SV=3 - [TWF1_HUMAN]	11.43	1	2	4	11	22.23	22.12	22.52	22.53	21.44	22.54	3	3	22.39	22.19	22.58	22.29	21.65	22.27	22.38	22.07	0.260	0.101	-0.313
P52907	F-actin-capping protein subunit alpha-1 OS=Homo sapiens GN=CAPZA1 PE=1 SV=3 - [CAZA1_HUMAN]	65.73	1	9	11	84	26.83	26.23	26.20	26.44	25.91	26.59	3	3	26.99	26.30	26.26	26.20	26.13	26.33	26.51	26.22	0.295	0.111	-0.294
O14556	Glyceraldehyde-3-phosphate dehydrogenase, testis-specific OS=Homo sapiens GN=GAPDHS PE=1 SV=2 - [G3PT_HUMAN]	4.41	1	1	2	10	28.28	27.83		26.21	28.59	29.23	2	3	28.44	27.90		25.96	28.81	28.97	28.17	27.91	0.854	0.252	-0.256
Q6ZVM7	TOM1-like protein 2 OS=Homo sapiens GN=TOM1L2 PE=1 SV=1 - [TM1L2_HUMAN]	7.69	1	3	3	5	21.54	20.66		21.41	21.04		2	2	21.71	20.73		21.16	21.25		21.22	21.21	0.981	0.278	-0.013
Q9UBN6	Tumor necrosis factor receptor superfamily member 10D OS=Homo sapiens GN=TNFRSF10D PE=1 SV=1 - [TR10D_HUMAN]	13.73	1	3	3	10	23.19	22.32		23.92	20.34	22.63	2	3	23.35	22.39		23.68	20.56	22.37	22.87	22.20	0.623	0.202	-0.666
P35222	Catenin beta-1 OS=Homo sapiens GN=CTNNB1 PE=1 SV=1 - [CTNB1_HUMAN]	18.18	1	8	10	36	22.92	23.30	22.89	23.28	21.61	23.38	3	3	23.09	23.37	22.95	23.03	21.83	23.12	23.14	22.66	0.335	0.123	-0.477
Q6FI81	Anamorsin OS=Homo sapiens GN=CIAPIN1 PE=1 SV=2 - [CPIN1_HUMAN]	13.14	1	2	2	12	23.11	23.37	23.20	23.82	23.02	23.20	3	3	23.27	23.44	23.26	23.58	23.24	22.94	23.32	23.25	0.741	0.229	-0.068
Q5T764	Interferon-induced protein with tetratricopeptide repeats 1B OS=Homo sapiens GN=IFIT1B PE=2 SV=1 - [IFT1B_HUMAN]	2.53	2	1	1	6	21.60	21.68	21.58	21.06	21.09	22.22	3	3	21.76	21.74	21.64	20.82	21.31	21.96	21.72	21.36	0.349	0.126	-0.354
Q8WTV0	Scavenger receptor class B member 1 OS=Homo sapiens GN=SCARB1 PE=1 SV=1 - [SCRB1_HUMAN]	5.43	1	3	3	11	22.33	23.43	23.36	23.63	20.88	23.25	3	3	22.49	23.50	23.42	23.38	21.10	22.99	23.13	22.49	0.454	0.156	-0.643
Q9H2H9	Sodium-coupled neutral amino acid transporter 1 OS=Homo sapiens GN=SLC38A1 PE=1 SV=1 - [S38A1_HUMAN]	8.62	1	3	3	12	23.88	24.71	24.88	24.36	24.14	25.40	3	3	24.04	24.78	24.94	24.12	24.35	25.14	24.59	24.54	0.908	0.262	-0.051
P10319	HLA class I histocompatibility antigen, B-58 alpha chain OS=Homo sapiens GN=HLA-B PE=1 SV=1 - [1B58_HUMAN]	32.60	2	0	8	49	26.46	26.64	26.56	27.68	25.34	27.58	3	3	26.62	26.71	26.62	27.44	25.56	27.32	26.65	26.77	0.849	0.251	0.123
Q9NZM1	Myoferlin OS=Homo sapiens GN=MYOF PE=1 SV=1 - [MYOF_HUMAN]	60.80	1	100	102	587	26.52	26.47	25.97	26.95	25.72	26.87	3	3	26.68	26.54	26.03	26.70	25.94	26.61	26.42	26.41	0.993	0.281	-0.003
Q16270	Insulin-like growth factor-binding protein 7 OS=Homo sapiens GN=IGFBP7 PE=1 SV=1 - [IBP7_HUMAN]	18.79	1	3	3	8		22.52	22.12		20.56	22.90	2	2		22.58	22.18		20.77	22.64	22.38	21.71	0.554	0.184	-0.674
Q08043	Alpha-actinin-3 OS=Homo sapiens GN=ACTN3 PE=1 SV=2 - [ACTN3_HUMAN]	10.32	1	1	9	45	24.87	25.76	25.66	25.74	24.75	26.49	3	3	25.03	25.83	25.72	25.50	24.97	26.23	25.52	25.56	0.933	0.267	0.039
Q01973	Inactive tyrosine-protein kinase transmembrane receptor ROR1 OS=Homo sapiens GN=ROR1 PE=1 SV=2 - [ROR1_HUMAN]	4.16	2	3	3	8	21.97	21.89	21.67	22.06	21.27	22.74	3	3	22.14	21.96	21.73	21.81	21.49	22.48	21.94	21.93	0.968	0.275	-0.014
Q9BSJ8	Extended synaptotagmin-1 OS=Homo sapiens GN=ESYT1 PE=1 SV=1 - [ESYT1_HUMAN]	11.96	1	8	8	22	22.30	22.54	22.90	22.19	22.28	23.11	3	3	22.46	22.61	22.96	21.94	22.49	22.85	22.68	22.43	0.464	0.159	-0.245
O00194	Ras-related protein Rab-27B OS=Homo sapiens GN=RAB27B PE=1 SV=4 - [RB27B_HUMAN]	5.05	1	1	1	5		22.61	22.53	22.97	22.33	23.21	2	3		22.68	22.59	22.72	22.55	22.95	22.64	22.74	0.547	0.182	0.103
CON_007038423	SWISS-PROT:Q29RQ1 Bos taurus Complement component C7 precursor	20.52	1	12	12	25	23.00	17.96	22.62	20.70	20.48	23.99	3	3	23.16	18.03	22.68	20.46	20.70	23.73	21.29	21.63	0.871	0.255	0.338
Q9NQW6	Actin-binding protein anillin OS=Homo sapiens GN=ANLN PE=1 SV=2 - [ANLN_HUMAN]	10.23	1	10	10	16	22.17	22.70	21.99	21.63		23.29	3	2	22.33	22.76	22.05	21.38		23.03	22.38	22.21	0.811	0.243	-0.175
O75663	TIP41-like protein OS=Homo sapiens GN=TIPRL PE=1 SV=2 - [TIPRL_HUMAN]	37.87	1	6	6	18	22.75	21.62	22.56	21.94	21.80	23.86	3	3	22.91	21.69	22.62	21.69	22.01	23.59	22.41	22.43	0.971	0.276	0.027
P63096	Guanine nucleotide-binding protein G(i) subunit alpha-1 OS=Homo sapiens GN=GNAI1 PE=1 SV=2 - [GNAI1_HUMAN]	53.95	4	6	15	121	27.14	27.41	27.33	27.91	26.59	27.93	3	3	27.30	27.48	27.39	27.66	26.81	27.67	27.39	27.38	0.970	0.276	-0.012
CON_Q3ZBD7	Glucose-6-phosphate isomerase OS=Bos taurus GN=GPI PE=2 SV=4 - [G6PI_BOVIN]	10.77	1	1	5	30	24.88	25.01	24.87	25.36	24.23	25.70	3	3	25.04	25.08	24.93	25.11	24.45	25.44	25.02	25.00	0.949	0.271	-0.020
Q03135	Caveolin-1 OS=Homo sapiens GN=CAV1 PE=1 SV=4 - [CAV1_HUMAN]	34.83	2	5	5	25	25.91	25.58	25.73	26.11	24.97	25.86	3	3	26.07	25.65	25.79	25.87	25.19	25.60	25.83	25.55	0.289	0.109	-0.285
O95445	Apolipoprotein M OS=Homo sapiens GN=APOM PE=1 SV=2 - [APOM_HUMAN]	6.91	1	2	2	7	23.27	23.02		23.84	22.77	24.12	2	3	23.43	23.09		23.59	22.99	23.86	23.26	23.48	0.579	0.190	0.220
P55060	Exportin-2 OS=Homo sapiens GN=CSE1L PE=1 SV=3 - [XPO2_HUMAN]	31.62	1	24	24	95	29.85	30.43	29.83	31.98	24.53	30.97	3	3	30.02	30.50	29.89	31.73	24.75	30.71	30.14	29.07	0.650	0.208	-1.070
O00220	Tumor necrosis factor receptor superfamily member 10A OS=Homo sapiens GN=TNFRSF10A PE=1 SV=3 - [TR10A_HUMAN]	8.76	1	3	3	10	22.13	22.20		24.18	21.31	22.86	2	3	22.29	22.27		23.93	21.52	22.60	22.28	22.69	0.685	0.216	0.403
P31040	Succinate dehydrogenase [ubiquinone] flavoprotein subunit, mitochondrial OS=Homo sapiens GN=SDHA PE=1 SV=2 - [SDHA_HUMAN]	26.20	1	10	10	25		27.21	25.73	26.16	25.47	26.50	2	3		27.28	25.79	25.92	25.69	26.24	26.53	25.95	0.397	0.139	-0.583
P63092	Guanine nucleotide-binding protein G(s) subunit alpha isoforms short OS=Homo sapiens GN=GNAS PE=1 SV=1 - [GNAS2_HUMAN]	53.30	3	17	18	127	27.33	27.36	27.32	27.69	26.36	27.52	3	3	27.49	27.43	27.37	27.44	26.58	27.26	27.43	27.09	0.270	0.103	-0.340
Q5VUB5	Protein FAM171A1 OS=Homo sapiens GN=FAM171A1 PE=1 SV=1 - [F1711_HUMAN]	6.85	1	6	6	27		20.46	17.98	22.59	19.03	21.15	2	3		20.53	18.04	22.34	19.25	20.89	19.29	20.83	0.374	0.133	1.543
Q7Z2W4	Zinc finger CCCH-type antiviral protein 1 OS=Homo sapiens GN=ZC3HAV1 PE=1 SV=3 - [ZCCHV_HUMAN]	12.20	1	8	8	30	22.35	22.85	22.48	22.81	22.22	23.03	3	3	22.51	22.92	22.54	22.57	22.43	22.77	22.66	22.59	0.699	0.219	-0.067
Q15942	Zyxin OS=Homo sapiens GN=ZYX PE=1 SV=1 - [ZYX_HUMAN]	16.26	1	5	5	18	22.97	23.38	23.14	23.59	22.73	23.56	3	3	23.13	23.45	23.20	23.34	22.95	23.30	23.26	23.20	0.709	0.221	-0.063
CON_006918191	SWISS-PROT:P02672 Bos taurus Fibrinogen alpha chain precursor	10.89	1	7	7	20	23.33	22.83	23.44	23.61	22.23	24.12	3	3	23.49	22.90	23.50	23.37	22.45	23.86	23.30	23.23	0.886	0.257	-0.070
P34931	Heat shock 70 kDa protein 1-like OS=Homo sapiens GN=HSPA1L PE=1 SV=2 - [HS71L_HUMAN]	30.11	1	1	15	133	30.26	30.91	30.75	30.94	30.48	30.81	3	3	30.42	30.98	30.81	30.69	30.70	30.55	30.74	30.65	0.625	0.202	-0.091
Q562R1	Beta-actin-like protein 2 OS=Homo sapiens GN=ACTBL2 PE=1 SV=2 - [ACTBL_HUMAN]	19.68	1	1	9	133	30.45	31.37	30.59	30.72	30.27	32.14	3	3	30.61	31.44	30.65	30.47	30.49	31.88	30.90	30.95	0.935	0.268	0.046
P29966	Myristoylated alanine-rich C-kinase substrate OS=Homo sapiens GN=MARCKS PE=1 SV=4 - [MARCS_HUMAN]	51.81	1	13	13	102	27.79	28.69	28.60	28.64	27.98	28.88	3	3	27.95	28.76	28.66	28.40	28.19	28.62	28.45	28.40	0.869	0.254	-0.050
Q14161	ARF GTPase-activating protein GIT2 OS=Homo sapiens GN=GIT2 PE=1 SV=2 - [GIT2_HUMAN]	3.69	1	1	1	7	21.75	22.18	21.83	22.73	21.70		3	2	21.91	22.25	21.89	22.49	21.92		22.02	22.20	0.519	0.174	0.189
P10620	Microsomal glutathione S-transferase 1 OS=Homo sapiens GN=MGST1 PE=1 SV=1 - [MGST1_HUMAN]	16.13	1	2	2	7	23.00	25.00	25.21	24.14	24.57	24.96	3	3	23.16	25.07	25.27	23.89	24.79	24.70	24.50	24.46	0.960	0.273	-0.039
P62745	Rho-related GTP-binding protein RhoB OS=Homo sapiens GN=RHOB PE=1 SV=1 - [RHOB_HUMAN]	39.29	1	3	5	24	26.24	26.28	26.30	25.67	26.37	26.55	3	3	26.40	26.35	26.36	25.42	26.58	26.29	26.37	26.10	0.479	0.164	-0.272
P84077	ADP-ribosylation factor 1 OS=Homo sapiens GN=ARF1 PE=1 SV=2 - [ARF1_HUMAN]	86.74	5	9	16	132	27.59	27.49	27.76	27.76	26.95	27.91	3	3	27.75	27.56	27.82	27.52	27.17	27.65	27.71	27.44	0.181	0.078	-0.266
Q8WWI5	Choline transporter-like protein 1 OS=Homo sapiens GN=SLC44A1 PE=1 SV=1 - [CTL1_HUMAN]	6.09	1	3	3	16	23.71	23.73	23.91	24.05		23.20	3	2	23.87	23.80	23.97	23.81		22.94	23.88	23.37	0.223	0.090	-0.507
O76003	Glutaredoxin-3 OS=Homo sapiens GN=GLRX3 PE=1 SV=2 - [GLRX3_HUMAN]	30.15	1	7	7	25	25.00	22.94	25.50	24.38	24.04	24.41	3	3	25.16	23.01	25.56	24.14	24.26	24.15	24.58	24.18	0.642	0.206	-0.398
Q15056	Eukaryotic translation initiation factor 4H OS=Homo sapiens GN=EIF4H PE=1 SV=5 - [IF4H_HUMAN]	25.81	1	4	4	15	23.58	24.16	24.24	24.20	23.44	23.98	3	3	23.74	24.23	24.30	23.96	23.65	23.72	24.09	23.78	0.187	0.079	-0.312
Q99439	Calponin-2 OS=Homo sapiens GN=CNN2 PE=1 SV=4 - [CNN2_HUMAN]	17.80	1	3	4	13	26.55	23.67	23.49	24.68	23.57	24.71	3	3	26.71	23.74	23.55	24.43	23.78	24.45	24.67	24.22	0.693	0.217	-0.445
P58335	Anthrax toxin receptor 2 OS=Homo sapiens GN=ANTXR2 PE=1 SV=5 - [ANTR2_HUMAN]	19.02	1	8	9	25	22.88	23.76	24.17	24.71	22.17	24.77	3	3	23.04	23.83	24.23	24.46	22.39	24.51	23.70	23.78	0.920	0.265	0.084
P13645	Keratin, type I cytoskeletal 10 OS=Homo sapiens GN=KRT10 PE=1 SV=6 - [K1C10_HUMAN]	55.82	14	23	27	168	27.00	25.61	24.77	26.88	25.49	24.94	3	3	27.17	25.68	24.83	26.63	25.70	24.67	25.89	25.67	0.816	0.244	-0.221
O14641	Segment polarity protein dishevelled homolog DVL-2 OS=Homo sapiens GN=DVL2 PE=1 SV=1 - [DVL2_HUMAN]	8.83	2	6	6	17	21.75	20.52	20.52	20.96	22.39	20.12	3	3	21.92	20.59	20.58	20.72	22.60	19.86	21.03	21.06	0.975	0.277	0.031
P04899	Guanine nucleotide-binding protein G(i) subunit alpha-2 OS=Homo sapiens GN=GNAI2 PE=1 SV=3 - [GNAI2_HUMAN]	69.30	4	16	25	235	27.46	27.78	27.75	28.20	26.93	28.26	3	3	27.62	27.85	27.81	27.95	27.15	28.00	27.76	27.70	0.844	0.250	-0.060
Q01970	1-phosphatidylinositol 4,5-bisphosphate phosphodiesterase beta-3 OS=Homo sapiens GN=PLCB3 PE=1 SV=2 - [PLCB3_HUMAN]	6.97	1	5	5	8	20.83	21.73	21.86	21.56		21.47	3	2	20.99	21.80	21.92	21.32		21.21	21.57	21.26	0.475	0.163	-0.308
P08754	Guanine nucleotide-binding protein G(k) subunit alpha OS=Homo sapiens GN=GNAI3 PE=1 SV=3 - [GNAI3_HUMAN]	59.60	4	10	17	176	27.26	27.56	27.52	27.97	26.71	28.07	3	3	27.43	27.63	27.58	27.73	26.92	27.81	27.55	27.49	0.846	0.251	-0.060
Q9H0U4	Ras-related protein Rab-1B OS=Homo sapiens GN=RAB1B PE=1 SV=1 - [RAB1B_HUMAN]	79.10	9	6	15	102	27.80	28.24	28.20	28.10	27.57	28.35	3	3	27.97	28.31	28.26	27.85	27.78	28.09	28.18	27.91	0.129	0.060	-0.273
P62820	Ras-related protein Rab-1A OS=Homo sapiens GN=RAB1A PE=1 SV=3 - [RAB1A_HUMAN]	75.61	8	7	16	100	27.80	28.24	28.20	28.10	27.57	28.35	3	3	27.97	28.31	28.26	27.85	27.78	28.09	28.18	27.91	0.129	0.060	-0.273
Q99715	Collagen alpha-1(XII) chain OS=Homo sapiens GN=COL12A1 PE=1 SV=2 - [COCA1_HUMAN]	20.93	1	49	49	106	24.58	20.45	23.65	25.87	20.84	21.07	3	3	24.74	20.52	23.71	25.62	21.06	20.81	22.99	22.50	0.819	0.245	-0.493
P07355	Annexin A2 OS=Homo sapiens GN=ANXA2 PE=1 SV=2 - [ANXA2_HUMAN]	84.07	2	36	36	333	30.09	30.82	30.69	30.54	30.14	30.68	3	3	30.25	30.89	30.75	30.29	30.35	30.41	30.63	30.35	0.235	0.093	-0.277
Q14118	Dystroglycan OS=Homo sapiens GN=DAG1 PE=1 SV=2 - [DAG1_HUMAN]	8.16	1	4	4	18	23.83	24.66	24.69	24.82	23.63	24.27	3	3	24.00	24.73	24.75	24.58	23.85	24.01	24.49	24.15	0.357	0.129	-0.346
P53597	Succinyl-CoA ligase [ADP/GDP-forming] subunit alpha, mitochondrial OS=Homo sapiens GN=SUCLG1 PE=1 SV=4 - [SUCA_HUMAN]	10.69	1	3	3	10	22.19	23.65	23.08		23.16	23.97	3	2	22.35	23.72	23.14		23.37	23.71	23.07	23.54	0.439	0.152	0.472
O00299	Chloride intracellular channel protein 1 OS=Homo sapiens GN=CLIC1 PE=1 SV=4 - [CLIC1_HUMAN]	87.14	1	16	17	119	28.15	27.92	27.76	28.26	27.14	28.16	3	3	28.32	27.99	27.82	28.01	27.36	27.90	28.04	27.76	0.321	0.119	-0.283
CON_006912121	SWISS-PROT:Q3SZR3 Bos taurus Alpha-1-acid glycoprotein precursor	34.65	1	7	7	32	23.40	24.72	24.61	25.17	24.28	23.87	3	3	23.57	24.79	24.67	24.93	24.49	23.61	24.34	24.34	0.995	0.281	0.004
O43707	Alpha-actinin-4 OS=Homo sapiens GN=ACTN4 PE=1 SV=2 - [ACTN4_HUMAN]	73.00	1	36	55	414	27.26	27.54	27.31	27.77	26.72	27.98	3	3	27.43	27.61	27.37	27.52	26.94	27.72	27.47	27.39	0.768	0.234	-0.077
Q9HCM2	Plexin-A4 OS=Homo sapiens GN=PLXNA4 PE=1 SV=4 - [PLXA4_HUMAN]	3.59	1	1	7	13	22.44	23.56	23.03	23.16	22.54	22.96	3	3	22.60	23.63	23.09	22.91	22.75	22.70	23.10	22.79	0.356	0.129	-0.317
Q7L576	Cytoplasmic FMR1-interacting protein 1 OS=Homo sapiens GN=CYFIP1 PE=1 SV=1 - [CYFP1_HUMAN]	12.93	2	15	15	45	23.45	22.89	23.02	23.57	21.61	23.75	3	3	23.62	22.96	23.07	23.33	21.83	23.48	23.22	22.88	0.581	0.190	-0.339
P38606	V-type proton ATPase catalytic subunit A OS=Homo sapiens GN=ATP6V1A PE=1 SV=2 - [VATA_HUMAN]	33.87	1	14	14	41	23.08	24.38	22.37	23.40	23.11	23.90	3	3	23.24	24.45	22.43	23.15	23.33	23.64	23.38	23.37	0.998	0.281	-0.002

P05556	Integrin beta-1 OS=Homo sapiens GN=ITGB1 PE=1 SV=2 - [ITB1_HUMAN]	49.00	1	37	37	446	30.38	30.27	30.29	30.69	29.41	30.56	3	3	30.54	30.34	30.35	30.45	29.63	30.30	30.41	30.12	0.336	0.123	-0.283
Q15417	Calponin-3 OS=Homo sapiens GN=CNN3 PE=1 SV=1 - [CNN3_HUMAN]	23.10	1	5	6	23	26.05	23.80	23.99	24.66	23.84	24.90	3	3	26.21	23.87	24.05	24.42	24.06	24.64	24.71	24.37	0.680	0.215	-0.342
P42685	Tyrosine-protein kinase FRK OS=Homo sapiens GN=FRK PE=1 SV=1 - [FRK_HUMAN]	3.17	4	1	2	6	23.00	21.92	22.07	22.75		22.22	3	2	23.17	21.99	22.13	22.51		21.96	22.43	22.23	0.736	0.227	-0.193
P06703	Protein S100-A6 OS=Homo sapiens GN=S100A6 PE=1 SV=1 - [S10A6_HUMAN]	45.56	1	5	5	32	28.22	28.58	28.65	28.40	27.94	29.38	3	3	28.39	28.65	28.71	28.15	28.16	29.12	28.58	28.48	0.768	0.234	-0.106
Q9UHD9	Ubiquilin-2 OS=Homo sapiens GN=UBQLN2 PE=1 SV=2 - [UBQL2_HUMAN]	25.96	1	6	11	41	23.46	24.80	24.59	24.64	24.32	24.25	3	3	23.62	24.87	24.65	24.39	24.53	23.99	24.38	24.30	0.863	0.253	-0.077
Q96N66	Lysophospholipid acyltransferase 7 OS=Homo sapiens GN=MBOAT7 PE=1 SV=2 - [MBOA7_HUMAN]	11.23	1	4	4	14		23.53	23.15	22.64	22.84	24.29	2	3		23.60	23.21	22.40	23.05	24.02	23.40	23.16	0.724	0.225	-0.243
Q7L9L4	MOB kinase activator 1B OS=Homo sapiens GN=MOB1B PE=1 SV=3 - [MOB1B_HUMAN]	29.63	2	5	5	14	23.46	23.75	23.31	23.92		22.55	3	2	23.62	23.82	23.37	23.67		22.29	23.60	22.98	0.335	0.123	-0.620
Q13131	5'-AMP-activated protein kinase catalytic subunit alpha-1 OS=Homo sapiens GN=PRKAA1 PE=1 SV=4 - [AAPK1_HUMAN]	29.16	2	12	12	35	23.39	22.53	23.07	22.82	22.83	23.55	3	3	23.55	22.59	23.13	22.57	23.05	23.29	23.09	22.97	0.744	0.229	-0.122
P42330	Aldo-keto reductase family 1 member C3 OS=Homo sapiens GN=AKR1C3 PE=1 SV=4 - [AK1C3_HUMAN]	12.38	2	2	2	7	23.23	22.80		23.45	22.44	24.14	2	3	23.39	22.87		23.21	22.65	23.88	23.13	23.25	0.831	0.247	0.116
Q9ULV4	Coronin-1C OS=Homo sapiens GN=CORO1C PE=1 SV=1 - [COR1C_HUMAN]	42.41	1	15	15	69	25.78	25.86	25.71	25.77	25.27	26.13	3	3	25.94	25.93	25.77	25.52	25.49	25.87	25.88	25.62	0.126	0.059	-0.255
Q9UHB6	LIM domain and actin-binding protein 1 OS=Homo sapiens GN=LIMA1 PE=1 SV=1 - [LIMA1_HUMAN]	5.27	1	3	3	16	22.17	21.83	21.77	19.80	22.11	23.15	3	3	22.33	21.90	21.83	19.55	22.33	22.89	22.02	21.59	0.702	0.219	-0.429
Q14344	Guanine nucleotide-binding protein subunit alpha-13 OS=Homo sapiens GN=GNA13 PE=1 SV=2 - [GNA13_HUMAN]	38.99	3	12	14	61	26.63	26.78	26.74	27.37	25.99	27.08	3	3	26.79	26.85	26.80	27.12	26.21	26.82	26.81	26.72	0.739	0.228	-0.097
O43795	Unconventional myosin-Ib OS=Homo sapiens GN=MYO1B PE=1 SV=3 - [MYO1B_HUMAN]	22.27	2	18	19	76	24.07	23.88	23.89	24.51	23.06	24.56	3	3	24.23	23.95	23.95	24.26	23.28	24.30	24.04	23.95	0.797	0.240	-0.096
P17655	Calpain-2 catalytic subunit OS=Homo sapiens GN=CAPN2 PE=1 SV=6 - [CAN2_HUMAN]	53.43	2	27	27	138	25.53	25.38	25.34	25.08	25.21	26.20	3	3	25.69	25.45	25.40	24.84	25.43	25.94	25.51	25.40	0.757	0.231	-0.110
Q9UBV8	Peflin OS=Homo sapiens GN=PEF1 PE=1 SV=1 - [PEF1_HUMAN]	33.80	1	7	7	24	24.89	23.79	23.67	24.35	23.66	24.62	3	3	25.05	23.85	23.73	24.10	23.87	24.36	24.21	24.11	0.831	0.247	-0.101
Q13433	Zinc transporter ZIP6 OS=Homo sapiens GN=SLC39A6 PE=1 SV=3 - [S39A6_HUMAN]	10.46	1	8	8	37	22.78	23.47	23.58	23.92	22.71	23.54	3	3	22.94	23.54	23.64	23.68	22.93	23.28	23.37	23.30	0.811	0.243	-0.079
P02786	Transferrin receptor protein 1 OS=Homo sapiens GN=TFRC PE=1 SV=2 - [TFR1_HUMAN]	49.74	2	33	33	219	27.47	27.30	26.99	27.99	26.52	27.52	3	3	27.64	27.37	27.05	27.75	26.74	27.26	27.35	27.25	0.771	0.234	-0.105
Q09666	Neuroblast differentiation-associated protein AHNAK OS=Homo sapiens GN=AHNAK PE=1 SV=2 - [AHNK_HUMAN]	41.49	2	105	105	364	24.76	26.65	26.25	25.95	25.29	25.97	3	3	24.92	26.72	26.30	25.70	25.51	25.71	25.98	25.64	0.567	0.187	-0.341
P11169	Solute carrier family 2, facilitated glucose transporter member 3 OS=Homo sapiens GN=SLC2A3 PE=1 SV=1 - [GTR3_HUMAN]	7.86	2	3	4	9	24.68	26.28	25.52	25.98	24.83	26.16	3	3	24.84	26.35	25.58	25.73	25.05	25.89	25.59	25.56	0.950	0.271	-0.034
P11142	Heat shock cognate 71 kDa protein OS=Homo sapiens GN=HSPA8 PE=1 SV=1 - [HSP7C_HUMAN]	60.06	2	29	34	465	30.66	31.16	30.94	31.00	30.63	30.97	3	3	30.82	31.23	31.00	30.76	30.85	30.71	31.02	30.77	0.119	0.056	-0.243
P62879	Guanine nucleotide-binding protein G(I)/G(S)/G(T) subunit beta-2 OS=Homo sapiens GN=GNB2 PE=1 SV=3 - [GBB2_HUMAN]	70.00	1	6	15	134	28.03	28.43	28.25	28.48	27.69	28.75	3	3	28.19	28.50	28.30	28.24	27.91	28.49	28.33	28.21	0.573	0.189	-0.117
Q4LE39	AT-rich interactive domain-containing protein 4B OS=Homo sapiens GN=ARID4B PE=1 SV=2 - [ARI4B_HUMAN]	0.99	1	1	1	6	19.32		21.10	18.68	19.10	22.08	2	3	19.49		21.16	18.44	19.31	21.82	20.32	19.86	0.769	0.234	-0.465
P61981	14-3-3 protein gamma OS=Homo sapiens GN=YWHAG PE=1 SV=2 - [1433G_HUMAN]	57.89	1	9	13	119	26.61	27.88	27.80	28.67	26.71	27.45	3	3	26.77	27.95	27.86	28.43	26.93	27.19	27.53	27.52	0.985	0.279	-0.012
O15498	Synaptobrevin homolog YKT6 OS=Homo sapiens GN=YKT6 PE=1 SV=1 - [YKT6_HUMAN]	29.80	1	6	6	16	23.24	23.29	23.11	24.03	22.40	23.49	3	3	23.40	23.36	23.17	23.79	22.62	23.23	23.31	23.21	0.787	0.238	-0.100
Q8IZP0	Abl interactor 1 OS=Homo sapiens GN=ABI1 PE=1 SV=4 - [ABI1_HUMAN]	14.57	2	6	6	21	23.20	23.29	22.78	23.22	22.73	23.17	3	3	23.36	23.36	22.84	22.98	22.95	22.90	23.19	22.94	0.234	0.093	-0.244
Q9UHN6	Transmembrane protein 2 OS=Homo sapiens GN=TMEM2 PE=1 SV=1 - [TMEM2_HUMAN]	8.10	1	6	6	16	23.41	23.71	23.62	23.53	23.33	23.78	3	3	23.57	23.78	23.68	23.28	23.55	23.51	23.67	23.45	0.093	0.048	-0.225
Q5TZA2	Rootletin OS=Homo sapiens GN=CROCC PE=1 SV=1 - [CROCC_HUMAN]	0.74	1	1	2	6		23.40	23.20	23.94	22.66	23.70	2	3		23.47	23.26	23.70	22.87	23.43	23.36	23.33	0.932	0.267	-0.030
P00338	L-lactate dehydrogenase A chain OS=Homo sapiens GN=LDHA PE=1 SV=2 - [LDHA_HUMAN]	82.53	4	22	24	209	28.63	28.34	27.99	28.56	27.51	29.19	3	3	28.79	28.41	28.04	28.32	27.73	28.93	28.41	28.32	0.836	0.248	-0.090
P27348	14-3-3 protein theta OS=Homo sapiens GN=YWHAQ PE=1 SV=1 - [1433T_HUMAN]	48.57	1	8	11	105	26.96	27.98	27.95	28.78	26.80	27.77	3	3	27.13	28.05	28.01	28.54	27.02	27.51	27.73	27.69	0.945	0.270	-0.040
Q9ULF5	Zinc transporter ZIP10 OS=Homo sapiens GN=SLC39A10 PE=1 SV=2 - [S39AA_HUMAN]	14.68	1	9	9	40	24.46	24.33	24.15	24.57	23.86	24.40	3	3	24.62	24.40	24.21	24.33	24.07	24.14	24.41	24.18	0.177	0.076	-0.232
Q01650	Large neutral amino acids transporter small subunit 1 OS=Homo sapiens GN=SLC7A5 PE=1 SV=2 - [LAT1_HUMAN]	7.10	3	4	4	13	25.41	25.67	25.42	26.77	24.24	25.90	3	3	25.57	25.74	25.48	26.52	24.45	25.64	25.60	25.54	0.930	0.267	-0.056
O00629	Importin subunit alpha-3 OS=Homo sapiens GN=KPNA4 PE=1 SV=1 - [IMA3_HUMAN]	14.78	1	4	4	10	23.98	23.44	23.45	23.22		23.98	3	2	24.14	23.51	23.51	22.98		23.71	23.72	23.35	0.404	0.141	-0.375
P10586	Receptor-type tyrosine-protein phosphatase F OS=Homo sapiens GN=PTPRF PE=1 SV=2 - [PTPRF_HUMAN]	5.77	3	7	7	12	22.51	22.83	22.95	23.68		22.14	3	2	22.67	22.90	23.01	23.44		21.88	22.86	22.66	0.758	0.232	-0.200
P57088	Transmembrane protein 33 OS=Homo sapiens GN=TMEM33 PE=1 SV=2 - [TMM33_HUMAN]	4.05	1	1	1	6	21.35	22.38	25.20	22.35		23.77	3	2	21.52	22.45	25.26	22.10		23.51	23.08	22.81	0.871	0.255	-0.272
P48960	CD97 antigen OS=Homo sapiens GN=CD97 PE=1 SV=4 - [CD97_HUMAN]	31.62	2	18	18	149	27.77	27.44	27.31	27.66	27.12	27.65	3	3	27.93	27.51	27.37	27.41	27.34	27.39	27.60	27.38	0.258	0.100	-0.225
P43121	Cell surface glycoprotein MUC18 OS=Homo sapiens GN=MCAM PE=1 SV=2 - [MUC18_HUMAN]	43.50	1	23	23	108	25.44	25.74	25.78	25.93	24.90	25.95	3	3	25.60	25.81	25.84	25.69	25.11	25.68	25.75	25.49	0.279	0.106	-0.257
CON_LysC	SWISS-PROT:P15636 Protease I precursor Lysyl endopeptidase Achromobacter lyticus	30.63	1	18	18	542	33.46	33.28	33.56	33.60	33.05	33.72	3	3	33.63	33.35	33.62	33.35	33.26	33.46	33.53	33.36	0.183	0.078	-0.174
CON_007137481	SWISS-PROT:Q9TT36 Bos taurus Thyroxine-binding globulin precursor	26.03	2	7	7	21	24.27		22.75	22.82	22.14	23.78	2	3	24.44		22.81	22.58	22.35	23.52	23.62	22.82	0.369	0.132	-0.804
P61077	Ubiquitin-conjugating enzyme E2 D3 OS=Homo sapiens GN=UBE2D3 PE=1 SV=1 - [UB2D3_HUMAN]	36.05	4	3	3	17	24.20	26.07	26.13	25.55	25.10	26.03	3	3	24.36	26.14	26.19	25.31	25.32	25.77	25.56	25.47	0.886	0.257	-0.095
O43491	Band 4.1-like protein 2 OS=Homo sapiens GN=EPB41L2 PE=1 SV=1 - [E41L2_HUMAN]	38.61	1	23	27	118	24.50	24.75	24.75	24.83	24.12	24.92	3	3	24.66	24.82	24.81	24.59	24.33	24.66	24.76	24.53	0.100	0.050	-0.236
P19827	Inter-alpha-trypsin inhibitor heavy chain H1 OS=Homo sapiens GN=ITIH1 PE=1 SV=3 - [ITIH1_HUMAN]	3.84	1	3	3	10	21.74		21.50	21.18	20.77	22.26	2	3	21.90		21.56	20.93	20.98	22.00	21.73	21.31	0.435	0.151	-0.420
Q15907	Ras-related protein Rab-11B OS=Homo sapiens GN=RAB11B PE=1 SV=4 - [RB11B_HUMAN]	60.55	3	12	12	102	26.96	27.19	26.83	27.31	26.49	27.34	3	3	27.12	27.26	26.89	27.07	26.70	27.08	27.09	26.95	0.446	0.154	-0.140
Q13464	Rho-associated protein kinase 1 OS=Homo sapiens GN=ROCK1 PE=1 SV=1 - [ROCK1_HUMAN]	4.28	1	3	4	8	21.49	20.05	20.25	20.75		20.79	3	2	21.65	20.12	20.31	20.51		20.53	20.69	20.52	0.795	0.239	-0.177
P62873	Guanine nucleotide-binding protein G(I)/G(S)/G(T) subunit beta-1 OS=Homo sapiens GN=GNB1 PE=1 SV=3 - [GBB1_HUMAN]	70.00	1	8	14	129	28.21	28.34	28.12	28.39	27.70	28.74	3	3	28.37	28.41	28.18	28.14	27.92	28.48	28.32	28.18	0.462	0.159	-0.144
P35908	Keratin, type II cytoskeletal 2 epidermal OS=Homo sapiens GN=KRT2 PE=1 SV=2 - [K22E_HUMAN]	72.14	3	25	35	132	26.99	25.63	25.61	27.11	26.17	25.07	3	3	27.15	25.70	25.67	26.87	26.38	24.81	26.17	26.02	0.857	0.252	-0.152
P61619	Protein transport protein Sec61 subunit alpha isoform 1 OS=Homo sapiens GN=SEC61A1 PE=1 SV=2 - [S61A1_HUMAN]	8.40	2	5	5	8		22.98	22.37		21.58	23.41	2	2		23.05	22.43		21.80	23.15	22.74	22.47	0.751	0.230	-0.270
Q9NUP9	Protein lin-7 homolog C OS=Homo sapiens GN=LIN7C PE=1 SV=1 - [LIN7C_HUMAN]	41.62	3	8	8	21	24.64	25.23	24.87	26.48	23.87	24.77	3	3	24.80	25.30	24.93	26.23	24.09	24.51	25.01	24.94	0.924	0.265	-0.068
Q8IYS1	Peptidase M20 domain-containing protein 2 OS=Homo sapiens GN=PM20D2 PE=1 SV=2 - [P20D2_HUMAN]	9.17	1	2	2	5	20.92	21.21		21.76	20.09	21.29	2	3	21.08	21.28		21.51	20.30	21.03	21.18	20.95	0.650	0.208	-0.232
P13928	Annexin A8 OS=Homo sapiens GN=ANXA8 PE=1 SV=3 - [ANXA8_HUMAN]	4.59	2	1	2	7	22.97		23.88	23.52		23.16	2	2	23.13		23.94	23.27		22.90	23.54	23.08	0.415	0.145	-0.455
Q12983	BCL2/adenovirus E1B 19 kDa protein-interacting protein 3 OS=Homo sapiens GN=BNIP3 PE=1 SV=3 - [BNIP3_HUMAN]	11.97	1	3	3	8	22.16	22.09	21.41	22.81	21.22		3	2	22.32	22.16	21.47	22.57	21.44		21.98	22.00	0.971	0.276	0.021
P60520	Gamma-aminobutyric acid receptor-associated protein-like 2 OS=Homo sapiens GN=GABARAPL2 PE=1 SV=1 - [GBRL2_HUMAN]	52.99	1	4	5	19	23.20	25.21	24.69	24.58	23.94	24.28	3	3	23.36	25.28	24.75	24.34	24.16	24.02	24.46	24.17	0.643	0.206	-0.290
P20020	Plasma membrane calcium-transporting ATPase 1 OS=Homo sapiens GN=ATP2B1 PE=1 SV=3 - [AT2B1_HUMAN]	39.35	3	27	38	217	25.57	25.49	25.43	26.00	24.58	26.06	3	3	25.73	25.56	25.49	25.76	24.80	25.80	25.59	25.45	0.704	0.220	-0.136
O15031	Plexin-B2 OS=Homo sapiens GN=PLXNB2 PE=1 SV=3 - [PLXB2_HUMAN]	27.80	1	33	33	117	24.56	24.14	24.25	24.25	23.93	24.69	3	3	24.72	24.21	24.31	24.00	24.14	24.43	24.41	24.19	0.333	0.123	-0.221
P04264	Keratin, type II cytoskeletal 1 OS=Homo sapiens GN=KRT1 PE=1 SV=6 - [K2C1_HUMAN]	63.04	2	33	40	180	27.27	25.99	25.90	27.45	26.60	25.24	3	3	27.43	26.06	25.96	27.20	26.82	24.98	26.48	26.33	0.866	0.254	-0.150
O75396	Vesicle-trafficking protein SEC22b OS=Homo sapiens GN=SEC22B PE=1 SV=4 - [SC22B_HUMAN]	25.12	1	4	4	11	22.48	21.98	21.45		20.86	22.34	3	2	22.64	22.05	21.51		21.07	22.08	22.07	21.58	0.449	0.155	-0.490
Q96NY7	Chloride intracellular channel protein 6 OS=Homo sapiens GN=CLIC6 PE=2 SV=3 - [CLIC6_HUMAN]	6.11	1	2	3	9	23.86	23.24	23.26	23.23		24.04	3	2	24.02	23.31	23.32	22.99		23.78	23.55	23.38	0.718	0.223	-0.168
P47756	F-actin-capping protein subunit beta OS=Homo sapiens GN=CAPZB PE=1 SV=4 - [CAPZB_HUMAN]	61.37	1	14	14	75	24.76	25.02	25.05	24.95	24.51	25.44	3	3	24.92	25.09	25.11	24.70	24.73	25.18	25.04	24.87	0.369	0.132	-0.168
Q13308	Inactive tyrosine-protein kinase 7 OS=Homo sapiens GN=PTK7 PE=1 SV=2 - [PTK7_HUMAN]	27.10	1	22	22	85	24.85	24.64	24.52	25.13	23.95	25.02	3	3	25.01	24.71	24.58	24.88	24.16	24.76	24.76	24.60	0.562	0.186	-0.162
O43657	Tetraspanin-6 OS=Homo sapiens GN=TSPAN6 PE=1 SV=1 - [TSN6_HUMAN]	18.78	1	5	5	24	25.88	25.82	22.81	24.46	23.69	25.94	3	3	26.04	25.89	22.87	24.22	23.91	25.68	24.94	24.60	0.790	0.238	-0.334
P61970	Nuclear transport factor 2 OS=Homo sapiens GN=NUTF2 PE=1 SV=1 - [NTF2_HUMAN]	56.69	1	5	5	38	26.12	25.57	25.29	25.75	25.17	25.97	3	3	26.28	25.64	25.35	25.51	25.38	25.71	25.76	25.53	0.488	0.166	-0.222
P54727	UV excision repair protein RAD23 homolog B OS=Homo sapiens GN=RAD23B PE=1 SV=1 - [RD23B_HUMAN]	21.52	1	8	9	50	23.71	24.73	24.64	24.43	23.90	24.64	3	3	23.87	24.80	24.70	24.19	24.12	24.38	24.46	24.23	0.494	0.168	-0.229
Q05209	Tyrosine-protein phosphatase non-receptor type 12 OS=Homo sapiens GN=PTPN12 PE=1 SV=3 - [PTN12_HUMAN]	3.08	1	2	2	7	18.63	19.08		17.09	20.38	21.14	2	3	18.79	19.15		16.85	20.59	20.88	18.97	19.44	0.799	0.240	0.467
P02768	Serum albumin OS=Homo sapiens GN=ALB PE=1 SV=2 - [ALBU_HUMAN]	8.37	1	1	6	50	29.32	30.73	28.91	30.03	29.10	29.65	3	3	29.48	30.80	28.96	29.78	29.32	29.39	29.75	29.50	0.680	0.215	-0.252
P0DMV8	Heat shock 70 kDa protein 1A OS=Homo sapiens GN=HSPA1A PE=1 SV=1 - [HS71A_HUMAN]	65.37	2	20	33	318	29.37	30.26	30.09	30.20	29.53	30.08	3	3	29.54	30.33	30.15	29.96	29.75	29.82	30.00	29.84	0.548	0.182	-0.163
Q92520	Protein FAM3C OS=Homo sapiens GN=FAM3C PE=1 SV=1 - [FAM3C_HUMAN]	22.03	1	4	4	11	21.66	21.83	21.01	21.62	20.82	22.19	3	3	21.82	21.90	21.07	21.38	21.04	21.93	21.60	21.45	0.705	0.220	-0.150
P61106	Ras-related protein Rab-14 OS=Homo sapiens GN=RAB14 PE=1 SV=4 - [RAB14_HUMAN]	50.70	7	7	8	40	26.91	27.35	27.38	27.37	26.68	27.52	3	3	27.08	27.42	27.44	27.12	26.90	27.26	27.31	27.09	0.241	0.095	-0.216
Q9H299	SH3 domain-binding glutamic acid-rich-like protein 3 OS=Homo sapiens GN=SH3BGRL3 PE=1 SV=1 - [SH3L3_HUMAN]	67.74	1	3	3	22	25.13	26.66	26.49	26.01	26.11	26.24	3	3	25.29	26.73	26.55	25.77	26.33	25.98	26.19	26.02	0.750	0.230	-0.164
O95379	Tumor necrosis factor alpha-induced protein 8 OS=Homo sapiens GN=TNFAIP8 PE=1 SV=1 - [TFIP8_HUMAN]	10.10	1	1	1	7	22.69	22.38	22.68	23.07	22.05	22.67	3	3	22.85	22.45	22.73	22.82	22.26	22.41	22.68	22.50	0.433	0.150	-0.180
Q9H2K8	Serine/threonine-protein kinase TAO3 OS=Homo sapiens GN=TAOK3 PE=1 SV=2 - [TAOK3_HUMAN]	7.57	1	5	6	10		21.89	25.55	22.66	24.21	22.88	2	3		21.96	25.61	22.42	24.43	22.62	23.79	23.16	0.718	0.223	-0.631
Q86TI2	Dipeptidyl peptidase 9 OS=Homo sapiens GN=DPP9 PE=1 SV=3 - [DPP9_HUMAN]	1.27	1	1	1	6	22.13	21.35		22.75	20.80	22.24	2	3	22.29	21.42		22.51	21.02	21.98	21.86	21.84	0.978	0.277	-0.020
P31947	14-3-3 protein sigma OS=Homo sapiens GN=SFN PE=1 SV=1 - [1433S_HUMAN]	54.03	1	10	12	83	27.05	28.01	27.96	28.67	26.72	27.46	3	3	27.21	28.07	28.01	28.43	26.94	27.20	27.77	27.52	0.670	0.213	-0.246
Q9HC38	Glyoxalase domain-containing protein 4 OS=Homo sapiens GN=GLOD4 PE=1 SV=1 - [GLOD4_HUMAN]	29.71	1	7	7	18	23.53	23.29	21.79	23.06	21.85	23.87	3	3	23.69	23.36	21.85	22.81	22.06	23.61	22.97	22.83	0.859	0.252	-0.137
P60983	Glia maturation factor beta OS=Homo sapiens GN=GMFB PE=1 SV=2 - [GMFB_HUMAN]	70.42	2	6	6	21	23.75	23.93		24.12	23.51	24.86	2	3	23.91	24.00		23.88	23.73	24.60	23.96	24.07	0.770	0.234	0.112
P07204	Thrombomodulin OS=Homo sapiens GN=THBD PE=1 SV=2 - [TRBM_HUMAN]	10.26	1	3	3	22	23.62	23.75	23.74	23.16		24.25	3	2	23.78	23.82	23.80	22.91		23.99	23.80	23.45	0.453	0.156	-0.345
Q9BZQ8	Protein Niban OS=Homo sapiens GN=FAM129A PE=1 SV=1 - [NIBAN_HUMAN]	15.52	1	10	10	26	23.83	23.75	23.72	24.52	22.39	24.48	3	3	23.99	23.81	23.78	24.27	22.61	24.22	23.86	23.70	0.784	0.237	-0.161
CON_006864501	SWISS-PROT:Q3SZ57 Bos taurus Alpha-fetoprotein precursor	55.08	1	20	25	123	26.87	27.91	26.86	27.59	26.42	27.52	3	3	27.03	27.98	26.92	27.34	26.64	27.26	27.31	27.08	0.605	0.197	-0.227
P46109	Crk-like protein OS=Homo sapiens GN=CRKL PE=1 SV=1 - [CRKL_HUMAN]	26.40	1	6	6	15	23.44	24.18	23.08	23.64	23.58	23.44	3	3	23.60	24.25	23.14	23.40	23.79	23.18	23.66	23.46	0.607	0.197	-0.206
Q9HAV0	Guanine nucleotide-binding protein subunit beta-4 OS=Homo sapiens GN=GNB4 PE=1 SV=3 - [GBB4_HUMAN]	62.06	13	7	15	112	27.86	28.43	28.25	28.19	27.64	28.75	3	3	28.02	28.50	28.30	27.95	27.85	28.49	28.27	28.10	0.505	0.171	-0.178
P78406	mRNA export factor OS=Homo sapiens GN=RAE1 PE=1 SV=1 - [RAE1L_HUMAN]	27.72	2	7	7	16	24.54	23.66	24.43	24.34	24.08	24.22	3	3	24.70	23.72	24.49	24.10	24.30	23.96	24.31	24.12	0.584	0.191	-0.186
P23634	Plasma membrane calcium-transporting ATPase 4 OS=Homo sapiens GN=ATP2B4 PE=1 SV=2 - [AT2B4_HUMAN]	12.81	1	2	13	78	25.56	25.31	25.25	25.93	24.26	25.88	3	3	25.72	25.38	25.31	25.68	24.47	25.62	25.47	25.26	0.635	0.205	-0.212
O75436	Vacuolar protein sorting-associated protein 26A OS=Homo sapiens GN=VPS26A PE=1 SV=2 - [VP26A_HUMAN]	16.82	1	4	4	19	23.08	23.31	23.15	23.67	22.20	23.62	3	3	23.24	23.38	23.21	23.43	22.42	23.36	23.28	23.07	0.555	0.184	-0.212
Q9UIW2	Plexin-A1 OS=Homo sapiens GN=PLXNA1 PE=1 SV=3 - [PLXA1_HUMAN]	15.30	2	15	23	56	23.74	23.84	23.47	24.22	22.54	24.37	3	3	23.90	23.91	23.53	23.97	22.75	24.11	23.78	23.61	0.723	0.224	-0.171
Q8WUM9	Sodium-dependent phosphate transporter 1 OS=Homo sapiens GN=SLC20A1 PE=1 SV=1 - [S20A1_HUMAN]	13.25	1	7	7	14	23.57	21.75	23.60	24.01	21.35		3	2	23.73	21.82	23.66	23.76	21.57		23.07	22.66	0.745	0.229	-0.410
Q8WZ42	Titin OS=Homo sapiens GN=TTN PE=1 SV=4 - [TITIN_HUMAN]	0.03	2	1	2	7	24.30	24.63	24.75	24.78	23.68	25.17	3	3	24.46	24.70	24.81	24.53	23.90	24.91	24.66	24.45	0.542	0.181	-0.208
Q15738	Sterol-4-alpha-carboxylate 3-dehydrogenase, decarboxylating OS=Homo sapiens GN=NSDHL PE=1 SV=2 - [NSDHL_HUMAN]	24.13	1	5	5	14	22.14	22.62		22.90	21.82	23.51	2	3	22.30	22.69		22.65	22.04	23.25	22.49	22.65	0.771	0.234	0.150
CON_006915331	SWISS-PROT:Q28107 Bos taurus Coagulation factor V precursor	20.85	2	35	35	96	25.52	23.62	25.10	24.66	24.27	25.28	3	3	25.68	23.69	25.16	24.42	24.48	25.02	24.84	24.64	0.761	0.233	-0.204
P35527	Keratin, type I cytoskeletal 9 OS=Homo sapiens GN=KRT9 PE=1 SV=3 - [K1C9_HUMAN]	55.86	2	23	24	116	26.13	24.94	25.89	26.42	26.75	23.73	3	3	26.30	25.01	25.95	26.17	26.96	23.47	25.75	25.54	0.858	0.252	-0.215
Q04917	14-3-3 protein eta OS=Homo sapiens GN=YWHAH PE=1 SV=4 - [1433F_HUMAN]	60.57	1	12	14	74	26.35	27.80	27.74	28.61	26.20	27.03	3	3	26.52	27.87	27.80	28.36	26.42	26.77	27.40	27.18	0.787	0.238	-0.215
P68036	Ubiquitin-conjugating enzyme E2 L3 OS=Homo sapiens GN=UBE2L3 PE=1 SV=1 - [UB2L3_HUMAN]	51.95	1	5	5	19	24.43	24.53		25.32	23.60	25.58	2	3	24.59	24.60		25.08	23.81	25.32	24.60	24.74	0.830	0.247	0.141
P53801	Pituitary tumor-transforming gene 1 protein-interacting protein OS=Homo sapiens GN=PTTG1IP PE=1 SV=1 - [PTTG_HUMAN]	13.33	1	2	2	17	23.65	24.93	22.01	23.33	25.27		3	2	23.82	25.00	22.07	23.08	25.49		23.63	24.28	0.676	0.214	0.653
O00468	Agrin OS=Homo sapiens GN=AGRN PE=1 SV=5 - [AGRIN_HUMAN]	10.74	1	14	14	35	22.47	22.23	22.73	23.33	22.13	21.95	3	3	22.63	22.30	22.79	23.08	22.35	21.69	22.57	22.37	0.666	0.212	-0.199
Q9H3Z4	DnaJ homolog subfamily C member 5 OS=Homo sapiens GN=DNAJC5 PE=1 SV=1 - [DNJC5_HUMAN]	14.65	1	2	2	16	23.35	23.62	24.26	24.52	22.51	24.17	3	3	23.51	23.69	24.32	24.27	22.73	23.91	23.84	23.64	0.723	0.224	-0.200
CON_007083981	SWISS-PROT:P02769 Bos taurus Bovine serum albumin precursor	74.46	1	42	47	503	30.34	31.39	29.67	30.85	30.01	30.55	3	3	30.50	31.46	29.73	30.61	30.23	30.29	30.57	30.38	0.734	0.227	-0.188
P62942	Peptidyl-prolyl cis-trans isomerase FKBP1A OS=Homo sapiens GN=FKBP1A PE=1 SV=2 - [FKB1A_HUMAN]	75.93	1	6	6	56	25.43	27.82	28.45	27.40	26.74	27.55	3	3	25.59	27.89	28.51	27.15	26.96	27.29	27.33	27.13	0.838	0.249	-0.195
P63218	Guanine nucleotide-binding protein G(I)/G(S)/G(O) subunit gamma-5 OS=Homo sapiens GN=GNG5 PE=1 SV=3 - [GBG5_HUMAN]	79.41	1	5	5	11			24.81	23.58	23.05	23.94	1	3			24.87	23.33	23.26	23.68	24.87	23.42	NA	NA	-1.446
Q9NS26	Sperm protein associated with the nucleus on the X chromosome A OS=Homo sapiens GN=SPANXA1 PE=1 SV=1 - [SPNXA_HUMAN]	76.29	1	1	4	17	24.82			26.63	26.52	26.54	1	3	24.98			26.38	26.73	26.28	24.98	26.47	NA	NA	1.482
Q9BWJ5	Splicing factor 3B subunit 5 OS=Homo sapiens GN=SF3B5 PE=1 SV=1 - [SF3B5_HUMAN]	70.93	1	5	5	10			22.88	24.24	23.62	23.73	1	3			22.94	23.99	23.83	23.47	22.94	23.76	NA	NA	0.824
O43504	Ragulator complex protein LAMTOR5 OS=Homo sapiens GN=LAMTOR5 PE=1 SV=1 - [LTOR5_HUMAN]	70.33	1	4	4	13		21.67		23.43	23.75		1	2		21.73		23.18	23.97		21.73	23.57	NA	NA	1.840
P61024	Cyclin-dependent kinases regulatory subunit 1 OS=Homo sapiens GN=CKS1B PE=1 SV=1 - [CKS1_HUMAN]	65.82	1	3	3	3		23.73	23.44		22.55		2	1		23.79	23.49		22.77		23.64	22.77	NA	NA	-0.874
Q9NRR8	CDC42 small effector protein 1 OS=Homo sapiens GN=CDC42SE1 PE=1 SV=1 - [C42S1_HUMAN]	62.03	1	2	2	4	22.40	23.19	20.83				3	0	22.56	23.26	20.89				22.24		0.000	0.000	
P62875	DNA-directed RNA polymerases I, II, and III subunit RPABC5 OS=Homo sapiens GN=POLR2L PE=1 SV=1 - [RPAB5_HUMAN]	59.70	1	2	2	3					22.55	21.91	0	2					22.76	21.65		22.21	NA	NA	
O60220	Mitochondrial import inner membrane translocase subunit Tim8 A OS=Homo sapiens GN=TIMM8A PE=1 SV=1 - [TIM8A_HUMAN]	56.70	1	3	3	7				23.72	24.07	24.35	0	3				23.47	24.28	24.09		23.95	0.000	0.000	
Q9Y6W3	Calpain-7 OS=Homo sapiens GN=CAPN7 PE=1 SV=1 - [CAN7_HUMAN]	56.46	1	36	36	102	25.63	25.63	25.62				3	0	25.79	25.70	25.68				25.72		0.000	0.000	
P62841	40S ribosomal protein S15 OS=Homo sapiens GN=RPS15 PE=1 SV=2 - [RS15_HUMAN]	55.17	1	4	4	15			25.25	25.99	27.54	27.42	1	3			25.31	25.74	27.75	27.16	25.31	26.88	NA	NA	1.576
P14854	Cytochrome c oxidase subunit 6B1 OS=Homo sapiens GN=COX6B1 PE=1 SV=2 - [CX6B1_HUMAN]	53.49	1	3	3	5					23.74	24.92	0	2					23.96	24.66		24.31	NA	NA	
P41227	N-alpha-acetyltransferase 10 OS=Homo sapiens GN=NAA10 PE=1 SV=1 - [NAA10_HUMAN]	52.77	2	8	8	20				24.71	24.76	24.97	0	3				24.47	24.98	24.71		24.72	0.000	0.000	
P61960	Ubiquitin-fold modifier 1 OS=Homo sapiens GN=UFM1 PE=1 SV=1 - [UFM1_HUMAN]	50.59	1	2	2	3			23.87		24.02		1	1			23.93		24.23		23.93	24.23	NA	NA	0.302
O75326	Semaphorin-7A OS=Homo sapiens GN=SEMA7A PE=1 SV=1 - [SEM7A_HUMAN]	50.15	1	25	25	68	26.23	26.14	25.23			21.93	3	1	26.39	26.21	25.28			21.67	25.96	21.67	NA	NA	-4.293
O75312	Zinc finger protein ZPR1 OS=Homo sapiens GN=ZPR1 PE=1 SV=1 - [ZPR1_HUMAN]	48.58	1	15	15	41				24.06	24.79	25.96	0	3				23.82	25.01	25.70		24.84	0.000	0.000	
O60925	Prefoldin subunit 1 OS=Homo sapiens GN=PFDN1 PE=1 SV=2 - [PFD1_HUMAN]	48.36	1	5	5	13				23.33	23.28	24.09	0	3				23.08	23.49	23.83		23.47	0.000	0.000	
O15212	Prefoldin subunit 6 OS=Homo sapiens GN=PFDN6 PE=1 SV=1 - [PFD6_HUMAN]	48.06	1	5	5	14		23.23		24.41	24.38	24.80	1	3		23.30		24.16	24.60	24.54	23.30	24.43	NA	NA	1.134
P26885	Peptidyl-prolyl cis-trans isomerase FKBP2 OS=Homo sapiens GN=FKBP2 PE=1 SV=2 - [FKBP2_HUMAN]	47.89	1	4	4	11				23.16	24.56	25.01	0	3				22.92	24.78	24.75		24.15	0.000	0.000	
Q9Y5J7	Mitochondrial import inner membrane translocase subunit Tim9 OS=Homo sapiens GN=TIMM9 PE=1 SV=1 - [TIM9_HUMAN]	47.19	1	3	3	8			21.49		23.87	24.20	1	2			21.55		24.08	23.94	21.55	24.01	NA	NA	2.464
P62273	40S ribosomal protein S29 OS=Homo sapiens GN=RPS29 PE=1 SV=2 - [RS29_HUMAN]	46.43	1	3	3	9				24.79	24.12	25.18	0	3				24.55	24.33	24.92		24.60	0.000	0.000	
Q9UBQ5	Eukaryotic translation initiation factor 3 subunit K OS=Homo sapiens GN=EIF3K PE=1 SV=1 - [EIF3K_HUMAN]	46.33	1	7	7	15	21.78			24.59	23.01	24.56	1	3	21.95			24.34	23.23	24.30	21.95	23.96	NA	NA	2.010
Q96GU1	P antigen family member 5 OS=Homo sapiens GN=PAGE5 PE=1 SV=2 - [PAGE5_HUMAN]	46.15	1	4	4	10				23.09	23.00	24.14	0	3				22.85	23.21	23.88		23.31	0.000	0.000	
Q14061	Cytochrome c oxidase copper chaperone OS=Homo sapiens GN=COX17 PE=1 SV=2 - [COX17_HUMAN]	46.03	1	2	2	2					20.30	22.30	0	2					20.52	22.04		21.28	NA	NA	
O95336	6-phosphogluconolactonase OS=Homo sapiens GN=PGLS PE=1 SV=2 - [6PGL_HUMAN]	45.35	1	6	6	12	24.33	22.44		24.93		23.33	2	2	24.49	22.51		24.69		23.07	23.50	23.88	0.795	0.239	0.379
Q96RS6	NudC domain-containing protein 1 OS=Homo sapiens GN=NUDCD1 PE=1 SV=2 - [NUDC1_HUMAN]	45.11	1	19	19	52			22.94	25.12	25.14	25.54	1	3			23.00	24.87	25.36	25.28	23.00	25.17	NA	NA	2.176
P48047	ATP synthase subunit O, mitochondrial OS=Homo sapiens GN=ATP5O PE=1 SV=1 - [ATPO_HUMAN]	44.60	1	7	7	11		23.23			23.93	23.83	1	2		23.30			24.15	23.57	23.30	23.86	NA	NA	0.560
O60493	Sorting nexin-3 OS=Homo sapiens GN=SNX3 PE=1 SV=3 - [SNX3_HUMAN]	43.21	1	6	7	17	23.41			25.09	24.23	24.32	1	3	23.57			24.84	24.45	24.06	23.57	24.45	NA	NA	0.878
P48730	Casein kinase I isoform delta OS=Homo sapiens GN=CSNK1D PE=1 SV=2 - [KC1D_HUMAN]	42.89	1	6	13	34	23.95	24.61	25.03	22.86			3	1	24.11	24.68	25.09	22.61			24.63	22.61	NA	NA	-2.020
Q0JRZ9	F-BAR domain only protein 2 OS=Homo sapiens GN=FCHO2 PE=1 SV=2 - [FCHO2_HUMAN]	42.22	1	23	23	82	25.09	25.62	25.54			22.23	3	1	25.25	25.69	25.60			21.97	25.51	21.97	NA	NA	-3.543
P03956	Interstitial collagenase OS=Homo sapiens GN=MMP1 PE=1 SV=3 - [MMP1_HUMAN]	42.00	1	19	19	42				25.87	25.23	24.52	0	3				25.62	25.44	24.26		25.11	0.000	0.000	
P13761	HLA class II histocompatibility antigen, DRB1-7 beta chain OS=Homo sapiens GN=HLA-DRB1 PE=1 SV=1 - [2B17_HUMAN]	41.73	2	3	8	24				26.00	25.34	25.94	0	3				25.75	25.56	25.68		25.66	0.000	0.000	
Q96H20	Vacuolar-sorting protein SNF8 OS=Homo sapiens GN=SNF8 PE=1 SV=1 - [SNF8_HUMAN]	41.47	1	7	7	27	24.79	25.16	25.27			23.11	3	1	24.96	25.23	25.33			22.85	25.17	22.85	NA	NA	-2.316

O75368	SH3 domain-binding glutamic acid-rich-like protein OS=Homo sapiens GN=SH3BGRL PE=1 SV=1 - [SH3L1_HUMAN]	41.23	1	4	4	9			20.62	23.67	21.76	23.54	1	3			20.68	23.42	21.97	23.28	20.68	22.89	NA	NA	2.212
P05455	Lupus La protein OS=Homo sapiens GN=SSB PE=1 SV=2 - [LA_HUMAN]	40.44	1	17	17	34		22.44		24.52	24.92	24.68	1	3		22.51		24.27	25.14	24.42	22.51	24.61	NA	NA	2.099
Q9Y333	U6 snRNA-associated Sm-like protein LSm2 OS=Homo sapiens GN=LSM2 PE=1 SV=1 - [LSM2_HUMAN]	40.00	1	3	3	8				23.55	23.00	23.79	0	3				23.31	23.21	23.53		23.35	0.000	0.000	
P04181	Ornithine aminotransferase, mitochondrial OS=Homo sapiens GN=OAT PE=1 SV=1 - [OAT_HUMAN]	39.18	1	10	10	23	21.51			23.32	24.40	24.33	1	3	21.67			23.07	24.62	24.07	21.67	23.92	NA	NA	2.253
Q8WV41	Sorting nexin-33 OS=Homo sapiens GN=SNX33 PE=1 SV=1 - [SNX33_HUMAN]	38.85	1	15	15	46	25.66	24.77	25.23				3	0	25.82	24.84	25.29				25.32		0.000	0.000	
Q99417	C-Myc-binding protein OS=Homo sapiens GN=MYCBP PE=1 SV=3 - [MYCBP_HUMAN]	38.83	1	3	3	6			21.28	24.73	22.16	24.55	1	3			21.34	24.48	22.38	24.28	21.34	23.72	NA	NA	2.377
Q16718	NADH dehydrogenase [ubiquinone] 1 alpha subcomplex subunit 5 OS=Homo sapiens GN=NDUFA5 PE=1 SV=3 - [NDUA5_HUMAN]	38.79	1	3	3	8			22.02	21.06	23.31	23.38	1	3			22.08	20.81	23.53	23.12	22.08	22.49	NA	NA	0.406
P22087	rRNA 2'-O-methyltransferase fibrillarin OS=Homo sapiens GN=FBL PE=1 SV=2 - [FBRL_HUMAN]	38.32	1	8	8	17		23.07		24.31	24.47	24.01	1	3		23.14		24.07	24.69	23.75	23.14	24.17	NA	NA	1.026
Q96GD4	Aurora kinase B OS=Homo sapiens GN=AURKB PE=1 SV=3 - [AURKB_HUMAN]	38.08	2	9	10	24	23.91	24.09	24.38			22.54	3	1	24.07	24.16	24.44			22.28	24.22	22.28	NA	NA	-1.943
Q9Y5X1	Sorting nexin-9 OS=Homo sapiens GN=SNX9 PE=1 SV=1 - [SNX9_HUMAN]	37.98	1	13	13	34	24.17	24.01	24.46				3	0	24.34	24.08	24.52				24.31		0.000	0.000	
P62995	Transformer-2 protein homolog beta OS=Homo sapiens GN=TRA2B PE=1 SV=1 - [TRA2B_HUMAN]	37.85	1	9	9	25	23.65			25.39	25.59	25.30	1	3	23.81			25.15	25.81	25.04	23.81	25.33	NA	NA	1.522
Q96B49	Mitochondrial import receptor subunit TOM6 homolog OS=Homo sapiens GN=TOMM6 PE=1 SV=1 - [TOM6_HUMAN]	37.84	1	1	1	1						24.71	0	1						24.45		24.45	NA	NA	
P06730	Eukaryotic translation initiation factor 4E OS=Homo sapiens GN=EIF4E PE=1 SV=2 - [IF4E_HUMAN]	37.33	1	5	5	12				24.37	21.01	24.60	0	3				24.13	21.23	24.33		23.23	0.000	0.000	
Q5Y7A7	HLA class II histocompatibility antigen, DRB1-13 beta chain OS=Homo sapiens GN=HLA-DRB1 PE=1 SV=1 - [2B1D_HUMAN]	37.22	3	6	8	22				26.19	25.63	26.32	0	3				25.94	25.85	26.06		25.95	0.000	0.000	
Q96QR8	Transcriptional activator protein Pur-beta OS=Homo sapiens GN=PURB PE=1 SV=3 - [PURB_HUMAN]	36.86	2	6	7	22				23.74	23.94	23.49	0	3				23.50	24.16	23.23		23.63	0.000	0.000	
Q9Y5L4	Mitochondrial import inner membrane translocase subunit Tim13 OS=Homo sapiens GN=TIMM13 PE=1 SV=1 - [TIM13_HUMAN]	36.84	1	3	3	7				23.81	24.32	24.69	0	3				23.56	24.53	24.42		24.17	0.000	0.000	
Q30134	HLA class II histocompatibility antigen, DRB1-8 beta chain OS=Homo sapiens GN=HLA-DRB1 PE=1 SV=2 - [2B18_HUMAN]	36.84	1	2	7	24				26.27	25.65	26.30	0	3				26.03	25.87	26.04		25.98	0.000	0.000	
Q9GZT3	SRA stem-loop-interacting RNA-binding protein, mitochondrial OS=Homo sapiens GN=SLIRP PE=1 SV=1 - [SLIRP_HUMAN]	36.70	1	3	3	5					22.83	23.30	0	2					23.05	23.03		23.04	NA	NA	
P48444	Coatomer subunit delta OS=Homo sapiens GN=ARCN1 PE=1 SV=1 - [COPD_HUMAN]	36.59	1	17	17	40	20.99			24.44	23.92	24.36	1	3	21.15			24.20	24.14	24.10	21.15	24.14	NA	NA	2.993
Q8NE71	ATP-binding cassette sub-family F member 1 OS=Homo sapiens GN=ABCF1 PE=1 SV=2 - [ABCF1_HUMAN]	36.57	1	21	21	46	21.24			24.55	24.58	24.76	1	3	21.40			24.31	24.79	24.49	21.40	24.53	NA	NA	3.134
P27695	DNA-(apurinic or apyrimidinic site) lyase OS=Homo sapiens GN=APEX1 PE=1 SV=2 - [APEX1_HUMAN]	36.48	1	8	8	15			21.85	23.34		23.86	1	2			21.91	23.10		23.60	21.91	23.35	NA	NA	1.441
Q9BXJ9	N-alpha-acetyltransferase 15, NatA auxiliary subunit OS=Homo sapiens GN=NAA15 PE=1 SV=1 - [NAA15_HUMAN]	36.26	1	23	26	59				24.64	24.40	24.90	0	3				24.39	24.61	24.64		24.55	0.000	0.000	
O60832	H/ACA ribonucleoprotein complex subunit 4 OS=Homo sapiens GN=DKC1 PE=1 SV=3 - [DKC1_HUMAN]	36.19	1	11	11	24	20.21			24.55	24.00	23.73	1	3	20.38			24.30	24.22	23.47	20.38	24.00	NA	NA	3.621
Q16891	MICOS complex subunit MIC60 OS=Homo sapiens GN=IMMT PE=1 SV=1 - [MIC60_HUMAN]	35.75	1	20	20	33		22.26			24.09	24.35	1	2		22.33			24.31	24.09	22.33	24.20	NA	NA	1.864
Q8N9N7	Leucine-rich repeat-containing protein 57 OS=Homo sapiens GN=LRRC57 PE=1 SV=1 - [LRC57_HUMAN]	35.56	1	6	6	10	23.62	23.24	23.40	22.96			3	1	23.79	23.31	23.46	22.72			23.52	22.72	NA	NA	-0.801
P27694	Replication protein A 70 kDa DNA-binding subunit OS=Homo sapiens GN=RPA1 PE=1 SV=2 - [RFA1_HUMAN]	35.55	1	15	15	34				24.60	24.34	24.25	0	3				24.35	24.56	23.98		24.30	0.000	0.000	
Q9GZL7	Ribosome biogenesis protein WDR12 OS=Homo sapiens GN=WDR12 PE=1 SV=2 - [WDR12_HUMAN]	35.22	1	10	10	23				23.43	24.25	24.17	0	3				23.18	24.46	23.91		23.85	0.000	0.000	
Q9Y2X3	Nucleolar protein 58 OS=Homo sapiens GN=NOP58 PE=1 SV=1 - [NOP58_HUMAN]	35.16	1	15	15	42	21.28			23.96	24.20	23.77	1	3	21.44			23.71	24.42	23.51	21.44	23.88	NA	NA	2.443
Q08752	Peptidyl-prolyl cis-trans isomerase D OS=Homo sapiens GN=PPID PE=1 SV=3 - [PPID_HUMAN]	35.14	1	10	10	30	21.75			24.59	24.47	24.69	1	3	21.91			24.35	24.69	24.43	21.91	24.49	NA	NA	2.574
P04179	Superoxide dismutase [Mn], mitochondrial OS=Homo sapiens GN=SOD2 PE=1 SV=2 - [SODM_HUMAN]	35.14	1	6	6	10		21.89		22.25	22.77	23.46	1	3		21.96		22.01	22.98	23.20	21.96	22.73	NA	NA	0.771
Q9UHV9	Prefoldin subunit 2 OS=Homo sapiens GN=PFDN2 PE=1 SV=1 - [PFD2_HUMAN]	35.06	1	4	4	10				24.85	24.72	24.91	0	3				24.60	24.94	24.65		24.73	0.000	0.000	
P56385	ATP synthase subunit e, mitochondrial OS=Homo sapiens GN=ATP5I PE=1 SV=2 - [ATP5I_HUMAN]	34.78	1	2	2	2					21.76	23.12	0	2					21.97	22.86		22.42	NA	NA	
Q9HAV7	GrpE protein homolog 1, mitochondrial OS=Homo sapiens GN=GRPEL1 PE=1 SV=2 - [GRPE1_HUMAN]	34.10	1	6	6	14			20.15	22.67	23.66	23.51	1	3			20.21	22.43	23.87	23.25	20.21	23.18	NA	NA	2.977
O43324	Eukaryotic translation elongation factor 1 epsilon-1 OS=Homo sapiens GN=EEF1E1 PE=1 SV=1 - [MCA3_HUMAN]	33.91	1	6	6	12	22.59			24.03	24.36	24.33	1	3	22.75			23.78	24.57	24.07	22.75	24.14	NA	NA	1.391
O75935	Dynactin subunit 3 OS=Homo sapiens GN=DCTN3 PE=1 SV=1 - [DCTN3_HUMAN]	33.33	1	4	4	8	22.33			23.27	22.67	21.35	1	3	22.49			23.02	22.88	21.08	22.49	22.33	NA	NA	-0.159
Q9BZM4	NKG2D ligand 3 OS=Homo sapiens GN=ULBP3 PE=1 SV=1 - [N2DL3_HUMAN]	33.20	1	5	5	10	24.03	24.59	23.97				3	0	24.19	24.66	24.03				24.29		0.000	0.000	
Q9UMY4	Sorting nexin-12 OS=Homo sapiens GN=SNX12 PE=1 SV=3 - [SNX12_HUMAN]	33.14	1	4	5	17	22.62			24.17	23.31	23.74	1	3	22.78			23.92	23.53	23.48	22.78	23.64	NA	NA	0.860
P79483	HLA class II histocompatibility antigen, DR beta 3 chain OS=Homo sapiens GN=HLA-DRB3 PE=1 SV=1 - [DRB3_HUMAN]	33.08	1	1	6	19				26.00	25.24	25.94	0	3				25.75	25.46	25.68		25.63	0.000	0.000	
P27105	Erythrocyte band 7 integral membrane protein OS=Homo sapiens GN=STOM PE=1 SV=3 - [STOM_HUMAN]	32.99	2	6	6	10				23.62		22.44	0	2				23.38		22.18		22.78	NA	NA	
Q8NHG7	Small VCP/p97-interacting protein OS=Homo sapiens GN=SVIP PE=1 SV=1 - [SVIP_HUMAN]	32.47	1	2	2	5	23.88	23.61	24.27				3	0	24.04	23.68	24.33				24.01		0.000	0.000	
P55145	Mesencephalic astrocyte-derived neurotrophic factor OS=Homo sapiens GN=MANF PE=1 SV=3 - [MANF_HUMAN]	32.42	1	5	5	11			22.16	22.97	24.56	24.79	1	3			22.22	22.72	24.77	24.53	22.22	24.01	NA	NA	1.783
Q9Y221	60S ribosome subunit biogenesis protein NIP7 homolog OS=Homo sapiens GN=NIP7 PE=1 SV=1 - [NIP7_HUMAN]	32.22	1	3	3	5			23.01	23.45	22.92		1	2			23.07	23.21	23.13		23.07	23.17	NA	NA	0.100
P58340	Myeloid leukemia factor 1 OS=Homo sapiens GN=MLF1 PE=1 SV=1 - [MLF1_HUMAN]	32.09	1	7	7	14	24.86	24.03	24.33				3	0	25.03	24.10	24.39				24.50		0.000	0.000	
O75964	ATP synthase subunit g, mitochondrial OS=Homo sapiens GN=ATP5L PE=1 SV=3 - [ATP5L_HUMAN]	32.04	1	3	3	5			22.72		23.73	23.56	1	2			22.78		23.94	23.30	22.78	23.62	NA	NA	0.843
Q9Y2V2	Calcium-regulated heat stable protein 1 OS=Homo sapiens GN=CARHSP1 PE=1 SV=2 - [CHSP1_HUMAN]	31.97	1	2	2	2					24.21		0	1					24.42			24.42	NA	NA	
O43396	Thioredoxin-like protein 1 OS=Homo sapiens GN=TXNL1 PE=1 SV=3 - [TXNL1_HUMAN]	31.83	1	7	7	22			22.19	24.94	24.49	25.25	1	3			22.25	24.69	24.70	24.99	22.25	24.79	NA	NA	2.545
P35270	Sepiapterin reductase OS=Homo sapiens GN=SPR PE=1 SV=1 - [SPRE_HUMAN]	31.80	1	5	5	11	21.74			23.57	22.40	22.65	1	3	21.90			23.32	22.62	22.39	21.90	22.78	NA	NA	0.879
P12277	Creatine kinase B-type OS=Homo sapiens GN=CKB PE=1 SV=1 - [KCRB_HUMAN]	31.76	1	7	7	17	23.52	23.69	23.59				3	0	23.69	23.76	23.65				23.70		0.000	0.000	
P13762	HLA class II histocompatibility antigen, DR beta 4 chain OS=Homo sapiens GN=HLA-DRB4 PE=1 SV=2 - [DRB4_HUMAN]	31.58	1	5	6	8				24.35	22.93		0	2				24.11	23.15			23.63	NA	NA	
O75223	Gamma-glutamylcyclotransferase OS=Homo sapiens GN=GGCT PE=1 SV=1 - [GGCT_HUMAN]	31.38	1	4	4	7				23.43	21.86	23.40	0	3				23.18	22.07	23.14		22.80	0.000	0.000	
Q9UKM9	RNA-binding protein Raly OS=Homo sapiens GN=RALY PE=1 SV=1 - [RALY_HUMAN]	31.37	2	9	9	16			20.63	23.84	24.36	24.08	1	3			20.69	23.59	24.58	23.82	20.69	24.00	NA	NA	3.310
P55769	NHP2-like protein 1 OS=Homo sapiens GN=NHP2L1 PE=1 SV=3 - [NH2L1_HUMAN]	31.25	1	3	3	11			22.99	24.72	25.04	24.97	1	3			23.05	24.48	25.26	24.71	23.05	24.82	NA	NA	1.765
O00170	AH receptor-interacting protein OS=Homo sapiens GN=AIP PE=1 SV=2 - [AIP_HUMAN]	31.21	1	6	6	17	22.85			23.76	23.76	24.42	1	3	23.01			23.52	23.97	24.16	23.01	23.88	NA	NA	0.874
P42704	Leucine-rich PPR motif-containing protein, mitochondrial OS=Homo sapiens GN=LRPPRC PE=1 SV=3 - [LPPRC_HUMAN]	30.63	1	34	34	67			21.52	23.56	23.53	24.88	1	3			21.58	23.31	23.75	24.62	21.58	23.89	NA	NA	2.316
P24752	Acetyl-CoA acetyltransferase, mitochondrial OS=Homo sapiens GN=ACAT1 PE=1 SV=1 - [THIL_HUMAN]	30.21	1	9	9	27				23.98	24.45	24.83	0	3				23.73	24.67	24.57		24.32	0.000	0.000	
P55327	Tumor protein D52 OS=Homo sapiens GN=TPD52 PE=1 SV=2 - [TPD52_HUMAN]	29.91	1	4	4	10		21.44		24.15	23.43	23.77	1	3		21.51		23.90	23.64	23.50	21.51	23.68	NA	NA	2.173
Q86XT2	Vacuolar protein sorting-associated protein 37D OS=Homo sapiens GN=VPS37D PE=1 SV=2 - [VP37D_HUMAN]	29.88	1	6	6	10	22.79	22.09	23.75				3	0	22.95	22.16	23.81				22.97		0.000	0.000	
O75348	V-type proton ATPase subunit G 1 OS=Homo sapiens GN=ATP6V1G1 PE=1 SV=3 - [VATG1_HUMAN]	29.66	2	3	3	7				23.45	23.35	23.33	0	3				23.21	23.57	23.07		23.28	0.000	0.000	
P16035	Metalloproteinase inhibitor 2 OS=Homo sapiens GN=TIMP2 PE=1 SV=2 - [TIMP2_HUMAN]	29.55	1	4	4	9				22.57	23.29	22.97	0	3				22.32	23.50	22.71		22.84	0.000	0.000	
P19388	DNA-directed RNA polymerases I, II, and III subunit RPABC1 OS=Homo sapiens GN=POLR2E PE=1 SV=4 - [RPAB1_HUMAN]	29.05	1	4	4	7	21.94			22.06	23.50	22.38	1	3	22.10			21.82	23.71	22.12	22.10	22.55	NA	NA	0.447
Q9NX24	H/ACA ribonucleoprotein complex subunit 2 OS=Homo sapiens GN=NHP2 PE=1 SV=1 - [NHP2_HUMAN]	28.76	1	2	2	7				23.87	24.35		0	2				23.63	24.56			24.10	NA	NA	
O14933	Ubiquitin/ISG15-conjugating enzyme E2 L6 OS=Homo sapiens GN=UBE2L6 PE=1 SV=4 - [UB2L6_HUMAN]	28.76	1	2	2	2					22.39		0	1					22.61			22.61	NA	NA	
O43598	2'-deoxynucleoside 5'-phosphate N-hydrolase 1 OS=Homo sapiens GN=DNPH1 PE=1 SV=1 - [DNPH1_HUMAN]	28.74	1	2	2	7	22.77	21.06	19.44			22.04	3	1	22.93	21.13	19.50			21.78	21.19	21.78	NA	NA	0.597
Q12905	Interleukin enhancer-binding factor 2 OS=Homo sapiens GN=ILF2 PE=1 SV=2 - [ILF2_HUMAN]	28.72	1	6	6	17				24.83	24.50	24.77	0	3				24.59	24.71	24.51		24.61	0.000	0.000	
Q00577	Transcriptional activator protein Pur-alpha OS=Homo sapiens GN=PURA PE=1 SV=2 - [PURA_HUMAN]	28.57	1	6	7	16				23.72	23.98	22.55	0	3				23.47	24.20	22.29		23.32	0.000	0.000	
Q13253	Noggin OS=Homo sapiens GN=NOG PE=1 SV=1 - [NOGG_HUMAN]	28.45	1	3	3	8			25.17	26.18	26.10		1	2			25.23	25.94	26.31		25.23	26.12	NA	NA	0.897
Q13158	FAS-associated death domain protein OS=Homo sapiens GN=FADD PE=1 SV=1 - [FADD_HUMAN]	28.37	1	2	2	6			22.28	23.74	23.25		1	2			22.34	23.50	23.46		22.34	23.48	NA	NA	1.142
Q12904	Aminoacyl tRNA synthase complex-interacting multifunctional protein 1 OS=Homo sapiens GN=AIMP1 PE=1 SV=2 - [AIMP1_HUMAN]	28.21	1	7	7	21	23.26			24.42	24.22	24.39	1	3	23.42			24.17	24.44	24.13	23.42	24.25	NA	NA	0.826
P21579	Synaptotagmin-1 OS=Homo sapiens GN=SYT1 PE=1 SV=1 - [SYT1_HUMAN]	28.20	3	8	8	12	22.32	23.21	23.04				3	0	22.48	23.28	23.10				22.95		0.000	0.000	
P18077	60S ribosomal protein L35a OS=Homo sapiens GN=RPL35A PE=1 SV=2 - [RL35A_HUMAN]	28.18	1	6	6	18			23.48	25.12	25.74	25.97	1	3			23.54	24.87	25.96	25.71	23.54	25.51	NA	NA	1.974
O75390	Citrate synthase, mitochondrial OS=Homo sapiens GN=CS PE=1 SV=2 - [CISY_HUMAN]	28.11	1	7	7	16				24.15	23.97	24.77	0	3				23.90	24.19	24.51		24.20	0.000	0.000	
P54819	Adenylate kinase 2, mitochondrial OS=Homo sapiens GN=AK2 PE=1 SV=2 - [KAD2_HUMAN]	28.03	1	4	4	15				23.90	23.50	24.39	0	3				23.65	23.72	24.13		23.83	0.000	0.000	
Q8WUW1	Protein BRICK1 OS=Homo sapiens GN=BRK1 PE=1 SV=1 - [BRK1_HUMAN]	28.00	1	2	2	5	23.24	22.65	22.90			24.06	3	1	23.41	22.72	22.96			23.80	23.03	23.80	NA	NA	0.769
P09669	Cytochrome c oxidase subunit 6C OS=Homo sapiens GN=COX6C PE=1 SV=2 - [COX6C_HUMAN]	28.00	1	3	3	6			23.07		22.86	23.43	1	2			23.13		23.08	23.17	23.13	23.12	NA	NA	-0.007
P01903	HLA class II histocompatibility antigen, DR alpha chain OS=Homo sapiens GN=HLA-DRA PE=1 SV=1 - [DRA_HUMAN]	27.95	2	7	7	29				27.06	26.38	26.89	0	3				26.82	26.60	26.63		26.68	0.000	0.000	
P10606	Cytochrome c oxidase subunit 5B, mitochondrial OS=Homo sapiens GN=COX5B PE=1 SV=2 - [COX5B_HUMAN]	27.91	1	3	3	8			23.49		24.53	25.30	1	2			23.55		24.74	25.04	23.55	24.89	NA	NA	1.341
Q16836	Hydroxyacyl-coenzyme A dehydrogenase, mitochondrial OS=Homo sapiens GN=HADH PE=1 SV=3 - [HCDH_HUMAN]	27.71	2	6	6	11					23.83	23.65	0	2					24.05	23.39		23.72	NA	NA	
Q96FX7	tRNA (adenine(58)-N(1))-methyltransferase catalytic subunit TRMT61A OS=Homo sapiens GN=TRMT61A PE=1 SV=1 - [TRM61_HUMAN]	27.68	1	5	5	8				24.12	24.14		0	2				23.88	24.35			24.11	NA	NA	
Q9H9B4	Sideroflexin-1 OS=Homo sapiens GN=SFXN1 PE=1 SV=4 - [SFXN1_HUMAN]	27.64	2	7	7	10					22.94	23.42	0	2					23.15	23.16		23.16	NA	NA	
Q00059	Transcription factor A, mitochondrial OS=Homo sapiens GN=TFAM PE=1 SV=1 - [TFAM_HUMAN]	27.64	1	7	7	12					23.66	23.58	0	2					23.88	23.32		23.60	NA	NA	
Q8WUH6	Transmembrane protein 263 OS=Homo sapiens GN=TMEM263 PE=1 SV=1 - [TM263_HUMAN]	27.59	1	2	2	3	21.87				22.34		1	1	22.03				22.56		22.03	22.56	NA	NA	0.527
Q15388	Mitochondrial import receptor subunit TOM20 homolog OS=Homo sapiens GN=TOMM20 PE=1 SV=1 - [TOM20_HUMAN]	27.59	1	3	3	9				22.18	23.61	23.85	0	3				21.93	23.82	23.59		23.11	0.000	0.000	
P26583	High mobility group protein B2 OS=Homo sapiens GN=HMGB2 PE=1 SV=2 - [HMGB2_HUMAN]	27.27	1	3	4	6				21.89	23.63	23.48	0	3				21.64	23.85	23.22		22.90	0.000	0.000	
Q01658	Protein Dr1 OS=Homo sapiens GN=DR1 PE=1 SV=1 - [NC2B_HUMAN]	27.27	1	2	2	6				23.70	23.52	23.52	0	3				23.45	23.73	23.26		23.48	0.000	0.000	
O60869	Endothelial differentiation-related factor 1 OS=Homo sapiens GN=EDF1 PE=1 SV=1 - [EDF1_HUMAN]	27.03	1	3	3	8		23.25	23.07				2	0		23.32	23.13				23.22		NA	NA	
P15954	Cytochrome c oxidase subunit 7C, mitochondrial OS=Homo sapiens GN=COX7C PE=1 SV=1 - [COX7C_HUMAN]	26.98	1	1	1	4					22.30	22.71	0	2					22.52	22.44		22.48	NA	NA	
Q8NEZ2	Vacuolar protein sorting-associated protein 37A OS=Homo sapiens GN=VPS37A PE=1 SV=1 - [VP37A_HUMAN]	26.95	1	8	8	18	24.07	23.83	23.58	21.65			3	1	24.23	23.90	23.64	21.40			23.92	21.40	NA	NA	-2.519
O75534	Cold shock domain-containing protein E1 OS=Homo sapiens GN=CSDE1 PE=1 SV=2 - [CSDE1_HUMAN]	26.69	1	17	17	43	21.38			23.61	24.07	24.18	1	3	21.54			23.36	24.28	23.92	21.54	23.85	NA	NA	2.315
O15355	Protein phosphatase 1G OS=Homo sapiens GN=PPM1G PE=1 SV=1 - [PPM1G_HUMAN]	26.19	1	8	8	14				22.73	22.99	23.73	0	3				22.49	23.21	23.47		23.06	0.000	0.000	
Q13794	Phorbol-12-myristate-13-acetate-induced protein 1 OS=Homo sapiens GN=PMAIP1 PE=1 SV=1 - [APR_HUMAN]	25.93	1	1	1	2				22.47	21.73		0	2				22.22	21.95			22.08	NA	NA	
P50583	Bis(5'-nucleosyl)-tetraphosphatase [asymmetrical] OS=Homo sapiens GN=NUDT2 PE=1 SV=3 - [AP4A_HUMAN]	25.85	1	2	2	2						23.07	0	1						22.80		22.80	NA	NA	
O75821	Eukaryotic translation initiation factor 3 subunit G OS=Homo sapiens GN=EIF3G PE=1 SV=2 - [EIF3G_HUMAN]	25.62	1	6	6	15	22.48			24.27	24.29	24.69	1	3	22.64			24.02	24.51	24.42	22.64	24.32	NA	NA	1.675
Q32P28	Prolyl 3-hydroxylase 1 OS=Homo sapiens GN=P3H1 PE=1 SV=2 - [P3H1_HUMAN]	25.54	1	11	11	18				21.80	23.51	24.46	0	3				21.56	23.73	24.20		23.16	0.000	0.000	
CON_006887832	SWISS-PROT:Q3Y5Z3 Bos taurus Adiponectin precursor	25.42	1	5	5	17	23.33			24.44	23.83	24.27	1	3	23.49			24.19	24.05	24.01	23.49	24.08	NA	NA	0.593
Q9C005	Protein dpy-30 homolog OS=Homo sapiens GN=DPY30 PE=1 SV=1 - [DPY30_HUMAN]	25.25	1	2	2	4				23.24		22.71	0	2				22.99		22.45		22.72	NA	NA	
P47224	Guanine nucleotide exchange factor MSS4 OS=Homo sapiens GN=RABIF PE=1 SV=2 - [MSS4_HUMAN]	25.20	1	2	2	4		23.33	23.27	22.96			2	1		23.40	23.33	22.71			23.36	22.71	NA	NA	-0.651
Q9P2T1	GMP reductase 2 OS=Homo sapiens GN=GMPR2 PE=1 SV=1 - [GMPR2_HUMAN]	25.00	2	6	6	10				23.18	22.22	23.23	0	3				22.94	22.44	22.97		22.78	0.000	0.000	
Q9NPE3	H/ACA ribonucleoprotein complex subunit 3 OS=Homo sapiens GN=NOP10 PE=1 SV=1 - [NOP10_HUMAN]	25.00	1	1	1	5			21.88	23.13	23.33	23.53	1	3			21.94	22.88	23.55	23.27	21.94	23.23	NA	NA	1.292
Q96S44	TP53-regulating kinase OS=Homo sapiens GN=TP53RK PE=1 SV=2 - [PRPK_HUMAN]	24.90	1	5	5	7				23.64	23.52		0	2				23.39	23.73			23.56	NA	NA	
Q9NZD2	Glycolipid transfer protein OS=Homo sapiens GN=GLTP PE=1 SV=3 - [GLTP_HUMAN]	24.88	1	3	3	6		22.44	22.33				2	0		22.51	22.39				22.45		NA	NA	
Q9NRY6	Phospholipid scramblase 3 OS=Homo sapiens GN=PLSCR3 PE=1 SV=2 - [PLS3_HUMAN]	24.75	1	5	5	10	22.54	23.32	23.27			22.98	3	1	22.71	23.39	23.33			22.72	23.14	22.72	NA	NA	-0.421
Q9BTT0	Acidic leucine-rich nuclear phosphoprotein 32 family member E OS=Homo sapiens GN=ANP32E PE=1 SV=1 - [AN32E_HUMAN]	24.63	1	3	3	7				23.84	24.65	24.76	0	3				23.60	24.87	24.50		24.32	0.000	0.000	
Q9NQP4	Prefoldin subunit 4 OS=Homo sapiens GN=PFDN4 PE=1 SV=1 - [PFD4_HUMAN]	24.63	1	3	3	7		22.46		24.05	24.50	24.45	1	3		22.53		23.80	24.72	24.19	22.53	24.24	NA	NA	1.708
P10515	Dihydrolipoyllysine-residue acetyltransferase component of pyruvate dehydrogenase complex, mitochondrial OS=Homo sapiens GN=DLAT PE=1 SV=3 - [ODP2_HUMAN]	24.57	1	11	11	22				22.86	24.65	24.97	0	3				22.61	24.86	24.71		24.06	0.000	0.000	
Q9UEU0	Vesicle transport through interaction with t-SNAREs homolog 1B OS=Homo sapiens GN=VTI1B PE=1 SV=3 - [VTI1B_HUMAN]	24.57	1	4	4	7				22.85		21.63	0	2				22.60		21.37		21.99	NA	NA	
Q15397	Pumilio domain-containing protein KIAA0020 OS=Homo sapiens GN=KIAA0020 PE=1 SV=3 - [K0020_HUMAN]	24.54	1	10	10	17				24.35	23.66	23.74	0	3				24.11	23.88	23.48		23.82	0.000	0.000	
Q96DR4	StAR-related lipid transfer protein 4 OS=Homo sapiens GN=STARD4 PE=2 SV=1 - [STAR4_HUMAN]	24.39	1	3	3	5	22.97	22.60	23.63				3	0	23.13	22.67	23.69				23.16		0.000	0.000	
P49674	Casein kinase I isoform epsilon OS=Homo sapiens GN=CSNK1E PE=1 SV=1 - [KC1E_HUMAN]	24.28	1	1	8	24	23.60	24.30	24.98	22.86			3	1	23.76	24.37	25.04	22.61			24.39	22.61	NA	NA	-1.782
P04216	Thy-1 membrane glycoprotein OS=Homo sapiens GN=THY1 PE=1 SV=2 - [THY1_HUMAN]	24.22	1	3	3	12				25.42	24.29	25.69	0	3				25.18	24.51	25.43		25.04	0.000	0.000	
P00403	Cytochrome c oxidase subunit 2 OS=Homo sapiens GN=MT-CO2 PE=1 SV=1 - [COX2_HUMAN]	23.79	1	6	6	13			23.72		23.66	24.99	1	2			23.78		23.87	24.73	23.78	24.30	NA	NA	0.524
Q92769	Histone deacetylase 2 OS=Homo sapiens GN=HDAC2 PE=1 SV=2 - [HDAC2_HUMAN]	23.77	1	4	7	13	22.49			22.84	23.53	23.98	1	3	22.65			22.59	23.74	23.72	22.65	23.35	NA	NA	0.705
P62312	U6 snRNA-associated Sm-like protein LSm6 OS=Homo sapiens GN=LSM6 PE=1 SV=1 - [LSM6_HUMAN]	23.75	1	2	2	5				22.17	21.71	22.30	0	3				21.92	21.92	22.04		21.96	0.000	0.000	
Q9P0L0	Vesicle-associated membrane protein-associated protein A OS=Homo sapiens GN=VAPA PE=1 SV=3 - [VAPA_HUMAN]	23.69	2	4	4	11	22.33			23.11	22.91	24.25	1	3	22.49			22.87	23.12	23.99	22.49	23.33	NA	NA	0.841
Q9Y5V0	Zinc finger protein 706 OS=Homo sapiens GN=ZNF706 PE=1 SV=1 - [ZN706_HUMAN]	23.68	1	2	2	2					20.94		0	1					21.16			21.16	NA	NA	
Q08J23	tRNA (cytosine(34)-C(5))-methyltransferase OS=Homo sapiens GN=NSUN2 PE=1 SV=2 - [NSUN2_HUMAN]	23.60	2	14	14	25				23.49	23.66	23.18	0	3				23.25	23.88	22.91		23.35	0.000	0.000	
Q9HDC9	Adipocyte plasma membrane-associated protein OS=Homo sapiens GN=APMAP PE=1 SV=2 - [APMAP_HUMAN]	23.56	1	7	7	14				24.21	25.22	25.16	0	3				23.97	25.43	24.90		24.76	0.000	0.000	
P00374	Dihydrofolate reductase OS=Homo sapiens GN=DHFR PE=1 SV=2 - [DYR_HUMAN]	23.53	2	3	3	11	23.66	23.13	22.94		21.68		3	1	23.82	23.20	23.00		21.90		23.34	21.90	NA	NA	-1.446
O14817	Tetraspanin-4 OS=Homo sapiens GN=TSPAN4 PE=1 SV=1 - [TSN4_HUMAN]	23.53	1	3	3	18	27.31	27.42	27.97			25.29	3	1	27.47	27.49	28.03			25.03	27.66	25.03	NA	NA	-2.636
P35354	Prostaglandin G/H synthase 2 OS=Homo sapiens GN=PTGS2 PE=1 SV=2 - [PGH2_HUMAN]	23.51	2	10	10	16				24.04	23.90	24.69	0	3				23.80	24.11	24.43		24.11	0.000	0.000	
Q96DI7	U5 small nuclear ribonucleoprotein 40 kDa protein OS=Homo sapiens GN=SNRNP40 PE=1 SV=1 - [SNR40_HUMAN]	23.25	13	3	4	7				22.66	22.65	20.29	0	3				22.42	22.87	20.02		21.77	0.000	0.000	
P07311	Acylphosphatase-1 OS=Homo sapiens GN=ACYP1 PE=1 SV=2 - [ACYP1_HUMAN]	23.23	1	2	2	2						22.86	0	1						22.60		22.60	NA	NA	
Q12834	Cell division cycle protein 20 homolog OS=Homo sapiens GN=CDC20 PE=1 SV=2 - [CDC20_HUMAN]	23.05	1	6	6	16	23.72	22.50	22.48		19.10		3	1	23.88	22.57	22.54		19.31		23.00	19.31	NA	NA	-3.682
A0AVT1	Ubiquitin-like modifier-activating enzyme 6 OS=Homo sapiens GN=UBA6 PE=1 SV=1 - [UBA6_HUMAN]	22.72	1	18	18	38			21.25	23.73	23.62	23.92	1	3			21.31	23.48	23.83	23.66	21.31	23.66	NA	NA	2.349
O75934	Pre-mRNA-splicing factor SPF27 OS=Homo sapiens GN=BCAS2 PE=1 SV=1 - [SPF27_HUMAN]	22.67	1	2	2	4				22.45	22.36		0	2				22.20	22.58			22.39	NA	NA	
Q9NRR3	CDC42 small effector protein 2 OS=Homo sapiens GN=CDC42SE2 PE=1 SV=1 - [C42S2_HUMAN]	22.62	1	1	1	1	22.58						1	0	22.74						22.74		NA	NA	
Q08945	FACT complex subunit SSRP1 OS=Homo sapiens GN=SSRP1 PE=1 SV=1 - [SSRP1_HUMAN]	22.57	1	9	9	19				23.69	24.20	23.49	0	3				23.45	24.41	23.23		23.70	0.000	0.000	

O15347	High mobility group protein B3 OS=Homo sapiens GN=HMGB3 PE=1 SV=4 - [HMGB3_HUMAN]	22.50	1	3	3	4				23.49		24.28	0	2				23.24		24.02		23.63	NA	NA	
Q9UNZ2	NSFL1 cofactor p47 OS=Homo sapiens GN=NSFL1C PE=1 SV=2 - [NSF1C_HUMAN]	22.43	1	6	6	12		21.35		22.98	22.58	22.86	1	3		21.41		22.73	22.79	22.60	21.41	22.71	NA	NA	1.295
Q9NSD9	Phenylalanine--tRNA ligase beta subunit OS=Homo sapiens GN=FARSB PE=1 SV=3 - [SYFB_HUMAN]	22.41	1	10	10	15				23.31	24.55	22.92	0	3				23.07	24.77	22.66		23.50	0.000	0.000	
P23297	Protein S100-A1 OS=Homo sapiens GN=S100A1 PE=1 SV=2 - [S10A1_HUMAN]	22.34	1	1	1	1	19.24						1	0	19.41						19.41		NA	NA	
Q9BT09	Protein canopy homolog 3 OS=Homo sapiens GN=CNPY3 PE=1 SV=1 - [CNPY3_HUMAN]	22.30	1	5	5	6					22.84	23.55	0	2					23.06	23.29		23.17	NA	NA	
Q13242	Serine/arginine-rich splicing factor 9 OS=Homo sapiens GN=SRSF9 PE=1 SV=1 - [SRSF9_HUMAN]	22.17	1	5	5	9	21.02			23.32	22.94	23.49	1	3	21.19			23.08	23.16	23.23	21.19	23.15	NA	NA	1.967
Q6P5R6	60S ribosomal protein L22-like 1 OS=Homo sapiens GN=RPL22L1 PE=1 SV=2 - [RL22L_HUMAN]	22.13	1	2	3	4					23.05	22.04	0	2					23.27	21.78		22.52	NA	NA	
P02686	Myelin basic protein OS=Homo sapiens GN=MBP PE=1 SV=3 - [MBP_HUMAN]	22.04	1	5	5	7		22.33	22.31	22.16			2	1		22.40	22.37	21.91			22.39	21.91	NA	NA	-0.474
Q9UN86	Ras GTPase-activating protein-binding protein 2 OS=Homo sapiens GN=G3BP2 PE=1 SV=2 - [G3BP2_HUMAN]	21.99	1	6	6	12				22.51	23.49	22.99	0	3				22.27	23.71	22.72		22.90	0.000	0.000	
Q1KMD3	Heterogeneous nuclear ribonucleoprotein U-like protein 2 OS=Homo sapiens GN=HNRNPUL2 PE=1 SV=1 - [HNRL2_HUMAN]	21.95	1	11	11	25				23.70	23.84	24.07	0	3				23.45	24.05	23.81		23.77	0.000	0.000	
Q9H0W9	Ester hydrolase C11orf54 OS=Homo sapiens GN=C11orf54 PE=1 SV=1 - [CK054_HUMAN]	21.90	1	5	5	8				23.18	22.51	23.47	0	3				22.93	22.72	23.21		22.96	0.000	0.000	
P25325	3-mercaptopyruvate sulfurtransferase OS=Homo sapiens GN=MPST PE=1 SV=3 - [THTM_HUMAN]	21.89	1	4	4	11	22.02			23.34	23.42	23.91	1	3	22.18			23.09	23.63	23.65	22.18	23.46	NA	NA	1.281
Q8N183	Mimitin, mitochondrial OS=Homo sapiens GN=NDUFAF2 PE=1 SV=1 - [MIMIT_HUMAN]	21.89	1	3	3	5			20.69	21.67	21.91	21.69	1	3			20.75	21.42	22.12	21.43	20.75	21.66	NA	NA	0.909
O00165	HCLS1-associated protein X-1 OS=Homo sapiens GN=HAX1 PE=1 SV=2 - [HAX1_HUMAN]	21.86	1	4	4	8	23.52	22.61	22.73				3	0	23.68	22.68	22.79				23.05		0.000	0.000	
O75204	Transmembrane protein 127 OS=Homo sapiens GN=TMEM127 PE=1 SV=1 - [TM127_HUMAN]	21.85	1	2	2	2				22.64		22.99	0	2				22.39		22.73		22.56	NA	NA	
P19367	Hexokinase-1 OS=Homo sapiens GN=HK1 PE=1 SV=3 - [HXK1_HUMAN]	21.81	1	14	17	28			22.00	22.51	22.93	24.01	1	3			22.06	22.26	23.14	23.75	22.06	23.05	NA	NA	0.993
Q8TDB6	E3 ubiquitin-protein ligase DTX3L OS=Homo sapiens GN=DTX3L PE=1 SV=1 - [DTX3L_HUMAN]	21.76	1	11	11	19	23.26	23.35	23.35				3	0	23.42	23.41	23.41				23.42		0.000	0.000	
P21741	Midkine OS=Homo sapiens GN=MDK PE=1 SV=1 - [MK_HUMAN]	21.68	1	3	3	5				23.93	22.85	23.08	0	3				23.68	23.06	22.82		23.19	0.000	0.000	
Q9NY12	H/ACA ribonucleoprotein complex subunit 1 OS=Homo sapiens GN=GAR1 PE=1 SV=1 - [GAR1_HUMAN]	21.66	1	4	4	8				25.60	24.39	25.33	0	3				25.36	24.60	25.07		25.01	0.000	0.000	
Q15050	Ribosome biogenesis regulatory protein homolog OS=Homo sapiens GN=RRS1 PE=1 SV=2 - [RRS1_HUMAN]	21.64	1	4	4	7				23.76	22.30	22.67	0	3				23.51	22.51	22.41		22.81	0.000	0.000	
P18754	Regulator of chromosome condensation OS=Homo sapiens GN=RCC1 PE=1 SV=1 - [RCC1_HUMAN]	21.62	1	5	5	10				23.52	23.26	23.76	0	3				23.27	23.47	23.50		23.42	0.000	0.000	
P18031	Tyrosine-protein phosphatase non-receptor type 1 OS=Homo sapiens GN=PTPN1 PE=1 SV=1 - [PTN1_HUMAN]	21.61	1	7	7	10					23.30	23.40	0	2					23.51	23.14		23.33	NA	NA	
P42166	Lamina-associated polypeptide 2, isoform alpha OS=Homo sapiens GN=TMPO PE=1 SV=2 - [LAP2A_HUMAN]	21.47	1	6	8	14		22.35		23.77	24.18	23.77	1	3		22.42		23.53	24.39	23.51	22.42	23.81	NA	NA	1.388
O00214	Galectin-8 OS=Homo sapiens GN=LGALS8 PE=1 SV=4 - [LEG8_HUMAN]	21.45	1	6	6	9	23.32	22.55	22.04	23.25			3	1	23.48	22.62	22.10	23.01			22.74	23.01	NA	NA	0.272
Q56VL3	OCIA domain-containing protein 2 OS=Homo sapiens GN=OCIAD2 PE=1 SV=1 - [OCAD2_HUMAN]	21.43	1	3	3	4			20.69		21.85	22.65	1	2			20.75		22.07	22.39	20.75	22.23	NA	NA	1.481
Q9H1E3	Nuclear ubiquitous casein and cyclin-dependent kinase substrate 1 OS=Homo sapiens GN=NUCKS1 PE=1 SV=1 - [NUCKS_HUMAN]	21.40	1	4	4	11	21.38			23.48	23.70	24.62	1	3	21.54			23.23	23.92	24.36	21.54	23.84	NA	NA	2.297
Q12874	Splicing factor 3A subunit 3 OS=Homo sapiens GN=SF3A3 PE=1 SV=1 - [SF3A3_HUMAN]	21.36	1	6	6	13				23.74	24.07	23.92	0	3				23.50	24.29	23.66		23.82	0.000	0.000	
P00750	Tissue-type plasminogen activator OS=Homo sapiens GN=PLAT PE=1 SV=1 - [TPA_HUMAN]	21.35	1	8	8	15			21.79	23.66	24.33	23.87	1	3			21.85	23.42	24.55	23.61	21.85	23.86	NA	NA	2.007
Q9UBT2	SUMO-activating enzyme subunit 2 OS=Homo sapiens GN=UBA2 PE=1 SV=2 - [SAE2_HUMAN]	21.25	1	7	7	12				22.28	23.90	22.61	0	3				22.04	24.12	22.35		22.83	0.000	0.000	
Q9H7B2	Ribosome production factor 2 homolog OS=Homo sapiens GN=RPF2 PE=1 SV=2 - [RPF2_HUMAN]	21.24	1	5	5	9				24.22	23.38	23.72	0	3				23.98	23.59	23.46		23.67	0.000	0.000	
Q9UBS4	DnaJ homolog subfamily B member 11 OS=Homo sapiens GN=DNAJB11 PE=1 SV=1 - [DJB11_HUMAN]	21.23	1	6	6	15	22.23			22.34	24.06	24.32	1	3	22.39			22.10	24.28	24.06	22.39	23.48	NA	NA	1.085
O43181	NADH dehydrogenase [ubiquinone] iron-sulfur protein 4, mitochondrial OS=Homo sapiens GN=NDUFS4 PE=1 SV=1 - [NDUS4_HUMAN]	21.14	1	2	2	4	24.10			25.55		23.15	1	2	24.26			25.31		22.89	24.26	24.10	NA	NA	-0.160
Q12849	G-rich sequence factor 1 OS=Homo sapiens GN=GRSF1 PE=1 SV=3 - [GRSF1_HUMAN]	21.04	1	5	5	9				22.45	23.38	23.06	0	3				22.20	23.59	22.80		22.86	0.000	0.000	
Q14232	Translation initiation factor eIF-2B subunit alpha OS=Homo sapiens GN=EIF2B1 PE=1 SV=1 - [EI2BA_HUMAN]	20.98	1	5	5	12			22.61	23.52	22.98	24.05	1	3			22.67	23.27	23.20	23.79	22.67	23.42	NA	NA	0.747
P25205	DNA replication licensing factor MCM3 OS=Homo sapiens GN=MCM3 PE=1 SV=3 - [MCM3_HUMAN]	20.92	1	13	13	19		19.06			23.76	22.65	1	2		19.13			23.97	22.39	19.13	23.18	NA	NA	4.050
P08574	Cytochrome c1, heme protein, mitochondrial OS=Homo sapiens GN=CYC1 PE=1 SV=3 - [CY1_HUMAN]	20.92	1	4	4	10			23.62		23.93	24.88	1	2			23.68		24.15	24.62	23.68	24.38	NA	NA	0.707
P05121	Plasminogen activator inhibitor 1 OS=Homo sapiens GN=SERPINE1 PE=1 SV=1 - [PAI1_HUMAN]	20.90	1	6	6	20	23.61		22.63	24.20	24.67		2	2	23.77		22.69	23.96	24.88		23.23	24.42	0.237	0.094	1.187
Q9Y4Y9	U6 snRNA-associated Sm-like protein LSm5 OS=Homo sapiens GN=LSM5 PE=1 SV=3 - [LSM5_HUMAN]	20.88	1	1	1	2					23.03		0	1					23.25			23.25	NA	NA	
Q13630	GDP-L-fucose synthase OS=Homo sapiens GN=TSTA3 PE=1 SV=1 - [FCL_HUMAN]	20.87	1	5	5	8	21.67			23.64	21.89	23.17	1	3	21.83			23.40	22.11	22.91	21.83	22.81	NA	NA	0.975
P49419	Alpha-aminoadipic semialdehyde dehydrogenase OS=Homo sapiens GN=ALDH7A1 PE=1 SV=5 - [AL7A1_HUMAN]	20.78	1	8	8	11		22.81			23.60	23.56	1	2		22.88			23.82	23.30	22.88	23.56	NA	NA	0.677
Q9H936	Mitochondrial glutamate carrier 1 OS=Homo sapiens GN=SLC25A22 PE=1 SV=1 - [GHC1_HUMAN]	20.74	2	4	4	6					22.48	23.89	0	2					22.69	23.63		23.16	NA	NA	
Q9NWV4	UPF0587 protein C1orf123 OS=Homo sapiens GN=C1orf123 PE=1 SV=1 - [CA123_HUMAN]	20.63	1	2	2	4				23.51	23.10	23.74	0	3				23.26	23.32	23.47		23.35	0.000	0.000	
Q02818	Nucleobindin-1 OS=Homo sapiens GN=NUCB1 PE=1 SV=4 - [NUCB1_HUMAN]	20.61	1	7	7	11				23.30	23.29	23.21	0	3				23.05	23.51	22.95		23.17	0.000	0.000	
Q9UNN8	Endothelial protein C receptor OS=Homo sapiens GN=PROCR PE=1 SV=1 - [EPCR_HUMAN]	20.59	1	4	4	8	24.10	23.76	23.49	23.17			3	1	24.26	23.83	23.55	22.93			23.88	22.93	NA	NA	-0.952
P62310	U6 snRNA-associated Sm-like protein LSm3 OS=Homo sapiens GN=LSM3 PE=1 SV=2 - [LSM3_HUMAN]	20.59	1	2	2	5				23.56	23.56	23.60	0	3				23.32	23.78	23.34		23.48	0.000	0.000	
P28482	Mitogen-activated protein kinase 1 OS=Homo sapiens GN=MAPK1 PE=1 SV=3 - [MK01_HUMAN]	20.56	13	5	5	7	22.39	22.34		23.15		22.67	2	2	22.56	22.41		22.90		22.41	22.48	22.66	0.564	0.186	0.175
P61218	DNA-directed RNA polymerases I, II, and III subunit RPABC2 OS=Homo sapiens GN=POLR2F PE=1 SV=1 - [RPAB2_HUMAN]	20.47	1	1	1	1					22.21		0	1					22.43			22.43	NA	NA	
P09661	U2 small nuclear ribonucleoprotein A' OS=Homo sapiens GN=SNRPA1 PE=1 SV=2 - [RU2A_HUMAN]	20.39	1	4	4	7				23.90	23.10	23.05	0	3				23.66	23.32	22.79		23.25	0.000	0.000	
Q92879	CUGBP Elav-like family member 1 OS=Homo sapiens GN=CELF1 PE=1 SV=2 - [CELF1_HUMAN]	20.37	2	8	8	13					23.63	23.07	0	2					23.85	22.81		23.33	NA	NA	
O75494	Serine/arginine-rich splicing factor 10 OS=Homo sapiens GN=SRSF10 PE=1 SV=1 - [SRS10_HUMAN]	20.23	2	4	4	8				24.06	24.31	24.13	0	3				23.81	24.53	23.87		24.07	0.000	0.000	
P53365	Arfaptin-2 OS=Homo sapiens GN=ARFIP2 PE=1 SV=1 - [ARFP2_HUMAN]	20.23	1	4	4	9				23.05	21.84	23.05	0	3				22.81	22.06	22.79		22.55	0.000	0.000	
Q16864	V-type proton ATPase subunit F OS=Homo sapiens GN=ATP6V1F PE=1 SV=2 - [VATF_HUMAN]	20.17	1	1	1	3				21.69	21.98	21.91	0	3				21.44	22.19	21.65		21.76	0.000	0.000	
Q9H0B6	Kinesin light chain 2 OS=Homo sapiens GN=KLC2 PE=1 SV=1 - [KLC2_HUMAN]	20.10	1	5	9	23	22.28			23.75	26.79	23.84	1	3	22.44			23.50	27.00	23.58	22.44	24.70	NA	NA	2.259
Q99627	COP9 signalosome complex subunit 8 OS=Homo sapiens GN=COPS8 PE=1 SV=1 - [CSN8_HUMAN]	20.10	1	3	3	9	22.47			23.43	24.03	23.51	1	3	22.63			23.18	24.24	23.25	22.63	23.56	NA	NA	0.927
Q9NZ09	Ubiquitin-associated protein 1 OS=Homo sapiens GN=UBAP1 PE=1 SV=1 - [UBAP1_HUMAN]	19.92	1	6	6	13	23.73	23.60	23.80			22.98	3	1	23.89	23.67	23.86			22.72	23.81	22.72	NA	NA	-1.084
Q9BZE4	Nucleolar GTP-binding protein 1 OS=Homo sapiens GN=GTPBP4 PE=1 SV=3 - [NOG1_HUMAN]	19.87	1	10	10	27				24.41	24.49	24.14	0	3				24.17	24.71	23.88		24.25	0.000	0.000	
Q9NRG0	Chromatin accessibility complex protein 1 OS=Homo sapiens GN=CHRAC1 PE=1 SV=1 - [CHRC1_HUMAN]	19.85	1	1	1	1				22.25			0	1				22.00				22.00	NA	NA	
Q8TDN6	Ribosome biogenesis protein BRX1 homolog OS=Homo sapiens GN=BRIX1 PE=1 SV=2 - [BRX1_HUMAN]	19.83	1	5	5	9				22.88	23.89	22.59	0	3				22.63	24.10	22.33		23.02	0.000	0.000	
Q12906	Interleukin enhancer-binding factor 3 OS=Homo sapiens GN=ILF3 PE=1 SV=3 - [ILF3_HUMAN]	19.80	2	12	12	35	21.43			24.75	24.76	24.50	1	3	21.60			24.50	24.98	24.24	21.60	24.58	NA	NA	2.980
P07919	Cytochrome b-c1 complex subunit 6, mitochondrial OS=Homo sapiens GN=UQCRH PE=1 SV=2 - [QCR6_HUMAN]	19.78	1	1	1	3		23.58			24.71		1	1		23.65			24.93		23.65	24.93	NA	NA	1.280
Q96CT7	Coiled-coil domain-containing protein 124 OS=Homo sapiens GN=CCDC124 PE=1 SV=1 - [CC124_HUMAN]	19.73	1	5	5	12	21.67	22.77	22.61			21.76	3	1	21.84	22.84	22.67			21.50	22.45	21.50	NA	NA	-0.947
P62891	60S ribosomal protein L39 OS=Homo sapiens GN=RPL39 PE=1 SV=2 - [RL39_HUMAN]	19.61	2	1	1	1				23.71			0	1				23.46				23.46	NA	NA	
Q9BQ67	Glutamate-rich WD repeat-containing protein 1 OS=Homo sapiens GN=GRWD1 PE=1 SV=1 - [GRWD1_HUMAN]	19.51	13	5	6	12	22.13			23.88	23.30	23.54	1	3	22.29			23.64	23.52	23.28	22.29	23.48	NA	NA	1.182
Q5RKV6	Exosome complex component MTR3 OS=Homo sapiens GN=EXOSC6 PE=1 SV=1 - [EXOS6_HUMAN]	19.49	1	3	3	5				23.37	22.12	21.88	0	3				23.13	22.33	21.62		22.36	0.000	0.000	
Q96A33	Coiled-coil domain-containing protein 47 OS=Homo sapiens GN=CCDC47 PE=1 SV=1 - [CCD47_HUMAN]	19.46	1	7	7	14			20.87	22.13	23.00	23.48	1	3			20.93	21.89	23.22	23.22	20.93	22.78	NA	NA	1.844
O95249	Golgi SNAP receptor complex member 1 OS=Homo sapiens GN=GOSR1 PE=1 SV=1 - [GOSR1_HUMAN]	19.20	1	4	4	10		21.26		23.23	22.40	22.46	1	3		21.33		22.98	22.62	22.20	21.33	22.60	NA	NA	1.271
P41208	Centrin-2 OS=Homo sapiens GN=CETN2 PE=1 SV=1 - [CETN2_HUMAN]	19.19	1	1	1	1						23.21	0	1						22.94		22.94	NA	NA	
P48060	Glioma pathogenesis-related protein 1 OS=Homo sapiens GN=GLIPR1 PE=1 SV=3 - [GLIP1_HUMAN]	19.17	1	3	3	7	23.28	23.78	23.66				3	0	23.45	23.85	23.72				23.67		0.000	0.000	
P49589	Cysteine--tRNA ligase, cytoplasmic OS=Homo sapiens GN=CARS PE=1 SV=3 - [SYCC_HUMAN]	19.12	1	11	11	27		22.58		24.10	23.07	24.16	1	3		22.65		23.85	23.28	23.90	22.65	23.68	NA	NA	1.025
P98179	RNA-binding protein 3 OS=Homo sapiens GN=RBM3 PE=1 SV=1 - [RBM3_HUMAN]	19.11	1	2	2	4	24.18	22.10	23.16				3	0	24.34	22.17	23.21				23.24		0.000	0.000	
Q9BTV4	Transmembrane protein 43 OS=Homo sapiens GN=TMEM43 PE=1 SV=1 - [TMM43_HUMAN]	19.00	1	4	4	7					22.70	24.42	0	2					22.91	24.16		23.54	NA	NA	
Q9UKY7	Protein CDV3 homolog OS=Homo sapiens GN=CDV3 PE=1 SV=1 - [CDV3_HUMAN]	18.99	1	1	1	5		22.94		23.45	22.67	23.40	1	3		23.01		23.20	22.88	23.14	23.01	23.08	NA	NA	0.067
Q9BV57	1,2-dihydroxy-3-keto-5-methylthiopentene dioxygenase OS=Homo sapiens GN=ADI1 PE=1 SV=1 - [MTND_HUMAN]	18.99	1	3	3	7				22.83	22.95	22.66	0	3				22.59	23.17	22.40		22.72	0.000	0.000	
P33552	Cyclin-dependent kinases regulatory subunit 2 OS=Homo sapiens GN=CKS2 PE=1 SV=1 - [CKS2_HUMAN]	18.99	1	2	2	3		21.39	22.64				2	0		21.46	22.70				22.08		NA	NA	
CON_007151881	SWISS-PROT:Q3MHN2 Bos taurus Complement component C9 precursor	18.98	1	6	6	13	23.37			23.03	22.44	23.28	1	3	23.53			22.78	22.66	23.02	23.53	22.82	NA	NA	-0.710
Q8ND90	Paraneoplastic antigen Ma1 OS=Homo sapiens GN=PNMA1 PE=1 SV=2 - [PNMA1_HUMAN]	18.98	1	4	4	7	22.56	22.09	22.85				3	0	22.72	22.16	22.91				22.60		0.000	0.000	
Q3MHD2	Protein LSM12 homolog OS=Homo sapiens GN=LSM12 PE=1 SV=2 - [LSM12_HUMAN]	18.97	1	2	2	5				23.57	23.51	23.51	0	3				23.32	23.73	23.25		23.43	0.000	0.000	
Q7Z7H5	Transmembrane emp24 domain-containing protein 4 OS=Homo sapiens GN=TMED4 PE=1 SV=1 - [TMED4_HUMAN]	18.94	1	3	4	7	21.88			23.23	21.27		1	2	22.04			22.99	21.49		22.04	22.24	NA	NA	0.198
Q9Y3A4	Ribosomal RNA-processing protein 7 homolog A OS=Homo sapiens GN=RRP7A PE=1 SV=2 - [RRP7A_HUMAN]	18.93	1	2	2	2					22.27		0	1					22.49			22.49	NA	NA	
Q9Y605	MORF4 family-associated protein 1 OS=Homo sapiens GN=MRFAP1 PE=1 SV=1 - [MOFA1_HUMAN]	18.90	1	1	1	1					21.19		0	1					21.41			21.41	NA	NA	
P09234	U1 small nuclear ribonucleoprotein C OS=Homo sapiens GN=SNRPC PE=1 SV=1 - [RU1C_HUMAN]	18.87	1	2	2	4			23.50		24.45	25.03	1	2			23.56		24.66	24.76	23.56	24.71	NA	NA	1.158
O15160	DNA-directed RNA polymerases I and III subunit RPAC1 OS=Homo sapiens GN=POLR1C PE=1 SV=1 - [RPAC1_HUMAN]	18.79	1	4	4	10				24.08	23.97	23.13	0	3				23.83	24.19	22.87		23.63	0.000	0.000	
Q13685	Angio-associated migratory cell protein OS=Homo sapiens GN=AAMP PE=1 SV=2 - [AAMP_HUMAN]	18.66	1	6	6	14				23.29	23.05	23.55	0	3				23.04	23.26	23.29		23.20	0.000	0.000	
Q16539	Mitogen-activated protein kinase 14 OS=Homo sapiens GN=MAPK14 PE=1 SV=3 - [MK14_HUMAN]	18.61	11	4	4	7		21.73	22.06	22.23	22.39		2	2		21.80	22.12	21.99	22.60		21.96	22.29	0.439	0.152	0.334
Q9UHG3	Prenylcysteine oxidase 1 OS=Homo sapiens GN=PCYOX1 PE=1 SV=3 - [PCYOX_HUMAN]	18.61	1	6	6	10					23.60	23.18	0	2					23.81	22.92		23.37	NA	NA	
Q14011	Cold-inducible RNA-binding protein OS=Homo sapiens GN=CIRBP PE=1 SV=1 - [CIRBP_HUMAN]	18.60	1	2	2	2					21.63	21.81	0	2					21.85	21.55		21.70	NA	NA	
A6ZKI3	Protein FAM127A OS=Homo sapiens GN=FAM127A PE=1 SV=1 - [F127A_HUMAN]	18.58	2	1	2	4	23.18	22.73					2	0	23.35	22.80					23.07		NA	NA	
Q9UHA4	Ragulator complex protein LAMTOR3 OS=Homo sapiens GN=LAMTOR3 PE=1 SV=1 - [LTOR3_HUMAN]	18.55	1	1	1	1				24.47			0	1				24.22				24.22	NA	NA	
Q5T1J5	Putative coiled-coil-helix-coiled-coil-helix domain-containing protein CHCHD2P9, mitochondrial OS=Homo sapiens GN=CHCHD2P9 PE=5 SV=1 - [CHCH9_HUMAN]	18.54	2	1	1	3				22.89	22.66	23.02	0	3				22.65	22.88	22.76		22.76	0.000	0.000	
P35249	Replication factor C subunit 4 OS=Homo sapiens GN=RFC4 PE=1 SV=2 - [RFC4_HUMAN]	18.46	1	6	6	10		21.64		22.81	22.54	23.47	1	3		21.71		22.56	22.76	23.20	21.71	22.84	NA	NA	1.130
P09012	U1 small nuclear ribonucleoprotein A OS=Homo sapiens GN=SNRPA PE=1 SV=3 - [SNRPA_HUMAN]	18.44	1	3	5	11				24.74	23.47	23.51	0	3				24.49	23.69	23.25		23.81	0.000	0.000	
Q9UGM3	Deleted in malignant brain tumors 1 protein OS=Homo sapiens GN=DMBT1 PE=1 SV=2 - [DMBT1_HUMAN]	18.40	1	4	4	9	22.49			23.35	22.65	25.07	1	3	22.65			23.11	22.86	24.81	22.65	23.59	NA	NA	0.944
Q7L0Y3	Mitochondrial ribonuclease P protein 1 OS=Homo sapiens GN=TRMT10C PE=1 SV=2 - [MRRP1_HUMAN]	18.36	1	6	6	10				22.84	22.69	22.77	0	3				22.59	22.91	22.51		22.67	0.000	0.000	
Q10589	Bone marrow stromal antigen 2 OS=Homo sapiens GN=BST2 PE=1 SV=1 - [BST2_HUMAN]	18.33	1	3	3	10				25.14	23.95	24.80	0	3				24.89	24.16	24.54		24.53	0.000	0.000	
Q8N4V1	Membrane magnesium transporter 1 OS=Homo sapiens GN=MMGT1 PE=1 SV=1 - [MMGT1_HUMAN]	18.32	1	1	1	4					22.93	23.66	0	2					23.15	23.40		23.27	NA	NA	
O15305	Phosphomannomutase 2 OS=Homo sapiens GN=PMM2 PE=1 SV=1 - [PMM2_HUMAN]	18.29	2	3	3	7				22.69	21.42	22.68	0	3				22.44	21.64	22.42		22.16	0.000	0.000	
Q99805	Transmembrane 9 superfamily member 2 OS=Homo sapiens GN=TM9SF2 PE=1 SV=1 - [TM9S2_HUMAN]	18.25	1	6	6	23	23.08	23.27		23.81		24.53	2	2	23.25	23.34		23.56		24.26	23.29	23.91	0.222	0.090	0.620
Q96EP5	DAZ-associated protein 1 OS=Homo sapiens GN=DAZAP1 PE=1 SV=1 - [DAZP1_HUMAN]	18.18	1	4	4	8				23.03	23.29	23.80	0	3				22.78	23.51	23.54		23.28	0.000	0.000	
O15269	Serine palmitoyltransferase 1 OS=Homo sapiens GN=SPTLC1 PE=1 SV=1 - [SPTC1_HUMAN]	18.18	1	5	5	8		21.34		22.43	20.86	22.38	1	3		21.41		22.19	21.08	22.12	21.41	21.79	NA	NA	0.381
O43447	Peptidyl-prolyl cis-trans isomerase H OS=Homo sapiens GN=PPIH PE=1 SV=1 - [PPIH_HUMAN]	18.08	1	3	3	5					22.59	23.16	0	2					22.80	22.90		22.85	NA	NA	
Q15435	Protein phosphatase 1 regulatory subunit 7 OS=Homo sapiens GN=PPP1R7 PE=1 SV=1 - [PP1R7_HUMAN]	18.06	1	5	5	15	21.82			23.62	23.69	23.61	1	3	21.98			23.37	23.91	23.35	21.98	23.54	NA	NA	1.563
Q9BT78	COP9 signalosome complex subunit 4 OS=Homo sapiens GN=COPS4 PE=1 SV=1 - [CSN4_HUMAN]	17.98	1	5	5	11	21.06			21.90	22.63	22.91	1	3	21.22			21.65	22.85	22.65	21.22	22.38	NA	NA	1.161
P48507	Glutamate--cysteine ligase regulatory subunit OS=Homo sapiens GN=GCLM PE=1 SV=1 - [GSH0_HUMAN]	17.88	1	4	4	8				22.61	22.23	22.54	0	3				22.36	22.45	22.28		22.36	0.000	0.000	
P51570	Galactokinase OS=Homo sapiens GN=GALK1 PE=1 SV=1 - [GALK1_HUMAN]	17.86	1	3	3	8			23.16	23.77	25.29	24.13	1	3			23.22	23.52	25.50	23.87	23.22	24.30	NA	NA	1.081
Q7Z3B1	Neuronal growth regulator 1 OS=Homo sapiens GN=NEGR1 PE=1 SV=3 - [NEGR1_HUMAN]	17.80	1	3	3	10	24.60	24.54	24.44				3	0	24.76	24.61	24.50				24.62		0.000	0.000	
Q9UNH7	Sorting nexin-6 OS=Homo sapiens GN=SNX6 PE=1 SV=1 - [SNX6_HUMAN]	17.73	2	6	6	16	21.97			23.04	23.35	24.03	1	3	22.14			22.79	23.57	23.77	22.14	23.38	NA	NA	1.241
Q15833	Syntaxin-binding protein 2 OS=Homo sapiens GN=STXBP2 PE=1 SV=2 - [STXB2_HUMAN]	17.71	1	7	7	10	23.44	23.35			22.54	24.11	2	2	23.60	23.42			22.76	23.85	23.51	23.30	0.747	0.230	-0.206
O95777	U6 snRNA-associated Sm-like protein LSm8 OS=Homo sapiens GN=LSM8 PE=1 SV=3 - [LSM8_HUMAN]	17.71	1	2	2	3					21.32	21.97	0	2					21.54	21.71		21.62	NA	NA	
Q9ULA0	Aspartyl aminopeptidase OS=Homo sapiens GN=DNPEP PE=1 SV=1 - [DNPEP_HUMAN]	17.68	1	4	4	12				23.22	22.88	23.38	0	3				22.98	23.09	23.12		23.06	0.000	0.000	
P62877	E3 ubiquitin-protein ligase RBX1 OS=Homo sapiens GN=RBX1 PE=1 SV=1 - [RBX1_HUMAN]	17.59	1	1	1	2		21.71	22.13				2	0		21.78	22.18				21.98		NA	NA	
Q96J84	Kin of IRRE-like protein 1 OS=Homo sapiens GN=KIRREL PE=1 SV=2 - [KIRR1_HUMAN]	17.57	1	8	8	21	23.30	23.30	23.64			21.88	3	1	23.46	23.37	23.69			21.62	23.51	21.62	NA	NA	-1.887
Q9Y237	Peptidyl-prolyl cis-trans isomerase NIMA-interacting 4 OS=Homo sapiens GN=PIN4 PE=1 SV=1 - [PIN4_HUMAN]	17.56	1	2	2	4				23.14	21.95	23.36	0	3				22.89	22.17	23.10		22.72	0.000	0.000	
Q96C01	Protein FAM136A OS=Homo sapiens GN=FAM136A PE=1 SV=1 - [F136A_HUMAN]	17.39	1	2	2	3					21.77	20.98	0	2					21.99	20.71		21.35	NA	NA	
Q15005	Signal peptidase complex subunit 2 OS=Homo sapiens GN=SPCS2 PE=1 SV=3 - [SPCS2_HUMAN]	17.26	1	3	3	9			20.57		17.29	21.13	1	2			20.63		17.51	20.87	20.63	19.19	NA	NA	-1.441
Q9GZN8	UPF0687 protein C20orf27 OS=Homo sapiens GN=C20orf27 PE=1 SV=3 - [CT027_HUMAN]	17.24	1	2	2	2		21.27		23.62			1	1		21.34		23.37			21.34	23.37	NA	NA	2.033
P16422	Epithelial cell adhesion molecule OS=Homo sapiens GN=EPCAM PE=1 SV=2 - [EPCAM_HUMAN]	17.20	1	3	3	6		19.57	21.40	22.53		24.06	2	2		19.64	21.45	22.28		23.80	20.55	23.04	0.170	0.074	2.493
P15559	NAD(P)H dehydrogenase [quinone] 1 OS=Homo sapiens GN=NQO1 PE=1 SV=1 - [NQO1_HUMAN]	17.15	1	4	4	4	23.71		21.78				2	0	23.87		21.84				22.85		NA	NA	
P80303	Nucleobindin-2 OS=Homo sapiens GN=NUCB2 PE=1 SV=2 - [NUCB2_HUMAN]	17.14	1	5	5	9				22.65	22.58	23.25	0	3				22.40	22.79	22.99		22.73	0.000	0.000	
O76071	Probable cytosolic iron-sulfur protein assembly protein CIAO1 OS=Homo sapiens GN=CIAO1 PE=1 SV=1 - [CIAO1_HUMAN]	17.11	1	4	4	12	22.17			22.94	23.13	23.68	1	3	22.33			22.69	23.34	23.42	22.33	23.15	NA	NA	0.820
Q8N884	Cyclic GMP-AMP synthase OS=Homo sapiens GN=MB21D1 PE=1 SV=2 - [CGAS_HUMAN]	17.05	1	5	5	14				23.76	23.21		0	2				23.51	23.42			23.47	NA	NA	
O75489	NADH dehydrogenase [ubiquinone] iron-sulfur protein 3, mitochondrial OS=Homo sapiens GN=NDUFS3 PE=1 SV=1 - [NDUS3_HUMAN]	17.05	1	3	3	4					22.43		0	1					22.64			22.64	NA	NA	
Q9Y2B0	Protein canopy homolog 2 OS=Homo sapiens GN=CNPY2 PE=1 SV=1 - [CNPY2_HUMAN]	17.03	1	2	2	3					23.84	23.77	0	2					24.06	23.51		23.79	NA	NA	
O00422	Histone deacetylase complex subunit SAP18 OS=Homo sapiens GN=SAP18 PE=1 SV=1 - [SAP18_HUMAN]	16.99	1	2	2	3				23.14	22.09		0	2				22.90	22.31			22.60	NA	NA	
P62861	40S ribosomal protein S30 OS=Homo sapiens GN=FAU PE=1 SV=1 - [RS30_HUMAN]	16.95	1	1	1	1				26.35			0	1				26.11				26.11	NA	NA	
P62633	Cellular nucleic acid-binding protein OS=Homo sapiens GN=CNBP PE=1 SV=1 - [CNBP_HUMAN]	16.95	1	2	2	5		23.03			22.76	22.87	1	2		23.10			22.98	22.60	23.10	22.79	NA	NA	-0.311
Q5XKP0	MICOS complex subunit MIC13 OS=Homo sapiens GN=MIC13 PE=1 SV=1 - [MIC13_HUMAN]	16.95	1	1	1	1					21.53		0	1					21.74			21.74	NA	NA	

P84101	Small EDRK-rich factor 2 OS=Homo sapiens GN=SERF2 PE=1 SV=1 - [SERF2_HUMAN]	16.95	1	1	1	2			22.06				1	0			22.12				22.12		NA	NA	
Q9BUP3	Oxidoreductase HTATIP2 OS=Homo sapiens GN=HTATIP2 PE=1 SV=2 - [HTAI2_HUMAN]	16.94	1	3	3	4		22.95	22.72		21.28		2	1		23.02	22.78		21.49		22.90	21.49	NA	NA	-1.406
Q8WXX5	DnaJ homolog subfamily C member 9 OS=Homo sapiens GN=DNAJC9 PE=1 SV=1 - [DNJC9_HUMAN]	16.92	1	2	2	3					21.08	22.18	0	2					21.30	21.92		21.61	NA	NA	
Q7KZI7	Serine/threonine-protein kinase MARK2 OS=Homo sapiens GN=MARK2 PE=1 SV=2 - [MARK2_HUMAN]	16.88	2	7	10	15	23.30	23.11	22.28				3	0	23.46	23.18	22.34				22.99		0.000	0.000	
Q9BWD3	Protein FAM127B OS=Homo sapiens GN=FAM127B PE=1 SV=3 - [F127B_HUMAN]	16.81	1	1	2	3	22.85	22.30					2	0	23.01	22.37					22.69		NA	NA	
Q8NFZ5	TNFAIP3-interacting protein 2 OS=Homo sapiens GN=TNIP2 PE=1 SV=1 - [TNIP2_HUMAN]	16.78	1	5	5	7	22.07		21.08				2	0	22.23		21.14				21.69		NA	NA	
Q9NVP1	ATP-dependent RNA helicase DDX18 OS=Homo sapiens GN=DDX18 PE=1 SV=2 - [DDX18_HUMAN]	16.72	1	8	8	14				23.35	23.51	22.43	0	3				23.11	23.72	22.17		23.00	0.000	0.000	
O95793	Double-stranded RNA-binding protein Staufen homolog 1 OS=Homo sapiens GN=STAU1 PE=1 SV=2 - [STAU1_HUMAN]	16.64	2	8	8	16				22.97	23.31	23.15	0	3				22.73	23.53	22.89		23.05	0.000	0.000	
Q8IUW5	RELT-like protein 1 OS=Homo sapiens GN=RELL1 PE=1 SV=1 - [RELL1_HUMAN]	16.61	1	3	3	4	22.00		21.60	22.15			2	1	22.16		21.66	21.90			21.91	21.90	NA	NA	-0.008
Q9Y5B9	FACT complex subunit SPT16 OS=Homo sapiens GN=SUPT16H PE=1 SV=1 - [SP16H_HUMAN]	16.52	1	14	14	27	20.15			23.77	24.04	26.24	1	3	20.31			23.53	24.26	25.98	20.31	24.59	NA	NA	4.275
Q969E4	Transcription elongation factor A protein-like 3 OS=Homo sapiens GN=TCEAL3 PE=1 SV=1 - [TCAL3_HUMAN]	16.50	3	2	2	6				21.09	20.87	21.41	0	3				20.85	21.08	21.15		21.03	0.000	0.000	
Q8WWB7	Glycosylated lysosomal membrane protein OS=Homo sapiens GN=GLMP PE=1 SV=1 - [GLMP_HUMAN]	16.50	1	3	3	8				24.24		22.31	0	2				24.00		22.05		23.03	NA	NA	
O75915	PRA1 family protein 3 OS=Homo sapiens GN=ARL6IP5 PE=1 SV=1 - [PRAF3_HUMAN]	16.49	1	2	2	4		23.82	23.80			22.84	2	1		23.89	23.86			22.58	23.87	22.58	NA	NA	-1.292
O60499	Syntaxin-10 OS=Homo sapiens GN=STX10 PE=1 SV=1 - [STX10_HUMAN]	16.47	1	2	2	2				24.09			0	1				23.84				23.84	NA	NA	
Q9BPX5	Actin-related protein 2/3 complex subunit 5-like protein OS=Homo sapiens GN=ARPC5L PE=1 SV=1 - [ARP5L_HUMAN]	16.34	1	3	3	5				22.42	23.25	22.41	0	3				22.18	23.46	22.15		22.60	0.000	0.000	
Q9UHX1	Poly(U)-binding-splicing factor PUF60 OS=Homo sapiens GN=PUF60 PE=1 SV=1 - [PUF60_HUMAN]	16.28	1	6	6	17				24.34	23.94	24.00	0	3				24.09	24.15	23.74		23.99	0.000	0.000	
P36776	Lon protease homolog, mitochondrial OS=Homo sapiens GN=LONP1 PE=1 SV=2 - [LONM_HUMAN]	16.27	1	8	8	16					24.01	23.81	0	2					24.23	23.55		23.89	NA	NA	
Q92743	Serine protease HTRA1 OS=Homo sapiens GN=HTRA1 PE=1 SV=1 - [HTRA1_HUMAN]	16.25	1	5	5	7	23.14		21.64				2	0	23.30		21.70				22.50		NA	NA	
Q969H8	Myeloid-derived growth factor OS=Homo sapiens GN=MYDGF PE=1 SV=1 - [MYDGF_HUMAN]	16.18	1	3	3	4					24.31	24.54	0	2					24.53	24.28		24.40	NA	NA	
P02656	Apolipoprotein C-III OS=Homo sapiens GN=APOC3 PE=1 SV=1 - [APOC3_HUMAN]	16.16	1	1	1	2	24.07				24.75		1	1	24.23				24.96		24.23	24.96	NA	NA	0.730
O14545	TRAF-type zinc finger domain-containing protein 1 OS=Homo sapiens GN=TRAFD1 PE=1 SV=1 - [TRAD1_HUMAN]	16.15	1	7	7	15		23.29	23.37		16.84		2	1		23.36	23.43		17.06		23.40	17.06	NA	NA	-6.338
Q96B97	SH3 domain-containing kinase-binding protein 1 OS=Homo sapiens GN=SH3KBP1 PE=1 SV=2 - [SH3K1_HUMAN]	16.09	1	8	8	13	22.01	22.59	21.79				3	0	22.17	22.66	21.85				22.22		0.000	0.000	
O60711	Leupaxin OS=Homo sapiens GN=LPXN PE=1 SV=1 - [LPXN_HUMAN]	16.06	1	4	4	13	21.50			24.09	23.86	23.83	1	3	21.66			23.84	24.08	23.57	21.66	23.83	NA	NA	2.169
P28799	Granulins OS=Homo sapiens GN=GRN PE=1 SV=2 - [GRN_HUMAN]	16.02	1	7	7	17		23.30		23.57	23.95	23.77	1	3		23.37		23.33	24.16	23.51	23.37	23.67	NA	NA	0.291
Q9BYD6	39S ribosomal protein L1, mitochondrial OS=Homo sapiens GN=MRPL1 PE=1 SV=2 - [RM01_HUMAN]	16.00	1	3	3	6				22.30	22.46	23.42	0	3				22.06	22.68	23.16		22.63	0.000	0.000	
Q13951	Core-binding factor subunit beta OS=Homo sapiens GN=CBFB PE=1 SV=2 - [PEBB_HUMAN]	15.93	1	1	1	3	23.35				23.29		1	1	23.51				23.51		23.51	23.51	NA	NA	-0.002
Q9UBE0	SUMO-activating enzyme subunit 1 OS=Homo sapiens GN=SAE1 PE=1 SV=1 - [SAE1_HUMAN]	15.90	1	3	3	6		19.90			22.75	22.10	1	2		19.97			22.96	21.84	19.97	22.40	NA	NA	2.433
Q9Y295	Developmentally-regulated GTP-binding protein 1 OS=Homo sapiens GN=DRG1 PE=1 SV=1 - [DRG1_HUMAN]	15.80	1	4	4	6	21.34			22.76		22.46	1	2	21.50			22.51		22.20	21.50	22.36	NA	NA	0.853
Q13547	Histone deacetylase 1 OS=Homo sapiens GN=HDAC1 PE=1 SV=1 - [HDAC1_HUMAN]	15.77	1	2	5	13	22.49			23.13	23.66	23.84	1	3	22.65			22.88	23.87	23.58	22.65	23.45	NA	NA	0.798
P56381	ATP synthase subunit epsilon, mitochondrial OS=Homo sapiens GN=ATP5E PE=1 SV=2 - [ATP5E_HUMAN]	15.69	2	1	1	1					21.96		0	1					22.18			22.18	NA	NA	
O95394	Phosphoacetylglucosamine mutase OS=Homo sapiens GN=PGM3 PE=1 SV=1 - [AGM1_HUMAN]	15.68	1	4	4	6	21.06			21.98	21.02	22.41	1	3	21.22			21.73	21.24	22.15	21.22	21.71	NA	NA	0.483
P00167	Cytochrome b5 OS=Homo sapiens GN=CYB5A PE=1 SV=2 - [CYB5_HUMAN]	15.67	1	1	1	4				22.65	22.80	23.52	0	3				22.41	23.02	23.26		22.90	0.000	0.000	
Q9BUJ2	Heterogeneous nuclear ribonucleoprotein U-like protein 1 OS=Homo sapiens GN=HNRNPUL1 PE=1 SV=2 - [HNRL1_HUMAN]	15.65	1	8	9	20	21.98			24.26	24.50	25.29	1	3	22.14			24.02	24.71	25.03	22.14	24.59	NA	NA	2.445
O75880	Protein SCO1 homolog, mitochondrial OS=Homo sapiens GN=SCO1 PE=1 SV=1 - [SCO1_HUMAN]	15.61	1	2	2	4					22.04	22.82	0	2					22.25	22.56		22.41	NA	NA	
Q7Z4F1	Low-density lipoprotein receptor-related protein 10 OS=Homo sapiens GN=LRP10 PE=1 SV=2 - [LRP10_HUMAN]	15.57	1	6	6	15		22.28		25.02	22.68	23.41	1	3		22.35		24.77	22.90	23.15	22.35	23.61	NA	NA	1.256
Q96HS1	Serine/threonine-protein phosphatase PGAM5, mitochondrial OS=Homo sapiens GN=PGAM5 PE=1 SV=2 - [PGAM5_HUMAN]	15.57	1	5	5	6					22.02	22.88	0	2					22.24	22.62		22.43	NA	NA	
P62072	Mitochondrial import inner membrane translocase subunit Tim10 OS=Homo sapiens GN=TIMM10 PE=1 SV=1 - [TIM10_HUMAN]	15.56	1	1	1	1						22.69	0	1						22.43		22.43	NA	NA	
Q9H2H8	Peptidyl-prolyl cis-trans isomerase-like 3 OS=Homo sapiens GN=PPIL3 PE=1 SV=1 - [PPIL3_HUMAN]	15.53	1	2	2	4				22.15	22.77	23.20	0	3				21.90	22.99	22.94		22.61	0.000	0.000	
Q13442	28 kDa heat- and acid-stable phosphoprotein OS=Homo sapiens GN=PDAP1 PE=1 SV=1 - [HAP28_HUMAN]	15.47	1	2	2	4			20.35	21.67	21.15	21.88	1	3			20.41	21.43	21.37	21.62	20.41	21.47	NA	NA	1.066
Q96RF0	Sorting nexin-18 OS=Homo sapiens GN=SNX18 PE=1 SV=2 - [SNX18_HUMAN]	15.45	1	6	6	10	21.79	22.78	23.50			21.47	3	1	21.96	22.85	23.56			21.21	22.79	21.21	NA	NA	-1.580
Q9BVG4	Protein PBDC1 OS=Homo sapiens GN=PBDC1 PE=1 SV=1 - [PBDC1_HUMAN]	15.45	1	3	3	8				23.11	22.98	23.25	0	3				22.86	23.20	22.99		23.02	0.000	0.000	
P30533	Alpha-2-macroglobulin receptor-associated protein OS=Homo sapiens GN=LRPAP1 PE=1 SV=1 - [AMRP_HUMAN]	15.41	1	4	4	6					22.50	23.29	0	2					22.72	23.03		22.87	NA	NA	
O00743	Serine/threonine-protein phosphatase 6 catalytic subunit OS=Homo sapiens GN=PPP6C PE=1 SV=1 - [PPP6_HUMAN]	15.41	1	4	4	4					22.77	22.47	0	2					22.98	22.21		22.60	NA	NA	
Q14657	EKC/KEOPS complex subunit LAGE3 OS=Homo sapiens GN=LAGE3 PE=1 SV=2 - [LAGE3_HUMAN]	15.38	1	1	1	4					16.89		0	1					17.11			17.11	NA	NA	
Q14696	LDLR chaperone MESD OS=Homo sapiens GN=MESDC2 PE=1 SV=2 - [MESD_HUMAN]	15.38	1	3	3	6			21.23		22.21	22.84	1	2			21.29		22.42	22.58	21.29	22.50	NA	NA	1.208
Q9Y2R5	28S ribosomal protein S17, mitochondrial OS=Homo sapiens GN=MRPS17 PE=1 SV=1 - [RT17_HUMAN]	15.38	1	1	1	1					22.41		0	1					22.63			22.63	NA	NA	
Q9NQ29	Putative RNA-binding protein Luc7-like 1 OS=Homo sapiens GN=LUC7L PE=1 SV=1 - [LUC7L_HUMAN]	15.36	1	3	5	15				23.61	23.64	23.43	0	3				23.36	23.86	23.17		23.46	0.000	0.000	
Q7L5N1	COP9 signalosome complex subunit 6 OS=Homo sapiens GN=COPS6 PE=1 SV=1 - [CSN6_HUMAN]	15.29	1	3	3	3	21.70					22.92	1	1	21.86					22.66	21.86	22.66	NA	NA	0.805
O75718	Cartilage-associated protein OS=Homo sapiens GN=CRTAP PE=1 SV=1 - [CRTAP_HUMAN]	15.21	1	6	6	10					23.34	23.24	0	2					23.55	22.98		23.26	NA	NA	
P09874	Poly [ADP-ribose] polymerase 1 OS=Homo sapiens GN=PARP1 PE=1 SV=4 - [PARP1_HUMAN]	15.19	1	11	11	24				23.65	23.78	23.11	0	3				23.41	24.00	22.85		23.42	0.000	0.000	
Q9Y3B8	Oligoribonuclease, mitochondrial OS=Homo sapiens GN=REXO2 PE=1 SV=3 - [ORN_HUMAN]	15.19	1	3	3	10			22.57	23.43	23.71	23.52	1	3			22.63	23.18	23.93	23.26	22.63	23.46	NA	NA	0.829
Q9Y478	5'-AMP-activated protein kinase subunit beta-1 OS=Homo sapiens GN=PRKAB1 PE=1 SV=4 - [AAKB1_HUMAN]	15.19	1	2	2	2					21.15	23.54	0	2					21.37	23.28		22.32	NA	NA	
Q9Y3C1	Nucleolar protein 16 OS=Homo sapiens GN=NOP16 PE=1 SV=2 - [NOP16_HUMAN]	15.17	1	2	2	4					21.90	22.41	0	2					22.12	22.15		22.13	NA	NA	
Q92688	Acidic leucine-rich nuclear phosphoprotein 32 family member B OS=Homo sapiens GN=ANP32B PE=1 SV=1 - [AN32B_HUMAN]	15.14	1	2	4	12		21.82		23.59	23.18	23.99	1	3		21.89		23.34	23.39	23.73	21.89	23.49	NA	NA	1.602
P08579	U2 small nuclear ribonucleoprotein B'' OS=Homo sapiens GN=SNRPB2 PE=1 SV=1 - [RU2B_HUMAN]	15.11	1	2	4	7			20.35		22.76	22.62	1	2			20.41		22.97	22.36	20.41	22.67	NA	NA	2.255
Q9NX14	NADH dehydrogenase [ubiquinone] 1 beta subcomplex subunit 11, mitochondrial OS=Homo sapiens GN=NDUFB11 PE=1 SV=1 - [NDUBB_HUMAN]	15.03	1	1	1	1						24.63	0	1						24.37		24.37	NA	NA	
Q13530	Serine incorporator 3 OS=Homo sapiens GN=SERINC3 PE=2 SV=2 - [SERC3_HUMAN]	15.01	1	7	7	26	26.15	26.32	26.43				3	0	26.31	26.39	26.49				26.40		0.000	0.000	
Q8NBZ7	UDP-glucuronic acid decarboxylase 1 OS=Homo sapiens GN=UXS1 PE=1 SV=1 - [UXS1_HUMAN]	15.00	1	3	3	6	23.23			23.01	22.63		1	2	23.39			22.77	22.85		23.39	22.81	NA	NA	-0.584
Q9BPZ3	Polyadenylate-binding protein-interacting protein 2 OS=Homo sapiens GN=PAIP2 PE=1 SV=1 - [PAIP2_HUMAN]	14.96	1	1	1	1						25.06	0	1						24.80		24.80	NA	NA	
P61011	Signal recognition particle 54 kDa protein OS=Homo sapiens GN=SRP54 PE=1 SV=1 - [SRP54_HUMAN]	14.88	1	7	7	10	26.21			22.91	22.75	23.40	1	3	26.37			22.67	22.97	23.14	26.37	22.92	NA	NA	-3.449
A1L188	Uncharacterized protein C17orf89 OS=Homo sapiens GN=C17orf89 PE=4 SV=1 - [CQ089_HUMAN]	14.86	1	1	1	1					17.29		0	1					17.51			17.51	NA	NA	
Q8TEA8	D-tyrosyl-tRNA(Tyr) deacylase 1 OS=Homo sapiens GN=DTD1 PE=1 SV=2 - [DTD1_HUMAN]	14.83	1	2	2	3				22.67		22.06	0	2				22.43		21.80		22.11	NA	NA	
P12955	Xaa-Pro dipeptidase OS=Homo sapiens GN=PEPD PE=1 SV=3 - [PEPD_HUMAN]	14.81	1	6	6	11			23.48	23.36	22.90	23.89	1	3			23.54	23.11	23.12	23.63	23.54	23.29	NA	NA	-0.253
Q00765	Receptor expression-enhancing protein 5 OS=Homo sapiens GN=REEP5 PE=1 SV=3 - [REEP5_HUMAN]	14.81	1	3	3	3			23.36			24.15	1	1			23.42			23.89	23.42	23.89	NA	NA	0.467
Q86V81	THO complex subunit 4 OS=Homo sapiens GN=ALYREF PE=1 SV=3 - [THOC4_HUMAN]	14.79	1	2	2	6				21.02	21.83		0	2				20.77	22.04			21.41	NA	NA	
P35237	Serpin B6 OS=Homo sapiens GN=SERPINB6 PE=1 SV=3 - [SPB6_HUMAN]	14.63	2	4	4	6	23.14			23.72		23.75	1	2	23.30			23.47		23.49	23.30	23.48	NA	NA	0.183
Q96AP7	Endothelial cell-selective adhesion molecule OS=Homo sapiens GN=ESAM PE=1 SV=1 - [ESAM_HUMAN]	14.62	1	3	3	5		24.72	23.64		22.41		2	1		24.79	23.70		22.63		24.25	22.63	NA	NA	-1.619
Q9GZT8	NIF3-like protein 1 OS=Homo sapiens GN=NIF3L1 PE=1 SV=2 - [NIF3L_HUMAN]	14.59	1	4	4	7				23.04	23.17	23.75	0	3				22.79	23.39	23.49		23.23	0.000	0.000	
Q8TCT9	Minor histocompatibility antigen H13 OS=Homo sapiens GN=HM13 PE=1 SV=1 - [HM13_HUMAN]	14.59	1	4	4	5					22.97	23.90	0	2					23.18	23.64		23.41	NA	NA	
P61289	Proteasome activator complex subunit 3 OS=Homo sapiens GN=PSME3 PE=1 SV=1 - [PSME3_HUMAN]	14.57	1	3	3	5				24.38	24.02	24.49	0	3				24.14	24.24	24.23		24.20	0.000	0.000	
Q96K17	Transcription factor BTF3 homolog 4 OS=Homo sapiens GN=BTF3L4 PE=1 SV=1 - [BT3L4_HUMAN]	14.56	1	2	2	3				23.82	23.27	24.09	0	3				23.58	23.48	23.83		23.63	0.000	0.000	
P37198	Nuclear pore glycoprotein p62 OS=Homo sapiens GN=NUP62 PE=1 SV=3 - [NUP62_HUMAN]	14.56	1	4	4	6		21.26		22.97	24.75	23.50	1	3		21.33		22.73	24.96	23.24	21.33	23.64	NA	NA	2.308
Q14247	Src substrate cortactin OS=Homo sapiens GN=CTTN PE=1 SV=2 - [SRC8_HUMAN]	14.55	1	4	4	6				22.26		21.61	0	2				22.02		21.35		21.68	NA	NA	
Q8WXF1	Paraspeckle component 1 OS=Homo sapiens GN=PSPC1 PE=1 SV=1 - [PSPC1_HUMAN]	14.53	1	6	7	12	22.93			23.48	23.56	23.54	1	3	23.09			23.24	23.78	23.28	23.09	23.43	NA	NA	0.336
P05204	Non-histone chromosomal protein HMG-17 OS=Homo sapiens GN=HMGN2 PE=1 SV=3 - [HMGN2_HUMAN]	14.44	1	1	1	1		23.27					1	0		23.34					23.34		NA	NA	
O95833	Chloride intracellular channel protein 3 OS=Homo sapiens GN=CLIC3 PE=1 SV=2 - [CLIC3_HUMAN]	14.41	1	2	2	4	22.31	21.92	21.62				3	0	22.47	21.99	21.68				22.05		0.000	0.000	
Q9UJX2	Cell division cycle protein 23 homolog OS=Homo sapiens GN=CDC23 PE=1 SV=3 - [CDC23_HUMAN]	14.41	1	7	7	8	22.81	21.81	22.44				3	0	22.97	21.88	22.50				22.45		0.000	0.000	
Q99986	Serine/threonine-protein kinase VRK1 OS=Homo sapiens GN=VRK1 PE=1 SV=1 - [VRK1_HUMAN]	14.39	1	4	4	5				22.34	21.87		0	2				22.10	22.08			22.09	NA	NA	
Q9Y6M1	Insulin-like growth factor 2 mRNA-binding protein 2 OS=Homo sapiens GN=IGF2BP2 PE=1 SV=2 - [IF2B2_HUMAN]	14.19	1	5	6	14		22.09		23.02	23.28	23.43	1	3		22.16		22.77	23.50	23.17	22.16	23.15	NA	NA	0.988
P36543	V-type proton ATPase subunit E 1 OS=Homo sapiens GN=ATP6V1E1 PE=1 SV=1 - [VATE1_HUMAN]	14.16	1	3	3	8	21.96			23.28	22.36	22.67	1	3	22.12			23.04	22.58	22.41	22.12	22.68	NA	NA	0.557
Q15555	Microtubule-associated protein RP/EB family member 2 OS=Homo sapiens GN=MAPRE2 PE=1 SV=1 - [MARE2_HUMAN]	14.07	1	3	4	8	22.80			23.95	23.71	23.92	1	3	22.96			23.70	23.93	23.66	22.96	23.76	NA	NA	0.804
Q9Y2S6	Translation machinery-associated protein 7 OS=Homo sapiens GN=TMA7 PE=1 SV=1 - [TMA7_HUMAN]	14.06	1	1	1	3		24.27	24.47		22.62		2	1		24.33	24.53		22.84		24.43	22.84	NA	NA	-1.595
Q9NWV8	BRISC and BRCA1-A complex member 1 OS=Homo sapiens GN=BABAM1 PE=1 SV=1 - [BABA1_HUMAN]	13.98	1	3	3	5				23.23	21.13	22.14	0	3				22.98	21.34	21.88		22.07	0.000	0.000	
Q5T0D9	Tumor protein p63-regulated gene 1-like protein OS=Homo sapiens GN=TPRG1L PE=1 SV=1 - [TPRGL_HUMAN]	13.97	1	1	1	3				24.29	23.29		0	2				24.04	23.50			23.77	NA	NA	
Q9UMX5	Neudesin OS=Homo sapiens GN=NENF PE=1 SV=1 - [NENF_HUMAN]	13.95	1	2	2	2					23.78		0	1					24.00			24.00	NA	NA	
P27448	MAP/microtubule affinity-regulating kinase 3 OS=Homo sapiens GN=MARK3 PE=1 SV=4 - [MARK3_HUMAN]	13.94	3	4	7	12	22.54	22.09	21.17			19.38	3	1	22.70	22.16	21.23			19.12	22.03	19.12	NA	NA	-2.910
Q9UKJ5	Cysteine-rich hydrophobic domain-containing protein 2 OS=Homo sapiens GN=CHIC2 PE=1 SV=1 - [CHIC2_HUMAN]	13.94	1	1	1	3	23.10	23.10		21.73			2	1	23.26	23.17		21.49			23.22	21.49	NA	NA	-1.727
P09417	Dihydropteridine reductase OS=Homo sapiens GN=QDPR PE=1 SV=2 - [DHPR_HUMAN]	13.93	1	2	2	6				22.90	22.54	23.39	0	3				22.65	22.76	23.13		22.85	0.000	0.000	
Q8IY57	YY1-associated factor 2 OS=Homo sapiens GN=YAF2 PE=1 SV=3 - [YAF2_HUMAN]	13.89	1	1	1	1						19.52	0	1						19.26		19.26	NA	NA	
Q6RW13	Type-1 angiotensin II receptor-associated protein OS=Homo sapiens GN=AGTRAP PE=1 SV=1 - [ATRAP_HUMAN]	13.84	1	1	1	5	23.94	23.54	23.06	23.16			3	1	24.10	23.61	23.12	22.92			23.61	22.92	NA	NA	-0.690
Q8TAT6	Nuclear protein localization protein 4 homolog OS=Homo sapiens GN=NPLOC4 PE=1 SV=3 - [NPL4_HUMAN]	13.82	1	6	6	14	21.52			22.83	22.23	23.36	1	3	21.69			22.58	22.45	23.10	21.69	22.71	NA	NA	1.023
O43252	Bifunctional 3'-phosphoadenosine 5'-phosphosulfate synthase 1 OS=Homo sapiens GN=PAPSS1 PE=1 SV=2 - [PAPS1_HUMAN]	13.78	1	4	5	12				23.14	23.41	23.59	0	3				22.90	23.62	23.33		23.28	0.000	0.000	
Q8N4H5	Mitochondrial import receptor subunit TOM5 homolog OS=Homo sapiens GN=TOMM5 PE=1 SV=1 - [TOM5_HUMAN]	13.73	1	1	1	1			21.83				1	0			21.89				21.89		NA	NA	
Q16739	Ceramide glucosyltransferase OS=Homo sapiens GN=UGCG PE=1 SV=1 - [CEGT_HUMAN]	13.71	1	3	3	7				23.53		22.56	0	2				23.28		22.30		22.79	NA	NA	
Q9Y6X5	Bis(5'-adenosyl)-triphosphatase ENPP4 OS=Homo sapiens GN=ENPP4 PE=1 SV=3 - [ENPP4_HUMAN]	13.69	2	3	3	11		24.13		24.08	23.17	23.80	1	3		24.20		23.83	23.39	23.54	24.20	23.59	NA	NA	-0.612
P30049	ATP synthase subunit delta, mitochondrial OS=Homo sapiens GN=ATP5D PE=1 SV=2 - [ATPD_HUMAN]	13.69	1	2	2	5		21.73			23.12	23.19	1	2		21.80			23.34	22.93	21.80	23.13	NA	NA	1.338
P53634	Dipeptidyl peptidase 1 OS=Homo sapiens GN=CTSC PE=1 SV=2 - [CATC_HUMAN]	13.61	1	4	4	6		23.50		23.00	22.43	23.53	1	3		23.57		22.75	22.65	23.27	23.57	22.89	NA	NA	-0.679
Q16531	DNA damage-binding protein 1 OS=Homo sapiens GN=DDB1 PE=1 SV=1 - [DDB1_HUMAN]	13.60	1	12	12	21				23.71	23.26	23.88	0	3				23.47	23.47	23.62		23.52	0.000	0.000	
P00390	Glutathione reductase, mitochondrial OS=Homo sapiens GN=GSR PE=1 SV=2 - [GSHR_HUMAN]	13.60	1	4	4	7		20.91		23.87	22.96	23.92	1	3		20.98		23.62	23.18	23.66	20.98	23.49	NA	NA	2.506
Q14108	Lysosome membrane protein 2 OS=Homo sapiens GN=SCARB2 PE=1 SV=2 - [SCRB2_HUMAN]	13.60	1	4	4	12			22.85	24.18	23.07	23.95	1	3			22.91	23.94	23.29	23.69	22.91	23.64	NA	NA	0.730
Q9H0A8	COMM domain-containing protein 4 OS=Homo sapiens GN=COMMD4 PE=1 SV=1 - [COMD4_HUMAN]	13.57	1	2	2	5		23.43	23.66			20.11	2	1		23.50	23.72			19.85	23.61	19.85	NA	NA	-3.752
Q6PHR2	Serine/threonine-protein kinase ULK3 OS=Homo sapiens GN=ULK3 PE=1 SV=2 - [ULK3_HUMAN]	13.56	1	5	5	14	23.60	22.39	23.29				3	0	23.76	22.46	23.35				23.19		0.000	0.000	
Q969G6	Riboflavin kinase OS=Homo sapiens GN=RFK PE=1 SV=2 - [RIFK_HUMAN]	13.55	1	1	1	3				21.68	22.05	22.10	0	3				21.44	22.27	21.84		21.85	0.000	0.000	
Q9Y3D0	Mitotic spindle-associated MMXD complex subunit MIP18 OS=Homo sapiens GN=FAM96B PE=1 SV=1 - [MIP18_HUMAN]	13.50	1	1	1	6	22.83			23.97	23.51	23.87	1	3	23.00			23.73	23.73	23.61	23.00	23.69	NA	NA	0.693
P32321	Deoxycytidylate deaminase OS=Homo sapiens GN=DCTD PE=1 SV=2 - [DCTD_HUMAN]	13.48	1	2	2	2				22.56			0	1				22.31				22.31	NA	NA	
Q92572	AP-3 complex subunit sigma-1 OS=Homo sapiens GN=AP3S1 PE=1 SV=1 - [AP3S1_HUMAN]	13.47	1	2	2	5				22.16	23.18	22.95	0	3				21.92	23.39	22.68		22.66	0.000	0.000	
Q96C36	Pyrroline-5-carboxylate reductase 2 OS=Homo sapiens GN=PYCR2 PE=1 SV=1 - [P5CR2_HUMAN]	13.44	2	4	4	6					23.30	22.67	0	2					23.51	22.40		22.96	NA	NA	
O95983	Methyl-CpG-binding domain protein 3 OS=Homo sapiens GN=MBD3 PE=1 SV=1 - [MBD3_HUMAN]	13.40	1	1	1	2				21.84		21.87	0	2				21.59		21.61		21.60	NA	NA	
O95881	Thioredoxin domain-containing protein 12 OS=Homo sapiens GN=TXNDC12 PE=1 SV=1 - [TXD12_HUMAN]	13.37	1	2	2	3					24.82		0	1					25.04			25.04	NA	NA	
Q9Y3D8	Adenylate kinase isoenzyme 6 OS=Homo sapiens GN=AK6 PE=1 SV=1 - [KAD6_HUMAN]	13.37	1	1	1	2					22.10		0	1					22.32			22.32	NA	NA	
P05091	Aldehyde dehydrogenase, mitochondrial OS=Homo sapiens GN=ALDH2 PE=1 SV=2 - [ALDH2_HUMAN]	13.35	1	4	4	7					22.44	22.91	0	2					22.66	22.65		22.65	NA	NA	
Q8IY81	pre-rRNA processing protein FTSJ3 OS=Homo sapiens GN=FTSJ3 PE=1 SV=2 - [SPB1_HUMAN]	13.34	1	7	7	10				22.62	23.36	23.66	0	3				22.38	23.58	23.40		23.12	0.000	0.000	
P49662	Caspase-4 OS=Homo sapiens GN=CASP4 PE=1 SV=1 - [CASP4_HUMAN]	13.26	1	3	3	3				24.37	20.71		0	2				24.12	20.92			22.52	NA	NA	
O95232	Luc7-like protein 3 OS=Homo sapiens GN=LUC7L3 PE=1 SV=2 - [LC7L3_HUMAN]	13.19	1	3	3	6				23.09		22.75	0	2				22.84		22.49		22.67	NA	NA	
P07384	Calpain-1 catalytic subunit OS=Homo sapiens GN=CAPN1 PE=1 SV=1 - [CAN1_HUMAN]	13.17	1	5	5	7		22.30	22.87			24.15	2	1		22.37	22.93			23.89	22.65	23.89	NA	NA	1.239
Q92905	COP9 signalosome complex subunit 5 OS=Homo sapiens GN=COPS5 PE=1 SV=4 - [CSN5_HUMAN]	13.17	1	3	3	4					22.73	23.71	0	2					22.94	23.45		23.20	NA	NA	
Q9UBR5	Chemokine-like factor OS=Homo sapiens GN=CKLF PE=2 SV=1 - [CKLF_HUMAN]	13.16	1	2	2	3		23.66					1	0		23.73					23.73		NA	NA	
Q14978	Nucleolar and coiled-body phosphoprotein 1 OS=Homo sapiens GN=NOLC1 PE=1 SV=2 - [NOLC1_HUMAN]	13.16	1	5	5	9				22.68	22.20	21.06	0	3				22.44	22.42	20.80		21.88	0.000	0.000	
P50213	Isocitrate dehydrogenase [NAD] subunit alpha, mitochondrial OS=Homo sapiens GN=IDH3A PE=1 SV=1 - [IDH3A_HUMAN]	13.11	1	3	3	6	22.89		21.70		23.52	23.13	2	2	23.05		21.76		23.73	22.87	22.40	23.30	0.366	0.131	0.900
Q8N5M9	Protein jagunal homolog 1 OS=Homo sapiens GN=JAGN1 PE=1 SV=1 - [JAGN1_HUMAN]	13.11	1	2	2	6		19.93		20.81	21.10	22.59	1	3		20.00		20.57	21.31	22.33	20.00	21.40	NA	NA	1.407
Q07666	KH domain-containing, RNA-binding, signal transduction-associated protein 1 OS=Homo sapiens GN=KHDRBS1 PE=1 SV=1 - [KHDR1_HUMAN]	13.09	2	4	5	13	22.86			24.36	24.42	24.35	1	3	23.02			24.11	24.63	24.09	23.02	24.28	NA	NA	1.257
Q96BQ5	Coiled-coil domain-containing protein 127 OS=Homo sapiens GN=CCDC127 PE=1 SV=1 - [CC127_HUMAN]	13.08	1	1	1	1			20.67				1	0			20.73				20.73		NA	NA	
Q9UHY7	Enolase-phosphatase E1 OS=Homo sapiens GN=ENOPH1 PE=1 SV=1 - [ENOPH_HUMAN]	13.03	1	2	2	5				23.06	22.96	23.48	0	3				22.81	23.18	23.22		23.07	0.000	0.000	
P35250	Replication factor C subunit 2 OS=Homo sapiens GN=RFC2 PE=1 SV=3 - [RFC2_HUMAN]	12.99	1	3	3	3					22.08		0	1					22.30			22.30	NA	NA	
Q9NR28	Diablo homolog, mitochondrial OS=Homo sapiens GN=DIABLO PE=1 SV=1 - [DBLOH_HUMAN]	12.97	1	2	2	4				22.41	22.30		0	2				22.17	22.52			22.34	NA	NA	
Q00688	Peptidyl-prolyl cis-trans isomerase FKBP3 OS=Homo sapiens GN=FKBP3 PE=1 SV=1 - [FKBP3_HUMAN]	12.95	1	2	2	6		22.82	22.74	23.00	23.63		2	2		22.89	22.80	22.76	23.85		22.84	23.30	0.489	0.167	0.459

P08572	Collagen alpha-2(IV) chain OS=Homo sapiens GN=COL4A2 PE=1 SV=4 - [CO4A2_HUMAN]	12.91	1	13	13	38	25.43	24.74	26.21		21.45		3	1	25.59	24.81	26.27		21.66		25.56	21.66	NA	NA	-3.892
P78310	Coxsackievirus and adenovirus receptor OS=Homo sapiens GN=CXADR PE=1 SV=1 - [CXAR_HUMAN]	12.88	1	3	3	5				23.38		22.71	0	2				23.13		22.45		22.79	NA	NA	
O43818	U3 small nucleolar RNA-interacting protein 2 OS=Homo sapiens GN=RRP9 PE=1 SV=1 - [U3IP2_HUMAN]	12.84	1	2	2	2					21.69		0	1					21.91			21.91	NA	NA	
Q6UVK1	Chondroitin sulfate proteoglycan 4 OS=Homo sapiens GN=CSPG4 PE=1 SV=2 - [CSPG4_HUMAN]	12.83	1	19	19	30	23.92	23.90	23.61				3	0	24.09	23.97	23.67				23.91		0.000	0.000	
Q8NBT0	POC1 centriolar protein homolog A OS=Homo sapiens GN=POC1A PE=1 SV=2 - [POC1A_HUMAN]	12.78	1	3	3	8	23.32	23.58	23.64				3	0	23.49	23.65	23.70				23.61		0.000	0.000	
P35052	Glypican-1 OS=Homo sapiens GN=GPC1 PE=1 SV=2 - [GPC1_HUMAN]	12.72	1	4	4	7	23.21	22.70	23.04				3	0	23.38	22.77	23.10				23.08		0.000	0.000	
Q9NZL4	Hsp70-binding protein 1 OS=Homo sapiens GN=HSPBP1 PE=1 SV=1 - [HPBP1_HUMAN]	12.71	1	4	4	7				21.60		22.53	0	2				21.36		22.27		21.81	NA	NA	
Q96SB4	SRSF protein kinase 1 OS=Homo sapiens GN=SRPK1 PE=1 SV=2 - [SRPK1_HUMAN]	12.67	3	5	5	6				22.13	22.99	21.93	0	3				21.89	23.20	21.67		22.25	0.000	0.000	
P33240	Cleavage stimulation factor subunit 2 OS=Homo sapiens GN=CSTF2 PE=1 SV=1 - [CSTF2_HUMAN]	12.65	1	2	5	10				23.51	23.16	22.86	0	3				23.26	23.38	22.59		23.08	0.000	0.000	
Q9BUT1	3-hydroxybutyrate dehydrogenase type 2 OS=Homo sapiens GN=BDH2 PE=1 SV=2 - [BDH2_HUMAN]	12.65	1	2	2	3				22.39		22.70	0	2				22.15		22.44		22.30	NA	NA	
P22307	Non-specific lipid-transfer protein OS=Homo sapiens GN=SCP2 PE=1 SV=2 - [NLTP_HUMAN]	12.61	1	6	6	17			20.60	22.91	24.10	24.01	1	3			20.66	22.66	24.31	23.75	20.66	23.58	NA	NA	2.919
O95831	Apoptosis-inducing factor 1, mitochondrial OS=Homo sapiens GN=AIFM1 PE=1 SV=1 - [AIFM1_HUMAN]	12.56	1	6	6	10				22.33	23.14	23.43	0	3				22.09	23.36	23.17		22.87	0.000	0.000	
P08559	Pyruvate dehydrogenase E1 component subunit alpha, somatic form, mitochondrial OS=Homo sapiens GN=PDHA1 PE=1 SV=3 - [ODPA_HUMAN]	12.56	2	3	3	3			22.83		20.80		1	1			22.89		21.02		22.89	21.02	NA	NA	-1.876
P51608	Methyl-CpG-binding protein 2 OS=Homo sapiens GN=MECP2 PE=1 SV=1 - [MECP2_HUMAN]	12.55	1	4	4	8				22.49	19.09	21.82	0	3				22.24	19.31	21.56		21.04	0.000	0.000	
Q9GZP9	Derlin-2 OS=Homo sapiens GN=DERL2 PE=1 SV=1 - [DERL2_HUMAN]	12.55	1	1	1	4			22.98		23.47	24.45	1	2			23.04		23.68	24.19	23.04	23.94	NA	NA	0.898
P36404	ADP-ribosylation factor-like protein 2 OS=Homo sapiens GN=ARL2 PE=1 SV=4 - [ARL2_HUMAN]	12.50	1	2	2	7	21.53			22.68	22.78	22.90	1	3	21.69			22.43	23.00	22.64	21.69	22.69	NA	NA	0.997
P04233	HLA class II histocompatibility antigen gamma chain OS=Homo sapiens GN=CD74 PE=1 SV=3 - [HG2A_HUMAN]	12.50	1	3	3	6				24.11	24.57	23.45	0	3				23.86	24.79	23.19		23.95	0.000	0.000	
Q8WUY1	Protein THEM6 OS=Homo sapiens GN=THEM6 PE=1 SV=2 - [THEM6_HUMAN]	12.50	1	1	1	1						23.91	0	1						23.65		23.65	NA	NA	
Q16666	Gamma-interferon-inducible protein 16 OS=Homo sapiens GN=IFI16 PE=1 SV=3 - [IF16_HUMAN]	12.48	1	7	7	13	20.68			22.11	22.99	22.27	1	3	20.85			21.86	23.21	22.01	20.85	22.36	NA	NA	1.512
Q9UJA5	tRNA (adenine(58)-N(1))-methyltransferase non-catalytic subunit TRM6 OS=Homo sapiens GN=TRMT6 PE=1 SV=1 - [TRM6_HUMAN]	12.47	1	4	4	7				22.71	22.73	23.10	0	3				22.47	22.94	22.84		22.75	0.000	0.000	
Q9Y5K5	Ubiquitin carboxyl-terminal hydrolase isozyme L5 OS=Homo sapiens GN=UCHL5 PE=1 SV=3 - [UCHL5_HUMAN]	12.46	1	2	2	2						23.39	0	1						23.13		23.13	NA	NA	
P50895	Basal cell adhesion molecule OS=Homo sapiens GN=BCAM PE=1 SV=2 - [BCAM_HUMAN]	12.42	1	4	4	5	20.55	22.26	24.20				3	0	20.71	22.33	24.26				22.43		0.000	0.000	
Q9GZM7	Tubulointerstitial nephritis antigen-like OS=Homo sapiens GN=TINAGL1 PE=1 SV=1 - [TINAL_HUMAN]	12.42	1	3	3	3	23.20						1	0	23.37						23.37		NA	NA	
O94905	Erlin-2 OS=Homo sapiens GN=ERLIN2 PE=1 SV=1 - [ERLN2_HUMAN]	12.39	1	2	3	3						22.42	0	1						22.16		22.16	NA	NA	
CON_007150335	Bos taurus Gene_Symbol=- 45 kDa protein	12.38	1	4	4	8	22.03			21.52	21.65	22.83	1	3	22.19			21.28	21.87	22.57	22.19	21.91	NA	NA	-0.289
P08240	Signal recognition particle receptor subunit alpha OS=Homo sapiens GN=SRPR PE=1 SV=2 - [SRPR_HUMAN]	12.38	1	6	6	11				22.39	22.61	23.38	0	3				22.15	22.82	23.12		22.70	0.000	0.000	
Q9UJX3	Anaphase-promoting complex subunit 7 OS=Homo sapiens GN=ANAPC7 PE=1 SV=4 - [APC7_HUMAN]	12.35	1	5	5	7	22.73		21.74			23.34	2	1	22.89		21.80			23.08	22.35	23.08	NA	NA	0.734
Q9H773	dCTP pyrophosphatase 1 OS=Homo sapiens GN=DCTPP1 PE=1 SV=1 - [DCTP1_HUMAN]	12.35	1	2	2	2				21.61			0	1				21.36				21.36	NA	NA	
P53367	Arfaptin-1 OS=Homo sapiens GN=ARFIP1 PE=1 SV=2 - [ARFP1_HUMAN]	12.33	1	4	4	7	25.30			23.34	22.44	22.61	1	3	25.46			23.09	22.66	22.35	25.46	22.70	NA	NA	-2.758
Q7Z5R6	Amyloid beta A4 precursor protein-binding family B member 1-interacting protein OS=Homo sapiens GN=APBB1IP PE=1 SV=1 - [AB1IP_HUMAN]	12.31	1	5	6	13		20.47		23.09	22.58	23.42	1	3		20.54		22.84	22.80	23.16	20.54	22.93	NA	NA	2.396
P29218	Inositol monophosphatase 1 OS=Homo sapiens GN=IMPA1 PE=1 SV=1 - [IMPA1_HUMAN]	12.27	1	2	2	6			21.45	23.04	22.95	23.66	1	3			21.51	22.79	23.17	23.40	21.51	23.12	NA	NA	1.610
Q86SJ2	Amphoterin-induced protein 2 OS=Homo sapiens GN=AMIGO2 PE=1 SV=1 - [AMGO2_HUMAN]	12.26	1	4	4	7	22.58	22.53	22.67				3	0	22.74	22.60	22.73				22.69		0.000	0.000	
Q6I9Y2	THO complex subunit 7 homolog OS=Homo sapiens GN=THOC7 PE=1 SV=3 - [THOC7_HUMAN]	12.25	1	2	2	2					21.89		0	1					22.11			22.11	NA	NA	
Q8NCW5	NAD(P)H-hydrate epimerase OS=Homo sapiens GN=APOA1BP PE=1 SV=2 - [NNRE_HUMAN]	12.15	1	2	2	2			21.38			23.77	1	1			21.44			23.51	21.44	23.51	NA	NA	2.068
Q15813	Tubulin-specific chaperone E OS=Homo sapiens GN=TBCE PE=1 SV=1 - [TBCE_HUMAN]	12.14	1	3	3	11	22.02			23.48	22.78	23.70	1	3	22.18			23.23	23.00	23.44	22.18	23.22	NA	NA	1.039
Q04446	1,4-alpha-glucan-branching enzyme OS=Homo sapiens GN=GBE1 PE=1 SV=3 - [GLGB_HUMAN]	12.11	1	5	5	9	22.69	22.43	22.47				3	0	22.85	22.50	22.52				22.62		0.000	0.000	
P28331	NADH-ubiquinone oxidoreductase 75 kDa subunit, mitochondrial OS=Homo sapiens GN=NDUFS1 PE=1 SV=3 - [NDUS1_HUMAN]	12.10	1	6	6	7					21.83	22.04	0	2					22.04	21.78		21.91	NA	NA	
Q9BR77	Coiled-coil domain-containing protein 77 OS=Homo sapiens GN=CCDC77 PE=2 SV=1 - [CCD77_HUMAN]	12.09	1	5	5	11	22.66	23.21	23.23				3	0	22.82	23.28	23.29				23.13		0.000	0.000	
Q86X83	COMM domain-containing protein 2 OS=Homo sapiens GN=COMMD2 PE=1 SV=2 - [COMD2_HUMAN]	12.06	1	1	1	1					22.49		0	1					22.70			22.70	NA	NA	
Q9NZ45	CDGSH iron-sulfur domain-containing protein 1 OS=Homo sapiens GN=CISD1 PE=1 SV=1 - [CISD1_HUMAN]	12.04	1	1	1	2					22.81	23.21	0	2					23.02	22.95		22.99	NA	NA	
O15116	U6 snRNA-associated Sm-like protein LSm1 OS=Homo sapiens GN=LSM1 PE=1 SV=1 - [LSM1_HUMAN]	12.03	1	1	1	3				23.70	23.49	23.48	0	3				23.46	23.71	23.22		23.46	0.000	0.000	
O75822	Eukaryotic translation initiation factor 3 subunit J OS=Homo sapiens GN=EIF3J PE=1 SV=2 - [EIF3J_HUMAN]	12.02	1	2	2	2				23.61			0	1				23.36				23.36	NA	NA	
Q8NFH4	Nucleoporin Nup37 OS=Homo sapiens GN=NUP37 PE=1 SV=1 - [NUP37_HUMAN]	11.96	1	2	2	3					21.87		0	1					22.09			22.09	NA	NA	
Q6P2Q9	Pre-mRNA-processing-splicing factor 8 OS=Homo sapiens GN=PRPF8 PE=1 SV=2 - [PRP8_HUMAN]	11.95	1	18	18	41	22.57			24.17	24.10	24.61	1	3	22.73			23.93	24.32	24.35	22.73	24.20	NA	NA	1.465
Q9BQ39	ATP-dependent RNA helicase DDX50 OS=Homo sapiens GN=DDX50 PE=1 SV=1 - [DDX50_HUMAN]	11.94	1	4	6	8				24.35	23.98	25.42	0	3				24.11	24.20	25.16		24.49	0.000	0.000	
Q9NYL9	Tropomodulin-3 OS=Homo sapiens GN=TMOD3 PE=1 SV=1 - [TMOD3_HUMAN]	11.93	1	3	3	5					22.76	23.44	0	2					22.98	23.18		23.08	NA	NA	
O00421	C-C chemokine receptor-like 2 OS=Homo sapiens GN=CCRL2 PE=1 SV=2 - [CCRL2_HUMAN]	11.92	1	2	2	5		22.90	22.99			22.74	2	1		22.97	23.05			22.48	23.01	22.48	NA	NA	-0.537
P11310	Medium-chain specific acyl-CoA dehydrogenase, mitochondrial OS=Homo sapiens GN=ACADM PE=1 SV=1 - [ACADM_HUMAN]	11.88	1	4	4	7					23.02	23.13	0	2					23.23	22.87		23.05	NA	NA	
Q7RTV0	PHD finger-like domain-containing protein 5A OS=Homo sapiens GN=PHF5A PE=1 SV=1 - [PHF5A_HUMAN]	11.82	1	1	1	1			20.89				1	0			20.95				20.95		NA	NA	
O14602	Eukaryotic translation initiation factor 1A, Y-chromosomal OS=Homo sapiens GN=EIF1AY PE=1 SV=4 - [IF1AY_HUMAN]	11.81	2	2	2	2				24.54	22.83		0	2				24.29	23.04			23.67	NA	NA	
O75607	Nucleoplasmin-3 OS=Homo sapiens GN=NPM3 PE=1 SV=3 - [NPM3_HUMAN]	11.80	1	1	1	1					23.98		0	1					24.19			24.19	NA	NA	
P21283	V-type proton ATPase subunit C 1 OS=Homo sapiens GN=ATP6V1C1 PE=1 SV=4 - [VATC1_HUMAN]	11.78	1	4	4	7	21.98				22.80	22.96	1	2	22.15				23.01	22.70	22.15	22.86	NA	NA	0.710
Q13435	Splicing factor 3B subunit 2 OS=Homo sapiens GN=SF3B2 PE=1 SV=2 - [SF3B2_HUMAN]	11.73	1	8	8	11					22.95	22.36	0	2					23.16	22.09		22.63	NA	NA	
Q9BT25	HAUS augmin-like complex subunit 8 OS=Homo sapiens GN=HAUS8 PE=1 SV=3 - [HAUS8_HUMAN]	11.71	1	2	2	6				22.05	22.29	21.92	0	3				21.81	22.51	21.66		21.99	0.000	0.000	
O00505	Importin subunit alpha-4 OS=Homo sapiens GN=KPNA3 PE=1 SV=2 - [IMA4_HUMAN]	11.71	1	3	3	5		22.94	22.94				2	0		23.01	23.00				23.00		NA	NA	
P56545	C-terminal-binding protein 2 OS=Homo sapiens GN=CTBP2 PE=1 SV=1 - [CTBP2_HUMAN]	11.69	1	2	2	6				21.71	23.19	23.31	0	3				21.46	23.41	23.05		22.64	0.000	0.000	
O00401	Neural Wiskott-Aldrich syndrome protein OS=Homo sapiens GN=WASL PE=1 SV=2 - [WASL_HUMAN]	11.68	1	3	3	5		22.00	22.61			22.64	2	1		22.07	22.67			22.38	22.37	22.38	NA	NA	0.013
P11172	Uridine 5'-monophosphate synthase OS=Homo sapiens GN=UMPS PE=1 SV=1 - [UMPS_HUMAN]	11.67	1	2	2	3	24.24		23.19				2	0	24.40		23.25				23.82		NA	NA	
P61009	Signal peptidase complex subunit 3 OS=Homo sapiens GN=SPCS3 PE=1 SV=1 - [SPCS3_HUMAN]	11.67	1	2	2	2					22.63	21.36	0	2					22.85	21.10		21.97	NA	NA	
Q13542	Eukaryotic translation initiation factor 4E-binding protein 2 OS=Homo sapiens GN=EIF4EBP2 PE=1 SV=1 - [4EBP2_HUMAN]	11.67	1	1	1	2					18.27	18.61	0	2					18.49	18.35		18.42	NA	NA	
Q7L014	Probable ATP-dependent RNA helicase DDX46 OS=Homo sapiens GN=DDX46 PE=1 SV=2 - [DDX46_HUMAN]	11.64	1	9	9	16				23.53	22.68	23.32	0	3				23.28	22.89	23.06		23.08	0.000	0.000	
P52815	39S ribosomal protein L12, mitochondrial OS=Homo sapiens GN=MRPL12 PE=1 SV=2 - [RM12_HUMAN]	11.62	1	3	3	5					23.49	23.61	0	2					23.70	23.35		23.53	NA	NA	
Q9H488	GDP-fucose protein O-fucosyltransferase 1 OS=Homo sapiens GN=POFUT1 PE=1 SV=1 - [OFUT1_HUMAN]	11.60	1	3	3	5				22.94	23.59	23.73	0	3				22.69	23.81	23.47		23.32	0.000	0.000	
Q8WZA0	Protein LZIC OS=Homo sapiens GN=LZIC PE=1 SV=1 - [LZIC_HUMAN]	11.58	1	1	1	1				21.32			0	1				21.07				21.07	NA	NA	
CON_007100251	SWISS-PROT:P50448 Bos taurus Factor XIIa inhibitor precursor	11.54	1	3	3	5	22.29			22.39		22.08	1	2	22.45			22.15		21.82	22.45	21.99	NA	NA	-0.465
O00764	Pyridoxal kinase OS=Homo sapiens GN=PDXK PE=1 SV=1 - [PDXK_HUMAN]	11.54	1	3	3	4			21.36		20.79	22.26	1	2			21.41		21.01	22.00	21.41	21.51	NA	NA	0.093
Q9NVS9	Pyridoxine-5'-phosphate oxidase OS=Homo sapiens GN=PNPO PE=1 SV=1 - [PNPO_HUMAN]	11.49	1	2	2	6		20.58		20.92	21.86	21.33	1	3		20.65		20.67	22.07	21.07	20.65	21.27	NA	NA	0.626
O95807	Transmembrane protein 50A OS=Homo sapiens GN=TMEM50A PE=1 SV=1 - [TM50A_HUMAN]	11.46	1	2	2	5	23.31	23.33	23.65				3	0	23.47	23.40	23.71				23.53		0.000	0.000	
Q96AT9	Ribulose-phosphate 3-epimerase OS=Homo sapiens GN=RPE PE=1 SV=1 - [RPE_HUMAN]	11.40	2	2	2	2				22.98		21.85	0	2				22.73		21.59		22.16	NA	NA	
P53582	Methionine aminopeptidase 1 OS=Homo sapiens GN=METAP1 PE=1 SV=2 - [MAP11_HUMAN]	11.40	1	3	3	6			19.56		23.61	23.62	1	2			19.62		23.82	23.36	19.62	23.59	NA	NA	3.976
O43237	Cytoplasmic dynein 1 light intermediate chain 2 OS=Homo sapiens GN=DYNC1LI2 PE=1 SV=1 - [DC1L2_HUMAN]	11.38	1	4	5	6		18.13			22.40	21.99	1	2		18.20			22.62	21.73	18.20	22.17	NA	NA	3.973
P43246	DNA mismatch repair protein Msh2 OS=Homo sapiens GN=MSH2 PE=1 SV=1 - [MSH2_HUMAN]	11.35	1	7	8	15				22.60	22.57	24.23	0	3				22.36	22.78	23.97		23.04	0.000	0.000	
Q9BRX8	Redox-regulatory protein FAM213A OS=Homo sapiens GN=FAM213A PE=1 SV=3 - [F213A_HUMAN]	11.35	1	2	2	6			22.27	21.72	22.63	23.01	1	3			22.33	21.47	22.84	22.74	22.33	22.35	NA	NA	0.026
Q13084	39S ribosomal protein L28, mitochondrial OS=Homo sapiens GN=MRPL28 PE=1 SV=4 - [RM28_HUMAN]	11.33	1	2	2	4		24.01			19.98	20.74	1	2		24.08			20.20	20.48	24.08	20.34	NA	NA	-3.740
O43920	NADH dehydrogenase [ubiquinone] iron-sulfur protein 5 OS=Homo sapiens GN=NDUFS5 PE=1 SV=3 - [NDUS5_HUMAN]	11.32	1	1	1	2					22.79	23.08	0	2					23.00	22.82		22.91	NA	NA	
Q13596	Sorting nexin-1 OS=Homo sapiens GN=SNX1 PE=1 SV=3 - [SNX1_HUMAN]	11.30	1	4	5	11			21.36		22.82		1	1			21.42		23.04		21.42	23.04	NA	NA	1.618
Q9UNP9	Peptidyl-prolyl cis-trans isomerase E OS=Homo sapiens GN=PPIE PE=1 SV=1 - [PPIE_HUMAN]	11.30	1	2	2	5				23.16	23.03	23.74	0	3				22.91	23.25	23.48		23.21	0.000	0.000	
Q96DE0	U8 snoRNA-decapping enzyme OS=Homo sapiens GN=NUDT16 PE=1 SV=2 - [NUD16_HUMAN]	11.28	1	1	1	1	22.09						1	0	22.25						22.25		NA	NA	
Q9Y2S0	DNA-directed RNA polymerases I and III subunit RPAC2 OS=Homo sapiens GN=POLR1D PE=1 SV=1 - [RPAC2_HUMAN]	11.28	1	1	1	1					23.21		0	1					23.43			23.43	NA	NA	
Q96T76	MMS19 nucleotide excision repair protein homolog OS=Homo sapiens GN=MMS19 PE=1 SV=2 - [MMS19_HUMAN]	11.26	1	6	6	12				22.16	22.82	23.58	0	3				21.91	23.03	23.32		22.75	0.000	0.000	
Q96F86	Enhancer of mRNA-decapping protein 3 OS=Homo sapiens GN=EDC3 PE=1 SV=1 - [EDC3_HUMAN]	11.22	1	3	3	4	22.50			23.98		22.78	1	2	22.66			23.73		22.52	22.66	23.13	NA	NA	0.463
P20036	HLA class II histocompatibility antigen, DP alpha 1 chain OS=Homo sapiens GN=HLA-DPA1 PE=1 SV=1 - [DPA1_HUMAN]	11.15	1	2	2	2				21.99		23.15	0	2				21.75		22.89		22.32	NA	NA	
O00139	Kinesin-like protein KIF2A OS=Homo sapiens GN=KIF2A PE=1 SV=3 - [KIF2A_HUMAN]	11.05	2	4	4	5				21.78	31.00	22.34	0	3				21.54	31.22	22.08		24.95	0.000	0.000	
Q13445	Transmembrane emp24 domain-containing protein 1 OS=Homo sapiens GN=TMED1 PE=1 SV=1 - [TMED1_HUMAN]	11.01	1	2	2	3				20.65		22.18	0	2				20.40		21.92		21.16	NA	NA	
Q0VDF9	Heat shock 70 kDa protein 14 OS=Homo sapiens GN=HSPA14 PE=1 SV=1 - [HSP7E_HUMAN]	11.00	1	3	3	6					21.75	23.28	0	2					21.97	23.02		22.50	NA	NA	
Q6ZUX7	LHFPL tetraspan subfamily member 2 protein OS=Homo sapiens GN=LHFPL2 PE=2 SV=2 - [LHPL2_HUMAN]	10.96	1	1	1	5	23.90	24.36	24.41			23.77	3	1	24.06	24.43	24.47			23.51	24.32	23.51	NA	NA	-0.809
P51151	Ras-related protein Rab-9A OS=Homo sapiens GN=RAB9A PE=1 SV=1 - [RAB9A_HUMAN]	10.95	1	2	2	6	23.28	22.71		23.37		23.36	2	2	23.44	22.78		23.13		23.10	23.11	23.11	0.996	0.281	-0.002
Q16625	Occludin OS=Homo sapiens GN=OCLN PE=1 SV=1 - [OCLN_HUMAN]	10.92	1	5	5	12			22.17	23.91	23.33	23.57	1	3			22.22	23.66	23.55	23.31	22.22	23.51	NA	NA	1.284
P51809	Vesicle-associated membrane protein 7 OS=Homo sapiens GN=VAMP7 PE=1 SV=3 - [VAMP7_HUMAN]	10.91	1	2	2	4	22.30			23.87	22.93		1	2	22.46			23.62	23.15		22.46	23.38	NA	NA	0.926
O75896	Tumor suppressor candidate 2 OS=Homo sapiens GN=TUSC2 PE=1 SV=3 - [TUSC2_HUMAN]	10.91	1	1	1	1				23.10			0	1				22.85				22.85	NA	NA	
O95218	Zinc finger Ran-binding domain-containing protein 2 OS=Homo sapiens GN=ZRANB2 PE=1 SV=2 - [ZRAB2_HUMAN]	10.91	1	2	2	2					21.69		0	1					21.90			21.90	NA	NA	
Q969S3	Zinc finger protein 622 OS=Homo sapiens GN=ZNF622 PE=1 SV=1 - [ZN622_HUMAN]	10.90	1	4	4	9				22.95	23.04	22.99	0	3				22.70	23.25	22.73		22.90	0.000	0.000	
Q99470	Stromal cell-derived factor 2 OS=Homo sapiens GN=SDF2 PE=1 SV=2 - [SDF2_HUMAN]	10.90	1	1	1	2					23.43	23.91	0	2					23.64	23.65		23.65	NA	NA	
O14745	Na(+)/H(+) exchange regulatory cofactor NHE-RF1 OS=Homo sapiens GN=SLC9A3R1 PE=1 SV=4 - [NHRF1_HUMAN]	10.89	1	3	3	5				24.71	25.00	24.73	0	3				24.46	25.21	24.46		24.71	0.000	0.000	
P31483	Nucleolysin TIA-1 isoform p40 OS=Homo sapiens GN=TIA1 PE=1 SV=3 - [TIA1_HUMAN]	10.88	1	1	3	4		21.66			23.10	20.45	1	2		21.73			23.32	20.19	21.73	21.75	NA	NA	0.028
Q9P2X0	Dolichol-phosphate mannosyltransferase subunit 3 OS=Homo sapiens GN=DPM3 PE=1 SV=2 - [DPM3_HUMAN]	10.87	1	1	1	3						22.65	0	1						22.39		22.39	NA	NA	
Q86Y82	Syntaxin-12 OS=Homo sapiens GN=STX12 PE=1 SV=1 - [STX12_HUMAN]	10.87	1	2	2	2				23.41		22.48	0	2				23.16		22.22		22.69	NA	NA	
Q92888	Rho guanine nucleotide exchange factor 1 OS=Homo sapiens GN=ARHGEF1 PE=1 SV=2 - [ARHG1_HUMAN]	10.86	1	8	8	12			21.82	23.28	22.62	23.41	1	3			21.88	23.03	22.84	23.15	21.88	23.01	NA	NA	1.126
Q96C19	EF-hand domain-containing protein D2 OS=Homo sapiens GN=EFHD2 PE=1 SV=1 - [EFHD2_HUMAN]	10.83	2	2	3	4				22.42	21.28		0	2				22.17	21.49			21.83	NA	NA	
Q16740	ATP-dependent Clp protease proteolytic subunit, mitochondrial OS=Homo sapiens GN=CLPP PE=1 SV=1 - [CLPP_HUMAN]	10.83	1	2	2	3						23.12	0	1						22.86		22.86	NA	NA	
Q86WQ0	Nuclear receptor 2C2-associated protein OS=Homo sapiens GN=NR2C2AP PE=1 SV=1 - [NR2CA_HUMAN]	10.79	1	1	1	1				21.37			0	1				21.12				21.12	NA	NA	
Q9H246	Uncharacterized protein C1orf21 OS=Homo sapiens GN=C1orf21 PE=1 SV=1 - [CA021_HUMAN]	10.74	1	1	1	4	19.23	19.57				19.00	2	1	19.40	19.63				18.74	19.52	18.74	NA	NA	-0.774
Q9BZX2	Uridine-cytidine kinase 2 OS=Homo sapiens GN=UCK2 PE=1 SV=1 - [UCK2_HUMAN]	10.73	1	2	2	2						23.95	0	1						23.69		23.69	NA	NA	
O95214	Leptin receptor overlapping transcript-like 1 OS=Homo sapiens GN=LEPROTL1 PE=2 SV=2 - [LERL1_HUMAN]	10.69	1	2	2	5	22.44	22.17	22.73				3	0	22.60	22.24	22.78				22.54		0.000	0.000	
P61803	Dolichyl-diphosphooligosaccharide--protein glycosyltransferase subunit DAD1 OS=Homo sapiens GN=DAD1 PE=1 SV=3 - [DAD1_HUMAN]	10.62	1	1	1	1						23.13	0	1						22.87		22.87	NA	NA	
Q04771	Activin receptor type-1 OS=Homo sapiens GN=ACVR1 PE=1 SV=1 - [ACVR1_HUMAN]	10.61	1	2	2	2	23.14	20.56					2	0	23.30	20.63					21.96		NA	NA	
O95147	Dual specificity protein phosphatase 14 OS=Homo sapiens GN=DUSP14 PE=1 SV=1 - [DUS14_HUMAN]	10.61	1	1	1	3	23.52	22.73	23.44				3	0	23.68	22.80	23.50				23.33		0.000	0.000	
Q14145	Kelch-like ECH-associated protein 1 OS=Homo sapiens GN=KEAP1 PE=1 SV=2 - [KEAP1_HUMAN]	10.58	1	4	4	6	22.03			22.57		22.30	1	2	22.19			22.32		22.04	22.19	22.18	NA	NA	-0.015
Q9HD67	Unconventional myosin-X OS=Homo sapiens GN=MYO10 PE=1 SV=3 - [MYO10_HUMAN]	10.54	1	14	14	21	22.91	21.13	23.27				3	0	23.07	21.20	23.33				22.54		0.000	0.000	
Q9BSD7	Cancer-related nucleoside-triphosphatase OS=Homo sapiens GN=NTPCR PE=1 SV=1 - [NTPCR_HUMAN]	10.53	1	1	1	3				21.66	21.62		0	2				21.42	21.83			21.63	NA	NA	
Q8N8S7	Protein enabled homolog OS=Homo sapiens GN=ENAH PE=1 SV=2 - [ENAH_HUMAN]	10.49	1	4	4	5				23.29	22.28	23.25	0	3				23.05	22.50	22.99		22.85	0.000	0.000	
Q96IU4	Alpha/beta hydrolase domain-containing protein 14B OS=Homo sapiens GN=ABHD14B PE=1 SV=1 - [ABHEB_HUMAN]	10.48	1	1	1	4	23.95			24.57	24.29		1	2	24.11			24.33	24.50		24.11	24.42	NA	NA	0.302
Q53EL6	Programmed cell death protein 4 OS=Homo sapiens GN=PDCD4 PE=1 SV=2 - [PDCD4_HUMAN]	10.45	1	3	3	3				20.77		23.40	0	2				20.52		23.14		21.83	NA	NA	
Q5W0Z9	Probable palmitoyltransferase ZDHHC20 OS=Homo sapiens GN=ZDHHC20 PE=1 SV=1 - [ZDH20_HUMAN]	10.41	1	2	2	3	21.62	21.86					2	0	21.78	21.93					21.86		NA	NA	
O75477	Erlin-1 OS=Homo sapiens GN=ERLIN1 PE=1 SV=1 - [ERLN1_HUMAN]	10.40	1	2	3	7	20.97			22.51	22.78	22.93	1	3	21.13			22.27	23.00	22.66	21.13	22.64	NA	NA	1.516
Q14677	Clathrin interactor 1 OS=Homo sapiens GN=CLINT1 PE=1 SV=1 - [EPN4_HUMAN]	10.40	1	5	5	12		21.19		22.74	22.44	22.89	1	3		21.26		22.50	22.65	22.63	21.26	22.59	NA	NA	1.332
Q9HB07	UPF0160 protein MYG1, mitochondrial OS=Homo sapiens GN=C12orf10 PE=1 SV=2 - [MYG1_HUMAN]	10.37	1	3	3	3				22.74	21.70		0	2				22.50	21.92			22.21	NA	NA	
O15126	Secretory carrier-associated membrane protein 1 OS=Homo sapiens GN=SCAMP1 PE=1 SV=2 - [SCAM1_HUMAN]	10.36	1	3	3	7	21.70			20.92	22.06	20.46	1	3	21.87			20.67	22.28	20.20	21.87	21.05	NA	NA	-0.817
O95347	Structural maintenance of chromosomes protein 2 OS=Homo sapiens GN=SMC2 PE=1 SV=2 - [SMC2_HUMAN]	10.36	1	9	9	17				23.25	23.49	23.81	0	3				23.00	23.71	23.55		23.42	0.000	0.000	
O43172	U4/U6 small nuclear ribonucleoprotein Prp4 OS=Homo sapiens GN=PRPF4 PE=1 SV=2 - [PRP4_HUMAN]	10.34	1	4	4	7				22.24	21.49	22.44	0	3				21.99	21.71	22.18		21.96	0.000	0.000	
Q99622	Protein C10 OS=Homo sapiens GN=C12orf57 PE=1 SV=1 - [C10_HUMAN]	10.32	1	1	1	1					22.76		0	1					22.97			22.97	NA	NA	
Q9Y3C4	EKC/KEOPS complex subunit TPRKB OS=Homo sapiens GN=TPRKB PE=1 SV=1 - [TPRKB_HUMAN]	10.29	1	1	1	1					21.34		0	1					21.55			21.55	NA	NA	
Q8WUN7	Ubiquitin domain-containing protein 2 OS=Homo sapiens GN=UBTD2 PE=1 SV=2 - [UBTD2_HUMAN]	10.26	1	1	2	5	23.21	23.72	22.73				3	0	23.37	23.79	22.79				23.32		0.000	0.000	
Q9H3M7	Thioredoxin-interacting protein OS=Homo sapiens GN=TXNIP PE=1 SV=1 - [TXNIP_HUMAN]	10.23	1	2	2	2	23.52						1	0	23.68						23.68		NA	NA	
P04818	Thymidylate synthase OS=Homo sapiens GN=TYMS PE=1 SV=3 - [TYSY_HUMAN]	10.22	1	3	3	6	22.12		22.32		21.91		2	1	22.28		22.37		22.12		22.33	22.12	NA	NA	-0.203
P15260	Interferon gamma receptor 1 OS=Homo sapiens GN=IFNGR1 PE=1 SV=1 - [INGR1_HUMAN]	10.22	1	3	3	4		21.87		23.25		22.08	1	2		21.94		23.01		21.82	21.94	22.41	NA	NA	0.469

Q9BWF3	RNA-binding protein 4 OS=Homo sapiens GN=RBM4 PE=1 SV=1 - [RBM4_HUMAN]	10.16	2	3	3	5		21.75	21.39			21.56	2	1		21.82	21.45			21.30	21.63	21.30	NA	NA	-0.336
P28062	Proteasome subunit beta type-8 OS=Homo sapiens GN=PSMB8 PE=1 SV=3 - [PSB8_HUMAN]	10.14	1	2	2	5				23.82	21.83	23.69	0	3				23.58	22.05	23.43		23.02	0.000	0.000	
Q13190	Syntaxin-5 OS=Homo sapiens GN=STX5 PE=1 SV=2 - [STX5_HUMAN]	10.14	1	3	3	4				22.76	21.84		0	2				22.52	22.06			22.29	NA	NA	
P61165	Transmembrane protein 258 OS=Homo sapiens GN=TMEM258 PE=1 SV=1 - [TM258_HUMAN]	10.13	1	1	1	1						21.59	0	1						21.33		21.33	NA	NA	
P61966	AP-1 complex subunit sigma-1A OS=Homo sapiens GN=AP1S1 PE=1 SV=1 - [AP1S1_HUMAN]	10.13	1	1	1	1						22.99	0	1						22.73		22.73	NA	NA	
Q9BV68	E3 ubiquitin-protein ligase RNF126 OS=Homo sapiens GN=RNF126 PE=1 SV=1 - [RN126_HUMAN]	10.12	1	2	2	5	21.97	24.05			21.12		2	1	22.13	24.12			21.34		23.12	21.34	NA	NA	-1.782
P52823	Stanniocalcin-1 OS=Homo sapiens GN=STC1 PE=1 SV=1 - [STC1_HUMAN]	10.12	1	2	2	6	20.23			23.69	23.33	22.94	1	3	20.39			23.45	23.55	22.68	20.39	23.23	NA	NA	2.837
Q9NTJ3	Structural maintenance of chromosomes protein 4 OS=Homo sapiens GN=SMC4 PE=1 SV=2 - [SMC4_HUMAN]	10.09	1	10	10	17	21.77			22.83	22.69	24.11	1	3	21.94			22.58	22.91	23.85	21.94	23.11	NA	NA	1.177
O95298	NADH dehydrogenase [ubiquinone] 1 subunit C2 OS=Homo sapiens GN=NDUFC2 PE=1 SV=1 - [NDUC2_HUMAN]	10.08	1	1	1	2		21.88	22.08				2	0		21.95	22.14				22.04		NA	NA	
Q99618	Cell division cycle-associated protein 3 OS=Homo sapiens GN=CDCA3 PE=1 SV=1 - [CDCA3_HUMAN]	10.07	1	1	1	3		22.85	22.80				2	0		22.92	22.86				22.89		NA	NA	
Q8N0X7	Spartin OS=Homo sapiens GN=SPG20 PE=1 SV=1 - [SPG20_HUMAN]	10.06	1	3	4	4	24.12	22.39				24.13	2	1	24.28	22.46				23.87	23.37	23.87	NA	NA	0.503
Q9Y3A6	Transmembrane emp24 domain-containing protein 5 OS=Homo sapiens GN=TMED5 PE=1 SV=1 - [TMED5_HUMAN]	10.04	1	1	1	1						23.43	0	1						23.17		23.17	NA	NA	
P56182	Ribosomal RNA processing protein 1 homolog A OS=Homo sapiens GN=RRP1 PE=1 SV=1 - [RRP1_HUMAN]	9.98	1	4	4	6				21.06	20.79	21.77	0	3				20.82	21.01	21.51		21.11	0.000	0.000	
Q86XI2	Condensin-2 complex subunit G2 OS=Homo sapiens GN=NCAPG2 PE=1 SV=1 - [CNDG2_HUMAN]	9.97	1	6	6	13	22.71	22.78	22.21				3	0	22.87	22.85	22.27				22.66		0.000	0.000	
Q9Y5Y2	Cytosolic Fe-S cluster assembly factor NUBP2 OS=Homo sapiens GN=NUBP2 PE=1 SV=1 - [NUBP2_HUMAN]	9.96	1	1	1	2					24.12		0	1					24.34			24.34	NA	NA	
Q96KB5	Lymphokine-activated killer T-cell-originated protein kinase OS=Homo sapiens GN=PBK PE=1 SV=3 - [TOPK_HUMAN]	9.94	1	1	1	4				22.52	23.78	24.09	0	3				22.27	24.00	23.83		23.37	0.000	0.000	
P62699	Protein yippee-like 5 OS=Homo sapiens GN=YPEL5 PE=1 SV=1 - [YPEL5_HUMAN]	9.92	1	1	1	1					21.68		0	1					21.90			21.90	NA	NA	
P25116	Proteinase-activated receptor 1 OS=Homo sapiens GN=F2R PE=1 SV=2 - [PAR1_HUMAN]	9.88	1	3	3	7	21.82	22.32	22.39			21.99	3	1	21.98	22.39	22.44			21.73	22.27	21.73	NA	NA	-0.538
Q16637	Survival motor neuron protein OS=Homo sapiens GN=SMN1 PE=1 SV=1 - [SMN_HUMAN]	9.86	1	2	2	2					22.12		0	1					22.34			22.34	NA	NA	
Q8IV08	Phospholipase D3 OS=Homo sapiens GN=PLD3 PE=1 SV=1 - [PLD3_HUMAN]	9.80	1	4	4	8		22.68		24.00	23.55	23.48	1	3		22.75		23.76	23.77	23.22	22.75	23.58	NA	NA	0.831
Q86X76	Nitrilase homolog 1 OS=Homo sapiens GN=NIT1 PE=1 SV=2 - [NIT1_HUMAN]	9.79	1	1	1	1				23.70			0	1				23.45				23.45	NA	NA	
Q6DKI1	60S ribosomal protein L7-like 1 OS=Homo sapiens GN=RPL7L1 PE=1 SV=1 - [RL7L_HUMAN]	9.76	1	2	2	4				23.11	23.76	23.37	0	3				22.86	23.97	23.11		23.31	0.000	0.000	
Q9H0L4	Cleavage stimulation factor subunit 2 tau variant OS=Homo sapiens GN=CSTF2T PE=1 SV=1 - [CSTFT_HUMAN]	9.74	1	1	4	9				23.30	22.87	22.40	0	3				23.05	23.08	22.14		22.76	0.000	0.000	
Q9UK76	Hematological and neurological expressed 1 protein OS=Homo sapiens GN=HN1 PE=1 SV=3 - [HN1_HUMAN]	9.74	1	1	1	1				23.30			0	1				23.05				23.05	NA	NA	
Q8N5I2	Arrestin domain-containing protein 1 OS=Homo sapiens GN=ARRDC1 PE=1 SV=1 - [ARRD1_HUMAN]	9.70	1	2	2	4	23.85	22.16			21.22		2	1	24.02	22.23			21.44		23.12	21.44	NA	NA	-1.683
O15258	Protein RER1 OS=Homo sapiens GN=RER1 PE=1 SV=1 - [RER1_HUMAN]	9.69	1	1	1	1						22.71	0	1						22.45		22.45	NA	NA	
P04637	Cellular tumor antigen p53 OS=Homo sapiens GN=TP53 PE=1 SV=4 - [P53_HUMAN]	9.67	1	4	4	5					21.22	22.32	0	2					21.44	22.06		21.75	NA	NA	
Q9NQC7	Ubiquitin carboxyl-terminal hydrolase CYLD OS=Homo sapiens GN=CYLD PE=1 SV=1 - [CYLD_HUMAN]	9.62	1	6	6	17	23.30	23.67	23.52				3	0	23.46	23.74	23.58				23.59		0.000	0.000	
O14561	Acyl carrier protein, mitochondrial OS=Homo sapiens GN=NDUFAB1 PE=1 SV=3 - [ACPM_HUMAN]	9.62	1	1	1	1					25.12		0	1					25.33			25.33	NA	NA	
Q9NZM3	Intersectin-2 OS=Homo sapiens GN=ITSN2 PE=1 SV=3 - [ITSN2_HUMAN]	9.61	1	13	14	21	22.82	23.08	23.66				3	0	22.98	23.15	23.72				23.28		0.000	0.000	
Q9P0I2	ER membrane protein complex subunit 3 OS=Homo sapiens GN=EMC3 PE=1 SV=3 - [EMC3_HUMAN]	9.58	1	1	1	1						23.31	0	1						23.05		23.05	NA	NA	
Q8N4T4	Rho guanine nucleotide exchange factor 39 OS=Homo sapiens GN=ARHGEF39 PE=1 SV=1 - [ARG39_HUMAN]	9.55	1	2	2	5		23.23	23.57				2	0		23.30	23.63				23.46		NA	NA	
O75629	Protein CREG1 OS=Homo sapiens GN=CREG1 PE=1 SV=1 - [CREG1_HUMAN]	9.55	1	1	1	1					23.89		0	1					24.10			24.10	NA	NA	
Q99735	Microsomal glutathione S-transferase 2 OS=Homo sapiens GN=MGST2 PE=1 SV=1 - [MGST2_HUMAN]	9.52	1	1	1	4				23.56	22.00	23.80	0	3				23.32	22.21	23.54		23.02	0.000	0.000	
Q6UW68	Transmembrane protein 205 OS=Homo sapiens GN=TMEM205 PE=1 SV=1 - [TM205_HUMAN]	9.52	1	2	2	7			27.67	24.03	23.32	24.52	1	3			27.73	23.78	23.54	24.25	27.73	23.86	NA	NA	-3.874
P51116	Fragile X mental retardation syndrome-related protein 2 OS=Homo sapiens GN=FXR2 PE=1 SV=2 - [FXR2_HUMAN]	9.51	2	3	5	14		21.80		23.50	23.52	23.74	1	3		21.87		23.26	23.74	23.47	21.87	23.49	NA	NA	1.616
Q9BVC6	Transmembrane protein 109 OS=Homo sapiens GN=TMEM109 PE=1 SV=1 - [TM109_HUMAN]	9.47	1	2	2	4				22.47	22.87	23.90	0	3				22.23	23.08	23.64		22.98	0.000	0.000	
Q8NFH3	Nucleoporin Nup43 OS=Homo sapiens GN=NUP43 PE=1 SV=1 - [NUP43_HUMAN]	9.47	1	3	3	4		22.97	22.87		22.68		2	1		23.04	22.93		22.90		22.98	22.90	NA	NA	-0.088
Q16772	Glutathione S-transferase A3 OS=Homo sapiens GN=GSTA3 PE=1 SV=3 - [GSTA3_HUMAN]	9.46	1	1	1	1						23.23	0	1						22.97		22.97	NA	NA	
P53041	Serine/threonine-protein phosphatase 5 OS=Homo sapiens GN=PPP5C PE=1 SV=1 - [PPP5_HUMAN]	9.42	1	4	4	8	21.99			23.19	22.27	22.87	1	3	22.15			22.94	22.48	22.61	22.15	22.68	NA	NA	0.530
O75643	U5 small nuclear ribonucleoprotein 200 kDa helicase OS=Homo sapiens GN=SNRNP200 PE=1 SV=2 - [U520_HUMAN]	9.41	1	11	11	21				23.74	23.45	23.15	0	3				23.50	23.67	22.89		23.35	0.000	0.000	
Q8WUA8	Tsukushin OS=Homo sapiens GN=TSKU PE=2 SV=3 - [TSK_HUMAN]	9.35	1	1	1	3				23.31			0	1				23.07				23.07	NA	NA	
P47974	Zinc finger protein 36, C3H1 type-like 2 OS=Homo sapiens GN=ZFP36L2 PE=1 SV=3 - [TISD_HUMAN]	9.31	2	3	3	6		22.78	22.79				2	0		22.85	22.85				22.85		NA	NA	
P23193	Transcription elongation factor A protein 1 OS=Homo sapiens GN=TCEA1 PE=1 SV=2 - [TCEA1_HUMAN]	9.30	1	1	1	2		20.77			21.45		1	1		20.84			21.67		20.84	21.67	NA	NA	0.829
O43708	Maleylacetoacetate isomerase OS=Homo sapiens GN=GSTZ1 PE=1 SV=3 - [MAAI_HUMAN]	9.26	1	1	1	6				22.96	22.52	23.11	0	3				22.72	22.74	22.85		22.77	0.000	0.000	
Q9GZP0	Platelet-derived growth factor D OS=Homo sapiens GN=PDGFD PE=1 SV=1 - [PDGFD_HUMAN]	9.19	1	3	3	4				21.27	22.62		0	2				21.02	22.83			21.93	NA	NA	
Q96A57	Transmembrane protein 230 OS=Homo sapiens GN=TMEM230 PE=1 SV=1 - [TM230_HUMAN]	9.17	1	1	1	2	21.42			23.27			1	1	21.58			23.02			21.58	23.02	NA	NA	1.440
Q9H098	Protein FAM107B OS=Homo sapiens GN=FAM107B PE=1 SV=1 - [F107B_HUMAN]	9.16	1	1	1	1						18.35	0	1						18.09		18.09	NA	NA	
Q6UWP8	Suprabasin OS=Homo sapiens GN=SBSN PE=1 SV=2 - [SBSN_HUMAN]	9.15	1	1	1	3	20.08	19.61		20.42			2	1	20.24	19.68		20.17			19.96	20.17	NA	NA	0.213
O95861	3'(2'),5'-bisphosphate nucleotidase 1 OS=Homo sapiens GN=BPNT1 PE=1 SV=1 - [BPNT1_HUMAN]	9.09	1	2	2	3				22.12		23.61	0	2				21.88		23.35		22.62	NA	NA	
P0DI83	Ras-related protein Rab-34, isoform NARR OS=Homo sapiens GN=RAB34 PE=1 SV=1 - [NARR_HUMAN]	9.09	1	1	1	1		21.51					1	0		21.58					21.58		NA	NA	
Q13724	Mannosyl-oligosaccharide glucosidase OS=Homo sapiens GN=MOGS PE=1 SV=5 - [MOGS_HUMAN]	9.08	1	5	5	5						22.97	0	1						22.71		22.71	NA	NA	
Q9GZT6	Coiled-coil domain-containing protein 90B, mitochondrial OS=Homo sapiens GN=CCDC90B PE=1 SV=2 - [CC90B_HUMAN]	9.06	1	1	1	1					21.50		0	1					21.72			21.72	NA	NA	
P07093	Glia-derived nexin OS=Homo sapiens GN=SERPINE2 PE=1 SV=1 - [GDN_HUMAN]	9.05	1	3	3	4				22.05	21.53		0	2				21.81	21.75			21.78	NA	NA	
Q9HCN8	Stromal cell-derived factor 2-like protein 1 OS=Homo sapiens GN=SDF2L1 PE=1 SV=2 - [SDF2L_HUMAN]	9.05	1	1	1	2					23.72	23.70	0	2					23.93	23.43		23.68	NA	NA	
O43402	ER membrane protein complex subunit 8 OS=Homo sapiens GN=EMC8 PE=1 SV=1 - [EMC8_HUMAN]	9.05	1	2	2	2					21.25	22.58	0	2					21.47	22.32		21.89	NA	NA	
P34810	Macrosialin OS=Homo sapiens GN=CD68 PE=1 SV=2 - [CD68_HUMAN]	9.04	1	1	1	1						24.22	0	1						23.96		23.96	NA	NA	
P42167	Lamina-associated polypeptide 2, isoforms beta/gamma OS=Homo sapiens GN=TMPO PE=1 SV=2 - [LAP2B_HUMAN]	9.03	1	1	3	3					21.77	23.03	0	2					21.98	22.77		22.38	NA	NA	
Q9UI09	NADH dehydrogenase [ubiquinone] 1 alpha subcomplex subunit 12 OS=Homo sapiens GN=NDUFA12 PE=1 SV=1 - [NDUAC_HUMAN]	8.97	1	1	1	1					20.98		0	1					21.20			21.20	NA	NA	
Q9NXV2	BTB/POZ domain-containing protein KCTD5 OS=Homo sapiens GN=KCTD5 PE=1 SV=1 - [KCTD5_HUMAN]	8.97	1	1	1	1					20.57		0	1					20.78			20.78	NA	NA	
P57076	UPF0769 protein C21orf59 OS=Homo sapiens GN=C21orf59 PE=1 SV=1 - [CU059_HUMAN]	8.97	1	1	1	2					23.19	23.40	0	2					23.41	23.14		23.27	NA	NA	
Q8TCD5	5'(3')-deoxyribonucleotidase, cytosolic type OS=Homo sapiens GN=NT5C PE=1 SV=2 - [NT5C_HUMAN]	8.96	1	1	1	1	21.41						1	0	21.57						21.57		NA	NA	
Q9P287	BRCA2 and CDKN1A-interacting protein OS=Homo sapiens GN=BCCIP PE=1 SV=1 - [BCCIP_HUMAN]	8.92	1	3	3	4				22.83	22.41		0	2				22.58	22.63			22.61	NA	NA	
Q9UI12	V-type proton ATPase subunit H OS=Homo sapiens GN=ATP6V1H PE=1 SV=1 - [VATH_HUMAN]	8.90	1	3	3	5	21.36		20.70		20.72	21.36	2	2	21.52		20.76		20.94	21.10	21.14	21.02	0.784	0.237	-0.121
O76095	Protein JTB OS=Homo sapiens GN=JTB PE=1 SV=1 - [JTB_HUMAN]	8.90	1	1	1	1				23.00			0	1				22.75				22.75	NA	NA	
Q14353	Guanidinoacetate N-methyltransferase OS=Homo sapiens GN=GAMT PE=1 SV=1 - [GAMT_HUMAN]	8.90	1	1	1	1			23.09				1	0			23.15				23.15		NA	NA	
Q8TB69	Zinc finger protein 519 OS=Homo sapiens GN=ZNF519 PE=2 SV=2 - [ZN519_HUMAN]	8.89	8	1	1	1						21.42	0	1						21.15		21.15	NA	NA	
O75380	NADH dehydrogenase [ubiquinone] iron-sulfur protein 6, mitochondrial OS=Homo sapiens GN=NDUFS6 PE=1 SV=1 - [NDUS6_HUMAN]	8.87	1	1	1	1					22.94		0	1					23.16			23.16	NA	NA	
Q9HAU4	E3 ubiquitin-protein ligase SMURF2 OS=Homo sapiens GN=SMURF2 PE=1 SV=1 - [SMUF2_HUMAN]	8.82	1	6	6	23	22.87	23.43	23.32			21.32	3	1	23.03	23.50	23.38			21.06	23.30	21.06	NA	NA	-2.243
P84157	Matrix-remodeling-associated protein 7 OS=Homo sapiens GN=MXRA7 PE=1 SV=1 - [MXRA7_HUMAN]	8.82	1	1	1	1						22.27	0	1						22.01		22.01	NA	NA	
P61073	C-X-C chemokine receptor type 4 OS=Homo sapiens GN=CXCR4 PE=1 SV=1 - [CXCR4_HUMAN]	8.81	1	2	2	5	21.95	22.06		22.29		21.85	2	2	22.11	22.13		22.05		21.59	22.12	21.82	0.323	0.119	-0.297
Q9UKD2	mRNA turnover protein 4 homolog OS=Homo sapiens GN=MRTO4 PE=1 SV=2 - [MRT4_HUMAN]	8.79	1	2	2	4				23.29	22.88	23.16	0	3				23.04	23.10	22.90		23.01	0.000	0.000	
Q9BYN8	28S ribosomal protein S26, mitochondrial OS=Homo sapiens GN=MRPS26 PE=1 SV=1 - [RT26_HUMAN]	8.78	1	1	1	1					21.37		0	1					21.59			21.59	NA	NA	
Q8N5M4	Tetratricopeptide repeat protein 9C OS=Homo sapiens GN=TTC9C PE=1 SV=1 - [TTC9C_HUMAN]	8.77	1	1	1	3				23.80	23.79	23.97	0	3				23.56	24.00	23.70		23.76	0.000	0.000	
Q13098	COP9 signalosome complex subunit 1 OS=Homo sapiens GN=GPS1 PE=1 SV=4 - [CSN1_HUMAN]	8.76	1	3	3	4				22.87		23.60	0	2				22.62		23.34		22.98	NA	NA	
P28068	HLA class II histocompatibility antigen, DM beta chain OS=Homo sapiens GN=HLA-DMB PE=1 SV=1 - [DMB_HUMAN]	8.75	1	1	1	1						23.07	0	1						22.81		22.81	NA	NA	
P33991	DNA replication licensing factor MCM4 OS=Homo sapiens GN=MCM4 PE=1 SV=5 - [MCM4_HUMAN]	8.69	1	5	5	10	20.99			22.55	22.95	23.42	1	3	21.15			22.31	23.16	23.16	21.15	22.88	NA	NA	1.731
Q9Y446	Plakophilin-3 OS=Homo sapiens GN=PKP3 PE=1 SV=1 - [PKP3_HUMAN]	8.66	1	7	7	11	22.38	22.51	22.37				3	0	22.54	22.58	22.43				22.52		0.000	0.000	
Q86X29	Lipolysis-stimulated lipoprotein receptor OS=Homo sapiens GN=LSR PE=1 SV=4 - [LSR_HUMAN]	8.63	1	5	5	9				23.04	20.15	22.56	0	3				22.80	20.37	22.30		21.82	0.000	0.000	
P30260	Cell division cycle protein 27 homolog OS=Homo sapiens GN=CDC27 PE=1 SV=2 - [CDC27_HUMAN]	8.62	1	3	3	7				23.42	22.58	23.09	0	3				23.18	22.80	22.82		22.93	0.000	0.000	
Q9UBV2	Protein sel-1 homolog 1 OS=Homo sapiens GN=SEL1L PE=1 SV=3 - [SE1L1_HUMAN]	8.56	1	3	3	4					22.05	23.33	0	2					22.27	23.07		22.67	NA	NA	
Q99755	Phosphatidylinositol 4-phosphate 5-kinase type-1 alpha OS=Homo sapiens GN=PIP5K1A PE=1 SV=1 - [PI51A_HUMAN]	8.54	1	3	3	3		21.70				23.01	1	1		21.77				22.75	21.77	22.75	NA	NA	0.980
Q9UDY2	Tight junction protein ZO-2 OS=Homo sapiens GN=TJP2 PE=1 SV=2 - [ZO2_HUMAN]	8.49	1	8	8	18				22.81	22.96	22.33	0	3				22.57	23.18	22.07		22.60	0.000	0.000	
Q9NYU2	UDP-glucose:glycoprotein glucosyltransferase 1 OS=Homo sapiens GN=UGGT1 PE=1 SV=3 - [UGGG1_HUMAN]	8.49	1	6	6	14		22.05		22.55	22.31	23.11	1	3		22.12		22.31	22.53	22.85	22.12	22.56	NA	NA	0.440
Q08AM6	Protein VAC14 homolog OS=Homo sapiens GN=VAC14 PE=1 SV=1 - [VAC14_HUMAN]	8.44	1	4	4	8	22.29			23.00	22.03	22.56	1	3	22.45			22.76	22.24	22.30	22.45	22.43	NA	NA	-0.021
Q9NSK0	Kinesin light chain 4 OS=Homo sapiens GN=KLC4 PE=1 SV=3 - [KLC4_HUMAN]	8.40	1	2	3	10	21.48			23.88	22.69	22.80	1	3	21.64			23.64	22.91	22.54	21.64	23.03	NA	NA	1.391
Q8NB90	Spermatogenesis-associated protein 5 OS=Homo sapiens GN=SPATA5 PE=1 SV=3 - [SPAT5_HUMAN]	8.40	1	3	4	13			23.84	24.04	24.02	24.14	1	3			23.90	23.80	24.24	23.88	23.90	23.97	NA	NA	0.074
Q9Y6Y8	SEC23-interacting protein OS=Homo sapiens GN=SEC23IP PE=1 SV=1 - [S23IP_HUMAN]	8.40	1	5	5	12				23.85	23.19	23.90	0	3				23.61	23.41	23.64		23.55	0.000	0.000	
O60568	Procollagen-lysine,2-oxoglutarate 5-dioxygenase 3 OS=Homo sapiens GN=PLOD3 PE=1 SV=1 - [PLOD3_HUMAN]	8.40	1	4	4	5						22.91	0	1						22.65		22.65	NA	NA	
Q9Y3E1	Hepatoma-derived growth factor-related protein 3 OS=Homo sapiens GN=HDGFRP3 PE=1 SV=1 - [HDGR3_HUMAN]	8.37	2	1	1	1				22.47			0	1				22.22				22.22	NA	NA	
P19387	DNA-directed RNA polymerase II subunit RPB3 OS=Homo sapiens GN=POLR2C PE=1 SV=2 - [RPB3_HUMAN]	8.36	1	2	2	7	22.89			23.07	23.35	22.51	1	3	23.05			22.83	23.57	22.25	23.05	22.88	NA	NA	-0.172
P52298	Nuclear cap-binding protein subunit 2 OS=Homo sapiens GN=NCBP2 PE=1 SV=1 - [NCBP2_HUMAN]	8.33	1	1	1	1				21.36			0	1				21.11				21.11	NA	NA	
Q8NBQ5	Estradiol 17-beta-dehydrogenase 11 OS=Homo sapiens GN=HSD17B11 PE=1 SV=3 - [DHB11_HUMAN]	8.33	1	2	2	3					21.03	22.37	0	2					21.25	22.11		21.68	NA	NA	
Q66K74	Microtubule-associated protein 1S OS=Homo sapiens GN=MAP1S PE=1 SV=2 - [MAP1S_HUMAN]	8.31	1	4	4	10	23.32			22.64	23.22	23.61	1	3	23.48			22.40	23.43	23.35	23.48	23.06	NA	NA	-0.417
Q13465	MDS1 and EVI1 complex locus protein MDS1 OS=Homo sapiens GN=MECOM PE=1 SV=1 - [MDS1_HUMAN]	8.28	1	1	1	1				22.49			0	1				22.25				22.25	NA	NA	
Q969X5	Endoplasmic reticulum-Golgi intermediate compartment protein 1 OS=Homo sapiens GN=ERGIC1 PE=1 SV=1 - [ERGI1_HUMAN]	8.28	1	1	1	2		22.59			22.56		1	1		22.66			22.78		22.66	22.78	NA	NA	0.120
P26440	Isovaleryl-CoA dehydrogenase, mitochondrial OS=Homo sapiens GN=IVD PE=1 SV=1 - [IVD_HUMAN]	8.27	1	1	1	1					21.20		0	1					21.41			21.41	NA	NA	
Q96ER9	Coiled-coil domain-containing protein 51 OS=Homo sapiens GN=CCDC51 PE=1 SV=2 - [CCD51_HUMAN]	8.27	1	2	2	2					21.85		0	1					22.07			22.07	NA	NA	
Q15024	Exosome complex component RRP42 OS=Homo sapiens GN=EXOSC7 PE=1 SV=3 - [EXOS7_HUMAN]	8.25	1	1	1	1					21.29		0	1					21.50			21.50	NA	NA	
O94776	Metastasis-associated protein MTA2 OS=Homo sapiens GN=MTA2 PE=1 SV=1 - [MTA2_HUMAN]	8.23	1	2	3	4				22.47	22.80		0	2				22.22	23.02			22.62	NA	NA	
O60763	General vesicular transport factor p115 OS=Homo sapiens GN=USO1 PE=1 SV=2 - [USO1_HUMAN]	8.21	1	4	4	8				23.43	21.90	23.67	0	3				23.18	22.12	23.41		22.90	0.000	0.000	
Q9Y3B2	Exosome complex component CSL4 OS=Homo sapiens GN=EXOSC1 PE=1 SV=1 - [EXOS1_HUMAN]	8.21	1	1	1	3				22.27	22.35	22.85	0	3				22.02	22.57	22.59		22.39	0.000	0.000	
Q96GA7	Serine dehydratase-like OS=Homo sapiens GN=SDSL PE=1 SV=1 - [SDSL_HUMAN]	8.21	1	1	1	1						21.60	0	1						21.34		21.34	NA	NA	
Q96EK9	Protein KTI12 homolog OS=Homo sapiens GN=KTI12 PE=1 SV=1 - [KTI12_HUMAN]	8.19	1	1	1	1					22.43		0	1					22.65			22.65	NA	NA	
P05120	Plasminogen activator inhibitor 2 OS=Homo sapiens GN=SERPINB2 PE=1 SV=2 - [PAI2_HUMAN]	8.19	1	1	1	1					22.03		0	1					22.25			22.25	NA	NA	
P40763	Signal transducer and activator of transcription 3 OS=Homo sapiens GN=STAT3 PE=1 SV=2 - [STAT3_HUMAN]	8.18	1	4	4	9	21.57			22.59	23.06	23.39	1	3	21.73			22.34	23.27	23.13	21.73	22.91	NA	NA	1.185
CON_006959651	SWISS-PROT:Q32PJ2 Bos taurus Apolipoprotein A-IV precursor	8.16	3	2	3	3			21.37			21.52	1	1			21.43			21.26	21.43	21.26	NA	NA	-0.174
Q13049	E3 ubiquitin-protein ligase TRIM32 OS=Homo sapiens GN=TRIM32 PE=1 SV=2 - [TRI32_HUMAN]	8.12	1	3	3	8	22.55	22.55	23.04	21.46			3	1	22.71	22.62	23.10	21.22			22.81	21.22	NA	NA	-1.594
P36639	7,8-dihydro-8-oxoguanine triphosphatase OS=Homo sapiens GN=NUDT1 PE=1 SV=3 - [8ODP_HUMAN]	8.12	1	1	1	1					21.95		0	1					22.16			22.16	NA	NA	
P07477	Trypsin-1 OS=Homo sapiens GN=PRSS1 PE=1 SV=1 - [TRY1_HUMAN]	8.10	3	1	1	4	22.59	25.70	22.83			29.82	3	1	22.76	25.77	22.89			29.56	23.80	29.56	NA	NA	5.755
P23921	Ribonucleoside-diphosphate reductase large subunit OS=Homo sapiens GN=RRM1 PE=1 SV=1 - [RIR1_HUMAN]	8.08	1	3	3	5				22.63	22.23	23.14	0	3				22.39	22.44	22.87		22.57	0.000	0.000	
Q9UGI8	Testin OS=Homo sapiens GN=TES PE=1 SV=1 - [TES_HUMAN]	8.08	1	3	3	4				20.76		21.25	0	2				20.51		20.99		20.75	NA	NA	
Q9NRP0	Oligosaccharyltransferase complex subunit OSTC OS=Homo sapiens GN=OSTC PE=1 SV=1 - [OSTC_HUMAN]	8.05	1	1	1	3		22.78	23.06			24.49	2	1		22.85	23.12			24.23	22.98	24.23	NA	NA	1.250
Q9BQB6	Vitamin K epoxide reductase complex subunit 1 OS=Homo sapiens GN=VKORC1 PE=1 SV=1 - [VKOR1_HUMAN]	7.98	1	1	1	1						22.52	0	1						22.26		22.26	NA	NA	
Q14332	Frizzled-2 OS=Homo sapiens GN=FZD2 PE=1 SV=1 - [FZD2_HUMAN]	7.96	3	5	5	9	22.73	23.23	23.18				3	0	22.89	23.30	23.24				23.14		0.000	0.000	
Q02978	Mitochondrial 2-oxoglutarate/malate carrier protein OS=Homo sapiens GN=SLC25A11 PE=1 SV=3 - [M2OM_HUMAN]	7.96	1	2	2	2						22.30	0	1						22.04		22.04	NA	NA	
Q8IZF2	Adhesion G protein-coupled receptor F5 OS=Homo sapiens GN=ADGRF5 PE=1 SV=3 - [AGRF5_HUMAN]	7.95	1	7	7	17				24.45	24.36	24.31	0	3				24.20	24.57	24.05		24.28	0.000	0.000	
Q13303	Voltage-gated potassium channel subunit beta-2 OS=Homo sapiens GN=KCNAB2 PE=1 SV=2 - [KCAB2_HUMAN]	7.90	1	1	1	1					22.53		0	1					22.75			22.75	NA	NA	
Q9NPH3	Interleukin-1 receptor accessory protein OS=Homo sapiens GN=IL1RAP PE=1 SV=2 - [IL1AP_HUMAN]	7.89	1	2	2	3		22.96	22.58				2	0		23.03	22.63				22.83		NA	NA	
Q96CS3	FAS-associated factor 2 OS=Homo sapiens GN=FAF2 PE=1 SV=2 - [FAF2_HUMAN]	7.87	1	2	2	5		22.38		23.05	23.05	23.42	1	3		22.45		22.81	23.27	23.16	22.45	23.08	NA	NA	0.630
B3EWG3	Protein FAM25A OS=Homo sapiens GN=FAM25A PE=3 SV=1 - [FM25A_HUMAN]	7.87	3	1	1	2					23.54		0	1					23.76			23.76	NA	NA	
Q8WWY3	U4/U6 small nuclear ribonucleoprotein Prp31 OS=Homo sapiens GN=PRPF31 PE=1 SV=2 - [PRP31_HUMAN]	7.82	1	3	3	4				22.73	21.93		0	2				22.49	22.15			22.32	NA	NA	
Q99798	Aconitate hydratase, mitochondrial OS=Homo sapiens GN=ACO2 PE=1 SV=2 - [ACON_HUMAN]	7.82	1	4	4	4					23.34	22.22	0	2					23.55	21.96		22.76	NA	NA	
Q9Y3C5	RING finger protein 11 OS=Homo sapiens GN=RNF11 PE=1 SV=1 - [RNF11_HUMAN]	7.79	1	1	1	3	21.83			22.84		21.82	1	2	21.99			22.59		21.55	21.99	22.07	NA	NA	0.086
Q9NZW5	MAGUK p55 subfamily member 6 OS=Homo sapiens GN=MPP6 PE=1 SV=2 - [MPP6_HUMAN]	7.78	1	3	3	6	22.38		22.07				2	0	22.54		22.13				22.34		NA	NA	
P49023	Paxillin OS=Homo sapiens GN=PXN PE=1 SV=3 - [PAXI_HUMAN]	7.78	1	3	3	7		22.24	22.34	22.96		22.90	2	2		22.31	22.40	22.71		22.64	22.36	22.67	0.033	0.022	0.316

Q12797	Aspartyl/asparaginyl beta-hydroxylase OS=Homo sapiens GN=ASPH PE=1 SV=3 - [ASPH_HUMAN]	7.78	1	3	3	10			18.19		21.17	21.79	1	2			18.25		21.38	21.53	18.25	21.46	NA	NA	3.209
O00330	Pyruvate dehydrogenase protein X component, mitochondrial OS=Homo sapiens GN=PDHX PE=1 SV=3 - [ODPX_HUMAN]	7.78	1	3	3	8					21.82	21.59	0	2					22.04	21.33		21.69	NA	NA	
Q9NZJ9	Diphosphoinositol polyphosphate phosphohydrolase 2 OS=Homo sapiens GN=NUDT4 PE=1 SV=2 - [NUDT4_HUMAN]	7.78	1	1	1	2					21.74	22.48	0	2					21.96	22.22		22.09	NA	NA	
Q96RD7	Pannexin-1 OS=Homo sapiens GN=PANX1 PE=1 SV=4 - [PANX1_HUMAN]	7.75	1	2	2	6				23.40	22.03	23.17	0	3				23.15	22.24	22.91		22.77	0.000	0.000	
Q9UDY4	DnaJ homolog subfamily B member 4 OS=Homo sapiens GN=DNAJB4 PE=1 SV=1 - [DNJB4_HUMAN]	7.72	1	1	2	4	20.53	21.29			24.67	24.67	2	2	20.69	21.36			24.89	24.41	21.02	24.65	0.013	0.012	3.627
P49593	Protein phosphatase 1F OS=Homo sapiens GN=PPM1F PE=1 SV=3 - [PPM1F_HUMAN]	7.71	1	2	2	5				22.15		23.23	0	2				21.91		22.97		22.44	NA	NA	
P55957	BH3-interacting domain death agonist OS=Homo sapiens GN=BID PE=1 SV=1 - [BID_HUMAN]	7.69	1	1	1	1						21.08	0	1						20.82		20.82	NA	NA	
Q9UJU6	Drebrin-like protein OS=Homo sapiens GN=DBNL PE=1 SV=1 - [DBNL_HUMAN]	7.67	1	2	2	3				23.06	22.73		0	2				22.82	22.95			22.88	NA	NA	
Q9UNQ2	Probable dimethyladenosine transferase OS=Homo sapiens GN=DIMT1 PE=1 SV=1 - [DIM1_HUMAN]	7.67	1	1	1	1						21.27	0	1						21.01		21.01	NA	NA	
P04062	Glucosylceramidase OS=Homo sapiens GN=GBA PE=1 SV=3 - [GLCM_HUMAN]	7.65	1	2	2	3				22.71	22.77		0	2				22.46	22.98			22.72	NA	NA	
Q05519	Serine/arginine-rich splicing factor 11 OS=Homo sapiens GN=SRSF11 PE=1 SV=1 - [SRS11_HUMAN]	7.64	1	3	3	6				23.80	21.10	22.69	0	3				23.55	21.31	22.43		22.43	0.000	0.000	
Q4VC31	Coiled-coil domain-containing protein 58 OS=Homo sapiens GN=CCDC58 PE=1 SV=1 - [CCD58_HUMAN]	7.64	1	1	1	1						22.24	0	1						21.98		21.98	NA	NA	
Q04206	Transcription factor p65 OS=Homo sapiens GN=RELA PE=1 SV=2 - [TF65_HUMAN]	7.62	1	4	4	8				21.44	21.26	21.69	0	3				21.19	21.47	21.43		21.36	0.000	0.000	
Q9BU89	Deoxyhypusine hydroxylase OS=Homo sapiens GN=DOHH PE=1 SV=1 - [DOHH_HUMAN]	7.62	1	1	1	2					21.56		0	1					21.78			21.78	NA	NA	
Q13277	Syntaxin-3 OS=Homo sapiens GN=STX3 PE=1 SV=3 - [STX3_HUMAN]	7.61	1	2	2	2		23.15			22.30		1	1		23.22			22.52		23.22	22.52	NA	NA	-0.704
Q9P0V3	SH3 domain-binding protein 4 OS=Homo sapiens GN=SH3BP4 PE=1 SV=1 - [SH3B4_HUMAN]	7.58	1	4	4	4				24.22		21.89	0	2				23.97		21.63		22.80	NA	NA	
Q92696	Geranylgeranyl transferase type-2 subunit alpha OS=Homo sapiens GN=RABGGTA PE=1 SV=2 - [PGTA_HUMAN]	7.58	1	2	2	2			20.88			21.72	1	1			20.94			21.46	20.94	21.46	NA	NA	0.515
P10646	Tissue factor pathway inhibitor OS=Homo sapiens GN=TFPI PE=1 SV=1 - [TFPI1_HUMAN]	7.57	1	2	2	3	23.28	22.88					2	0	23.44	22.95					23.20		NA	NA	
Q15287	RNA-binding protein with serine-rich domain 1 OS=Homo sapiens GN=RNPS1 PE=1 SV=1 - [RNPS1_HUMAN]	7.54	1	2	2	3				23.76	23.78		0	2				23.51	23.99			23.75	NA	NA	
Q8NBJ4	Golgi membrane protein 1 OS=Homo sapiens GN=GOLM1 PE=1 SV=1 - [GOLM1_HUMAN]	7.48	1	3	3	5	22.27	22.49	20.95				3	0	22.43	22.56	21.01				22.00		0.000	0.000	
P08567	Pleckstrin OS=Homo sapiens GN=PLEK PE=1 SV=3 - [PLEK_HUMAN]	7.43	1	1	1	8	22.92	21.82	22.56				3	0	23.08	21.89	22.62				22.53		0.000	0.000	
P54886	Delta-1-pyrroline-5-carboxylate synthase OS=Homo sapiens GN=ALDH18A1 PE=1 SV=2 - [P5CS_HUMAN]	7.42	1	4	4	8				21.46	22.56	23.26	0	3				21.22	22.78	22.99		22.33	0.000	0.000	
P51636	Caveolin-2 OS=Homo sapiens GN=CAV2 PE=1 SV=2 - [CAV2_HUMAN]	7.41	1	1	1	2	21.79	21.81					2	0	21.95	21.88					21.91		NA	NA	
P52888	Thimet oligopeptidase OS=Homo sapiens GN=THOP1 PE=1 SV=2 - [THOP1_HUMAN]	7.40	1	3	3	10	20.55			22.67	21.85	22.98	1	3	20.71			22.42	22.07	22.72	20.71	22.40	NA	NA	1.695
Q96GC9	Vacuole membrane protein 1 OS=Homo sapiens GN=VMP1 PE=1 SV=1 - [VMP1_HUMAN]	7.39	1	1	1	6				23.68	24.20	25.20	0	3				23.44	24.41	24.94		24.26	0.000	0.000	
Q9NW13	RNA-binding protein 28 OS=Homo sapiens GN=RBM28 PE=1 SV=3 - [RBM28_HUMAN]	7.38	1	4	4	9				21.58	21.11	22.16	0	3				21.33	21.33	21.90		21.52	0.000	0.000	
P31146	Coronin-1A OS=Homo sapiens GN=CORO1A PE=1 SV=4 - [COR1A_HUMAN]	7.38	1	1	1	5	23.59		23.58	23.70	22.97		2	2	23.76		23.64	23.46	23.18		23.70	23.32	0.126	0.059	-0.377
Q16204	Coiled-coil domain-containing protein 6 OS=Homo sapiens GN=CCDC6 PE=1 SV=2 - [CCDC6_HUMAN]	7.38	1	4	4	9		20.56		22.14	21.76	22.19	1	3		20.63		21.89	21.97	21.93	20.63	21.93	NA	NA	1.302
P00740	Coagulation factor IX OS=Homo sapiens GN=F9 PE=1 SV=2 - [FA9_HUMAN]	7.38	1	1	1	2			22.50			22.82	1	1			22.56			22.56	22.56	22.56	NA	NA	-0.003
P26358	DNA (cytosine-5)-methyltransferase 1 OS=Homo sapiens GN=DNMT1 PE=1 SV=2 - [DNMT1_HUMAN]	7.36	1	10	10	17		17.79		23.01	24.43	22.56	1	3		17.86		22.76	24.65	22.30	17.86	23.24	NA	NA	5.374
O75439	Mitochondrial-processing peptidase subunit beta OS=Homo sapiens GN=PMPCB PE=1 SV=2 - [MPPB_HUMAN]	7.36	1	1	2	3				20.22	22.28	22.50	0	3				19.97	22.49	22.24		21.57	0.000	0.000	
Q9HD26	Golgi-associated PDZ and coiled-coil motif-containing protein OS=Homo sapiens GN=GOPC PE=1 SV=1 - [GOPC_HUMAN]	7.36	1	2	2	3					22.44	20.52	0	2					22.65	20.26		21.45	NA	NA	
Q9UHA3	Probable ribosome biogenesis protein RLP24 OS=Homo sapiens GN=RSL24D1 PE=1 SV=1 - [RLP24_HUMAN]	7.36	1	1	1	1					23.02		0	1					23.24			23.24	NA	NA	
Q13153	Serine/threonine-protein kinase PAK 1 OS=Homo sapiens GN=PAK1 PE=1 SV=2 - [PAK1_HUMAN]	7.34	2	1	3	8	22.77			23.87	23.59	23.71	1	3	22.93			23.62	23.81	23.45	22.93	23.63	NA	NA	0.693
P23470	Receptor-type tyrosine-protein phosphatase gamma OS=Homo sapiens GN=PTPRG PE=1 SV=4 - [PTPRG_HUMAN]	7.34	1	6	6	10	21.84	22.09	21.55				3	0	22.00	22.16	21.61				21.92		0.000	0.000	
Q13257	Mitotic spindle assembly checkpoint protein MAD2A OS=Homo sapiens GN=MAD2L1 PE=1 SV=1 - [MD2L1_HUMAN]	7.32	1	2	2	2						23.50	0	1						23.24		23.24	NA	NA	
Q7Z7N9	Transmembrane protein 179B OS=Homo sapiens GN=TMEM179B PE=1 SV=1 - [T179B_HUMAN]	7.31	1	1	1	1				23.22			0	1				22.97				22.97	NA	NA	
P39060	Collagen alpha-1(XVIII) chain OS=Homo sapiens GN=COL18A1 PE=1 SV=5 - [COIA1_HUMAN]	7.30	1	7	7	11	24.23	23.03	23.00				3	0	24.39	23.10	23.06				23.52		0.000	0.000	
Q07812	Apoptosis regulator BAX OS=Homo sapiens GN=BAX PE=1 SV=1 - [BAX_HUMAN]	7.29	1	1	1	1						22.34	0	1						22.08		22.08	NA	NA	
Q7Z7H8	39S ribosomal protein L10, mitochondrial OS=Homo sapiens GN=MRPL10 PE=1 SV=3 - [RM10_HUMAN]	7.28	1	1	1	2					21.89	22.18	0	2					22.11	21.92		22.01	NA	NA	
Q96JJ7	Protein disulfide-isomerase TMX3 OS=Homo sapiens GN=TMX3 PE=1 SV=2 - [TMX3_HUMAN]	7.27	1	2	2	5			20.29	21.92	21.43	22.22	1	3			20.35	21.67	21.65	21.96	20.35	21.76	NA	NA	1.415
Q9UBR2	Cathepsin Z OS=Homo sapiens GN=CTSZ PE=1 SV=1 - [CATZ_HUMAN]	7.26	1	2	2	2	20.95					22.12	1	1	21.11					21.86	21.11	21.86	NA	NA	0.742
Q9BQ24	Zinc finger FYVE domain-containing protein 21 OS=Homo sapiens GN=ZFYVE21 PE=1 SV=1 - [ZFY21_HUMAN]	7.26	1	1	1	1			22.48				1	0			22.54				22.54		NA	NA	
Q9H8M9	Protein eva-1 homolog A OS=Homo sapiens GN=EVA1A PE=1 SV=1 - [EVA1A_HUMAN]	7.24	1	1	1	5	25.37	26.00	26.15	25.85			3	1	25.53	26.07	26.21	25.60			25.94	25.60	NA	NA	-0.333
Q9P2E9	Ribosome-binding protein 1 OS=Homo sapiens GN=RRBP1 PE=1 SV=4 - [RRBP1_HUMAN]	7.23	1	8	8	13				19.57	22.40	23.41	0	3				19.32	22.61	23.15		21.69	0.000	0.000	
Q9H190	Syntenin-2 OS=Homo sapiens GN=SDCBP2 PE=1 SV=2 - [SDCB2_HUMAN]	7.19	1	2	2	4	22.71	21.74	22.05				3	0	22.87	21.81	22.11				22.27		0.000	0.000	
Q9BXP5	Serrate RNA effector molecule homolog OS=Homo sapiens GN=SRRT PE=1 SV=1 - [SRRT_HUMAN]	7.19	1	4	4	11				22.98	23.42	23.41	0	3				22.73	23.64	23.15		23.17	0.000	0.000	
Q86U90	YrdC domain-containing protein, mitochondrial OS=Homo sapiens GN=YRDC PE=1 SV=1 - [YRDC_HUMAN]	7.17	1	1	1	1					22.82		0	1					23.04			23.04	NA	NA	
Q9UJW0	Dynactin subunit 4 OS=Homo sapiens GN=DCTN4 PE=1 SV=1 - [DCTN4_HUMAN]	7.17	1	1	1	1						23.73	0	1						23.47		23.47	NA	NA	
Q9NPJ3	Acyl-coenzyme A thioesterase 13 OS=Homo sapiens GN=ACOT13 PE=1 SV=1 - [ACO13_HUMAN]	7.14	1	1	1	1						21.50	0	1						21.24		21.24	NA	NA	
Q13617	Cullin-2 OS=Homo sapiens GN=CUL2 PE=1 SV=2 - [CUL2_HUMAN]	7.11	1	3	4	11		22.07		23.59	22.37	23.33	1	3		22.14		23.34	22.59	23.07	22.14	23.00	NA	NA	0.862
P55083	Microfibril-associated glycoprotein 4 OS=Homo sapiens GN=MFAP4 PE=1 SV=2 - [MFAP4_HUMAN]	7.06	1	1	1	2				21.97	23.06		0	2				21.72	23.27			22.50	NA	NA	
Q9UM54	Unconventional myosin-VI OS=Homo sapiens GN=MYO6 PE=1 SV=4 - [MYO6_HUMAN]	7.03	1	5	5	10				22.04	22.06	23.33	0	3				21.80	22.28	23.07		22.38	0.000	0.000	
Q9H7E9	UPF0488 protein C8orf33 OS=Homo sapiens GN=C8orf33 PE=1 SV=1 - [CH033_HUMAN]	6.99	1	1	1	1				22.09			0	1				21.84				21.84	NA	NA	
P54289	Voltage-dependent calcium channel subunit alpha-2/delta-1 OS=Homo sapiens GN=CACNA2D1 PE=1 SV=3 - [CA2D1_HUMAN]	6.98	1	5	5	9	22.33	23.04	22.17				3	0	22.49	23.10	22.23				22.61		0.000	0.000	
P34741	Syndecan-2 OS=Homo sapiens GN=SDC2 PE=1 SV=2 - [SDC2_HUMAN]	6.97	1	1	1	4	23.84	23.44	23.86				3	0	24.00	23.51	23.92				23.81		0.000	0.000	
Q15125	3-beta-hydroxysteroid-Delta(8),Delta(7)-isomerase OS=Homo sapiens GN=EBP PE=1 SV=3 - [EBP_HUMAN]	6.96	1	1	1	1		24.22					1	0		24.29					24.29		NA	NA	
Q8NDC0	MAPK-interacting and spindle-stabilizing protein-like OS=Homo sapiens GN=MAPK1IP1L PE=1 SV=4 - [MISSL_HUMAN]	6.94	1	1	1	5			25.25	25.06	24.36	24.46	1	3			25.31	24.82	24.58	24.20	25.31	24.53	NA	NA	-0.775
Q03252	Lamin-B2 OS=Homo sapiens GN=LMNB2 PE=1 SV=4 - [LMNB2_HUMAN]	6.94	1	2	5	7				22.31	22.98	21.94	0	3				22.07	23.20	21.68		22.31	0.000	0.000	
P41223	Protein BUD31 homolog OS=Homo sapiens GN=BUD31 PE=1 SV=2 - [BUD31_HUMAN]	6.94	1	1	1	1		20.29					1	0		20.36					20.36		NA	NA	
P82663	28S ribosomal protein S25, mitochondrial OS=Homo sapiens GN=MRPS25 PE=1 SV=1 - [RT25_HUMAN]	6.94	1	1	1	2					23.13	23.24	0	2					23.35	22.97		23.16	NA	NA	
Q9Y6C9	Mitochondrial carrier homolog 2 OS=Homo sapiens GN=MTCH2 PE=1 SV=1 - [MTCH2_HUMAN]	6.93	1	1	1	3		21.04				23.19	1	1		21.11				22.92	21.11	22.92	NA	NA	1.815
Q9GZS3	WD repeat-containing protein 61 OS=Homo sapiens GN=WDR61 PE=1 SV=1 - [WDR61_HUMAN]	6.89	1	1	1	1	21.47						1	0	21.63						21.63		NA	NA	
Q00653	Nuclear factor NF-kappa-B p100 subunit OS=Homo sapiens GN=NFKB2 PE=1 SV=4 - [NFKB2_HUMAN]	6.89	1	3	3	4				21.26		23.19	0	2				21.02		22.92		21.97	NA	NA	
P49756	RNA-binding protein 25 OS=Homo sapiens GN=RBM25 PE=1 SV=3 - [RBM25_HUMAN]	6.88	1	3	3	3					22.83		0	1					23.04			23.04	NA	NA	
Q86U42	Polyadenylate-binding protein 2 OS=Homo sapiens GN=PABPN1 PE=1 SV=3 - [PABP2_HUMAN]	6.86	1	2	2	3				19.91	19.92		0	2				19.67	20.14			19.90	NA	NA	
Q9NRW3	DNA dC->dU-editing enzyme APOBEC-3C OS=Homo sapiens GN=APOBEC3C PE=1 SV=2 - [ABC3C_HUMAN]	6.84	1	1	1	3	20.33	20.53				22.07	2	1	20.49	20.60				21.81	20.55	21.81	NA	NA	1.266
P04183	Thymidine kinase, cytosolic OS=Homo sapiens GN=TK1 PE=1 SV=2 - [KITH_HUMAN]	6.84	1	1	1	1						22.85	0	1						22.59		22.59	NA	NA	
Q03426	Mevalonate kinase OS=Homo sapiens GN=MVK PE=1 SV=1 - [KIME_HUMAN]	6.82	1	1	1	1					22.46		0	1					22.68			22.68	NA	NA	
P48723	Heat shock 70 kDa protein 13 OS=Homo sapiens GN=HSPA13 PE=1 SV=1 - [HSP13_HUMAN]	6.79	1	2	2	2					22.78		0	1					23.00			23.00	NA	NA	
P51398	28S ribosomal protein S29, mitochondrial OS=Homo sapiens GN=DAP3 PE=1 SV=1 - [RT29_HUMAN]	6.78	1	2	2	3	22.98	22.89			20.90		2	1	23.14	22.96			21.12		23.05	21.12	NA	NA	-1.929
Q9BQ52	Zinc phosphodiesterase ELAC protein 2 OS=Homo sapiens GN=ELAC2 PE=1 SV=2 - [RNZ2_HUMAN]	6.78	1	3	3	3					23.23		0	1					23.44			23.44	NA	NA	
P61201	COP9 signalosome complex subunit 2 OS=Homo sapiens GN=COPS2 PE=1 SV=1 - [CSN2_HUMAN]	6.77	1	3	3	6				21.84	21.05	21.99	0	3				21.59	21.27	21.73		21.53	0.000	0.000	
P43897	Elongation factor Ts, mitochondrial OS=Homo sapiens GN=TSFM PE=1 SV=2 - [EFTS_HUMAN]	6.77	1	1	1	1						22.71	0	1						22.45		22.45	NA	NA	
CON_P02663	Alpha-S2-casein OS=Bos taurus GN=CSN1S2 PE=1 SV=2 - [CASA2_BOVIN]	6.76	1	2	2	2	23.48						1	0	23.64						23.64		NA	NA	
P52701	DNA mismatch repair protein Msh6 OS=Homo sapiens GN=MSH6 PE=1 SV=2 - [MSH6_HUMAN]	6.76	1	6	6	8				21.70	22.38	22.61	0	3				21.45	22.60	22.35		22.13	0.000	0.000	
Q6NYC1	Bifunctional arginine demethylase and lysyl-hydroxylase JMJD6 OS=Homo sapiens GN=JMJD6 PE=1 SV=1 - [JMJD6_HUMAN]	6.70	1	2	2	2					21.91	21.87	0	2					22.13	21.60		21.87	NA	NA	
P33993	DNA replication licensing factor MCM7 OS=Homo sapiens GN=MCM7 PE=1 SV=4 - [MCM7_HUMAN]	6.68	1	3	3	7				22.51	23.28	23.09	0	3				22.27	23.50	22.83		22.86	0.000	0.000	
Q96H79	Zinc finger CCCH-type antiviral protein 1-like OS=Homo sapiens GN=ZC3HAV1L PE=1 SV=2 - [ZCCHL_HUMAN]	6.67	1	1	1	2	21.43		20.18				2	0	21.59		20.24				20.92		NA	NA	
Q9H2P9	Diphthine methyl ester synthase OS=Homo sapiens GN=DPH5 PE=1 SV=2 - [DPH5_HUMAN]	6.67	1	1	1	2					21.46		0	1					21.67			21.67	NA	NA	
P46087	Probable 28S rRNA (cytosine(4447)-C(5))-methyltransferase OS=Homo sapiens GN=NOP2 PE=1 SV=2 - [NOP2_HUMAN]	6.65	1	4	4	6				24.20	23.72	23.04	0	3				23.95	23.93	22.78		23.55	0.000	0.000	
P49768	Presenilin-1 OS=Homo sapiens GN=PSEN1 PE=1 SV=1 - [PSN1_HUMAN]	6.64	2	2	2	4				22.36	20.90	22.66	0	3				22.11	21.11	22.40		21.87	0.000	0.000	
O43615	Mitochondrial import inner membrane translocase subunit TIM44 OS=Homo sapiens GN=TIMM44 PE=1 SV=2 - [TIM44_HUMAN]	6.64	1	2	2	3					23.00	23.31	0	2					23.21	23.05		23.13	NA	NA	
Q8TD06	Anterior gradient protein 3 OS=Homo sapiens GN=AGR3 PE=1 SV=1 - [AGR3_HUMAN]	6.63	2	1	1	2					23.15	23.48	0	2					23.37	23.21		23.29	NA	NA	
Q9UNN5	FAS-associated factor 1 OS=Homo sapiens GN=FAF1 PE=1 SV=2 - [FAF1_HUMAN]	6.62	1	3	3	9	22.55	22.48	22.50				3	0	22.71	22.55	22.56				22.61		0.000	0.000	
Q9BWS9	Chitinase domain-containing protein 1 OS=Homo sapiens GN=CHID1 PE=1 SV=1 - [CHID1_HUMAN]	6.62	1	1	1	2					22.32	22.42	0	2					22.54	22.16		22.35	NA	NA	
O75475	PC4 and SFRS1-interacting protein OS=Homo sapiens GN=PSIP1 PE=1 SV=1 - [PSIP1_HUMAN]	6.60	1	3	3	3		23.42			21.08		1	1		23.49			21.29		23.49	21.29	NA	NA	-2.203
P22059	Oxysterol-binding protein 1 OS=Homo sapiens GN=OSBP PE=1 SV=1 - [OSBP1_HUMAN]	6.57	2	4	4	6						22.55	0	1						22.29		22.29	NA	NA	
P60673	Profilin-3 OS=Homo sapiens GN=PFN3 PE=2 SV=1 - [PROF3_HUMAN]	6.57	1	1	1	1				23.29			0	1				23.04				23.04	NA	NA	
Q9H813	Transmembrane protein 206 OS=Homo sapiens GN=TMEM206 PE=1 SV=1 - [TM206_HUMAN]	6.57	1	1	1	1		23.11					1	0		23.18					23.18		NA	NA	
Q14642	Type I inositol 1,4,5-trisphosphate 5-phosphatase OS=Homo sapiens GN=INPP5A PE=1 SV=1 - [I5P1_HUMAN]	6.55	1	1	1	1		22.19					1	0		22.26					22.26		NA	NA	
P27707	Deoxycytidine kinase OS=Homo sapiens GN=DCK PE=1 SV=1 - [DCK_HUMAN]	6.54	1	2	2	4				22.50	22.25	22.55	0	3				22.26	22.46	22.29		22.34	0.000	0.000	
A6NDG6	Phosphoglycolate phosphatase OS=Homo sapiens GN=PGP PE=1 SV=1 - [PGP_HUMAN]	6.54	1	1	1	1						21.21	0	1						20.95		20.95	NA	NA	
Q5BJD5	Transmembrane protein 41B OS=Homo sapiens GN=TMEM41B PE=1 SV=1 - [TM41B_HUMAN]	6.53	1	1	1	2					21.10	22.80	0	2					21.32	22.54		21.93	NA	NA	
Q9H0X4	Protein ITFG3 OS=Homo sapiens GN=ITFG3 PE=1 SV=1 - [ITFG3_HUMAN]	6.52	1	2	2	3				22.81			0	1				22.56				22.56	NA	NA	
Q99614	Tetratricopeptide repeat protein 1 OS=Homo sapiens GN=TTC1 PE=1 SV=1 - [TTC1_HUMAN]	6.51	1	2	2	4				23.64	22.57	23.74	0	3				23.39	22.78	23.48		23.22	0.000	0.000	
A5PLN9	Trafficking protein particle complex subunit 13 OS=Homo sapiens GN=TRAPPC13 PE=1 SV=2 - [TPC13_HUMAN]	6.47	1	1	1	2				21.03			0	1				20.78				20.78	NA	NA	
O75884	Putative hydrolase RBBP9 OS=Homo sapiens GN=RBBP9 PE=1 SV=2 - [RBBP9_HUMAN]	6.45	1	1	1	1						21.53	0	1						21.27		21.27	NA	NA	
Q14839	Chromodomain-helicase-DNA-binding protein 4 OS=Homo sapiens GN=CHD4 PE=1 SV=2 - [CHD4_HUMAN]	6.43	3	8	8	20		24.98		17.30	22.41	22.18	1	3		25.05		17.06	22.63	21.92	25.05	20.53	NA	NA	-4.511
O00499	Myc box-dependent-interacting protein 1 OS=Homo sapiens GN=BIN1 PE=1 SV=1 - [BIN1_HUMAN]	6.41	1	2	2	2						22.87	0	1						22.61		22.61	NA	NA	
Q9Y316	Protein MEMO1 OS=Homo sapiens GN=MEMO1 PE=1 SV=1 - [MEMO1_HUMAN]	6.40	1	1	1	1	21.84						1	0	22.00						22.00		NA	NA	
Q9NY93	Probable ATP-dependent RNA helicase DDX56 OS=Homo sapiens GN=DDX56 PE=1 SV=1 - [DDX56_HUMAN]	6.40	1	3	3	3				20.05	21.00		0	2				19.80	21.21			20.51	NA	NA	
O00425	Insulin-like growth factor 2 mRNA-binding protein 3 OS=Homo sapiens GN=IGF2BP3 PE=1 SV=2 - [IF2B3_HUMAN]	6.39	1	2	3	9				22.69	22.75	23.16	0	3				22.44	22.96	22.90		22.77	0.000	0.000	
P20774	Mimecan OS=Homo sapiens GN=OGN PE=1 SV=1 - [MIME_HUMAN]	6.38	1	1	1	2	23.29			22.97			1	1	23.45			22.72			23.45	22.72	NA	NA	-0.730
Q9UJ68	Mitochondrial peptide methionine sulfoxide reductase OS=Homo sapiens GN=MSRA PE=1 SV=1 - [MSRA_HUMAN]	6.38	1	1	1	1						22.74	0	1						22.48		22.48	NA	NA	
Q9NXU5	ADP-ribosylation factor-like protein 15 OS=Homo sapiens GN=ARL15 PE=1 SV=1 - [ARL15_HUMAN]	6.37	1	1	1	1	22.34						1	0	22.50						22.50		NA	NA	
P46379	Large proline-rich protein BAG6 OS=Homo sapiens GN=BAG6 PE=1 SV=2 - [BAG6_HUMAN]	6.36	1	5	5	13		21.08		23.19	23.40	23.82	1	3		21.15		22.94	23.61	23.56	21.15	23.37	NA	NA	2.220
O95707	Ribonuclease P protein subunit p29 OS=Homo sapiens GN=POP4 PE=1 SV=2 - [RPP29_HUMAN]	6.36	1	1	1	1				19.98			0	1				19.73				19.73	NA	NA	
Q32NC0	UPF0711 protein C18orf21 OS=Homo sapiens GN=C18orf21 PE=1 SV=1 - [CR021_HUMAN]	6.36	1	1	1	2					17.83		0	1					18.04			18.04	NA	NA	
Q16630	Cleavage and polyadenylation specificity factor subunit 6 OS=Homo sapiens GN=CPSF6 PE=1 SV=2 - [CPSF6_HUMAN]	6.35	1	3	3	6				22.31	22.39	22.77	0	3				22.07	22.61	22.51		22.40	0.000	0.000	
P15169	Carboxypeptidase N catalytic chain OS=Homo sapiens GN=CPN1 PE=1 SV=1 - [CBPN_HUMAN]	6.33	1	2	2	6	24.21	23.37	24.40				3	0	24.38	23.44	24.46				24.09		0.000	0.000	
P98194	Calcium-transporting ATPase type 2C member 1 OS=Homo sapiens GN=ATP2C1 PE=1 SV=3 - [AT2C1_HUMAN]	6.31	1	4	4	4				22.33			0	1				22.08				22.08	NA	NA	
Q32MZ4	Leucine-rich repeat flightless-interacting protein 1 OS=Homo sapiens GN=LRRFIP1 PE=1 SV=2 - [LRRF1_HUMAN]	6.31	2	5	5	10			20.55	21.74	21.18	22.33	1	3			20.61	21.49	21.40	22.07	20.61	21.65	NA	NA	1.045
Q9H6Y2	WD repeat-containing protein 55 OS=Homo sapiens GN=WDR55 PE=1 SV=2 - [WDR55_HUMAN]	6.27	1	1	1	2					22.09		0	1					22.31			22.31	NA	NA	
P49821	NADH dehydrogenase [ubiquinone] flavoprotein 1, mitochondrial OS=Homo sapiens GN=NDUFV1 PE=1 SV=4 - [NDUV1_HUMAN]	6.25	1	2	2	2					21.35	22.51	0	2					21.56	22.25		21.91	NA	NA	
O95562	Vesicle transport protein SFT2B OS=Homo sapiens GN=SFT2D2 PE=1 SV=1 - [SFT2B_HUMAN]	6.25	1	1	1	2					21.78	23.22	0	2					22.00	22.96		22.48	NA	NA	
P01135	Protransforming growth factor alpha OS=Homo sapiens GN=TGFA PE=1 SV=1 - [TGFA_HUMAN]	6.25	1	1	1	1			20.97				1	0			21.03				21.03		NA	NA	
Q92859	Neogenin OS=Homo sapiens GN=NEO1 PE=1 SV=2 - [NEO1_HUMAN]	6.23	1	6	6	11	23.36	23.16	23.05	21.03			3	1	23.53	23.23	23.11	20.79			23.29	20.79	NA	NA	-2.505
Q92615	La-related protein 4B OS=Homo sapiens GN=LARP4B PE=1 SV=3 - [LAR4B_HUMAN]	6.23	1	2	3	5				21.12	22.82	23.20	0	3				20.88	23.04	22.94		22.28	0.000	0.000	
Q9UHQ4	B-cell receptor-associated protein 29 OS=Homo sapiens GN=BCAP29 PE=1 SV=2 - [BAP29_HUMAN]	6.22	1	1	1	2			21.28			21.38	1	1			21.34			21.11	21.34	21.11	NA	NA	-0.227
Q9NX55	Huntingtin-interacting protein K OS=Homo sapiens GN=HYPK PE=1 SV=2 - [HYPK_HUMAN]	6.20	1	1	1	2					22.86	23.03	0	2					23.08	22.77		22.92	NA	NA	
CON_006867021	SWISS-PROT:Q9N2I2 Bos taurus Plasma serine protease inhibitor precursor	6.19	1	1	1	2				24.58	24.54		0	2				24.34	24.76			24.55	NA	NA	
Q6PJF5	Inactive rhomboid protein 2 OS=Homo sapiens GN=RHBDF2 PE=1 SV=2 - [RHDF2_HUMAN]	6.19	1	3	3	7		21.21		21.80		22.47	1	2		21.28		21.56		22.21	21.28	21.88	NA	NA	0.605
Q9Y2Q3	Glutathione S-transferase kappa 1 OS=Homo sapiens GN=GSTK1 PE=1 SV=3 - [GSTK1_HUMAN]	6.19	1	1	1	2					22.51		0	1					22.73			22.73	NA	NA	
O43929	Origin recognition complex subunit 4 OS=Homo sapiens GN=ORC4 PE=1 SV=2 - [ORC4_HUMAN]	6.19	1	1	1	1					20.74		0	1					20.95			20.95	NA	NA	
P61927	60S ribosomal protein L37 OS=Homo sapiens GN=RPL37 PE=1 SV=2 - [RL37_HUMAN]	6.19	1	1	1	2					23.85	24.85	0	2					24.06	24.58		24.32	NA	NA	

Q02809	Procollagen-lysine,2-oxoglutarate 5-dioxygenase 1 OS=Homo sapiens GN=PLOD1 PE=1 SV=2 - [PLOD1_HUMAN]	6.19	1	3	3	3						23.33	0	1						23.07		23.07	NA	NA	
Q9P258	Protein RCC2 OS=Homo sapiens GN=RCC2 PE=1 SV=2 - [RCC2_HUMAN]	6.13	1	2	2	2				22.28	22.80		0	2				22.04	23.02			22.53	NA	NA	
P49247	Ribose-5-phosphate isomerase OS=Homo sapiens GN=RPIA PE=1 SV=3 - [RPIA_HUMAN]	6.11	1	1	1	1				22.89			0	1				22.65				22.65	NA	NA	
O75976	Carboxypeptidase D OS=Homo sapiens GN=CPD PE=1 SV=2 - [CBPD_HUMAN]	6.09	1	5	5	6			16.78	23.37		22.34	1	2			16.84	23.12		22.08	16.84	22.60	NA	NA	5.761
Q15021	Condensin complex subunit 1 OS=Homo sapiens GN=NCAPD2 PE=1 SV=3 - [CND1_HUMAN]	6.07	1	6	6	9				21.82		23.59	0	2				21.57		23.33		22.45	NA	NA	
Q9BTE1	Dynactin subunit 5 OS=Homo sapiens GN=DCTN5 PE=1 SV=1 - [DCTN5_HUMAN]	6.04	1	1	1	1						21.63	0	1						21.37		21.37	NA	NA	
P63272	Transcription elongation factor SPT4 OS=Homo sapiens GN=SUPT4H1 PE=1 SV=1 - [SPT4H_HUMAN]	5.98	1	1	1	1	23.21						1	0	23.37						23.37		NA	NA	
Q96EK6	Glucosamine 6-phosphate N-acetyltransferase OS=Homo sapiens GN=GNPNAT1 PE=1 SV=1 - [GNA1_HUMAN]	5.98	1	1	1	2				22.67		23.45	0	2				22.42		23.19		22.80	NA	NA	
P16930	Fumarylacetoacetase OS=Homo sapiens GN=FAH PE=1 SV=2 - [FAAA_HUMAN]	5.97	1	2	2	2				21.35		22.45	0	2				21.10		22.19		21.65	NA	NA	
O60256	Phosphoribosyl pyrophosphate synthase-associated protein 2 OS=Homo sapiens GN=PRPSAP2 PE=1 SV=1 - [KPRB_HUMAN]	5.96	2	2	2	3	21.99					23.54	1	1	22.15					23.28	22.15	23.28	NA	NA	1.129
Q03169	Tumor necrosis factor alpha-induced protein 2 OS=Homo sapiens GN=TNFAIP2 PE=2 SV=2 - [TNAP2_HUMAN]	5.96	1	2	2	2	22.70						1	0	22.86						22.86		NA	NA	
Q9UMR5	Lysosomal thioesterase PPT2 OS=Homo sapiens GN=PPT2 PE=1 SV=4 - [PPT2_HUMAN]	5.96	1	1	1	1					23.13		0	1					23.35			23.35	NA	NA	
Q96IF1	LIM domain-containing protein ajuba OS=Homo sapiens GN=AJUBA PE=1 SV=1 - [AJUBA_HUMAN]	5.95	1	2	2	5	20.92	22.71	23.11				3	0	21.08	22.78	23.17				22.35		0.000	0.000	
Q8NCG7	Sn1-specific diacylglycerol lipase beta OS=Homo sapiens GN=DAGLB PE=1 SV=2 - [DGLB_HUMAN]	5.95	1	2	2	5		21.83		22.51	21.69		1	2		21.90		22.27	21.91		21.90	22.09	NA	NA	0.189
Q13217	DnaJ homolog subfamily C member 3 OS=Homo sapiens GN=DNAJC3 PE=1 SV=1 - [DNJC3_HUMAN]	5.95	1	1	1	3					23.10	22.91	0	2					23.32	22.65		22.98	NA	NA	
Q3ZAQ7	Vacuolar ATPase assembly integral membrane protein VMA21 OS=Homo sapiens GN=VMA21 PE=1 SV=1 - [VMA21_HUMAN]	5.94	1	1	1	3		20.55		22.28		22.88	1	2		20.62		22.03		22.62	20.62	22.33	NA	NA	1.710
Q9BZZ5	Apoptosis inhibitor 5 OS=Homo sapiens GN=API5 PE=1 SV=3 - [API5_HUMAN]	5.92	1	2	2	3				22.49	21.62	22.05	0	3				22.25	21.84	21.79		21.96	0.000	0.000	
O75886	Signal transducing adapter molecule 2 OS=Homo sapiens GN=STAM2 PE=1 SV=1 - [STAM2_HUMAN]	5.90	1	1	3	7	22.92			25.60	24.31		1	2	23.08			25.35	24.53		23.08	24.94	NA	NA	1.856
P42785	Lysosomal Pro-X carboxypeptidase OS=Homo sapiens GN=PRCP PE=1 SV=1 - [PCP_HUMAN]	5.85	1	1	1	1						22.28	0	1						22.02		22.02	NA	NA	
P23142	Fibulin-1 OS=Homo sapiens GN=FBLN1 PE=1 SV=4 - [FBLN1_HUMAN]	5.83	1	3	3	6	24.10		23.07			24.61	2	1	24.26		23.13			24.34	23.69	24.34	NA	NA	0.650
Q8NEM2	SHC SH2 domain-binding protein 1 OS=Homo sapiens GN=SHCBP1 PE=1 SV=3 - [SHCBP_HUMAN]	5.80	1	3	3	3	21.81		22.27				2	0	21.97		22.33				22.15		NA	NA	
Q9Y5X9	Endothelial lipase OS=Homo sapiens GN=LIPG PE=1 SV=1 - [LIPE_HUMAN]	5.80	1	2	2	2	23.25						1	0	23.41						23.41		NA	NA	
Q06203	Amidophosphoribosyltransferase OS=Homo sapiens GN=PPAT PE=1 SV=1 - [PUR1_HUMAN]	5.80	1	2	2	4		21.00		23.25	22.61	23.25	1	3		21.07		23.01	22.83	22.99	21.07	22.94	NA	NA	1.877
Q92552	28S ribosomal protein S27, mitochondrial OS=Homo sapiens GN=MRPS27 PE=1 SV=3 - [RT27_HUMAN]	5.80	1	1	1	1					20.88		0	1					21.09			21.09	NA	NA	
Q14914	Prostaglandin reductase 1 OS=Homo sapiens GN=PTGR1 PE=1 SV=2 - [PTGR1_HUMAN]	5.78	1	1	1	1		19.66					1	0		19.72					19.72		NA	NA	
O95490	Adhesion G protein-coupled receptor L2 OS=Homo sapiens GN=ADGRL2 PE=1 SV=2 - [AGRL2_HUMAN]	5.76	1	4	4	11	23.34	22.69	22.74				3	0	23.51	22.76	22.80				23.02		0.000	0.000	
Q8NFW8	N-acylneuraminate cytidylyltransferase OS=Homo sapiens GN=CMAS PE=1 SV=2 - [NEUA_HUMAN]	5.76	1	1	1	1					22.19		0	1					22.41			22.41	NA	NA	
Q8NEJ9	Neuroguidin OS=Homo sapiens GN=NGDN PE=1 SV=1 - [NGDN_HUMAN]	5.71	1	1	1	1					22.07		0	1					22.29			22.29	NA	NA	
Q12999	Tetraspanin-31 OS=Homo sapiens GN=TSPAN31 PE=2 SV=1 - [TSN31_HUMAN]	5.71	1	1	1	1						21.51	0	1						21.25		21.25	NA	NA	
Q9NPF4	Probable tRNA N6-adenosine threonylcarbamoyltransferase OS=Homo sapiens GN=OSGEP PE=1 SV=1 - [OSGEP_HUMAN]	5.67	1	2	2	2						24.67	0	1						24.41		24.41	NA	NA	
Q9NP77	RNA polymerase II subunit A C-terminal domain phosphatase SSU72 OS=Homo sapiens GN=SSU72 PE=1 SV=1 - [SSU72_HUMAN]	5.67	1	1	1	1					20.62		0	1					20.83			20.83	NA	NA	
Q9BTW9	Tubulin-specific chaperone D OS=Homo sapiens GN=TBCD PE=1 SV=2 - [TBCD_HUMAN]	5.62	1	4	4	6				21.59	21.17	22.58	0	3				21.35	21.38	22.32		21.68	0.000	0.000	
CON_007060941	SWISS-PROT:P02662| Bos taurus Gene_Symbol=CSN1S1 Alpha-S1-casein	5.61	1	1	1	2				23.93		23.58	0	2				23.69		23.32		23.50	NA	NA	
P33992	DNA replication licensing factor MCM5 OS=Homo sapiens GN=MCM5 PE=1 SV=5 - [MCM5_HUMAN]	5.59	1	2	2	6				22.20	22.64	23.20	0	3				21.95	22.85	22.94		22.58	0.000	0.000	
P41240	Tyrosine-protein kinase CSK OS=Homo sapiens GN=CSK PE=1 SV=1 - [CSK_HUMAN]	5.56	1	2	2	4	21.50					22.27	1	1	21.66					22.01	21.66	22.01	NA	NA	0.349
Q15645	Pachytene checkpoint protein 2 homolog OS=Homo sapiens GN=TRIP13 PE=1 SV=2 - [PCH2_HUMAN]	5.56	1	2	2	2				23.03		22.39	0	2				22.79		22.13		22.46	NA	NA	
Q9HC07	Transmembrane protein 165 OS=Homo sapiens GN=TMEM165 PE=1 SV=1 - [TM165_HUMAN]	5.56	1	1	1	1				23.82			0	1				23.57				23.57	NA	NA	
O95456	Proteasome assembly chaperone 1 OS=Homo sapiens GN=PSMG1 PE=1 SV=1 - [PSMG1_HUMAN]	5.56	1	1	1	1						22.98	0	1						22.72		22.72	NA	NA	
P20936	Ras GTPase-activating protein 1 OS=Homo sapiens GN=RASA1 PE=1 SV=1 - [RASA1_HUMAN]	5.54	1	3	3	4	22.98				22.86	23.03	1	2	23.14				23.08	22.77	23.14	22.93	NA	NA	-0.219
P30740	Leukocyte elastase inhibitor OS=Homo sapiens GN=SERPINB1 PE=1 SV=1 - [ILEU_HUMAN]	5.54	1	2	2	2			21.75			23.07	1	1			21.81			22.81	21.81	22.81	NA	NA	0.999
Q96GQ7	Probable ATP-dependent RNA helicase DDX27 OS=Homo sapiens GN=DDX27 PE=1 SV=2 - [DDX27_HUMAN]	5.53	1	2	2	3					21.86	20.35	0	2					22.07	20.09		21.08	NA	NA	
Q9BRK5	45 kDa calcium-binding protein OS=Homo sapiens GN=SDF4 PE=1 SV=1 - [CAB45_HUMAN]	5.52	1	2	2	3				23.24	20.20		0	2				23.00	20.42			21.71	NA	NA	
P55082	Microfibril-associated glycoprotein 3 OS=Homo sapiens GN=MFAP3 PE=2 SV=1 - [MFAP3_HUMAN]	5.52	1	1	1	1				23.84			0	1				23.59				23.59	NA	NA	
Q96RU3	Formin-binding protein 1 OS=Homo sapiens GN=FNBP1 PE=1 SV=2 - [FNBP1_HUMAN]	5.51	1	3	3	4		23.96		22.95	20.80		1	2		24.03		22.70	21.02		24.03	21.86	NA	NA	-2.172
Q13601	KRR1 small subunit processome component homolog OS=Homo sapiens GN=KRR1 PE=1 SV=4 - [KRR1_HUMAN]	5.51	1	2	2	2					22.70		0	1					22.92			22.92	NA	NA	
Q8NHQ9	ATP-dependent RNA helicase DDX55 OS=Homo sapiens GN=DDX55 PE=1 SV=3 - [DDX55_HUMAN]	5.50	1	2	2	2				22.08			0	1				21.84				21.84	NA	NA	
Q9Y2T2	AP-3 complex subunit mu-1 OS=Homo sapiens GN=AP3M1 PE=1 SV=1 - [AP3M1_HUMAN]	5.50	1	1	1	2					23.54	24.18	0	2					23.76	23.92		23.84	NA	NA	
P55001	Microfibrillar-associated protein 2 OS=Homo sapiens GN=MFAP2 PE=2 SV=1 - [MFAP2_HUMAN]	5.46	1	1	1	1					20.45		0	1					20.67			20.67	NA	NA	
Q9BZK7	F-box-like/WD repeat-containing protein TBL1XR1 OS=Homo sapiens GN=TBL1XR1 PE=1 SV=1 - [TBL1R_HUMAN]	5.45	3	1	1	2						23.00	0	1						22.73		22.73	NA	NA	
O60636	Tetraspanin-2 OS=Homo sapiens GN=TSPAN2 PE=1 SV=2 - [TSN2_HUMAN]	5.43	1	1	1	1		20.71					1	0		20.78					20.78		NA	NA	
Q8IUW3	Spermatogenesis-associated protein 2-like protein OS=Homo sapiens GN=SPATA2L PE=2 SV=1 - [SPA2L_HUMAN]	5.42	1	1	1	1		21.66					1	0		21.73					21.73		NA	NA	
Q13619	Cullin-4A OS=Homo sapiens GN=CUL4A PE=1 SV=3 - [CUL4A_HUMAN]	5.40	1	1	4	5				21.80	20.52	22.81	0	3				21.55	20.73	22.55		21.61	0.000	0.000	
O15554	Intermediate conductance calcium-activated potassium channel protein 4 OS=Homo sapiens GN=KCNN4 PE=1 SV=1 - [KCNN4_HUMAN]	5.39	1	1	1	1	20.17						1	0	20.34						20.34		NA	NA	
O15499	Homeobox protein goosecoid-2 OS=Homo sapiens GN=GSC2 PE=2 SV=1 - [GSC2_HUMAN]	5.37	1	1	1	2	20.58			20.90			1	1	20.74			20.65			20.74	20.65	NA	NA	-0.086
Q14919	Dr1-associated corepressor OS=Homo sapiens GN=DRAP1 PE=1 SV=3 - [NC2A_HUMAN]	5.37	1	1	1	2				22.55	22.16		0	2				22.31	22.38			22.34	NA	NA	
P31937	3-hydroxyisobutyrate dehydrogenase, mitochondrial OS=Homo sapiens GN=HIBADH PE=1 SV=2 - [3HIDH_HUMAN]	5.36	1	1	1	1					20.97		0	1					21.19			21.19	NA	NA	
P49902	Cytosolic purine 5'-nucleotidase OS=Homo sapiens GN=NT5C2 PE=1 SV=1 - [5NTC_HUMAN]	5.35	1	2	2	2					22.23	21.95	0	2					22.45	21.69		22.07	NA	NA	
Q8N8F7	Leucine-rich single-pass membrane protein 1 OS=Homo sapiens GN=LSMEM1 PE=1 SV=1 - [LSME1_HUMAN]	5.34	1	1	1	1			22.08				1	0			22.14				22.14		NA	NA	
P0CG29	Glutathione S-transferase theta-2 OS=Homo sapiens GN=GSTT2 PE=1 SV=1 - [GST2_HUMAN]	5.33	2	1	1	2					21.88	22.55	0	2					22.10	22.29		22.19	NA	NA	
P22352	Glutathione peroxidase 3 OS=Homo sapiens GN=GPX3 PE=1 SV=2 - [GPX3_HUMAN]	5.31	1	1	1	1	22.58						1	0	22.74						22.74		NA	NA	
Q5VYK3	Proteasome-associated protein ECM29 homolog OS=Homo sapiens GN=ECM29 PE=1 SV=2 - [ECM29_HUMAN]	5.31	1	7	7	18		21.28		22.57	21.31	22.20	1	3		21.35		22.32	21.52	21.94	21.35	21.93	NA	NA	0.576
P07858	Cathepsin B OS=Homo sapiens GN=CTSB PE=1 SV=3 - [CATB_HUMAN]	5.31	1	1	1	5				23.00	22.62	23.15	0	3				22.75	22.83	22.89		22.82	0.000	0.000	
Q9NX40	OCIA domain-containing protein 1 OS=Homo sapiens GN=OCIAD1 PE=1 SV=1 - [OCAD1_HUMAN]	5.31	1	1	1	2					21.94	22.09	0	2					22.16	21.83		21.99	NA	NA	
Q5JUR7	Testis-expressed sequence 30 protein OS=Homo sapiens GN=TEX30 PE=2 SV=1 - [TEX30_HUMAN]	5.29	2	1	2	4	22.07			23.71	23.01		1	2	22.24			23.46	23.22		22.24	23.34	NA	NA	1.107
P55011	Solute carrier family 12 member 2 OS=Homo sapiens GN=SLC12A2 PE=1 SV=1 - [S12A2_HUMAN]	5.28	1	5	6	10		21.30		23.77	20.88	23.72	1	3		21.37		23.52	21.09	23.46	21.37	22.69	NA	NA	1.323
Q02218	2-oxoglutarate dehydrogenase, mitochondrial OS=Homo sapiens GN=OGDH PE=1 SV=3 - [ODO1_HUMAN]	5.28	2	4	4	6					22.15	23.00	0	2					22.37	22.73		22.55	NA	NA	
CON_P02668	Kappa-casein OS=Bos taurus GN=CSN3 PE=1 SV=1 - [CASK_BOVIN]	5.26	1	1	1	2	24.47			24.77			1	1	24.63			24.53			24.63	24.53	NA	NA	-0.104
Q2TAY7	WD40 repeat-containing protein SMU1 OS=Homo sapiens GN=SMU1 PE=1 SV=2 - [SMU1_HUMAN]	5.26	1	3	3	5				20.70	21.71	21.10	0	3				20.45	21.93	20.84		21.07	0.000	0.000	
P46977	Dolichyl-diphosphooligosaccharide--protein glycosyltransferase subunit STT3A OS=Homo sapiens GN=STT3A PE=1 SV=2 - [STT3A_HUMAN]	5.25	1	3	3	6						23.29	0	1						23.03		23.03	NA	NA	
Q5JTH9	RRP12-like protein OS=Homo sapiens GN=RRP12 PE=1 SV=2 - [RRP12_HUMAN]	5.24	1	4	4	8					22.93	22.35	0	2					23.15	22.09		22.62	NA	NA	
Q8TCS8	Polyribonucleotide nucleotidyltransferase 1, mitochondrial OS=Homo sapiens GN=PNPT1 PE=1 SV=2 - [PNPT1_HUMAN]	5.24	1	2	2	3						22.19	0	1						21.93		21.93	NA	NA	
Q9Y2A7	Nck-associated protein 1 OS=Homo sapiens GN=NCKAP1 PE=1 SV=1 - [NCKP1_HUMAN]	5.23	1	6	6	8		23.06	22.46			22.73	2	1		23.13	22.52			22.47	22.82	22.47	NA	NA	-0.354
Q96G25	Mediator of RNA polymerase II transcription subunit 8 OS=Homo sapiens GN=MED8 PE=1 SV=2 - [MED8_HUMAN]	5.22	1	1	1	1					19.86		0	1					20.08			20.08	NA	NA	
Q8N163	Cell cycle and apoptosis regulator protein 2 OS=Homo sapiens GN=CCAR2 PE=1 SV=2 - [CCAR2_HUMAN]	5.20	1	4	4	9				22.13	23.66	22.99	0	3				21.88	23.88	22.73		22.83	0.000	0.000	
Q13670	Putative postmeiotic segregation increased 2-like protein 11 OS=Homo sapiens GN=PMS2P11 PE=5 SV=1 - [PM2PB_HUMAN]	5.19	1	1	1	2	21.68			22.51			1	1	21.84			22.26			21.84	22.26	NA	NA	0.422
Q9UPY5	Cystine/glutamate transporter OS=Homo sapiens GN=SLC7A11 PE=1 SV=1 - [XCT_HUMAN]	5.19	1	2	2	5		22.82	22.73	23.47		22.92	2	2		22.89	22.78	23.22		22.66	22.84	22.94	0.751	0.230	0.104
P84022	Mothers against decapentaplegic homolog 3 OS=Homo sapiens GN=SMAD3 PE=1 SV=1 - [SMAD3_HUMAN]	5.18	4	1	2	7	23.07	22.74	22.84			23.54	3	1	23.24	22.81	22.90			23.28	22.98	23.28	NA	NA	0.301
Q9BSQ5	Cerebral cavernous malformations 2 protein OS=Homo sapiens GN=CCM2 PE=1 SV=1 - [CCM2_HUMAN]	5.18	1	1	1	1				21.22			0	1				20.97				20.97	NA	NA	
Q92896	Golgi apparatus protein 1 OS=Homo sapiens GN=GLG1 PE=1 SV=2 - [GSLG1_HUMAN]	5.17	1	4	4	7	21.71	21.79	22.33		20.07		3	1	21.87	21.86	22.39		20.29		22.04	20.29	NA	NA	-1.751
Q14653	Interferon regulatory factor 3 OS=Homo sapiens GN=IRF3 PE=1 SV=1 - [IRF3_HUMAN]	5.15	1	1	1	2				21.50		21.30	0	2				21.26		21.04		21.15	NA	NA	
Q15811	Intersectin-1 OS=Homo sapiens GN=ITSN1 PE=1 SV=3 - [ITSN1_HUMAN]	5.11	1	6	7	11	23.46	23.09	23.47			20.90	3	1	23.62	23.16	23.53			20.64	23.43	20.64	NA	NA	-2.790
Q8WVC0	RNA polymerase-associated protein LEO1 OS=Homo sapiens GN=LEO1 PE=1 SV=1 - [LEO1_HUMAN]	5.11	1	2	2	6				20.14	19.79	20.26	0	3				19.89	20.01	20.00		19.97	0.000	0.000	
O75694	Nuclear pore complex protein Nup155 OS=Homo sapiens GN=NUP155 PE=1 SV=1 - [NU155_HUMAN]	5.10	1	4	4	6			21.57	24.07	22.55	22.30	1	3			21.63	23.82	22.76	22.04	21.63	22.87	NA	NA	1.248
Q9UBW8	COP9 signalosome complex subunit 7a OS=Homo sapiens GN=COPS7A PE=1 SV=1 - [CSN7A_HUMAN]	5.09	1	1	1	2					21.73	22.07	0	2					21.95	21.81		21.88	NA	NA	
Q8NF37	Lysophosphatidylcholine acyltransferase 1 OS=Homo sapiens GN=LPCAT1 PE=1 SV=2 - [PCAT1_HUMAN]	5.06	1	2	2	5	24.17	22.85	24.11		23.56		3	1	24.33	22.92	24.17		23.78		23.81	23.78	NA	NA	-0.028
Q9BTE7	DCN1-like protein 5 OS=Homo sapiens GN=DCUN1D5 PE=1 SV=1 - [DCNL5_HUMAN]	5.06	1	1	1	2					21.37	22.40	0	2					21.59	22.14		21.86	NA	NA	
Q9HAS0	Protein Njmu-R1 OS=Homo sapiens GN=C17orf75 PE=1 SV=2 - [NJMU_HUMAN]	5.05	2	2	2	4	22.32		22.69			23.17	2	1	22.49		22.75			22.91	22.62	22.91	NA	NA	0.288
Q9NQX3	Gephyrin OS=Homo sapiens GN=GPHN PE=1 SV=1 - [GEPH_HUMAN]	5.03	1	1	1	2				21.59	21.76		0	2				21.34	21.98			21.66	NA	NA	
P51159	Ras-related protein Rab-27A OS=Homo sapiens GN=RAB27A PE=1 SV=3 - [RB27A_HUMAN]	4.98	1	1	1	1	21.54						1	0	21.70						21.70		NA	NA	
Q9H0A0	N-acetyltransferase 10 OS=Homo sapiens GN=NAT10 PE=1 SV=2 - [NAT10_HUMAN]	4.98	1	3	3	7			19.79	21.51	22.20	22.60	1	3			19.85	21.26	22.41	22.34	19.85	22.00	NA	NA	2.157
Q9UHI6	Probable ATP-dependent RNA helicase DDX20 OS=Homo sapiens GN=DDX20 PE=1 SV=2 - [DDX20_HUMAN]	4.98	1	2	2	3				22.76			0	1				22.52				22.52	NA	NA	
Q9UI10	Translation initiation factor eIF-2B subunit delta OS=Homo sapiens GN=EIF2B4 PE=1 SV=2 - [EI2BD_HUMAN]	4.97	1	1	1	6				24.47	24.31	24.61	0	3				24.22	24.52	24.34		24.36	0.000	0.000	
P32929	Cystathionine gamma-lyase OS=Homo sapiens GN=CTH PE=1 SV=3 - [CGL_HUMAN]	4.94	1	1	1	6	21.89	22.04	22.16		22.24		3	1	22.06	22.11	22.22		22.46		22.13	22.46	NA	NA	0.331
P04049	RAF proto-oncogene serine/threonine-protein kinase OS=Homo sapiens GN=RAF1 PE=1 SV=1 - [RAF1_HUMAN]	4.94	1	1	1	2	21.05			21.97			1	1	21.22			21.72			21.22	21.72	NA	NA	0.505
Q8TBZ3	WD repeat-containing protein 20 OS=Homo sapiens GN=WDR20 PE=1 SV=2 - [WDR20_HUMAN]	4.92	1	1	1	7	22.41	22.78	21.58		20.52		3	1	22.57	22.84	21.64		20.74		22.35	20.74	NA	NA	-1.614
O75716	Serine/threonine-protein kinase 16 OS=Homo sapiens GN=STK16 PE=1 SV=4 - [STK16_HUMAN]	4.92	1	1	1	2				20.61		21.81	0	2				20.37		21.55		20.96	NA	NA	
Q9BTA0	Protein FAM167B OS=Homo sapiens GN=FAM167B PE=2 SV=2 - [F167B_HUMAN]	4.91	1	1	1	1				20.35			0	1				20.11				20.11	NA	NA	
Q9NR56	Muscleblind-like protein 1 OS=Homo sapiens GN=MBNL1 PE=1 SV=2 - [MBNL1_HUMAN]	4.90	3	2	2	3				21.99	22.70		0	2				21.75	22.92			22.33	NA	NA	
Q96I24	Far upstream element-binding protein 3 OS=Homo sapiens GN=FUBP3 PE=1 SV=2 - [FUBP3_HUMAN]	4.90	1	1	1	3					24.14	23.89	0	2					24.36	23.63		23.99	NA	NA	
Q9UIQ6	Leucyl-cystinyl aminopeptidase OS=Homo sapiens GN=LNPEP PE=1 SV=3 - [LCAP_HUMAN]	4.88	1	3	3	6	22.59		21.67			23.05	2	1	22.75		21.73			22.79	22.24	22.79	NA	NA	0.543
Q9Y5Z4	Heme-binding protein 2 OS=Homo sapiens GN=HEBP2 PE=1 SV=1 - [HEBP2_HUMAN]	4.88	1	1	1	1					20.58		0	1					20.80			20.80	NA	NA	
O60547	GDP-mannose 4,6 dehydratase OS=Homo sapiens GN=GMDS PE=1 SV=1 - [GMDS_HUMAN]	4.84	1	2	2	4				21.87	20.44	21.86	0	3				21.62	20.66	21.60		21.29	0.000	0.000	
Q92485	Acid sphingomyelinase-like phosphodiesterase 3b OS=Homo sapiens GN=SMPDL3B PE=2 SV=2 - [ASM3B_HUMAN]	4.84	1	2	2	4				23.61	22.13	22.84	0	3				23.36	22.35	22.58		22.76	0.000	0.000	
Q3ZCQ8	Mitochondrial import inner membrane translocase subunit TIM50 OS=Homo sapiens GN=TIMM50 PE=1 SV=2 - [TIM50_HUMAN]	4.82	1	1	1	3			20.35			21.64	1	1			20.41			21.38	20.41	21.38	NA	NA	0.964
Q8TEY5	Cyclic AMP-responsive element-binding protein 3-like protein 4 OS=Homo sapiens GN=CREB3L4 PE=1 SV=1 - [CR3L4_HUMAN]	4.81	1	1	1	1	25.30						1	0	25.47						25.47		NA	NA	
O60936	Nucleolar protein 3 OS=Homo sapiens GN=NOL3 PE=1 SV=2 - [NOL3_HUMAN]	4.81	1	1	1	1					20.60		0	1					20.82			20.82	NA	NA	
Q969E2	Secretory carrier-associated membrane protein 4 OS=Homo sapiens GN=SCAMP4 PE=2 SV=1 - [SCAM4_HUMAN]	4.80	1	1	1	4	23.87	24.11		24.85		24.87	2	2	24.03	24.18		24.60		24.61	24.10	24.61	0.022	0.017	0.503
O75569	Interferon-inducible double-stranded RNA-dependent protein kinase activator A OS=Homo sapiens GN=PRKRA PE=1 SV=1 - [PRKRA_HUMAN]	4.79	1	1	1	1						21.82	0	1						21.56		21.56	NA	NA	
Q7LGA3	Heparan sulfate 2-O-sulfotransferase 1 OS=Homo sapiens GN=HS2ST1 PE=1 SV=1 - [HS2ST_HUMAN]	4.78	1	1	1	5	21.24			21.48	21.37	22.18	1	3	21.41			21.23	21.58	21.92	21.41	21.58	NA	NA	0.172
O94966	Ubiquitin carboxyl-terminal hydrolase 19 OS=Homo sapiens GN=USP19 PE=1 SV=2 - [UBP19_HUMAN]	4.78	1	3	3	5					22.45	22.85	0	2					22.66	22.59		22.63	NA	NA	
Q9C0K1	Zinc transporter ZIP8 OS=Homo sapiens GN=SLC39A8 PE=2 SV=1 - [S39A8_HUMAN]	4.78	1	1	1	1						24.34	0	1						24.08		24.08	NA	NA	
Q8NB49	Phospholipid-transporting ATPase IG OS=Homo sapiens GN=ATP11C PE=1 SV=3 - [AT11C_HUMAN]	4.77	1	3	3	4				22.11		22.82	0	2				21.87		22.56		22.21	NA	NA	
Q9NUM4	Transmembrane protein 106B OS=Homo sapiens GN=TMEM106B PE=1 SV=2 - [T106B_HUMAN]	4.74	1	1	1	2				21.37		21.64	0	2				21.13		21.38		21.25	NA	NA	
P23469	Receptor-type tyrosine-protein phosphatase epsilon OS=Homo sapiens GN=PTPRE PE=1 SV=1 - [PTPRE_HUMAN]	4.71	1	1	2	2						23.34	0	1						23.08		23.08	NA	NA	
Q8N3E9	1-phosphatidylinositol 4,5-bisphosphate phosphodiesterase delta-3 OS=Homo sapiens GN=PLCD3 PE=1 SV=3 - [PLCD3_HUMAN]	4.69	1	2	2	4	22.34		22.40				2	0	22.51		22.46				22.48		NA	NA	
P42224	Signal transducer and activator of transcription 1-alpha/beta OS=Homo sapiens GN=STAT1 PE=1 SV=2 - [STAT1_HUMAN]	4.67	1	2	2	6	23.18				23.30	23.69	1	2	23.35				23.52	23.43	23.35	23.48	NA	NA	0.129
Q14116	Interleukin-18 OS=Homo sapiens GN=IL18 PE=1 SV=1 - [IL18_HUMAN]	4.66	1	1	1	1		21.84					1	0		21.91					21.91		NA	NA	
P69849	Nodal modulator 3 OS=Homo sapiens GN=NOMO3 PE=3 SV=2 - [NOMO3_HUMAN]	4.66	3	3	3	10			21.56		22.40	23.03	1	2			21.62		22.61	22.77	21.62	22.69	NA	NA	1.068
Q8WZA9	Immunity-related GTPase family Q protein OS=Homo sapiens GN=IRGQ PE=1 SV=1 - [IRGQ_HUMAN]	4.65	1	2	2	2				21.80		21.48	0	2				21.55		21.22		21.38	NA	NA	
Q96D71	RalBP1-associated Eps domain-containing protein 1 OS=Homo sapiens GN=REPS1 PE=1 SV=3 - [REPS1_HUMAN]	4.65	2	3	3	5			19.59	21.81	19.95	23.14	1	3			19.65	21.56	20.17	22.88	19.65	21.54	NA	NA	1.885
Q712K3	Ubiquitin-conjugating enzyme E2 R2 OS=Homo sapiens GN=UBE2R2 PE=1 SV=1 - [UB2R2_HUMAN]	4.62	1	1	1	2				22.07			0	1				21.83				21.83	NA	NA	
P78536	Disintegrin and metalloproteinase domain-containing protein 17 OS=Homo sapiens GN=ADAM17 PE=1 SV=1 - [ADA17_HUMAN]	4.61	1	3	3	4		21.87	22.52	21.37			2	1		21.94	22.58	21.13			22.26	21.13	NA	NA	-1.131
Q9Y3B9	RRP15-like protein OS=Homo sapiens GN=RRP15 PE=1 SV=2 - [RRP15_HUMAN]	4.61	1	1	1	2					19.71	18.72	0	2					19.92	18.46		19.19	NA	NA	
Q9Y530	O-acetyl-ADP-ribose deacetylase 1 OS=Homo sapiens GN=OARD1 PE=1 SV=2 - [OARD1_HUMAN]	4.61	1	1	1	2					21.17	21.42	0	2					21.38	21.16		21.27	NA	NA	
Q96KG9	N-terminal kinase-like protein OS=Homo sapiens GN=SCYL1 PE=1 SV=1 - [NTKL_HUMAN]	4.58	1	1	1	2		22.51					1	0		22.58					22.58		NA	NA	
P26374	Rab proteins geranylgeranyltransferase component A 2 OS=Homo sapiens GN=CHML PE=1 SV=2 - [RAE2_HUMAN]	4.57	1	1	1	1				20.80			0	1				20.55				20.55	NA	NA	

Q9NVI7	ATPase family AAA domain-containing protein 3A OS=Homo sapiens GN=ATAD3A PE=1 SV=2 - [ATD3A_HUMAN]	4.57	2	3	3	6			21.48		22.22	22.54	1	2			21.54		22.43	22.28	21.54	22.36	NA	NA	0.815
P28065	Proteasome subunit beta type-9 OS=Homo sapiens GN=PSMB9 PE=1 SV=2 - [PSB9_HUMAN]	4.57	1	1	1	1					23.72		0	1					23.93			23.93	NA	NA	
Q71RC2	La-related protein 4 OS=Homo sapiens GN=LARP4 PE=1 SV=3 - [LARP4_HUMAN]	4.56	1	1	2	4					22.15	21.10	0	2					22.37	20.84		21.60	NA	NA	
O60938	Keratocan OS=Homo sapiens GN=KERA PE=1 SV=1 - [KERA_HUMAN]	4.55	1	1	1	1	22.33						1	0	22.50						22.50		NA	NA	
Q53TN4	Cytochrome b reductase 1 OS=Homo sapiens GN=CYBRD1 PE=1 SV=1 - [CYBR1_HUMAN]	4.55	1	1	1	3	22.82		22.80	22.56			2	1	22.99		22.86	22.31			22.92	22.31	NA	NA	-0.608
Q15459	Splicing factor 3A subunit 1 OS=Homo sapiens GN=SF3A1 PE=1 SV=1 - [SF3A1_HUMAN]	4.54	3	4	4	6				23.51	22.85	19.25	0	3				23.27	23.07	18.99		21.77	0.000	0.000	
Q8IYB3	Serine/arginine repetitive matrix protein 1 OS=Homo sapiens GN=SRRM1 PE=1 SV=2 - [SRRM1_HUMAN]	4.54	1	3	3	6				20.59	20.04	21.05	0	3				20.34	20.26	20.79		20.46	0.000	0.000	
Q99717	Mothers against decapentaplegic homolog 5 OS=Homo sapiens GN=SMAD5 PE=1 SV=1 - [SMAD5_HUMAN]	4.52	4	1	2	6	23.63	22.59	22.61			23.54	3	1	23.79	22.66	22.67			23.28	23.04	23.28	NA	NA	0.244
Q6N069	N-alpha-acetyltransferase 16, NatA auxiliary subunit OS=Homo sapiens GN=NAA16 PE=1 SV=2 - [NAA16_HUMAN]	4.51	1	1	4	7				25.05	23.05	23.66	0	3				24.80	23.27	23.40		23.82	0.000	0.000	
P61587	Rho-related GTP-binding protein RhoE OS=Homo sapiens GN=RND3 PE=1 SV=1 - [RND3_HUMAN]	4.51	1	1	1	1			21.77				1	0			21.83				21.83		NA	NA	
Q96MC6	Hippocampus abundant transcript 1 protein OS=Homo sapiens GN=HIAT1 PE=2 SV=2 - [HIAT1_HUMAN]	4.49	1	1	1	1				22.94			0	1				22.69				22.69	NA	NA	
O95202	LETM1 and EF-hand domain-containing protein 1, mitochondrial OS=Homo sapiens GN=LETM1 PE=1 SV=1 - [LETM1_HUMAN]	4.47	1	2	2	5		20.92			23.95	24.28	1	2		20.99			24.17	24.02	20.99	24.09	NA	NA	3.107
Q15036	Sorting nexin-17 OS=Homo sapiens GN=SNX17 PE=1 SV=1 - [SNX17_HUMAN]	4.47	1	1	1	4		21.58		23.54		22.97	1	2		21.65		23.30		22.71	21.65	23.01	NA	NA	1.357
O94906	Pre-mRNA-processing factor 6 OS=Homo sapiens GN=PRPF6 PE=1 SV=1 - [PRP6_HUMAN]	4.46	1	4	4	5					21.14	21.43	0	2					21.35	21.17		21.26	NA	NA	
P30408	Transmembrane 4 L6 family member 1 OS=Homo sapiens GN=TM4SF1 PE=1 SV=1 - [T4S1_HUMAN]	4.46	1	1	1	2			24.11				1	0			24.17				24.17		NA	NA	
Q9BYG3	MKI67 FHA domain-interacting nucleolar phosphoprotein OS=Homo sapiens GN=NIFK PE=1 SV=1 - [MK67I_HUMAN]	4.44	1	1	1	1				21.40			0	1				21.16				21.16	NA	NA	
CON_007041501	SWISS-PROT:P07224 Bos taurus Vitamin K-dependent protein S precursor	4.44	1	2	2	3				22.15	21.13		0	2				21.91	21.35			21.63	NA	NA	
Q9NQ55	Suppressor of SWI4 1 homolog OS=Homo sapiens GN=PPAN PE=2 SV=1 - [SSF1_HUMAN]	4.44	1	2	2	3					22.15	21.46	0	2					22.36	21.20		21.78	NA	NA	
Q8NI36	WD repeat-containing protein 36 OS=Homo sapiens GN=WDR36 PE=1 SV=1 - [WDR36_HUMAN]	4.42	1	2	2	6				21.85	22.21	22.33	0	3				21.60	22.43	22.07		22.03	0.000	0.000	
P00846	ATP synthase subunit a OS=Homo sapiens GN=MT-ATP6 PE=1 SV=1 - [ATP6_HUMAN]	4.42	1	1	1	1						22.96	0	1						22.70		22.70	NA	NA	
Q8ND76	Cyclin-Y OS=Homo sapiens GN=CCNY PE=1 SV=2 - [CCNY_HUMAN]	4.40	1	1	1	3	22.88	22.19	21.88				3	0	23.04	22.26	21.94				22.41		0.000	0.000	
Q8NEB9	Phosphatidylinositol 3-kinase catalytic subunit type 3 OS=Homo sapiens GN=PIK3C3 PE=1 SV=1 - [PK3C3_HUMAN]	4.40	1	2	2	3		21.08		21.86			1	1		21.15		21.61			21.15	21.61	NA	NA	0.463
P32455	Guanylate-binding protein 1 OS=Homo sapiens GN=GBP1 PE=1 SV=2 - [GBP1_HUMAN]	4.39	2	1	1	2	20.77		21.02				2	0	20.93		21.08				21.00		NA	NA	
Q6P3X3	Tetratricopeptide repeat protein 27 OS=Homo sapiens GN=TTC27 PE=1 SV=1 - [TTC27_HUMAN]	4.39	1	3	3	7		22.19		22.55	20.59	22.35	1	3		22.26		22.31	20.81	22.08	22.26	21.73	NA	NA	-0.524
O60271	C-Jun-amino-terminal kinase-interacting protein 4 OS=Homo sapiens GN=SPAG9 PE=1 SV=4 - [JIP4_HUMAN]	4.39	1	5	5	9				22.05	21.59	21.08	0	3				21.81	21.80	20.82		21.48	0.000	0.000	
Q96AQ6	Pre-B-cell leukemia transcription factor-interacting protein 1 OS=Homo sapiens GN=PBXIP1 PE=1 SV=1 - [PBIP1_HUMAN]	4.38	1	3	3	3				20.78	21.82		0	2				20.53	22.03			21.28	NA	NA	
Q99732	Lipopolysaccharide-induced tumor necrosis factor-alpha factor OS=Homo sapiens GN=LITAF PE=1 SV=2 - [LITAF_HUMAN]	4.35	1	1	1	2		22.66	22.41				2	0		22.73	22.47				22.60		NA	NA	
O75525	KH domain-containing, RNA-binding, signal transduction-associated protein 3 OS=Homo sapiens GN=KHDRBS3 PE=1 SV=1 - [KHDR3_HUMAN]	4.34	2	1	2	4				23.06	21.89	22.73	0	3				22.81	22.11	22.47		22.46	0.000	0.000	
Q8N131	Porimin OS=Homo sapiens GN=TMEM123 PE=1 SV=1 - [PORIM_HUMAN]	4.33	1	1	1	1	23.61						1	0	23.78						23.78		NA	NA	
O94925	Glutaminase kidney isoform, mitochondrial OS=Homo sapiens GN=GLS PE=1 SV=1 - [GLSK_HUMAN]	4.33	1	2	2	2					22.02		0	1					22.24			22.24	NA	NA	
Q15075	Early endosome antigen 1 OS=Homo sapiens GN=EEA1 PE=1 SV=2 - [EEA1_HUMAN]	4.32	1	5	5	7	21.40			23.51	21.52	22.06	1	3	21.56			23.27	21.73	21.80	21.56	22.27	NA	NA	0.704
Q86V21	Acetoacetyl-CoA synthetase OS=Homo sapiens GN=AACS PE=1 SV=1 - [AACS_HUMAN]	4.32	1	1	1	3						21.93	0	1						21.67		21.67	NA	NA	
Q96GM8	Target of EGR1 protein 1 OS=Homo sapiens GN=TOE1 PE=1 SV=1 - [TOE1_HUMAN]	4.31	1	1	1	2					22.36		0	1					22.57			22.57	NA	NA	
Q6YN16	Hydroxysteroid dehydrogenase-like protein 2 OS=Homo sapiens GN=HSDL2 PE=1 SV=1 - [HSDL2_HUMAN]	4.31	1	1	1	2					22.95	23.06	0	2					23.17	22.80		22.98	NA	NA	
Q8WV44	E3 ubiquitin-protein ligase TRIM41 OS=Homo sapiens GN=TRIM41 PE=1 SV=3 - [TRI41_HUMAN]	4.29	1	2	2	2	21.89						1	0	22.05						22.05		NA	NA	
O75051	Plexin-A2 OS=Homo sapiens GN=PLXNA2 PE=1 SV=4 - [PLXA2_HUMAN]	4.28	1	1	8	12	21.58	21.73		22.31		22.11	2	2	21.75	21.80		22.06		21.85	21.77	21.96	0.224	0.091	0.185
Q8N1I8	Putative uncharacterized protein encoded by CACTIN-AS1 OS=Homo sapiens GN=CACTIN-AS1 PE=2 SV=1 - [CAAS1_HUMAN]	4.27	1	1	1	1	24.62						1	0	24.78						24.78		NA	NA	
O95721	Synaptosomal-associated protein 29 OS=Homo sapiens GN=SNAP29 PE=1 SV=1 - [SNP29_HUMAN]	4.26	1	1	1	1				21.33			0	1				21.08				21.08	NA	NA	
P08833	Insulin-like growth factor-binding protein 1 OS=Homo sapiens GN=IGFBP1 PE=1 SV=1 - [IBP1_HUMAN]	4.25	1	1	1	1					20.64		0	1					20.86			20.86	NA	NA	
Q9UNK0	Syntaxin-8 OS=Homo sapiens GN=STX8 PE=1 SV=2 - [STX8_HUMAN]	4.24	1	1	1	1				22.44			0	1				22.20				22.20	NA	NA	
Q8WVV9	Heterogeneous nuclear ribonucleoprotein L-like OS=Homo sapiens GN=HNRNPLL PE=1 SV=1 - [HNRLL_HUMAN]	4.24	1	1	2	4		22.20	22.31		23.32		2	1		22.27	22.37		23.54		22.32	23.54	NA	NA	1.222
P48147	Prolyl endopeptidase OS=Homo sapiens GN=PREP PE=1 SV=2 - [PPCE_HUMAN]	4.23	1	2	2	4				23.03		23.26	0	2				22.79		23.00		22.89	NA	NA	
P19075	Tetraspanin-8 OS=Homo sapiens GN=TSPAN8 PE=1 SV=1 - [TSN8_HUMAN]	4.22	1	1	1	3			22.01	22.61		22.02	1	2			22.07	22.36		21.76	22.07	22.06	NA	NA	-0.011
Q9HA64	Ketosamine-3-kinase OS=Homo sapiens GN=FN3KRP PE=1 SV=2 - [KT3K_HUMAN]	4.21	1	1	1	1	21.62						1	0	21.78						21.78		NA	NA	
Q5VTL8	Pre-mRNA-splicing factor 38B OS=Homo sapiens GN=PRPF38B PE=1 SV=1 - [PR38B_HUMAN]	4.21	1	2	2	5				20.47	20.53	21.34	0	3				20.22	20.74	21.08		20.68	0.000	0.000	
Q9NZB2	Constitutive coactivator of PPAR-gamma-like protein 1 OS=Homo sapiens GN=FAM120A PE=1 SV=2 - [F120A_HUMAN]	4.20	1	3	3	4				22.36	21.55	22.00	0	3				22.12	21.76	21.74		21.87	0.000	0.000	
Q9BRP1	Programmed cell death protein 2-like OS=Homo sapiens GN=PDCD2L PE=1 SV=1 - [PDD2L_HUMAN]	4.19	1	1	1	4	20.88	20.57	21.01				3	0	21.04	20.64	21.07				20.92		0.000	0.000	
Q9NQS3	Nectin-3 OS=Homo sapiens GN=NECTIN3 PE=1 SV=1 - [NECT3_HUMAN]	4.19	1	1	1	1		22.45					1	0		22.52					22.52		NA	NA	
Q96PK6	RNA-binding protein 14 OS=Homo sapiens GN=RBM14 PE=1 SV=2 - [RBM14_HUMAN]	4.19	1	2	2	4			22.24		24.36	20.48	1	2			22.30		24.57	20.22	22.30	22.40	NA	NA	0.094
Q9Y653	G-protein coupled receptor 56 OS=Homo sapiens GN=GPR56 PE=1 SV=2 - [GPR56_HUMAN]	4.18	1	2	2	5	21.89	21.13					2	0	22.05	21.20					21.63		NA	NA	
O00461	Golgi integral membrane protein 4 OS=Homo sapiens GN=GOLIM4 PE=1 SV=1 - [GOLI4_HUMAN]	4.17	1	2	2	5	21.29	21.40				19.73	2	1	21.45	21.47				19.47	21.46	19.47	NA	NA	-1.990
Q9NWQ8	Phosphoprotein associated with glycosphingolipid-enriched microdomains 1 OS=Homo sapiens GN=PAG1 PE=1 SV=2 - [PHAG1_HUMAN]	4.17	1	1	1	3				21.50	20.65	21.05	0	3				21.26	20.87	20.79		20.97	0.000	0.000	
Q9NV92	NEDD4 family-interacting protein 2 OS=Homo sapiens GN=NDFIP2 PE=1 SV=2 - [NFIP2_HUMAN]	4.17	1	1	1	2				24.03			0	1				23.78				23.78	NA	NA	
Q6RFH5	WD repeat-containing protein 74 OS=Homo sapiens GN=WDR74 PE=1 SV=1 - [WDR74_HUMAN]	4.16	1	1	1	3				22.19	22.33	22.35	0	3				21.94	22.55	22.09		22.19	0.000	0.000	
Q9NT62	Ubiquitin-like-conjugating enzyme ATG3 OS=Homo sapiens GN=ATG3 PE=1 SV=1 - [ATG3_HUMAN]	4.14	1	1	1	1						22.68	0	1						22.42		22.42	NA	NA	
Q9NQX7	Integral membrane protein 2C OS=Homo sapiens GN=ITM2C PE=1 SV=1 - [ITM2C_HUMAN]	4.12	1	1	1	3				25.02	23.73	23.84	0	3				24.78	23.94	23.57		24.10	0.000	0.000	
Q9ULG6	Cell cycle progression protein 1 OS=Homo sapiens GN=CCPG1 PE=1 SV=3 - [CCPG1_HUMAN]	4.10	1	3	3	4				23.58		20.56	0	2				23.33		20.30		21.82	NA	NA	
P00742	Coagulation factor X OS=Homo sapiens GN=F10 PE=1 SV=2 - [FA10_HUMAN]	4.10	1	2	2	4		23.19		23.67		23.48	1	2		23.26		23.42		23.22	23.26	23.32	NA	NA	0.062
Q9BY44	Eukaryotic translation initiation factor 2A OS=Homo sapiens GN=EIF2A PE=1 SV=3 - [EIF2A_HUMAN]	4.10	1	2	2	3			22.23		21.91		1	1			22.29		22.13		22.29	22.13	NA	NA	-0.163
Q7Z4W1	L-xylulose reductase OS=Homo sapiens GN=DCXR PE=1 SV=2 - [DCXR_HUMAN]	4.10	1	1	1	1			21.48				1	0			21.54				21.54		NA	NA	
O00116	Alkyldihydroxyacetonephosphate synthase, peroxisomal OS=Homo sapiens GN=AGPS PE=1 SV=1 - [ADAS_HUMAN]	4.10	1	2	2	2						22.64	0	1						22.38		22.38	NA	NA	
Q96C23	Aldose 1-epimerase OS=Homo sapiens GN=GALM PE=1 SV=1 - [GALM_HUMAN]	4.09	1	1	1	1				22.66			0	1				22.42				22.42	NA	NA	
Q96T37	Putative RNA-binding protein 15 OS=Homo sapiens GN=RBM15 PE=1 SV=2 - [RBM15_HUMAN]	4.09	1	3	3	4		18.14	18.52		20.28		2	1		18.21	18.57		20.49		18.39	20.49	NA	NA	2.101
O95858	Tetraspanin-15 OS=Homo sapiens GN=TSPAN15 PE=1 SV=1 - [TSN15_HUMAN]	4.08	1	1	1	3	22.91		23.15	23.15			2	1	23.07		23.21	22.90			23.14	22.90	NA	NA	-0.239
Q93009	Ubiquitin carboxyl-terminal hydrolase 7 OS=Homo sapiens GN=USP7 PE=1 SV=2 - [UBP7_HUMAN]	4.08	1	3	3	4				22.93		21.18	0	2				22.69		20.92		21.80	NA	NA	
Q9NPD3	Exosome complex component RRP41 OS=Homo sapiens GN=EXOSC4 PE=1 SV=3 - [EXOS4_HUMAN]	4.08	1	1	1	1					21.11		0	1					21.33			21.33	NA	NA	
P36969	Phospholipid hydroperoxide glutathione peroxidase, mitochondrial OS=Homo sapiens GN=GPX4 PE=1 SV=3 - [GPX4_HUMAN]	4.06	1	1	1	1						23.23	0	1						22.97		22.97	NA	NA	
Q15006	ER membrane protein complex subunit 2 OS=Homo sapiens GN=EMC2 PE=1 SV=1 - [EMC2_HUMAN]	4.04	1	1	1	3					22.44	23.08	0	2					22.65	22.82		22.73	NA	NA	
P40189	Interleukin-6 receptor subunit beta OS=Homo sapiens GN=IL6ST PE=1 SV=2 - [IL6RB_HUMAN]	4.03	1	2	2	3				22.56		22.28	0	2				22.31		22.02		22.17	NA	NA	
P07199	Major centromere autoantigen B OS=Homo sapiens GN=CENPB PE=1 SV=2 - [CENPB_HUMAN]	4.01	1	2	2	4	21.71	22.19	22.39				3	0	21.87	22.26	22.45				22.19		0.000	0.000	
Q8IY22	C-Maf-inducing protein OS=Homo sapiens GN=CMIP PE=1 SV=3 - [CMIP_HUMAN]	4.01	1	1	1	2	21.70		21.22				2	0	21.86		21.28				21.57		NA	NA	
Q9C004	Protein sprouty homolog 4 OS=Homo sapiens GN=SPRY4 PE=1 SV=2 - [SPY4_HUMAN]	4.01	1	1	1	2		23.07	22.64				2	0		23.14	22.70				22.92		NA	NA	
Q14155	Rho guanine nucleotide exchange factor 7 OS=Homo sapiens GN=ARHGEF7 PE=1 SV=2 - [ARHG7_HUMAN]	3.99	1	2	2	4			22.11	22.28		23.75	1	2			22.17	22.03		23.49	22.17	22.76	NA	NA	0.596
P42574	Caspase-3 OS=Homo sapiens GN=CASP3 PE=1 SV=2 - [CASP3_HUMAN]	3.97	1	1	1	2						23.19	0	1						22.93		22.93	NA	NA	
Q86T03	Type 1 phosphatidylinositol 4,5-bisphosphate 4-phosphatase OS=Homo sapiens GN=TMEM55B PE=1 SV=1 - [TM55B_HUMAN]	3.97	1	1	1	3				23.35	22.48	23.28	0	3				23.10	22.69	23.02		22.94	0.000	0.000	
Q9H0S4	Probable ATP-dependent RNA helicase DDX47 OS=Homo sapiens GN=DDX47 PE=1 SV=1 - [DDX47_HUMAN]	3.96	1	1	1	1					23.14		0	1					23.36			23.36	NA	NA	
Q5JTV8	Torsin-1A-interacting protein 1 OS=Homo sapiens GN=TOR1AIP1 PE=1 SV=2 - [TOIP1_HUMAN]	3.95	2	2	2	3						22.73	0	1						22.47		22.47	NA	NA	
Q9NUQ6	SPATS2-like protein OS=Homo sapiens GN=SPATS2L PE=1 SV=2 - [SPS2L_HUMAN]	3.94	1	2	2	5		20.66	20.74	21.89	21.15		2	2		20.73	20.80	21.65	21.36		20.76	21.51	0.036	0.024	0.741
Q9NW15	Anoctamin-10 OS=Homo sapiens GN=ANO10 PE=1 SV=2 - [ANO10_HUMAN]	3.94	1	1	1	1			21.83				1	0			21.89				21.89		NA	NA	
Q13609	Deoxyribonuclease gamma OS=Homo sapiens GN=DNASE1L3 PE=1 SV=1 - [DNSL3_HUMAN]	3.93	1	1	1	2	22.01		22.25				2	0	22.17		22.31				22.24		NA	NA	
Q96A65	Exocyst complex component 4 OS=Homo sapiens GN=EXOC4 PE=1 SV=1 - [EXOC4_HUMAN]	3.90	1	3	3	6					21.12	22.00	0	2					21.34	21.74		21.54	NA	NA	
Q9HCE1	Putative helicase MOV-10 OS=Homo sapiens GN=MOV10 PE=1 SV=2 - [MOV10_HUMAN]	3.89	1	3	3	3	22.20	21.45					2	0	22.36	21.52					21.94		NA	NA	
Q14318	Peptidyl-prolyl cis-trans isomerase FKBP8 OS=Homo sapiens GN=FKBP8 PE=1 SV=2 - [FKBP8_HUMAN]	3.88	1	1	1	1						22.37	0	1						22.11		22.11	NA	NA	
Q9Y676	28S ribosomal protein S18b, mitochondrial OS=Homo sapiens GN=MRPS18B PE=1 SV=1 - [RT18B_HUMAN]	3.88	1	1	1	2						22.54	0	1						22.28		22.28	NA	NA	
Q96I13	Protein ABHD8 OS=Homo sapiens GN=ABHD8 PE=2 SV=1 - [ABHD8_HUMAN]	3.87	1	1	1	4	17.62		17.54	18.65			2	1	17.78		17.60	18.41			17.69	18.41	NA	NA	0.716
O14657	Torsin-1B OS=Homo sapiens GN=TOR1B PE=1 SV=2 - [TOR1B_HUMAN]	3.87	1	1	1	1				22.58			0	1				22.34				22.34	NA	NA	
Q9UJP4	Kelch-like protein 21 OS=Homo sapiens GN=KLHL21 PE=1 SV=4 - [KLH21_HUMAN]	3.85	1	1	1	1	22.55						1	0	22.71						22.71		NA	NA	
O43592	Exportin-T OS=Homo sapiens GN=XPOT PE=1 SV=2 - [XPOT_HUMAN]	3.85	1	1	1	1				21.37			0	1				21.13				21.13	NA	NA	
P48788	Troponin I, fast skeletal muscle OS=Homo sapiens GN=TNNI2 PE=1 SV=2 - [TNNI2_HUMAN]	3.85	2	1	1	2				23.61		23.08	0	2				23.36		22.82		23.09	NA	NA	
Q8WXF5	Gamma-crystallin N OS=Homo sapiens GN=CRYGN PE=2 SV=1 - [CRGN_HUMAN]	3.85	1	1	1	1		27.79					1	0		27.86					27.86		NA	NA	
Q12788	Transducin beta-like protein 3 OS=Homo sapiens GN=TBL3 PE=1 SV=2 - [TBL3_HUMAN]	3.84	1	2	2	2				22.53	21.74		0	2				22.28	21.96			22.12	NA	NA	
Q9H6R4	Nucleolar protein 6 OS=Homo sapiens GN=NOL6 PE=1 SV=2 - [NOL6_HUMAN]	3.84	1	2	2	2					22.14	21.52	0	2					22.36	21.26		21.81	NA	NA	
Q9GZU7	Carboxy-terminal domain RNA polymerase II polypeptide A small phosphatase 1 OS=Homo sapiens GN=CTDSP1 PE=1 SV=1 - [CTDS1_HUMAN]	3.83	2	1	1	3	22.35		21.93				2	0	22.51		21.99				22.25		NA	NA	
Q9Y3Z3	Deoxynucleoside triphosphate triphosphohydrolase SAMHD1 OS=Homo sapiens GN=SAMHD1 PE=1 SV=2 - [SAMH1_HUMAN]	3.83	1	2	2	2					22.13		0	1					22.35			22.35	NA	NA	
Q9P0T7	Transmembrane protein 9 OS=Homo sapiens GN=TMEM9 PE=1 SV=1 - [TMEM9_HUMAN]	3.83	2	1	1	1					22.19		0	1					22.40			22.40	NA	NA	
Q9H3F6	BTB/POZ domain-containing adapter for CUL3-mediated RhoA degradation protein 3 OS=Homo sapiens GN=KCTD10 PE=1 SV=1 - [BACD3_HUMAN]	3.83	1	1	1	1			19.51				1	0			19.57				19.57		NA	NA	
Q96DX4	RING finger and SPRY domain-containing protein 1 OS=Homo sapiens GN=RSPRY1 PE=1 SV=1 - [RSPRY_HUMAN]	3.82	1	1	1	1			22.09				1	0			22.15				22.15		NA	NA	
O15394	Neural cell adhesion molecule 2 OS=Homo sapiens GN=NCAM2 PE=1 SV=2 - [NCAM2_HUMAN]	3.82	1	3	3	3						21.55	0	1						21.29		21.29	NA	NA	
Q8WWK9	Cytoskeleton-associated protein 2 OS=Homo sapiens GN=CKAP2 PE=1 SV=1 - [CKAP2_HUMAN]	3.81	1	2	2	4	21.63	22.22	22.29				3	0	21.79	22.29	22.35				22.14		0.000	0.000	
Q9Y5A9	YTH domain-containing family protein 2 OS=Homo sapiens GN=YTHDF2 PE=1 SV=2 - [YTHD2_HUMAN]	3.80	3	2	2	5			20.39	21.38	21.53	21.78	1	3			20.45	21.14	21.74	21.52	20.45	21.47	NA	NA	1.016
O60216	Double-strand-break repair protein rad21 homolog OS=Homo sapiens GN=RAD21 PE=1 SV=2 - [RAD21_HUMAN]	3.80	1	2	2	2					20.35	20.47	0	2					20.57	20.21		20.39	NA	NA	
Q5VZV1	Protein-lysine methyltransferase METTL21C OS=Homo sapiens GN=METTL21C PE=1 SV=1 - [MT21C_HUMAN]	3.79	1	1	1	1						23.05	0	1						22.79		22.79	NA	NA	
Q9Y289	Sodium-dependent multivitamin transporter OS=Homo sapiens GN=SLC5A6 PE=2 SV=2 - [SC5A6_HUMAN]	3.78	1	2	2	4	23.46	23.61	23.86			23.83	3	1	23.62	23.68	23.92			23.57	23.74	23.57	NA	NA	-0.170
Q8NBN3	Transmembrane protein 87A OS=Homo sapiens GN=TMEM87A PE=1 SV=3 - [TM87A_HUMAN]	3.78	1	1	1	1				23.86			0	1				23.61				23.61	NA	NA	
Q14457	Beclin-1 OS=Homo sapiens GN=BECN1 PE=1 SV=2 - [BECN1_HUMAN]	3.78	1	1	1	2				22.95	21.93		0	2				22.71	22.15			22.43	NA	NA	
P19883	Follistatin OS=Homo sapiens GN=FST PE=1 SV=2 - [FST_HUMAN]	3.78	1	2	2	3				20.99	19.94		0	2				20.75	20.15			20.45	NA	NA	
Q9UJ70	N-acetyl-D-glucosamine kinase OS=Homo sapiens GN=NAGK PE=1 SV=4 - [NAGK_HUMAN]	3.78	1	1	1	2					21.18	22.25	0	2					21.40	21.99		21.69	NA	NA	
P49736	DNA replication licensing factor MCM2 OS=Homo sapiens GN=MCM2 PE=1 SV=4 - [MCM2_HUMAN]	3.76	1	3	3	5				23.11	21.65	23.11	0	3				22.87	21.86	22.85		22.53	0.000	0.000	
Q9Y2L1	Exosome complex exonuclease RRP44 OS=Homo sapiens GN=DIS3 PE=1 SV=2 - [RRP44_HUMAN]	3.76	1	3	3	6				22.43	22.14	22.34	0	3				22.18	22.36	22.08		22.21	0.000	0.000	
Q14790	Caspase-8 OS=Homo sapiens GN=CASP8 PE=1 SV=1 - [CASP8_HUMAN]	3.76	1	2	2	4				21.79	20.37	21.31	0	3				21.54	20.59	21.05		21.06	0.000	0.000	
O75400	Pre-mRNA-processing factor 40 homolog A OS=Homo sapiens GN=PRPF40A PE=1 SV=2 - [PR40A_HUMAN]	3.76	1	3	3	4					21.89	22.01	0	2					22.10	21.75		21.92	NA	NA	
Q8IZ81	ELMO domain-containing protein 2 OS=Homo sapiens GN=ELMOD2 PE=1 SV=1 - [ELMD2_HUMAN]	3.75	1	1	1	2			19.57		19.99		1	1			19.63		20.21		19.63	20.21	NA	NA	0.581
P55212	Caspase-6 OS=Homo sapiens GN=CASP6 PE=1 SV=2 - [CASP6_HUMAN]	3.75	1	1	1	1					19.23		0	1					19.44			19.44	NA	NA	
Q9Y3T9	Nucleolar complex protein 2 homolog OS=Homo sapiens GN=NOC2L PE=1 SV=4 - [NOC2L_HUMAN]	3.74	1	2	2	9			20.53	21.82	21.57	21.98	1	3			20.59	21.57	21.79	21.72	20.59	21.69	NA	NA	1.102
Q9Y223	Bifunctional UDP-N-acetylglucosamine 2-epimerase/N-acetylmannosamine kinase OS=Homo sapiens GN=GNE PE=1 SV=1 - [GLCNE_HUMAN]	3.74	1	2	2	2						22.77	0	1						22.51		22.51	NA	NA	
Q9HCC0	Methylcrotonoyl-CoA carboxylase beta chain, mitochondrial OS=Homo sapiens GN=MCCC2 PE=1 SV=1 - [MCCB_HUMAN]	3.73	1	2	2	2					20.34		0	1					20.56			20.56	NA	NA	
Q9BUU2	Methyltransferase-like protein 22 OS=Homo sapiens GN=METTL22 PE=1 SV=2 - [MET22_HUMAN]	3.71	1	1	1	1						23.67	0	1						23.41		23.41	NA	NA	
Q92990	Glomulin OS=Homo sapiens GN=GLMN PE=1 SV=2 - [GLMN_HUMAN]	3.70	1	2	2	2				20.60			0	1				20.36				20.36	NA	NA	
Q9NY26	Zinc transporter ZIP1 OS=Homo sapiens GN=SLC39A1 PE=1 SV=1 - [S39A1_HUMAN]	3.70	1	1	1	2				24.55		23.07	0	2				24.31		22.81		23.56	NA	NA	
Q03701	CCAAT/enhancer-binding protein zeta OS=Homo sapiens GN=CEBPZ PE=1 SV=3 - [CEBPZ_HUMAN]	3.70	1	2	2	2					22.83	21.47	0	2					23.04	21.21		22.13	NA	NA	
P11717	Cation-independent mannose-6-phosphate receptor OS=Homo sapiens GN=IGF2R PE=1 SV=3 - [MPRI_HUMAN]	3.69	1	6	6	9		21.06		21.95		22.76	1	2		21.13		21.70		22.50	21.13	22.10	NA	NA	0.970
Q9BSR8	Protein YIPF4 OS=Homo sapiens GN=YIPF4 PE=1 SV=1 - [YIPF4_HUMAN]	3.69	1	1	1	1				23.60			0	1				23.36				23.36	NA	NA	
Q92600	Cell differentiation protein RCD1 homolog OS=Homo sapiens GN=RQCD1 PE=1 SV=1 - [RCD1_HUMAN]	3.68	1	1	1	1				20.93			0	1				20.68				20.68	NA	NA	
Q10471	Polypeptide N-acetylgalactosaminyltransferase 2 OS=Homo sapiens GN=GALNT2 PE=1 SV=1 - [GALT2_HUMAN]	3.68	1	2	2	2						22.48	0	1						22.22		22.22	NA	NA	
P48163	NADP-dependent malic enzyme OS=Homo sapiens GN=ME1 PE=1 SV=1 - [MAOX_HUMAN]	3.67	1	1	1	5	21.56	21.49		21.97		22.47	2	2	21.72	21.56		21.73		22.21	21.64	21.97	0.321	0.119	0.331
P17302	Gap junction alpha-1 protein OS=Homo sapiens GN=GJA1 PE=1 SV=2 - [CXA1_HUMAN]	3.66	1	1	1	2				23.74		22.65	0	2				23.50		22.39		22.95	NA	NA	

Q14139	Ubiquitin conjugation factor E4 A OS=Homo sapiens GN=UBE4A PE=1 SV=2 - [UBE4A_HUMAN]	3.66	1	2	2	2						20.93	0	1						20.67		20.67	NA	NA	
Q15208	Serine/threonine-protein kinase 38 OS=Homo sapiens GN=STK38 PE=1 SV=1 - [STK38_HUMAN]	3.66	1	1	1	1						23.23	0	1						22.97		22.97	NA	NA	
Q8TCT8	Signal peptide peptidase-like 2A OS=Homo sapiens GN=SPPL2A PE=1 SV=2 - [SPP2A_HUMAN]	3.65	1	2	2	3				22.55		22.81	0	2				22.31		22.55		22.43	NA	NA	
Q15653	NF-kappa-B inhibitor beta OS=Homo sapiens GN=NFKBIB PE=1 SV=2 - [IKBB_HUMAN]	3.65	1	1	1	5				19.29	20.77	20.45	0	3				19.04	20.98	20.18		20.07	0.000	0.000	
Q9HAV4	Exportin-5 OS=Homo sapiens GN=XPO5 PE=1 SV=1 - [XPO5_HUMAN]	3.65	1	3	3	4					21.88	23.11	0	2					22.10	22.85		22.48	NA	NA	
Q9NXH9	tRNA (guanine(26)-N(2))-dimethyltransferase OS=Homo sapiens GN=TRMT1 PE=1 SV=1 - [TRM1_HUMAN]	3.64	1	1	1	1				22.10			0	1				21.85				21.85	NA	NA	
Q52LJ0	Protein FAM98B OS=Homo sapiens GN=FAM98B PE=1 SV=1 - [FA98B_HUMAN]	3.64	1	1	1	1						21.86	0	1						21.60		21.60	NA	NA	
A1L0T0	Acetolactate synthase-like protein OS=Homo sapiens GN=ILVBL PE=1 SV=2 - [ILVBL_HUMAN]	3.64	1	1	1	1						22.66	0	1						22.40		22.40	NA	NA	
P04920	Anion exchange protein 2 OS=Homo sapiens GN=SLC4A2 PE=1 SV=4 - [B3A2_HUMAN]	3.63	2	3	3	5	21.86	22.28					2	0	22.03	22.34					22.19		NA	NA	
Q9H0F6	Sharpin OS=Homo sapiens GN=SHARPIN PE=1 SV=1 - [SHRPN_HUMAN]	3.62	1	1	1	1	18.01						1	0	18.17						18.17		NA	NA	
Q9H6Y5	PDZ domain-containing protein MAGIX OS=Homo sapiens GN=MAGIX PE=1 SV=3 - [MAGIX_HUMAN]	3.59	1	1	1	1					17.72		0	1					17.94			17.94	NA	NA	
P07942	Laminin subunit beta-1 OS=Homo sapiens GN=LAMB1 PE=1 SV=2 - [LAMB1_HUMAN]	3.58	1	4	4	5	20.86			21.42	20.52		1	2	21.02			21.18	20.74		21.02	20.96	NA	NA	-0.062
Q9NTJ5	Phosphatidylinositide phosphatase SAC1 OS=Homo sapiens GN=SACM1L PE=1 SV=2 - [SAC1_HUMAN]	3.58	1	2	2	3				20.59		21.83	0	2				20.34		21.57		20.95	NA	NA	
Q96HE8	Transmembrane protein 80 OS=Homo sapiens GN=TMEM80 PE=2 SV=3 - [TMM80_HUMAN]	3.57	1	1	1	1				23.53			0	1				23.28				23.28	NA	NA	
O43760	Synaptogyrin-2 OS=Homo sapiens GN=SYNGR2 PE=1 SV=1 - [SNG2_HUMAN]	3.57	1	1	1	1						22.97	0	1						22.71		22.71	NA	NA	
Q9NQY0	Bridging integrator 3 OS=Homo sapiens GN=BIN3 PE=1 SV=1 - [BIN3_HUMAN]	3.56	1	1	1	3				21.88	21.52	22.25	0	3				21.63	21.73	21.98		21.78	0.000	0.000	
Q8TBX8	Phosphatidylinositol 5-phosphate 4-kinase type-2 gamma OS=Homo sapiens GN=PIP4K2C PE=1 SV=3 - [PI42C_HUMAN]	3.56	1	1	1	2					22.07	22.21	0	2					22.29	21.95		22.12	NA	NA	
A6NJY1	Putative SLC9B1-like protein SLC9B1P1 OS=Homo sapiens GN=SLC9B1P1 PE=5 SV=4 - [SL9P1_HUMAN]	3.55	2	1	1	1	25.83						1	0	25.99						25.99		NA	NA	
Q96P70	Importin-9 OS=Homo sapiens GN=IPO9 PE=1 SV=3 - [IPO9_HUMAN]	3.55	1	3	3	4		23.39	21.06			21.61	2	1		23.45	21.12			21.35	22.29	21.35	NA	NA	-0.933
P34949	Mannose-6-phosphate isomerase OS=Homo sapiens GN=MPI PE=1 SV=2 - [MPI_HUMAN]	3.55	1	1	1	2					19.87	21.44	0	2					20.09	21.18		20.63	NA	NA	
Q8N7X8	SIGLEC family-like protein 1 OS=Homo sapiens GN=SIGLECL1 PE=2 SV=1 - [SIGL1_HUMAN]	3.55	1	1	1	1					23.26		0	1					23.47			23.47	NA	NA	
O95081	Arf-GAP domain and FG repeat-containing protein 2 OS=Homo sapiens GN=AGFG2 PE=1 SV=2 - [AGFG2_HUMAN]	3.53	2	1	2	4	22.10			22.20	21.28		1	2	22.26			21.95	21.50		22.26	21.72	NA	NA	-0.534
Q8WUX9	Charged multivesicular body protein 7 OS=Homo sapiens GN=CHMP7 PE=1 SV=1 - [CHMP7_HUMAN]	3.53	1	1	1	1				20.32			0	1				20.07				20.07	NA	NA	
O14494	Phospholipid phosphatase 1 OS=Homo sapiens GN=PLPP1 PE=1 SV=1 - [PLPP1_HUMAN]	3.52	1	1	1	2	21.70		21.66				2	0	21.86		21.72				21.79		NA	NA	
Q9Y6V7	Probable ATP-dependent RNA helicase DDX49 OS=Homo sapiens GN=DDX49 PE=1 SV=1 - [DDX49_HUMAN]	3.52	1	1	1	1					19.97		0	1					20.19			20.19	NA	NA	
Q15003	Condensin complex subunit 2 OS=Homo sapiens GN=NCAPH PE=1 SV=3 - [CND2_HUMAN]	3.51	1	2	2	3	20.89	21.27					2	0	21.05	21.34					21.20		NA	NA	
Q9NRR5	Ubiquilin-4 OS=Homo sapiens GN=UBQLN4 PE=1 SV=2 - [UBQL4_HUMAN]	3.49	1	1	2	4				23.06	22.82	24.02	0	3				22.82	23.03	23.76		23.20	0.000	0.000	
Q9NRG9	Aladin OS=Homo sapiens GN=AAAS PE=1 SV=1 - [AAAS_HUMAN]	3.48	1	1	1	4		20.51		21.16		21.73	1	2		20.58		20.92		21.47	20.58	21.19	NA	NA	0.608
A6NED2	RCC1 domain-containing protein 1 OS=Homo sapiens GN=RCCD1 PE=1 SV=1 - [RCCD1_HUMAN]	3.46	1	1	1	1	21.26						1	0	21.42						21.42		NA	NA	
O75152	Zinc finger CCCH domain-containing protein 11A OS=Homo sapiens GN=ZC3H11A PE=1 SV=3 - [ZC11A_HUMAN]	3.46	2	1	2	4				21.36	21.46	21.55	0	3				21.12	21.68	21.29		21.36	0.000	0.000	
Q9UM82	Spermatogenesis-associated protein 2 OS=Homo sapiens GN=SPATA2 PE=1 SV=2 - [SPAT2_HUMAN]	3.46	1	1	1	1		21.41					1	0		21.48					21.48		NA	NA	
Q7Z7A3	Cytoplasmic tRNA 2-thiolation protein 1 OS=Homo sapiens GN=CTU1 PE=1 SV=1 - [CTU1_HUMAN]	3.45	1	1	1	1			21.10				1	0			21.16				21.16		NA	NA	
Q8NBF2	NHL repeat-containing protein 2 OS=Homo sapiens GN=NHLRC2 PE=1 SV=1 - [NHLC2_HUMAN]	3.44	1	1	1	3				23.12		22.53	0	2				22.87		22.27		22.57	NA	NA	
P43304	Glycerol-3-phosphate dehydrogenase, mitochondrial OS=Homo sapiens GN=GPD2 PE=1 SV=3 - [GPDM_HUMAN]	3.44	1	1	1	4					22.69	23.76	0	2					22.91	23.50		23.20	NA	NA	
Q53LP3	Ankyrin repeat domain-containing protein SOWAHC OS=Homo sapiens GN=SOWAHC PE=1 SV=1 - [SWAHC_HUMAN]	3.43	1	2	2	5	19.51	19.11		20.45		20.05	2	2	19.67	19.18		20.20		19.79	19.42	19.99	0.217	0.089	0.571
Q7L2E3	Putative ATP-dependent RNA helicase DHX30 OS=Homo sapiens GN=DHX30 PE=1 SV=1 - [DHX30_HUMAN]	3.43	1	2	2	3				22.95	22.21		0	2				22.70	22.43			22.57	NA	NA	
P49354	Protein farnesyltransferase/geranylgeranyltransferase type-1 subunit alpha OS=Homo sapiens GN=FNTA PE=1 SV=1 - [FNTA_HUMAN]	3.43	1	1	1	1				19.77			0	1				19.53				19.53	NA	NA	
Q9Y496	Kinesin-like protein KIF3A OS=Homo sapiens GN=KIF3A PE=1 SV=4 - [KIF3A_HUMAN]	3.43	4	1	2	10			21.46	21.94	21.90	21.77	1	3			21.52	21.69	22.12	21.51	21.52	21.77	NA	NA	0.256
Q15020	Squamous cell carcinoma antigen recognized by T-cells 3 OS=Homo sapiens GN=SART3 PE=1 SV=1 - [SART3_HUMAN]	3.43	1	2	2	2					23.19		0	1					23.41			23.41	NA	NA	
Q6UXD5	Seizure 6-like protein 2 OS=Homo sapiens GN=SEZ6L2 PE=1 SV=2 - [SE6L2_HUMAN]	3.41	1	1	1	1				22.58			0	1				22.33				22.33	NA	NA	
P19838	Nuclear factor NF-kappa-B p105 subunit OS=Homo sapiens GN=NFKB1 PE=1 SV=2 - [NFKB1_HUMAN]	3.41	1	3	3	3						22.73	0	1						22.47		22.47	NA	NA	
Q8WX93	Palladin OS=Homo sapiens GN=PALLD PE=1 SV=3 - [PALLD_HUMAN]	3.40	2	4	4	7				21.38	21.35	22.05	0	3				21.14	21.57	21.79		21.50	0.000	0.000	
Q6IN84	rRNA methyltransferase 1, mitochondrial OS=Homo sapiens GN=MRM1 PE=1 SV=1 - [MRM1_HUMAN]	3.40	1	1	1	2					20.97	21.20	0	2					21.19	20.94		21.07	NA	NA	
Q8N3J3	Uncharacterized protein C17orf53 OS=Homo sapiens GN=C17orf53 PE=1 SV=1 - [CQ053_HUMAN]	3.40	1	2	2	2			19.92				1	0			19.98				19.98		NA	NA	
O60678	Protein arginine N-methyltransferase 3 OS=Homo sapiens GN=PRMT3 PE=1 SV=3 - [ANM3_HUMAN]	3.39	1	1	1	2						22.23	0	1						21.97		21.97	NA	NA	
P10696	Alkaline phosphatase, placental-like OS=Homo sapiens GN=ALPPL2 PE=2 SV=4 - [PPBN_HUMAN]	3.38	2	1	1	2				22.46			0	1				22.22				22.22	NA	NA	
O95864	Fatty acid desaturase 2 OS=Homo sapiens GN=FADS2 PE=1 SV=1 - [FADS2_HUMAN]	3.38	1	1	1	2						22.77	0	1						22.51		22.51	NA	NA	
P61764	Syntaxin-binding protein 1 OS=Homo sapiens GN=STXBP1 PE=1 SV=1 - [STXB1_HUMAN]	3.37	1	1	1	1	23.60						1	0	23.76						23.76		NA	NA	
Q96M27	Protein PRRC1 OS=Homo sapiens GN=PRRC1 PE=1 SV=1 - [PRRC1_HUMAN]	3.37	1	1	1	1				22.41			0	1				22.16				22.16	NA	NA	
Q7Z698	Sprouty-related, EVH1 domain-containing protein 2 OS=Homo sapiens GN=SPRED2 PE=1 SV=2 - [SPRE2_HUMAN]	3.35	1	1	1	1	21.30						1	0	21.46						21.46		NA	NA	
Q7Z3B4	Nucleoporin p54 OS=Homo sapiens GN=NUP54 PE=1 SV=2 - [NUP54_HUMAN]	3.35	1	1	1	1						21.80	0	1						21.54		21.54	NA	NA	
Q9H1J7	Protein Wnt-5b OS=Homo sapiens GN=WNT5B PE=2 SV=2 - [WNT5B_HUMAN]	3.34	2	1	1	1				21.01			0	1				20.76				20.76	NA	NA	
Q9Y5P6	Mannose-1-phosphate guanyltransferase beta OS=Homo sapiens GN=GMPPB PE=1 SV=2 - [GMPPB_HUMAN]	3.33	1	1	1	1	20.71						1	0	20.87						20.87		NA	NA	
Q9UGC6	Regulator of G-protein signaling 17 OS=Homo sapiens GN=RGS17 PE=1 SV=2 - [RGS17_HUMAN]	3.33	3	1	1	1	21.50						1	0	21.66						21.66		NA	NA	
Q96S86	Hyaluronan and proteoglycan link protein 3 OS=Homo sapiens GN=HAPLN3 PE=2 SV=1 - [HPLN3_HUMAN]	3.33	1	1	1	1						20.12	0	1						19.86		19.86	NA	NA	
O75923	Dysferlin OS=Homo sapiens GN=DYSF PE=1 SV=1 - [DYSF_HUMAN]	3.32	1	2	3	4	26.30	23.25			25.44		2	1	26.46	23.32			25.66		24.89	25.66	NA	NA	0.769
O00273	DNA fragmentation factor subunit alpha OS=Homo sapiens GN=DFFA PE=1 SV=1 - [DFFA_HUMAN]	3.32	1	1	1	1					18.66		0	1					18.88			18.88	NA	NA	
P11182	Lipoamide acyltransferase component of branched-chain alpha-keto acid dehydrogenase complex, mitochondrial OS=Homo sapiens GN=DBT PE=1 SV=3 - [ODB2_HUMAN]	3.32	1	1	1	1					22.56		0	1					22.78			22.78	NA	NA	
Q9H1K6	Mesoderm development candidate 1 OS=Homo sapiens GN=MESDC1 PE=1 SV=1 - [MESD1_HUMAN]	3.31	1	1	1	3	22.74			22.97	22.77		1	2	22.90			22.72	22.99		22.90	22.86	NA	NA	-0.045
Q15637	Splicing factor 1 OS=Homo sapiens GN=SF1 PE=1 SV=4 - [SF01_HUMAN]	3.29	1	1	1	2			21.51		23.02		1	1			21.57		23.24		21.57	23.24	NA	NA	1.663
Q14692	Ribosome biogenesis protein BMS1 homolog OS=Homo sapiens GN=BMS1 PE=1 SV=1 - [BMS1_HUMAN]	3.28	1	2	2	2				19.72	22.47		0	2				19.48	22.68			21.08	NA	NA	
Q7L5Y9	Macrophage erythroblast attacher OS=Homo sapiens GN=MAEA PE=1 SV=1 - [MAEA_HUMAN]	3.28	1	1	1	1				20.63			0	1				20.38				20.38	NA	NA	
P55283	Cadherin-4 OS=Homo sapiens GN=CDH4 PE=2 SV=2 - [CADH4_HUMAN]	3.28	1	1	1	1		23.64					1	0		23.71					23.71		NA	NA	
Q6L8Q7	2',5'-phosphodiesterase 12 OS=Homo sapiens GN=PDE12 PE=1 SV=2 - [PDE12_HUMAN]	3.28	1	1	1	1						23.35	0	1						23.08		23.08	NA	NA	
Q8N565	Melanoregulin OS=Homo sapiens GN=MREG PE=1 SV=1 - [MREG_HUMAN]	3.27	1	1	1	1	23.33						1	0	23.49						23.49		NA	NA	
Q16790	Carbonic anhydrase 9 OS=Homo sapiens GN=CA9 PE=1 SV=2 - [CAH9_HUMAN]	3.27	1	1	1	1		23.12					1	0		23.19					23.19		NA	NA	
P98160	Basement membrane-specific heparan sulfate proteoglycan core protein OS=Homo sapiens GN=HSPG2 PE=1 SV=4 - [PGBM_HUMAN]	3.26	1	9	9	13	20.94	19.52	22.20				3	0	21.10	19.59	22.26				20.99		0.000	0.000	
Q15042	Rab3 GTPase-activating protein catalytic subunit OS=Homo sapiens GN=RAB3GAP1 PE=1 SV=3 - [RB3GP_HUMAN]	3.26	1	2	2	2						22.62	0	1						22.36		22.36	NA	NA	
P41214	Eukaryotic translation initiation factor 2D OS=Homo sapiens GN=EIF2D PE=1 SV=3 - [EIF2D_HUMAN]	3.25	1	1	1	1					21.30		0	1					21.52			21.52	NA	NA	
Q9UHE8	Metalloreductase STEAP1 OS=Homo sapiens GN=STEAP1 PE=2 SV=1 - [STEA1_HUMAN]	3.24	1	1	1	1				21.21			0	1				20.97				20.97	NA	NA	
Q13588	GRB2-related adapter protein OS=Homo sapiens GN=GRAP PE=1 SV=1 - [GRAP_HUMAN]	3.23	1	1	1	3	21.51	23.18	22.89				3	0	21.67	23.25	22.95				22.62		0.000	0.000	
Q13769	THO complex subunit 5 homolog OS=Homo sapiens GN=THOC5 PE=1 SV=2 - [THOC5_HUMAN]	3.22	1	1	1	4				23.18	21.93		0	2				22.93	22.14			22.54	NA	NA	
O14495	Lipid phosphate phosphohydrolase 3 OS=Homo sapiens GN=PPAP2B PE=1 SV=1 - [LPP3_HUMAN]	3.22	1	1	1	1			21.12				1	0			21.18				21.18		NA	NA	
P21912	Succinate dehydrogenase [ubiquinone] iron-sulfur subunit, mitochondrial OS=Homo sapiens GN=SDHB PE=1 SV=3 - [SDHB_HUMAN]	3.21	1	1	1	1					23.07		0	1					23.29			23.29	NA	NA	
Q9BW27	Nuclear pore complex protein Nup85 OS=Homo sapiens GN=NUP85 PE=1 SV=1 - [NUP85_HUMAN]	3.20	1	1	1	2				20.98			0	1				20.74				20.74	NA	NA	
Q9BTC8	Metastasis-associated protein MTA3 OS=Homo sapiens GN=MTA3 PE=1 SV=2 - [MTA3_HUMAN]	3.20	2	1	2	3			19.58	22.53	22.70		1	2			19.64	22.29	22.92		19.64	22.60	NA	NA	2.961
Q96JJ3	Engulfment and cell motility protein 2 OS=Homo sapiens GN=ELMO2 PE=1 SV=2 - [ELMO2_HUMAN]	3.19	1	1	1	5	22.92			22.64	23.16	23.85	1	3	23.08			22.39	23.38	23.59	23.08	23.12	NA	NA	0.043
Q13595	Transformer-2 protein homolog alpha OS=Homo sapiens GN=TRA2A PE=1 SV=1 - [TRA2A_HUMAN]	3.19	1	1	1	2					21.69		0	1					21.91			21.91	NA	NA	
Q14CX7	N-alpha-acetyltransferase 25, NatB auxiliary subunit OS=Homo sapiens GN=NAA25 PE=1 SV=1 - [NAA25_HUMAN]	3.19	1	2	2	2						22.32	0	1						22.06		22.06	NA	NA	
Q5VTR2	E3 ubiquitin-protein ligase BRE1A OS=Homo sapiens GN=RNF20 PE=1 SV=2 - [BRE1A_HUMAN]	3.18	1	2	2	2					20.62		0	1					20.84			20.84	NA	NA	
Q99943	1-acyl-sn-glycerol-3-phosphate acyltransferase alpha OS=Homo sapiens GN=AGPAT1 PE=1 SV=2 - [PLCA_HUMAN]	3.18	1	1	1	4					20.17	21.30	0	2					20.38	21.04		20.71	NA	NA	
Q9Y2B5	VPS9 domain-containing protein 1 OS=Homo sapiens GN=VPS9D1 PE=2 SV=2 - [VP9D1_HUMAN]	3.17	1	2	2	4	21.53	21.67					2	0	21.69	21.74					21.72		NA	NA	
P05090	Apolipoprotein D OS=Homo sapiens GN=APOD PE=1 SV=1 - [APOD_HUMAN]	3.17	1	1	1	2				24.14		24.21	0	2				23.90		23.95		23.92	NA	NA	
P46734	Dual specificity mitogen-activated protein kinase kinase 3 OS=Homo sapiens GN=MAP2K3 PE=1 SV=2 - [MP2K3_HUMAN]	3.17	1	1	1	1						21.94	0	1						21.68		21.68	NA	NA	
Q9UBB4	Ataxin-10 OS=Homo sapiens GN=ATXN10 PE=1 SV=1 - [ATX10_HUMAN]	3.16	1	1	1	5	21.76			23.12	22.24	23.37	1	3	21.93			22.88	22.46	23.11	21.93	22.81	NA	NA	0.889
Q9NV06	DDB1- and CUL4-associated factor 13 OS=Homo sapiens GN=DCAF13 PE=1 SV=2 - [DCA13_HUMAN]	3.15	1	1	1	1					21.64		0	1					21.86			21.86	NA	NA	
Q9BPX3	Condensin complex subunit 3 OS=Homo sapiens GN=NCAPG PE=1 SV=1 - [CND3_HUMAN]	3.15	1	2	2	3					22.28	23.02	0	2					22.50	22.76		22.63	NA	NA	
Q92995	Ubiquitin carboxyl-terminal hydrolase 13 OS=Homo sapiens GN=USP13 PE=1 SV=2 - [UBP13_HUMAN]	3.13	1	1	1	1	22.69						1	0	22.85						22.85		NA	NA	
Q14624	Inter-alpha-trypsin inhibitor heavy chain H4 OS=Homo sapiens GN=ITIH4 PE=1 SV=4 - [ITIH4_HUMAN]	3.12	1	3	3	8	23.93	22.47	23.66	24.44			3	1	24.09	22.54	23.72	24.19			23.45	24.19	NA	NA	0.745
Q15061	WD repeat-containing protein 43 OS=Homo sapiens GN=WDR43 PE=1 SV=3 - [WDR43_HUMAN]	3.10	1	2	2	3				24.79		20.86	0	2				24.55		20.60		22.58	NA	NA	
P19174	1-phosphatidylinositol 4,5-bisphosphate phosphodiesterase gamma-1 OS=Homo sapiens GN=PLCG1 PE=1 SV=1 - [PLCG1_HUMAN]	3.10	1	2	2	2						21.79	0	1						21.53		21.53	NA	NA	
P35269	General transcription factor IIF subunit 1 OS=Homo sapiens GN=GTF2F1 PE=1 SV=2 - [T2FA_HUMAN]	3.09	1	1	1	1						24.91	0	1						24.65		24.65	NA	NA	
P35914	Hydroxymethylglutaryl-CoA lyase, mitochondrial OS=Homo sapiens GN=HMGCL PE=1 SV=2 - [HMGCL_HUMAN]	3.08	1	1	1	1						20.69	0	1						20.43		20.43	NA	NA	
Q6UWI4	Protein shisa-2 homolog OS=Homo sapiens GN=SHISA2 PE=1 SV=1 - [SHSA2_HUMAN]	3.05	1	1	1	2	18.63					19.31	1	1	18.79					19.05	18.79	19.05	NA	NA	0.253
Q8TAA9	Vang-like protein 1 OS=Homo sapiens GN=VANGL1 PE=1 SV=1 - [VANG1_HUMAN]	3.05	1	1	1	2				22.39			0	1				22.14				22.14	NA	NA	
Q9H7B4	Histone-lysine N-methyltransferase SMYD3 OS=Homo sapiens GN=SMYD3 PE=1 SV=4 - [SMYD3_HUMAN]	3.04	1	1	1	1	21.92						1	0	22.08						22.08		NA	NA	
Q9ULI3	Protein HEG homolog 1 OS=Homo sapiens GN=HEG1 PE=1 SV=3 - [HEG1_HUMAN]	3.04	1	4	4	5	21.72	22.41	22.45	22.27			3	1	21.88	22.48	22.51	22.03			22.29	22.03	NA	NA	-0.261
Q9Y5P4	Collagen type IV alpha-3-binding protein OS=Homo sapiens GN=COL4A3BP PE=1 SV=1 - [C43BP_HUMAN]	3.04	1	1	1	1						21.51	0	1						21.25		21.25	NA	NA	
P82933	28S ribosomal protein S9, mitochondrial OS=Homo sapiens GN=MRPS9 PE=1 SV=2 - [RT09_HUMAN]	3.03	1	1	1	4		20.39		20.01	21.74	21.84	1	3		20.46		19.76	21.95	21.58	20.46	21.10	NA	NA	0.642
Q9C0C9	(E3-independent) E2 ubiquitin-conjugating enzyme OS=Homo sapiens GN=UBE2O PE=1 SV=3 - [UBE2O_HUMAN]	3.02	1	3	3	3					21.86		0	1					22.07			22.07	NA	NA	
P55039	Developmentally-regulated GTP-binding protein 2 OS=Homo sapiens GN=DRG2 PE=1 SV=1 - [DRG2_HUMAN]	3.02	1	1	1	2			20.63				1	0			20.69				20.69		NA	NA	
Q8NDI1	EH domain-binding protein 1 OS=Homo sapiens GN=EHBP1 PE=1 SV=3 - [EHBP1_HUMAN]	3.01	1	3	3	4	20.23	19.83		20.37			2	1	20.39	19.90		20.13			20.14	20.13	NA	NA	-0.015
Q8IWS0	PHD finger protein 6 OS=Homo sapiens GN=PHF6 PE=1 SV=1 - [PHF6_HUMAN]	3.01	1	1	1	1					21.15		0	1					21.36			21.36	NA	NA	
Q9NVA1	Ubiquinol-cytochrome-c reductase complex assembly factor 1 OS=Homo sapiens GN=UQCC1 PE=1 SV=3 - [UQCC1_HUMAN]	3.01	1	1	1	1					19.96		0	1					20.17			20.17	NA	NA	
Q86YS7	C2 domain-containing protein 5 OS=Homo sapiens GN=C2CD5 PE=1 SV=1 - [C2CD5_HUMAN]	3.00	1	2	2	2	20.45		21.52				2	0	20.62		21.58				21.10		NA	NA	
P14635	G2/mitotic-specific cyclin-B1 OS=Homo sapiens GN=CCNB1 PE=1 SV=1 - [CCNB1_HUMAN]	3.00	1	1	1	2					21.75	22.61	0	2					21.96	22.35		22.15	NA	NA	
P36897	TGF-beta receptor type-1 OS=Homo sapiens GN=TGFBR1 PE=1 SV=1 - [TGFR1_HUMAN]	2.98	1	1	1	1	21.82						1	0	21.98						21.98		NA	NA	
O75794	Cell division cycle protein 123 homolog OS=Homo sapiens GN=CDC123 PE=1 SV=1 - [CD123_HUMAN]	2.98	1	1	1	2					21.49	21.76	0	2					21.71	21.50		21.60	NA	NA	
P43363	Melanoma-associated antigen 10 OS=Homo sapiens GN=MAGEA10 PE=2 SV=2 - [MAGAA_HUMAN]	2.98	1	1	1	1						21.84	0	1						21.58		21.58	NA	NA	
Q32P41	tRNA (guanine(37)-N1)-methyltransferase OS=Homo sapiens GN=TRMT5 PE=1 SV=2 - [TRM5_HUMAN]	2.95	1	1	1	1					20.68		0	1					20.89			20.89	NA	NA	
Q9BRQ8	Apoptosis-inducing factor 2 OS=Homo sapiens GN=AIFM2 PE=1 SV=1 - [AIFM2_HUMAN]	2.95	1	1	1	1					21.10		0	1					21.32			21.32	NA	NA	
Q9H267	Vacuolar protein sorting-associated protein 33B OS=Homo sapiens GN=VPS33B PE=1 SV=2 - [VP33B_HUMAN]	2.92	1	1	1	2				23.26		23.69	0	2				23.02		23.43		23.22	NA	NA	
P35610	Sterol O-acyltransferase 1 OS=Homo sapiens GN=SOAT1 PE=1 SV=3 - [SOAT1_HUMAN]	2.91	1	1	1	4				22.12	21.53	22.70	0	3				21.88	21.75	22.44		22.02	0.000	0.000	
O95365	Zinc finger and BTB domain-containing protein 7A OS=Homo sapiens GN=ZBTB7A PE=1 SV=1 - [ZBT7A_HUMAN]	2.91	1	1	1	3				17.94	17.37		0	2				17.69	17.58			17.64	NA	NA	
Q15113	Procollagen C-endopeptidase enhancer 1 OS=Homo sapiens GN=PCOLCE PE=1 SV=2 - [PCOC1_HUMAN]	2.90	1	1	1	1	20.81						1	0	20.97						20.97		NA	NA	
Q96JB2	Conserved oligomeric Golgi complex subunit 3 OS=Homo sapiens GN=COG3 PE=1 SV=3 - [COG3_HUMAN]	2.90	1	2	2	2				17.54			0	1				17.29				17.29	NA	NA	
P30405	Peptidyl-prolyl cis-trans isomerase F, mitochondrial OS=Homo sapiens GN=PPIF PE=1 SV=1 - [PPIF_HUMAN]	2.90	2	1	1	2					21.20	22.63	0	2					21.42	22.37		21.90	NA	NA	
P11498	Pyruvate carboxylase, mitochondrial OS=Homo sapiens GN=PC PE=1 SV=2 - [PYC_HUMAN]	2.89	1	2	2	4				22.39	22.29	23.23	0	3				22.14	22.50	22.97		22.54	0.000	0.000	
Q9P0J7	E3 ubiquitin-protein ligase KCMF1 OS=Homo sapiens GN=KCMF1 PE=1 SV=2 - [KCMF1_HUMAN]	2.89	1	1	1	1					19.90		0	1					20.12			20.12	NA	NA	
Q9H7D7	WD repeat-containing protein 26 OS=Homo sapiens GN=WDR26 PE=1 SV=3 - [WDR26_HUMAN]	2.87	1	1	1	3	21.99			23.21	23.27		1	2	22.15			22.96	23.49		22.15	23.22	NA	NA	1.073
A2VCK2	Doublecortin domain-containing protein 2B OS=Homo sapiens GN=DCDC2B PE=1 SV=1 - [DCD2B_HUMAN]	2.87	1	1	1	1	20.93						1	0	21.09						21.09		NA	NA	
Q92804	TATA-binding protein-associated factor 2N OS=Homo sapiens GN=TAF15 PE=1 SV=1 - [RBP56_HUMAN]	2.87	1	1	2	6		23.02		23.56	22.90	23.52	1	3		23.09		23.32	23.12	23.26	23.09	23.23	NA	NA	0.144
Q13555	Calcium/calmodulin-dependent protein kinase type II subunit gamma OS=Homo sapiens GN=CAMK2G PE=1 SV=3 - [KCC2G_HUMAN]	2.87	1	1	1	2					21.83	22.41	0	2					22.05	22.15		22.10	NA	NA	
P56945	Breast cancer anti-estrogen resistance protein 1 OS=Homo sapiens GN=BCAR1 PE=1 SV=2 - [BCAR1_HUMAN]	2.87	1	1	1	2						21.03	0	1						20.77		20.77	NA	NA	
O75586	Mediator of RNA polymerase II transcription subunit 6 OS=Homo sapiens GN=MED6 PE=1 SV=2 - [MED6_HUMAN]	2.85	1	1	1	5	23.35		22.79	23.73	22.49		2	2	23.52		22.85	23.48	22.70		23.18	23.09	0.876	0.256	-0.090
Q96J01	THO complex subunit 3 OS=Homo sapiens GN=THOC3 PE=1 SV=1 - [THOC3_HUMAN]	2.85	1	1	1	2	22.15				22.30		1	1	22.31				22.52		22.31	22.52	NA	NA	0.206

P08582	Melanotransferrin OS=Homo sapiens GN=MFI2 PE=1 SV=2 - [TRFM_HUMAN]	2.85	1	2	2	6	21.70		21.99			22.39	2	1	21.86		22.05			22.13	21.96	22.13	NA	NA	0.170
Q8N8N7	Prostaglandin reductase 2 OS=Homo sapiens GN=PTGR2 PE=1 SV=1 - [PTGR2_HUMAN]	2.85	1	1	1	2				19.84		21.46	0	2				19.60		21.20		20.40	NA	NA	
Q68EM7	Rho GTPase-activating protein 17 OS=Homo sapiens GN=ARHGAP17 PE=1 SV=1 - [RHG17_HUMAN]	2.84	1	1	1	3	24.11					24.24	1	1	24.28					23.98	24.28	23.98	NA	NA	-0.300
Q9BXS5	AP-1 complex subunit mu-1 OS=Homo sapiens GN=AP1M1 PE=1 SV=3 - [AP1M1_HUMAN]	2.84	1	1	1	1				21.79			0	1				21.55				21.55	NA	NA	
Q9Y697	Cysteine desulfurase, mitochondrial OS=Homo sapiens GN=NFS1 PE=1 SV=3 - [NFS1_HUMAN]	2.84	1	1	1	1					21.11		0	1					21.32			21.32	NA	NA	
Q9H832	Ubiquitin-conjugating enzyme E2 Z OS=Homo sapiens GN=UBE2Z PE=1 SV=2 - [UBE2Z_HUMAN]	2.82	1	1	1	1	21.18						1	0	21.34						21.34		NA	NA	
O75382	Tripartite motif-containing protein 3 OS=Homo sapiens GN=TRIM3 PE=1 SV=2 - [TRIM3_HUMAN]	2.82	1	1	1	2					20.02	20.38	0	2					20.24	20.11		20.18	NA	NA	
Q16394	Exostosin-1 OS=Homo sapiens GN=EXT1 PE=1 SV=2 - [EXT1_HUMAN]	2.82	1	1	1	1						22.82	0	1						22.56		22.56	NA	NA	
Q7L7X3	Serine/threonine-protein kinase TAO1 OS=Homo sapiens GN=TAOK1 PE=1 SV=1 - [TAOK1_HUMAN]	2.80	1	1	2	2		22.66		22.19			1	1		22.73		21.94			22.73	21.94	NA	NA	-0.785
Q9NRL3	Striatin-4 OS=Homo sapiens GN=STRN4 PE=1 SV=2 - [STRN4_HUMAN]	2.79	1	2	2	2						21.13	0	1						20.87		20.87	NA	NA	
Q16850	Lanosterol 14-alpha demethylase OS=Homo sapiens GN=CYP51A1 PE=1 SV=3 - [CP51A_HUMAN]	2.78	1	1	1	4	20.65	20.79	22.13			21.63	3	1	20.81	20.86	22.19			21.37	21.29	21.37	NA	NA	0.085
Q8NG99	Olfactory receptor 7G2 OS=Homo sapiens GN=OR7G2 PE=3 SV=1 - [OR7G2_HUMAN]	2.78	1	1	1	3		22.62		25.03		24.62	1	2		22.69		24.79		24.36	22.69	24.57	NA	NA	1.885
Q8TCJ2	Dolichyl-diphosphooligosaccharide--protein glycosyltransferase subunit STT3B OS=Homo sapiens GN=STT3B PE=1 SV=1 - [STT3B_HUMAN]	2.78	1	2	2	2						23.28	0	1						23.02		23.02	NA	NA	
Q16537	Serine/threonine-protein phosphatase 2A 56 kDa regulatory subunit epsilon isoform OS=Homo sapiens GN=PPP2R5E PE=1 SV=1 - [2A5E_HUMAN]	2.78	1	1	1	1						22.56	0	1						22.30		22.30	NA	NA	
Q5VT25	Serine/threonine-protein kinase MRCK alpha OS=Homo sapiens GN=CDC42BPA PE=1 SV=1 - [MRCKA_HUMAN]	2.77	2	5	5	9	21.40	26.38	25.64			27.08	3	1	21.56	26.45	25.70			26.82	24.57	26.82	NA	NA	2.247
O60637	Tetraspanin-3 OS=Homo sapiens GN=TSPAN3 PE=2 SV=1 - [TSN3_HUMAN]	2.77	1	1	1	4	25.73	25.56		25.72		25.08	2	2	25.90	25.63		25.47		24.82	25.76	25.15	0.220	0.090	-0.617
Q05682	Caldesmon OS=Homo sapiens GN=CALD1 PE=1 SV=3 - [CALD1_HUMAN]	2.77	1	3	3	5		20.83				20.80	1	1		20.90				20.54	20.90	20.54	NA	NA	-0.365
Q9UIA9	Exportin-7 OS=Homo sapiens GN=XPO7 PE=1 SV=3 - [XPO7_HUMAN]	2.76	1	2	2	5	19.93			20.89	20.38	21.87	1	3	20.10			20.65	20.60	21.61	20.10	20.95	NA	NA	0.857
O95067	G2/mitotic-specific cyclin-B2 OS=Homo sapiens GN=CCNB2 PE=1 SV=1 - [CCNB2_HUMAN]	2.76	1	1	1	3	20.28	20.57	20.85				3	0	20.44	20.64	20.91				20.66		0.000	0.000	
P27986	Phosphatidylinositol 3-kinase regulatory subunit alpha OS=Homo sapiens GN=PIK3R1 PE=1 SV=2 - [P85A_HUMAN]	2.76	1	1	1	1				21.61			0	1				21.37				21.37	NA	NA	
Q15072	Zinc finger protein OZF OS=Homo sapiens GN=ZNF146 PE=1 SV=2 - [OZF_HUMAN]	2.74	1	1	1	1				22.36			0	1				22.11				22.11	NA	NA	
Q8IWX8	Calcium homeostasis endoplasmic reticulum protein OS=Homo sapiens GN=CHERP PE=1 SV=3 - [CHERP_HUMAN]	2.73	1	1	1	4				22.26	22.22	22.93	0	3				22.01	22.44	22.67		22.37	0.000	0.000	
Q9H4G0	Band 4.1-like protein 1 OS=Homo sapiens GN=EPB41L1 PE=1 SV=2 - [E41L1_HUMAN]	2.72	1	1	2	2	22.70					22.55	1	1	22.86					22.29	22.86	22.29	NA	NA	-0.571
Q15269	Periodic tryptophan protein 2 homolog OS=Homo sapiens GN=PWP2 PE=2 SV=2 - [PWP2_HUMAN]	2.72	1	1	1	4					22.93	23.32	0	2					23.14	23.06		23.10	NA	NA	
Q8IX12	Cell division cycle and apoptosis regulator protein 1 OS=Homo sapiens GN=CCAR1 PE=1 SV=2 - [CCAR1_HUMAN]	2.70	1	2	2	4		18.85		20.85	21.60		1	2		18.92		20.61	21.81		18.92	21.21	NA	NA	2.294
Q5VV17	OTU domain-containing protein 1 OS=Homo sapiens GN=OTUD1 PE=1 SV=1 - [OTUD1_HUMAN]	2.70	1	1	1	2				25.54		25.61	0	2				25.30		25.35		25.32	NA	NA	
Q8N1F7	Nuclear pore complex protein Nup93 OS=Homo sapiens GN=NUP93 PE=1 SV=2 - [NUP93_HUMAN]	2.69	1	2	2	2					21.30		0	1					21.52			21.52	NA	NA	
Q9BXT2	Voltage-dependent calcium channel gamma-6 subunit OS=Homo sapiens GN=CACNG6 PE=2 SV=1 - [CCG6_HUMAN]	2.69	1	1	1	2					22.09	22.67	0	2					22.30	22.41		22.35	NA	NA	
Q9H0U3	Magnesium transporter protein 1 OS=Homo sapiens GN=MAGT1 PE=1 SV=1 - [MAGT1_HUMAN]	2.69	1	1	1	1						23.05	0	1						22.79		22.79	NA	NA	
Q9UH92	Max-like protein X OS=Homo sapiens GN=MLX PE=1 SV=2 - [MLX_HUMAN]	2.68	1	1	1	1				25.58			0	1				25.34				25.34	NA	NA	
Q8IWB7	WD repeat and FYVE domain-containing protein 1 OS=Homo sapiens GN=WDFY1 PE=1 SV=1 - [WDFY1_HUMAN]	2.68	1	1	1	1				22.12			0	1				21.87				21.87	NA	NA	
O94813	Slit homolog 2 protein OS=Homo sapiens GN=SLIT2 PE=1 SV=1 - [SLIT2_HUMAN]	2.68	1	3	3	3					19.45		0	1					19.67			19.67	NA	NA	
P40818	Ubiquitin carboxyl-terminal hydrolase 8 OS=Homo sapiens GN=USP8 PE=1 SV=1 - [UBP8_HUMAN]	2.68	1	1	1	2			21.39			21.44	1	1			21.45			21.18	21.45	21.18	NA	NA	-0.268
Q9BYE0	Transcription factor HES-7 OS=Homo sapiens GN=HES7 PE=1 SV=2 - [HES7_HUMAN]	2.67	1	1	1	1				22.19			0	1				21.95				21.95	NA	NA	
Q8TBE7	Solute carrier family 35 member G2 OS=Homo sapiens GN=SLC35G2 PE=1 SV=3 - [S35G2_HUMAN]	2.67	1	1	1	1		25.10					1	0		25.17					25.17		NA	NA	
Q09161	Nuclear cap-binding protein subunit 1 OS=Homo sapiens GN=NCBP1 PE=1 SV=1 - [NCBP1_HUMAN]	2.66	1	1	1	3				23.13	23.22	23.90	0	3				22.89	23.44	23.64		23.32	0.000	0.000	
Q05655	Protein kinase C delta type OS=Homo sapiens GN=PRKCD PE=1 SV=2 - [KPCD_HUMAN]	2.66	4	1	2	3				22.04		23.19	0	2				21.80		22.93		22.36	NA	NA	
Q969V3	Nicalin OS=Homo sapiens GN=NCLN PE=1 SV=2 - [NCLN_HUMAN]	2.66	1	1	1	1						24.57	0	1						24.31		24.31	NA	NA	
Q8WVM8	Sec1 family domain-containing protein 1 OS=Homo sapiens GN=SCFD1 PE=1 SV=4 - [SCFD1_HUMAN]	2.65	1	1	1	2					22.33	23.14	0	2					22.55	22.88		22.72	NA	NA	
Q676U5	Autophagy-related protein 16-1 OS=Homo sapiens GN=ATG16L1 PE=1 SV=2 - [A16L1_HUMAN]	2.64	13	1	2	2				21.24	22.40		0	2				20.99	22.62			21.80	NA	NA	
P49221	Protein-glutamine gamma-glutamyltransferase 4 OS=Homo sapiens GN=TGM4 PE=1 SV=2 - [TGM4_HUMAN]	2.63	1	1	2	10			26.59	24.93	24.44	24.93	1	3			26.65	24.68	24.66	24.67	26.65	24.67	NA	NA	-1.977
A6NMK8	Protein FAM196B OS=Homo sapiens GN=FAM196B PE=2 SV=1 - [F196B_HUMAN]	2.62	1	1	1	4	23.68	25.74	25.77				3	0	23.84	25.81	25.83				25.16		0.000	0.000	
Q9HC36	rRNA methyltransferase 3, mitochondrial OS=Homo sapiens GN=RNMTL1 PE=1 SV=2 - [MRM3_HUMAN]	2.62	1	1	1	2					21.06	21.46	0	2					21.28	21.19		21.24	NA	NA	
Q8TCT1	Phosphoethanolamine/phosphocholine phosphatase OS=Homo sapiens GN=PHOSPHO1 PE=1 SV=1 - [PHOP1_HUMAN]	2.62	2	1	1	1			23.05				1	0			23.11				23.11		NA	NA	
Q96NT5	Proton-coupled folate transporter OS=Homo sapiens GN=SLC46A1 PE=1 SV=1 - [PCFT_HUMAN]	2.61	1	1	1	1				21.75			0	1				21.50				21.50	NA	NA	
Q4KMP7	TBC1 domain family member 10B OS=Homo sapiens GN=TBC1D10B PE=1 SV=3 - [TB10B_HUMAN]	2.60	1	1	1	3				22.05			0	1				21.80				21.80	NA	NA	
Q14683	Structural maintenance of chromosomes protein 1A OS=Homo sapiens GN=SMC1A PE=1 SV=2 - [SMC1A_HUMAN]	2.60	1	3	3	4				21.49	21.35		0	2				21.24	21.57			21.41	NA	NA	
P11117	Lysosomal acid phosphatase OS=Homo sapiens GN=ACP2 PE=1 SV=3 - [PPAL_HUMAN]	2.60	1	1	1	2				21.37	19.89		0	2				21.12	20.11			20.62	NA	NA	
Q14493	Histone RNA hairpin-binding protein OS=Homo sapiens GN=SLBP PE=1 SV=1 - [SLBP_HUMAN]	2.59	1	1	1	14		21.84	28.33				2	0		21.90	28.39				25.15		NA	NA	
Q8TEQ6	Gem-associated protein 5 OS=Homo sapiens GN=GEMIN5 PE=1 SV=3 - [GEMI5_HUMAN]	2.59	1	3	3	5				21.79	21.65	21.99	0	3				21.54	21.86	21.73		21.71	0.000	0.000	
Q8NC56	LEM domain-containing protein 2 OS=Homo sapiens GN=LEMD2 PE=1 SV=1 - [LEMD2_HUMAN]	2.58	1	1	1	1						21.96	0	1						21.70		21.70	NA	NA	
P08069	Insulin-like growth factor 1 receptor OS=Homo sapiens GN=IGF1R PE=1 SV=1 - [IGF1R_HUMAN]	2.56	1	2	2	5	22.57	21.77	22.47				3	0	22.73	21.84	22.53				22.37		0.000	0.000	
Q9UKX7	Nuclear pore complex protein Nup50 OS=Homo sapiens GN=NUP50 PE=1 SV=2 - [NUP50_HUMAN]	2.56	1	1	1	1				19.23			0	1				18.99				18.99	NA	NA	
Q96BD6	SPRY domain-containing SOCS box protein 1 OS=Homo sapiens GN=SPSB1 PE=1 SV=1 - [SPSB1_HUMAN]	2.56	1	1	1	1				25.86			0	1				25.62				25.62	NA	NA	
Q8N684	Cleavage and polyadenylation specificity factor subunit 7 OS=Homo sapiens GN=CPSF7 PE=1 SV=1 - [CPSF7_HUMAN]	2.55	1	1	1	1						20.46	0	1						20.20		20.20	NA	NA	
Q8TB61	Adenosine 3'-phospho 5'-phosphosulfate transporter 1 OS=Homo sapiens GN=SLC35B2 PE=1 SV=1 - [S35B2_HUMAN]	2.55	1	1	1	1						22.92	0	1						22.66		22.66	NA	NA	
O60291	E3 ubiquitin-protein ligase MGRN1 OS=Homo sapiens GN=MGRN1 PE=1 SV=2 - [MGRN1_HUMAN]	2.54	1	1	1	3				20.44		18.04	0	2				20.20		17.78		18.99	NA	NA	
P09603	Macrophage colony-stimulating factor 1 OS=Homo sapiens GN=CSF1 PE=1 SV=2 - [CSF1_HUMAN]	2.53	1	1	1	3	22.37			22.25			1	1	22.53			22.00			22.53	22.00	NA	NA	-0.527
Q9H583	HEAT repeat-containing protein 1 OS=Homo sapiens GN=HEATR1 PE=1 SV=3 - [HEAT1_HUMAN]	2.52	1	3	3	4					21.30	21.19	0	2					21.52	20.93		21.23	NA	NA	
O94915	Protein furry homolog-like OS=Homo sapiens GN=FRYL PE=1 SV=2 - [FRYL_HUMAN]	2.52	1	3	3	8		20.96		21.94	21.96	22.38	1	3		21.02		21.70	22.17	22.12	21.02	22.00	NA	NA	0.970
Q15392	Delta(24)-sterol reductase OS=Homo sapiens GN=DHCR24 PE=1 SV=2 - [DHC24_HUMAN]	2.52	1	1	1	1						23.20	0	1						22.94		22.94	NA	NA	
Q96IG2	F-box/LRR-repeat protein 20 OS=Homo sapiens GN=FBXL20 PE=1 SV=2 - [FXL20_HUMAN]	2.52	1	1	1	1						20.58	0	1						20.32		20.32	NA	NA	
Q9H074	Polyadenylate-binding protein-interacting protein 1 OS=Homo sapiens GN=PAIP1 PE=1 SV=1 - [PAIP1_HUMAN]	2.51	1	1	1	3	21.61		20.80			22.97	2	1	21.77		20.86			22.71	21.32	22.71	NA	NA	1.394
Q6NY19	KN motif and ankyrin repeat domain-containing protein 3 OS=Homo sapiens GN=KANK3 PE=1 SV=1 - [KANK3_HUMAN]	2.50	1	1	1	1			23.98				1	0			24.04				24.04		NA	NA	
Q14997	Proteasome activator complex subunit 4 OS=Homo sapiens GN=PSME4 PE=1 SV=2 - [PSME4_HUMAN]	2.50	1	2	2	4				22.66	22.55		0	2				22.42	22.77			22.59	NA	NA	
A6NC97	Putative protein FAM172B OS=Homo sapiens GN=FAM172BP PE=5 SV=3 - [F172B_HUMAN]	2.49	1	1	1	1					23.80		0	1					24.02			24.02	NA	NA	
Q27J81	Inverted formin-2 OS=Homo sapiens GN=INF2 PE=1 SV=2 - [INF2_HUMAN]	2.48	1	2	2	2		21.20	21.00				2	0		21.27	21.06				21.17		NA	NA	
P51955	Serine/threonine-protein kinase Nek2 OS=Homo sapiens GN=NEK2 PE=1 SV=1 - [NEK2_HUMAN]	2.47	1	1	1	1	20.36						1	0	20.52						20.52		NA	NA	
Q9P253	Vacuolar protein sorting-associated protein 18 homolog OS=Homo sapiens GN=VPS18 PE=1 SV=2 - [VPS18_HUMAN]	2.47	1	2	2	4				20.56	20.01	21.00	0	3				20.31	20.22	20.74		20.43	0.000	0.000	
Q86TB9	Protein PAT1 homolog 1 OS=Homo sapiens GN=PATL1 PE=1 SV=2 - [PATL1_HUMAN]	2.47	1	1	1	1						22.37	0	1						22.11		22.11	NA	NA	
Q9H3P7	Golgi resident protein GCP60 OS=Homo sapiens GN=ACBD3 PE=1 SV=4 - [GCP60_HUMAN]	2.46	1	1	1	1						22.37	0	1						22.11		22.11	NA	NA	
Q6PKG0	La-related protein 1 OS=Homo sapiens GN=LARP1 PE=1 SV=2 - [LARP1_HUMAN]	2.46	1	2	2	2					22.72		0	1					22.94			22.94	NA	NA	
Q16706	Alpha-mannosidase 2 OS=Homo sapiens GN=MAN2A1 PE=1 SV=2 - [MA2A1_HUMAN]	2.45	1	1	1	1				21.15			0	1				20.90				20.90	NA	NA	
Q9HB40	Retinoid-inducible serine carboxypeptidase OS=Homo sapiens GN=SCPEP1 PE=1 SV=1 - [RISC_HUMAN]	2.43	1	1	1	2	21.81			21.50			1	1	21.98			21.26			21.98	21.26	NA	NA	-0.718
P38432	Coilin OS=Homo sapiens GN=COIL PE=1 SV=1 - [COIL_HUMAN]	2.43	1	1	1	1						19.55	0	1						19.29		19.29	NA	NA	
P02671	Fibrinogen alpha chain OS=Homo sapiens GN=FGA PE=1 SV=2 - [FIBA_HUMAN]	2.42	1	3	3	4	22.07			22.92		22.96	1	2	22.23			22.67		22.70	22.23	22.69	NA	NA	0.452
Q13428	Treacle protein OS=Homo sapiens GN=TCOF1 PE=1 SV=3 - [TCOF_HUMAN]	2.42	1	2	2	4				20.51	20.22	20.92	0	3				20.26	20.44	20.66		20.45	0.000	0.000	
Q5H9R7	Serine/threonine-protein phosphatase 6 regulatory subunit 3 OS=Homo sapiens GN=PPP6R3 PE=1 SV=2 - [PP6R3_HUMAN]	2.41	1	2	2	3				21.41		22.70	0	2				21.16		22.44		21.80	NA	NA	
Q14517	Protocadherin Fat 1 OS=Homo sapiens GN=FAT1 PE=1 SV=2 - [FAT1_HUMAN]	2.40	1	8	8	12	22.58	21.58	21.46				3	0	22.75	21.65	21.52				21.97		0.000	0.000	
Q9H078	Caseinolytic peptidase B protein homolog OS=Homo sapiens GN=CLPB PE=1 SV=1 - [CLPB_HUMAN]	2.40	1	1	2	2			22.25				1	0			22.31				22.31		NA	NA	
P47712	Cytosolic phospholipase A2 OS=Homo sapiens GN=PLA2G4A PE=1 SV=2 - [PA24A_HUMAN]	2.40	1	1	1	2						20.40	0	1						20.14		20.14	NA	NA	
O75175	CCR4-NOT transcription complex subunit 3 OS=Homo sapiens GN=CNOT3 PE=1 SV=1 - [CNOT3_HUMAN]	2.39	1	2	2	5		18.24		20.06	19.33	24.42	1	3		18.31		19.81	19.55	24.16	18.31	21.17	NA	NA	2.859
Q96CN4	EVI5-like protein OS=Homo sapiens GN=EVI5L PE=1 SV=1 - [EVI5L_HUMAN]	2.39	1	2	2	3		19.40		23.41		23.92	1	2		19.47		23.16		23.66	19.47	23.41	NA	NA	3.937
Q9NRY5	Protein FAM114A2 OS=Homo sapiens GN=FAM114A2 PE=1 SV=4 - [F1142_HUMAN]	2.38	1	1	1	3				16.97	18.66	18.14	0	3				16.73	18.88	17.88		17.83	0.000	0.000	
Q9Y4W6	AFG3-like protein 2 OS=Homo sapiens GN=AFG3L2 PE=1 SV=2 - [AFG32_HUMAN]	2.38	2	2	2	2					21.34		0	1					21.56			21.56	NA	NA	
Q9H2M9	Rab3 GTPase-activating protein non-catalytic subunit OS=Homo sapiens GN=RAB3GAP2 PE=1 SV=1 - [RBGPR_HUMAN]	2.37	1	2	2	3					21.83		0	1					22.04			22.04	NA	NA	
Q9NYK5	39S ribosomal protein L39, mitochondrial OS=Homo sapiens GN=MRPL39 PE=1 SV=3 - [RM39_HUMAN]	2.37	1	1	1	1			24.32				1	0			24.38				24.38		NA	NA	
Q86VE9	Serine incorporator 5 OS=Homo sapiens GN=SERINC5 PE=2 SV=1 - [SERC5_HUMAN]	2.36	1	1	1	1		23.04					1	0		23.11					23.11		NA	NA	
Q9BZE9	Tether containing UBX domain for GLUT4 OS=Homo sapiens GN=ASPSCR1 PE=1 SV=1 - [ASPC1_HUMAN]	2.35	1	1	1	1				16.80			0	1				16.55				16.55	NA	NA	
Q96PU8	Protein quaking OS=Homo sapiens GN=QKI PE=1 SV=1 - [QKI_HUMAN]	2.35	1	1	1	1					21.18		0	1					21.40			21.40	NA	NA	
O15484	Calpain-5 OS=Homo sapiens GN=CAPN5 PE=1 SV=2 - [CAN5_HUMAN]	2.34	1	1	1	1	22.69						1	0	22.85						22.85		NA	NA	
P35858	Insulin-like growth factor-binding protein complex acid labile subunit OS=Homo sapiens GN=IGFALS PE=1 SV=1 - [ALS_HUMAN]	2.31	1	1	1	2	23.17			22.96			1	1	23.33			22.72			23.33	22.72	NA	NA	-0.614
P52306	Rap1 GTPase-GDP dissociation stimulator 1 OS=Homo sapiens GN=RAP1GDS1 PE=1 SV=3 - [GDS1_HUMAN]	2.31	1	1	1	1						22.63	0	1						22.37		22.37	NA	NA	
O00267	Transcription elongation factor SPT5 OS=Homo sapiens GN=SUPT5H PE=1 SV=1 - [SPT5H_HUMAN]	2.30	1	2	2	2					21.14		0	1					21.36			21.36	NA	NA	
Q6ZV65	Protein FAM47E OS=Homo sapiens GN=FAM47E PE=2 SV=3 - [FA47E_HUMAN]	2.29	1	1	1	2		26.43	25.98				2	0		26.50	26.04				26.27		NA	NA	
Q8NBI6	Xyloside xylosyltransferase 1 OS=Homo sapiens GN=XXYLT1 PE=1 SV=1 - [XXLT1_HUMAN]	2.29	1	1	1	1					23.08		0	1					23.29			23.29	NA	NA	
Q9UHI8	A disintegrin and metalloproteinase with thrombospondin motifs 1 OS=Homo sapiens GN=ADAMTS1 PE=1 SV=4 - [ATS1_HUMAN]	2.28	1	1	1	1					21.87		0	1					22.08			22.08	NA	NA	
Q8IYA6	Cytoskeleton-associated protein 2-like OS=Homo sapiens GN=CKAP2L PE=1 SV=4 - [CKP2L_HUMAN]	2.28	1	1	1	1			20.87				1	0			20.93				20.93		NA	NA	
Q15063	Periostin OS=Homo sapiens GN=POSTN PE=1 SV=2 - [POSTN_HUMAN]	2.27	1	1	1	4	23.29		21.95		22.06		2	1	23.45		22.01		22.27		22.73	22.27	NA	NA	-0.456
O95980	Reversion-inducing cysteine-rich protein with Kazal motifs OS=Homo sapiens GN=RECK PE=1 SV=1 - [RECK_HUMAN]	2.27	1	2	2	3	20.58	21.00					2	0	20.74	21.07					20.91		NA	NA	
Q9NVX2	Notchless protein homolog 1 OS=Homo sapiens GN=NLE1 PE=1 SV=4 - [NLE1_HUMAN]	2.27	1	1	1	1				22.73			0	1				22.48				22.48	NA	NA	
O15439	Multidrug resistance-associated protein 4 OS=Homo sapiens GN=ABCC4 PE=1 SV=3 - [MRP4_HUMAN]	2.26	1	1	1	1						23.12	0	1						22.86		22.86	NA	NA	
Q96SW2	Protein cereblon OS=Homo sapiens GN=CRBN PE=1 SV=1 - [CRBN_HUMAN]	2.26	1	1	1	1						19.53	0	1						19.27		19.27	NA	NA	
P49747	Cartilage oligomeric matrix protein OS=Homo sapiens GN=COMP PE=1 SV=2 - [COMP_HUMAN]	2.25	1	1	1	3	21.55					22.83	1	1	21.71					22.57	21.71	22.57	NA	NA	0.863
Q96HP4	Oxidoreductase NAD-binding domain-containing protein 1 OS=Homo sapiens GN=OXNAD1 PE=1 SV=1 - [OXND1_HUMAN]	2.24	1	1	1	1		22.80					1	0		22.87					22.87		NA	NA	
Q96EP0	E3 ubiquitin-protein ligase RNF31 OS=Homo sapiens GN=RNF31 PE=1 SV=1 - [RNF31_HUMAN]	2.24	1	1	1	1	23.36						1	0	23.52						23.52		NA	NA	
Q9UGP8	Translocation protein SEC63 homolog OS=Homo sapiens GN=SEC63 PE=1 SV=2 - [SEC63_HUMAN]	2.24	1	1	1	1						17.17	0	1						16.91		16.91	NA	NA	
O96005	Cleft lip and palate transmembrane protein 1 OS=Homo sapiens GN=CLPTM1 PE=1 SV=1 - [CLPT1_HUMAN]	2.24	1	1	1	1						23.17	0	1						22.91		22.91	NA	NA	
Q93070	Ecto-ADP-ribosyltransferase 4 OS=Homo sapiens GN=ART4 PE=2 SV=2 - [NAR4_HUMAN]	2.23	1	1	1	3	21.80	23.42					2	0	21.96	23.49					22.72		NA	NA	
Q6ZSZ5	Rho guanine nucleotide exchange factor 18 OS=Homo sapiens GN=ARHGEF18 PE=1 SV=3 - [ARHGI_HUMAN]	2.22	1	1	1	2				22.28	21.79		0	2				22.03	22.00			22.02	NA	NA	
Q8TAF3	WD repeat-containing protein 48 OS=Homo sapiens GN=WDR48 PE=1 SV=1 - [WDR48_HUMAN]	2.22	1	1	1	1						22.06	0	1						21.80		21.80	NA	NA	
Q96Q45	Transmembrane protein 237 OS=Homo sapiens GN=TMEM237 PE=1 SV=2 - [TM237_HUMAN]	2.21	1	1	1	2		20.87				21.36	1	1		20.94				21.10	20.94	21.10	NA	NA	0.156
Q6P2E9	Enhancer of mRNA-decapping protein 4 OS=Homo sapiens GN=EDC4 PE=1 SV=1 - [EDC4_HUMAN]	2.21	1	2	2	3					22.46	21.47	0	2					22.67	21.21		21.94	NA	NA	
Q9C037	E3 ubiquitin-protein ligase TRIM4 OS=Homo sapiens GN=TRIM4 PE=1 SV=2 - [TRIM4_HUMAN]	2.20	1	1	1	2	29.02				30.57		1	1	29.18				30.79		29.18	30.79	NA	NA	1.609
Q9UKJ3	G patch domain-containing protein 8 OS=Homo sapiens GN=GPATCH8 PE=1 SV=2 - [GPTC8_HUMAN]	2.20	1	2	2	6			17.83		18.03	17.80	1	2			17.89		18.25	17.54	17.89	17.89	NA	NA	0.007
P85037	Forkhead box protein K1 OS=Homo sapiens GN=FOXK1 PE=1 SV=1 - [FOXK1_HUMAN]	2.18	1	1	1	4					17.28	17.54	0	2					17.50	17.28		17.39	NA	NA	
P78325	Disintegrin and metalloproteinase domain-containing protein 8 OS=Homo sapiens GN=ADAM8 PE=1 SV=2 - [ADAM8_HUMAN]	2.18	1	1	1	2					19.63	20.67	0	2					19.84	20.41		20.13	NA	NA	
O95396	Adenylyltransferase and sulfurtransferase MOCS3 OS=Homo sapiens GN=MOCS3 PE=1 SV=1 - [MOCS3_HUMAN]	2.17	1	1	1	1						23.58	0	1						23.32		23.32	NA	NA	
Q13671	Ras and Rab interactor 1 OS=Homo sapiens GN=RIN1 PE=1 SV=4 - [RIN1_HUMAN]	2.17	1	1	1	2			18.84			19.28	1	1			18.90			19.02	18.90	19.02	NA	NA	0.120
P13805	Troponin T, slow skeletal muscle OS=Homo sapiens GN=TNNT1 PE=1 SV=4 - [TNNT1_HUMAN]	2.16	1	1	1	2				24.85	24.28		0	2				24.60	24.49			24.55	NA	NA	
Q56NI9	N-acetyltransferase ESCO2 OS=Homo sapiens GN=ESCO2 PE=1 SV=1 - [ESCO2_HUMAN]	2.16	1	2	2	3					27.03	25.67	0	2					27.25	25.41		26.33	NA	NA	
P49336	Cyclin-dependent kinase 8 OS=Homo sapiens GN=CDK8 PE=1 SV=1 - [CDK8_HUMAN]	2.16	1	1	1	1		28.41					1	0		28.48					28.48		NA	NA	
P41143	Delta-type opioid receptor OS=Homo sapiens GN=OPRD1 PE=1 SV=4 - [OPRD_HUMAN]	2.15	1	1	1	1				22.02			0	1				21.78				21.78	NA	NA	
Q96KR7	Phosphatase and actin regulator 3 OS=Homo sapiens GN=PHACTR3 PE=1 SV=1 - [PHAR3_HUMAN]	2.15	1	1	1	1					24.72		0	1					24.93			24.93	NA	NA	
Q658Y4	Protein FAM91A1 OS=Homo sapiens GN=FAM91A1 PE=1 SV=3 - [F91A1_HUMAN]	2.15	1	1	1	1						23.74	0	1						23.48		23.48	NA	NA	
O00391	Sulfhydryl oxidase 1 OS=Homo sapiens GN=QSOX1 PE=1 SV=3 - [QSOX1_HUMAN]	2.14	1	1	1	2	20.12				20.96		1	1	20.28				21.18		20.28	21.18	NA	NA	0.898

O43298	Zinc finger and BTB domain-containing protein 43 OS=Homo sapiens GN=ZBTB43 PE=1 SV=1 - [ZBT43_HUMAN]	2.14	1	1	1	1		20.27					1	0		20.34					20.34		NA	NA	
Q99700	Ataxin-2 OS=Homo sapiens GN=ATXN2 PE=1 SV=2 - [ATX2_HUMAN]	2.13	1	2	3	6	25.84		26.56		26.54	24.93	2	2	26.00		26.62		26.76	24.67	26.31	25.72	0.641	0.206	-0.592
Q8TEA1	Putative methyltransferase NSUN6 OS=Homo sapiens GN=NSUN6 PE=1 SV=1 - [NSUN6_HUMAN]	2.13	1	1	1	1					20.61		0	1					20.83			20.83	NA	NA	
Q9H6Z4	Ran-binding protein 3 OS=Homo sapiens GN=RANBP3 PE=1 SV=1 - [RANB3_HUMAN]	2.12	1	1	1	2					20.32	21.65	0	2					20.54	21.39		20.97	NA	NA	
Q13438	Protein OS-9 OS=Homo sapiens GN=OS9 PE=1 SV=1 - [OS9_HUMAN]	2.10	1	1	1	1					21.74		0	1					21.96			21.96	NA	NA	
O00203	AP-3 complex subunit beta-1 OS=Homo sapiens GN=AP3B1 PE=1 SV=3 - [AP3B1_HUMAN]	2.10	2	2	2	2					21.20		0	1					21.42			21.42	NA	NA	
Q9P265	Disco-interacting protein 2 homolog B OS=Homo sapiens GN=DIP2B PE=1 SV=3 - [DIP2B_HUMAN]	2.09	1	2	2	2		20.99		21.75			1	1		21.06		21.50			21.06	21.50	NA	NA	0.438
Q05048	Cleavage stimulation factor subunit 1 OS=Homo sapiens GN=CSTF1 PE=1 SV=1 - [CSTF1_HUMAN]	2.09	1	1	1	2					21.00	21.59	0	2					21.22	21.33		21.27	NA	NA	
Q70E73	Ras-associated and pleckstrin homology domains-containing protein 1 OS=Homo sapiens GN=RAPH1 PE=1 SV=3 - [RAPH1_HUMAN]	2.08	1	1	2	3			22.21	21.96			1	1			22.27	21.71			22.27	21.71	NA	NA	-0.556
Q9NXX6	Non-structural maintenance of chromosomes element 4 homolog A OS=Homo sapiens GN=NSMCE4A PE=1 SV=2 - [NSE4A_HUMAN]	2.08	1	1	1	1					21.44		0	1					21.65			21.65	NA	NA	
Q8N2I9	Serine/threonine-protein kinase 40 OS=Homo sapiens GN=STK40 PE=1 SV=2 - [STK40_HUMAN]	2.07	1	1	1	1		23.26					1	0		23.32					23.32		NA	NA	
Q14781	Chromobox protein homolog 2 OS=Homo sapiens GN=CBX2 PE=1 SV=2 - [CBX2_HUMAN]	2.07	1	1	1	1					17.19		0	1					17.40			17.40	NA	NA	
Q9H8Y5	Ankyrin repeat and zinc finger domain-containing protein 1 OS=Homo sapiens GN=ANKZF1 PE=1 SV=1 - [ANKZ1_HUMAN]	2.07	1	1	1	1					20.78		0	1					21.00			21.00	NA	NA	
P0C0L4	Complement C4-A OS=Homo sapiens GN=C4A PE=1 SV=2 - [CO4A_HUMAN]	2.06	2	1	3	5	22.58			23.00		22.81	1	2	22.74			22.75		22.55	22.74	22.65	NA	NA	-0.090
O15460	Prolyl 4-hydroxylase subunit alpha-2 OS=Homo sapiens GN=P4HA2 PE=1 SV=1 - [P4HA2_HUMAN]	2.06	1	1	1	1						23.52	0	1						23.26		23.26	NA	NA	
Q5VYX0	Renalase OS=Homo sapiens GN=RNLS PE=1 SV=1 - [RNLS_HUMAN]	2.05	1	1	1	1				22.56			0	1				22.31				22.31	NA	NA	
O60658	High affinity cAMP-specific and IBMX-insensitive 3',5'-cyclic phosphodiesterase 8A OS=Homo sapiens GN=PDE8A PE=1 SV=2 - [PDE8A_HUMAN]	2.05	1	1	1	1			20.19				1	0			20.25				20.25		NA	NA	
Q9H4H8	Protein FAM83D OS=Homo sapiens GN=FAM83D PE=1 SV=3 - [FA83D_HUMAN]	2.05	1	1	1	1			18.48				1	0			18.54				18.54		NA	NA	
Q9HC62	Sentrin-specific protease 2 OS=Homo sapiens GN=SENP2 PE=1 SV=3 - [SENP2_HUMAN]	2.04	1	1	1	1			19.46				1	0			19.52				19.52		NA	NA	
Q9H8K7	Uncharacterized protein C10orf88 OS=Homo sapiens GN=C10orf88 PE=1 SV=2 - [CJ088_HUMAN]	2.02	1	1	1	1				21.87			0	1				21.62				21.62	NA	NA	
Q9H9A5	CCR4-NOT transcription complex subunit 10 OS=Homo sapiens GN=CNOT10 PE=1 SV=1 - [CNO10_HUMAN]	2.02	1	1	1	1				20.85			0	1				20.60				20.60	NA	NA	
P23942	Peripherin-2 OS=Homo sapiens GN=PRPH2 PE=1 SV=1 - [PRPH2_HUMAN]	2.02	1	1	1	2				23.29	21.98		0	2				23.05	22.20			22.62	NA	NA	
Q9UNX4	WD repeat-containing protein 3 OS=Homo sapiens GN=WDR3 PE=1 SV=1 - [WDR3_HUMAN]	2.01	1	1	1	1					22.81		0	1					23.03			23.03	NA	NA	
P09001	39S ribosomal protein L3, mitochondrial OS=Homo sapiens GN=MRPL3 PE=1 SV=1 - [RM03_HUMAN]	2.01	1	1	1	1						24.63	0	1						24.37		24.37	NA	NA	
Q13454	Tumor suppressor candidate 3 OS=Homo sapiens GN=TUSC3 PE=1 SV=1 - [TUSC3_HUMAN]	2.01	1	1	1	1						22.19	0	1						21.93		21.93	NA	NA	
P42356	Phosphatidylinositol 4-kinase alpha OS=Homo sapiens GN=PI4KA PE=1 SV=4 - [PI4KA_HUMAN]	2.00	2	4	4	6	20.54	19.33	17.55			18.89	3	1	20.70	19.40	17.61			18.63	19.24	18.63	NA	NA	-0.608
Q99459	Cell division cycle 5-like protein OS=Homo sapiens GN=CDC5L PE=1 SV=2 - [CDC5L_HUMAN]	2.00	1	1	1	5				20.38	20.61	20.09	0	3				20.14	20.82	19.82		20.26	0.000	0.000	
Q14980	Nuclear mitotic apparatus protein 1 OS=Homo sapiens GN=NUMA1 PE=1 SV=2 - [NUMA1_HUMAN]	1.99	1	3	4	6				21.83	20.17	20.12	0	3				21.59	20.38	19.85		20.61	0.000	0.000	
Q9Y5J1	U3 small nucleolar RNA-associated protein 18 homolog OS=Homo sapiens GN=UTP18 PE=1 SV=3 - [UTP18_HUMAN]	1.98	1	1	1	1				20.74			0	1				20.50				20.50	NA	NA	
Q9Y4P8	WD repeat domain phosphoinositide-interacting protein 2 OS=Homo sapiens GN=WIPI2 PE=1 SV=1 - [WIPI2_HUMAN]	1.98	1	1	1	2				22.30	22.22		0	2				22.05	22.43			22.24	NA	NA	
B9ZVM9	T-complex protein 10A homolog 2 OS=Homo sapiens GN=TCP10L2 PE=3 SV=2 - [TCP2L_HUMAN]	1.98	2	1	1	1			23.01				1	0			23.07				23.07		NA	NA	
P10398	Serine/threonine-protein kinase A-Raf OS=Homo sapiens GN=ARAF PE=1 SV=2 - [ARAF_HUMAN]	1.98	1	1	1	1						21.78	0	1						21.52		21.52	NA	NA	
Q86TG7	Retrotransposon-derived protein PEG10 OS=Homo sapiens GN=PEG10 PE=1 SV=2 - [PEG10_HUMAN]	1.98	1	1	1	1			21.79				1	0			21.84				21.84		NA	NA	
P07686	Beta-hexosaminidase subunit beta OS=Homo sapiens GN=HEXB PE=1 SV=3 - [HEXB_HUMAN]	1.98	1	1	1	1						22.73	0	1						22.47		22.47	NA	NA	
Q9BV44	THUMP domain-containing protein 3 OS=Homo sapiens GN=THUMPD3 PE=1 SV=1 - [THUM3_HUMAN]	1.97	1	1	1	1			19.75				1	0			19.81				19.81		NA	NA	
Q15714	TSC22 domain family protein 1 OS=Homo sapiens GN=TSC22D1 PE=1 SV=3 - [T22D1_HUMAN]	1.96	4	2	2	2			20.87				1	0			20.93				20.93		NA	NA	
Q99741	Cell division control protein 6 homolog OS=Homo sapiens GN=CDC6 PE=1 SV=1 - [CDC6_HUMAN]	1.96	1	1	1	3	20.56	20.94				19.69	2	1	20.72	21.01				19.43	20.87	19.43	NA	NA	-1.437
Q9H3H5	UDP-N-acetylglucosamine--dolichyl-phosphate N-acetylglucosaminephosphotransferase OS=Homo sapiens GN=DPAGT1 PE=1 SV=2 - [GPT_HUMAN]	1.96	1	1	1	2					20.69	22.10	0	2					20.91	21.84		21.37	NA	NA	
Q96N67	Dedicator of cytokinesis protein 7 OS=Homo sapiens GN=DOCK7 PE=1 SV=4 - [DOCK7_HUMAN]	1.96	1	2	2	2						22.65	0	1						22.39		22.39	NA	NA	
Q86V48	Leucine zipper protein 1 OS=Homo sapiens GN=LUZP1 PE=1 SV=2 - [LUZP1_HUMAN]	1.95	1	2	2	2				22.94	21.40		0	2				22.69	21.62			22.16	NA	NA	
O95786	Probable ATP-dependent RNA helicase DDX58 OS=Homo sapiens GN=DDX58 PE=1 SV=2 - [DDX58_HUMAN]	1.95	1	1	1	1				23.23			0	1				22.99				22.99	NA	NA	
P52948	Nuclear pore complex protein Nup98-Nup96 OS=Homo sapiens GN=NUP98 PE=1 SV=4 - [NUP98_HUMAN]	1.93	1	2	2	2						21.62	0	1						21.36		21.36	NA	NA	
Q9BW19	Kinesin-like protein KIFC1 OS=Homo sapiens GN=KIFC1 PE=1 SV=2 - [KIFC1_HUMAN]	1.93	1	1	1	1					20.77		0	1					20.99			20.99	NA	NA	
Q6Y7W6	PERQ amino acid-rich with GYF domain-containing protein 2 OS=Homo sapiens GN=GIGYF2 PE=1 SV=1 - [PERQ2_HUMAN]	1.92	1	1	1	2				22.62		22.26	0	2				22.37		22.00		22.19	NA	NA	
Q9UBT7	Alpha-catulin OS=Homo sapiens GN=CTNNAL1 PE=1 SV=2 - [CTNL1_HUMAN]	1.91	1	1	1	1		20.11					1	0		20.18					20.18		NA	NA	
Q6NUS8	UDP-glucuronosyltransferase 3A1 OS=Homo sapiens GN=UGT3A1 PE=2 SV=1 - [UD3A1_HUMAN]	1.91	1	1	1	1			23.16				1	0			23.22				23.22		NA	NA	
O14757	Serine/threonine-protein kinase Chk1 OS=Homo sapiens GN=CHEK1 PE=1 SV=2 - [CHK1_HUMAN]	1.89	1	1	1	1		20.12					1	0		20.19					20.19		NA	NA	
Q9NRC1	Suppressor of tumorigenicity 7 protein OS=Homo sapiens GN=ST7 PE=1 SV=1 - [ST7_HUMAN]	1.88	1	1	1	2				22.63	23.24		0	2				22.39	23.46			22.92	NA	NA	
Q2M389	WASH complex subunit 7 OS=Homo sapiens GN=KIAA1033 PE=1 SV=2 - [WASH7_HUMAN]	1.88	1	2	2	2						22.41	0	1						22.15		22.15	NA	NA	
Q8NBX0	Saccharopine dehydrogenase-like oxidoreductase OS=Homo sapiens GN=SCCPDH PE=1 SV=1 - [SCPDL_HUMAN]	1.86	1	1	1	1					20.85		0	1					21.06			21.06	NA	NA	
Q5C9Z4	Nucleolar MIF4G domain-containing protein 1 OS=Homo sapiens GN=NOM1 PE=1 SV=1 - [NOM1_HUMAN]	1.86	1	1	1	1					18.22		0	1					18.44			18.44	NA	NA	
Q5JTC6	APC membrane recruitment protein 1 OS=Homo sapiens GN=AMER1 PE=1 SV=2 - [AMER1_HUMAN]	1.85	1	2	2	3	20.85		20.54				2	0	21.02		20.60				20.81		NA	NA	
Q8WTW3	Conserved oligomeric Golgi complex subunit 1 OS=Homo sapiens GN=COG1 PE=1 SV=1 - [COG1_HUMAN]	1.84	1	2	2	2			16.76				1	0			16.81				16.81		NA	NA	
Q8TD55	Pleckstrin homology domain-containing family O member 2 OS=Homo sapiens GN=PLEKHO2 PE=1 SV=1 - [PKHO2_HUMAN]	1.84	1	1	1	1			19.74				1	0			19.80				19.80		NA	NA	
P49748	Very long-chain specific acyl-CoA dehydrogenase, mitochondrial OS=Homo sapiens GN=ACADVL PE=1 SV=1 - [ACADV_HUMAN]	1.83	1	1	1	2				23.11		23.43	0	2				22.86		23.17		23.02	NA	NA	
O75449	Katanin p60 ATPase-containing subunit A1 OS=Homo sapiens GN=KATNA1 PE=1 SV=1 - [KTNA1_HUMAN]	1.83	1	1	1	1					22.16		0	1					22.37			22.37	NA	NA	
P50416	Carnitine O-palmitoyltransferase 1, liver isoform OS=Homo sapiens GN=CPT1A PE=1 SV=2 - [CPT1A_HUMAN]	1.81	1	1	1	1						21.23	0	1						20.97		20.97	NA	NA	
Q9Y2J4	Angiomotin-like protein 2 OS=Homo sapiens GN=AMOTL2 PE=1 SV=3 - [AMOL2_HUMAN]	1.80	1	1	1	3	21.13	21.09	20.84				3	0	21.29	21.16	20.90				21.12		0.000	0.000	
Q9HD40	O-phosphoseryl-tRNA(Sec) selenium transferase OS=Homo sapiens GN=SEPSECS PE=1 SV=2 - [SPCS_HUMAN]	1.80	1	1	1	1						25.45	0	1						25.18		25.18	NA	NA	
Q8TD31	Coiled-coil alpha-helical rod protein 1 OS=Homo sapiens GN=CCHCR1 PE=1 SV=2 - [CCHCR_HUMAN]	1.79	1	2	2	2	21.21						1	0	21.37						21.37		NA	NA	
Q7Z2K8	G protein-regulated inducer of neurite outgrowth 1 OS=Homo sapiens GN=GPRIN1 PE=2 SV=2 - [GRIN1_HUMAN]	1.79	1	1	1	4		17.94	18.67	17.37			2	1		18.01	18.73	17.13			18.37	17.13	NA	NA	-1.242
Q14520	Hyaluronan-binding protein 2 OS=Homo sapiens GN=HABP2 PE=1 SV=1 - [HABP2_HUMAN]	1.79	1	1	1	2				23.81		22.43	0	2				23.56		22.17		22.86	NA	NA	
P00488	Coagulation factor XIII A chain OS=Homo sapiens GN=F13A1 PE=1 SV=4 - [F13A_HUMAN]	1.78	1	1	1	1						22.98	0	1						22.71		22.71	NA	NA	
Q7LC44	Activity-regulated cytoskeleton-associated protein OS=Homo sapiens GN=ARC PE=2 SV=1 - [ARC_HUMAN]	1.77	1	1	1	1					23.08		0	1					23.30			23.30	NA	NA	
Q9NZT2	Opioid growth factor receptor OS=Homo sapiens GN=OGFR PE=1 SV=3 - [OGFR_HUMAN]	1.77	1	1	1	1			19.99				1	0			20.04				20.04		NA	NA	
A2RTX5	Probable threonine--tRNA ligase 2, cytoplasmic OS=Homo sapiens GN=TARSL2 PE=1 SV=1 - [SYTC2_HUMAN]	1.75	1	1	2	4				24.53	23.35	23.84	0	3				24.28	23.57	23.58		23.81	0.000	0.000	
O00515	Ladinin-1 OS=Homo sapiens GN=LAD1 PE=1 SV=2 - [LAD1_HUMAN]	1.74	1	1	1	1					23.59		0	1					23.81			23.81	NA	NA	
Q13444	Disintegrin and metalloproteinase domain-containing protein 15 OS=Homo sapiens GN=ADAM15 PE=1 SV=4 - [ADA15_HUMAN]	1.74	1	1	1	1		21.89					1	0		21.96					21.96		NA	NA	
Q53GT1	Kelch-like protein 22 OS=Homo sapiens GN=KLHL22 PE=1 SV=2 - [KLH22_HUMAN]	1.74	1	1	1	1		24.27					1	0		24.34					24.34		NA	NA	
O76031	ATP-dependent Clp protease ATP-binding subunit clpX-like, mitochondrial OS=Homo sapiens GN=CLPX PE=1 SV=2 - [CLPX_HUMAN]	1.74	1	1	1	1					20.91		0	1					21.12			21.12	NA	NA	
Q6IQ21	Zinc finger protein 770 OS=Homo sapiens GN=ZNF770 PE=2 SV=1 - [ZN770_HUMAN]	1.74	1	1	1	1			24.74				1	0			24.80				24.80		NA	NA	
P0DJG4	Testicular haploid expressed gene protein-like OS=Homo sapiens GN=THEGL PE=2 SV=1 - [THEGL_HUMAN]	1.72	1	1	1	1		22.54					1	0		22.61					22.61		NA	NA	
P04198	N-myc proto-oncogene protein OS=Homo sapiens GN=MYCN PE=1 SV=2 - [MYCN_HUMAN]	1.72	1	1	1	2			25.23		26.37		1	1			25.28		26.58		25.28	26.58	NA	NA	1.298
Q9UH65	Switch-associated protein 70 OS=Homo sapiens GN=SWAP70 PE=1 SV=1 - [SWP70_HUMAN]	1.71	1	1	1	3				21.40		21.16	0	2				21.16		20.90		21.03	NA	NA	
P38935	DNA-binding protein SMUBP-2 OS=Homo sapiens GN=IGHMBP2 PE=1 SV=3 - [SMBP2_HUMAN]	1.71	1	1	1	1				17.27			0	1				17.03				17.03	NA	NA	
Q9Y5T5	Ubiquitin carboxyl-terminal hydrolase 16 OS=Homo sapiens GN=USP16 PE=1 SV=1 - [UBP16_HUMAN]	1.70	1	1	1	1					17.69		0	1					17.91			17.91	NA	NA	
P00966	Argininosuccinate synthase OS=Homo sapiens GN=ASS1 PE=1 SV=2 - [ASSY_HUMAN]	1.70	1	1	1	1						21.62	0	1						21.36		21.36	NA	NA	
P49757	Protein numb homolog OS=Homo sapiens GN=NUMB PE=1 SV=2 - [NUMB_HUMAN]	1.69	1	1	1	3	21.98	21.30	21.36				3	0	22.14	21.37	21.42				21.64		0.000	0.000	
P49959	Double-strand break repair protein MRE11A OS=Homo sapiens GN=MRE11A PE=1 SV=3 - [MRE11_HUMAN]	1.69	1	1	1	2					22.20	22.20	0	2					22.41	21.94		22.17	NA	NA	
Q14BN4	Sarcolemmal membrane-associated protein OS=Homo sapiens GN=SLMAP PE=1 SV=1 - [SLMAP_HUMAN]	1.69	1	1	1	2					17.81	18.79	0	2					18.03	18.53		18.28	NA	NA	
Q9Y4E1	WASH complex subunit FAM21C OS=Homo sapiens GN=FAM21C PE=1 SV=3 - [FA21C_HUMAN]	1.67	2	1	1	4				21.62	21.65	21.65	0	3				21.38	21.87	21.39		21.55	0.000	0.000	
Q96BT7	Alkylated DNA repair protein alkB homolog 8 OS=Homo sapiens GN=ALKBH8 PE=1 SV=2 - [ALKB8_HUMAN]	1.66	1	1	1	1				24.34			0	1				24.10				24.10	NA	NA	
Q8IY50	Putative thiamine transporter SLC35F3 OS=Homo sapiens GN=SLC35F3 PE=2 SV=2 - [S35F3_HUMAN]	1.66	1	1	1	2				24.18	24.28		0	2				23.94	24.49			24.22	NA	NA	
Q6GQQ9	OTU domain-containing protein 7B OS=Homo sapiens GN=OTUD7B PE=1 SV=1 - [OTU7B_HUMAN]	1.66	1	1	1	2			17.37				1	0			17.43				17.43		NA	NA	
Q8TEC5	Putative E3 ubiquitin-protein ligase SH3RF2 OS=Homo sapiens GN=SH3RF2 PE=1 SV=3 - [SH3R2_HUMAN]	1.65	1	1	1	2	20.60	20.77					2	0	20.76	20.84					20.80		NA	NA	
Q96S59	Ran-binding protein 9 OS=Homo sapiens GN=RANBP9 PE=1 SV=1 - [RANB9_HUMAN]	1.65	1	1	1	1					21.13		0	1					21.34			21.34	NA	NA	
Q8WU90	Zinc finger CCCH domain-containing protein 15 OS=Homo sapiens GN=ZC3H15 PE=1 SV=1 - [ZC3HF_HUMAN]	1.64	1	1	1	2	20.16			20.86			1	1	20.32			20.61			20.32	20.61	NA	NA	0.294
Q9C030	Tripartite motif-containing protein 6 OS=Homo sapiens GN=TRIM6 PE=1 SV=1 - [TRIM6_HUMAN]	1.64	1	1	1	1	24.30						1	0	24.46						24.46		NA	NA	
Q13823	Nucleolar GTP-binding protein 2 OS=Homo sapiens GN=GNL2 PE=1 SV=1 - [NOG2_HUMAN]	1.64	1	1	1	3				21.01	21.55	21.51	0	3				20.77	21.77	21.25		21.26	0.000	0.000	
P48995	Short transient receptor potential channel 1 OS=Homo sapiens GN=TRPC1 PE=1 SV=1 - [TRPC1_HUMAN]	1.64	1	1	2	2			23.44		22.51		1	1			23.50		22.73		23.50	22.73	NA	NA	-0.775
Q9NQS7	Inner centromere protein OS=Homo sapiens GN=INCENP PE=1 SV=3 - [INCE_HUMAN]	1.63	1	1	1	5	21.24						1	0	21.40						21.40		NA	NA	
Q9GZR7	ATP-dependent RNA helicase DDX24 OS=Homo sapiens GN=DDX24 PE=1 SV=1 - [DDX24_HUMAN]	1.63	1	1	1	2					20.45	20.51	0	2					20.66	20.24		20.45	NA	NA	
Q0ZGT2	Nexilin OS=Homo sapiens GN=NEXN PE=1 SV=1 - [NEXN_HUMAN]	1.63	1	1	1	1					20.36		0	1					20.58			20.58	NA	NA	
Q8TDW0	Volume-regulated anion channel subunit LRRC8C OS=Homo sapiens GN=LRRC8C PE=1 SV=2 - [LRC8C_HUMAN]	1.62	1	1	1	1	21.51						1	0	21.67						21.67		NA	NA	
O75387	Large neutral amino acids transporter small subunit 3 OS=Homo sapiens GN=SLC43A1 PE=1 SV=1 - [LAT3_HUMAN]	1.61	1	1	1	2	21.76	21.85					2	0	21.93	21.92					21.92		NA	NA	
P78347	General transcription factor II-I OS=Homo sapiens GN=GTF2I PE=1 SV=2 - [GTF2I_HUMAN]	1.60	1	1	1	1						21.70	0	1						21.44		21.44	NA	NA	
Q9UF83	Uncharacterized protein DKFZp434B061 OS=Homo sapiens PE=2 SV=2 - [YM012_HUMAN]	1.60	1	1	1	1						24.90	0	1						24.63		24.63	NA	NA	
Q03181	Peroxisome proliferator-activated receptor delta OS=Homo sapiens GN=PPARD PE=1 SV=1 - [PPARD_HUMAN]	1.59	1	1	1	1	20.91						1	0	21.07						21.07		NA	NA	
Q9P1Z2	Calcium-binding and coiled-coil domain-containing protein 1 OS=Homo sapiens GN=CALCOCO1 PE=1 SV=2 - [CACO1_HUMAN]	1.59	1	1	1	2			18.32		18.55		1	1			18.38		18.76		18.38	18.76	NA	NA	0.383
O60245	Protocadherin-7 OS=Homo sapiens GN=PCDH7 PE=1 SV=2 - [PCDH7_HUMAN]	1.59	1	1	1	1		18.94					1	0		19.01					19.01		NA	NA	
Q96ST2	Protein IWS1 homolog OS=Homo sapiens GN=IWS1 PE=1 SV=2 - [IWS1_HUMAN]	1.59	1	1	1	2					18.99	19.55	0	2					19.21	19.29		19.25	NA	NA	
Q14008	Cytoskeleton-associated protein 5 OS=Homo sapiens GN=CKAP5 PE=1 SV=3 - [CKAP5_HUMAN]	1.57	1	2	2	7	21.76			23.27	21.17	22.36	1	3	21.92			23.02	21.39	22.10	21.92	22.17	NA	NA	0.249
Q9UKK3	Poly [ADP-ribose] polymerase 4 OS=Homo sapiens GN=PARP4 PE=1 SV=3 - [PARP4_HUMAN]	1.57	1	2	2	3			19.34	22.04	20.37		1	2			19.40	21.80	20.58		19.40	21.19	NA	NA	1.787
Q6IA86	Elongator complex protein 2 OS=Homo sapiens GN=ELP2 PE=1 SV=2 - [ELP2_HUMAN]	1.57	1	1	1	3				20.97		21.59	0	2				20.72		21.33		21.02	NA	NA	
Q6P1M3	Lethal(2) giant larvae protein homolog 2 OS=Homo sapiens GN=LLGL2 PE=1 SV=2 - [L2GL2_HUMAN]	1.57	1	1	1	1			20.21				1	0			20.27				20.27		NA	NA	
P12109	Collagen alpha-1(VI) chain OS=Homo sapiens GN=COL6A1 PE=1 SV=3 - [CO6A1_HUMAN]	1.56	1	1	1	3	21.25		23.42	23.87			2	1	21.42		23.48	23.63			22.45	23.63	NA	NA	1.182
Q93008	Probable ubiquitin carboxyl-terminal hydrolase FAF-X OS=Homo sapiens GN=USP9X PE=1 SV=3 - [USP9X_HUMAN]	1.56	2	4	4	6			21.31	21.11		21.50	1	2			21.36	20.86		21.24	21.36	21.05	NA	NA	-0.315
O15234	Protein CASC3 OS=Homo sapiens GN=CASC3 PE=1 SV=2 - [CASC3_HUMAN]	1.56	1	1	1	1					19.41		0	1					19.63			19.63	NA	NA	
O75182	Paired amphipathic helix protein Sin3b OS=Homo sapiens GN=SIN3B PE=1 SV=2 - [SIN3B_HUMAN]	1.55	1	1	1	4				18.82	17.81	18.69	0	3				18.57	18.03	18.43		18.34	0.000	0.000	
P30837	Aldehyde dehydrogenase X, mitochondrial OS=Homo sapiens GN=ALDH1B1 PE=1 SV=3 - [AL1B1_HUMAN]	1.55	1	1	1	1					21.09		0	1					21.31			21.31	NA	NA	
Q96II8	Leucine-rich repeat and calponin homology domain-containing protein 3 OS=Homo sapiens GN=LRCH3 PE=1 SV=2 - [LRCH3_HUMAN]	1.54	1	1	1	2	20.13		20.04				2	0	20.30		20.09				20.20		NA	NA	
Q14680	Maternal embryonic leucine zipper kinase OS=Homo sapiens GN=MELK PE=1 SV=3 - [MELK_HUMAN]	1.54	1	1	1	1	20.23						1	0	20.40						20.40		NA	NA	
O75843	AP-1 complex subunit gamma-like 2 OS=Homo sapiens GN=AP1G2 PE=1 SV=1 - [AP1G2_HUMAN]	1.53	1	1	1	1			23.17				1	0			23.23				23.23		NA	NA	
P0C7X5	Zinc finger protein 806 OS=Homo sapiens GN=ZNF806 PE=3 SV=1 - [ZN806_HUMAN]	1.53	1	1	1	1			22.09				1	0			22.15				22.15		NA	NA	
Q6PD62	RNA polymerase-associated protein CTR9 homolog OS=Homo sapiens GN=CTR9 PE=1 SV=1 - [CTR9_HUMAN]	1.53	1	1	1	1						18.09	0	1						17.83		17.83	NA	NA	
Q9NWZ3	Interleukin-1 receptor-associated kinase 4 OS=Homo sapiens GN=IRAK4 PE=1 SV=1 - [IRAK4_HUMAN]	1.52	1	1	1	1					22.66		0	1					22.88			22.88	NA	NA	
Q6ZT52	Protein FAM43B OS=Homo sapiens GN=FAM43B PE=2 SV=1 - [FA43B_HUMAN]	1.52	1	1	1	1					24.04		0	1					24.26			24.26	NA	NA	
O00459	Phosphatidylinositol 3-kinase regulatory subunit beta OS=Homo sapiens GN=PIK3R2 PE=1 SV=2 - [P85B_HUMAN]	1.51	1	1	1	1						20.32	0	1						20.06		20.06	NA	NA	
O95985	DNA topoisomerase 3-beta-1 OS=Homo sapiens GN=TOP3B PE=1 SV=1 - [TOP3B_HUMAN]	1.51	1	1	1	2						25.05	0	1						24.79		24.79	NA	NA	
Q8IZ83	Aldehyde dehydrogenase family 16 member A1 OS=Homo sapiens GN=ALDH16A1 PE=1 SV=2 - [A16A1_HUMAN]	1.50	1	1	1	1	21.22						1	0	21.38						21.38		NA	NA	
P11047	Laminin subunit gamma-1 OS=Homo sapiens GN=LAMC1 PE=1 SV=3 - [LAMC1_HUMAN]	1.49	1	2	2	6	20.89	20.97		21.56		22.25	2	2	21.06	21.04		21.32		21.99	21.05	21.65	0.216	0.088	0.604
O95447	Lebercilin-like protein OS=Homo sapiens GN=LCA5L PE=1 SV=1 - [LCA5L_HUMAN]	1.49	1	1	1	2	21.79			24.25			1	1	21.95			24.01			21.95	24.01	NA	NA	2.053
Q96PC5	Melanoma inhibitory activity protein 2 OS=Homo sapiens GN=MIA2 PE=1 SV=4 - [MIA2_HUMAN]	1.49	3	1	2	3	25.60						1	0	25.76						25.76		NA	NA	
Q86YT6	E3 ubiquitin-protein ligase MIB1 OS=Homo sapiens GN=MIB1 PE=1 SV=1 - [MIB1_HUMAN]	1.49	1	1	1	2		20.10		20.37			1	1		20.17		20.13			20.17	20.13	NA	NA	-0.043
Q9NUL7	Probable ATP-dependent RNA helicase DDX28 OS=Homo sapiens GN=DDX28 PE=1 SV=2 - [DDX28_HUMAN]	1.48	1	1	1	1						24.02	0	1						23.76		23.76	NA	NA	
Q9ULS5	Transmembrane and coiled-coil domains protein 3 OS=Homo sapiens GN=TMCC3 PE=2 SV=3 - [TMCC3_HUMAN]	1.47	1	1	1	3	28.13		27.98	25.10			2	1	28.29		28.04	24.85			28.17	24.85	NA	NA	-3.311

O75122	CLIP-associating protein 2 OS=Homo sapiens GN=CLASP2 PE=1 SV=2 - [CLAP2_HUMAN]	1.47	1	1	1	1						20.51	0	1						20.25		20.25	NA	NA	
Q12769	Nuclear pore complex protein Nup160 OS=Homo sapiens GN=NUP160 PE=1 SV=3 - [NU160_HUMAN]	1.46	1	2	2	2					19.79	22.30	0	2					20.01	22.04		21.02	NA	NA	
A6NMB1	Sialic acid-binding Ig-like lectin 16 OS=Homo sapiens GN=SIGLEC16 PE=2 SV=3 - [SIG16_HUMAN]	1.46	1	1	1	1	22.97						1	0	23.13						23.13		NA	NA	
Q02952	A-kinase anchor protein 12 OS=Homo sapiens GN=AKAP12 PE=1 SV=4 - [AKA12_HUMAN]	1.46	1	1	1	1			20.49				1	0			20.55				20.55		NA	NA	
Q9Y450	HBS1-like protein OS=Homo sapiens GN=HBS1L PE=1 SV=1 - [HBS1L_HUMAN]	1.46	1	1	1	1						21.34	0	1						21.08		21.08	NA	NA	
Q6NUT3	Major facilitator superfamily domain-containing protein 12 OS=Homo sapiens GN=MFSD12 PE=1 SV=2 - [MFS12_HUMAN]	1.46	1	1	1	1						20.76	0	1						20.50		20.50	NA	NA	
Q9Y228	TRAF3-interacting JNK-activating modulator OS=Homo sapiens GN=TRAF3IP3 PE=1 SV=2 - [T3JAM_HUMAN]	1.45	1	1	1	1					24.93		0	1					25.15			25.15	NA	NA	
P32004	Neural cell adhesion molecule L1 OS=Homo sapiens GN=L1CAM PE=1 SV=2 - [L1CAM_HUMAN]	1.43	1	2	2	4	21.42	21.58					2	0	21.58	21.65					21.61		NA	NA	
P55196	Afadin OS=Homo sapiens GN=MLLT4 PE=1 SV=3 - [AFAD_HUMAN]	1.43	1	2	2	6		21.40		22.01	21.10	21.96	1	3		21.47		21.76	21.31	21.70	21.47	21.59	NA	NA	0.116
Q8IV01	Synaptotagmin-12 OS=Homo sapiens GN=SYT12 PE=1 SV=1 - [SYT12_HUMAN]	1.43	1	1	1	1			23.41				1	0			23.47				23.47		NA	NA	
O15061	Synemin OS=Homo sapiens GN=SYNM PE=1 SV=2 - [SYNEM_HUMAN]	1.41	1	2	2	5	20.93	21.32	21.25				3	0	21.09	21.39	21.31				21.26		0.000	0.000	
P05546	Heparin cofactor 2 OS=Homo sapiens GN=SERPIND1 PE=1 SV=3 - [HEP2_HUMAN]	1.40	1	1	1	1	22.48						1	0	22.64						22.64		NA	NA	
O60732	Melanoma-associated antigen C1 OS=Homo sapiens GN=MAGEC1 PE=1 SV=3 - [MAGC1_HUMAN]	1.40	1	1	1	3				21.75			0	1				21.51				21.51	NA	NA	
Q9Y561	Low-density lipoprotein receptor-related protein 12 OS=Homo sapiens GN=LRP12 PE=1 SV=1 - [LRP12_HUMAN]	1.40	1	1	1	1				22.34			0	1				22.09				22.09	NA	NA	
O75150	E3 ubiquitin-protein ligase BRE1B OS=Homo sapiens GN=RNF40 PE=1 SV=4 - [BRE1B_HUMAN]	1.40	1	1	1	1					21.39		0	1					21.61			21.61	NA	NA	
Q9UDR5	Alpha-aminoadipic semialdehyde synthase, mitochondrial OS=Homo sapiens GN=AASS PE=1 SV=1 - [AASS_HUMAN]	1.40	1	1	1	2			23.97			26.00	1	1			24.03			25.74	24.03	25.74	NA	NA	1.713
Q12979	Active breakpoint cluster region-related protein OS=Homo sapiens GN=ABR PE=2 SV=2 - [ABR_HUMAN]	1.40	1	1	1	1						21.91	0	1						21.65		21.65	NA	NA	
Q3V5L5	Alpha-1,6-mannosylglycoprotein 6-beta-N-acetylglucosaminyltransferase B OS=Homo sapiens GN=MGAT5B PE=1 SV=2 - [MGT5B_HUMAN]	1.39	1	1	1	1	23.16						1	0	23.32						23.32		NA	NA	
Q5TID7	Coiled-coil domain-containing protein 181 OS=Homo sapiens GN=CCDC181 PE=2 SV=1 - [CC181_HUMAN]	1.38	1	1	1	2			27.93		28.13		1	1			27.99		28.35		27.99	28.35	NA	NA	0.359
Q969N2	GPI transamidase component PIG-T OS=Homo sapiens GN=PIGT PE=1 SV=1 - [PIGT_HUMAN]	1.38	1	1	1	1		25.09					1	0		25.16					25.16		NA	NA	
Q92839	Hyaluronan synthase 1 OS=Homo sapiens GN=HAS1 PE=1 SV=2 - [HYAS1_HUMAN]	1.38	1	1	1	1			24.14				1	0			24.20				24.20		NA	NA	
Q9NZ08	Endoplasmic reticulum aminopeptidase 1 OS=Homo sapiens GN=ERAP1 PE=1 SV=3 - [ERAP1_HUMAN]	1.38	1	1	1	1						22.20	0	1						21.94		21.94	NA	NA	
P18583	Protein SON OS=Homo sapiens GN=SON PE=1 SV=4 - [SON_HUMAN]	1.36	1	2	2	4				20.10	21.31	21.07	0	3				19.85	21.53	20.81		20.73	0.000	0.000	
Q03518	Antigen peptide transporter 1 OS=Homo sapiens GN=TAP1 PE=1 SV=2 - [TAP1_HUMAN]	1.36	1	1	1	3				20.60	19.68	20.90	0	3				20.36	19.90	20.64		20.30	0.000	0.000	
Q14154	Death ligand signal enhancer OS=Homo sapiens GN=KIAA0141 PE=1 SV=3 - [DELE_HUMAN]	1.36	1	1	1	1				22.89			0	1				22.65				22.65	NA	NA	
Q9UQ35	Serine/arginine repetitive matrix protein 2 OS=Homo sapiens GN=SRRM2 PE=1 SV=2 - [SRRM2_HUMAN]	1.34	1	3	3	10	25.31			20.65	21.13	21.45	1	3	25.48			20.40	21.35	21.19	25.48	20.98	NA	NA	-4.496
Q8IUR7	Armadillo repeat-containing protein 8 OS=Homo sapiens GN=ARMC8 PE=1 SV=2 - [ARMC8_HUMAN]	1.34	1	1	1	2					23.37	23.51	0	2					23.58	23.25		23.42	NA	NA	
Q9Y6N7	Roundabout homolog 1 OS=Homo sapiens GN=ROBO1 PE=1 SV=1 - [ROBO1_HUMAN]	1.33	1	1	1	1	22.18						1	0	22.34						22.34		NA	NA	
Q8TD19	Serine/threonine-protein kinase Nek9 OS=Homo sapiens GN=NEK9 PE=1 SV=2 - [NEK9_HUMAN]	1.33	1	1	1	1					16.79		0	1					17.01			17.01	NA	NA	
Q5JSH3	WD repeat-containing protein 44 OS=Homo sapiens GN=WDR44 PE=1 SV=1 - [WDR44_HUMAN]	1.31	1	1	1	1	18.67						1	0	18.83						18.83		NA	NA	
O95155	Ubiquitin conjugation factor E4 B OS=Homo sapiens GN=UBE4B PE=1 SV=1 - [UBE4B_HUMAN]	1.31	1	2	2	3				20.29	22.20		0	2				20.05	22.42			21.23	NA	NA	
Q9BVR0	Putative HERC2-like protein 3 OS=Homo sapiens GN=HERC2P3 PE=5 SV=2 - [HRC23_HUMAN]	1.30	2	1	1	2				18.11		19.21	0	2				17.86		18.95		18.41	NA	NA	
Q9BZ29	Dedicator of cytokinesis protein 9 OS=Homo sapiens GN=DOCK9 PE=1 SV=2 - [DOCK9_HUMAN]	1.30	1	2	2	2		20.61				21.42	1	1		20.68				21.16	20.68	21.16	NA	NA	0.484
Q5XG87	Non-canonical poly(A) RNA polymerase PAPD7 OS=Homo sapiens GN=PAPD7 PE=1 SV=3 - [PAPD7_HUMAN]	1.30	1	1	1	1		20.81					1	0		20.88					20.88		NA	NA	
Q13618	Cullin-3 OS=Homo sapiens GN=CUL3 PE=1 SV=2 - [CUL3_HUMAN]	1.30	1	1	1	1						21.24	0	1						20.98		20.98	NA	NA	
Q13478	Interleukin-18 receptor 1 OS=Homo sapiens GN=IL18R1 PE=1 SV=1 - [IL18R_HUMAN]	1.29	1	1	1	3	24.04	23.61	22.98				3	0	24.20	23.68	23.04				23.64		0.000	0.000	
Q8TCC7	Solute carrier family 22 member 8 OS=Homo sapiens GN=SLC22A8 PE=1 SV=1 - [S22A8_HUMAN]	1.29	1	1	1	2				23.98		24.30	0	2				23.73		24.04		23.89	NA	NA	
O60306	Intron-binding protein aquarius OS=Homo sapiens GN=AQR PE=1 SV=4 - [AQR_HUMAN]	1.28	1	1	1	3				22.76	22.00		0	2				22.52	22.22			22.37	NA	NA	
Q16512	Serine/threonine-protein kinase N1 OS=Homo sapiens GN=PKN1 PE=1 SV=2 - [PKN1_HUMAN]	1.27	1	1	1	4	19.24			20.71	19.45	20.71	1	3	19.40			20.46	19.67	20.44	19.40	20.19	NA	NA	0.791
Q9NWF9	E3 ubiquitin-protein ligase RNF216 OS=Homo sapiens GN=RNF216 PE=1 SV=3 - [RN216_HUMAN]	1.27	1	1	1	2		20.02	20.25				2	0		20.09	20.31				20.20		NA	NA	
P51797	Chloride transport protein 6 OS=Homo sapiens GN=CLCN6 PE=1 SV=2 - [CLCN6_HUMAN]	1.27	1	1	1	1	24.53						1	0	24.69						24.69		NA	NA	
Q9UK99	F-box only protein 3 OS=Homo sapiens GN=FBXO3 PE=1 SV=3 - [FBX3_HUMAN]	1.27	1	1	1	1				22.64			0	1				22.39				22.39	NA	NA	
Q92922	SWI/SNF complex subunit SMARCC1 OS=Homo sapiens GN=SMARCC1 PE=1 SV=3 - [SMRC1_HUMAN]	1.27	1	1	1	1						16.85	0	1						16.59		16.59	NA	NA	
Q8TE58	A disintegrin and metalloproteinase with thrombospondin motifs 15 OS=Homo sapiens GN=ADAMTS15 PE=2 SV=1 - [ATS15_HUMAN]	1.26	1	1	1	1				22.12			0	1				21.88				21.88	NA	NA	
Q9UPN7	Serine/threonine-protein phosphatase 6 regulatory subunit 1 OS=Homo sapiens GN=PPP6R1 PE=1 SV=5 - [PP6R1_HUMAN]	1.25	1	1	1	2				21.33			0	1				21.09				21.09	NA	NA	
P30622	CAP-Gly domain-containing linker protein 1 OS=Homo sapiens GN=CLIP1 PE=1 SV=2 - [CLIP1_HUMAN]	1.25	1	2	2	3		20.20			21.63	20.87	1	2		20.27			21.85	20.61	20.27	21.23	NA	NA	0.961
Q96CU9	FAD-dependent oxidoreductase domain-containing protein 1 OS=Homo sapiens GN=FOXRED1 PE=1 SV=2 - [FXRD1_HUMAN]	1.23	1	1	1	2		26.46					1	0		26.53					26.53		NA	NA	
P48449	Lanosterol synthase OS=Homo sapiens GN=LSS PE=1 SV=1 - [ERG7_HUMAN]	1.23	1	1	1	2				20.40		21.51	0	2				20.16		21.25		20.70	NA	NA	
A0AUZ9	KAT8 regulatory NSL complex subunit 1-like protein OS=Homo sapiens GN=KANSL1L PE=1 SV=2 - [KAL1L_HUMAN]	1.22	1	1	1	1				24.24			0	1				24.00				24.00	NA	NA	
P10632	Cytochrome P450 2C8 OS=Homo sapiens GN=CYP2C8 PE=1 SV=2 - [CP2C8_HUMAN]	1.22	4	1	1	1					25.71		0	1					25.93			25.93	NA	NA	
Q92878	DNA repair protein RAD50 OS=Homo sapiens GN=RAD50 PE=1 SV=1 - [RAD50_HUMAN]	1.22	1	2	2	2					19.57		0	1					19.79			19.79	NA	NA	
Q9HCG8	Pre-mRNA-splicing factor CWC22 homolog OS=Homo sapiens GN=CWC22 PE=1 SV=3 - [CWC22_HUMAN]	1.21	1	1	1	4		18.15		20.06	20.49	20.44	1	3		18.22		19.81	20.71	20.18	18.22	20.23	NA	NA	2.012
Q9BTC0	Death-inducer obliterator 1 OS=Homo sapiens GN=DIDO1 PE=1 SV=5 - [DIDO1_HUMAN]	1.21	1	1	1	1		23.54					1	0		23.61					23.61		NA	NA	
Q8TD16	Protein bicaudal D homolog 2 OS=Homo sapiens GN=BICD2 PE=1 SV=1 - [BICD2_HUMAN]	1.21	1	1	1	2						20.99	0	1						20.73		20.73	NA	NA	
Q8N5G2	Macoilin OS=Homo sapiens GN=TMEM57 PE=1 SV=1 - [MACOI_HUMAN]	1.20	1	1	1	1						21.92	0	1						21.66		21.66	NA	NA	
O60299	Leucine zipper putative tumor suppressor 3 OS=Homo sapiens GN=LZTS3 PE=2 SV=1 - [LZTS3_HUMAN]	1.19	1	1	1	2				24.25		25.02	0	2				24.00		24.76		24.38	NA	NA	
Q8NI27	THO complex subunit 2 OS=Homo sapiens GN=THOC2 PE=1 SV=2 - [THOC2_HUMAN]	1.19	1	1	1	1				22.04			0	1				21.80				21.80	NA	NA	
Q9ULH0	Kinase D-interacting substrate of 220 kDa OS=Homo sapiens GN=KIDINS220 PE=1 SV=3 - [KDIS_HUMAN]	1.19	1	1	1	2				21.98	20.82		0	2				21.74	21.03			21.39	NA	NA	
O43719	HIV Tat-specific factor 1 OS=Homo sapiens GN=HTATSF1 PE=1 SV=1 - [HTSF1_HUMAN]	1.19	1	1	1	1					21.72		0	1					21.94			21.94	NA	NA	
Q05932	Folylpolyglutamate synthase, mitochondrial OS=Homo sapiens GN=FPGS PE=1 SV=3 - [FOLC_HUMAN]	1.19	1	1	1	2					21.91	22.77	0	2					22.13	22.51		22.32	NA	NA	
Q8N1N0	C-type lectin domain family 4 member F OS=Homo sapiens GN=CLEC4F PE=2 SV=2 - [CLC4F_HUMAN]	1.19	1	1	1	2			17.55				1	0			17.61				17.61		NA	NA	
O43156	TELO2-interacting protein 1 homolog OS=Homo sapiens GN=TTI1 PE=1 SV=3 - [TTI1_HUMAN]	1.19	1	1	1	1						21.26	0	1						20.99		20.99	NA	NA	
O43896	Kinesin-like protein KIF1C OS=Homo sapiens GN=KIF1C PE=1 SV=3 - [KIF1C_HUMAN]	1.18	3	1	1	2	19.82				20.36		1	1	19.98				20.58		19.98	20.58	NA	NA	0.602
Q6NXR4	TELO2-interacting protein 2 OS=Homo sapiens GN=TTI2 PE=1 SV=1 - [TTI2_HUMAN]	1.18	1	1	1	1						22.61	0	1						22.35		22.35	NA	NA	
Q9NXF1	Testis-expressed sequence 10 protein OS=Homo sapiens GN=TEX10 PE=1 SV=2 - [TEX10_HUMAN]	1.18	1	1	1	1		19.91					1	0		19.98					19.98		NA	NA	
Q9UBG0	C-type mannose receptor 2 OS=Homo sapiens GN=MRC2 PE=1 SV=2 - [MRC2_HUMAN]	1.15	1	1	1	2	20.55					20.94	1	1	20.71					20.68	20.71	20.68	NA	NA	-0.032
P11168	Solute carrier family 2, facilitated glucose transporter member 2 OS=Homo sapiens GN=SLC2A2 PE=1 SV=1 - [GTR2_HUMAN]	1.15	3	1	1	1	23.90						1	0	24.06						24.06		NA	NA	
Q9P215	Pogo transposable element with KRAB domain OS=Homo sapiens GN=POGK PE=1 SV=2 - [POGK_HUMAN]	1.15	1	1	1	2				24.04	23.02		0	2				23.79	23.24			23.52	NA	NA	
A4IF30	Solute carrier family 35 member F4 OS=Homo sapiens GN=SLC35F4 PE=2 SV=2 - [S35F4_HUMAN]	1.15	2	1	1	1					25.75		0	1					25.97			25.97	NA	NA	
Q8N6R0	Methyltransferase-like protein 13 OS=Homo sapiens GN=METTL13 PE=1 SV=1 - [MET13_HUMAN]	1.14	1	1	1	3				21.73	21.49	22.17	0	3				21.49	21.71	21.91		21.70	0.000	0.000	
P19878	Neutrophil cytosol factor 2 OS=Homo sapiens GN=NCF2 PE=1 SV=2 - [NCF2_HUMAN]	1.14	1	1	1	1		32.21					1	0		32.28					32.28		NA	NA	
Q96KG7	Multiple epidermal growth factor-like domains protein 10 OS=Homo sapiens GN=MEGF10 PE=1 SV=1 - [MEG10_HUMAN]	1.14	1	1	1	4			20.09		17.72		1	1			20.15		17.93		20.15	17.93	NA	NA	-2.214
Q9BU23	Lipase maturation factor 2 OS=Homo sapiens GN=LMF2 PE=1 SV=2 - [LMF2_HUMAN]	1.13	1	1	1	1						20.53	0	1						20.27		20.27	NA	NA	
O14544	Suppressor of cytokine signaling 6 OS=Homo sapiens GN=SOCS6 PE=1 SV=2 - [SOCS6_HUMAN]	1.12	1	1	1	4		25.56	25.37	27.51		27.63	2	2		25.63	25.43	27.26		27.37	25.53	27.32	0.004	0.006	1.788
Q9P2R3	Rabankyrin-5 OS=Homo sapiens GN=ANKFY1 PE=1 SV=2 - [ANFY1_HUMAN]	1.11	1	1	1	1				22.12			0	1				21.88				21.88	NA	NA	
Q8TDW5	Synaptotagmin-like protein 5 OS=Homo sapiens GN=SYTL5 PE=1 SV=1 - [SYTL5_HUMAN]	1.10	1	1	1	1	23.64						1	0	23.80						23.80		NA	NA	
O95487	Protein transport protein Sec24B OS=Homo sapiens GN=SEC24B PE=1 SV=2 - [SC24B_HUMAN]	1.10	1	1	1	3				21.56	20.64	21.74	0	3				21.32	20.85	21.47		21.21	0.000	0.000	
Q96PP9	Guanylate-binding protein 4 OS=Homo sapiens GN=GBP4 PE=2 SV=2 - [GBP4_HUMAN]	1.09	1	1	1	1	22.17						1	0	22.33						22.33		NA	NA	
O95382	Mitogen-activated protein kinase kinase kinase 6 OS=Homo sapiens GN=MAP3K6 PE=1 SV=3 - [M3K6_HUMAN]	1.09	1	1	1	2	24.33			23.43			1	1	24.50			23.19			24.50	23.19	NA	NA	-1.309
Q96AX9	E3 ubiquitin-protein ligase MIB2 OS=Homo sapiens GN=MIB2 PE=1 SV=3 - [MIB2_HUMAN]	1.09	1	1	1	1		22.93					1	0		23.00					23.00		NA	NA	
Q9BVP2	Guanine nucleotide-binding protein-like 3 OS=Homo sapiens GN=GNL3 PE=1 SV=2 - [GNL3_HUMAN]	1.09	1	1	1	1					21.87		0	1					22.09			22.09	NA	NA	
Q96R06	Sperm-associated antigen 5 OS=Homo sapiens GN=SPAG5 PE=1 SV=2 - [SPAG5_HUMAN]	1.09	1	1	1	1						20.58	0	1						20.32		20.32	NA	NA	
O95197	Reticulon-3 OS=Homo sapiens GN=RTN3 PE=1 SV=2 - [RTN3_HUMAN]	1.07	1	1	1	7	22.13	23.17	22.92		22.19		3	1	22.29	23.24	22.98		22.40		22.84	22.40	NA	NA	-0.437
Q9NNW5	WD repeat-containing protein 6 OS=Homo sapiens GN=WDR6 PE=1 SV=1 - [WDR6_HUMAN]	1.07	1	1	1	1	20.35						1	0	20.51						20.51		NA	NA	
Q12986	Transcriptional repressor NF-X1 OS=Homo sapiens GN=NFX1 PE=1 SV=2 - [NFX1_HUMAN]	1.07	1	1	1	1				20.13			0	1				19.89				19.89	NA	NA	
Q5MIZ7	Serine/threonine-protein phosphatase 4 regulatory subunit 3B OS=Homo sapiens GN=SMEK2 PE=1 SV=2 - [P4R3B_HUMAN]	1.06	1	1	1	1	25.12						1	0	25.28						25.28		NA	NA	
O00408	cGMP-dependent 3',5'-cyclic phosphodiesterase OS=Homo sapiens GN=PDE2A PE=1 SV=1 - [PDE2A_HUMAN]	1.06	1	1	1	1	22.83						1	0	22.99						22.99		NA	NA	
P10253	Lysosomal alpha-glucosidase OS=Homo sapiens GN=GAA PE=1 SV=4 - [LYAG_HUMAN]	1.05	1	1	1	5	19.85	20.20		20.89		21.15	2	2	20.02	20.27		20.64		20.89	20.14	20.77	0.071	0.039	0.625
Q9UDT6	CAP-Gly domain-containing linker protein 2 OS=Homo sapiens GN=CLIP2 PE=1 SV=1 - [CLIP2_HUMAN]	1.05	1	1	1	1				22.49			0	1				22.25				22.25	NA	NA	
P52732	Kinesin-like protein KIF11 OS=Homo sapiens GN=KIF11 PE=1 SV=2 - [KIF11_HUMAN]	1.04	1	1	1	1				21.47			0	1				21.22				21.22	NA	NA	
Q5TBA9	Protein furry homolog OS=Homo sapiens GN=FRY PE=1 SV=1 - [FRY_HUMAN]	1.03	1	2	2	2	23.05						1	0	23.21						23.21		NA	NA	
Q9Y3P9	Rab GTPase-activating protein 1 OS=Homo sapiens GN=RABGAP1 PE=1 SV=3 - [RBGP1_HUMAN]	1.03	1	1	1	2				20.56		20.78	0	2				20.31		20.52		20.42	NA	NA	
Q8IWU6	Extracellular sulfatase Sulf-1 OS=Homo sapiens GN=SULF1 PE=1 SV=1 - [SULF1_HUMAN]	1.03	1	1	1	1		27.40					1	0		27.46					27.46		NA	NA	
Q01780	Exosome component 10 OS=Homo sapiens GN=EXOSC10 PE=1 SV=2 - [EXOSX_HUMAN]	1.02	1	1	1	2					20.42	20.86	0	2					20.64	20.60		20.62	NA	NA	
Q70CQ1	Ubiquitin carboxyl-terminal hydrolase 49 OS=Homo sapiens GN=USP49 PE=1 SV=1 - [UBP49_HUMAN]	1.02	1	1	1	1					26.95		0	1					27.17			27.17	NA	NA	
O75808	Calpain-15 OS=Homo sapiens GN=CAPN15 PE=1 SV=1 - [CAN15_HUMAN]	1.01	1	1	1	1		19.92					1	0		19.99					19.99		NA	NA	
Q8NI35	InaD-like protein OS=Homo sapiens GN=INADL PE=1 SV=3 - [INADL_HUMAN]	1.00	1	1	2	5	26.82		26.58	27.98	27.14		2	2	26.98		26.64	27.74	27.35		26.81	27.55	0.103	0.051	0.734
Q9BVL2	Nucleoporin p58/p45 OS=Homo sapiens GN=NUPL1 PE=1 SV=1 - [NUPL1_HUMAN]	1.00	1	1	1	1				23.68			0	1				23.43				23.43	NA	NA	
A9Z1Z3	Fer-1-like protein 4 OS=Homo sapiens GN=FER1L4 PE=2 SV=1 - [FR1L4_HUMAN]	1.00	1	2	3	5				22.31	22.48	22.30	0	3				22.07	22.69	22.04		22.27	0.000	0.000	
Q5TB30	DEP domain-containing protein 1A OS=Homo sapiens GN=DEPDC1 PE=1 SV=2 - [DEP1A_HUMAN]	0.99	1	1	1	1	21.17						1	0	21.33						21.33		NA	NA	
O60765	Zinc finger protein 354A OS=Homo sapiens GN=ZNF354A PE=2 SV=2 - [Z354A_HUMAN]	0.99	1	1	1	2				23.21		23.43	0	2				22.96		23.17		23.07	NA	NA	
Q9NSE4	Isoleucine--tRNA ligase, mitochondrial OS=Homo sapiens GN=IARS2 PE=1 SV=2 - [SYIM_HUMAN]	0.99	1	1	1	1						21.82	0	1						21.56		21.56	NA	NA	
Q7Z3K3	Pogo transposable element with ZNF domain OS=Homo sapiens GN=POGZ PE=1 SV=2 - [POGZ_HUMAN]	0.99	1	1	1	1						18.84	0	1						18.58		18.58	NA	NA	
Q8N4X5	Actin filament-associated protein 1-like 2 OS=Homo sapiens GN=AFAP1L2 PE=1 SV=1 - [AF1L2_HUMAN]	0.98	1	1	1	1	22.34						1	0	22.50						22.50		NA	NA	
P10275	Androgen receptor OS=Homo sapiens GN=AR PE=1 SV=3 - [ANDR_HUMAN]	0.98	1	1	1	1	24.92						1	0	25.09						25.09		NA	NA	
A0FGR8	Extended synaptotagmin-2 OS=Homo sapiens GN=ESYT2 PE=1 SV=1 - [ESYT2_HUMAN]	0.98	1	1	1	1			20.94				1	0			21.00				21.00		NA	NA	
Q13085	Acetyl-CoA carboxylase 1 OS=Homo sapiens GN=ACACA PE=1 SV=2 - [ACACA_HUMAN]	0.98	2	1	1	1						23.47	0	1						23.21		23.21	NA	NA	
A6NKT7	RanBP2-like and GRIP domain-containing protein 3 OS=Homo sapiens GN=RGPD3 PE=3 SV=2 - [RGPD3_HUMAN]	0.97	5	1	1	2				17.91		17.94	0	2				17.67		17.68		17.67	NA	NA	
Q9BZQ6	ER degradation-enhancing alpha-mannosidase-like protein 3 OS=Homo sapiens GN=EDEM3 PE=1 SV=2 - [EDEM3_HUMAN]	0.97	1	1	1	3		25.41		25.23	24.13		1	2		25.48		24.99	24.34		25.48	24.66	NA	NA	-0.819
Q13061	Triadin OS=Homo sapiens GN=TRDN PE=1 SV=4 - [TRDN_HUMAN]	0.96	1	1	1	1				24.25			0	1				24.00				24.00	NA	NA	
Q99570	Phosphoinositide 3-kinase regulatory subunit 4 OS=Homo sapiens GN=PIK3R4 PE=1 SV=3 - [PI3R4_HUMAN]	0.96	1	1	1	1					21.15		0	1					21.36			21.36	NA	NA	
Q4ZHG4	Fibronectin type III domain-containing protein 1 OS=Homo sapiens GN=FNDC1 PE=2 SV=4 - [FNDC1_HUMAN]	0.95	1	1	1	3					24.87		0	1					25.09			25.09	NA	NA	
Q5T0Z8	Uncharacterized protein C6orf132 OS=Homo sapiens GN=C6orf132 PE=1 SV=4 - [CF132_HUMAN]	0.93	1	1	1	2	24.10				24.43		1	1	24.26				24.65		24.26	24.65	NA	NA	0.384
O60524	Nuclear export mediator factor NEMF OS=Homo sapiens GN=NEMF PE=1 SV=4 - [NEMF_HUMAN]	0.93	1	1	1	2						21.22	0	1						20.96		20.96	NA	NA	
O75153	Clustered mitochondria protein homolog OS=Homo sapiens GN=CLUH PE=1 SV=2 - [CLU_HUMAN]	0.92	1	1	1	2				23.55			0	1				23.30				23.30	NA	NA	
Q01826	DNA-binding protein SATB1 OS=Homo sapiens GN=SATB1 PE=1 SV=1 - [SATB1_HUMAN]	0.92	1	1	1	2	22.80		22.06				2	0	22.96		22.12				22.54		NA	NA	
Q70J99	Protein unc-13 homolog D OS=Homo sapiens GN=UNC13D PE=1 SV=1 - [UN13D_HUMAN]	0.92	1	1	1	1			22.08				1	0			22.14				22.14		NA	NA	
Q6ZSB9	Zinc finger and BTB domain-containing protein 49 OS=Homo sapiens GN=ZBTB49 PE=1 SV=3 - [ZBT49_HUMAN]	0.92	1	1	1	1						23.92	0	1						23.66		23.66	NA	NA	
O15049	NEDD4-binding protein 3 OS=Homo sapiens GN=N4BP3 PE=1 SV=3 - [N4BP3_HUMAN]	0.92	1	1	1	1						22.99	0	1						22.73		22.73	NA	NA	
P82094	TATA element modulatory factor OS=Homo sapiens GN=TMF1 PE=1 SV=2 - [TMF1_HUMAN]	0.91	1	1	1	1					19.70		0	1					19.92			19.92	NA	NA	
Q96NG3	Tetratricopeptide repeat protein 25 OS=Homo sapiens GN=TTC25 PE=1 SV=2 - [TTC25_HUMAN]	0.89	1	1	1	1					23.03		0	1					23.25			23.25	NA	NA	
Q6ZMW3	Echinoderm microtubule-associated protein-like 6 OS=Homo sapiens GN=EML6 PE=2 SV=2 - [EMAL6_HUMAN]	0.87	1	2	2	2				24.29	21.30		0	2				24.04	21.52			22.78	NA	NA	
Q9Y592	Centrosomal protein of 83 kDa OS=Homo sapiens GN=CEP83 PE=1 SV=2 - [CEP83_HUMAN]	0.87	1	1	1	1			28.53				1	0			28.59				28.59		NA	NA	
O14617	AP-3 complex subunit delta-1 OS=Homo sapiens GN=AP3D1 PE=1 SV=1 - [AP3D1_HUMAN]	0.87	1	1	1	1						22.05	0	1						21.79		21.79	NA	NA	
Q9UHF7	Zinc finger transcription factor Trps1 OS=Homo sapiens GN=TRPS1 PE=1 SV=2 - [TRPS1_HUMAN]	0.86	1	1	1	3			22.00				1	0			22.06				22.06		NA	NA	
Q9NSY1	BMP-2-inducible protein kinase OS=Homo sapiens GN=BMP2K PE=1 SV=2 - [BMP2K_HUMAN]	0.86	1	1	1	1						19.97	0	1						19.71		19.71	NA	NA	

P51532	Transcription activator BRG1 OS=Homo sapiens GN=SMARCA4 PE=1 SV=2 - [SMCA4_HUMAN]	0.85	1	1	1	2				19.46	19.71		0	2				19.22	19.93			19.57	NA	NA	
Q8N3D4	EH domain-binding protein 1-like protein 1 OS=Homo sapiens GN=EHBP1L1 PE=1 SV=2 - [EH1L1_HUMAN]	0.85	1	1	1	2			18.92				1	0			18.98				18.98		NA	NA	
Q5SY16	Polynucleotide 5'-hydroxyl-kinase NOL9 OS=Homo sapiens GN=NOL9 PE=1 SV=1 - [NOL9_HUMAN]	0.85	1	1	1	1						23.25	0	1						22.99		22.99	NA	NA	
Q96DN5	TBC1 domain family member 31 OS=Homo sapiens GN=TBC1D31 PE=2 SV=2 - [TBC31_HUMAN]	0.84	1	1	1	1		21.05					1	0		21.12					21.12		NA	NA	
Q92503	SEC14-like protein 1 OS=Homo sapiens GN=SEC14L1 PE=1 SV=2 - [S14L1_HUMAN]	0.84	1	1	1	2		30.63					1	0		30.70					30.70		NA	NA	
Q13753	Laminin subunit gamma-2 OS=Homo sapiens GN=LAMC2 PE=1 SV=2 - [LAMC2_HUMAN]	0.84	1	1	1	1						21.18	0	1						20.92		20.92	NA	NA	
Q4KWH8	1-phosphatidylinositol 4,5-bisphosphate phosphodiesterase eta-1 OS=Homo sapiens GN=PLCH1 PE=1 SV=1 - [PLCH1_HUMAN]	0.83	1	2	2	7	22.04	22.22			25.22	24.92	2	2	22.20	22.29			25.44	24.66	22.24	25.05	0.019	0.016	2.805
Q68CZ2	Tensin-3 OS=Homo sapiens GN=TNS3 PE=1 SV=2 - [TENS3_HUMAN]	0.83	1	1	1	1			22.05				1	0			22.11				22.11		NA	NA	
Q5JSL3	Dedicator of cytokinesis protein 11 OS=Homo sapiens GN=DOCK11 PE=1 SV=2 - [DOC11_HUMAN]	0.82	1	1	1	1	18.46						1	0	18.63						18.63		NA	NA	
Q96RS0	Trimethylguanosine synthase OS=Homo sapiens GN=TGS1 PE=1 SV=3 - [TGS1_HUMAN]	0.82	1	1	1	1						25.60	0	1						25.34		25.34	NA	NA	
Q6ZN28	Metastasis-associated in colon cancer protein 1 OS=Homo sapiens GN=MACC1 PE=1 SV=2 - [MACC1_HUMAN]	0.82	1	1	1	1		22.15					1	0		22.22					22.22		NA	NA	
Q9H7F0	Probable cation-transporting ATPase 13A3 OS=Homo sapiens GN=ATP13A3 PE=1 SV=4 - [AT133_HUMAN]	0.82	1	1	1	2		20.73				21.61	1	1		20.80				21.35	20.80	21.35	NA	NA	0.546
Q6PJI9	WD repeat-containing protein 59 OS=Homo sapiens GN=WDR59 PE=1 SV=2 - [WDR59_HUMAN]	0.82	1	1	1	4		23.44	26.09			26.07	2	1		23.51	26.14			25.80	24.83	25.80	NA	NA	0.976
Q9UKY4	Protein O-mannosyl-transferase 2 OS=Homo sapiens GN=POMT2 PE=1 SV=2 - [POMT2_HUMAN]	0.80	1	1	1	1				24.13			0	1				23.88				23.88	NA	NA	
Q14684	Ribosomal RNA processing protein 1 homolog B OS=Homo sapiens GN=RRP1B PE=1 SV=3 - [RRP1B_HUMAN]	0.79	1	1	1	1	22.87						1	0	23.03						23.03		NA	NA	
Q96JK4	HHIP-like protein 1 OS=Homo sapiens GN=HHIPL1 PE=2 SV=2 - [HIPL1_HUMAN]	0.77	1	1	1	1					23.97		0	1					24.19			24.19	NA	NA	
Q9BXU7	Ubiquitin carboxyl-terminal hydrolase 26 OS=Homo sapiens GN=USP26 PE=1 SV=1 - [UBP26_HUMAN]	0.77	1	1	1	1						22.26	0	1						22.00		22.00	NA	NA	
O14522	Receptor-type tyrosine-protein phosphatase T OS=Homo sapiens GN=PTPRT PE=1 SV=6 - [PTPRT_HUMAN]	0.76	2	1	1	1				22.05			0	1				21.80				21.80	NA	NA	
Q92621	Nuclear pore complex protein Nup205 OS=Homo sapiens GN=NUP205 PE=1 SV=3 - [NU205_HUMAN]	0.75	1	1	1	1					21.07		0	1					21.29			21.29	NA	NA	
Q8IVL0	Neuron navigator 3 OS=Homo sapiens GN=NAV3 PE=1 SV=3 - [NAV3_HUMAN]	0.75	1	2	2	3			22.66	22.45			1	1			22.72	22.20			22.72	22.20	NA	NA	-0.517
Q86X52	Chondroitin sulfate synthase 1 OS=Homo sapiens GN=CHSY1 PE=1 SV=3 - [CHSS1_HUMAN]	0.75	2	1	1	1					21.30		0	1					21.51			21.51	NA	NA	
Q7LBC6	Lysine-specific demethylase 3B OS=Homo sapiens GN=KDM3B PE=1 SV=2 - [KDM3B_HUMAN]	0.74	5	1	2	2		24.37			21.69		1	1		24.44			21.90		24.44	21.90	NA	NA	-2.533
Q16099	Glutamate receptor ionotropic, kainate 4 OS=Homo sapiens GN=GRIK4 PE=2 SV=2 - [GRIK4_HUMAN]	0.73	2	1	1	1						25.11	0	1						24.85		24.85	NA	NA	
P55265	Double-stranded RNA-specific adenosine deaminase OS=Homo sapiens GN=ADAR PE=1 SV=4 - [DSRAD_HUMAN]	0.73	1	1	1	1					24.06		0	1					24.27			24.27	NA	NA	
Q5VV67	Peroxisome proliferator-activated receptor gamma coactivator-related protein 1 OS=Homo sapiens GN=PPRC1 PE=1 SV=1 - [PPRC1_HUMAN]	0.72	1	1	1	1		21.68					1	0		21.75					21.75		NA	NA	
Q6N043	Zinc finger protein 280D OS=Homo sapiens GN=ZNF280D PE=1 SV=3 - [Z280D_HUMAN]	0.72	1	1	1	1	23.31						1	0	23.47						23.47		NA	NA	
Q53R41	FAST kinase domain-containing protein 1 OS=Homo sapiens GN=FASTKD1 PE=1 SV=1 - [FAKD1_HUMAN]	0.71	1	1	1	2	21.98		21.96				2	0	22.14		22.02				22.08		NA	NA	
O43847	Nardilysin OS=Homo sapiens GN=NRD1 PE=1 SV=2 - [NRDC_HUMAN]	0.70	1	1	1	1						22.66	0	1						22.40		22.40	NA	NA	
B2RC85	Radial spoke head 10 homolog B2 OS=Homo sapiens GN=RSPH10B2 PE=2 SV=2 - [R10B2_HUMAN]	0.69	2	1	1	3	23.46	22.96	23.04				3	0	23.62	23.03	23.10				23.25		0.000	0.000	
O75578	Integrin alpha-10 OS=Homo sapiens GN=ITGA10 PE=2 SV=2 - [ITA10_HUMAN]	0.69	1	1	1	1					24.32		0	1					24.53			24.53	NA	NA	
P11532	Dystrophin OS=Homo sapiens GN=DMD PE=1 SV=3 - [DMD_HUMAN]	0.68	1	2	3	4	25.46		22.17	22.79	22.40		2	2	25.62		22.23	22.55	22.61		23.92	22.58	0.511	0.173	-1.344
O75581	Low-density lipoprotein receptor-related protein 6 OS=Homo sapiens GN=LRP6 PE=1 SV=2 - [LRP6_HUMAN]	0.68	1	1	1	2	19.87		19.48				2	0	20.04		19.54				19.79		NA	NA	
P04114	Apolipoprotein B-100 OS=Homo sapiens GN=APOB PE=1 SV=2 - [APOB_HUMAN]	0.66	1	3	3	4	22.77			23.03	22.58		1	2	22.94			22.79	22.79		22.94	22.79	NA	NA	-0.145
P42261	Glutamate receptor 1 OS=Homo sapiens GN=GRIA1 PE=1 SV=2 - [GRIA1_HUMAN]	0.66	1	1	1	2	21.28			22.01			1	1	21.45			21.76			21.45	21.76	NA	NA	0.317
Q2NKJ3	CST complex subunit CTC1 OS=Homo sapiens GN=CTC1 PE=1 SV=2 - [CTC1_HUMAN]	0.66	1	1	1	1			21.60				1	0			21.66				21.66		NA	NA	
B1ANS9	WD repeat-containing protein 64 OS=Homo sapiens GN=WDR64 PE=2 SV=1 - [WDR64_HUMAN]	0.65	1	1	1	1						24.79	0	1						24.52		24.52	NA	NA	
Q6ZU35	Uncharacterized protein KIAA1211 OS=Homo sapiens GN=KIAA1211 PE=1 SV=3 - [K1211_HUMAN]	0.65	1	1	1	1		22.33					1	0		22.40					22.40		NA	NA	
Q6UY14	ADAMTS-like protein 4 OS=Homo sapiens GN=ADAMTSL4 PE=1 SV=2 - [ATL4_HUMAN]	0.65	1	1	1	3		30.03	22.97			27.84	2	1		30.10	23.02			27.58	26.56	27.58	NA	NA	1.022
Q9H270	Vacuolar protein sorting-associated protein 11 homolog OS=Homo sapiens GN=VPS11 PE=1 SV=1 - [VPS11_HUMAN]	0.64	1	1	1	1		22.41					1	0		22.48					22.48		NA	NA	
Q7Z6I6	Rho GTPase-activating protein 30 OS=Homo sapiens GN=ARHGAP30 PE=1 SV=3 - [RHG30_HUMAN]	0.64	1	1	1	1			22.63				1	0			22.69				22.69		NA	NA	
Q96QP1	Alpha-protein kinase 1 OS=Homo sapiens GN=ALPK1 PE=2 SV=3 - [ALPK1_HUMAN]	0.64	1	1	1	1						21.86	0	1						21.60		21.60	NA	NA	
Q9P278	Folliculin-interacting protein 2 OS=Homo sapiens GN=FNIP2 PE=1 SV=2 - [FNIP2_HUMAN]	0.63	1	1	1	1						25.03	0	1						24.76		24.76	NA	NA	
Q8IZF6	Adhesion G-protein coupled receptor G4 OS=Homo sapiens GN=ADGRG4 PE=2 SV=2 - [AGRG4_HUMAN]	0.62	1	2	2	2	23.46	24.32					2	0	23.62	24.39					24.01		NA	NA	
O60237	Protein phosphatase 1 regulatory subunit 12B OS=Homo sapiens GN=PPP1R12B PE=1 SV=2 - [MYPT2_HUMAN]	0.61	1	1	1	1	22.22						1	0	22.38						22.38		NA	NA	
P01031	Complement C5 OS=Homo sapiens GN=C5 PE=1 SV=4 - [CO5_HUMAN]	0.60	1	1	1	1						22.46	0	1						22.20		22.20	NA	NA	
Q14289	Protein-tyrosine kinase 2-beta OS=Homo sapiens GN=PTK2B PE=1 SV=2 - [FAK2_HUMAN]	0.59	1	1	1	3	21.84		22.81	22.18			2	1	22.01		22.87	21.94			22.44	21.94	NA	NA	-0.503
O95425	Supervillin OS=Homo sapiens GN=SVIL PE=1 SV=2 - [SVIL_HUMAN]	0.59	1	1	1	3	20.29			20.46		20.89	1	2	20.45			20.21		20.62	20.45	20.42	NA	NA	-0.034
O00443	Phosphatidylinositol 4-phosphate 3-kinase C2 domain-containing subunit alpha OS=Homo sapiens GN=PIK3C2A PE=1 SV=2 - [P3C2A_HUMAN]	0.59	1	1	1	1						21.02	0	1						20.76		20.76	NA	NA	
Q9ULU4	Protein kinase C-binding protein 1 OS=Homo sapiens GN=ZMYND8 PE=1 SV=2 - [PKCB1_HUMAN]	0.59	1	1	1	2		24.17	25.16				2	0		24.24	25.22				24.73		NA	NA	
Q9ULU8	Calcium-dependent secretion activator 1 OS=Homo sapiens GN=CADPS PE=1 SV=3 - [CAPS1_HUMAN]	0.59	1	1	1	1		22.03					1	0		22.10					22.10		NA	NA	
Q14999	Cullin-7 OS=Homo sapiens GN=CUL7 PE=1 SV=2 - [CUL7_HUMAN]	0.59	1	1	1	2			20.77			21.01	1	1			20.83			20.74	20.83	20.74	NA	NA	-0.082
Q86UK0	ATP-binding cassette sub-family A member 12 OS=Homo sapiens GN=ABCA12 PE=1 SV=3 - [ABCAC_HUMAN]	0.58	1	1	2	2			26.51				1	0			26.57				26.57		NA	NA	
Q9UQ26	Regulating synaptic membrane exocytosis protein 2 OS=Homo sapiens GN=RIMS2 PE=1 SV=2 - [RIMS2_HUMAN]	0.57	1	1	1	1			22.63				1	0			22.69				22.69		NA	NA	
Q7Z6G8	Ankyrin repeat and sterile alpha motif domain-containing protein 1B OS=Homo sapiens GN=ANKS1B PE=1 SV=2 - [ANS1B_HUMAN]	0.56	1	1	1	3		23.41		23.47		23.96	1	2		23.48		23.22		23.70	23.48	23.46	NA	NA	-0.016
A6NHR9	Structural maintenance of chromosomes flexible hinge domain-containing protein 1 OS=Homo sapiens GN=SMCHD1 PE=1 SV=2 - [SMHD1_HUMAN]	0.55	1	1	1	1					21.06		0	1					21.28			21.28	NA	NA	
Q9UPV0	Centrosomal protein of 164 kDa OS=Homo sapiens GN=CEP164 PE=1 SV=3 - [CE164_HUMAN]	0.55	1	1	1	2	28.66		28.43				2	0	28.82		28.49				28.65		NA	NA	
Q15386	Ubiquitin-protein ligase E3C OS=Homo sapiens GN=UBE3C PE=1 SV=3 - [UBE3C_HUMAN]	0.55	1	1	1	1						22.57	0	1						22.31		22.31	NA	NA	
O14976	Cyclin-G-associated kinase OS=Homo sapiens GN=GAK PE=1 SV=2 - [GAK_HUMAN]	0.53	1	1	1	1	22.76						1	0	22.93						22.93		NA	NA	
Q96Q15	Serine/threonine-protein kinase SMG1 OS=Homo sapiens GN=SMG1 PE=1 SV=3 - [SMG1_HUMAN]	0.52	2	2	2	3					22.13	23.57	0	2					22.34	23.31		22.83	NA	NA	
P12111	Collagen alpha-3(VI) chain OS=Homo sapiens GN=COL6A3 PE=1 SV=5 - [CO6A3_HUMAN]	0.50	1	1	1	2	19.86			19.52			1	1	20.02			19.28			20.02	19.28	NA	NA	-0.740
P12107	Collagen alpha-1(XI) chain OS=Homo sapiens GN=COL11A1 PE=1 SV=4 - [COBA1_HUMAN]	0.50	1	1	1	1			19.89				1	0			19.94				19.94		NA	NA	
Q6RI45	Bromodomain and WD repeat-containing protein 3 OS=Homo sapiens GN=BRWD3 PE=1 SV=2 - [BRWD3_HUMAN]	0.50	1	1	1	1			25.19				1	0			25.25				25.25		NA	NA	
Q96BY6	Dedicator of cytokinesis protein 10 OS=Homo sapiens GN=DOCK10 PE=1 SV=3 - [DOC10_HUMAN]	0.50	1	1	1	1			28.40				1	0			28.46				28.46		NA	NA	
Q9ULT8	E3 ubiquitin-protein ligase HECTD1 OS=Homo sapiens GN=HECTD1 PE=1 SV=3 - [HECD1_HUMAN]	0.50	1	1	1	1						21.10	0	1						20.84		20.84	NA	NA	
O75037	Kinesin-like protein KIF21B OS=Homo sapiens GN=KIF21B PE=1 SV=2 - [KI21B_HUMAN]	0.49	1	1	1	1				24.69			0	1				24.44				24.44	NA	NA	
Q92608	Dedicator of cytokinesis protein 2 OS=Homo sapiens GN=DOCK2 PE=1 SV=2 - [DOCK2_HUMAN]	0.49	1	1	1	1			22.28				1	0			22.34				22.34		NA	NA	
Q6ZP82	Coiled-coil domain-containing protein 141 OS=Homo sapiens GN=CCDC141 PE=1 SV=2 - [CC141_HUMAN]	0.48	1	1	1	1			30.24				1	0			30.30				30.30		NA	NA	
P25054	Adenomatous polyposis coli protein OS=Homo sapiens GN=APC PE=1 SV=2 - [APC_HUMAN]	0.46	1	1	1	1		18.17					1	0		18.24					18.24		NA	NA	
Q99683	Mitogen-activated protein kinase kinase kinase 5 OS=Homo sapiens GN=MAP3K5 PE=1 SV=1 - [M3K5_HUMAN]	0.44	1	1	1	1		24.39					1	0		24.46					24.46		NA	NA	
Q9Y6R4	Mitogen-activated protein kinase kinase kinase 4 OS=Homo sapiens GN=MAP3K4 PE=1 SV=2 - [M3K4_HUMAN]	0.44	1	1	1	1						25.57	0	1						25.31		25.31	NA	NA	
P35125	Ubiquitin carboxyl-terminal hydrolase 6 OS=Homo sapiens GN=USP6 PE=1 SV=2 - [UBP6_HUMAN]	0.43	1	1	1	1				26.77			0	1				26.52				26.52	NA	NA	
P48681	Nestin OS=Homo sapiens GN=NES PE=1 SV=2 - [NEST_HUMAN]	0.43	1	1	1	2		23.36	23.20				2	0		23.43	23.26				23.34		NA	NA	
Q14191	Werner syndrome ATP-dependent helicase OS=Homo sapiens GN=WRN PE=1 SV=2 - [WRN_HUMAN]	0.42	2	1	1	1						22.33	0	1						22.07		22.07	NA	NA	
P09884	DNA polymerase alpha catalytic subunit OS=Homo sapiens GN=POLA1 PE=1 SV=2 - [DPOLA_HUMAN]	0.41	1	1	1	1		26.04					1	0		26.11					26.11		NA	NA	
Q9P2D1	Chromodomain-helicase-DNA-binding protein 7 OS=Homo sapiens GN=CHD7 PE=1 SV=3 - [CHD7_HUMAN]	0.40	2	2	2	2			22.33	26.68			1	1			22.39	26.43			22.39	26.43	NA	NA	4.043
O15078	Centrosomal protein of 290 kDa OS=Homo sapiens GN=CEP290 PE=1 SV=2 - [CE290_HUMAN]	0.40	1	1	1	1					22.49		0	1					22.70			22.70	NA	NA	
Q8IWZ3	Ankyrin repeat and KH domain-containing protein 1 OS=Homo sapiens GN=ANKHD1 PE=1 SV=1 - [ANKH1_HUMAN]	0.39	1	1	1	1					19.59		0	1					19.81			19.81	NA	NA	
Q9H4A3	Serine/threonine-protein kinase WNK1 OS=Homo sapiens GN=WNK1 PE=1 SV=2 - [WNK1_HUMAN]	0.38	1	1	1	1				19.63			0	1				19.38				19.38	NA	NA	
Q96RT7	Gamma-tubulin complex component 6 OS=Homo sapiens GN=TUBGCP6 PE=1 SV=3 - [GCP6_HUMAN]	0.38	1	1	1	1					23.81		0	1					24.03			24.03	NA	NA	
Q9UFH2	Dynein heavy chain 17, axonemal OS=Homo sapiens GN=DNAH17 PE=1 SV=2 - [DYH17_HUMAN]	0.38	1	1	1	1						20.44	0	1						20.17		20.17	NA	NA	
Q8N7Z5	Putative ankyrin repeat domain-containing protein 31 OS=Homo sapiens GN=ANKRD31 PE=5 SV=2 - [ANR31_HUMAN]	0.37	1	1	1	1	22.70						1	0	22.87						22.87		NA	NA	
Q86U86	Protein polybromo-1 OS=Homo sapiens GN=PBRM1 PE=1 SV=1 - [PB1_HUMAN]	0.36	1	1	1	1				22.76			0	1				22.51				22.51	NA	NA	
Q8N1W1	Rho guanine nucleotide exchange factor 28 OS=Homo sapiens GN=ARHGEF28 PE=1 SV=3 - [ARG28_HUMAN]	0.35	1	1	1	4	30.96		32.13				2	0	31.13		32.19				31.66		NA	NA	
O94822	E3 ubiquitin-protein ligase listerin OS=Homo sapiens GN=LTN1 PE=1 SV=6 - [LTN1_HUMAN]	0.34	1	1	1	1					21.18		0	1					21.40			21.40	NA	NA	
Q5VWN6	Protein FAM208B OS=Homo sapiens GN=FAM208B PE=1 SV=1 - [F208B_HUMAN]	0.33	1	1	1	1						25.86	0	1						25.60		25.60	NA	NA	
Q68DK2	Zinc finger FYVE domain-containing protein 26 OS=Homo sapiens GN=ZFYVE26 PE=1 SV=3 - [ZFY26_HUMAN]	0.32	1	1	1	1				22.00			0	1				21.75				21.75	NA	NA	
P48634	Protein PRRC2A OS=Homo sapiens GN=PRRC2A PE=1 SV=3 - [PRC2A_HUMAN]	0.32	1	1	1	1		24.27					1	0		24.34					24.34		NA	NA	
Q9NR48	Histone-lysine N-methyltransferase ASH1L OS=Homo sapiens GN=ASH1L PE=1 SV=2 - [ASH1L_HUMAN]	0.30	1	1	1	1					16.12		0	1					16.33			16.33	NA	NA	
Q92614	Unconventional myosin-XVIIIa OS=Homo sapiens GN=MYO18A PE=1 SV=3 - [MY18A_HUMAN]	0.29	1	1	1	2				20.02	23.22		0	2				19.78	23.44			21.61	NA	NA	
P15924	Desmoplakin OS=Homo sapiens GN=DSP PE=1 SV=3 - [DESP_HUMAN]	0.28	1	1	1	2			22.05				1	0			22.11				22.11		NA	NA	
Q9BYW2	Histone-lysine N-methyltransferase SETD2 OS=Homo sapiens GN=SETD2 PE=1 SV=3 - [SETD2_HUMAN]	0.27	1	1	1	1	24.08						1	0	24.24						24.24		NA	NA	
Q13535	Serine/threonine-protein kinase ATR OS=Homo sapiens GN=ATR PE=1 SV=3 - [ATR_HUMAN]	0.26	1	1	1	1				22.55			0	1				22.30				22.30	NA	NA	
Q8WXH0	Nesprin-2 OS=Homo sapiens GN=SYNE2 PE=1 SV=3 - [SYNE2_HUMAN]	0.22	1	1	1	1				23.66			0	1				23.42				23.42	NA	NA	
Q8TD26	Chromodomain-helicase-DNA-binding protein 6 OS=Homo sapiens GN=CHD6 PE=1 SV=4 - [CHD6_HUMAN]	0.22	1	1	1	1						22.98	0	1						22.72		22.72	NA	NA	
Q96T58	Msx2-interacting protein OS=Homo sapiens GN=SPEN PE=1 SV=1 - [MINT_HUMAN]	0.22	1	1	1	1			26.55				1	0			26.61				26.61		NA	NA	
O14686	Histone-lysine N-methyltransferase 2D OS=Homo sapiens GN=KMT2D PE=1 SV=2 - [KMT2D_HUMAN]	0.20	1	1	1	1	20.96						1	0	21.12						21.12		NA	NA	
Q9UPA5	Protein bassoon OS=Homo sapiens GN=BSN PE=2 SV=4 - [BSN_HUMAN]	0.20	1	1	1	1				24.44			0	1				24.20				24.20	NA	NA	
Q8IZT6	Abnormal spindle-like microcephaly-associated protein OS=Homo sapiens GN=ASPM PE=1 SV=2 - [ASPM_HUMAN]	0.20	1	1	1	1					23.03		0	1					23.24			23.24	NA	NA	
Q8N3K9	Cardiomyopathy-associated protein 5 OS=Homo sapiens GN=CMYA5 PE=1 SV=3 - [CMYA5_HUMAN]	0.17	1	1	1	1	22.50						1	0	22.66						22.66		NA	NA	
Q9NYQ8	Protocadherin Fat 2 OS=Homo sapiens GN=FAT2 PE=1 SV=2 - [FAT2_HUMAN]	0.16	1	1	1	1			23.54				1	0			23.60				23.60		NA	NA	
Q9P2P6	StAR-related lipid transfer protein 9 OS=Homo sapiens GN=STARD9 PE=1 SV=3 - [STAR9_HUMAN]	0.15	1	1	1	1				18.94			0	1				18.70				18.70	NA	NA	
Q8NEZ4	Histone-lysine N-methyltransferase 2C OS=Homo sapiens GN=KMT2C PE=1 SV=3 - [KMT2C_HUMAN]	0.14	1	1	1	1			24.69				1	0			24.75				24.75		NA	NA	
Q8IVF4	Dynein heavy chain 10, axonemal OS=Homo sapiens GN=DNAH10 PE=1 SV=4 - [DYH10_HUMAN]	0.13	1	1	1	1			25.70				1	0			25.75				25.75		NA	NA	
Q9NU22	Midasin OS=Homo sapiens GN=MDN1 PE=1 SV=2 - [MDN1_HUMAN]	0.11	1	1	1	1					23.33		0	1					23.55			23.55	NA	NA	
Q8NF91	Nesprin-1 OS=Homo sapiens GN=SYNE1 PE=1 SV=4 - [SYNE1_HUMAN]	0.08	1	1	1	1						24.71	0	1						24.45		24.45	NA	NA	
							23.93																		
