# Supplementary material for: ITGB3-mediated uptake of small extracellular vesicles facilitates intercellular communication in breast cancer cells
Source: Nat Commun. 2020 Aug 26;11:4261. doi: 10.1038/s41467-020-18081-9 (PMC7450082; doi:10.1038/s41467-020-18081-9)
Supplement: Supplementary file 5 — Reporting Summary [file 41467_2020_18081_MOESM5_ESM.pdf]

nature research

Corresponding author(s): Stefan Hümmer, Santiago Ramón y Cajal

Last updated by author(s): Jul 20, 2020

Reporting Summary

Nature Research wishes to improve the reproducibility of the work that we publish. This form provides structure for consistency and transparency in reporting. For further information on Nature Research policies, see our Editorial Policies and the Editorial Policy Checklist.

Statistics

For all statistical analyses, confirm that the following items are present in the figure legend, table legend, main text, or Methods section.

n/a	Confirmed	
☐	☒	The exact sample size (n) for each experimental group/condition, given as a discrete number and unit of measurement
☐	☒	A statement on whether measurements were taken from distinct samples or whether the same sample was measured repeatedly
☐	☒	The statistical test(s) used AND whether they are one- or two-sided<br>*Only common tests should be described solely by name; describe more complex techniques in the Methods section.*
☒	☐	A description of all covariates tested
☒	☐	A description of any assumptions or corrections, such as tests of normality and adjustment for multiple comparisons
☐	☒	A full description of the statistical parameters including central tendency (e.g. means) or other basic estimates (e.g. regression coefficient) AND variation (e.g. standard deviation) or associated estimates of uncertainty (e.g. confidence intervals)
☐	☒	For null hypothesis testing, the test statistic (e.g. F, t, r) with confidence intervals, effect sizes, degrees of freedom and P value noted<br>*Give P values as exact values whenever suitable.*
☒	☐	For Bayesian analysis, information on the choice of priors and Markov chain Monte Carlo settings
☒	☐	For hierarchical and complex designs, identification of the appropriate level for tests and full reporting of outcomes
☒	☐	Estimates of effect sizes (e.g. Cohen's d, Pearson's r), indicating how they were calculated

Our web collection on statistics for biologists contains articles on many of the points above.

Software and code

Policy information about availability of computer code

Data collection	Methods section of the manuscript contains detailed information of all the softwares used for data collection.<br>XCalibur software v2.2, Imaris software (7.2.3), Proteome Discoverer software suite (v1.4) Mascot search engine (v2.5), FACSDiva (v8.0.1), Digital Micrograph software package (Gatan). G*Power Version 3.1.0.6 (Heinrich-Heine-Universität Düsseldorf)
Data analysis	Microsoft Excel 2017 and Graphad PRISM 6.0 .1 was used to perform data analysis including statistical analysis.<br>ImageJ/Fiji 64bit JAVA 1.8.0_112 and Zeiss Zen 3.0 was used to perform images analysis.<br>FACSDiva (v8.0.1) and FCS Express 4 Flow Research was used to perform Flow cytometry analysis.<br>NTA version 3.1 Build 3.1.45 (Nanosight NS-300) was used to perform real-time characterization of the vesicles.<br>Chromatographic and mass spectrometric analysis were done using an LTQ-Orbitrap Velos Pro mass spectrometer coupled to an EASYnLC.<br>Acquired spectra were analysed using the Proteome Discoverer software suite (v1.4, Thermo Fisher Scientific) and the Mascot search engine (v2.5, Matrix Science).

For manuscripts utilizing custom algorithms or software that are central to the research but not yet described in published literature, software must be made available to editors and reviewers. We strongly encourage code deposition in a community repository (e.g. GitHub). See the Nature Research guidelines for submitting code & software for further information.

Data

Policy information about availability of data

All manuscripts must include a data availability statement. This statement should provide the following information, where applicable:

- Accession codes, unique identifiers, or web links for publicly available datasets
- A list of figures that have associated raw data
- A description of any restrictions on data availability

The data were searched against a Swiss-Prot human database plus a list of common contaminants.
The mass spectrometry raw data have been deposited into the PRIDE repository with the dataset identifier PXD013489 (https://www.ebi.ac.uk/pride/archive/projects/PXD013489). All the data supporting the findings of this study are available within the article and its supplementary information files and from the corresponding author upon reasonable request. A reporting summary for this article is available as a Supplementary Information file. The source data underlying Figures 1–6 and Supplementary Figures 1–1811 are provided as a Source Data file.
and all the corresponding decoy entries.

Field-specific reporting

Please select the one below that is the best fit for your research. If you are not sure, read the appropriate sections before making your selection.

☒ Life sciences ☐ Behavioural & social sciences ☐ Ecological, evolutionary & environmental sciences

For a reference copy of the document with all sections, see nature.com/documents/nr-reporting-summary-flat.pdf

Life sciences study design

All studies must disclose on these points even when the disclosure is negative.

Sample size	Animal size for the experiments in Figure S1, was calculated following G*Power Version 3.1.0.6 (Heinrich-Heine-Universität Düsseldorf). Our previous experiments (Sese et al. Oncotarget, 2017), revealed that the number of metastasis caused by tail vein injected shITGB3 MDA-MB231 cells is reduced to approximately 50% of control cells. We determined now that after overnight injection, the lung tissue contains about 7% of control MDA-MB231 cells (standard deviation of 3%). Considering that a defect of homing in lung tissue is the cause for the reduced metastasis of shITGB3 cells, a reduction to about 3.5% (=50% of control cells) with a p Value<0.05 should be expected. With these parameters and using a t-test (Means: difference between two different means (two groups)) the sample size was determined with eight mice per group .<br>All other results are obtained from in vitro experiments, for which the sample size is not limited and at least three independent experimental replicates were performed. For those experiments, we followed the general advice of the Statistics and Bioinformatic Unit.
Data exclusions	No data was excluded.
Replication	All experiments were repeated at least three times with reproducibility, except for those experiments which are also noted in the figure legends, that were repeated more than 3 times.<br>The replication number is indicated in the legend of the corresponding figure.
Randomization	Mice experimentations: For the in vivo experiments, mice were previously randomized into experimental groups.<br>Cell line experimentation: For each experiment, the total amount of cells from one cell line required for all tested conditions were pooled and seeded randomly into different plates, pre-labeled with the treatment to be applied.
Blinding	For the imaging experiments, including CryoEM and Confocal microscopy, where manual counting was required, samples were labeled with numbers in order to avoid preconceptions of the analyzing investigator. Automated software was used for the quantification of mass-spectrometry, NTA and FACS analysis, precluding bias of the investigator. If other than the default settings of a software was used, the parameters are specified in the respective methodology section. The remaining experiments were analyzed by western blotting. In this case, codification was maintained during sample preparation. Decoding was done before loading the samples on the SDS-PAGE gel, in order to ensure an adequate presentation of the results.

Reporting for specific materials, systems and methods

We require information from authors about some types of materials, experimental systems and methods used in many studies. Here, indicate whether each material, system or method listed is relevant to your study. If you are not sure if a list item applies to your research, read the appropriate section before selecting a response.

Materials & experimental systems

n/a	Involved in the study	
☐	☒	Antibodies
☐	☒	Eukaryotic cell lines
☒	☐	Palaeontology and archaeology
☐	☒	Animals and other organisms
☒	☐	Human research participants
☒	☐	Clinical data
☒	☐	Dual use research of concern

Methods

n/a	Involved in the study	
☒	☐	ChIP-seq
☐	☒	Flow cytometry
☒	☐	MRI-based neuroimaging

Antibodies

Antibodies used

The following primary antibodies were used:
anti-CD81 (Santa Cruz Biotechnology), Anti-CD81 Antibody (B-11) is a mouse monoclonal IgG2b (kappa light chain). sc-166029
anti-pFAK (Santa Cruz Biotechnology), p-FAK Antibody (2D11) is a mouse monoclonal IgG1 (kappa light chain). sc-81493
anti-FAK (Santa Cruz Biotechnology), Anti-FAK Antibody (D-1) is a mouse monoclonal IgG1 (kappa light chain). sc-271126
anti-DYNAMIN 2 (Santa Cruz Biotechnology), Anti-Dynamin II Antibody (G-4) is a mouse monoclonal IgG3 (kappa light chain) . sc-166669.
anti-LAMP1 (Santa Cruz Biotechnology) WB and immunoflorescence, Anti-LAMP-1 Antibody (H4A3) is a mouse monoclonal IgG1 (kappa light chain). sc-20011.
anti-ERK (Santa Cruz Biotechnology); ERK 2 Antibody (C-14) is a rabbit polyclonal IgG. sc-1647
anti-Vinculin (Sigma-Aldrich); Monoclonal Anti-Vinculin antibody produced in mouse clone hVIN-1. V9131
anti-TSG101, Anti-TSG101 antibody [4A10] (ab83).
anti-αv integrin, Recombinant Anti-Integrin alpha V antibody [EPR16800] (ab179475).
anti-β1 integrin (Abcam); Recombinant Anti-Integrin beta 1 antibody [EPR16895] (ab179471).
anti-β3 integrin,
WB application: Integrin β3 Antibody #4702. (Cell Signaling)
IF/FACS application: MAB1976 Sigma-Aldrich Anti-Integrin αVβ3 Antibody, clone LM609.
anti-pERK (Cell Signaling), Phospho-p44/42 MAPK (Erk1/2) (Thr202/Tyr204) Antibody #9101.
anti-pSrc (Cell Signaling), Phospho-Src Family (Tyr416) Antibody #2101
anti-Src (Cell Signaling), Src (36D10) Rabbit mAb #2109.
anti-pAKT (Cell Signaling), Phospho-Akt (Ser473) (D9E) XP® Rabbit mAb #4060.
anti-AKT (1:1000),
anti-EEA1 (Cell Signaling), WB and IF application. EEA1 (C45B10) Rabbit mAb #3288
and anti-EIF4E (Cell Signalling); eIF4E (C46H6) Rabbit mAb #2067.
anti-flotillin-1 (Novus Biologicals); Flotillin-1 polyclonal Antibody NB100-1043.
and anti-β-actin. Mouse monoclonal anti-beta-actin-HRP conjugated clone (AC-15) SIGMA Cat# A3854 RRID:AB_262011
CD45 Monoclonal Antibody (30-F11), FITC, eBioscience™.
anti-tubulin (DM1a-FTIC, Sigma), mouse monoclonal, F2168. IF application.

Validation

Antibodies were chosen based on previous literature. Validation and quality control is available from the manufacturers using the catalog number of each antibody. When necessary, additional validations were performed in our laboratory (Western Blotting, flow cytometry, immunofluorescence) using siRNA- or shRNA- treated cells for the depletion of the targeted protein.

CD81 (B-11) is recommended for detection of CD81 of mouse, rat and human origin by Western Blotting (starting dilution 1:100, dilution range 1:100-1:1000), immunoprecipitation [1-2 µg per 100-500 µg of total protein (1 ml of cell lysate)], immunofluorescence (starting dilution 1:50, dilution range 1:50-1:500), immunohistochemistry (including paraffin-embedded sections) (starting dilution 1:50, dilution range 1:50-1:500) and solid phase ELISA (starting dilution 1:30, dilution range 1:30-1:3000).

p-FAK Antibody (A-12) is rrecommended for detection of Tyr 397 phosphorylated FAK of mouse, rat, human and canine origin by WB and IP. Additional validation was performed in our laboratory by WB using siRNA against FAK protein and using FAK protein inhibitor FAK14.

Anti-FAK Antibody (D-1) is recommended for detection of FAK p125 of mouse, rat and human origin by WB, IP, IF, IHC(P) and ELISA; also reactive with additional species, including and equine, canine and porcine. Specific for an epitope mapping between amino acids 2-31 at the N-terminus of FAK of human origin. Additional validation was performed in our laboratory by WB using siRNA against FAK protein.

Anti-Dynamin II Antibody (G-4) specific for an epitope mapping between amino acids 853-870 at the C-terminus of Dynamin II of human origin is recommended for detection of Dynamin II of mouse, rat and human origin by WB, IP, IF and ELISA; also reactive with additional species, including and equine, bovine, porcine and canine; may cross-react with Dynamin III. Additional validation was performed in our laboratory by WB using shRNA against Dynamin 2 protein and Dynamin 2 plasmid transfection.

Anti-LAMP-1 Antibody (H4A3) is recommended for detection of LAMP-1 of mouse, rat and human origin by WB, IP, IF, IHC(P) and FCM; also reactive with additional species, including monkey.

ERK 2 Antibody (D-2) specific for an epitope mapping between amino acids 345-358 at the C-terminus of ERK 2 of human origin is recommended for detection of ERK 2 p42 of mouse, rat, human and avian origin by WB, IP, IF, IHC(P), FCM and ELISA; also reactive with additional species, including and canine, bovine, porcine and avian.

Monoclonal Anti-Vinculin may be used for the localization of human and animal vinculin using various immunoassays including immunocytochemical localization by means of immunofluorescence labeling of cultured cells, immunohistological staining of frozen tissue sections, and immunoblotting

anti-TSG101 antibody recognizes the TSG-101 protein, the product of a recently identified tumor susceptibility gene the inactivation of which in mouse fibroblasts results in cell transformation and the ability of those cells to form tumors in nude mice. Suitable for: Flow Cyt, ICC/IF, WB, IHC-P.

Recombinant Anti-Integrin alpha V antibody [EPR16800] (ab179475). Recombinant fragment within Human Integrin alpha V aa 1-250. The exact sequence is proprietary. Suitable for: WB, IP, ICC/IF, IHC-P. Reacts with: Mouse, Rat, Human

Recombinant Anti-Integrin beta 1 antibody [EPR16895] (ab179471). KO validated. Recombinant fragment within Human Integrin beta 1 aa 1-250. The exact sequence is proprietary. Suitable for: Flow Cyt, WB, IHC-P

anti-β3 integrin:
WB application: Integrin β3 Antibody #4702. Integrin beta3 Antibody detects endogenous levels of total integrin beta3. This antibody does not cross-react with other integrin isoforms. Species Reactivity: Human, Mouse, Rat, Monkey, Bovine. Additional validation was performed in our laboratory by WB using shRNA against ITGB3 protein.
IF/FACS application: MAB1976 Sigma-Aldrich Anti-Integrin αVβ3 Antibody, clone LM609. Anti-Integrin αVβ3 Antibody, clone LM609 is an antibody against Integrin αVβ3 for use in FC, IF, IH and IP and has published in over 60 citations. Additional validation was performed in our laboratory by IF using shRNA against ITGB3 protein.

Phospho-p44/42 MAPK (Erk1/2) (Thr202/Tyr204) Antibody detects endogenous levels of p44 and p42 MAP Kinase (Erk1 and Erk2) when phosphorylated either individually or dually at Thr202 and Tyr204 of Erk1 (Thr185 and Tyr187 of Erk2). The antibody does not cross-react with the corresponding phosphorylated residues of either JNK/SAPK or p38 MAP Kinase, and does not cross-react with non-phosphorylated Erk1/2. Species Reactivity: Human, Mouse, Rat, Hamster, Monkey, Mink, D. melanogaster, Zebrafish, Bovine, Pig, C. elegans

Phospho-Src Family (Tyr416) Antibody detects endogenous levels of Src only when phosphorylated at tyrosine 416. The antibody may cross-react with other Src family members (Lyn, Fyn, Lck, Yes and Hck) when phosphorylated at equivalent sites. It does not cross-react with Src phosphorylated at tyrosine 527. It may cross react with phosphorylated RTKs. Species Reactivity: Human, Mouse, Rat

Src (36D10) RmAb detects endogenous levels of Src proteins. This antibody cross-reacts with over-expressed levels of Yes and Fyn but does not cross-react with other Src family members. Species Reactivity: Human, Mouse, Rat, Hamster, Monkey, Bovine, Pig

Phospho-Akt (Ser473) (D9E) XP® Rabbit mAb detects endogenous levels of Akt only when phosphorylated at Ser473. Species Reactivity: Human, Mouse, Rat, Hamster, Monkey, D. melanogaster, Zebrafish, Bovine

EEA1 (C45B10) Rabbit mAb detects endogenous levels of total EEA1 protein. Species Reactivity: Human, Mouse, Rat

eIF4E (C46H6) Rabbit mAb detects endogenous levels of total eIF4E protein. Species Reactivity: Human, Mouse, Rat, Monkey. Additional validation was performed in our laboratory by WB using drug inhibitor treatment targeting eIF4E.

anti-flotillin-1 (Novus Biologicals); Flotillin-1 Antibody NB100-1043. Immunogen Peptide with sequence C-SISQVNHKPLRTA corresponding to C-Terminus according to NP_005794.1. Confirmed species: Human. Tested in WB application.

The 30-F11 monoclonal antibody reacts with all isoforms of mouse CD45, also known as Leukocyte Common Antigen (LCA). CD45 is expressed by all hematopoietic cells excluding mature erythrocytes and platelets. The cytoplasmic portion of CD45 has tyrosine phosphatase enzymatic activity and plays an important role in activation of lymphocytes. Applications Reported: The 30-F11 antibody has been reported for use in flow cytometric analysis. Applications Tested: The 30-F11 antibody has been tested by flow cytometric analysis.

Monoclonal Anti-α-Tubulin antibody is suitable for immunostaining of:
• respiratory epithelium tissue in a study to determine the tubulin expression in the mice cilia
• breast cancer tissue sections to study the effect of LMO4 on the centrosome amplification and mitotic spindle abnormalities
• spindle and chromosomes of oocytes
Mouse monoclonal antibody has been used in the:
• detection of tubulin by immunofluorescence and confocal microscopy in lung carcinoma cell.
• immunofluorescent staining of microtubules in human embryos and mitotic spindles from spleen lymphoblast.

Eukaryotic cell lines

Policy information about cell lines

Cell line source(s)	MCF7, MDA.MB.21 and IMR90 Cell lines were purchased from the American Type Culture Collection (ATCC).
Authentication	Authenticated by DNA profiling using short tandem repeat (GenePrint® 10 System, Promega) at Genomics Core Facility, Instituto de Investigaciones Biomédicas Alberto Sols CSIC-UAM.
Mycoplasma contamination	All cell lines used in this study were negative for mycoplasma contamination. Mycoplasma contamination was monthly tested using a PCR assay.
Commonly misidentified lines (See ICLAC register)	No commonly misidentified cell lines were used in the study.

Animals and other organisms

Policy information about studies involving animals; ARRIVE guidelines recommended for reporting animal research

Laboratory animals	Hsd: Athymic Nude-Foxn1nu. Female, 4-6 weeks.
Wild animals	This study did not involve wild animals.
Field-collected samples	None
Ethics oversight	All animal experiments were reviewed and approved by the ethics committee of the "Vall d'Hebron Research Institute (VHIR)" Barcelona, Spain (76/17 CEEA).

Note that full information on the approval of the study protocol must also be provided in the manuscript.

Flow Cytometry

Plots

Confirm that:

☒ The axis labels state the marker and fluorochrome used (e.g. CD4-FITC).

☒ The axis scales are clearly visible. Include numbers along axes only for bottom left plot of group (a 'group' is an analysis of identical markers).

☒ All plots are contour plots with outliers or pseudocolor plots.

☒ A numerical value for number of cells or percentage (with statistics) is provided.

Methodology

Sample preparation	Cells were detached using enzyme-free PBS-based Cell Dissociation Buffer (Gibco). Cells were washed in PBS solution, suspended in FACS buffer (EDTA 2.5 mM, 1% BSA in PBS).
Instrument	FACSCalibur instrument.
Software	FACSDiva (BD Biosciences)
Cell population abundance	Between 10,000 and 100,000 cells were acquired per sample, and the total population was analysed.
Gating strategy	From the starting cell population, the cells were gated by: FSC-H/FSC-A; SSCA/FSC-A; F VIOLET-A/FSC-A; C RED-A/FSC-A for uptake experiments, where PKH26 Red fluorescent signal was analyzed.<br>From the starting cell population, the cells were gated by: FSC-H/FSC-A; SSCA/FSC-A; F VIOLET-A/FSC-A; C RED-A/B BLUE-A for cell surface protein experiments, where GFP expression was analyzed.<br>Unlabelled cells were used as negative control for measurement of background signal.

☒ Tick this box to confirm that a figure exemplifying the gating strategy is provided in the Supplementary Information.
